# *Giardia lamblia* miRNAs as a new diagnostic tool for human giardiasis

**DOI:** 10.1371/journal.pntd.0007398

**Published:** 2019-06-17

**Authors:** Tal Meningher, Daniel Boleslavsky, Iris Barshack, Hila Tabibian-Keissar, Refael Kohen, Devorah Gur-Wahnon, Iddo Z. Ben-Dov, Yechezkel Sidi, Dror Avni, Eli Schwartz

**Affiliations:** 1 Laboratory of Molecular Cell Biology, Sheba Medical Center, Ramat Gan, Israel; 2 Department of Medicine C, Sheba Medical Center, Ramat Gan, Israel; 3 Molecular Laboratory for the Study of Tropical Diseases, Sheba Medical Center, Ramat Gan, Israel; 4 Department of Pathology, Sheba Medical Center, Ramat Gan, Israel; 5 Faculty of Medicine, Sackler School of Medicine, Tel Aviv University, Tel Aviv, Israel; 6 The Mina and Everard Goodman Faculty of Life Sciences, Bar-Ilan University, Ramat Gan, Israel; 7 Israel National Center for Personalized Medicine, Weizmann Institute of Science, Rehovot, Israel; 8 Laboratory of Medical Transcriptomics, Nephrology and Hypertension Services, Hadassah—Hebrew University Medical Center, Jerusalem, Israel; 9 The Center for Geographic Medicine, Sheba Medical Center, Ramat Gan, Israel; London School of Hygiene and Tropical Medicine, UNITED KINGDOM

## Abstract

**Background:**

*Giardia lamblia* is a very common cause of gastrointestinal symptoms worldwide. There are several methods for the diagnosis of *Giardia* infection, however none are ideal. We aim to find a new, microRNA-based method that will improve the currently available diagnostic methods for giardiasis.

**Methods:**

Deep-sequence profiling of *Giardia* small-RNA revealed that miR5 and miR6 are highly expressed in *Giardia*. These miRNAs were tested by qRT-PCR in duodenal biopsies of patients with giardiasis who were positive by microscopic pathological evaluation. The gastric biopsies of the same patients served as negative control tissues. Additionally, these miRNAs were evaluated in stool samples of patients with proven giardiasis.

**Results:**

All histologically proven duodenal biopsies of patients with *Giardia* infection were positive for *Giardia* miR5, with a mean threshold cycle (Ct) of 23.7, as well as for *Giardia* DNA qPCR (16S-like gene, mean Ct 26.3). Gastric biopsies which were tested as a control all were negative. Stool evaluation of miR6 in patients with giardiasis showed 90% specificity but only 66% sensitivity, and a lower accuracy rate was obtained with miR5.

**Conclusion:**

*Giardia* miR5 testing in duodenal biopsies may be a new method for the diagnosis of giardiasis. It seems to be more sensitive when compared with testing for *Giardia* DNA by qPCR in duodenal biopsies. It will be important to investigate the contribution of routine *Giardia* miRNA testing in duodenal biopsies from patients with persistent abdominal symptoms

## Author summary

Giardiasis is a major cause of diarrheal disease throughout the world. It is more common in areas with poor sanitation such as in many low-income countries, but it occurs in high-income countries as well. It is the most commonly identified intestinal parasite in the United States and it is endemic in other industrialized countries. The causative agent is the flagellate protozoan *Giardia lamblia*, and transmission is mainly by the fecal-oral route. The basic method of diagnosis is stool examination. It is usually found through stool microscopy examination which should be performed on fresh stool and repeated in 3 days. Despite some newer diagnostic methods, *Giardia* is still difficult to detect, often leading to misdiagnoses.

In this study we show that using *Giardia* microRNA (miR5) as a marker for *Giardia* infection in duodenal biopsies may be a new method for diagnosis of giardiasis. It appears to be more sensitive than histological diagnosis and also more sensitive than *Giardia* DNA testing in duodenal biopsies. Interestingly, in our patients, duodenal biopsies were done for persistent abdominal symptoms and the finding of *Giardia* in their biopsy was unexpected. Thus, testing duodenal biopsies for *Giardia* miRNA in patients with persistent abdominal symptoms might contribute to diagnosis and prompt treatment for those with giardiasis.

## Introduction

*Giardia lamblia* (also known as *G*. *intestinalis* or *G*. *duodenalis*) is the causative agent of giardiasis. Among the intestinal protozoan parasites, it is one of the three most common agents of diarrhea worldwide [1]. *Giardia* species have two life cycle stages; the flagellated trophozoite that attaches to the intestinal microvilli and an infectious cyst that persists in the environment [2]. Transmission to humans occurs either through direct person-to-person contact in environments with compromised hygiene levels [3] or by the contamination of water or food by the cysts, the resistant form. In industrialized countries, *Giardia* is the most common parasite identified in stool samples [4, 5]. Symptoms include diarrhea, flatulence, excessive fatigue, nausea, foul smelling stools, abdominal cramps and weight loss. In about 16% of patients the disease may become chronic, with long-term effects such as loose stools, malnutrition, growth delays, cognitive impairment, abdominal pain, malabsorption and malaise [6, 7].

In travelers returning from tropical countries, persistent abdominal symptoms (PAS) including chronic diarrhea, abdominal pain, flatulence and fatigue are common [8]. Unfortunately, in most cases, the etiology of these complaints remains unknown, partially due to the low sensitivity of current tests. In only about a third of the cases pathogens can be identified, the most common being *Giardia* [9]. Although the majority of returning travelers with chronic complaints remain undiagnosed, many of them nonetheless respond to anti-parasitic treatment [8].

Currently, there are several diagnostic methods for *Giardia*, but none serves as a real gold- standard. Classically, laboratory diagnosis is performed by microscopic examination of stool samples, ‘‘ova and parasite examination” (O&P) [10]. The recommendation is to collect at least three independent, preferably watery stool specimens to maximize the sensitivity of the detection. Moreover, the stool should be fresh because the trophozoites break down very quickly [10]. Direct fluorescent antibody assay and stool antigen detection using enzyme-linked immunosorbent assay (ELISA) have been accepted as more sensitive tools for diagnosis of giardiasis and have provided a potentially attractive alternative to conventional O&P examinations [11, 12]. However, studies have shown that at least a pair of stool samples is needed for sufficient sensitivity [11]. Furthermore, in recent years, PCR-based nucleic acid detection methods have shown higher sensitivity compared to microscopy and antigen detection tests [13]. Since *Giardia* parasites reside in the small intestines, searching for it in the duodenum seems reasonable [14]. A prospective study done in Italy has found giardiasis through direct histological examination of duodenal biopsy specimens in 9 of the 137 patients (6.5%) with symptoms consistent with irritable bowel syndrome (IBS) and no alarming signs [15].

miRNAs are small non-coding regulatory RNAs that can direct post-transcriptional repression of protein synthesis from mRNAs containing miRNA binding sites. In animals, miRNAs have diverse biological functions, including regulation of key aspects of development and life cycles [16]. These molecules, are also found in unicellular organisms, including *Giardia* species [17] (although the existence of canonical miRNA in these primitive eukaryotes is under debate [18], being that their genome lacks orthologs of the miRNA processing genes *DROSHA* and *XPO5* [19]), and as potential markers have the advantage of being in part genus specific [20, 21] and relatively stable. It was shown that miRNAs are resistant to freeze-thaw cycling, RNase A digestion, and treatment with a high pH solution [22]. In addition, miRNAs were shown to be stable in FFPE specimens [23] and in feces [24]. Therefore, we attempted to identify *Giardia* in positive duodenal biopsies and stool samples of proven giardiasis patients through detecting *Giardia* miRNAs by quantitative reverse-transcription-PCR.

## Materials and methods

### Ethics statement

The study was approved by the Sheba Medical Center ethics Committee (Helsinki), protocol number 1335-14-SMC. The FFPE sample were taken from the archives of the Department of Pathology at Sheba Medical Center. The requirement for informed consent was waived due to the archived nature of the study FFPE specimens. The control newborn fecal samples were collected after the mothers were informed and provided consent. Anonymized frozen fecal samples of *Giardia*-infected patients were provided by the Helsinki Committee-approved repositories of the parasitology laboratory of the Israeli Ministry of Health and the microbiology laboratory at Sheba Medical Center.

***Giardia* parasite strains** *G*. *lamblia* trophozoite were obtained from the BEI Resources (https://www.beiresources.org/Home.aspx). WB clone 6 (NR-9706, assemblage A [25]), Egypt-4 (NR-9231, assemblage A), Mario (NR-9232, assemblage A), Sug (NR-9233, assemblage not determined) and G2M (NR-9232, assemblage not determined).

### *Giardia* growing conditions

*Giardia* cells were grown in Keister's Modified TYI-S-33 [26], which contains: 2% casein, 1% yeast extract, 1% glucose, 0.2% NaCl, 0.2% L-Cystein, 0.02% L(+)-ascorbic acid, 0.1% K_2_HPO_4_·3H_2_O, 0.06% KH_2_PO_4_, 0.00228% ammonium iron (III) citrate 1% bovine bile solution (all from sigma) and 10% bovine calf serum (Gibco), in double distilled water (DDW). The trophozoites were grown in a polystyrene cell culture tube (Greiner bio-one, Cellstar, Cat. No. 163–160) at 37°C under anaerobic conditions.

### RNA extraction from trophozoites and miRNA profiling via small RNA sequencing

Total RNA were extracted from one million trophozoite cells of each *Giardia* isolate using mirVana miRNA isolation kit (Ambion, AM1561). Barcoded cDNA libraries of small RNA (19–35 nt) were prepared from the total RNA using an in-house method, as previously described [27]. Libraries were deep-sequenced on an Illumina sequencer (HiSeq 2500). Additionally, in order to facilitate miRNA discovery which requires identification of passenger strand reads, that are typically rare compared to the mature strand, we subjected the cDNA libraries to cleavage with a duplex-specific nuclease (DSN, Evrogen, cat. #EA003) [28] and sequenced the cleaved libraries as well. Resulting FASTQ read files were processed as described [29], and demultiplexed data were deposited at NCBI’s gene expression omnibus (GEO record GSE116101).

### Bioinformatics analysis

Discovery of miRNA in the demultiplexed libraries was performed using the miRDeep2 algorithm [30]. For this purpose, we combined the intact and DSN-cleaved cDNA library FASTQ files. We allowed reads up to length 29 nt to be included in the analysis, as opposed to the default 25 nt, because a previous investigation of Argonaute-associated small RNAs in *Giardia* reported a mode length of 26 nt [17], concordant with structural modeling of *Giardia* Dicer [31]. Previously reported putative *Giardia* miRNA (**S1 Table**, adapted from Liao et al [17–19, 32–37]), were specifically sought, by including them as presumed known mature miRNA in the miRDeep2 analysis. For genome mapping, we downloaded the GiardiaDB-37_GintestinalisAssemblageAWB_Genome.fasta file (2018-04-19) from GiardiaDB.

### Patient samples

FFPE duodenal biopsy tissue blocks from histopathology proven giardiasis patients were obtained from the Department of Pathology, Sheba Medical Center. In addition, gastric biopsies from the same patients and from unrelated patients were used as negative controls. Fecal samples from microscopy and/or antigen proven giardiasis patients were collected from the Institute of Geographic Medicine and Tropical Diseases, Sheba Medical Center, and from the Parasitology Laboratory of the Israeli Ministry of Health, Jerusalem. Negative control fecal samples were collected from newborns and from infants that had just started eating but are still not attending day-care. Stool samples were stored at -80°C until analysis.

### DNA and RNA extraction from FFPE

DNA was extracted from 10–20 slices of FFPE tissue blocks by the QIAamp DNA Mini Kit (Qiagen, 51304) according to the manufacturer’s protocol with minor changes. Proteinase K incubation was performed overnight and then additional proteinase K was added for 1 hour. After adding AL buffer and ethanol, the samples were incubated in -20°C for 1 hour. Subsequent steps were according to protocol. Isolation of RNA from FFPE biopsies was performed using miRNeasy FFPE Kit (Qiagen, 217504) according to the manufacturer’s protocol.

### RNA extraction from human stool samples

0.1g of stool was added to 200 μl of 2% polyvinylpolypyrolidone (PVPP, Sigma) suspension and frozen in -20°C overnight. Subsequently, the suspension was heated for 10 min at 100°C [1]. Isolation of miRNA from the suspension was performed using mirVana miRNA isolation kit (Ambion, AM1561) according to the manufacturer’s protocol.

### Real time qRT-PCR/qPCR

The ABI Quant Studio6 Flex Real-Time PCR System (Applied Biosystems, Foster City, CA, USA) was used in all qRT-PCR and qPCR experiments.

### One step qRT-PCR

Detection of miRNAs, isolated from fecal samples, by Eva Green technology, was performed as previously described [38, 39].

### Primers for *Giardia* miRNA detection in fecal samples

Giardia miR5

RP1-miR5: 5'-GGACGGTAGCAAGCAAAGAGAGAGAAGGCTCGGACAT-3'

RP2-miR5:5'-GGGATTCTGGAAGATGATGATGACGATGCTTCCTTGG-3'

P1: 5'-GGACGGTAGCAAGCAAAGAGAGAG-3'

P2: 5'-GGGATTCTGGAAGATGATGATGAC-3'

Giardia miR6:

RP1-miR6: 5'-GGACGGTAGCAAGCAAAGAGAGAGCAGAATACGACAAA-3'

RP2-miR6: 5'-GGGATTCTGGAAGATGATGATGACGACGCGTGACGAAG'-3'

P1: 5'-GGACGGTAGCAAGCAAAGAGAGAG-3'

P2: 5'-GGGATTCTGGAAGATGATGATGAC-3'

Human RNU6B:

RP1-RNU6B: 5'-GGACGGTAGCAAGCAAAGAGAGAGAAAAATATGGAACGCTTCACGAA-3'

RP2-RNU6B: 5'-GGGATTCTGGAAGATGATGATGACCGCAAGGATGACACGCAAA-3'

P1: 5'-GGACGGTAGCAAGCAAAGAGAGAG-3'

P2: 5'-GGGATTCTGGAAGATGATGATGAC-3'

### TaqMan microRNA qRT- PCR

qRT-PCR of miRNAs isolated from FFPE samples was done according to manufacturer’s protocol (Applied Biosystems), using custom primers designed according to the Applied Biosystems miRNA quantification method [40], for either Giardia miR5 (Applied Biosystems Assay-ID 5737335_1) or Giardia miR6 (Applied Biosystems Assay-ID 5710263_1).

### TaqMan DNA qPCR

qPCR of DNA isolated from FFPE samples, was done with specific primers to *Giardia* small subunit ribosomal (16S-like) RNA gene by TaqMan qPCR as described by Verweij et al. [1].

### Statistics

Statistical significance was evaluated using Student’s t-test or One-way ANOVA. A probability value of *p*< 0.05 was considered significant. For the comparison of miR5 Ct with DNA Ct, paired student t-test was use. Unpaired t-test and area under the Receiver Operating Characteristic (ROC) curve and the cutoff Ct were computed using GraphPad Prism version 5.00 for Windows, GraphPad Software, San Diego California USA, www.graphpad.com. Briefly, after entering Ct values (infected and healthy control), a series of cutoff values is proposed. The graph plots percentage sensitivity versus percentage of false positive rate (100-specificity) for the different cutoff points. The optimal cutoff is determined as the cutoff with the highest likelihood ratio [defined as %sensitivity / (100-%specificity)]. ROC curve data is presented in S2 and S3 Tables.

## Results

### Characterization of *Giardia* miRNAs

Five different *Giardia* isolates were grown. Total RNA was extracted and small RNA libraries were prepared and deep-sequenced. Results and insights from this small RNA transcriptome analysis are presented in the **S1 Appendix**. Our deep-sequencing data confirmed the presence of reads arising from two putative miRNAs that have been previously reported as having highest expression levels in *Giardia* trophozoites, miR5 and miR6 [17], and these were used for subsequent patient-sample analyses. In addition, these two miRNAs are conserved between the three main assemblages infecting humans; A, B and E. miR5 is 26 nt long and 100% identical in all three assemblages. miR6 is 28 nt long and 100% identical in assemblages A and E and 24 out of the 28 are identical in assemblage B (S1 Table).

### Detection of *Giardia* miRNAs in human duodenal biopsies

Eight duodenal biopsies that were determined as *Giardia* positive by the pathologist were studied. Samples were from patients referred to gastroscopy due to prolonged gastrointestinal system complaints or anemia (Table 1) but had not been diagnosed with giardiasis prior to gastroscopy. RNA and DNA were extracted from biopsy material and analyzed using TaqMan® MicroRNA Assays and TaqMan qPCR DNA Assay, respectively. As can be seen in Table 1, all samples were positive for *Giardia* miR5 with mean threshold cycle (Ct) value of 23.7. miR6 was mostly undetectable and thus not further examined in duodenal biopsies. We also performed a qPCR test for *Giardia* DNA using specific primers to *Giardia* small subunit ribosomal (16S-like) RNA gene by TaqMan qPCR as described by Verweij et al. [1]. Comparison of the Ct levels of *Giardia* miR5 vs. *Giardia* DNA on the same samples, using equivalent volumes of the extracted nucleic acid, shows that miR5 yielded lower mean Ct values, 23.7 versus 26.3 (by paired t-test p = 0.004). These findings suggest that the miRNA amplification may be more robust than DNA amplification.

**Table 1:** miRNA quantification in duodenal and gastric biopsies of positive *Giardia* patients detected in their duodenum by histological examination.

	Age	Sex	Medical history	Biopsy pathology	Ct miR6 duodenum	Ct miR5 duodenum	Ct DNA	Ct miR5 stomach
1	17	F	Iron deficiency anemia	Positive for *Giardia*	33.7	22.7	26.7	39
2	38	M	Iron deficiency anemia and weight loss in CVID patient	Positive for *Giardia*	34.0	21.2	26.0	>40
3	46	M	Iron deficiency and hemorrhoids	Positive for *Giardia*	34.0	32.9	33.8	-
4	56	M	Iron deficiency in ITP patient	Positive for *Giardia*	31.7	21.1	26.0	-
5	46	M	Chronic abdominal pain	Positive for *Giardia*	ND	25.7	27.6	-
6	26	F	Chronic abdominal pain	Positive for *Giardia*	ND	21.5	23.3	-
7	8	F	Chronic abdominal pain	Positive for Giardia	ND	23.4	23.9	>40
8	5	M	FTT and iron deficiency anemia	Positive for *Giardia*	ND	21.4	23.1	>40
			**Mean±SD**		**33.4±1.1**	**23.7±4**	**26.3±3.4**	

Cutoff for positive Ct for miR5< 33.5. Abbreviations: CVID, common variable immunodeficiency; ITP, immune thrombocytopenic purpura; FTT, failure to thrive.

**Fig 1:**
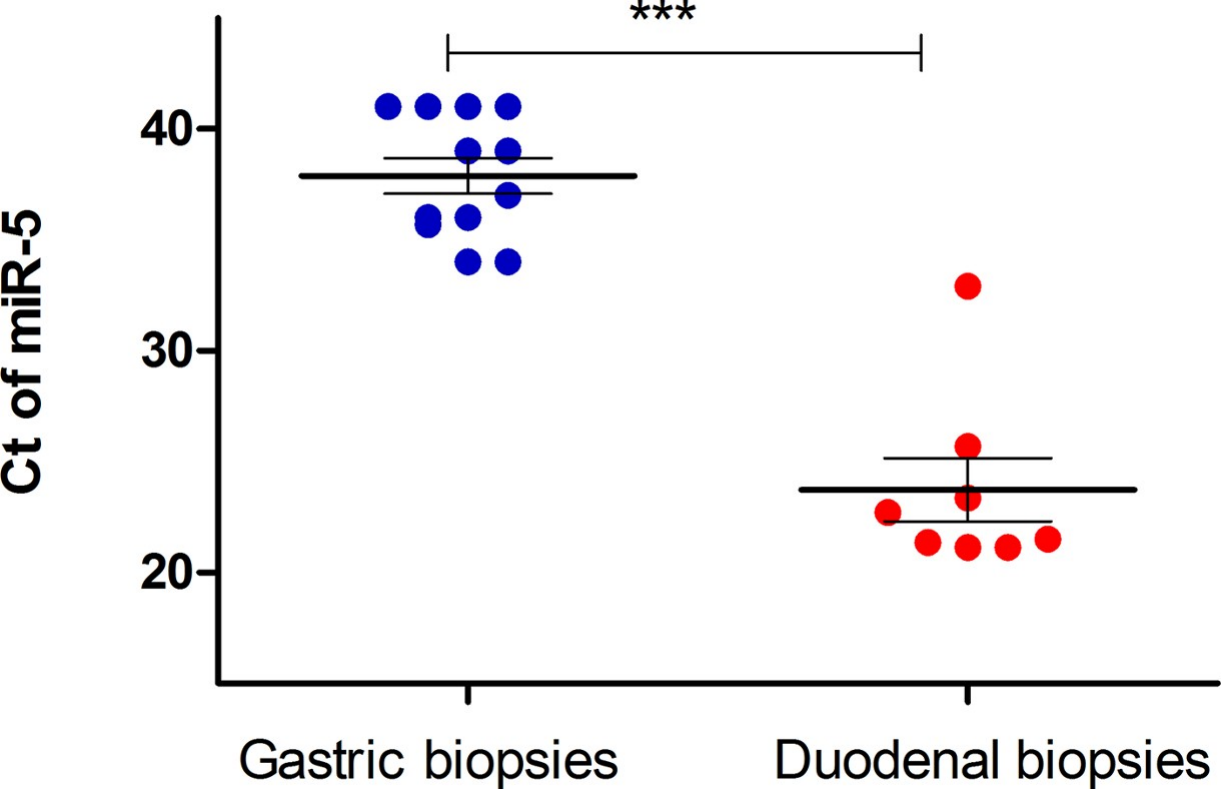
Detection of *Giardia* miR5 in human duodenum biopsies. RNA was extracted from 8 duodenum biopsies of *Giardia* positive patients or as control group, from 4 gastric biopsies of *Giardia* positive patients and from 6 random gastric biopsies. The RNA was subjected to qRT-PCR (see Methods) with specific primers to miR5. Each dot represents patient’s or control individual’s samples. Mean ± SEM are depicted by horizontal lines. ***, P< 0.0001, calculated by unpaired t-test.

As a negative control, we used gastric biopsies which are not expected to harbor *Giardia* parasites. Firstly, we used 4 available gastric biopsies from the 8 patients mentioned above with positive duodenal histology. Indeed, no miR5 was detected in gastric biopsies of these patients (Table 1). Six additional gastric biopsies from randomly selected patients who underwent gastroscopy for various reasons showed Ct values above 34. Altogether in all 10 control biopsies miR5 Ct values were above 34 with mean 35.9 (±1.6) compared to 23.7 (±4.0) in the positive cases (p<0.0001) (Fig 1).

**Fig 2:**
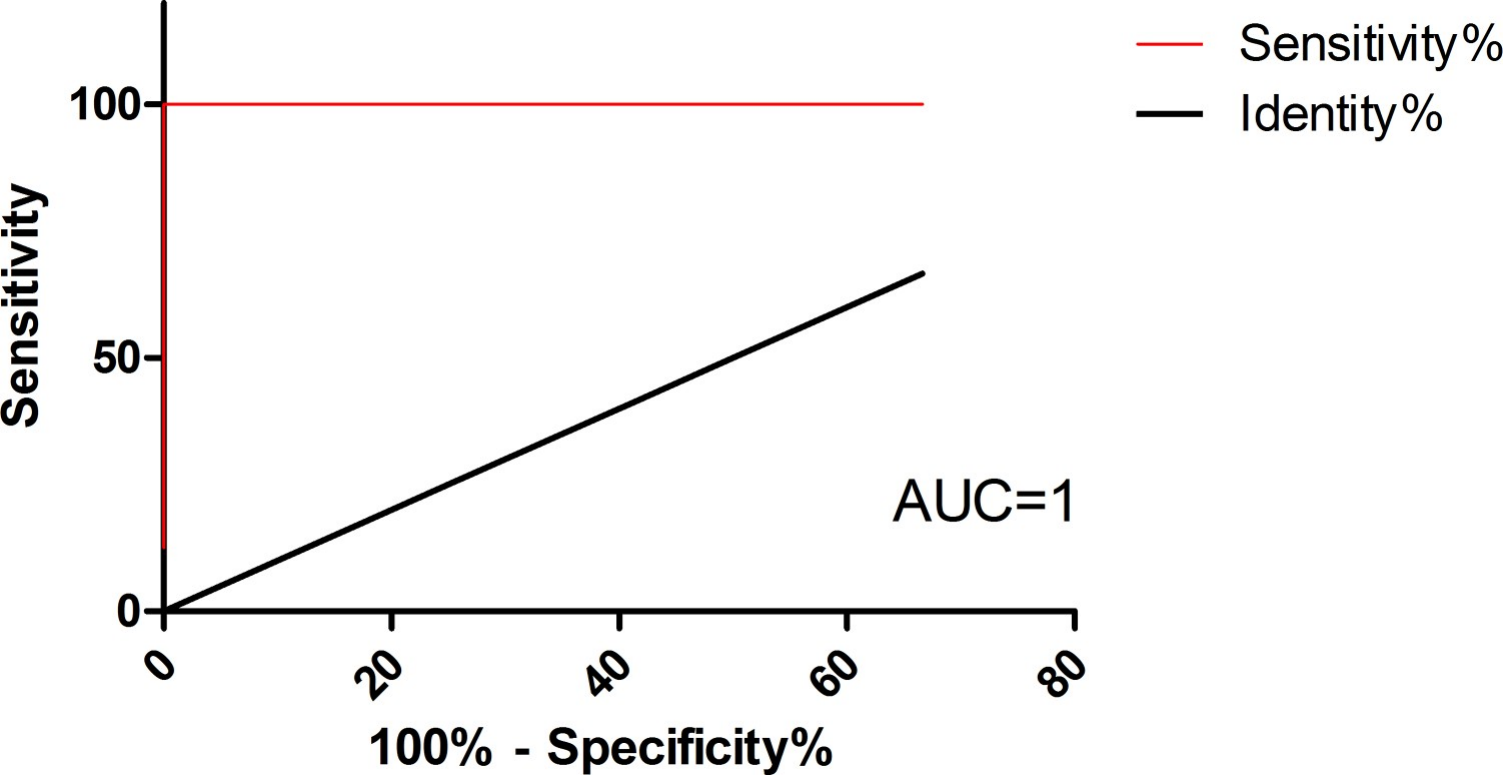
ROC curve analysis. Area under ROC curves (AUC) analysis were performed with Ct values of the *Giardia* miR-5 as predictors for disease category (infected = duodenum positive /control = gastric) as response variable (red lines). P value = 0.0002. ROC curve was fitted using GraphPad Prism version 5.00 for Windows. The diagonal black line reflects the performance of a diagnostic test that is no better than chance level.

Applying receiver operating characteristic (ROC) curve analysis, we observed highly sensitive and specific results. The calculated area under the curve (AUC) for miR5 was 1, representing a perfect test; [41]. The optimal cutoff is determined as the cutoff with the highest likelihood ratio [likelihood ratio is defined as %sensitivity / (100-%specificity)]. With miR5, Ct <33.5 yielded 100% sensitivity and specificity and therefore likelihood ration of ∞ (95% confidence intervals–sensitivity 63.1% to 100.0%, specificity 59.0% to 100.0%) (Fig 2) and S2 Table.

**Fig 3:**
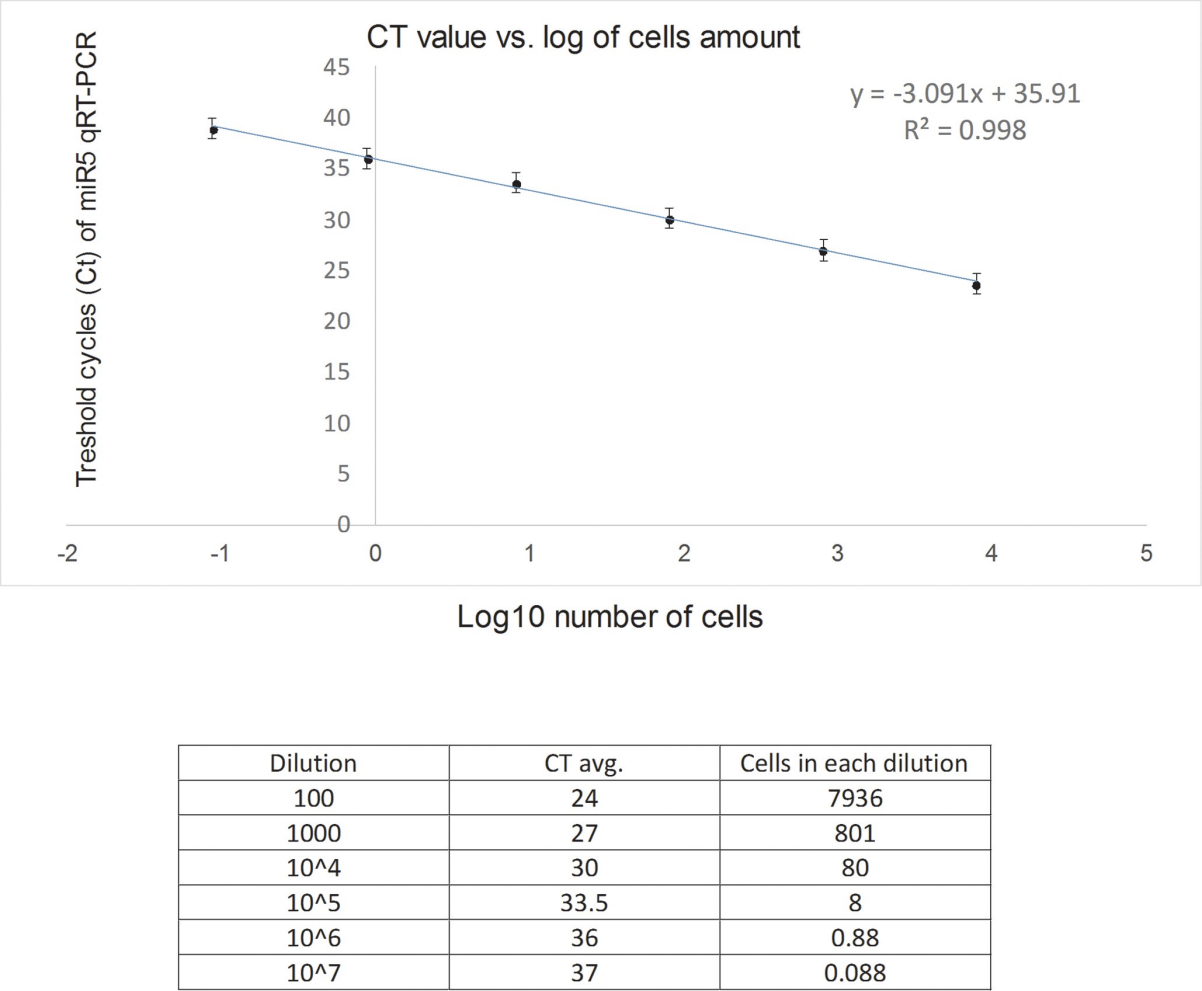
In-vitro calculation of miR5 qRT-PCR Ct value according to number of parasites. WB strain *Giardia* were grown in culture. Number of parasites were counted, and diluted as shown in the table. From each dilution, RNA was extracted and subject to qRT-PCR. The graph represents an average of three repeat, and as the log of parasites concentration versus obtained Ct.

To determine the minimal number of *Giardia* parasites which can be detected by miR5 qRT-PCR we used an in-vitro model, extracted RNA from parasites, and correlated the number of parasite cells with Ct reading (Fig 3). As can be seen, at 10^4^ dilution we counted ~8 parasites with Ct value of 33.1, slightly below the 33.5 Ct cutoff defined above. Thus, the lower limit of detection of our miR5 assay may be 8 parasite cells.

Interestingly, 3 patients with suspected travel-acquired giardiasis without evidence for *Giardia* by histological examination of duodenum tissue nor by stool microscopy were analyzed for miR5 in the duodenum. In one of these patients the biopsy which was taken before treatment yielded a miR5 Ct value of 31.0, positive for *Giardia* infection, while the biopsies of the other two patients, which were taken after anti-giardia treatment, yielded borderline Ct levels, 33.8 and 34 (Table 2). All three patients’ symptoms responded to anti *Giardia* treatment.

**Table 2:** miR5 detection in duodenal biopsies of patients with travel related persistent abdominal symptoms and no *Giardia* detection by histological examination.

	Sex	Age	Medical history	Biopsy timing	Improvement after treatment	Biopsy pathology	Ct miR5 duodenum	Ct DNA
1	M	34	Chronic diarrhea	Pre-treatment	Yes	Normal examination	31.0	>40
2	F	59	Chronic flatulence	Post-treatment	Yes	Normal examination	33.8	>40
3	M	68	Chronic diarrhea	Post-treatment	Yes	Normal examination	34.0	>40

Cutoff Ct for positive miR5: 33.5

### Detection of *Giardia* miRNAs in stool samples

19 stool samples were collected and included in the study. 9 stool samples were taken from patients who suffered from diarrhea with proven giardiasis. In the control group, 10 stool samples were taken from healthy infants; 6 newborns and 4 toddlers, aged 1 to 1.5 years, who have just started eating solid foods and have not yet entered nursery school. Patient samples were tested for the presence of *Giardia* by three different methods: microscopic examination (O&P), ELISA and DNA PCR. Samples were defined as positive if at least two of the above mentioned diagnostic methods were positive.

**Fig 4:**
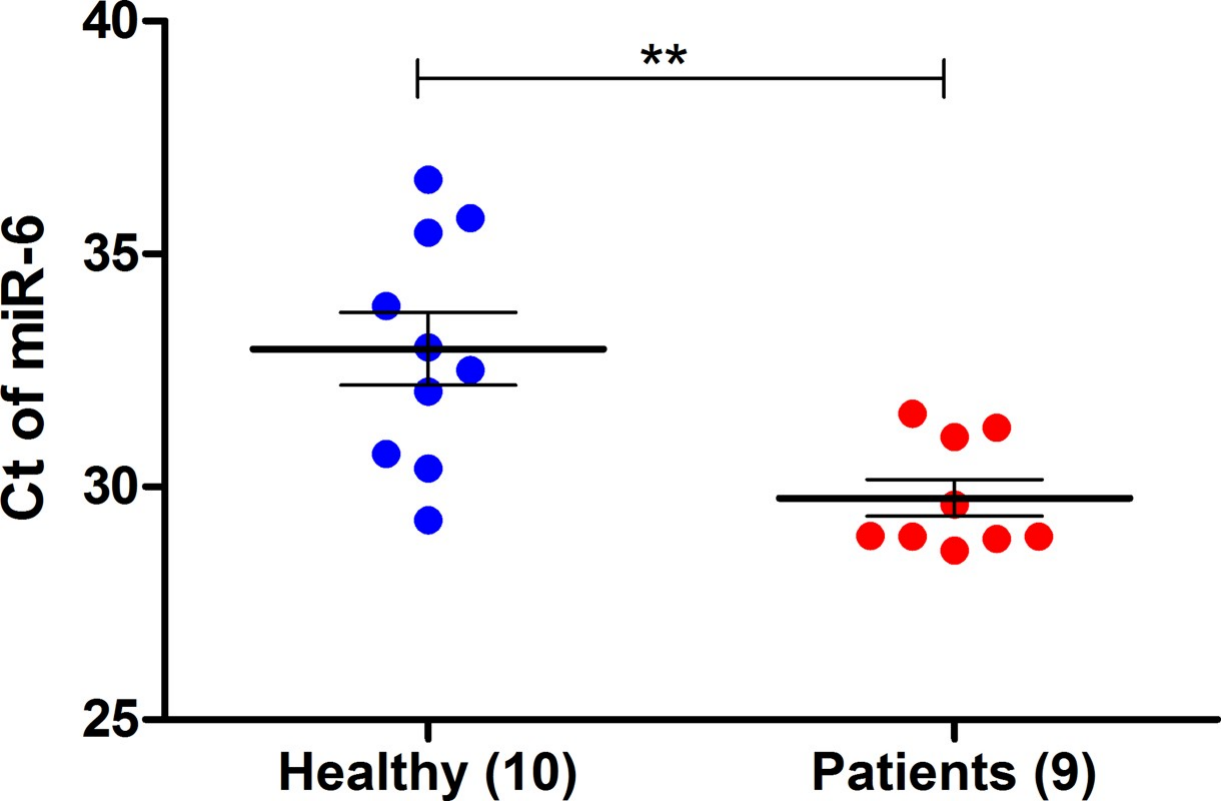
Detection of *Giardia* miR6 in human feces. RNA was extracted from the feces and was subjected to one step RT-PCR (see Methods) with specific primers to miR6. Each dot represents patient’s or healthy individual’s samples. Mean ± SEM are denoted by horizontal lines. **, P value, 0.0025, was calculated by unpaired t-test.

**Fig 5:**
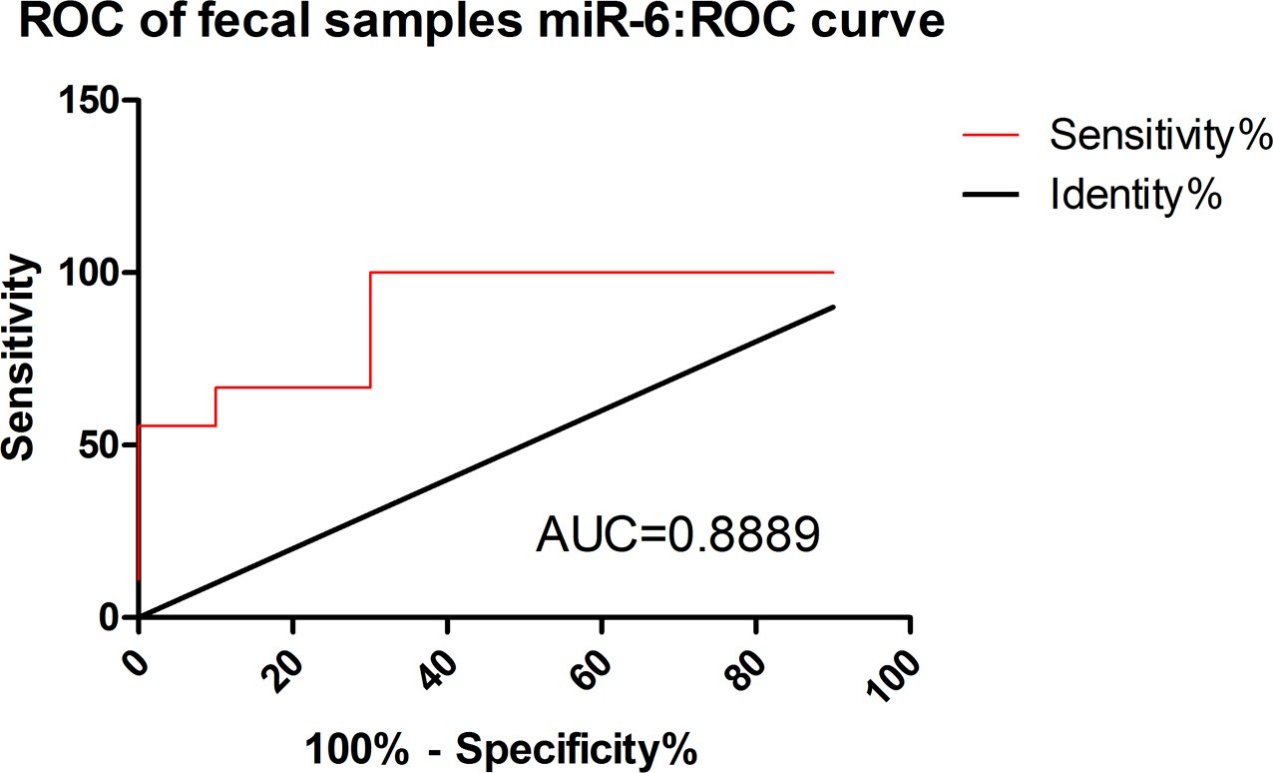
ROC curve analysis. Area under ROC curves (AUC) analysis were performed with Ct values of the *Giardia* miR6 as predictors for disease category (infected = stool positive /control = stool negative) as response variable (red lines). P value = 0.0043. ROC curve was fitted using GraphPad Prism version 5.00 for Windows. The diagonal black line reflects the performance of a diagnostic test that is no better than chance level.

Measurement of miR5 and miR6, in patients’ fecal samples was performed. RNA from stool was extracted and one-step real time RT-PCR analysis was done [39], an assay we have previously used, successfully identifying very small amount of miRNA [38]. As can be seen in Fig 4, a significant difference was found for miR6 between positive patients compared to healthy individuals (p = 0.0025). We applied ROC curve analysis to evaluate the accuracy of our test. As shown in Fig 5, the accuracy of analyzing the presence of *Giardia* miRNAs extracted from stool samples of infected patients was moderate; with AUC for miR6 is 0.88. The likelihood ratios for miR6, based on, sensitivity of 66.6% and specificity of 90% was Ct of 30.0 (3S Table).

Unlike miR6, when miR5 was measured, its levels did not differ between the infected and healthy samples (data not shown), implying non-specific (false-positive) amplification.

## Discussion

Giardiasis is a significant disease and is underdiagnosed. In this study, our aim was to find a new method that will contribute to the current available diagnostic methods of *Giardia* infection. In order to do this, we chose to focus on miRNA molecules of *Giardia* due to their stability, which makes them good candidates to survive complex extraction methods. The fact that miRNA molecules do not exist in the bacteria that constitute most of the gut flora makes them promising in terms of specificity. The use of miRNA for diagnosis of parasitic infections is in its prime [17] and to our knowledge has never been used in the diagnosis of *Giardia* infection.

We are aware of the discussion of whether *Giardia* has *bona fide* miRNA. However, this discussion is less relevant to our work. The precise biochemical definition and function of these small RNAs is of lesser importance with regards to their potential as biomarkers. Nevertheless, since the small-RNA we analyzed were already named as miRNA by others, we maintained this nomenclature (e.g., miR5 and miR6).

We performed deep sequencing analysis of small RNA extracted from 5 different *Giardia* isolates. Based on their abundance in our data as well as in previous reports by Saraiya et al and Liao et al [17, 18, 36], we decided to focus on two molecules- miR5 and miR6, which by BLAST analysis appear to be non-cognate to the human genome and transcriptome.

Since *Giardia* resides in the upper gut, we decided to look for the presence of our two chosen molecules in duodenal biopsy specimens of 8 patients who were found to have *Giardia* parasites on histological examination (Table 1). These 8 patients were referred to gastroscopy due to prolonged gastrointestinal system complaints. Interestingly, none of these patients were suspected of having giardiasis prior to the gastroscopy.

All eight specimens determined as positive by pathology were verified as positive by using a DNA PCR test for *Giardia*. Applying qRT-PCR for *Giardia* miR5 was also positive in all samples. Interestingly, miR6 was less efficient, moreover in 4 out of the 8 samples we did not detect it at all (Table 1). This might be because our primers were designed to miR6 of assemblages A and as shown in S1 Table there are 4 nucleotides different between miR6 of assemblage A and assemblage B. Interestingly, although we extracted DNA and RNA from the same amount of FFPE slides, we identified miR5 at lower Ct cycles, that might indicate robustness of miR5 compared to DNA testing. Using gastric biopsies as negative controls, we indeed found that no miR5 was detected even in patients who had *Giardia* in their duodenal specimen. In addition, all of the control gastric biopsies from non-*Giardia* patients were negative for miR5.

We had three additional cases with clinically suspected giardiasis but negative *Giardia* diagnosis by conventional methods and negative duodenal biopsies (Table 2). One of these patients was miR5 positive with Ct of 31 (our calculated cutoff is 33.5). The two other patients were biopsied after empiric treatment and according to the Ct values of 33.8 and 34.0 could be categorized as borderline or negative after treatment, for *Giardia* miRNA. Moreover, although we do not have any additional supporting evidence that these three patients had giardiasis, all three responded to anti-*Giardia* treatment. As can be seen in Fig 3A and 3B we generated a calibration-curve of the ratio of Ct obtained in qRT-PCR of *Giardia* miRNA to the number of parasite cells counted. Based on this curve, Ct of 33.0 indicates 8 parasite cells. Hence, as our calculated cutoff for positive infection is 33.5, it would suggest that qRT-PCR can detect as few as 8 parasites, while based on the mean Ct level (23.7) of histology-positive biopsy there is a need for 100–1000 times more parasites to be detected by histopathology (Fig 3), illustrating the advantage of qRT-PCR miRNA detection in duodenal biopsies. Additionally, these positive *Giardia* miRNA cases were negative for *Giardia* DNA which also strengthens the superiority of miRNA on DNA in duodenal biopsies.

As mentioned, none of the research group patients were suspected to have giardiasis prior to the diagnostic gastroscopy (Table 1). This suggests that there is indeed under-diagnosis of *Giardia* infections in Israel and probably in the industrialized world in general. In addition, our assumption is that giardiasis is an underdiagnosed illness due to the low sensitivity of current diagnostic tools. This assumption was made from observing patients with chronic gastrointestinal complaints, some being returning travelers from the tropics. These patients had negative stool tests for parasites and were left with a presumable diagnosis of irritable bowel syndrome, however empiric anti-protozoal treatment led to significant improvement in 70% [8].

Early studies showed that a single duodenal biopsy might be insufficient for diagnosis of giardiasis. In one of the studies it was suggested that two samples might be sufficient to diagnose all of the cases probably due to the non-homogenous distribution of the parasites in the duodenum [42].

Comparing duodenal aspirate samples to biopsy samples in giardiasis patients had mixed results in different studies. Some studies have shown superiority of duodenal aspirate samples over duodenal mucosal biopsy [43]. Others showed that duodenal mucosal biopsies were more sensitive compared to duodenal aspirate samples [44].

In both studies the test was histological examination and both suggested that duodenal aspirate samples or duodenal mucosal biopsies were more accurate, compared to stool examination.

Fouad and colleagues subjected stool samples and duodenal aspirates from 120 patients with dyspepsia to PCR analysis for *Giardia* DNA and searched concurrent duodenal biopsy samples for organisms. *Giardia* was detected by PCR in duodenal aspirates in 23 cases, but organisms were present in biopsy samples from only 2 of these patients (sensitivity 9%). While a study done in a high prevalence setting showed that as many as 44% (96/220) of patients who underwent gastroduodenoscopy due to dyspeptic symptoms had *Giardia* on duodenal biopsy [45]. In that study it seemed that duodenal diagnosis is much more sensitive than stool microscopy since in only a minority of them (5/85, 6%) *Giardia* parasites were found in stool examination.

In the last part of our study we aimed to detect miR5 and miR6 molecules in stool samples of patients with proven giardiasis (who were found to be positive by at least two alternative stool tests). As a control group we chose two populations. The first were newborns, who presumably were not yet to be exposed to the parasite. The second control group was toddlers without siblings, who had not yet started attending nursery school but were no longer breastfed and started eating solid foods. These groups had a relatively small chance of exposure to *Giardia*. The results of our study showed moderate accuracy with 90% specificity and only 66.7% sensitivity in diagnosing *Giardia* infection using stool miR6. The results of miR5 quantification in stool were even less accurate.

It seems that identifying *Giardia* miRNA molecules in duodenal specimens shows more potential than in stool sampling. Extraction of miRNA from paraffin is straightforward and less prone to the background noise that exists in the stool due to heavy bacterial burden. *Giardia* parasites reside in the duodenum where they reach high cell counts, whereas in the stool their numbers are relatively small and excretion is periodic. The fact that miR5 was identified in duodenal specimens but not stool samples, and vice versa miR6, suggests differences in expression of miRNA molecules between *Giardia* trophozoites (exist in duodenum) and *Giardia* cysts (exist in stool). miRNA libraries that we have built were derived from sequencing *Giardia* cultures in its trophozoite form. That is also the main form of the parasite residing in the duodenum, though in a different environment.

The main limitation of our study is the relatively small sample numbers in both the experimental and the control groups. Another limitation is that we have assessed only two miRNA molecules.

In conclusion, miR5 testing for *Giardia* infection in duodenal biopsies may be a breakthrough method for diagnosis of giardiasis that has a potential of being more sensitive than current methods. Obviously, we do not suggest duodenal biopsies for each patient suspected of giardiasis. However, we think that qRT-PCR for *Giardia* miRNAs should be one of the tests in patients undergo endoscopy investigation for undiagnosed persistent abdominal symptoms.

We want to emphasize that fact the duodenal biopsy in our samples were taken from patients that suffer from persistent abdominal symptoms and were not suspected of being infected with *Giardia*. Only the pathologist, unexpectedly, detect *Giardia* in there duodenal biopsy. Therefore, we think that qRT-PCR for *Giardia* miRNAs should be one of the tests such patients undergo. Therefore, it would be important to further investigate the contribution of *Giardia* miRNA testing in duodenal biopsies and duodenal aspirates from patients with persistent abdominal symptoms.

## Supporting information

S1 Appendix Results and insights from our small RNA transcriptome analysis of 5 isolates of *Giardia* trophozoites (reagents provided by the BEI Resources). Included in this appendix are:
(DOCX)

S1 Folder The miRDeep2 algorithm (when allowed up to 50% of the mapped reads to be inconsistent with Dicer processing) yielded 58 novel suggestions for miRNA precursors, which are shown in this folder. The result_16_06_2018_t_13_52_59.html file is an index, through which pdf plot can be accessed.
(ZIP)

S1 Text The output of miRDeep2 analysis searching for known and novel miRNA in the small RNA libraries of 5 *Giardia* trophozoite isolates (run with the “-t” option so that disqualified precursors are included in the output). The output shows collapsed read pile-ups for every proposed miRNA precursor and lists reasons for disqualification. The sequences are sorted by abundance and their source library is specified. For example, the sequence “Gi3_8900428_x63185” was found 63185 times in library Gi3
(PDF)

S1 Table Previously reported putative *Giardia* miRNA (adapted from Liao et al, Proc Natl Acad Sci USA 2014; S1 Table). We specifically sought these sequences in the current analysis, by including them as presumed known mature miRNA in the miRDeep2 analysis.
(DOCX)

S2 Table Data of Giardia miR5 in human duodenal biopsies, real time Ct results and ROC curve data. Calculation of the optimal cutoff as determined as the cutoff with the highest likelihood ratio [defined as %sensitivity / (100-%specificity)].
(DOCX)

S3 Table Data of Giardia miR6 in human stool samples real time Ct results and ROC curve data. Calculation of the optimal cutoff as determined as the cutoff with the highest likelihood ratio [defined as %sensitivity / (100-%specificity)].
(DOCX)

S1 Fig The top 5 novel candidates (S1 Folder), in terms of consistency with canonical miRNA processing, are also depicted in this figure. All are lowly expressed, and thus unlikely to exert significant regulation.
(PDF)

S2 Fig The predicted structure and partial pile-up of collapsed reads mapping to the putative miR5 precursor. The sequences are sorted by abundance and their source library is specified (as in S1 Text).
(PDF)

S3 Fig The predicted structure and partial pile-up of collapsed reads mapping to the putative miR6 precursor. The sequences are sorted by abundance and their source library is specified (as in S1 Text).
(PDF)
