# Supplementary material for: Giardia lamblia miRNAs as a new diagnostic tool for human giardiasis
Source: PLoS Negl Trop Dis. 2019 Jun 17;13(6):e0007398. doi: 10.1371/journal.pntd.0007398 (PMC6597124; doi:10.1371/journal.pntd.0007398)
Supplement: S1 Appendix — Included in this appendix are: (DOCX) [file pntd.0007398.s001.docx]

**Supplementary Results**

The numbers of reads obtained from small RNA sequencing of libraries prepared from 5 Giardia strains are depicted in **Table A**.

| **Table A:** Sequencing reads statistics from Giardia trophozoite small RNA profiling | | | | | |
| --- | --- | --- | --- | --- | --- |
| Library | Isolate^a^ | Total reads | Reads ≥18 nt | Genome-mapped reads^b^ | Length (nt) of mapped reads, mean ± sd, mode |
| Gi1 | Egypt-4 | 4903304 | 4390722 | 27.6% | 21.4 ± 2.7, 21 |
| Gi2 | Mario | 13055306 | 11407891 | 41.6% | 21.6 ± 2.0, 18 |
| Gi3 | SUG | 31968094 | 28988905 | 35.4% | 21.9 ± 2.8, 18 |
| Gi4 | WB cl.6 | 738628 | 614388 | 13.1% | 21.9 ± 2.7, 18 |
| Gi5 | G2M | 9968845 | 9138545 | 39.8% | 22.3 ± 2.7, 22 |
| a, BEI Resources Catalog No. NR-9231, NR-9232, NR-9233, NR-9706 and NR-9240, respectively.  b, percentage of ≥18 nt reads mapping to the GiardiaDB-37_GintestinalisAssemblageAWB genome downloaded from [GiardiaDB](http://www.giardiadb.org/). | | | | | |

Table B. Previously identified miRNAs from Giardia lamblia (modified from Liao et al, Both endo-siRNAs and tRNA-derived small RNAs are involved in the differentiation of primitive eukaryote Giardia lamblia. Proc Natl Acad Sci U S A. 2014 Sep 30;111(39):14159-64)

| **miRNA** | **Sequence** | **Length, nt** | **Overlap with known ncRNA** | **Reference** |
| --- | --- | --- | --- | --- |
| **glm-001** | GCTGGGATCGTATAAAGG | 18 | tRNA-HisGTG | 1 |
| **Gl-mir-1** | CTCATCTATACACAGTGTCGC | 21 |  | 2 |
| **Gl-mir-2** | GCAGGAGTTGCAGCGCAGTGC | 21 |  | 2 |
| **Gl-mir-3** | CTCTACCTCCTGACTGCATTG | 21 |  | 2 |
| **Gl-mir-4** | ATCGCACACCATAGCCTTGTG | 21 |  | 2 |
| **Gl-mir-5** | AGCAGAGAAAGTGTCTTTCCG | 21 |  | 2 |
| **Gl-mir-6** | AGGTCGGACTCGCGCGCCTCC | 21 |  | 2 |
| **Gl-mir-7** | GGACCAGGCCGTCGTCGAAGT | 21 |  | 2 |
| **Gl-mir-8** | TAGCACGGCGATCTGTGTCTG | 21 |  | 2 |
| **Gl-mir-9** | AGGACGTAGATGATGACATCT | 21 |  | 2 |
| **Gl-mir-10** | GAGGTCTTCTTCCAAGGCAAT | 21 |  | 2 |
| **Gl-mir-11** | CTATAAGAAGAGATATCTTGT | 21 |  | 2 |
| **Gl-mir-12** | TAGCTTATGGTAGTTGCACCT | 21 |  | 2 |
| **Gl-mir-13** | CGTGCGCACCCCCCTTGGTAT | 21 |  | 2 |
| **Gl-mir-14** | GACGAAGATCGCGCCCCACGA | 21 |  | 2 |
| **Gl-mir-15** | CATCCATGTAGACATCTTGCG | 21 |  | 2 |
| **Gl-mir-16** | TGGGAGCGTGTGCACAGGCAG | 21 |  | 2 |
| **Gl-mir-17** | AGTGCGAGGACCAACGCGGTT | 21 |  | 2 |
| **Gl-mir-18** | CCCCTCTGTGGACTCGTGAGT | 21 |  | 2 |
| **Gl-mir-19** | GTCCGATTACCAACCCTTTGA | 21 |  | 2 |
| **Gl-mir-20** | TGAGCAGATCTCAGAGGTATA | 21 |  | 2 |
| **Gl-mir-21** | TGAGTACTGGTTTTGCACCTT | 21 |  | 2 |
| **Gl-mir-22** | TGTAACAGTCATACATGTACA | 21 |  | 2 |
| **Gl-mir-23** | CGTGCATGGAATAGTGTGTAT | 21 |  | 2 |
| **Gl-mir-24** | TGTCTTTTGAAGAGCTTTGTG | 21 |  | 2 |
| **Gl-mir-25** | AGATCTGGATCGCCTTCAGCT | 21 |  | 2 |
| **Gl-mir-26** | CGTGCGGGCCCGGGGGCAGGG | 21 |  | 2 |
| **Gl-mir-27** | TTGGCTACTGCAGTCAGCTCT | 21 |  | 2 |
| **Gl-mir-28** | TGAGATCATAGAGCCGCTTTG | 21 |  | 2 |
| **Gl-mir-29** | CCCTTCAAATCCCTGATTATG | 21 |  | 2 |
| **Gl-mir-30** | CAGCGTGTGTGTCCTGCCGAG | 21 |  | 2 |
| **Gl-mir-31** | CGAGGAGACGTTGGAGAATGT | 21 |  | 2 |
| **Gl-mir-32** | GTAGCAAAGATCCTTCTTGCT | 21 |  | 2 |
| **Gl-mir-33** | ATCGAAAGCACTCTGGAGGCT | 21 |  | 2 |
| **Gl-mir-34** | CGCCGTCACGGAGGTCCGCCG | 21 |  | 2 |
| **Gl-mir-35** | AGTGTATCTAAAACCTGGCAC | 21 |  | 2 |
| **Gl-mir-36** | TGCCAGGTTTTAGATACACTA | 21 |  | 2 |
| **Gl-mir-37** | ATCCATGTTGTAGCGCTGGTT | 21 |  | 2 |
| **Gl-mir-38** | TCCCTGAAGGTACTTGGCTAA | 21 |  | 2 |
| **Gl-mir-39** | CCCAGAGTGTCGTCTCTCAGC | 21 |  | 2 |
| **Gl-mir-40** | TCCGGTAGGTATTTTATAAAT | 21 |  | 2 |
| **Gl-mir-41** | CCCGTTTCTTGGGGTGCCTCT | 21 |  | 2 |
| **Gl-mir-42** | CCAGTCATACATGTACACAGT | 21 |  | 2 |
| **Gl-mir-43** | GATGGGTGGTAATGAGGCCTG | 21 |  | 2 |
| **Gl-mir-44** | AAGCTGCTTGTTTGTGAAGTC | 21 |  | 2 |
| **Gl-mir-45** | TGGATTCAGGCCGCGCGTGTT | 21 |  | 2 |
| **Gl-mir-46** | GAGCAGTAGCTGCCTTTGATA | 21 |  | 2 |
| **Gl-mir-47** | TTCGAAATAGGAGCCAAGAGT | 21 |  | 2 |
| **Gl-mir-48** | GGCGTCCATGCACCTGACGCT | 21 |  | 2 |
| **Gl-mir-49** | AGGTGCATGGACGCCGGGCGC | 21 |  | 2 |
| **Gl-mir-50** | CACGAGAGTGCGCGACCGGGG | 21 |  | 2 |
| **Gim1** | ATCAACGTGACTGATGCTGGCTCT | 24 |  | 3 |
| **Gim2** | ATCTCGCACATATACCGGCCTCCT | 24 |  | 3 |
| **Gim3** | GTGCAGAGGCATGGAGCACGGGAA | 24 | U0121 | 3 |
| **Gim4** | GTGGTCTGCATCTGGACCTTCACT | 24 |  | 3 |
| **Gim5** | GGCCGTGTGGTTAGGTGGTTGTTG | 24 |  | 3 |
| **Gim6** | GTGGTGAGTAGAAGTCAGATTATAA | 25 |  | 3 |
| **Gim7** | GCGGTCGCTTGGGTCCCAGCGGGTTC | 26 |  | 3 |
| **Gim8** | GGTCGGTTAGCTCAGTCGGTAGAGCG | 26 | tRNA-IleGAT | 3 |
| **Gim9** | GTAGGATGCCCCAGAGACTGCCGAG | 25 |  | 3 |
| **Gim10** | AAACTCTCCGCACAGGGGCGCGCCTG | 26 |  | 3 |
| **miR2** | CAGCCTAATCACCGCCCCTATAGTCC | 26 | GlsR17 | 4 |
| **miR3** | GCAGACAACGCATCACCGCTCTGACC | 26 | GlsR16 | 4 |
| **miR4** | TCTGCACCAAGGAGCTGATCCAGACC | 26 | SGR | 5 |
| **miR5** | GATGCTTCCTTGGATGTCCGAGCCTT | 26 | GlsR2 | 6 |
| **miR6** | GACGCGTGACGAAGTTTGTCGTATTCTG | 28 | GlsR1 | 7 |
| **miR10** | TGAGGAAGAAACCGCCTTTCGTCTGACC | 28 | GlsR8 | 7 |
| **miR11** | ATGTCTGCCGTGTGCGCGCATATCCT | 26 |  | 8 |
| **miR12** | TGTGTTTCTAGTAAACAGTCGGGATC | 26 |  | 8 |
| **miR13** | CCTGATGGAGAAACCTTCCTGGACGG | 26 |  | 8 |
| **miR14** | AGGCGCAGGCCCGCCGCGAGGG | 22 |  | 8 |
| **miR15** | CCCGTGAGGTCTGCCAGGAGGAGGGT | 26 |  | 8 |
| **miR18** | ACCACAAACATCGACACGGAGAGCTGC | 27 |  | 8 |
| **miR19** | GTTCTTTTCGCGTGAGCTGGTCGACGCC | 28 |  | 8 |
| **miR20** | GACGTGCTAGGTCAGGCAGACGGGCTCC | 28 |  | 8 |
| **miR21** | ATGGGCTGGGTTAAGGTTCAGAAGACGC | 28 |  | 8 |
| **miR22** | AAATTGACAAGAAGATCGCTGAGGCA | 26 |  | 8 |
| **miR23** | CGTTTGCCGTCTTACAATGCTCTGACC | 27 |  | 8 |
| **miR24** | TTCGCCTGGATCGCATAGGCAA | 22 |  | 8 |
| **miR25** | TGGGCCCAAAAAAACGAAAGCGTCCC | 26 | SGR | 8 |
| **miR26** | TCACGGTCTCGCAGATCATGCGCAA | 25 |  | 8 |
| **miR27** | TAGTGTAGGGATGTCCAAAAAACCAG | 26 |  | 8 |
| **miR28** | TACTACGACGAGGGCAAGAACACGAA | 26 | SGR | 8 |
| **miR29** | TACAACGGCGTGCCCCAGACCTTCAA | 26 | SGR | 8 |
| **miR30** | GACGGGGCGGGAGGATTTGCCA | 22 |  | 8 |
| **miR31** | TACCACCGCGACGACGACCCCCACA | 25 | SGR | 8 |
| **miR32** | TGCTTCCACGGGGCGAACAATG | 22 |  | 8 |
| **miR33** | TACGAGATGGAGAGCAGACGGACC | 24 | SGR | 8 |
| **miR34** | TCTACACCCTCAAGGGCCACCCGAA | 25 |  | 8 |
| **miR35** | CATCAACCGCCAGACGGAGAAGGGCC | 26 | SGR | 8 |
| **miR36** | AGCCCCTCCGGCGAGATGTTCG | 22 |  | 8 |
| **miR37** | CGGTCGAGGCGTTGAGGAAGCA | 22 |  | 8 |
| **miR38** | CCCGCCCAGTAAACAAGCCCTGCA | 24 |  | 8 |
| **miR39** | CCTGGGATAATGCGCTTCTTTGAGCCGCG | 29 |  | 8 |
| **miR40** | AGCCTAATCACCGCCCCTATAGTCC | 25 |  | 8 |
| **miR41** | GTTCGAGGGCGCGGAGCACATTCCAAA | 27 |  | 8 |
| **miR42** | GACCTCACCAAGATTTGCAAGGACGCTG | 28 |  | 8 |
| **miR43** | CTCATTGTGAAGGAGGCAGACCCAGG | 26 |  | 8 |
| **miR44** | TCTGCGACGATCGGGTAGACGATGC | 25 | SGR | 8 |
| **miR45** | TATTCCATTCACTCAGTCAGACCCAG | 26 |  | 8 |
| **miR46** | TGGTCCCAGTAGCAATAGACGTAG | 24 |  | 8 |
| **miR47** | CGACGTCATCCACGCGAAGATCCGCTC | 27 | SGR | 8 |
| **miR48** | TGAAGGGCCTCGACACGAATCCGAAGA | 27 | SGR | 8 |
| **miR49** | TCAAGCCGTATGGCACCCAGAGGACC | 26 | SGR | 8 |
| **miR50** | TGAACTGGGCGAGGTTCCAGCGGACG | 26 | SGR | 8 |
| **miR51** | AGTAAACATCGAATTCACGTCAGCGTT | 27 | SGR | 8 |
| **miR52** | CGAAGACGACCTTCTGCATCGGCTCCC | 27 | SGR | 8 |
| **miR53** | TGCGAGCTTGCGGACGTTCTCCTGCG | 26 | SGR | 8 |
| **miR54** | GCTGGAGAGTGCCCTTCAATCGCTGG | 26 |  | 8 |
| **miR55** | AACTATGATGAGGTTAGCGATCCCAAGC | 28 |  | 8 |
| **miR56** | GATGACAAGGACAAGTATGAGACGTTC | 27 |  | 8 |
| **miR57** | AACGTTGCTGATCCAGAGGTTCCTCTC | 27 |  | 8 |
| **miR58** | TTAGACTGCTGAGACAGTGTTATATGATT | 29 |  | 8 |
| **miR59** | CTATGTTGAGAACCACCCAAACAA | 24 |  | 8 |
| **miR60** | GTCTTCCGAACACACCTGCGATAAAC | 26 |  | 8 |
| **miR61** | GGCGGAATGTTCGGCGGCGACTCAT | 25 |  | 8 |
| **miR62** | GGTGGAATGTTCGGCGGCGACTCGT | 25 |  | 8 |
| **miR63** | GAGAAGATCCGCGAGGGCCAGAAGG | 25 |  | 8 |
| **miR64** | GCCAAGGAGGACGAGAAGATCCGCGAGG | 28 |  | 8 |
| **miR65** | CTTCAAGGGCGGGAACGACGAGAGGT | 26 |  | 8 |
| **miR66** | CCCGGGTAGGCACGGTCAAAGAGT | 24 |  | 8 |
| **miR67** | AGAAGATAGAGAAAGAGCTTTCGGAC | 26 |  | 8 |
| **miR68** | TGCTAGTCACCGTCCCTCTGTGGCGTC | 27 |  | 8 |
| **miR69** | TGAGGTCCATGAAGGCCGTCGCCA | 24 |  | 8 |
| **miR70** | TGAGGGAGCTGAGGTCCATGAAGGC | 25 |  | 8 |
| **miR71** | TGGCAGGCCGTGCAGGACGAGGCGT | 25 |  | 8 |
| **miR72** | CGCAAAGGAGTGCAAGAAGTGTGCAGAA | 28 |  | 8 |
| **miR73** | CGTTAAAGAGGCTCAGGACTGGCTCC | 26 |  | 8 |
| **miR74** | GCCAATAAATGCGCGTGTGATCGAAGT | 27 |  | 8 |
| **miR75** | CTCACGGAAGAAGAGGCGCTGCTCAGG | 27 |  | 8 |
| **miR76** | CGAGAACGGAAAGCTCTGGATGCTTCA | 27 |  | 8 |
| **miR77** | GTCAACAAGAGCGGCCTCAGCACAGGT | 27 |  | 8 |
| **miR78** | GGGTGATTAGCAGTCATACAGTCC | 24 |  | 8 |
| **miR79** | GGCTAGAGCGCGACTGGTTGAGTTCCC | 27 |  | 8 |
| **miR80** | CCTTGTCCCGGCTGGCGCCGTCCACCTT | 28 |  | 8 |
| **miR81** | GTCCACCTCTGGTTCGGCACACATT | 25 |  | 8 |
| **miR82** | GAAGAGTGTCAAGGAGAAGACGGAGAT | 27 |  | 8 |
| **miR83** | GGCAGATGATGACTTTGCGACGGGCG | 26 |  | 8 |
| **miR84** | CCAAGGAGATGATGAGGGAGATGGCCCA | 28 |  | 8 |
| **miR85** | GTACGAGAAGCGCGCGACGGAGATG | 25 |  | 8 |
| **miR86** | GCTGAGGAGGTCGACCAGAAGCTCCGCG | 28 |  | 8 |
| **miR87** | GAAGGACGCAACGTACGATGAGATCTGC | 28 |  | 8 |
| **miR88** | TGCAGAGCCCCGCCGCCTCAGTGATC | 26 |  | 8 |
| **miR89** | CTGTGATGACGGCTATGACGGAGAC | 25 |  | 8 |
| **miR90** | GCCTTGCCCGACTGAGAGTGCTCGCT | 26 |  | 8 |
| **miR91** | ATGATTCCTCTGTCCATTCCCCTGATC | 27 |  | 8 |
| **miR92** | CGCGTCGTGCAGGCCTTCAAGGATCC | 26 | SGR | 8 |
| **miR93** | TACGACAGCGGCGTTTACATCA | 22 |  | 8 |
| **miR94** | AGCCGATGCAGAAGGTCGTCTTCG | 24 |  | 8 |
| **miR95** | TTGAAGATGCCCTTCGGCGTATT | 23 |  | 8 |
| **miR96** | ATACTCTGACGCAAAGCTAAGCTGA | 25 | SGR | 8 |
| **miR97** | ACGCCTTCTGCACCAAGGAGCTGA | 24 |  | 8 |
| **miR98** | ACCTCTTCTGCGGGCTCTACGACAA | 25 |  | 8 |
| **miR99** | CTTGTAGATCCTCTTCGCGTGGTA | 24 | SGR | 8 |
| **miR100** | GGCGAGCAAGATCATCGCGTTCACGA | 26 |  | 8 |
| **miR101** | GCTCCTCTTCGTACGCCAGTGCGTGA | 26 |  | 8 |
| **miR102** | TTCGAAGCGCGGCTCAAGTTCA | 22 |  | 8 |
| **miR103** | GGACGTACTGGGCGAAGGACTACAACA | 27 | SGR | 8 |
| **miR104** | GCTCGAGCGGGAGCTCCTCGACAACT | 26 | SGR | 8 |
| **miR105** | GACTTCAAGCGCCGCGTCATCT | 22 |  | 8 |
| **miR106** | CTGCGCTGGACGATGAACTGGAGAT | 25 |  | 8 |
| **miR107** | ACCGCTCAGAGTAACTATGAGTGCTTC | 27 |  | 8 |
| **miR108** | TAGGTGGCCAGGACGAGCTGCA | 22 |  | 8 |
| **miR109** | CCTGTGATGCCGTGGGTTGCGTCGCT | 26 |  | 8 |
| **miR110** | GCAATGGCCACCCAGTGCGTGTCTCGT | 27 |  | 8 |
| **miR111** | ACCCAGGGACAGAGCAGCGAGTCA | 24 |  | 8 |

None of the previously reported putative Giardia miRNA ([Table](https://rockefeller.box.com/s/66nfi5n0n175yywixaha58roaehr8lc5) B) were called by the miRDeep2 algorithm as potential miRNA. Reasons for disqualification of potential precursors were lack of read evidence, non-mapping to the reference genome or various type of incompatibilities of the predicted structure with Dicer processing. The results of this miRDeep2 analysis are available in the [**S1 Text**](https://rockefeller.box.com/s/eao9z6hop9va8hqkgajo528473f3402c). The algorithm did yield however 58 novel suggestions for miRNA precursors (allowing up to 50% of the mapped reads to be inconsistent with Dicer processing), which are shown in [**S1 Folder**](https://rockefeller.box.com/s/dlvg6jhfau5jhls0af706ptlp5iaazqq). The top 5 candidates, in terms of consistency with canonical miRNA processing, are also depicted in **S1 Figure**. All are lowly expressed, and thus unlikely to exert significant regulation.

Notwithstanding the doubts regarding the role of miRNA in Giardia, we wished to further characterize the previously reported putative miR5 and miR6, in light of their stated abundance – a prerequisite for potential markers. Blasting against NCBI’s non-redundant nucleotide (nr/nt) collection yielded perfect alignments only with Giardia transcripts (not shown). The predicted structure and the pile up of reads mapping to the putative miR5 and miR6 precursors are shown in [**S2 Figure**](https://rockefeller.box.com/s/aiex9g49s99ngc24qoprnrlbw5r6fg3r) and [**S3 Figure**](https://rockefeller.box.com/s/8u6uy7nfv5ragy2yovoxbnd782c8ipin). The sum of reads mapping to the putative miR5 and miR6 precursors was 15980 and 18835, respectively, comprising 802 and 945 per million mapped small RNA reads (≥18 nt), likely too few to have sizeable regulatory influence [9]. Thus, while small RNA sequencing does not support the notion that miR5 and miR6 are canonical miRNA, sequences derived from their putative precursors have been found in Giardia Argonaute immune-precipitates [10], are specific to Giardia and are sufficiently abundant to serve as molecular markers.

**References**

1. Huang PJ, et al. 2012 Identification of putative miRNAs from the deep-branching unicellular flagellates. Genomics 992:101-107.

2. Zhang YQ, Chen DL, Tian HF, Zhang BH, Wen JF 2009 Genome-wide computational identification of microRNAs and their targets in the deep-branching eukaryote Giardia lamblia. Comput Biol Chem 335:391-396.

3. Chen XS, Collins LJ, Biggs PJ, Penny D 2009 High throughput genome-wide survey of small RNAs from the parasitic protists Giardia intestinalis and Trichomonas vaginalis. Genome Biol Evol 1:165-175.

4. Saraiya AA, Wang CC 2008 snoRNA, a novel precursor of microRNA in Giardia lamblia. PLoS Pathog 411:e1000224.

5. Saraiya AA, Li W, Wang CC 2011 A microRNA derived from an apparent canonical biogenesis pathway regulates variant surface protein gene expression in Giardia lamblia. RNA 1712:2152-2164.

6. Li W, Saraiya AA, Wang CC 2011 Gene regulation in Giardia lambia involves a putative microRNA derived from a small nucleolar RNA. PLoS Negl Trop Dis 510:e1338.

7. Li W, Saraiya AA, Wang CC 2012 The profile of snoRNA-derived microRNAs that regulate expression of variant surface proteins in Giardia lamblia. Cell Microbiol 149:1455-1473.

8. Saraiya AA, Li W, Wu J, Chang CH, Wang CC 2014 The microRNAs in an Ancient Protist Repress the Variant-Specific Surface Protein Expression by Targeting the Entire Coding Sequence. PLoS Pathog 102:e1003791.

9. Mullokandov G, Baccarini A, Ruzo A, Jayaprakash AD, Tung N, Israelow B, Evans MJ, Sachidanandam R, Brown BD. High-throughput assessment of microRNA activity and function using microRNA sensor and decoy libraries. Nat Methods. 2012;9(8):840-6.

10. Li W, Saraiya AA, Wang CC. Gene regulation in Giardia lambia involves a putative microRNA derived from a small nucleolar RNA. PLoS Negl Trop Dis. 2011;5(10):e1338.
