# Supplementary material for: Giardia lamblia miRNAs as a new diagnostic tool for human giardiasis
Source: PLoS Negl Trop Dis. 2019 Jun 17;13(6):e0007398. doi: 10.1371/journal.pntd.0007398 (PMC6597124; doi:10.1371/journal.pntd.0007398)
Supplement: S1 Folder — The result_16_06_2018_t_13_52_59.html file is an index, through which pdf plot can be accessed. (ZIP) [file pntd.0007398.s002.zip › S1 folder/Giardia predicted miRNAs secondary structure/GLCHR03_3173.pdf]

Provisional ID : GLCHR03_3173
Score total : 1.8
Score for star read(s) : -1.3
Score for read counts : 0
Score for mfe : 1.5
Score for randfold : 1.6
Score for cons. seed :
Total read count : 102
Mature read count : 102
Loop read count : 0
Star read count : 0

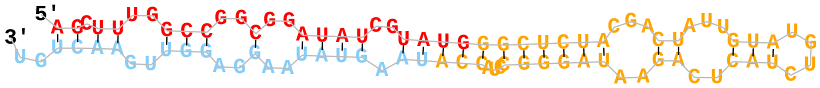

1 26 69 92

Mature Star

5'- gcuuuucuaggaagcaggacagcuuuggccggcggauaucguauggcucuacgacuauuguaugucuacucagaauagggccuaccauaaguauaaggagguugaacuguccau -3' exp

(((((.....))))).((((((.((..(((..(..((((..(((((((((((...((...(((....)))..))..))))))...))))).))))..)..)))..)))))))).. reads mm sample
