# Supplementary material for: Giardia lamblia miRNAs as a new diagnostic tool for human giardiasis
Source: PLoS Negl Trop Dis. 2019 Jun 17;13(6):e0007398. doi: 10.1371/journal.pntd.0007398 (PMC6597124; doi:10.1371/journal.pntd.0007398)
Supplement: S1 Folder — The result_16_06_2018_t_13_52_59.html file is an index, through which pdf plot can be accessed. (ZIP) [file pntd.0007398.s002.zip › S1 folder/Giardia predicted miRNAs secondary structure/GLCHR04_4042.pdf]

| | | |
|---|---|---|
| Provisional ID | : | GLCHR04_4042 |
| Score total | : | 0.8 |
| Score for star read(s) | : | -1.3 |
| Score for read counts | : | 0 |
| Score for mfe | : | 0.5 |
| Score for randfold | : | 1.6 |
| Score for cons. seed | : | |
| Total read count | : | 278 |
| Mature read count | : | 278 |
| Loop read count | : | 0 |
| Star read count | : | 0 |

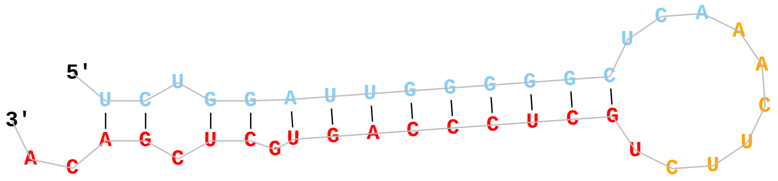


```
                                                1                     18      24                      42

                                                 Star                        Mature
5'- uaccagugacucacagucaggaaaaugcaaacaaaauaugugcaaugucuggauuggggcucaaacuucugcucccagugcucgacagcagcagagcaucuugucaa   -3'    exp
    ..((..((((.....)))))).........((((.((.(.(((..((((.((((((((((((..........))))))))).)).)))))...))).)..)).))))...    reads      mm         sample
```
