# Supplementary material for: Giardia lamblia miRNAs as a new diagnostic tool for human giardiasis
Source: PLoS Negl Trop Dis. 2019 Jun 17;13(6):e0007398. doi: 10.1371/journal.pntd.0007398 (PMC6597124; doi:10.1371/journal.pntd.0007398)
Supplement: S1 Folder — The result_16_06_2018_t_13_52_59.html file is an index, through which pdf plot can be accessed. (ZIP) [file pntd.0007398.s002.zip › S1 folder/Giardia predicted miRNAs secondary structure/GLCHR04_4458.pdf]

Provisional ID	:	GLCHR04_4458
Score total	:	44.7
Score for star read(s)	:	3.9
Score for read counts	:	40.4
Score for mfe	:	2.6
Score for randfold	:	-2.2
Score for cons. seed	:	
Total read count	:	91
Mature read count	:	89
Loop read count	:	0
Star read count	:	2

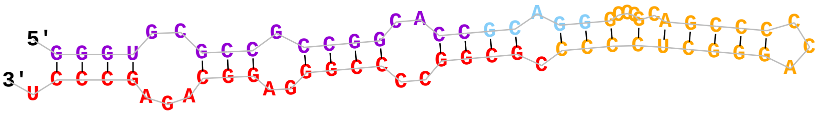


```
                      1                       19                                   47                             71

                      Star                                                         Mature
5'- agcucgcggccucgcuguacgcggggugcgccgccggcaccgcagggcccgcagcccccagggcuccccgcggccccgggaggcagagcccuccggcgcgcaggccgucgag   -3'    obs
    agcucgcggccucgcuguacgcggggugcgccgccggcaccgcagggcccgcagcccccagggcuccccgcggccccgggaggcagagcccuccggcgcgcaggccgucgag          exp
    ..((((((((((((.((.((.(((((..(((.((((..((((.(((...(.(((((...))))))))).))))..))))..)))...))))).))))))).)))))).))))   reads      mm         sample
```
