# Supplementary material for: Giardia lamblia miRNAs as a new diagnostic tool for human giardiasis
Source: PLoS Negl Trop Dis. 2019 Jun 17;13(6):e0007398. doi: 10.1371/journal.pntd.0007398 (PMC6597124; doi:10.1371/journal.pntd.0007398)
Supplement: S1 Folder — The result_16_06_2018_t_13_52_59.html file is an index, through which pdf plot can be accessed. (ZIP) [file pntd.0007398.s002.zip › S1 folder/Giardia predicted miRNAs secondary structure/GLCHR05_4933.pdf]

Provisional ID	:	GLCHR05_4933
Score total	:	2.2
Score for star read(s)	:	-1.3
Score for read counts	:	0
Score for mfe	:	1.9
Score for randfold	:	1.6
Score for cons. seed	:	
Total read count	:	149
Mature read count	:	149
Loop read count	:	0
Star read count	:	0

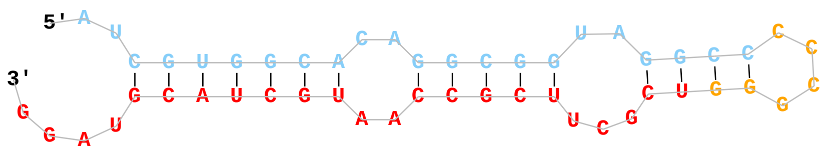

1 23 29 52

Star Mature

```
5'- auagacagcagagcucugcucaagcagauucggaauacaggcaucguggcacaggcgguaggcccccggggucgcuucgccaaugcuacguaggcaugcagcuaguaguaugug   -3'    exp
    ....(((((.(((.(((((....))))))))......((.((..(((((((..(((((..((((....))))...)))))..)))))))...)).))..))).))........   reads    mm    sample
```
