# Supplementary material for: Giardia lamblia miRNAs as a new diagnostic tool for human giardiasis
Source: PLoS Negl Trop Dis. 2019 Jun 17;13(6):e0007398. doi: 10.1371/journal.pntd.0007398 (PMC6597124; doi:10.1371/journal.pntd.0007398)
Supplement: S1 Folder — The result_16_06_2018_t_13_52_59.html file is an index, through which pdf plot can be accessed. (ZIP) [file pntd.0007398.s002.zip › S1 folder/Giardia predicted miRNAs secondary structure/GLCHR05_7206.pdf]

Provisional ID : GLCHR05_7206
Score total : 135.6
Score for star read(s) : 3.9
Score for read counts : 133.2
Score for mfe : 0.7
Score for randfold : -2.2
Score for cons. seed :
Total read count : 273
Mature read count : 224
Loop read count : 0
Star read count : 49

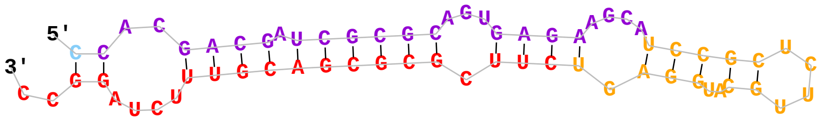

```
                            0                 26          44             66

                             Star                         Mature
5'- acuagcagauuccuuucgucgucggaccacgacgaucgcgcagugagaagcauccgcucuugcauggagucuucgcgcgacguuucuaggccgguuuaugacaacuuugaga   -3'   obs
    acuagcagauuccuuucgucgucggaccacgacgaucgcgcagugagaagcauccgcucuugcauggagucuucgcgcgacguuucuaggccgguuuaugacaacuuugaga         exp
    .................(((((((.((..((((.((((((...((((....(((((....))..))).)))).))))))))))....)))))....)))))..........   reads   mm   sample
```
