# Supplementary figures and images for: Giardia lamblia miRNAs as a new diagnostic tool for human giardiasis
Source: PLoS Negl Trop Dis. 2019 Jun 17;13(6):e0007398. doi: 10.1371/journal.pntd.0007398 (PMC6597124; doi:10.1371/journal.pntd.0007398)

**1**                      **26**                      **37**                      **58**

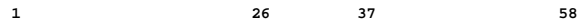

**1**                      **26**                      **37**                      **58**

**1**                      **26**                      **37**                      **58**

Supplement: S1 Folder — The result_16_06_2018_t_13_52_59.html file is an index, through which pdf plot can be accessed. (ZIP) [file pntd.0007398.s002.zip › S1 folder/Giardia predicted miRNAs secondary structure/CH991789_9112.pdf]

**26                  36                                  60**

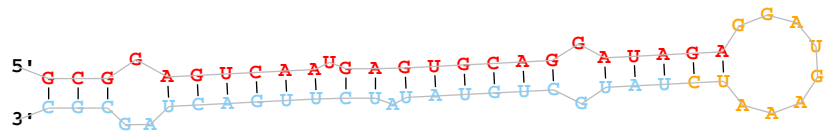

Star

[illegible]

Supplement: S1 Folder — The result_16_06_2018_t_13_52_59.html file is an index, through which pdf plot can be accessed. (ZIP) [file pntd.0007398.s002.zip › S1 folder/Giardia predicted miRNAs secondary structure/GLCHR01_711.pdf]

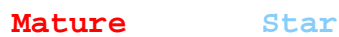[illegible]

Supplement: S1 Folder — The result_16_06_2018_t_13_52_59.html file is an index, through which pdf plot can be accessed. (ZIP) [file pntd.0007398.s002.zip › S1 folder/Giardia predicted miRNAs secondary structure/GLCHR01_779.pdf]

78

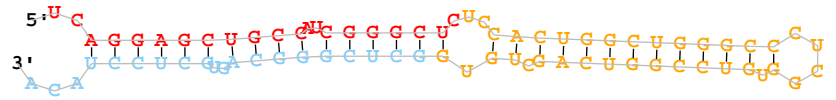

Star

[illegible]

Supplement: S1 Folder — The result_16_06_2018_t_13_52_59.html file is an index, through which pdf plot can be accessed. (ZIP) [file pntd.0007398.s002.zip › S1 folder/Giardia predicted miRNAs secondary structure/GLCHR02_1340.pdf]

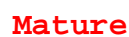[illegible]

Supplement: S1 Folder — The result_16_06_2018_t_13_52_59.html file is an index, through which pdf plot can be accessed. (ZIP) [file pntd.0007398.s002.zip › S1 folder/Giardia predicted miRNAs secondary structure/GLCHR02_1407.pdf]

|   |    |    |    |
|---|----|----|----|
| 1 | 19 | 45 | 65 |
|---|----|----|----|

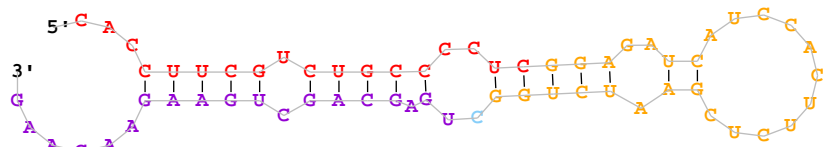

Star

[illegible]

Supplement: S1 Folder — The result_16_06_2018_t_13_52_59.html file is an index, through which pdf plot can be accessed. (ZIP) [file pntd.0007398.s002.zip › S1 folder/Giardia predicted miRNAs secondary structure/GLCHR03_2963.pdf]

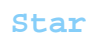

```
-3'      exp
reads      mm      sample
1          0      Gi4
```

Supplement: S1 Folder — The result_16_06_2018_t_13_52_59.html file is an index, through which pdf plot can be accessed. (ZIP) [file pntd.0007398.s002.zip › S1 folder/Giardia predicted miRNAs secondary structure/GLCHR04_3522.pdf]

64

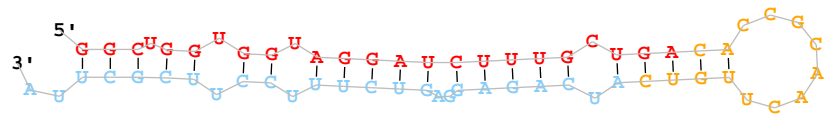

Star

[illegible]

Supplement: S1 Folder — The result_16_06_2018_t_13_52_59.html file is an index, through which pdf plot can be accessed. (ZIP) [file pntd.0007398.s002.zip › S1 folder/Giardia predicted miRNAs secondary structure/GLCHR04_3830.pdf]

1 :            0            19

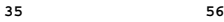

Star

-3'      exp

reads mm

sample

Supplement: S1 Folder — The result_16_06_2018_t_13_52_59.html file is an index, through which pdf plot can be accessed. (ZIP) [file pntd.0007398.s002.zip › S1 folder/Giardia predicted miRNAs secondary structure/GLCHR04_4509.pdf]

Mature

Star

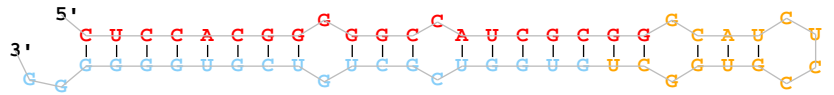[illegible]

Supplement: S1 Folder — The result_16_06_2018_t_13_52_59.html file is an index, through which pdf plot can be accessed. (ZIP) [file pntd.0007398.s002.zip › S1 folder/Giardia predicted miRNAs secondary structure/GLCHR05_6393.pdf]

>GLCHR03\_2019

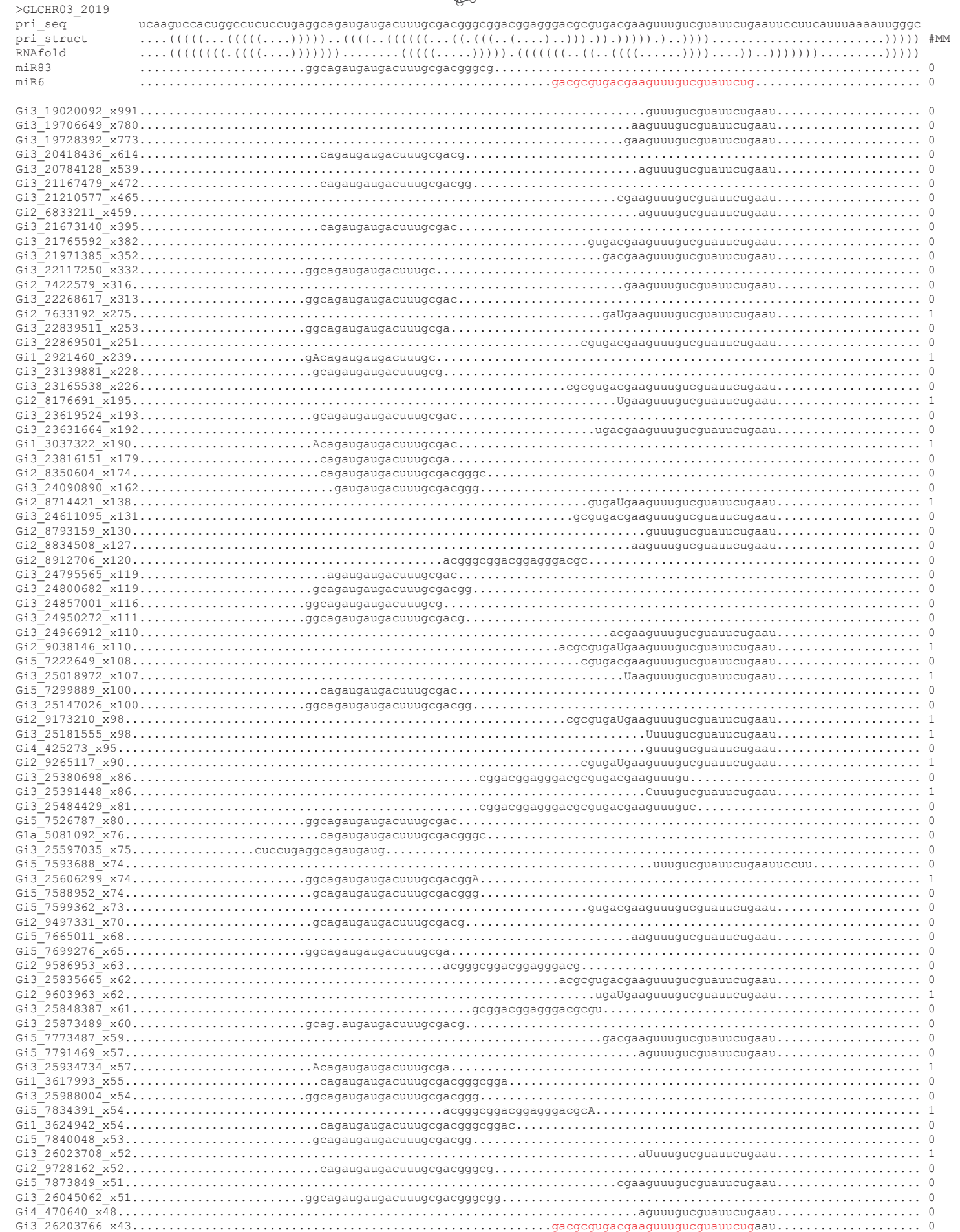

Supplement: S3 Fig — The sequences are sorted by abundance and their source library is specified (as in S1 Text). (PDF) [file pntd.0007398.s009.pdf]
