# Supplementary material for: Giardia lamblia miRNAs as a new diagnostic tool for human giardiasis
Source: PLoS Negl Trop Dis. 2019 Jun 17;13(6):e0007398. doi: 10.1371/journal.pntd.0007398 (PMC6597124; doi:10.1371/journal.pntd.0007398)
Supplement: S1 Table — We specifically sought these sequences in the current analysis, by including them as presumed known mature miRNA in the miRDeep2 analysis. (DOCX) [file pntd.0007398.s004.docx]

Table S1. Previously identified miRNAs from Giardia lamblia (modified from Liao et al, Both endo-siRNAs and tRNA-derived small RNAs are involved in the differentiation of primitive eukaryote Giardia lamblia. Proc Natl Acad Sci U S A. 2014 Sep 30;111(39):14159-64)

|  | |  | | | | | | | |  | |  | | | |  | |  | | | | | | |  | | |  |  |  |  |  |  |  |  |  |
| --- | --- | --- | --- | --- | --- | --- | --- | --- | --- | --- | --- | --- | --- | --- | --- | --- | --- | --- | --- | --- | --- | --- | --- | --- | --- | --- | --- | --- | --- | --- | --- | --- | --- | --- | --- | --- |
|  | |  |  |  |  | **NCBI blastn output with GiardiaDB-41 genomes as subjects** | | | | | | | | | | | | | | | | | | | | | | |  |  |  |  |  |  |  |  |
|  | |  |  |  |  | **assemblage A best hit** | | | **assemblage B best hit** | | | | | **assemblage E best hit** | | | | | | | **human best hit** | | | | | | | |  |  |  |  |  |  |  |  |
| **miRNA** | **Sequence** | | **Length, nt** | **Overlap with known ncRNA** | **Ref.** | **% identity** | **alignment length** | **evalue** | **% identity** | | **alignment length** | | **evalue** | **% identity** | **alignment length** | | **evalue** | | | | **% identity** | | **alignment length** | | | **evalue** | | |  |  |  |  |  |  |  |  |
| **glm-001** | GCTGGGATCGTATAAAGG | | 18 | tRNA-HisGTG | 1 | 100 | 18 | 8.26E-04 | 100 | | 18 | | 7.73E-04 | 100 | 18 | | 7.42E-04 | | | | 100 | | 15 | | | 8.4 | | |  |  |  |  |  |  |  |  |
| **Gl-mir-1** | CTCATCTATACACAGTGTCGC | | 21 |  | 2 | 100 | 21 | 2.14E-05 | 100 | | 14 | | 0.3 | 100 | 14 | | 0.29 | | | | 100 | | 17 | | | 0.54 | | |  |  |  |  |  |  |  |  |
| **Gl-mir-2** | GCAGGAGTTGCAGCGCAGTGC | | 21 |  | 2 | 100 | 21 | 2.14E-05 | 100 | | 13 | | 1.2 | 100 | 14 | | 0.29 | | | | 100 | | 16 | | | 2.1 | | |  |  |  |  |  |  |  |  |
| **Gl-mir-3** | CTCTACCTCCTGACTGCATTG | | 21 |  | 2 | 100 | 21 | 2.14E-05 | 100 | | 14 | | 0.3 | 100 | 13 | | 1.1 | | | | 100 | | 18 | | | 0.14 | | |  |  |  |  |  |  |  |  |
| **Gl-mir-4** | ATCGCACACCATAGCCTTGTG | | 21 |  | 2 | 100 | 21 | 2.14E-05 | 100 | | 13 | | 1.2 | 100 | 13 | | 1.1 | | | | 100 | | 17 | | | 0.54 | | |  |  |  |  |  |  |  |  |
| **Gl-mir-5** | AGCAGAGAAAGTGTCTTTCCG | | 21 |  | 2 | 100 | 21 | 2.14E-05 | 100 | | 14 | | 0.3 | 95 | 20 | | 0.019 | | | | 100 | | 17 | | | 0.54 | | |  |  |  |  |  |  |  |  |
| **Gl-mir-6** | AGGTCGGACTCGCGCGCCTCC | | 21 |  | 2 | 100 | 21 | 2.14E-05 | 100 | | 17 | | 0.005 | 100 | 21 | | 1.92E-05 | | | | 100 | | 16 | | | 2.1 | | |  |  |  |  |  |  |  |  |
| **Gl-mir-7** | GGACCAGGCCGTCGTCGAAGT | | 21 |  | 2 | 100 | 21 | 2.14E-05 | 100 | | 16 | | 0.019 | 95 | 21 | | 0.005 | | | | 100 | | 18 | | | 0.14 | | |  |  |  |  |  |  |  |  |
| **Gl-mir-8** | TAGCACGGCGATCTGTGTCTG | | 21 |  | 2 | 100 | 21 | 2.14E-05 | 100 | | 14 | | 0.3 | 95 | 19 | | 0.073 | | | |  | |  | | |  | | |  |  |  |  |  |  |  |  |
| **Gl-mir-9** | AGGACGTAGATGATGACATCT | | 21 |  | 2 | 100 | 21 | 2.14E-05 | 100 | | 14 | | 0.3 | 100 | 15 | | 0.073 | | | | 100 | | 17 | | | 0.54 | | |  |  |  |  |  |  |  |  |
| **Gl-mir-10** | GAGGTCTTCTTCCAAGGCAAT | | 21 |  | 2 | 100 | 21 | 2.14E-05 | 100 | | 14 | | 0.3 | 100 | 14 | | 0.29 | | | | 100 | | 17 | | | 0.54 | | |  |  |  |  |  |  |  |  |
| **Gl-mir-11** | CTATAAGAAGAGATATCTTGT | | 21 |  | 2 | 100 | 21 | 2.14E-05 | 100 | | 14 | | 0.3 | 100 | 14 | | 0.29 | | | | 100 | | 18 | | | 0.14 | | |  |  |  |  |  |  |  |  |
| **Gl-mir-12** | TAGCTTATGGTAGTTGCACCT | | 21 |  | 2 | 100 | 21 | 2.14E-05 | 100 | | 13 | | 1.2 | 100 | 13 | | 1.1 | | | | 100 | | 17 | | | 0.54 | | |  |  |  |  |  |  |  |  |
| **Gl-mir-13** | CGTGCGCACCCCCCTTGGTAT | | 21 |  | 2 | 100 | 21 | 2.14E-05 | 100 | | 12 | | 4.7 | 100 | 13 | | 1.1 | | | |  | |  | | |  | | |  |  |  |  |  |  |  |  |
| **Gl-mir-14** | GACGAAGATCGCGCCCCACGA | | 21 |  | 2 | 100 | 21 | 2.14E-05 | 100 | | 13 | | 1.2 | 100 | 16 | | 0.019 | | | |  | |  | | |  | | |  |  |  |  |  |  |  |  |
| **Gl-mir-15** | CATCCATGTAGACATCTTGCG | | 21 |  | 2 | 100 | 21 | 2.14E-05 | 100 | | 14 | | 0.3 | 100 | 14 | | 0.29 | | | | 100 | | 16 | | | 2.1 | | |  |  |  |  |  |  |  |  |
| **Gl-mir-16** | TGGGAGCGTGTGCACAGGCAG | | 21 |  | 2 | 100 | 21 | 2.14E-05 | 100 | | 14 | | 0.3 | 100 | 14 | | 0.29 | | | | 100 | | 16 | | | 2.1 | | |  |  |  |  |  |  |  |  |
| **Gl-mir-17** | AGTGCGAGGACCAACGCGGTT | | 21 |  | 2 | 100 | 21 | 2.14E-05 | 100 | | 13 | | 1.2 | 100 | 13 | | 1.1 | | | | 100 | | 16 | | | 2.1 | | |  |  |  |  |  |  |  |  |
| **Gl-mir-18** | CCCCTCTGTGGACTCGTGAGT | | 21 |  | 2 | 100 | 21 | 2.14E-05 | 100 | | 14 | | 0.3 | 100 | 13 | | 1.1 | | | | 100 | | 17 | | | 0.54 | | |  |  |  |  |  |  |  |  |
| **Gl-mir-19** | GTCCGATTACCAACCCTTTGA | | 21 |  | 2 | 100 | 21 | 2.14E-05 | 100 | | 14 | | 0.3 | 100 | 14 | | 0.29 | | | | 100 | | 16 | | | 2.1 | | |  |  |  |  |  |  |  |  |
| **Gl-mir-20** | TGAGCAGATCTCAGAGGTATA | | 21 |  | 2 | 100 | 21 | 2.14E-05 | 100 | | 15 | | 0.076 | 95 | 20 | | 0.019 | | | | 95.238 | | 21 | | | 0.54 | | |  |  |  |  |  |  |  |  |
| **Gl-mir-21** | TGAGTACTGGTTTTGCACCTT | | 21 |  | 2 | 100 | 21 | 2.14E-05 | 100 | | 13 | | 1.2 | 100 | 13 | | 1.1 | | | | 100 | | 17 | | | 0.54 | | |  |  |  |  |  |  |  |  |
| **Gl-mir-22** | TGTAACAGTCATACATGTACA | | 21 |  | 2 | 100 | 21 | 2.14E-05 | 100 | | 14 | | 0.3 | 100 | 13 | | 1.1 | | | | 100 | | 18 | | | 0.14 | | |  |  |  |  |  |  |  |  |
| **Gl-mir-23** | CGTGCATGGAATAGTGTGTAT | | 21 |  | 2 | 100 | 21 | 2.14E-05 | 100 | | 16 | | 0.019 | 95 | 21 | | 0.005 | | | | 100 | | 16 | | | 2.1 | | |  |  |  |  |  |  |  |  |
| **Gl-mir-24** | TGTCTTTTGAAGAGCTTTGTG | | 21 |  | 2 | 100 | 21 | 2.14E-05 | 100 | | 16 | | 0.019 | 100 | 18 | | 0.001 | | | | 100 | | 18 | | | 0.14 | | |  |  |  |  |  |  |  |  |
| **Gl-mir-25** | AGATCTGGATCGCCTTCAGCT | | 21 |  | 2 | 100 | 21 | 2.14E-05 | 100 | | 14 | | 0.3 | 100 | 14 | | 0.29 | | | | 100 | | 17 | | | 0.54 | | |  |  |  |  |  |  |  |  |
| **Gl-mir-26** | CGTGCGGGCCCGGGGGCAGGG | | 21 |  | 2 | 100 | 21 | 2.14E-05 | 100 | | 14 | | 0.3 | 100 | 15 | | 0.073 | | | | 100 | | 18 | | | 0.14 | | |  |  |  |  |  |  |  |  |
| **Gl-mir-27** | TTGGCTACTGCAGTCAGCTCT | | 21 |  | 2 | 100 | 21 | 2.14E-05 | 100 | | 13 | | 1.2 | 94 | 18 | | 0.29 | | | | 100 | | 17 | | | 0.54 | | |  |  |  |  |  |  |  |  |
| **Gl-mir-28** | TGAGATCATAGAGCCGCTTTG | | 21 |  | 2 | 100 | 21 | 2.14E-05 | 100 | | 15 | | 0.076 | 95 | 21 | | 0.005 | | | | 100 | | 17 | | | 0.54 | | |  |  |  |  |  |  |  |  |
| **Gl-mir-29** | CCCTTCAAATCCCTGATTATG | | 21 |  | 2 | 100 | 21 | 2.14E-05 | 94 | | 18 | | 0.3 | 95 | 21 | | 0.005 | | | | 100 | | 20 | | | 0.009 | | |  |  |  |  |  |  |  |  |
| **Gl-mir-30** | CAGCGTGTGTGTCCTGCCGAG | | 21 |  | 2 | 100 | 21 | 2.14E-05 | 100 | | 13 | | 1.2 | 100 | 13 | | 1.1 | | | | 100 | | 17 | | | 0.54 | | |  |  |  |  |  |  |  |  |
| **Gl-mir-31** | CGAGGAGACGTTGGAGAATGT | | 21 |  | 2 | 100 | 21 | 2.14E-05 | 94 | | 18 | | 0.3 | 95 | 19 | | 0.073 | | | | 100 | | 17 | | | 0.54 | | |  |  |  |  |  |  |  |  |
| **Gl-mir-32** | GTAGCAAAGATCCTTCTTGCT | | 21 |  | 2 | 100 | 21 | 2.14E-05 | 95 | | 19 | | 0.076 | 95 | 19 | | 0.073 | | | | 100 | | 17 | | | 0.54 | | |  |  |  |  |  |  |  |  |
| **Gl-mir-33** | ATCGAAAGCACTCTGGAGGCT | | 21 |  | 2 | 100 | 21 | 2.14E-05 | 100 | | 14 | | 0.3 | 100 | 21 | | 1.92E-05 | | | | 100 | | 17 | | | 0.54 | | |  |  |  |  |  |  |  |  |
| **Gl-mir-34** | CGCCGTCACGGAGGTCCGCCG | | 21 |  | 2 | 100 | 21 | 2.14E-05 | 100 | | 12 | | 4.7 | 100 | 13 | | 1.1 | | | | 100 | | 16 | | | 2.1 | | |  |  |  |  |  |  |  |  |
| **Gl-mir-35** | AGTGTATCTAAAACCTGGCAC | | 21 |  | 2 | 100 | 21 | 2.14E-05 | 100 | | 14 | | 0.3 | 100 | 13 | | 1.1 | | | | 100 | | 18 | | | 0.14 | | |  |  |  |  |  |  |  |  |
| **Gl-mir-36** | TGCCAGGTTTTAGATACACTA | | 21 |  | 2 | 100 | 21 | 2.14E-05 | 100 | | 15 | | 0.076 | 100 | 13 | | 1.1 | | | | 100 | | 17 | | | 0.54 | | |  |  |  |  |  |  |  |  |
| **Gl-mir-37** | ATCCATGTTGTAGCGCTGGTT | | 21 |  | 2 | 100 | 21 | 2.14E-05 | 100 | | 12 | | 4.7 | 100 | 20 | | 7.60E-05 | | | | 100 | | 16 | | | 2.1 | | |  |  |  |  |  |  |  |  |
| **Gl-mir-38** | TCCCTGAAGGTACTTGGCTAA | | 21 |  | 2 | 100 | 21 | 2.14E-05 | 100 | | 16 | | 0.019 | 100 | 14 | | 0.29 | | | | 100 | | 17 | | | 0.54 | | |  |  |  |  |  |  |  |  |
| **Gl-mir-39** | CCCAGAGTGTCGTCTCTCAGC | | 21 |  | 2 | 100 | 21 | 2.14E-05 | 100 | | 14 | | 0.3 | 100 | 14 | | 0.29 | | | |  | |  | | |  | | |  |  |  |  |  |  |  |  |
| **Gl-mir-40** | TCCGGTAGGTATTTTATAAAT | | 21 |  | 2 | 100 | 21 | 2.14E-05 | 100 | | 13 | | 1.2 | 100 | 14 | | 0.29 | | | | 100 | | 18 | | | 0.14 | | |  |  |  |  |  |  |  |  |
| **Gl-mir-41** | CCCGTTTCTTGGGGTGCCTCT | | 21 |  | 2 | 100 | 21 | 2.14E-05 | 100 | | 12 | | 4.7 | 100 | 14 | | 0.29 | | | | 100 | | 17 | | | 0.54 | | |  |  |  |  |  |  |  |  |
| **Gl-mir-42** | CCAGTCATACATGTACACAGT | | 21 |  | 2 | 100 | 21 | 2.14E-05 | 100 | | 14 | | 0.3 | 100 | 14 | | 0.29 | | | | 100 | | 17 | | | 0.54 | | |  |  |  |  |  |  |  |  |
| **Gl-mir-43** | GATGGGTGGTAATGAGGCCTG | | 21 |  | 2 | 100 | 21 | 2.14E-05 | 100 | | 12 | | 4.7 | 100 | 13 | | 1.1 | | | | 100 | | 17 | | | 0.54 | | |  |  |  |  |  |  |  |  |
| **Gl-mir-44** | AAGCTGCTTGTTTGTGAAGTC | | 21 |  | 2 | 100 | 21 | 2.14E-05 | 100 | | 14 | | 0.3 | 100 | 14 | | 0.29 | | | | 100 | | 18 | | | 0.14 | | |  |  |  |  |  |  |  |  |
| **Gl-mir-45** | TGGATTCAGGCCGCGCGTGTT | | 21 |  | 2 | 100 | 21 | 2.14E-05 | 100 | | 13 | | 1.2 | 100 | 14 | | 0.29 | | | |  | |  | | |  | | |  |  |  |  |  |  |  |  |
| **Gl-mir-46** | GAGCAGTAGCTGCCTTTGATA | | 21 |  | 2 | 100 | 21 | 2.14E-05 | 100 | | 14 | | 0.3 | 100 | 13 | | 1.1 | | | | 100 | | 20 | | | 0.009 | | |  |  |  |  |  |  |  |  |
| **Gl-mir-47** | TTCGAAATAGGAGCCAAGAGT | | 21 |  | 2 | 100 | 21 | 2.14E-05 | 94 | | 18 | | 0.3 | 100 | 14 | | 0.29 | | | | 100 | | 18 | | | 0.14 | | |  |  |  |  |  |  |  |  |
| **Gl-mir-48** | GGCGTCCATGCACCTGACGCT | | 21 |  | 2 | 100 | 21 | 2.14E-05 | 100 | | 15 | | 0.076 | 100 | 17 | | 0.005 | | | | 100 | | 17 | | | 0.54 | | |  |  |  |  |  |  |  |  |
| **Gl-mir-49** | AGGTGCATGGACGCCGGGCGC | | 21 |  | 2 | 100 | 21 | 2.14E-05 | 94 | | 17 | | 1.2 | 95 | 20 | | 0.019 | | | | 95.238 | | 21 | | | 0.54 | | |  |  |  |  |  |  |  |  |
| **Gl-mir-50** | CACGAGAGTGCGCGACCGGGG | | 21 |  | 2 | 100 | 21 | 2.14E-05 | 100 | | 14 | | 0.3 | 100 | 13 | | 1.1 | | | |  | |  | | |  | | |  |  |  |  |  |  |  |  |
| **Gim1** | ATCAACGTGACTGATGCTGGCTCT | | 24 |  | 3 | 100 | 24 | 4.34E-07 | 100 | | 15 | | 0.1 | 100 | 13 | | 1.6 | | | | 100 | | 17 | | | 0.54 | | |  |  |  |  |  |  |  |  |
| **Gim2** | ATCTCGCACATATACCGGCCTCCT | | 24 |  | 3 | 100 | 24 | 4.34E-07 | 100 | | 15 | | 0.1 | 100 | 13 | | 1.6 | | | |  | |  | | |  | | |  |  |  |  |  |  |  |  |
| **Gim3** | GTGCAGAGGCATGGAGCACGGGAA | | 24 | U0121 | 3 | 100 | 24 | 4.34E-07 | 100 | | 16 | | 0.027 | 100 | 15 | | 0.1 | | | | 100 | | 17 | | | 0.54 | | |  |  |  |  |  |  |  |  |
| **Gim4** | GTGGTCTGCATCTGGACCTTCACT | | 24 |  | 3 | 100 | 24 | 4.34E-07 | 100 | | 14 | | 0.41 | 100 | 14 | | 0.4 | | | | 100 | | 17 | | | 0.54 | | |  |  |  |  |  |  |  |  |
| **Gim5** | GGCCGTGTGGTTAGGTGGTTGTTG | | 24 |  | 3 | 100 | 24 | 4.34E-07 | 100 | | 14 | | 0.41 | 94 | 17 | | 1.6 | | | | 100 | | 17 | | | 0.54 | | |  |  |  |  |  |  |  |  |
| **Gim6** | GTGGTGAGTAGAAGTCAGATTATAA | | 25 |  | 3 | 100 | 25 | 1.21E-07 | 100 | | 15 | | 0.1 | 100 | 12 | | 6.2 | | | | 100 | | 21 | | | 0.002 | | |  |  |  |  |  |  |  |  |
| **Gim7** | GCGGTCGCTTGGGTCCCAGCGGGTTC | | 26 |  | 3 | 100 | 26 | 3.34E-08 | 100 | | 15 | | 0.11 | 100 | 13 | | 1.7 | | | | 100 | | 19 | | | 0.035 | | |  |  |  |  |  |  |  |  |
| **Gim8** | GGTCGGTTAGCTCAGTCGGTAGAGCG | | 26 | tRNA-IleGAT | 3 | 100 | 26 | 3.34E-08 | 100 | | 26 | | 3.12E-08 | 100 | 26 | | 3.00E-08 | | | | 95.455 | | 22 | | | 0.035 | | |  |  |  |  |  |  |  |  |
| **Gim9** | GTAGGATGCCCCAGAGACTGCCGAG | | 25 |  | 3 | 100 | 20 | 1.16E-04 | 100 | | 13 | | 1.6 | 100 | 13 | | 1.6 | | | | 100 | | 17 | | | 0.54 | | |  |  |  |  |  |  |  |  |
| **Gim10** | AAACTCTCCGCACAGGGGCGCGCCTG | | 26 |  | 3 | 100 | 26 | 3.34E-08 | 100 | | 13 | | 1.8 | 100 | 13 | | 1.7 | | | | 100 | | 16 | | | 2.1 | | |  |  |  |  |  |  |  |  |
| **miR2** | CAGCCTAATCACCGCCCCTATAGTCC | | 26 | GlsR17 | 4 | 100 | 26 | 3.34E-08 | 100 | | 15 | | 0.11 | 100 | 26 | | 3.00E-08 | | | | 100 | | 17 | | | 0.54 | | |  |  |  |  |  |  |  |  |
| **miR3** | GCAGACAACGCATCACCGCTCTGACC | | 26 | GlsR16 | 4 | 100 | 26 | 3.34E-08 | 100 | | 26 | | 3.12E-08 | 100 | 26 | | 3.00E-08 | | | |  | |  | | |  | | |  |  |  |  |  |  |  |  |
| **miR4** | TCTGCACCAAGGAGCTGATCCAGACC | | 26 | SGR | 5 | 100 | 26 | 3.34E-08 | 100 | | 16 | | 0.029 | 100 | 14 | | 0.43 | | | | 100 | | 18 | | | 0.14 | | |  |  |  |  |  |  |  |  |
| **miR5** | GATGCTTCCTTGGATGTCCGAGCCTT | | 26 | GlsR2 | 6 | 100 | 26 | 3.34E-08 | 100 | | 26 | | 3.12E-08 | 100 | 26 | | 3.00E-08 | | | | 95 | | 20 | | | 2.1 | | |  |  |  |  |  |  |  |  |
| **miR6** | GACGCGTGACGAAGTTTGTCGTATTCTG | | 28 | GlsR1 | 7 | 100 | 28 | 2.49E-09 | 92 | | 24 | | 0.034 | 100 | 28 | | 2.24E-09 | | | |  | |  | | |  | | |  |  |  |  |  |  |  |  |
| **miR10** | TGAGGAAGAAACCGCCTTTCGTCTGACC | | 28 | GlsR8 | 7 | 100 | 28 | 2.49E-09 | 100 | | 26 | | 3.64E-08 | 100 | 28 | | 2.24E-09 | | | | 100 | | 17 | | | 0.54 | | |  |  |  |  |  |  |  |  |
| **miR11** | ATGTCTGCCGTGTGCGCGCATATCCT | | 26 |  | 8 | 100 | 26 | 3.34E-08 | 96 | | 26 | | 7.61E-06 | 100 | 25 | | 1.18E-07 | | | |  | |  | | |  | | |  |  |  |  |  |  |  |  |
| **miR12** | TGTGTTTCTAGTAAACAGTCGGGATC | | 26 |  | 8 | 100 | 26 | 3.34E-08 | 100 | | 24 | | 4.88E-07 | 100 | 26 | | 3.00E-08 | | | | 100 | | 19 | | | 0.035 | | |  |  |  |  |  |  |  |  |
| **miR13** | CCTGATGGAGAAACCTTCCTGGACGG | | 26 |  | 8 | 100 | 26 | 3.34E-08 | 100 | | 13 | | 1.8 | 91 | 23 | | 0.11 | | | | 95.238 | | 21 | | | 0.54 | | |  |  |  |  |  |  |  |  |
| **miR14** | AGGCGCAGGCCCGCCGCGAGGG | | 22 |  | 8 | 100 | 22 | 6.10E-06 | 100 | | 15 | | 0.086 | 100 | 22 | | 5.48E-06 | | | | 100 | | 17 | | | 0.54 | | |  |  |  |  |  |  |  |  |
| **miR15** | CCCGTGAGGTCTGCCAGGAGGAGGGT | | 26 |  | 8 | 100 | 26 | 3.34E-08 | 96 | | 25 | | 3.01E-05 | 92 | 24 | | 0.028 | | | | 100 | | 18 | | | 0.14 | | |  |  |  |  |  |  |  |  |
| **miR18** | ACCACAAACATCGACACGGAGAGCTGC | | 27 |  | 8 | 100 | 27 | 9.15E-09 | 93 | | 27 | | 5.09E-04 | 100 | 18 | | 0.002 | | | | 100 | | 16 | | | 2.1 | | |  |  |  |  |  |  |  |  |
| **miR19** | GTTCTTTTCGCGTGAGCTGGTCGACGCC | | 28 |  | 8 | 100 | 28 | 2.49E-09 | 96 | | 27 | | 2.25E-06 | 96 | 28 | | 5.46E-07 | | | |  | |  | | |  | | |  |  |  |  |  |  |  |  |
| **miR20** | GACGTGCTAGGTCAGGCAGACGGGCTCC | | 28 |  | 8 | 100 | 28 | 2.49E-09 | 96 | | 23 | | 5.48E-04 | 93 | 28 | | 1.33E-04 | | | | 100 | | 17 | | | 0.54 | | |  |  |  |  |  |  |  |  |
| **miR21** | ATGGGCTGGGTTAAGGTTCAGAAGACGC | | 28 |  | 8 | 100 | 28 | 2.49E-09 | 93 | | 28 | | 1.39E-04 | 100 | 28 | | 2.24E-09 | | | | 100 | | 19 | | | 0.035 | | |  |  |  |  |  |  |  |  |
| **miR22** | AAATTGACAAGAAGATCGCTGAGGCA | | 26 |  | 8 | 100 | 26 | 3.34E-08 | 96 | | 23 | | 4.70E-04 | 100 | 24 | | 4.68E-07 | | | | 95.238 | | 21 | | | 0.54 | | |  |  |  |  |  |  |  |  |
| **miR23** | CGTTTGCCGTCTTACAATGCTCTGACC | | 27 |  | 8 | 100 | 27 | 9.15E-09 | 96 | | 23 | | 5.09E-04 | 100 | 27 | | 8.21E-09 | | | | 100 | | 17 | | | 0.54 | | |  |  |  |  |  |  |  |  |
| **miR24** | TTCGCCTGGATCGCATAGGCAA | | 22 |  | 8 | 100 | 22 | 6.10E-06 | 100 | | 16 | | 0.022 | 100 | 14 | | 0.33 | | | | 100 | | 16 | | | 2.1 | | |  |  |  |  |  |  |  |  |
| **miR25** | TGGGCCCAAAAAAACGAAAGCGTCCC | | 26 | SGR | 8 | 100 | 26 | 3.34E-08 | 100 | | 12 | | 7.1 | 100 | 22 | | 7.30E-06 | | | |  | |  | | |  | | |  |  |  |  |  |  |  |  |
| **miR26** | TCACGGTCTCGCAGATCATGCGCAA | | 25 |  | 8 | 100 | 25 | 1.21E-07 | 96 | | 25 | | 2.76E-05 | 100 | 25 | | 1.08E-07 | | | | 100 | | 16 | | | 2.1 | | |  |  |  |  |  |  |  |  |
| **miR27** | TAGTGTAGGGATGTCCAAAAAACCAG | | 26 |  | 8 | 100 | 26 | 3.34E-08 | 100 | | 15 | | 0.11 | 96 | 26 | | 7.30E-06 | | | | 100 | | 19 | | | 0.035 | | |  |  |  |  |  |  |  |  |
| **miR28** | TACTACGACGAGGGCAAGAACACGAA | | 26 | SGR | 8 | 100 | 26 | 3.34E-08 | 100 | | 24 | | 4.88E-07 | 100 | 26 | | 3.00E-08 | | | |  | |  | | |  | | |  |  |  |  |  |  |  |  |
| **miR29** | TACAACGGCGTGCCCCAGACCTTCAA | | 26 | SGR | 8 | 100 | 26 | 3.34E-08 | 100 | | 13 | | 1.8 | 100 | 26 | | 3.00E-08 | | | | 95.238 | | 21 | | | 0.54 | | |  |  |  |  |  |  |  |  |
| **miR30** | GACGGGGCGGGAGGATTTGCCA | | 22 |  | 8 | 100 | 22 | 6.10E-06 | 100 | | 13 | | 1.3 | 100 | 15 | | 0.082 | | | | 100 | | 17 | | | 0.54 | | |  |  |  |  |  |  |  |  |
| **miR31** | TACCACCGCGACGACGACCCCCACA | | 25 | SGR | 8 | 100 | 25 | 1.21E-07 | 95 | | 19 | | 0.1 | 96 | 23 | | 4.13E-04 | | | | 100 | | 16 | | | 2.1 | | |  |  |  |  |  |  |  |  |
| **miR32** | TGCTTCCACGGGGCGAACAATG | | 22 |  | 8 | 100 | 22 | 6.10E-06 | 95 | | 20 | | 0.022 | 100 | 14 | | 0.33 | | | | 100 | | 17 | | | 0.54 | | |  |  |  |  |  |  |  |  |
| **miR33** | TACGAGATGGAGAGCAGACGGACC | | 24 | SGR | 8 | 100 | 24 | 4.34E-07 | 100 | | 16 | | 0.027 | 100 | 14 | | 0.4 | | | | 100 | | 17 | | | 0.54 | | |  |  |  |  |  |  |  |  |
| **miR34** | TCTACACCCTCAAGGGCCACCCGAA | | 25 |  | 8 | 100 | 25 | 1.21E-07 | 95 | | 22 | | 0.002 | 95 | 22 | | 0.002 | | | | 100 | | 17 | | | 0.54 | | |  |  |  |  |  |  |  |  |
| **miR35** | CATCAACCGCCAGACGGAGAAGGGCC | | 26 | SGR | 8 | 100 | 26 | 3.34E-08 | 100 | | 26 | | 3.12E-08 | 92 | 24 | | 0.028 | | | | 100 | | 17 | | | 0.54 | | |  |  |  |  |  |  |  |  |
| **miR36** | AGCCCCTCCGGCGAGATGTTCG | | 22 |  | 8 | 100 | 22 | 6.10E-06 | 100 | | 12 | | 5.3 | 95 | 19 | | 0.082 | | | |  | |  | | |  | | |  |  |  |  |  |  |  |  |
| **miR37** | CGGTCGAGGCGTTGAGGAAGCA | | 22 |  | 8 | 100 | 22 | 6.10E-06 | 100 | | 14 | | 0.34 | 95 | 21 | | 0.005 | | | | 100 | | 17 | | | 0.54 | | |  |  |  |  |  |  |  |  |
| **miR38** | CCCGCCCAGTAAACAAGCCCTGCA | | 24 |  | 8 | 100 | 24 | 4.34E-07 | 100 | | 14 | | 0.41 | 100 | 14 | | 0.4 | | | | 100 | | 17 | | | 0.54 | | |  |  |  |  |  |  |  |  |
| **miR39** | CCTGGGATAATGCGCTTCTTTGAGCCGCG | | 29 |  | 8 | 100 | 29 | 6.76E-10 | 100 | | 22 | | 9.52E-06 | 100 | 29 | | 6.07E-10 | | | |  | |  | | |  | | |  |  |  |  |  |  |  |  |
| **miR40** | AGCCTAATCACCGCCCCTATAGTCC | | 25 |  | 8 | 100 | 25 | 1.21E-07 | 100 | | 15 | | 0.1 | 100 | 25 | | 1.08E-07 | | | | 100 | | 17 | | | 0.54 | | |  |  |  |  |  |  |  |  |
| **miR41** | GTTCGAGGGCGCGGAGCACATTCCAAA | | 27 |  | 8 | 100 | 27 | 9.15E-09 | 100 | | 13 | | 1.9 | 91 | 23 | | 0.12 | | | |  | |  | | |  | | |  |  |  |  |  |  |  |  |
| **miR42** | GACCTCACCAAGATTTGCAAGGACGCTG | | 28 |  | 8 | 100 | 28 | 2.49E-09 | 100 | | 15 | | 0.13 | 94 | 17 | | 2 | | | | 100 | | 18 | | | 0.14 | | |  |  |  |  |  |  |  |  |
| **miR43** | CTCATTGTGAAGGAGGCAGACCCAGG | | 26 |  | 8 | 100 | 26 | 3.34E-08 | 100 | | 15 | | 0.11 | 92 | 26 | | 0.002 | | | | 100 | | 19 | | | 0.035 | | |  |  |  |  |  |  |  |  |
| **miR44** | TCTGCGACGATCGGGTAGACGATGC | | 25 | SGR | 8 | 100 | 25 | 1.21E-07 | 100 | | 22 | | 6.98E-06 | 100 | 25 | | 1.08E-07 | | | | 100 | | 16 | | | 2.1 | | |  |  |  |  |  |  |  |  |
| **miR45** | TATTCCATTCACTCAGTCAGACCCAG | | 26 |  | 8 | 100 | 26 | 3.34E-08 | 100 | | 14 | | 0.45 | 100 | 26 | | 3.00E-08 | | | | 100 | | 17 | | | 0.54 | | |  |  |  |  |  |  |  |  |
| **miR46** | TGGTCCCAGTAGCAATAGACGTAG | | 24 |  | 8 | 100 | 24 | 4.34E-07 | 95 | | 20 | | 0.027 | 100 | 20 | | 1.05E-04 | | | | 100 | | 18 | | | 0.14 | | |  |  |  |  |  |  |  |  |
| **miR47** | CGACGTCATCCACGCGAAGATCCGCTC | | 27 | SGR | 8 | 100 | 27 | 9.15E-09 | 96 | | 27 | | 2.09E-06 | 100 | 27 | | 8.21E-09 | | | |  | |  | | |  | | |  |  |  |  |  |  |  |  |
| **miR48** | TGAAGGGCCTCGACACGAATCCGAAGA | | 27 | SGR | 8 | 100 | 27 | 9.15E-09 | 100 | | 15 | | 0.12 | 100 | 27 | | 8.21E-09 | | | |  | |  | | |  | | |  |  |  |  |  |  |  |  |
| **miR49** | TCAAGCCGTATGGCACCCAGAGGACC | | 26 | SGR | 8 | 100 | 26 | 3.34E-08 | 100 | | 13 | | 1.8 | 94 | 18 | | 0.43 | | | | 100 | | 17 | | | 0.54 | | |  |  |  |  |  |  |  |  |
| **miR50** | TGAACTGGGCGAGGTTCCAGCGGACG | | 26 | SGR | 8 | 100 | 26 | 3.34E-08 | 91 | | 22 | | 0.45 | 96 | 26 | | 7.30E-06 | | | | 100 | | 18 | | | 0.14 | | |  |  |  |  |  |  |  |  |
| **miR51** | AGTAAACATCGAATTCACGTCAGCGTT | | 27 | SGR | 8 | 100 | 27 | 9.15E-09 | 100 | | 13 | | 1.9 | 100 | 27 | | 8.21E-09 | | | | 95 | | 20 | | | 2.1 | | |  |  |  |  |  |  |  |  |
| **miR52** | CGAAGACGACCTTCTGCATCGGCTCCC | | 27 | SGR | 8 | 100 | 27 | 9.15E-09 | 100 | | 14 | | 0.49 | 93 | 27 | | 4.88E-04 | | | | 100 | | 17 | | | 0.54 | | |  |  |  |  |  |  |  |  |
| **miR53** | TGCGAGCTTGCGGACGTTCTCCTGCG | | 26 | SGR | 8 | 100 | 26 | 3.34E-08 | 100 | | 16 | | 0.029 | 100 | 16 | | 0.028 | | | |  | |  | | |  | | |  |  |  |  |  |  |  |  |
| **miR54** | GCTGGAGAGTGCCCTTCAATCGCTGG | | 26 |  | 8 | 100 | 26 | 3.34E-08 | 100 | | 15 | | 0.11 | 92 | 26 | | 0.002 | | | | 100 | | 18 | | | 0.14 | | |  |  |  |  |  |  |  |  |
| **miR55** | AACTATGATGAGGTTAGCGATCCCAAGC | | 28 |  | 8 | 100 | 28 | 2.49E-09 | 96 | | 25 | | 3.51E-05 | 100 | 25 | | 1.38E-07 | | | | 100 | | 17 | | | 0.54 | | |  |  |  |  |  |  |  |  |
| **miR56** | GATGACAAGGACAAGTATGAGACGTTC | | 27 |  | 8 | 100 | 27 | 9.15E-09 | 95 | | 20 | | 0.031 | 100 | 23 | | 2.00E-06 | | | | 100 | | 19 | | | 0.035 | | |  |  |  |  |  |  |  |  |
| **miR57** | AACGTTGCTGATCCAGAGGTTCCTCTC | | 27 |  | 8 | 100 | 27 | 9.15E-09 | 92 | | 26 | | 0.002 | 100 | 24 | | 5.07E-07 | | | | 95.238 | | 21 | | | 0.54 | | |  |  |  |  |  |  |  |  |
| **miR58** | TTAGACTGCTGAGACAGTGTTATATGATT | | 29 |  | 8 | 100 | 29 | 6.76E-10 | 97 | | 29 | | 1.54E-07 | 97 | 29 | | 1.48E-07 | | | | 100 | | 18 | | | 0.14 | | |  |  |  |  |  |  |  |  |
| **miR59** | CTATGTTGAGAACCACCCAAACAA | | 24 |  | 8 | 100 | 24 | 4.34E-07 | 100 | | 13 | | 1.6 | 100 | 14 | | 0.4 | | | | 100 | | 18 | | | 0.14 | | |  |  |  |  |  |  |  |  |
| **miR60** | GTCTTCCGAACACACCTGCGATAAAC | | 26 |  | 8 | 100 | 26 | 3.34E-08 | 100 | | 13 | | 1.8 | 95 | 19 | | 0.11 | | | |  | |  | | |  | | |  |  |  |  |  |  |  |  |
| **miR61** | GGCGGAATGTTCGGCGGCGACTCAT | | 25 |  | 8 | 100 | 25 | 1.21E-07 | 95 | | 22 | | 0.002 | 96 | 23 | | 4.13E-04 | | | |  | |  | | |  | | |  |  |  |  |  |  |  |  |
| **miR62** | GGTGGAATGTTCGGCGGCGACTCGT | | 25 |  | 8 | 100 | 25 | 1.21E-07 | 96 | | 25 | | 2.76E-05 | 95 | 22 | | 0.002 | | | |  | |  | | |  | | |  |  |  |  |  |  |  |  |
| **miR63** | GAGAAGATCCGCGAGGGCCAGAAGG | | 25 |  | 8 | 100 | 25 | 1.21E-07 | 92 | | 25 | | 0.007 | 92 | 25 | | 0.006 | | | | 100 | | 20 | | | 0.009 | | |  |  |  |  |  |  |  |  |
| **miR64** | GCCAAGGAGGACGAGAAGATCCGCGAGG | | 28 |  | 8 | 100 | 28 | 2.49E-09 | 89 | | 28 | | 0.034 | 96 | 25 | | 3.37E-05 | | | | 100 | | 17 | | | 0.54 | | |  |  |  |  |  |  |  |  |
| **miR65** | CTTCAAGGGCGGGAACGACGAGAGGT | | 26 |  | 8 | 100 | 26 | 3.34E-08 | 96 | | 26 | | 7.61E-06 | 100 | 26 | | 3.00E-08 | | | | 100 | | 16 | | | 2.1 | | |  |  |  |  |  |  |  |  |
| **miR66** | CCCGGGTAGGCACGGTCAAAGAGT | | 24 |  | 8 | 100 | 24 | 4.34E-07 | 100 | | 14 | | 0.41 | 100 | 15 | | 0.1 | | | | 100 | | 16 | | | 2.1 | | |  |  |  |  |  |  |  |  |
| **miR67** | AGAAGATAGAGAAAGAGCTTTCGGAC | | 26 |  | 8 | 100 | 26 | 3.34E-08 | 100 | | 15 | | 0.11 | 96 | 26 | | 7.30E-06 | | | | 100 | | 19 | | | 0.035 | | |  |  |  |  |  |  |  |  |
| **miR68** | TGCTAGTCACCGTCCCTCTGTGGCGTC | | 27 |  | 8 | 100 | 27 | 9.15E-09 | 100 | | 13 | | 1.9 | 100 | 13 | | 1.9 | | | | 100 | | 16 | | | 2.1 | | |  |  |  |  |  |  |  |  |
| **miR69** | TGAGGTCCATGAAGGCCGTCGCCA | | 24 |  | 8 | 100 | 24 | 4.34E-07 | 100 | | 14 | | 0.41 | 100 | 13 | | 1.6 | | | | 95 | | 20 | | | 2.1 | | |  |  |  |  |  |  |  |  |
| **miR70** | TGAGGGAGCTGAGGTCCATGAAGGC | | 25 |  | 8 | 100 | 25 | 1.21E-07 | 100 | | 13 | | 1.6 | 91 | 22 | | 0.4 | | | | 100 | | 18 | | | 0.14 | | |  |  |  |  |  |  |  |  |
| **miR71** | TGGCAGGCCGTGCAGGACGAGGCGT | | 25 |  | 8 | 100 | 25 | 1.21E-07 | 100 | | 14 | | 0.41 | 100 | 17 | | 0.006 | | | | 100 | | 17 | | | 0.54 | | |  |  |  |  |  |  |  |  |
| **miR72** | CGCAAAGGAGTGCAAGAAGTGTGCAGAA | | 28 |  | 8 | 100 | 28 | 2.49E-09 | 100 | | 15 | | 0.13 | 100 | 17 | | 0.008 | | | | 100 | | 18 | | | 0.14 | | |  |  |  |  |  |  |  |  |
| **miR73** | CGTTAAAGAGGCTCAGGACTGGCTCC | | 26 |  | 8 | 100 | 26 | 3.34E-08 | 100 | | 13 | | 1.8 | 100 | 16 | | 0.028 | | | | 100 | | 18 | | | 0.14 | | |  |  |  |  |  |  |  |  |
| **miR74** | GCCAATAAATGCGCGTGTGATCGAAGT | | 27 |  | 8 | 100 | 27 | 9.15E-09 | 100 | | 13 | | 1.9 | 96 | 27 | | 2.00E-06 | | | | 100 | | 16 | | | 2.1 | | |  |  |  |  |  |  |  |  |
| **miR75** | CTCACGGAAGAAGAGGCGCTGCTCAGG | | 27 |  | 8 | 100 | 27 | 9.15E-09 | 100 | | 15 | | 0.12 | 96 | 26 | | 7.91E-06 | | | | 100 | | 17 | | | 0.54 | | |  |  |  |  |  |  |  |  |
| **miR76** | CGAGAACGGAAAGCTCTGGATGCTTCA | | 27 |  | 8 | 100 | 27 | 9.15E-09 | 100 | | 17 | | 0.008 | 96 | 24 | | 1.24E-04 | | | | 100 | | 18 | | | 0.14 | | |  |  |  |  |  |  |  |  |
| **miR77** | GTCAACAAGAGCGGCCTCAGCACAGGT | | 27 |  | 8 | 100 | 27 | 9.15E-09 | 100 | | 22 | | 8.25E-06 | 100 | 26 | | 3.25E-08 | | | | 100 | | 17 | | | 0.54 | | |  |  |  |  |  |  |  |  |
| **miR78** | GGGTGATTAGCAGTCATACAGTCC | | 24 |  | 8 | 100 | 24 | 4.34E-07 | 92 | | 24 | | 0.027 | 95 | 20 | | 0.025 | | | | 95.238 | | 21 | | | 0.54 | | |  |  |  |  |  |  |  |  |
| **miR79** | GGCTAGAGCGCGACTGGTTGAGTTCCC | | 27 |  | 8 | 100 | 27 | 9.15E-09 | 96 | | 26 | | 8.25E-06 | 93 | 27 | | 4.88E-04 | | | |  | |  | | |  | | |  |  |  |  |  |  |  |  |
| **miR80** | CCTTGTCCCGGCTGGCGCCGTCCACCTT | | 28 |  | 8 | 100 | 28 | 2.49E-09 | 96 | | 28 | | 5.69E-07 | 100 | 28 | | 2.24E-09 | | | |  | |  | | |  | | |  |  |  |  |  |  |  |  |
| **miR81** | GTCCACCTCTGGTTCGGCACACATT | | 25 |  | 8 | 100 | 25 | 1.21E-07 | 100 | | 14 | | 0.41 | 100 | 13 | | 1.6 | | | |  | |  | | |  | | |  |  |  |  |  |  |  |  |
| **miR82** | GAAGAGTGTCAAGGAGAAGACGGAGAT | | 27 |  | 8 | 100 | 27 | 9.15E-09 | 100 | | 17 | | 0.008 | 100 | 14 | | 0.47 | | | | 100 | | 19 | | | 0.035 | | |  |  |  |  |  |  |  |  |
| **miR83** | GGCAGATGATGACTTTGCGACGGGCG | | 26 |  | 8 | 100 | 26 | 3.34E-08 | 100 | | 24 | | 4.88E-07 | 100 | 26 | | 3.00E-08 | | | | 100 | | 18 | | | 0.14 | | |  |  |  |  |  |  |  |  |
| **miR84** | CCAAGGAGATGATGAGGGAGATGGCCCA | | 28 |  | 8 | 100 | 28 | 2.49E-09 | 96 | | 23 | | 5.48E-04 | 100 | 28 | | 2.24E-09 | | | | 100 | | 20 | | | 0.009 | | |  |  |  |  |  |  |  |  |
| **miR85** | GTACGAGAAGCGCGCGACGGAGATG | | 25 |  | 8 | 100 | 25 | 1.21E-07 | 100 | | 25 | | 1.13E-07 | 100 | 25 | | 1.08E-07 | | | |  | |  | | |  | | |  |  |  |  |  |  |  |  |
| **miR86** | GCTGAGGAGGTCGACCAGAAGCTCCGCG | | 28 |  | 8 | 100 | 28 | 2.49E-09 | 96 | | 25 | | 3.51E-05 | 100 | 25 | | 1.38E-07 | | | |  | |  | | |  | | |  |  |  |  |  |  |  |  |
| **miR87** | GAAGGACGCAACGTACGATGAGATCTGC | | 28 |  | 8 | 100 | 28 | 2.49E-09 | 89 | | 28 | | 0.034 | 89 | 27 | | 0.13 | | | |  | |  | | |  | | |  |  |  |  |  |  |  |  |
| **miR88** | TGCAGAGCCCCGCCGCCTCAGTGATC | | 26 |  | 8 | 100 | 26 | 3.34E-08 | 100 | | 14 | | 0.45 | 94 | 18 | | 0.43 | | | | 95 | | 20 | | | 2.1 | | |  |  |  |  |  |  |  |  |
| **miR89** | CTGTGATGACGGCTATGACGGAGAC | | 25 |  | 8 | 100 | 25 | 1.21E-07 | 92 | | 25 | | 0.007 | 100 | 25 | | 1.08E-07 | | | | 100 | | 16 | | | 2.1 | | |  |  |  |  |  |  |  |  |
| **miR90** | GCCTTGCCCGACTGAGAGTGCTCGCT | | 26 |  | 8 | 100 | 26 | 3.34E-08 | 91 | | 23 | | 0.11 | 100 | 23 | | 1.85E-06 | | | |  | |  | | |  | | |  |  |  |  |  |  |  |  |
| **miR91** | ATGATTCCTCTGTCCATTCCCCTGATC | | 27 |  | 8 | 100 | 27 | 9.15E-09 | 100 | | 14 | | 0.49 | 100 | 25 | | 1.28E-07 | | | | 100 | | 20 | | | 0.009 | | |  |  |  |  |  |  |  |  |
| **miR92** | CGCGTCGTGCAGGCCTTCAAGGATCC | | 26 | SGR | 8 | 100 | 26 | 3.34E-08 | 96 | | 23 | | 4.70E-04 | 100 | 26 | | 3.00E-08 | | | | 100 | | 17 | | | 0.54 | | |  |  |  |  |  |  |  |  |
| **miR93** | TACGACAGCGGCGTTTACATCA | | 22 |  | 8 | 100 | 22 | 6.10E-06 | 95 | | 20 | | 0.022 | 100 | 14 | | 0.33 | | | |  | |  | | |  | | |  |  |  |  |  |  |  |  |
| **miR94** | AGCCGATGCAGAAGGTCGTCTTCG | | 24 |  | 8 | 100 | 24 | 4.34E-07 | 100 | | 14 | | 0.41 | 92 | 24 | | 0.025 | | | | 100 | | 17 | | | 0.54 | | |  |  |  |  |  |  |  |  |
| **miR95** | TTGAAGATGCCCTTCGGCGTATT | | 23 |  | 8 | 100 | 23 | 1.72E-06 | 100 | | 13 | | 1.5 | 100 | 14 | | 0.36 | | | |  | |  | | |  | | |  |  |  |  |  |  |  |  |
| **miR96** | ATACTCTGACGCAAAGCTAAGCTGA | | 25 | SGR | 8 | 100 | 25 | 1.21E-07 | 95 | | 19 | | 0.1 | 100 | 14 | | 0.4 | | | |  | |  | | |  | | |  |  |  |  |  |  |  |  |
| **miR97** | ACGCCTTCTGCACCAAGGAGCTGA | | 24 |  | 8 | 100 | 24 | 4.34E-07 | 100 | | 14 | | 0.41 | 100 | 15 | | 0.1 | | | | 100 | | 19 | | | 0.035 | | |  |  |  |  |  |  |  |  |
| **miR98** | ACCTCTTCTGCGGGCTCTACGACAA | | 25 |  | 8 | 100 | 25 | 1.21E-07 | 96 | | 23 | | 4.31E-04 | 100 | 15 | | 0.1 | | | | 100 | | 16 | | | 2.1 | | |  |  |  |  |  |  |  |  |
| **miR99** | CTTGTAGATCCTCTTCGCGTGGTA | | 24 | SGR | 8 | 100 | 24 | 4.34E-07 | 95 | | 19 | | 0.1 | 96 | 24 | | 1.05E-04 | | | |  | |  | | |  | | |  |  |  |  |  |  |  |  |
| **miR100** | GGCGAGCAAGATCATCGCGTTCACGA | | 26 |  | 8 | 100 | 26 | 3.34E-08 | 100 | | 25 | | 1.23E-07 | 96 | 26 | | 7.30E-06 | | | | 100 | | 16 | | | 2.1 | | |  |  |  |  |  |  |  |  |
| **miR101** | GCTCCTCTTCGTACGCCAGTGCGTGA | | 26 |  | 8 | 100 | 26 | 3.34E-08 | 96 | | 26 | | 7.61E-06 | 96 | 26 | | 7.30E-06 | | | |  | |  | | |  | | |  |  |  |  |  |  |  |  |
| **miR102** | TTCGAAGCGCGGCTCAAGTTCA | | 22 |  | 8 | 100 | 22 | 6.10E-06 | 100 | | 13 | | 1.3 | 95 | 22 | | 0.001 | | | | 100 | | 16 | | | 2.1 | | |  |  |  |  |  |  |  |  |
| **miR103** | GGACGTACTGGGCGAAGGACTACAACA | | 27 | SGR | 8 | 100 | 27 | 9.15E-09 | 100 | | 15 | | 0.12 | 100 | 27 | | 8.21E-09 | | | | 100 | | 17 | | | 0.54 | | |  |  |  |  |  |  |  |  |
| **miR104** | GCTCGAGCGGGAGCTCCTCGACAACT | | 26 | SGR | 8 | 100 | 26 | 3.34E-08 | 96 | | 25 | | 3.01E-05 | 96 | 25 | | 2.89E-05 | | | | 100 | | 16 | | | 2.1 | | |  |  |  |  |  |  |  |  |
| **miR105** | GACTTCAAGCGCCGCGTCATCT | | 22 |  | 8 | 100 | 22 | 6.10E-06 | 100 | | 15 | | 0.086 | 100 | 13 | | 1.3 | | | |  | |  | | |  | | |  |  |  |  |  |  |  |  |
| **miR106** | CTGCGCTGGACGATGAACTGGAGAT | | 25 |  | 8 | 100 | 25 | 1.21E-07 | 100 | | 15 | | 0.1 | 92 | 25 | | 0.006 | | | | 100 | | 16 | | | 2.1 | | |  |  |  |  |  |  |  |  |
| **miR107** | ACCGCTCAGAGTAACTATGAGTGCTTC | | 27 |  | 8 | 100 | 27 | 9.15E-09 | 100 | | 20 | | 1.29E-04 | 100 | 20 | | 1.24E-04 | | | | 100 | | 17 | | | 0.54 | | |  |  |  |  |  |  |  |  |
| **miR108** | TAGGTGGCCAGGACGAGCTGCA | | 22 |  | 8 | 100 | 22 | 6.10E-06 | 100 | | 14 | | 0.34 | 100 | 14 | | 0.33 | | | | 100 | | 17 | | | 0.54 | | |  |  |  |  |  |  |  |  |
| **miR109** | CCTGTGATGCCGTGGGTTGCGTCGCT | | 26 |  | 8 | 100 | 26 | 3.34E-08 | 100 | | 13 | | 1.8 | 100 | 14 | | 0.43 | | | | 100 | | 17 | | | 0.54 | | |  |  |  |  |  |  |  |  |
| **miR110** | GCAATGGCCACCCAGTGCGTGTCTCGT | | 27 |  | 8 | 100 | 27 | 9.15E-09 | 92 | | 24 | | 0.031 | 100 | 18 | | 0.002 | | | | 100 | | 17 | | | 0.54 | | |  |  |  |  |  |  |  |  |
| **miR111** | ACCCAGGGACAGAGCAGCGAGTCA | | 24 |  | 8 | 100 | 24 | 4.34E-07 | 100 | | 13 | | 1.6 | 100 | 14 | | 0.4 | | | | 100 | | 17 | | | 0.54 | | |  |  |  |  |  |  |  |  |
|  | |  | | | | | | | |  | |  | | | |  | | |  |  | |  | |  | | |  | | |  |  |  |  |  |  |  |

References

1. Huang PJ, et al. 2012 Identification of putative miRNAs from the deep-branching unicellular flagellates. Genomics 992:101-107.

2. Zhang YQ, Chen DL, Tian HF, Zhang BH, Wen JF 2009 Genome-wide computational identification of microRNAs and their targets in the deep-branching eukaryote Giardia lamblia. Comput Biol Chem 335:391-396.

3. Chen XS, Collins LJ, Biggs PJ, Penny D 2009 High throughput genome-wide survey of small RNAs from the parasitic protists Giardia intestinalis and Trichomonas vaginalis. Genome Biol Evol 1:165-175.

4. Saraiya AA, Wang CC 2008 snoRNA, a novel precursor of microRNA in Giardia lamblia. PLoS Pathog 411:e1000224.

5. Saraiya AA, Li W, Wang CC 2011 A microRNA derived from an apparent canonical biogenesis pathway regulates variant surface protein gene expression in Giardia lamblia. RNA 1712:2152-2164.

6. Li W, Saraiya AA, Wang CC 2011 Gene regulation in Giardia lambia involves a putative microRNA derived from a small nucleolar RNA. PLoS Negl Trop Dis 510:e1338.

7. Li W, Saraiya AA, Wang CC 2012 The profile of snoRNA-derived microRNAs that regulate expression of variant surface proteins in Giardia lamblia. Cell Microbiol 149:1455-1473.

8. Saraiya AA, Li W, Wu J, Chang CH, Wang CC 2014 The microRNAs in an Ancient Protist Repress the Variant-Specific Surface Protein Expression by Targeting the Entire Coding Sequence. PLoS Pathog 102:e1003791.
