# Supplementary material for: Giardia lamblia miRNAs as a new diagnostic tool for human giardiasis
Source: PLoS Negl Trop Dis. 2019 Jun 17;13(6):e0007398. doi: 10.1371/journal.pntd.0007398 (PMC6597124; doi:10.1371/journal.pntd.0007398)
Supplement: S2 Table — Calculation of the optimal cutoff as determined as the cutoff with the highest likelihood ratio [defined as %sensitivity / (100-%specificity)]. (DOCX) [file pntd.0007398.s005.docx]

**S2 Table:** ***Giardia* miR5 in human duodenal biopsies**

| **Ct of infected duodenum biopsies** | **Ct of infected** **gastric biopsies** |  |  | **Area under the ROC curve** | |
| --- | --- | --- | --- | --- | --- |
| 22.72 | 35.685 |  |  | Area | 1.0 |
| 21.15 | 39.000 |  |  | Std. Error | 0.0 |
| 32.92 | 37.000 |  |  | 95% CI | 1.0 to 1.0 |
| 21.14 | 36.000 |  |  | P value | 0.0002151 |
| 23.35 | 34.000 |  |  | **Data** |  |
| 21.35 | 34.000 |  |  | Control | 12 |
| 25.69 | 36.000 |  |  | Patient | 8 |
| 21.51 | 39.000 |  |  |  |  |
| 22.72 | 41.000 |  |  |  |  |
|  | 41.000 |  |  |  |  |
|  | 41.000 |  |  |  |  |
|  | 41.000 |  |  |  |  |
|  |  |  |  |  |  |
|  |  |  |  |  |  |
| **Cut-off** | **Sensitivity%** | **95% CI** | **Specificity%** | **95% CI** | **Likelihood ratio** |
| < 21.15 | 12.50 | 0.3160% to 52.65% | 100.0 | 73.54% to 100.0% |  |
| < 21.25 | 25.00 | 3.185% to 65.09% | 100.0 | 73.54% to 100.0% |  |
| < 21.43 | 37.50 | 8.523% to 75.51% | 100.0 | 73.54% to 100.0% |  |
| < 22.12 | 50.00 | 15.70% to 84.30% | 100.0 | 73.54% to 100.0% |  |
| < 23.04 | 62.50 | 24.49% to 91.48% | 100.0 | 73.54% to 100.0% |  |
| < 24.52 | 75.00 | 34.91% to 96.81% | 100.0 | 73.54% to 100.0% |  |
| < 29.31 | 87.50 | 47.35% to 99.68% | 100.0 | 73.54% to 100.0% |  |
| < 33.46 | 100.0 | 63.06% to 100.0% | 100.0 | 73.54% to 100.0% |  |
| < 34.84 | 100.0 | 63.06% to 100.0% | 83.33 | 51.59% to 97.91% | 6.00 |
| < 35.84 | 100.0 | 63.06% to 100.0% | 75.00 | 42.81% to 94.51% | 4.00 |
| < 36.50 | 100.0 | 63.06% to 100.0% | 58.33 | 27.67% to 84.83% | 2.40 |
| < 38.00 | 100.0 | 63.06% to 100.0% | 50.00 | 21.09% to 78.91% | 2.00 |
| < 40.00 | 100.0 | 63.06% to 100.0% | 33.33 | 9.925% to 65.11% | 1.50 |
|  |  |  |  |  |  |
