# Supplementary material for: Giardia lamblia miRNAs as a new diagnostic tool for human giardiasis
Source: PLoS Negl Trop Dis. 2019 Jun 17;13(6):e0007398. doi: 10.1371/journal.pntd.0007398 (PMC6597124; doi:10.1371/journal.pntd.0007398)
Supplement: S3 Table — Calculation of the optimal cutoff as determined as the cutoff with the highest likelihood ratio [defined as %sensitivity / (100-%specificity)]. (DOCX) [file pntd.0007398.s006.docx]

**S3 Table: *Giardia* miR6 in human stool samples**

| **CT healthy** | **Ct infected Patients** |  |  | **Area under the ROC curve** | |
| --- | --- | --- | --- | --- | --- |
| 33.877830 | 28.636000 |  |  | Area | 0.8889 |
| 32.504670 | 28.948000 |  |  | Std. Error | 0.07488 |
| 32.988000 | 29.622750 |  |  | 95% CI | 0.7421 to 1.00 |
| 35.459000 | 28.886500 |  |  | P value | 0.004284 |
| 35.766250 | 28.927500 |  |  | **Data** |  |
| 36.598000 | 31.572500 |  |  | Control | 10 |
| 29.284620 | 31.063500 |  |  | Patient | 9 |
| 32.042500 | 28.927500 |  |  |  |  |
| 30.394120 | 31.270000 |  |  |  |  |
| 30.708120 |  |  |  |  |  |
|  |  |  |  |  |  |
|  |  |  |  |  |  |
|  |  |  |  |  |  |
| **Cut-off** | **Sensitivity%** | **95% CI** | **Specificity%** | **95% CI** | **Likelihood ratio** |
| < 28.76 | 11.11 | 0.2809% to 48.25% | 100.0 | 69.15% to 100.0% |  |
| < 28.91 | 22.22 | 2.815% to 60.01% | 100.0 | 69.15% to 100.0% |  |
| < 28.94 | 44.44 | 13.70% to 78.80% | 100.0 | 69.15% to 100.0% |  |
| < 29.12 | 55.56 | 21.20% to 86.30% | 100.0 | 69.15% to 100.0% |  |
| < 29.45 | 55.56 | 21.20% to 86.30% | 90.00 | 55.50% to 99.75% | 5.56 |
| < 30.01 | 66.67 | 29.93% to 92.51% | 90.00 | 55.50% to 99.75% | 6.67 |
| < 30.55 | 66.67 | 29.93% to 92.51% | 80.00 | 44.39% to 97.48% | 3.33 |
| < 30.89 | 66.67 | 29.93% to 92.51% | 70.00 | 34.75% to 93.33% | 2.22 |
| < 31.17 | 77.78 | 39.99% to 97.19% | 70.00 | 34.75% to 93.33% | 2.59 |
| < 31.42 | 88.89 | 51.75% to 99.72% | 70.00 | 34.75% to 93.33% | 2.96 |
| < 31.81 | 100.0 | 66.37% to 100.0% | 70.00 | 34.75% to 93.33% | 3.33 |
| < 32.27 | 100.0 | 66.37% to 100.0% | 60.00 | 26.24% to 87.84% | 2.50 |
| < 32.75 | 100.0 | 66.37% to 100.0% | 50.00 | 18.71% to 81.29% | 2.00 |
| < 33.43 | 100.0 | 66.37% to 100.0% | 40.00 | 12.16% to 73.76% | 1.67 |
| < 34.67 | 100.0 | 66.37% to 100.0% | 30.00 | 6.674% to 65.25% | 1.43 |
| < 35.61 | 100.0 | 66.37% to 100.0% | 20.00 | 2.521% to 55.61% | 1.25 |
| < 36.18 | 100.0 | 66.37% to 100.0% | 10.00 | 0.2529% to 44.50% | 1.11 |
|  |  |  |  |  |  |
