# Supplementary material for: Giardia lamblia miRNAs as a new diagnostic tool for human giardiasis
Source: PLoS Negl Trop Dis. 2019 Jun 17;13(6):e0007398. doi: 10.1371/journal.pntd.0007398 (PMC6597124; doi:10.1371/journal.pntd.0007398)
Supplement: S1 Fig — All are lowly expressed, and thus unlikely to exert significant regulation. (PDF) [file pntd.0007398.s007.pdf]

```
Provisional ID   : GLCHR05_3794
Score total      :       95.3
Score for star read(s)    3.9
Score for read counts    92.9
Score for mfe    :        0.7
Score for randfold       -2.2
Score for cons. seed
Total read count :        194
Mature read count:        145
Loop read count  :          0
Star read count  :         49
```

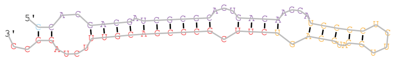

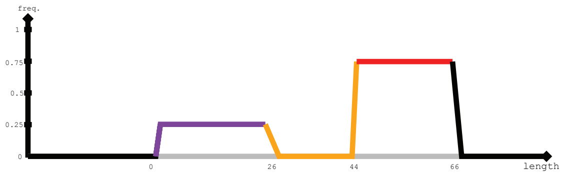


```
                          Star                                     Mature
5'- acuagcagauuccuuucgucgucggaccacgacgaucgcgcagugagaagcauccgcucuugcauggagucuucgcgcgacguuucuaggccgguuuaugacaacuuugaga  -3'  obs
    acuagcagauuccuuucgucgucggaccacgacgaucgcgcagugagaagcauccgcucuugcauggagucuucgcgcgacguuucuaggccgguuuaugacaacuuugaga       exp
    ................(((((((.((..((((.((((((...((((....(((((....))..))).)))).))))))))))....)))))....))))).........   reads  mm  sample
    ......................................................................cuucgcgcgacguuucuaggcc....................   120    0   seq
    ...........................cacgacgaucgcgcagugagaagca............................................................   43     0   seq
    ......................................................................cGucgcgcgacguuucuaggcc....................   5      1   seq
    ......................................................................Guucgcgcgacguuucuaggcc....................   5      1   seq
    ......................................................................cuucgcgcgacguuucAaggcc....................   2      1   seq
    ......................................................................cuucgcgcgacguCucuaggcc....................   2      1   seq
    ......................................................................cuucgcgcgacguuucuaggcA....................   1      1   seq
    ......................................................................cuCcgcgcgacguuucuaggcc....................   1      1   seq
    ......................................................................cuucgcgcgGcguuucuaggcc....................   1      1   seq
    ......................................................................cuucgcgcgUcguuucuaggcc....................   1      1   seq
    ......................................................................cuucgcgcgacguuuUuaggcc....................   1      1   seq
    ......................................................................cuucgUgcgacguuucuaggcc....................   1      1   seq
    ......................................................................cuucgcgcgacAuuucuaggcc....................   1      1   seq
    ......................................................................cuucgcgcAacguuucuaggcc....................   1      1   seq
    ......................................................................cuucgcgcgaUguuucuaggcc....................   1      1   seq
    ......................................................................cuucgcgcgacguuucuaggUc....................   1      1   seq
    ......................................................................cuucgcgcgacguuucuaggAc....................   1      1   seq
    ...........................cacgacgaucgcgcGgugagaagca............................................................   1      1   seq
    ...........................Gacgacgaucgcgcagugagaagca............................................................   1      1   seq
    ...........................cacgacgaucgcgcagugGgaagca............................................................   1      1   seq
    ...........................cUcgacgaucgcgcagugagaagca............................................................   1      1   seq
    ...........................cacgacgaucgcgcagugCgaagca............................................................   1      1   seq
    ...........................cacgacgaucgcgcCgugagaagca............................................................   1      1   seq
```

```
Provisional ID   : GLCHR03_2963
Score total      :      179.7
Score for star read(s)    3.9
Score for read counts   178.6
Score for mfe    :       -0.5
Score for randfold       -2.2
Score for cons. seed
Total read count :        362
Mature read count:        239
Loop read count  :         23
Star read count  :        100
```

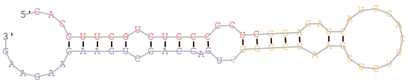

```
                        Mature                                      Star
5'- uucuucuacccgcucgacuucaccuucgucugccccucggagaucauccacuucucgaaucuggcugagcagcugaagaagaagugcaacgcggagaucaucaucggc  -3'  obs
    uucuucuacccgcucgacuucaccuucgucugccccucggagaucauccacuucucgaaucuggcugagcagcugaagaagaagugcaacgcggagaucaucaucggc       exp
    ....(((..((((..((((((..(((((.(((((..(((((..((..........)).)))))..).)))).))))).)))))..))))).)...)))))))...........

Gi3_23566110_x197.................caccuucgucugccccuc...................................................................... 0
Gi5_7475040_x84................................................................ugagcagcugaagaagaag........................ 0
G1a_5548289_x15.....................................ggagaucauccacuucucgaaucuggcu.......................................... 0
Gi5_8482001_x8.................................................................ugUgcagcugaagaagaag........................ 1
Gi3_27535030_x6...................Gaccuucgucugccccuc...................................................................... 1
Gi3_27516292_x6...................caccuucgucugccccCc...................................................................... 1
Gi3_27684541_x4...................cGccuucgucugccccuc...................................................................... 1
Gi3_27757425_x4...................caccuucgNcugccccuc...................................................................... 1
Gi5_8608051_x4.................................................................uUagcagcugaagaagaag........................ 1
G1a_5831167_x3......................................Ugagaucauccacuucucgaaucuggcu.......................................... 1
Gi3_28203340_x2...................caccuCcgucugccccuc...................................................................... 1
Gi3_27995022_x2...................Uaccuucgucugccccuc...................................................................... 1
G1a_5948646_x2......................................gUagaucauccacuucucgaaucuggcu.......................................... 1
Gi3_28013188_x2...................caUcuucgucugccccuc...................................................................... 1
G1a_6331257_x1......................................ggagaucauccGcuucucgaaucuggcu.......................................... 1
Gi3_28312174_x1...................cCccuucgucugccccuc...................................................................... 1
Gi3_28816657_x1...................caccuucgucugccccGc...................................................................... 1
Gi5_8832335_x1.................................................................uAagcagcugaagaagaag........................ 1
Gi5_8917844_x1.................................................................ugagcagcugaagaagaGg........................ 1
Gi3_28874001_x1...................caccuucgCcugccccuc...................................................................... 1
Gi5_9029076_x1.................................................................ugagcagcugaCgaagaag........................ 1
Gi3_28405665_x1...................caccuucgucugGcccuc...................................................................... 1
Gi3_28450358_x1...................caccuucgucugccAcuc...................................................................... 1
Gi3_28482085_x1...................caccuucgucCgccccuc...................................................................... 1
Gi3_28821535_x1...................Aaccuucgucugccccuc...................................................................... 1
G1a_6308435_x1......................................ggagaucauccUcuucucgaaucuggcu.......................................... 1
Gi3_28667735_x1...................cUccuucgucugccccuc...................................................................... 1
Gi3_28576974_x1...................caccuucgucAgccccuc...................................................................... 1
Gi3_28937042_x1...................caccAucgucugccccuc...................................................................... 1
Gi3_28453551_x1...................caccGucgucugccccuc...................................................................... 1
Gi3_28400470_x1...................caccuucgucugcccAuc...................................................................... 1
G1a_5992616_x1......................................ggagaucaNccacuucucgaaucuggcu.......................................... 1
Gi3_28702504_x1...................cacUuucgucugccccuc...................................................................... 1
Gi3_28502675_x1...................cacAuucgucugccccuc...................................................................... 1
Gi5_8899174_x1.................................................................ugagcagcugCagaagaag........................ 1
Gi3_28551526_x1...................caccCucgucugccccuc...................................................................... 1
Gi3_28822757_x1...................caccuucgucugccccuA...................................................................... 1
```

```
Provisional ID   : GLCHR04_3925
Score total      :       35.6
Score for star read(s)    3.9
Score for read counts    32.2
Score for mfe    :        1.6
Score for randfold       -2.2
Score for cons. seed
Total read count :         75
Mature read count:         68
Loop read count  :          0
Star read count  :          7
```

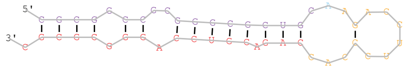

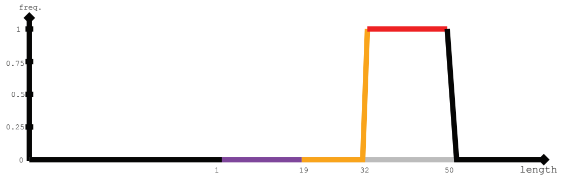


```
                                          Star                          Mature
5'- aagggcaaccucggcuaccucccccgcaagcgcugcacgcgcggccgcgggcgcugcaagaccuucccaccagacguccagggcgcgcccauccaccucacggccuuc  -3'  obs
    aagggcaaccucggcuaccucccccgcaagcgcugcacgcgcggccgcgggcgcugcaagaccuucccaccagacguccagggcgcgcccauccaccucacggccuuc       exp
    .(((....))).((((.........(((.....)))..(((((.((..((((((((...(......)...))).))))).)).)))))............))))...

G1a_5174667_x61......................................................................cagacguccagggcgcgc....................
Gi3_27565942_x5.......................................cgcggccgcgggcgcCgc...................................................
Gi3_27989138_x2.......................................cCcggccgcgggcgcugc...................................................
G1a_5897308_x2.......................................................................cUgacguccagggcgcgc....................
G1a_6021477_x1.......................................................................cagacguUcagggcgcgc....................
G1a_6010028_x1.......................................................................cagacguccGgggcgcgc....................
Gi1_4376926_x1.......................................................................cagacguccagggcgcgc....................
G1a_6108736_x1.......................................................................cagacguccagggcgUgc....................
Gi4_598799_x1 .......................................................................cagacgucUagggcgcgc....................
```

```
Provisional ID   : GLCHR04_4478
Score total      :       44.7
Score for star read(s)    3.9
Score for read counts    40.4
Score for mfe    :        2.6
Score for randfold       -2.2
Score for cons. seed
Total read count :         91
Mature read count:         89
Loop read count  :          0
Star read count  :          2
```

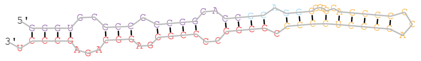

```
                             Star                                             Mature
5'- a cucgcggccucgcuguacgcggggugcgccgccggcaccgcagggcccgcagcccccagggcuccccgcggccccgggaggcagagcccuccggcgcgcaggccgucgag  -3'  obs
    agcucgcggccucgcuguacgcgggugcgccgccggcaccgcagggcccgcagcccccagggcuccccgcggccccgggaggcagagcccuccggcgcgcaggccgucgag       exp
    ..((((((((((((.((.((.(((((..(((.((((..((((.(((...(.(((((...)))))))))).))))..))))..)))...))))).))))))).)))))).))))

Gi5_7635768_x70....................................................................cgcggccccgggaggcagagcccu.................... 0
Gi5_8549593_x6.....................................................................cgcggccccgggaggcagaAcccu.................... 1
Gi5_8753515_x2.....................................................................cgcggccccgggaggcagCgcccu.................... 1
Gi5_8829463_x2.....................................................................cUcggccccgggaggcagagcccu.................... 1
Gi5_8981940_x1.....................................................................cCcggccccgggaggcagagcccu.................... 1
Gi5_9085006_x1.....................................................................cgcggccccgggGggcagagcccu.................... 1
Gi5_9060210_x1.....................................................................cgcggccccgggaggcUgagcccu.................... 1
Gi5_9006297_x1.....................................................................cgcggcccNgggaggcagagcccu.................... 1
Gi5_9032993_x1.....................................................................cgcggccccggUaggcagagcccu.................... 1
Gi3_28676375_x1......................gggugcgccgccggGacc........................................................................ 1
Gi5_9083292_x1.....................................................................cgcgAccccgggaggcagagcccu.................... 1
Gi2_11335059_x1.......................ggugcgccgccggcaccA....................................................................... 1
Gi5_9130021_x1.....................................................................Ggcggccccgggaggcagagcccu.................... 1
Gi5_8902937_x1.....................................................................cgUggccccgggaggcagagcccu.................... 1
Gi3_28899353_x1....................cggggugcgccgccggGa.......................................................................... 1
Gi5_9082987_x1.....................................................................cgcggccccgggaggcagagcccC.................... 1
```

```
Provisional ID   : GLCHR04_4458
Score total      :       44.7
Score for star read(s)    3.9
Score for read counts    40.4
Score for mfe    :        2.6
Score for randfold       -2.2
Score for cons. seed
Total read count :         91
Mature read count:         89
Loop read count  :          0
Star read count  :          2
```

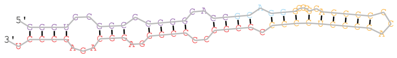

```
                             Star                                             Mature
5'- agcucgcggccucgcuguacgcggggugcgccgccggcaccgcagggcccgcagcccccagggcuccccgcggccccgggaggcagagcccuccggcgcgcaggccgucgag  -3'  obs
    agcucgcggccucgcuguacgcgggugcgccgccggcaccgcagggcccgcagcccccagggcuccccgcggccccgggaggcagagcccuccggcgcgcaggccgucgag       exp
    ..((((((((((((.((.((.(((((..(((.((((..((((.(((...(.(((((...)))))))))).))))..))))..)))...))))).))))))).)))))).))))

Gi5_7635768_x70......................................................................cgcggccccgggaggcagagcccu.................... 0
Gi5_8549593_x6.......................................................................cgcggccccgggaggcagaAcccu.................... 1
Gi5_8829463_x2.......................................................................cUcggccccgggaggcagagcccu.................... 1
Gi5_8753515_x2.......................................................................cgcggccccgggaggcagCgcccu.................... 1
Gi5_8902937_x1.......................................................................cgUggccccgggaggcagagcccu.................... 1
Gi5_9083292_x1.......................................................................cgcgAccccgggaggcagagcccu.................... 1
Gi2_11335059_x1.........................ggugcgccgccggcaccA....................................................................... 1
Gi5_9130021_x1.......................................................................Ggcggccccgggaggcagagcccu.................... 1
Gi5_9082987_x1.......................................................................cgcggccccgggaggcagagcccC.................... 1
Gi3_28899353_x1......................cggggugcgccgccggGa.......................................................................... 1
Gi5_8981940_x1.......................................................................cCcggccccgggaggcagagcccu.................... 1
Gi5_9060210_x1.......................................................................cgcggccccgggaggcUgagcccu.................... 1
Gi5_9085006_x1.......................................................................cgcggccccgggGggcagagcccu.................... 1
Gi5_9006297_x1.......................................................................cgcggcccNgggaggcagagcccu.................... 1
Gi3_28676375_x1........................gggugcgccgccggGacc........................................................................ 1
Gi5_9032993_x1.......................................................................cgcggccccggUaggcagagcccu.................... 1
```
