# Supplementary material for: Giardia lamblia miRNAs as a new diagnostic tool for human giardiasis
Source: PLoS Negl Trop Dis. 2019 Jun 17;13(6):e0007398. doi: 10.1371/journal.pntd.0007398 (PMC6597124; doi:10.1371/journal.pntd.0007398)
Supplement: S2 Fig — The sequences are sorted by abundance and their source library is specified (as in S1 Text). (PDF) [file pntd.0007398.s008.pdf]

S2 Figure

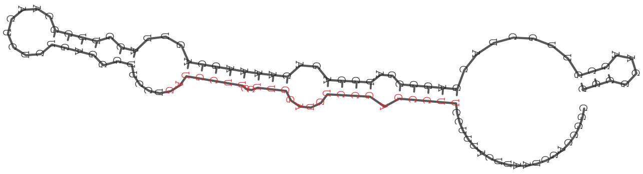

```
>GLCHR05_5465
pri_seq          cgcgcaagcguugcuacgaggcgauggagacaaaagcaguuacguucgcaacucucugaggguuccugaugcuuccuuggauguccgagccuuccuuuacuuaaucgaccggc
pri_struct       (((....))).........((((((.(((.....((.((((.(....).))))))..((((((((..(((...((....)).))).)))))))).....))).)))).))... #MM
RNAfold          (((....))).......(((((..((((..((((((((...((.((((........)))).))......)))))..)))....)))).)))))....................
miR5             ...................................................................gaugcuuccuuggauguccgagccuu.................... 0



Gi3_19756878_x767..........................................................................ccuuggauguccgagccuu.................... 0
Gi3_20371972_x624......................................................................gcuuccuuggauguccgagccuu.................... 0
Gi3_20831703_x531.........................................................................uccuuggauguccgagccuu.................... 0
Gi3_21816573_x374..................aggcgauggagacaaaagcagu......................................................................... 0
Gi3_21935972_x357.................gaggcgauggagacaaaagcagu......................................................................... 0
Gi3_22578925_x279...................................................................gaugcuuccuuggauguccgagccuu.................... 0
Gi2_7660723_x270 ......................................................................gcuuccuuggauguccgagccuu.................... 0
Gi3_22700524_x267.....................................................................ugcuuccuuggauguccgagccuu.................... 0
Gi1_2867381_x266 ......................................................................gcuuccuuggauguccgagccuu.................... 0
Gi3_22923146_x246..........................................................................ccuuggauguccgagccuA.................... 1
Gi3_22967603_x242...........................................................................cuuggauguccgagccuuA................... 1
Gi3_22970507_x242...................................................................gaugcuuccuuggauguccg.......................... 0
Gi5_6296647_x235 ....................................................................augcuuccuuggauguccgagccuu.................... 0
Gi3_23078057_x233...........................................................................cuuggauguccgagccuu.................... 0
Gi3_23375397_x210.........................................................................uccuuggauguccgagccuuA................... 1
Gi3_23415721_x207..................................................................ugaugcuuccuuggauguccgagcc...................... 0
Gi3_23447075_x205........................................................................uuccuuggauguccgagccuu.................... 0
Gi3_23581225_x196...................ggcgauggagacaaaagcaguu........................................................................ 0
Gi2_8177666_x195 ........................................................................uuccuuggauguccgagccu..................... 0
Gi5_6554375_x187 ...........................................................................cuuggauguccgagccuu.................... 0
Gi1_3047861_x186 ...........................................................................cuuggauguccgagccuuA................... 1
Gi3_23718298_x186................cgaggcgauggagacaaaagcagu......................................................................... 0
Gi3_23767757_x183................................................................ccugaugcuuccuuggauguccgagccuu.................... 0
Gi1_3064383_x181 ....................................................................augcuuccuuggauguccgagccuu.................... 0
Gi2_8347457_x175 ........................................................................uuccuuggauguccgagccuu.................... 0
Gi2_8345882_x175 .........................................................................uccuuggauguccgagccuu.................... 0
Gi5_6648918_x174 .........................................................................uccuuggauguccgagccuuc................... 0
Gi1_3099532_x170 .........................................................................uccuuggauguccgagccu..................... 0
Gi2_8391088_x170 .....................................................................ugcuuccuuggauguccgagcc...................... 0
Gi3_24063987_x164...............acgaggcgauggagacaaaagcagu......................................................................... 0
Gi3_24134126_x159.......................................................................cuuccuuggauguccgagccuu.................... 0
Gi2_8554896_x153 ..........................................cguucgcaacucucugagg.................................................... 0
Gi3_24403808_x143...................ggcgauggagacaaaagcag.......................................................................... 0
Gi3_24432711_x141.........................................................................uccuuggauguccgagccuuccuu................ 0
Gi2_8750698_x134 .......................................................................cuuccuuggauguccgagccuu.................... 0
Gi3_24625025_x130.................................................................cugaugcuuccuuggauguccgagccuu.................... 0
Gi3_24654048_x128....................................................................augcuuccuuggauguccgagccuu.................... 0
Gi3_24790166_x120.....................................aguuacguucgcaacucucugaggg................................................... 0
Gi2_8940367_x118 ..................aggcgauggagacaaaagcaguu........................................................................ 0
Gi2_8931989_x118 ...................................................................gaugcuuccuuggauguccgagccu..................... 0
Gi2_8952434_x117 ................................................................ccugaugcuuccuuggauguccga......................... 0
Gi3_24864077_x116...............................................................uccugaugcuuccuuggauguccgagccuu.................... 0
Gi2_8962899_x116 ...................ggcgauggagacaaaagc............................................................................ 0
Gi3_24922850_x112...................ggcgauggagacaaaagcagu......................................................................... 0
Gi5_7190615_x111 ..................aggcgauggagacaaaagcaguu........................................................................ 0
Gi2_9031986_x110 ....................................................................augcuuccuuggauguccgagcc...................... 0
Gi5_7345870_x96  ...................................................................gaugcuuccuuggauguccgagccuu.................... 0
Gi1_3417573_x95  .........................................................................uccuuggauguccgagccuuc................... 0
Gi1_3422317_x94  ...................................................................gaugcuuccuuggauguccgagccuu.................... 0
Gi1_3426077_x94  ..........................................................................ccuuggauguccgagccuu.................... 0
Gi1_3430184_x93  ...........................................................................cuuggauguccgagccuu.................... 0
Gi2_9235789_x93  ..........uugcuacgaggcgauggagac.................................................................................. 0
Gi2_9226489_x93  ......................................guuacguucgcaacucucugaggg................................................... 0
Gi2_9248224_x92  .................gaggcgauggagacaaaagcag.......................................................................... 0
Gi3_25318313_x90 ..................aggcgauggagacaaaagcaguuacgu.................................................................... 0
Gi2_9296450_x87  ...................ggcgauggagacaaaagcaA.......................................................................... 1
Gi3_25470902_x81 ................cgaggcgauggagacaaaagca........................................................................... 0
Gi3_25538858_x78 ..................aggcgauggagacaaaagcaguA........................................................................ 1
Gi3_25534412_x78 .....................................................................ugcuuccuuggauguccgagccu..................... 0
Gi2_9426389_x76  ................................................................ccugaugcuuccuuggauguccgagccu..................... 0
Gi3_25595535_x75..................................................................cugaugcuuccuuggauguccgagccu..................... 0
Gi5_7596870_x74...................gaggcgauggagacaaaagcagu......................................................................... 0
Gi2_9472151_x72...................................................................ugaugcuuccuuggauguccgagccuu.................... 0
Gi3_25660464_x71..................gaggcgauggagacaaaagcaguu........................................................................ 0
Gi2_9497191_x70.......................................................................ugcuuccuuggauguccgagccuu.................... 0
Gi2_9501531_x70....................aggcgauggagacaaaagcagu......................................................................... 0
Gi3_25702465_x69...................aggcgauggagacaaaagcaguua....................................................................... 0
Gi1_3551891_x68...................................................................ugaugcuuccuuggauguccgagccuu.................... 0
Gi3_25760977_x66.......................................................................Ucuuccuuggauguccgagccuu.................... 1
Gi3_25804281_x63...................................................acucucugaggguuccugaugcuuccu.................................... 0
Gi3_25817448_x63................................................................uccugaugcuuccuuggauguccgagcc...................... 0
Gi3_25824133_x62..................Uaggcgauggagacaaaagcagu......................................................................... 1
Gi1_3595760_x60...........................................................................uccuuggauguccAagccu..................... 1
Gi2_9625265_x60...................gaggcgauggagacaaaagcagu......................................................................... 0
Gi3_25924246_x57..................gaggcgauggagacaaaagc............................................................................ 0
Gi3_25931542_x57................acgaggcgauggagacaaaagc............................................................................ 0
Gi4_462528_x57.....................................................................cugaugcuuccuuggauguccgagccuuc................... 0
Gi2_9724418_x52.................cgaggcgauggagacaaaagcagu......................................................................... 0
Gi1_3641154_x51.....................ggcgauggagaUaaaagc............................................................................ 1
Gi2_9755045_x50........................................................................gcuuccuuggauguccgagccuuccuA................ 1
Gi3_26091277_x49...................aggcgauggagacaaaag............................................................................. 0
Gi5_7917279_x47....................aggcgauggagacaaaagcaguuacg..................................................................... 0
```
