# Supplementary material for: Giardia lamblia miRNAs as a new diagnostic tool for human giardiasis
Source: PLoS Negl Trop Dis. 2019 Jun 17;13(6):e0007398. doi: 10.1371/journal.pntd.0007398 (PMC6597124; doi:10.1371/journal.pntd.0007398)
Supplement: S1 Text — The output shows collapsed read pile-ups for every proposed miRNA precursor and lists reasons for disqualification. The sequences are sorted by abundance and their source library is specified. For example, the sequence “Gi3_8900428_x63185” was found 63185 times in library Gi3 (PDF) [file pntd.0007398.s003.pdf]

```
# This file shows the first 100K lines of the full S1_Text file, which can be accessed at:
# https://rockefeller.box.com/s/eao9z6hop9va8hqkgajo528473f3402c
#
#
>GLCHR05_6861
The candidate has been discarded because the structure is inconsistent with Dicer processing:
the candidate mature sequence does not form part of a likely miRNA duplex
exp           nnnnnnnnnnnnnnnnnnnnnnnnnnnnnnnnnnnnnnnnnnnnnnnnnnnnnnnnnnnnnnnnnnnnnnnnnnnnnnnnnnnnnnnnnnnnnnnnnnnnnnnnnnnnnnnn
pri_seq       cucacccaacagauccggguucgauucccggcgccggagcguuucccgccuuccuucgucuuagcauacgccgccccacggugggcccagacccuaaguaucgauuugcaaa
pri_struct    .........((((((((((..((....(((....)))..))...)))).........((((..((...(((((.....))))).))..))))..........))))))....      #MM



Gi3_8900428_x63185.................gguucgauucccggcgccggagc........................................................................  0
Gi3_9927636_x38745.................gguucgauucccggcgccggagcC.......................................................................  1
Gi2_2216035_x30808.................gguucgauucccggcgccggagc........................................................................  0
Gi1_1331494_x26933..................guucgauucccggcgccgg...........................................................................  0
Gi3_12301936_x14323.................gguucgauucccggcgccgga.......................................................................... 0
Gi3_12467744_x13384..................guucgauucccggcgccgg........................................................................... 0
Gi3_12507553_x13080..................guucgauucccggcgccggagcC....................................................................... 1
Gi2_2855789_x12876.................gguucgauucccggcgccggagcC.......................................................................  1
Gi3_12546417_x12822.................gguucgauucccggcgccggag......................................................................... 0
Gi3_12730796_x11530..................guucgauucccggcgccgga.......................................................................... 0
Gi5_3589931_x10963..................guucgauucccggcgccgg...........................................................................  0
Gi2_3032616_x10345..................guucgauucccggcgccggagc........................................................................  0
Gi5_3654433_x9879..................guucgauucccggcgccg............................................................................  0
Gi3_13160908_x9669..................guucgauucccggcgccggagc........................................................................  0
Gi2_3177371_x8982..................guucgauucccggcgccgg...........................................................................  0
Gi5_3728878_x8684.................gguucgauucccggcgccggagc........................................................................  0
Gi3_13864308_x6910.................gguucgauucccggcgccg............................................................................  0
Gi2_3537957_x6599..................guucgauucccggcgccggagcC.......................................................................  1
Gi3_14018747_x6489.................Uguucgauucccggcgccggagc........................................................................  1
Gi3_14430267_x5377..................guucgauucccggcgccggag.........................................................................  0
Gi5_3990121_x5185.................gguucgauucccggcgccggagcC.......................................................................  1
Gi3_14604826_x4914.................gguucgauucccggcgccgg...........................................................................  0
Gi1_1946380_x4673.................gguucgauucccggcgccgg...........................................................................  0
Gi3_14790128_x4604.................gUuucgauucccggcgccggagc........................................................................  1
Gi2_3825436_x4527.................gguucgauucccggcgccggag.........................................................................  0
Gi2_3883487_x4338.................gguucgauucccggcgcc.............................................................................  0
Gi5_4133188_x4096.................gguucgauucccggcgccg............................................................................  0
Gi3_15580261_x3458.................gguucgauucccggcgcc.............................................................................  0
Gi2_4129219_x3450.................gguucgauucccggcgccg............................................................................  0
Gi5_4342263_x3360.................gguucgauucccggcgccgga..........................................................................  0
Gi2_4175715_x3240.................Uguucgauucccggcgccggagc........................................................................  1
Gi2_4266688_x3008..................guucgauucccggcgccg............................................................................  0
Gi2_4338576_x2763.................gguucgauucccggcgccgga..........................................................................  0
Gi3_16196749_x2745..................guucgauucccggcgccg............................................................................  0
```

```
Gi5_4466747_x2732..................guucgauucccggcgccgga..........................................................................   0
Gi1_2098525_x2672..................Uuucgauucccggcgccgg...........................................................................   1
Gi1_2106457_x2553..................guucgauucccggcgccggagcC.......................................................................   1
Gi5_4584884_x2359.................gguucgauucccggcgccgg...........................................................................   0
Gi2_4550526_x2301.................gUuucgauucccggcgccggagc........................................................................   1
Gi3_16719768_x2271....................ucgauucccggcgccggagcC.......................................................................  1
Gi1_2160670_x2236..................guucgauucccggcgccgga..........................................................................   0
Gi2_4659874_x2088..................guucgauucccggcgccgga..........................................................................   0
Gi5_4689295_x2063..................guucgauucccggcgccggagcC.......................................................................   1
Gi2_4678540_x2059..................guucgauucccggcgccggag.........................................................................   0
Gi5_4746918_x1924.................gguucgauucccggcgcc.............................................................................   0
Gi5_4797320_x1809..................guucgauucccggcgccggagc........................................................................   0
Gi5_4829364_x1752.................gguucgauucccggcgccggag.........................................................................   0
Gi5_4856838_x1692cucacccaacagauccgg..............................................................................................   0
Gi3_17723670_x1584.................Uguucgauucccggcgccgga..........................................................................  1
Gi1_2263260_x1508..................guucgauucccggcgccg............................................................................   0
Gi1_2279516_x1437..................gGucgauucccggcgccgg...........................................................................   1
Gi3_18002792_x1423....................ucgauucccggcgccggagc........................................................................  0
Gi3_18114895_x1376.................Uguucgauucccggcgccggag.........................................................................  1
Gi2_5265076_x1323....................ucgauucccggcgccggagc........................................................................   0
Gi3_18253831_x1317..................Uuucgauucccggcgccgg...........................................................................  1
Gi1_2332554_x1264.................gguucgauucccggcgccggagc........................................................................   0
Gi1_2340050_x1226..................guucgauucccggcgccggagc........................................................................   0
Gi3_18631876_x1148..................Uuucgauucccggcgccgga..........................................................................  1
Gi5_5178308_x1083..................Uuucgauucccggcgccgg...........................................................................   1
Gi5_5191186_x1059..................Uuucgauucccggcgccg............................................................................   1
Gi3_18881639_x1044.................gUuucgauucccggcgccgga..........................................................................  1
Gi2_5612893_x1032..................Uuucgauucccggcgccggagc........................................................................   1
Gi3_18985137_x1003..................Uuucgauucccggcgccggagc........................................................................  1
Gi3_19108956_x964.................gUuucgauucccggcgccggag.........................................................................   1
Gi5_5277356_x931.................Uguucgauucccggcgccggagc........................................................................    1
Gi3_19223083_x923...................uucgauucccggcgccggagc........................................................................   0
Gi3_19277081_x906......................gauucccggcgccggagc........................................................................   0
Gi2_5857907_x868..................Uuucgauucccggcgccgg...........................................................................    1
Gi1_2480982_x805.................gguucgauucccggcgccg............................................................................    0
Gi3_19615623_x803.................Uguucgauucccggcgccg............................................................................   1
G1a_3739453_x774.................gguucgauucccggcgccggagc........................................................................    0
Gi2_6110175_x725.................gguucgauucccggcgccgg...........................................................................    0
Gi3_20044512_x691..................gGucgauucccggcgccgg...........................................................................   1
Gi3_20062420_x686..................guucgauucccggcgccgC...........................................................................   1
Gi5_5504875_x652.................gUuucgauucccggcgccggagc........................................................................    1
Gi1_2562309_x634.................gguucgauucccggcgccgga..........................................................................    0
Gi3_20331714_x633.................Aguucgauucccggcgccggagc........................................................................   1
Gi3_20356944_x628.....................cgauucccggcgccggagc........................................................................   0
Gi3_20450237_x607...................uucgauucccggcgccgga..........................................................................   0
Gi3_20477458_x603...................uucgauucccggcgccggagcC.......................................................................   1
Gi3_20490687_x600..................gGucgauucccggcgccgga..........................................................................   1
```

```
Gi3_20592804_x574......................gauucccggcgccggagcC.......................................................................  1
Gi3_20641249_x565.................gguucgauucccggcgccgC...........................................................................  1
Gi2_6524433_x562..................gGucgauucccggcgccggagc........................................................................  1
Gi3_20654213_x562..................guucgaCucccggcgccggagc........................................................................  1
Gi3_20711587_x552.................gguucgauNcccggcgccggagc........................................................................  1
Gi3_20742876_x547.................gUuucgauucccggcgccg............................................................................  1
Gi5_5635437_x543..................gGucgauucccggcgccgg...........................................................................  1
Gi3_20766281_x542....................ucgauucccggcgccggag.........................................................................  0
Gi3_20813076_x534..................Uuucgauucccggcgccggag.........................................................................  1
Gi1_2629414_x520.................Uguucgauucccggcgccgg...........................................................................  1
Gi3_20890919_x518....................ucgauucccggcgccgga..........................................................................  0
Gi3_20912056_x514.................Uguucgauucccggcgccgg...........................................................................  1
Gi3_20940158_x508cucacccaacagauccgg..............................................................................................  0
Gi2_6718036_x497......................gauucccggcgccggagc........................................................................  0
Gi2_6721016_x496..................gGucgauucccggcgccgg...........................................................................  1
Gi3_21017851_x495.......................auucccggcgccggagcC.......................................................................  1
Gi3_21069318_x486.................gguucgauucccggcgccggCgc........................................................................  1
Gi2_6751449_x486.................Uguucgauucccggcgcc.............................................................................  1
Gi2_6769824_x481.................Uguucgauucccggcgccggag.........................................................................  1
Gi2_6779412_x478....................ucgauucccggcgccggagcC.......................................................................  1
Gi1_2664335_x471cucacccaacagauccgg..............................................................................................  0
Gi5_5742002_x468..................gGucgauucccggcgccg............................................................................  1
Gi3_21241144_x461..................gGucgauucccggcgccggagc........................................................................  1
Gi1_2686601_x437...................uucgauucccggcgccgg...........................................................................  0
Gi5_5802448_x432.................Uguucgauucccggcgccg............................................................................  1
Gi3_21433943_x429.................ggCucgauucccggcgccggagc........................................................................  1
Gi2_7002965_x415.................Uguucgauucccggcgccg............................................................................  1
G1a_4132002_x412.................gguucgauucccggcgccggag.........................................................................  0
Gi3_21767117_x381.................Uguucgauucccggcgcc.............................................................................  1
Gi5_5902217_x373.................Uguucgauucccggcgccgga..........................................................................  1
Gi3_21824415_x373.................gUuucgauucccggcgccgg...........................................................................  1
Gi3_21849616_x369.....................cgauucccggcgccggagcC.......................................................................  1
Gi3_21850354_x369.................gguucgauucccggcgccggagcCu......................................................................  1
Gi1_2749901_x367.................gguucgauucccggcgccggag.........................................................................  0
Gi5_5915080_x362......................gauucccggcgccggagc........................................................................  0
Gi2_7279530_x347......................gauucccggcgccggagcC.......................................................................  1
Gi3_22045248_x341.........cagauccggguucgauuccc...................................................................................  0
Gi1_2780262_x336.................gUuucgauucccggcgccgg...........................................................................  1
Gi2_7348465_x332.................Aguucgauucccggcgccggagc........................................................................  1
Gi2_7360380_x330.................gUuucgauucccggcgccggag.........................................................................  1
Gi5_6005812_x327.................gUuucgauucccggcgccg............................................................................  1
Gi2_7428877_x314..................Uuucgauucccggcgccg............................................................................  1
Gi3_22310260_x308.................gguucgauucccggcgccggGgc........................................................................  1
Gi2_7451906_x308..................guucgaCucccggcgccggagc........................................................................  1
Gi3_22313956_x308.................gUuucgauucccggcgcc.............................................................................  1
Gi3_22350015_x303.............uccggguucgauucccggcgccgga..........................................................................  0
Gi3_22369960_x301..................gGucgauucccggcgccggag.........................................................................  1
```

```
G1a_4343709_x299.................gguucgauucccggcgccggagcC....................................................................... 1
Gi3_22406772_x297.................gguucgauuccUggcgccggagc........................................................................ 1
Gi3_22426894_x295..................Uuucgauucccggcgccg............................................................................ 1
Gi3_22440421_x293.................Cguucgauucccggcgccggagc........................................................................ 1
Gi3_22470199_x290...................uucgauucccggcgccgg........................................................................... 0
Gi1_2833307_x289Uucacccaacagauccgg.............................................................................................. 1
Gi1_2837629_x286.................gguucgauucccggcgccggagcC....................................................................... 1
Gi2_7584647_x283.................gUuucgauucccggcgcc............................................................................. 1
Gi2_7604625_x280...................uucgauucccggcgccgg........................................................................... 0
Gi2_7623265_x277.................Uguucgauucccggcgccgga.......................................................................... 1
Gi2_7633467_x275.................gguucgauNcccggcgccggagc........................................................................ 1
Gi3_22667789_x270..................guucgauucccggcgccggagcCu...................................................................... 1
Gi1_2865516_x267..................guucgauucccggcgccggagcCu...................................................................... 1
Gi5_6179994_x266..................Uuucgauucccggcgccgga.......................................................................... 1
G1a_4422156_x265.................gguucgauucccggcgccgga.......................................................................... 0
Gi3_22715945_x265..........agauccggguucgauuccc................................................................................... 0
Gi1_2868710_x265....................ucgauucccggcgccggagc........................................................................ 0
Gi3_22720450_x265.................gguucgaCucccggcgccggagc........................................................................ 1
Gi2_7692801_x265.................gUuucgauucccggcgccg............................................................................ 1
Gi1_2876324_x260..................guucgaCucccggcgccgg........................................................................... 1
Gi5_6222628_x254..........agauccggguucgauuccc................................................................................... 0
Gi5_6228460_x253....cccaacagauccggguuc.......................................................................................... 0
Gi3_22899164_x248..................guucgaCucccggcgccgga.......................................................................... 1
Gi5_6245734_x248.................Uguucgauucccggcgcc............................................................................. 1
Gi4_339684_x242.................gguucgauucccggcgccgg........................................................................... 0
Gi3_22994850_x240.................gguucgGuucccggcgccggagc........................................................................ 1
Gi1_2928825_x236...................uucgauucccggcgccggagcC....................................................................... 1
Gi3_23052063_x235..cacccaacagauccggguuc.......................................................................................... 0
Gi5_6308565_x232.................Uguucgauucccggcgccgg........................................................................... 1
Gi5_6317809_x230....................ucgauucccggcgccggag......................................................................... 0
Gi2_7899826_x230...................uucgauucccggcgccggagcC....................................................................... 1
Gi1_2938596_x230.................Cguucgauucccggcgccgg........................................................................... 1
Gi5_6326282_x228....................ucgauucccggcgccggagc........................................................................ 0
Gi5_6331966_x226..................guucgauucccggcgccggag......................................................................... 0
Gi2_7938475_x225.................gguucgauucccggcgccggCgc........................................................................ 1
Gi3_23193875_x224.................gguucgauuccUggcgccggag......................................................................... 1
Gi3_23200799_x223..................guucgaCucccggcgccgg........................................................................... 1
Gi1_2963766_x219..................gCucgauucccggcgccgg........................................................................... 1
G1a_4546649_x218.................gguucgauucccggcgccgg........................................................................... 0
Gi2_7994399_x217..................Uuucgauucccggcgccggag......................................................................... 1
G1a_4557874_x214.................gguucgauucccggcgccg............................................................................ 0
Gi5_6398702_x214.................gUuucgauucccggcgccgga.......................................................................... 1
Gi2_8017908_x214...................uucgauucccggcgccggagc........................................................................ 0
Gi5_6417005_x211....................ucgauucccggcgccggagcC....................................................................... 1
Gi3_23382327_x210..................guucgauucccggcgccgA........................................................................... 1
Gi1_2985636_x210..................Uuucgauucccggcgccgga.......................................................................... 1
Gi5_6461919_x203.................Uguucgauucccggcgccggag......................................................................... 1
```

```
Gi1_3010075_x201...................uucgauucccggcgccggagc........................................................................   0
Gi3_23506713_x201.....caacagauccggguucgauuccc....................................................................................   0
Gi2_8127732_x200.................ggCucgauucccggcgccggagc........................................................................   1
Gi3_23516931_x200.................gguCcgauucccggcgccggagc........................................................................   1
Gi2_8144076_x198..................Uuucgauucccggcgccgga..........................................................................   1
Gi3_23560200_x197...........gauccggguucgauuccc...................................................................................   0
Gi3_23573973_x196.................gCuucgauucccggcgccggagc........................................................................   1
Gi3_23641643_x191..................guucgauucccggUgccggag.........................................................................   1
G1a_4632800_x191..................guucgauucccggcgccggagcC.......................................................................   1
Gi3_23694905_x188................Agguucgauucccggcgccggagc........................................................................   1
Gi3_23697725_x188.................ggGucgauucccggcgccggagc........................................................................   1
G1a_4644493_x187..................guucgauucccggcgccggagc........................................................................   0
Gi5_6557180_x187.ucacccaacagauccggguu...........................................................................................   0
Gi2_8259226_x185....cccaacagauccggguuc..........................................................................................   0
Gi3_23749033_x184.................gAuucgauucccggcgccggagc........................................................................   1
G1a_4658536_x183.................gguucgauucccggcgcc.............................................................................   0
Gi4_367620_x179..................guucgauucccggcgccgg...........................................................................   0
Gi5_6615364_x179..................Uuucgauucccggcgccggagc........................................................................   1
Gi1_3070143_x179..........agauccggguucgauuccc...................................................................................   0
Gi5_6632435_x177.................gguucgauucccggcgccggagcCu......................................................................   1
Gi3_23865777_x176.....................cgauucccggcgccggag.........................................................................   0
Gi1_3086057_x175..................Uuucgauucccggcgccg............................................................................   1
Gi3_23948305_x171..................guucgaCucccggcgccggag.........................................................................   1
Gi2_8390578_x170.................gUuucgauucccggcgccgga..........................................................................   1
Gi5_6685354_x169.................gUuucgauucccggcgccgg...........................................................................   1
Gi1_3103256_x169.ucacccaacagauccgggu............................................................................................   0
Gi2_8419886_x166..................gGucgauucccggcgccg............................................................................   1
Gi2_8436782_x165.................gguucgauucccggcgccggGgc........................................................................   1
Gi5_6719756_x164......................gauucccggcgccggagcC.......................................................................   1
G1a_4721617_x163..................guucgauucccggcgccgg...........................................................................   0
Gi3_24072831_x163.................gguucgauucccggUgccggagc........................................................................   1
Gi5_6738710_x162..................guucgaCucccggcgccgg...........................................................................   1
Gi3_24090728_x162...............cggguucgauucccggcgccgga..........................................................................   0
Gi3_24111706_x161..................gGucgauucccggcgccg............................................................................   1
Gi3_24113960_x161.................gguucgauCcccggcgccggagc........................................................................   1
Gi2_8472510_x161....................ucgauucccggcgccggag.........................................................................   0
Gi5_6756601_x160...................uucgauucccggcgccgg...........................................................................   0
Gi3_24142553_x159.................gguucgauucccAgcgccggagc........................................................................   1
Gi3_24150185_x159.ucacccaacagauccggguuc..........................................................................................   0
Gi5_6778507_x158.............uccggguucgauucccggc................................................................................   0
Gi2_8512592_x157.................gguucgaCucccggcgccggagc........................................................................   1
Gi2_8518708_x156.....................cgauucccggcgccggagcC.......................................................................   1
Gi1_3162430_x154.................gguucgauucccggcgccggagcCu......................................................................   1
Gi5_6811730_x153..................guucgauucccggcgccggaCc........................................................................   1
Gi1_3167020_x152..................Auucgauucccggcgccgg...........................................................................   1
Gi3_24257654_x151.................Aguucgauucccggcgccggag.........................................................................   1
Gi3_24263996_x151.................gguucgauucccggcgccggagcA.......................................................................   1
```

Gi2_8579332_x150..........agauccggguucgauucc.. 0
Gi3_24312302_x148.................gguucgauucccggcgAcggagc.. 1
Gi3_24331334_x147............auccggguucgauucccggcgccgga.. 0
Gi1_3193841_x145..................guucgauuNccggcgccgg... 1
Gi3_24415889_x142.................gguucAauucccggcgccggagc.. 1
Gi3_24417877_x142..................guucgauucccggcgccggaC... 1
Gi2_8670143_x142.................Cguucgauucccggcgccggagc.. 1
Gi5_6930592_x139.......................auucccggcgccggagcC... 1
Gi5_6938237_x139..................guucgauucccggcgccggagcCu.. 1
Gi3_24512108_x136.................ggAucgauucccggcgccggagc.. 1
Gi2_8736673_x136..................gGucgauucccggcgccgga.. 1
Gi2_8744776_x135..................guuUgauucccggcgccggag... 1
Gi3_24551410_x134................ggguucgauucccggcgccggagc.. 0
Gi5_6979068_x134.................gguucgauuUccggcgccgg... 1
Gi5_6975584_x134.................gUuucgauucccggcgccggag... 1
Gi1_3244829_x134..................Uuucgauucccggcgccggagc.. 1
Gi3_24613453_x131.................gguuUgauucccggcgccggagc.. 1
Gi1_3258716_x131cucacccaacagauccCg.. 1
Gi5_7003636_x131..................gGucgauucccggcgccgga.. 1
Gi5_7008202_x130.................gUuucgauucccggcgcc... 1
Gi5_7036657_x127..................guucgaCucccggcgccg.. 1
Gi1_3271991_x127..................guCcgauucccggcgccgg... 1
Gi3_24687352_x126.................Aguucgauucccggcgccgga.. 1
Gi3_24694660_x126.................gguucgauucccggcgccggagA.. 1
Gi3_24739411_x123.................gguucgauNcccggcgccgga.. 1
Gi3_24749611_x122.................gguucgauucccggcgcUggagc.. 1
Gi2_8905500_x121.................gguCcgauucccggcgccggagc.. 1
Gi3_24812582_x119......caacagauccggguucga.. 0
Gi1_3311683_x118.................Uguucgauucccggcgccggagc.. 1
Gi5_7131131_x117.................Cguucgauucccggcgcc... 1
Gi3_24835557_x117.................gguucgauNcccggcgccggag... 1
Gi2_8951030_x117.................Uguucgauucccggcgccgg... 1
Gi3_24942169_x111.................gguucgauucccggcgccggaCc.. 1
Gi2_9037156_x110....................ucgauucccggcgccgga.. 0
Gi3_25015334_x107................ggguucgauucccggcgccggag... 0
Gi3_25009663_x107......................Uauucccggcgccggagc.. 1
Gi3_25019293_x107..................guucgaCucccggcgccg.. 1
G1a_4940392_x105...................uucgauucccggcgccggagc.. 0
Gi4_415438_x105.................gguucgauucccggcgccggagc.. 0
Gi1_3372775_x105..................gGucgauucccggcgccgga.. 1
Gi2_9109341_x104..................gGucgauucccggcgccggag... 1
Gi2_9115834_x103.................gguucgGuucccggcgccggagc.. 1
Gi3_25104011_x102.................gguAcgauucccggcgccggagc.. 1
Gi3_25119990_x101.................gguucgCuucccggcgccggagc.. 1
Gi5_7296674_x101.................gguucgauucccggcgccggagcgu.. 0
Gi2_9144974_x100.....................cgauucccggcgccggagcguuA.. 1
Gi1_3403571_x98.................gUuucgauucccggcgccggagc.. 1

```
Gi1_3410380_x97.................gguucgauucccggcgccggagcU......................................................................    1
Gi2_9186957_x97..................guucgaCucccggcgccgg..........................................................................    1
Gi1_3412405_x96.................gguucgauucccggcgcc............................................................................    0
Gi1_3412885_x96.................gguucgauucccggcgccAg..........................................................................    1
Gi3_25246829_x94..........agauccggguucgauucc...................................................................................    0
Gi5_7369994_x94..................gCucgauucccggcgccg...........................................................................    1
Gi3_25254537_x94.................gguucgauucUcggcgccggagc.......................................................................    1
Gi1_3431858_x93..................Cuucgauucccggcgccgg..........................................................................    1
Gi3_25304938_x91.................gguucgauucccggcgUcggagc.......................................................................    1
Gi3_25293472_x91..................Auucgauucccggcgccgga.........................................................................    1
Gi1_3443389_x90................Agguucgauucccggcgccgg..........................................................................    1
Gi2_9277568_x89.................gAuucgauucccggcgccggagc.......................................................................    1
Gi5_7424212_x89........acagauccggguucgauucccggcgc.............................................................................    0
Gi1_3455056_x88..................guucgGuucccggcgccgg..........................................................................    1
Gi5_7436018_x88..................gGucgauucccggcgccggagc.......................................................................    1
Gi2_9296624_x87.................ggGucgauucccggcgccggagc.......................................................................    1
Gi1_3464465_x86.................Uguucgauucccggcgccg...........................................................................    1
Gi3_25413852_x85....cccaacagauccggguucgauuccc..................................................................................    0
Gi3_25425115_x84.........cagauccggguucgauucccggcgccgg..........................................................................    0
Gi3_25423855_x84.................gguucgauucccggcAccggagc.......................................................................    1
Gi3_25418899_x84..................Auucgauucccggcgccggagc.......................................................................    1
Gi3_25424863_x84.................gguucgauucccggcgcAggagc.......................................................................    1
Gi5_7492827_x83.................Aguucgauucccggcgccggagc.......................................................................    1
Gi2_9348290_x83cucacccaacagauccgg.............................................................................................    0
G1a_5050925_x83.................Uguucgauucccggcgccggagc.......................................................................    1
Gi1_3484149_x82..................guucAauucccggcgccgg..........................................................................    1
Gi5_7506722_x82.................gguucgauucccggcgccggagcguu....................................................................    0
Gi3_25483214_x81.................gguucgauucccggcgccggG.........................................................................    1
Gi3_25483376_x81.................gguucgauucccggcgccAgagc.......................................................................    1
Gi1_3491081_x81..................guucgauucccggcgccAg..........................................................................    1
Gi3_25469201_x81.................gguucgauucccUgcgccggagc.......................................................................    1
Gi2_9378699_x80...................uucgauucccggcgccggag........................................................................    0
Gi3_25486936_x80.................ggCucgauucccggcgccgga.........................................................................    1
Gi3_25504765_x79..................guucgauucccggcgccggGg........................................................................    1
Gi1_3501457_x78..................guuUgauucccggcgccgg..........................................................................    1
Gi2_9414179_x77..................Auucgauucccggcgccgg..........................................................................    1
Gi1_3510223_x77..................gGucgauucccggcgccg...........................................................................    1
Gi2_9417028_x77.................ggAucgauucccggcgccggagc.......................................................................    1
Gi5_7559961_x77.....................cgauucccggcgccggagcguuuc..................................................................    0
Gi1_3506604_x77..................guucUauucccggcgccgg..........................................................................    1
Gi4_442433_x77..................guucgauucccggcgccgga.........................................................................    0
Gi3_25568178_x76.................gguucgauucccggcgccggagU.......................................................................    1
Gi2_9443182_x75.................gguuUgauucccggcgccggagc.......................................................................    1
Gi3_25594935_x75..................guucgauuNccggcgccgga.........................................................................    1
Gi5_7589840_x74..................guucgaCucccggcgccggagc.......................................................................    1
Gi3_25620397_x73..................guucgauuNccggcgccgg..........................................................................    1
Gi3_25621492_x73..................Auucgauucccggcgccgg..........................................................................    1
```

```
Gi2_9477623_x72.....................cgauucccggcgccggagcguuuc...................................................................    0
Gi3_25652418_x72.................gguucgauuUccggcgccggagc........................................................................    1
Gi3_25661387_x71.................Cguucgauucccggcgccgga..........................................................................    1
Gi1_3536125_x71.................Uguucgauucccggcgccgga..........................................................................    1
Gi3_25659541_x71................ggguucgauucccggcgccgga..........................................................................    0
G1a_5110245_x71.....................cgauucccggcgccggagcC.......................................................................    1
Gi1_3538184_x71..................gGucgauucccggcgccggagc........................................................................    1
Gi2_9490621_x71.................gCuucgauucccggcgccggagc........................................................................    1
Gi1_3539746_x71..................guucgauucccggUgccgg...........................................................................    1
Gi4_448843_x70cucacccaacagauccggguucg.........................................................................................    0
Gi2_9498941_x70.................gguucgauucccggcgAcggagc........................................................................    1
G1a_5117872_x70..................guucgauucccggcgccg............................................................................    0
Gi3_25690730_x70..................guucgGuucccggcgccgga..........................................................................    1
Gi2_9511814_x69.................gUuucgauucccggcgccgg...........................................................................    1
Gi3_25706398_x69..................guucgauucccggcgccggG..........................................................................    1
Gi3_25697014_x69.................ggCucgauucccggcgccggag.........................................................................    1
Gi3_25693495_x69..................gCucgauucccggcgccgg...........................................................................    1
Gi2_9507881_x69.................gguucgauucccggcgccggagA........................................................................    1
Gi5_7667663_x68..................Auucgauucccggcgccg............................................................................    1
Gi5_7659095_x68.................gguucgauNcccggcgccggagc........................................................................    1
Gi3_25725127_x68.................Aguucgauucccggcgccg............................................................................    1
Gi2_9519724_x68.................gguucgauucccAgcgccggagc........................................................................    1
Gi2_9536503_x67.................gguucgauCcccggcgccggagc........................................................................    1
Gi5_7668269_x67.................gguucgauucccggcgccAg...........................................................................    1
Gi2_9546257_x66.................gguucAauucccggcgccggagc........................................................................    1
Gi5_7691722_x66.........cagauccggguucgauucccggcgccg............................................................................    0
Gi1_3561746_x66..................guucgauCcccggcgccgg...........................................................................    1
Gi1_3559898_x66..................guucgauucccAgcgccgg...........................................................................    1
Gi5_7680238_x66................ggguucgauucccggcgccggagc........................................................................    0
Gi5_7687168_x66....................ucgauucccggcgccgga..........................................................................    0
Gi5_7698366_x65..............ccggguucgauucccggcgccgga..........................................................................    0
Gi3_25769037_x65..................gCucgauucccggcgccggagc........................................................................    1
Gi3_25791259_x64.................Cguucgauucccggcgccggag.........................................................................    1
Gi1_3572008_x64......caacagauccggguucgauucccggc................................................................................    0
Gi2_9577273_x64..................Auucgauucccggcgccggagc........................................................................    1
Gi1_3573800_x64..................guucgauucccggcgcUgg...........................................................................    1
Gi2_9578297_x64.....................cgauucccggcgccggagcguuu....................................................................    0
Gi3_25813920_x63..................guucgauucccggcgccggCgc........................................................................    1
Gi3_25805856_x63.................gguucgauucccggcgccggagcgA......................................................................    1
Gi2_9582921_x63..................guucgaCucccggcgccg............................................................................    1
Gi5_7721679_x63.................gguucgauucccggcgccggagcguuu....................................................................    0
Gi5_7726656_x63..................Auucgauucccggcgccgg...........................................................................    1
Gi1_3575068_x63.................gUuucgauucccggcgccg............................................................................    1
Gi3_25836223_x62.................gguucgauucccggcgAc.............................................................................    1
Gi3_25833123_x62.................gguucgauucccggUgcc.............................................................................    1
Gi3_25826303_x62..................guucgGuucccggcgccggagc........................................................................    1
Gi3_25833619_x62.................gguucgauucccggcgccgAagc........................................................................    1
```

```
Gi2_9593361_x62.................gguucgauucccggcgccggGg........................................................................ 1
Gi1_3588651_x61.........cagauccggguucgauucccggcgcc............................................................................ 0
Gi1_3588041_x61..................guucgauuccUggcgccgg.......................................................................... 1
Gi1_3585662_x61..cacccaacagauccggguucgauuccc.................................................................................. 0
Gi3_25847533_x61.................gguucgauucccggcgccggGg........................................................................ 1
Gi2_9614597_x61.................gguucgauuccUggcgccggagc....................................................................... 1
Gi3_25867609_x60.................gguucgauNcccggcgccg........................................................................... 1
Gi3_25870549_x60.................gguucgauuccAggcgccggagc....................................................................... 1
Gi5_7768413_x59.....................cgauucccggcgccggagcguuuA.................................................................. 1
Gi2_9636740_x59..................guucgauucccggcgccggagcCu..................................................................... 1
Gi5_7785515_x58.................gguucgauucccggcgccggCgc....................................................................... 1
Gi3_25913050_x58..................gCucgauucccggcgccgga......................................................................... 1
Gi2_9646919_x58.................Aguucgauucccggcgccggag........................................................................ 1
Gi2_9663188_x57..................guucgauuNccggcgccggagc....................................................................... 1
Gi3_25956726_x56.................Aguucgauucccggcgccgg.......................................................................... 1
Gi3_25942054_x56.................gguucgauucccggcgccggaAc....................................................................... 1
Gi2_9671918_x56......................Uauucccggcgccggagc....................................................................... 1
Gi3_25948550_x56..................guCcgauucccggcgccgg.......................................................................... 1
Gi2_9670686_x56..................gCucgauucccggcgccggagc....................................................................... 1
Gi3_25940150_x56..................guucgauucccggcgccggagcA...................................................................... 1
Gi3_25954822_x56.................gguucgaCucccggcgccgga......................................................................... 1
Gi2_9670070_x56...................uucgauucccggcgccgga......................................................................... 0
Gi5_7824359_x55..................gCucgauucccggcgccgg.......................................................................... 1
Gi3_25969945_x55.................gguucgauucccggcgcUggag........................................................................ 1
Gi3_25983306_x54.....................cUauucccggcgccggagc....................................................................... 1
Gi2_9702216_x54.................gguucgauucccggcAccggagc....................................................................... 1
Gi3_26016048_x53.................gguucgauucccggAgccggagc....................................................................... 1
Gi3_26032808_x52.....................cgauucccggcgccggagcguuucA................................................................. 1
Gi3_26031092_x52....................Gcgauucccggcgccggagc....................................................................... 1
Gi3_26033432_x52..................Cuucgauucccggcgccgga......................................................................... 1
Gi1_3633854_x52..................guucgauucccggcAccgg.......................................................................... 1
Gi5_7864261_x51.................ggCucgauucccggcgccggagc....................................................................... 1
Gi1_3640695_x51..................guucgauucccggcgcAgg.......................................................................... 1
Gi5_7874053_x51.................gguucgauucccggcgccggagcA...................................................................... 1
Gi3_26052100_x51.................gguucgauucccgAcgccggagc....................................................................... 1
Gi3_26049448_x51..................Cuucgauucccggcgccgg.......................................................................... 1
Gi3_26058286_x50.................gCuucgauucccggcgccggag........................................................................ 1
Gi1_3642664_x50..................guucgauucUcggcgccgg.......................................................................... 1
Gi3_26064286_x50.................gguucgauNcccggcgccgg.......................................................................... 1
Gi3_26077036_x50..................guucgauuNccggcgccggagc....................................................................... 1
Gi3_26069236_x50.................Cguucgauucccggcgccg........................................................................... 1
Gi2_9747145_x50.................gguucgauucccggcgcAggagc....................................................................... 1
Gi3_26057686_x50.................gguuGgauucccggcgccggagc....................................................................... 1
Gi3_26077436_x50.................gguucgauucccggcgccggagcU...................................................................... 1
Gi3_26081820_x49.................gAuucgauucccggcgccggag........................................................................ 1
Gi1_3647644_x49............auccggguucgauucccggcgccgga......................................................................... 0
Gi5_7897160_x49......................Uauucccggcgccggagc....................................................................... 1
```

```
Gi2_9757944_x49..................guucgauucccggcgccAga..........................................................................    1
Gi1_3647301_x49.................gguucgauNcccggcgccgg...........................................................................    1
Gi3_26079272_x49..................guucgGuucccggcgccgg...........................................................................    1
Gi2_9755445_x49.................gguucgauucccggcgccggagcCu......................................................................    1
Gi1_3652936_x49.................gguucgauCcccggcgccgg...........................................................................    1
Gi2_9773741_x48.................Aguucgauucccggcgcc.............................................................................    1
Gi1_3653081_x48..................guucgauucccggcgccggagcA.......................................................................    1
Gi1_3657785_x48.................Aguucgauucccggcgccgg...........................................................................    1
Gi2_9777389_x48..................guCcgauucccggcgccgg...........................................................................    1
Gi3_26113280_x48.................gguucgauCcccggcgccgga..........................................................................    1
Gi2_9772973_x48.................gguucgCuucccggcgccggagc........................................................................    1
Gi3_26130456_x47.................gguucgGuucccggcgccggag.........................................................................    1
Gi2_9784714_x47.................gguucgauucccggcgccggagU........................................................................    1
Gi1_3662555_x47..................guucgauucccggcgUcgg...........................................................................    1
Gi2_9784902_x47..................guucgauucccggcgccggCgc........................................................................    1
Gi5_7923906_x47.................gguucgauucccCgcgccggag.........................................................................    1
Gi5_7937975_x46..................Cuucgauucccggcgccgg...........................................................................    1
Gi3_26153651_x46.................gguucgauuUccggcgccggag.........................................................................    1
G1a_5280140_x46.................gUucgauucccggcgccggagc.........................................................................    1
Gi5_7940367_x46..................guucgGuucccggcgccg............................................................................    1
Gi3_26152501_x46.................gguucgauCcccggcgccggag.........................................................................    1
Gi2_9805034_x46.................gguucgauucccggUgccggagc........................................................................    1
Gi2_9796478_x46..................guucgauucccggcgccggGgc........................................................................    1
Gi3_26153973_x46................ggguucgauucccggcgccg............................................................................    0
Gi5_7938021_x46..................guucgauuNccggcgccg............................................................................    1
Gi2_9809439_x45....................Gcgauucccggcgccggagc........................................................................    1
Gi5_7946502_x45..................guCcgauucccggcgccgg...........................................................................    1
Gi2_9809574_x45.................gguucgauucccgAcgccggagc........................................................................    1
Gi2_9814704_x45.................gguucgauucccggcgcUggagc........................................................................    1
Gi3_26186901_x44.................gguucgauucccAgcgccgga..........................................................................    1
Gi1_3679485_x44..................guGcgauucccggcgccgg...........................................................................    1
Gi2_9825942_x44.................gguucgauucccggcgccggagcA.......................................................................    1
G1a_5301606_x43.................Uguucgauucccggcgccggag.........................................................................    1
Gi5_7973936_x43..................guucgauuNccggcgccgg...........................................................................    1
Gi2_9841797_x43..................guCcgauucccggcgccggagc........................................................................    1
Gi5_7989610_x42.................gguucgauucccggcgccggagcgA......................................................................    1
Gi2_9852685_x42.................gguucgauucUcggcgccggagc........................................................................    1
Gi5_7984822_x42..cacccaacagauccggguucga........................................................................................    0
Gi2_9858523_x42.................gguucgauucccggcgccAgagc........................................................................    1
Gi3_26228521_x42..................guCcgauucccggcgccggagc........................................................................    1
Gi2_9851257_x42...............cggguucgauucccggcgccgga..........................................................................    0
Gi2_9863452_x41..................guucgaCucccggcgccggag.........................................................................    1
Gi1_3695766_x41..................guAcgauucccggcgccgg...........................................................................    1
Gi5_7996569_x41.................gguucgauNcccggcgccg............................................................................    1
Gi3_26252492_x41.................gguucgauucccggcgccggUgc........................................................................    1
Gi3_26260364_x41............auccggguucgauucccggcgccggagc........................................................................    0
Gi5_7993863_x41.................Aguucgauucccggcgccg............................................................................    1
```

```
Gi3_26276010_x40.................gguucgauAcccggcgccggagc.........................................................................   1
Gi1_3697317_x40.................gUuucgauucccggcgccgga...........................................................................   1
Gi3_26276090_x40..................guucgauucccggcgccAga...........................................................................   1
Gi1_3698477_x40..................guucgauucccgAcgccgg............................................................................   1
Gi3_26263930_x40.................gCuucgauucccggcgccgga...........................................................................   1
Gi2_9893236_x39..................Cuucgauucccggcgccgg............................................................................   1
Gi3_26283693_x39.................gguucgaCucccggcgccggag..........................................................................   1
Gi3_26299059_x39..................Cuucgauucccggcgccggagc.........................................................................   1
Gi5_8026280_x39..................guucgauucccggcAccgg............................................................................   1
Gi3_26305572_x39.................gguucgauucAcggcgccggagc.........................................................................   1
Gi3_26305026_x39..........agauccggguucgauucccggcgccggagcC........................................................................   1
Gi2_9912081_x38..................guucgaCucccggcgccgga...........................................................................   1
Gi5_8031125_x38..................guucgauucccggcgccA.............................................................................   1
Gi2_9911093_x38..................guucgauuNccggcgccgg............................................................................   1
Gi3_26318608_x38.................gguucgGuucccggcgccgga...........................................................................   1
Gi3_26320318_x38.................gguucgauucccggcAccgg............................................................................   1
Gi3_26311426_x38.................gguucgauucccggcgccggCg..........................................................................   1
Gi2_9909383_x38..................guucgGuucccggcgccggagc.........................................................................   1
Gi2_9905051_x38.............uccggguucgauucccggcgccgga...........................................................................   0
Gi2_9913069_x38.................gguucgauNcccggcgccggag..........................................................................   1
Gi1_3709984_x38.................gUuucgauucccggcgccggag..........................................................................   1
Gi5_8033481_x38...acccaacagauccggguucgauucc.....................................................................................   0
Gi3_26326702_x38..................guucgauucccggUgccgg............................................................................   1
Gi3_26345147_x37.................ggAucgauucccggcgccgga...........................................................................   1
Gi3_26335638_x37.................gguucgauucccggcgccA.............................................................................   1
Gi5_8046744_x37.................gguucgauNcccggcgccgga...........................................................................   1
Gi5_8052664_x37.................Cguucgauucccggcgccggagc.........................................................................   1
Gi2_9916965_x37.................Aguucgauucccggcgccg.............................................................................   1
Gi3_26331161_x37..................Auucgauucccggcgccggag..........................................................................   1
Gi1_3717732_x37..................guucgauucccggcgAcgg............................................................................   1
Gi3_26349402_x37.................gguucgauucccggcgccgAag..........................................................................   1
Gi2_9924698_x37.................gguucgauucccggcgAc..............................................................................   1
Gi2_9923107_x37.................gguucgauNcccggcgcc..............................................................................   1
Gi1_3715031_x37..................guucgauucccggcgccggag..........................................................................   0
Gi3_26352767_x36.................gguucgauucccggUgccgga...........................................................................   1
Gi3_26372855_x36.................gguucgauucccggcgccggC...........................................................................   1
Gi3_26359031_x36.................gguucgauucccggcAccgga...........................................................................   1
Gi5_8055436_x36........................................guuucccgccuuccuucgucu....................................................   0
Gi2_9929187_x36.................gguucgauucccggAgccggagc.........................................................................   1
Gi2_9939159_x36.................gguucgauucccggcgccgAagc.........................................................................   1
Gi1_3721587_x36cucacccaacagauccggguucgauucc.....................................................................................   0
Gi3_26361443_x36.................gguCcgauucccggcgccggag..........................................................................   1
Gi5_8058424_x36cucacccCacagauccgg...............................................................................................   1
Gi3_26352083_x36..................guucgauucccggcgccggGgc.........................................................................   1
Gi2_9939771_x36.................gguucgauucccggcgccggaAc.........................................................................   1
Gi2_9949979_x35.................gguuGgauucccggcgccggagc.........................................................................   1
Gi3_26384355_x35.................ggCucgauucccggcgccg.............................................................................   1
```

```
Gi5_8068229_x35..................guucgaCucccggcgccgga...........................................................................    1
Gi1_3728247_x35..................guucgauucccggAgccgg............................................................................    1
G1a_5365600_x35..................guucgauucccggcgccgga...........................................................................    0
Gi3_26393525_x35..................guucgauucccAgcgccgg............................................................................   1
Gi5_8077714_x35..................guucUauucccggcgccgg............................................................................    1
Gi3_26381415_x35..........agauccggguucgauucccggcgccggagc.........................................................................   0
Gi3_26377950_x35..................guCcgauucccggcgccgga...........................................................................   1
Gi3_26394120_x35.............uccggguucgauucccggcgccg.............................................................................   0
Gi3_26394540_x35..................guucAauucccggcgccgg............................................................................   1
Gi5_8072429_x35..................Cuucgauucccggcgccg.............................................................................    1
Gi2_9967459_x34.................gguucgauuUccggcgccggagc.........................................................................    1
Gi2_9956477_x34.................gguucgauucccUgcgccggagc.........................................................................    1
Gi5_8085535_x34..................guCcgauucccggcgccg.............................................................................    1
Gi2_9965147_x34.................gguucgauNcccggcgccg.............................................................................    1
Gi5_8085025_x34cGcacccaacagauccgg...............................................................................................    1
Gi3_26414972_x34..................guucgauucccggUgccgga...........................................................................   1
Gi2_9958755_x34.................Aguucgauucccggcgccgga...........................................................................    1
Gi3_26413068_x34.................gguucgauucccAgcgccggag..........................................................................   1
Gi3_26424136_x33.................gguCcgauucccggcgccgga...........................................................................   1
Gi1_3738717_x33.............uccggguucgauucccggcgccgga...........................................................................    0
Gi5_8097372_x33.................gguucgauucccggcgccggGgc.........................................................................    1
Gi3_26426809_x33.................Aguucgauucccggcgcc..............................................................................   1
Gi5_8105061_x33.................gguucgGuucccggcgccggagc.........................................................................    1
Gi2_9982581_x33.................gguucgauAcccggcgccggagc.........................................................................    1
Gi5_8099451_x33..................guucgauucccggcgcUg.............................................................................    1
Gi3_26428129_x33.................gguucgauuccUggcgccgga...........................................................................   1
Gi2_9978951_x33.................gguucgauucccggcgccggCg..........................................................................    1
Gi3_26426050_x33..................guucUauucccggcgccgg............................................................................   1
Gi3_26434465_x33..................guucgauucccggcgccA.............................................................................   1
Gi2_9980106_x33.................gguucgauucccggcgUcggagc.........................................................................    1
Gi3_26442022_x33..................guucgauucccggcgccAg............................................................................   1
Gi3_26460286_x32.................gAuucgauucccggcgccgga...........................................................................   1
Gi3_26450590_x32..................guuUgauucccggcgccgga...........................................................................   1
Gi3_26446270_x32........acagauccggguucgauucccggcgccgg............................................................................   0
Gi5_8108550_x32.................gAuucgauucccggcgccggagc.........................................................................    1
Gi3_26457758_x32.................gguucgauuAccggcgccggagc.........................................................................   1
Gi3_26466494_x32.................ggGucgauucccggcgccggag..........................................................................   1
Gi3_26457918_x32.................ggGucgauucccggcgccgga...........................................................................   1
Gi1_3745344_x32..................guucgauucccgUcgccgg............................................................................    1
Gi3_26469278_x32.................gguucgauucccggcgcUgga...........................................................................   1
Gi5_8111366_x32cucacccaNcagauccgg...............................................................................................    1
Gi3_26491728_x31..................guucgauuccUggcgccgg............................................................................   1
Gi1_3754578_x31................ggguucgauucccggcgccgga...........................................................................    0
Gi5_8130315_x31..................guucgauucccggcAccg.............................................................................    1
Gi2_10011860_x31..................guucgauucccggcgccgC............................................................................   1
Gi3_26487574_x31.................gguuAgauucccggcgccggagc.........................................................................   1
Gi3_26480072_x31.................gguucgaCucccggcgccgg............................................................................   1
```

Gi3_26475019_x31..................guucgauCcccggcgccgga.. 1
Gi3_26478336_x31..................guucUauucccggcgccgga.. 1
Gi3_26477902_x31..........agauccggguucgauucccggcgccggag... 0
Gi5_8126192_x31.................gguucgaCucccggcgccggagc.. 1
Gi3_26473655_x31..................guucgauCcccggcgccgg... 1
Gi3_26472911_x31..................guuUgauucccggcgccgg... 1
Gi2_9999863_x31..................Cuucgauucccggcgccggagc.. 1
Gi5_8137855_x30..................guucgauuccUggcgccg.. 1
Gi2_10018675_x30..................guucgauucccAgcgccggagc.. 1
Gi3_26516520_x30..................guucgauucccggcgUcgga.. 1
Gi3_26512770_x30..................guucgauucccAgcgccgga.. 1
Gi3_26506200_x30.................gguucAauucccggcgccgga.. 1
Gi2_10028005_x30.................Cguucgauucccggcgcc... 1
Gi1_3758705_x30cucacccaacagauccggguucga.. 0
Gi5_8141425_x30.................gguucgauucccggcgccggC.. 1
G1a_5399946_x30.................Uguucgauucccggcgccg.. 1
Gi1_3758915_x30............auccggguucgauucccggcgccgg... 0
Gi1_3756845_x30.......aacagauccggguucgauucccggcgccgg... 0
Gi5_8148740_x29...acccaacagauccggguucgauuccc... 0
Gi3_26542980_x29.................gguucgauucccggcgAcggag... 1
Gi2_10032948_x29.........cagauccggguucgauucccggcgccggag... 0
Gi3_26527030_x29..................guucgauucccggcgcUgga.. 1
Gi5_8152887_x29..................guucgauuccUggcgccgg... 1
Gi1_3764407_x29...acccaacagauccggguucgauucccg.. 0
Gi3_26528828_x29..................guucAauucccggcgccgga.. 1
Gi3_26539645_x29...................Gucgauucccggcgccggagc.. 1
Gi2_10029874_x29.................gguCcgauucccggcgcc... 1
Gi1_3767597_x29..................guucgauuUccggcgccgg... 1
Gi1_3764610_x29..................guucgauucccggcgccggagcgA.. 1
Gi5_8144825_x29..................guucgGuucccggcgccgg... 1
Gi3_26538688_x29.................gguucgauucccggcgGcggagc.. 1
Gi1_3767858_x29..................guucgauucccggcgccCg... 1
Gi3_26537209_x29..................guucgauuNccggcgccggag... 1
Gi2_10045729_x28..................guucgauuccUggcgccggagc.. 1
Gi2_10045757_x28.................gguuAgauucccggcgccggagc.. 1
Gi3_26555953_x28..................guucgauuccUggcgccggagc.. 1
Gi3_26556541_x28..................guucgauucccggcAccgga.. 1
Gi3_26554693_x28.................gguucgauucccggcgAcgga.. 1
Gi3_26547861_x28..................guucgGuucccggcgccggag... 1
Gi5_8162657_x28.................gCuucgauucccggcgccggagc.. 1
Gi2_10055053_x28.................gguucgauucccggcgccggG.. 1
Gi2_10046429_x28.................gguucgauucAcggcgccggagc.. 1
Gi5_8160949_x28.................gguucgauNcccggcgccggag... 1
Gi1_3770940_x28.................Uguucgauucccggcgccggag... 1
Gi5_8166913_x28.................gguucgauucccggcgAc... 1
Gi3_26554441_x28cucacccaacagauccggguucgauucc.. 0
Gi5_8156945_x28..................guucgauucccggcgUcgg... 1

```
Gi5_8169947_x27...........gauccggguucgauucccggcgccgga..........................................................................    0
Gi3_26592168_x27..................guucgauucccAgcgccggagc........................................................................    1
Gi1_3776601_x27..................guucgaCucccggcgccgga..........................................................................    1
Gi2_10067744_x27..................guucgauucccggcAccgg...........................................................................    1
Gi3_26587578_x27.................gguucgauucccggcgccAga..........................................................................    1
Gi2_10059158_x27..................guucUauucccggcgccgg...........................................................................    1
Gi3_26588604_x27.................gguucgauucccggcAccggag.........................................................................    1
Gi1_3780354_x27.................gguucgauucccggcgccgU...........................................................................    1
Gi3_26577156_x27..................guucgaGucccggcgccggagc........................................................................    1
Gi3_26578533_x27cucacccaacagauccggguucgauu......................................................................................    0
Gi3_26585094_x27.................ggAucgauucccggcgccggag.........................................................................    1
Gi5_8170649_x27..................guucgauCcccggcgccg............................................................................    1
G1a_5426177_x27.................Uguucgauucccggcgccgg...........................................................................    1
Gi3_26590710_x27.......aacagauccggguucgauucccggcgccgga..........................................................................    0
Gi3_26601935_x26.................gguucgauucUcggcgccggag.........................................................................    1
Gi1_3781945_x26..................guucgauucccggcgccgA...........................................................................    1
Gi5_8186590_x26..................guucgauucccAgcgccg............................................................................    1
Gi5_8191868_x26Gucacccaacagauccgg..............................................................................................    1
Gi3_26596267_x26..................guucgauucccggcgAcgg...........................................................................    1
Gi3_26600531_x26........acagauccggguucgauucccggcgccggagc........................................................................    0
Gi3_26621123_x26...................Gucgauucccggcgccgga..........................................................................    1
Gi2_10076679_x26..................guucgGuucccggcgccgg...........................................................................    1
Gi5_8183444_x26..................guGcgauucccggcgccg............................................................................    1
Gi5_8186200_x26..................guucgauucccggUgccg............................................................................    1
Gi5_8194208_x26.................gguucgauucccggUgcc.............................................................................    1
Gi2_10097647_x25.................gguucgauucccggUgcc.............................................................................    1
Gi2_10095222_x25..................guuUgauucccggcgccggagc........................................................................    1
Gi2_10090447_x25..................guucgauuccUggcgccgg...........................................................................    1
Gi5_8203184_x25.................gguuUgauucccggcgccggagc........................................................................    1
Gi1_3790881_x25..................gAucgauucccggcgccgg...........................................................................    1
Gi3_26624893_x25..................guucgauucUcggcgccg............................................................................    1
G1a_5450987_x25.................Uguucgauucccggcgccgga..........................................................................    1
Gi3_26647618_x25.................gguuUgauucccggcgccggag.........................................................................    1
G1a_5444687_x25.................gUuucgauucccggcgccggag.........................................................................    1
Gi2_10100297_x25.......aacagauccggguucgauucccggcgccg............................................................................    0
Gi5_8207059_x25..................guucgauucccggcgccAg...........................................................................    1
Gi3_26633193_x25.................gguucgauNcccggcgcc.............................................................................    1
Gi2_10104647_x25..................guucgauucccggcgccAg...........................................................................    1
Gi3_26638018_x25.................gguucgauuccAggcgccgg...........................................................................    1
Gi3_26649193_x25.................gguucgauucccggUgccggag.........................................................................    1
Gi2_10100072_x25.................gguucgauNcccggcgccgga..........................................................................    1
Gi3_26640343_x25..................guucgauucGcggcgccggagc........................................................................    1
Gi3_26645368_x25..................guucgauucccggcAccgg...........................................................................    1
Gi5_8197809_x25.................Aguucgauucccggcgccgga..........................................................................    1
Gi1_3790131_x25..................guucgaCucccggcgccg............................................................................    1
Gi3_26621768_x25.................gguucgCuucccggcgccgga..........................................................................    1
Gi3_26674952_x24.................gguucgauucccggcgUcgga..........................................................................    1
```

```
Gi3_26663672_x24.................gguucAauucccggcgccggag.......................................................................    1
Gi3_26666672_x24..........aUauccggguucgauuccc.................................................................................    1
Gi3_26675528_x24.................gguucgauucccggcgccggUg.......................................................................    1
Gi2_10115803_x24..................guucgauucccggUgccggagc......................................................................    1
Gi1_3798686_x24.................gguucgaCucccggcgccgg.........................................................................    1
Gi3_26665520_x24.................gguucgaCucccggcgccg..........................................................................    1
Gi5_8218584_x24..................guucgauucccggcgccggagcguuucc................................................................    0
Gi3_26671712_x24.................gguuUgauucccggcgccgga........................................................................    1
Gi3_26658896_x24..................guucgauucccggcgUcgg.........................................................................    1
Gi3_26675624_x24..................guucgauCcccggcgccggagc......................................................................    1
Gi5_8228700_x23..................guucAauucccggcgccg..........................................................................    1
Gi4_499827_x23.................Uguucgauucccggcgccgg.........................................................................    1
Gi3_26687529_x23.................gguAcgauucccggcgccggag.......................................................................    1
Gi5_8231069_x23..................guucUauucccggcgccg..........................................................................    1
Gi3_26687230_x23.................ggGucgauucccggcgccg..........................................................................    1
Gi5_8229942_x23.................Cguucgauucccggcgccg..........................................................................    1
Gi3_26697833_x23.................gguucgauCcccggcgccg..........................................................................    1
Gi1_3799979_x23..................guucgCuucccggcgccgg.........................................................................    1
Gi5_8227343_x23.................gguucgauucccAgcgccggagc......................................................................    1
Gi1_3800485_x23..................guucgauucccggcgccggaU.......................................................................    1
Gi2_10137065_x23..................gCucgauucccggcgccgg.........................................................................    1
Gi4_499091_x23.................gUuucgauucccggcgccgg.........................................................................    1
Gi3_26697948_x23.................gguucgauucUcggcgccgga........................................................................    1
Gi3_26701122_x23..................guucgauucccgAcgccggagc......................................................................    1
Gi5_8222398_x23..................guucgauucccggcgccggagcA.....................................................................    1
Gi5_8228263_x23.................gguucgauuccUggcgccggagc......................................................................    1
Gi2_10128003_x23..................guucUauucccggcgccggagc......................................................................    1
Gi5_8223571_x23..................guucgauucccggcgccggagcgA....................................................................    1
Gi5_8220144_x23..................guuUgauucccggcgccgg.........................................................................    1
Gi1_3813437_x22.................gAuucgauucccggcgccgg.........................................................................    1
Gi2_10138452_x22..................guuUgauucccggcgccgg.........................................................................    1
Gi5_8240427_x22.................gguucgauNcccggcgccgg.........................................................................    1
Gi3_26727450_x22cGcacccaacagauccgg............................................................................................    1
Gi3_26709784_x22..................guAcgauucccggcgccgg.........................................................................    1
Gi1_3812227_x22..................guucgauucccggcgccgC.........................................................................    1
Gi3_26716120_x22..................gCucgauucccggcgccggag.......................................................................    1
Gi3_26725954_x22.................gguucgauucccggcgcAgga........................................................................    1
Gi3_26711566_x22..................guucgauucccAgcgccggag.......................................................................    1
Gi5_8234575_x22.................Aguucgauucccggcgcc...........................................................................    1
Gi2_10139948_x22..................guucgauucccggcgccggG........................................................................    1
Gi3_26730772_x22.................gguucgauucccUgcgccgga........................................................................    1
Gi3_26717924_x22...................uGcgauucccggcgccgga........................................................................    1
Gi3_26717066_x22.................gguucgGuucccggcgccgg.........................................................................    1
Gi3_26740848_x22.................gguCcgauucccggcgccg..........................................................................    1
Gi3_26718166_x22..................guAcgauucccggcgccgga........................................................................    1
Gi2_10147758_x22.................Cguucgauucccggcgccggag.......................................................................    1
Gi3_26731828_x22.................gguCcgauucccggcgccgg.........................................................................    1
```

```
Gi3_26728088_x22..................guucgauucccggcgcAgga..........................................................................    1
Gi3_26728792_x22.................gguNcgauucccggcgccggagc........................................................................    1
Gi2_10140696_x22..................guGcgauucccggcgccgg...........................................................................    1
Gi2_10151036_x22....................uGgauucccggcgccggagc........................................................................    1
Gi3_26727098_x22..................guucAauucccggcgccggagc........................................................................    1
Gi3_26726196_x22.................Cguucgauucccggcgccgg...........................................................................    1
Gi1_3814533_x21.................gguucgauucccggcgccggaU.........................................................................    1
Gi2_10157743_x21.................gguucgauuccAggcgccggagc........................................................................    1
Gi3_26765145_x21.........cagauccggguucgauucccggcgccggagcC.......................................................................    1
Gi5_8252838_x21.................gguCcgauucccggcgccggagc........................................................................    1
Gi5_8253741_x21..................guucgauucccggcgccgC...........................................................................    1
Gi5_8251956_x21.................ggAucgauucccggcgccggagc........................................................................    1
Gi3_26755716_x21...........Uauccggguucgauuccc...................................................................................    1
Gi2_10171771_x21.................gguucgauucccAgcgccggag.........................................................................    1
Gi5_8254014_x21.................gguucgauucccggcgccggagA........................................................................    1
Gi2_10171897_x21..................guucgauucccggcgUcggagc........................................................................    1
Gi5_8257437_x21.................gguucgauCcccggcgccggagc........................................................................    1
Gi5_8255085_x21..................guucgaCucccggcgccggag.........................................................................    1
Gi5_8255211_x21.................gguucAauucccggcgccggagc........................................................................    1
G1a_5485777_x21.................gUuucgauucccggcgccg............................................................................    1
Gi2_10163098_x21.................gguucgauucccggcgcUg............................................................................    1
Gi1_3818985_x21Gucacccaacagauccgg..............................................................................................    1
Gi5_8258970_x21.................ggGucgauucccggcgccggagc........................................................................    1
Gi3_26754267_x21..................guGcgauucccggcgccgga..........................................................................    1
Gi2_10183169_x20..................guucgauCcccggcgccgg...........................................................................    1
Gi3_26804425_x20................Ugguucgauucccggcgccggagc........................................................................    1
Gi5_8268609_x20..................guucgauucccggcgAcgg...........................................................................    1
Gi2_10175769_x20.................Aguucgauucccggcgccgg...........................................................................    1
Gi2_10186169_x20.................gguucgauucccggcgccggaCc........................................................................    1
Gi5_8276429_x20..................guucAauucccggcgccgg...........................................................................    1
Gi5_8271389_x20..................guuUgauucccggcgccg............................................................................    1
Gi2_10173549_x20..................guucgauucccggcgccggagcA.......................................................................    1
Gi3_26799545_x20.................gguucgauucccgAcgccgga..........................................................................    1
Gi3_26802185_x20.................gguucgCuucccggcgccggag.........................................................................    1
Gi3_26786165_x20...........gauccggguucgauucccggcgccgga..........................................................................    0
Gi2_10181629_x20..................guucgauCcccggcgccggagc........................................................................    1
Gi3_26801825_x20..................gAucgauucccggcgccgg...........................................................................    1
Gi2_10185989_x20.................gguucgauucccggcgccggagcU.......................................................................    1
Gi1_3825530_x20.................ggCucgauucccggcgccgg...........................................................................    1
Gi1_3821550_x20..................guucgauAcccggcgccgg...........................................................................    1
Gi2_10187089_x20.................ggGucgauucccggcgccggag.........................................................................    1
Gi3_26803925_x20.................gguucgauucccggcgccAgag.........................................................................    1
Gi1_3823770_x20.................gguucgauucccggcgAcgg...........................................................................    1
Gi5_8287020_x19..................guucgauCcccggcgccgg...........................................................................    1
Gi2_10206731_x19.................gguucgauuAccggcgccggagc........................................................................    1
Gi3_26808840_x19.................gguucgauucccggcgccAg...........................................................................    1
Gi5_8291903_x19.................gguucgauucccggcAccggagc........................................................................    1
```

Gi1_3834930_x19..................Auucgauucccggcgccggagc.. 1
Gi3_26805230_x19..................guCcgauucccggcgccggag... 1
Gi2_10199340_x19..................guucgauucccggcgAcgg... 1
Gi2_10199530_x19..................guAcgauucccggcgccggagc.. 1
Gi3_26832894_x19..................guucgauucUcggcgccgg... 1
Gi3_26820183_x19..................guucgauucccgAcgccgg... 1
Gi1_3830427_x19.ucacccaacagauccggguucga.. 0
Gi3_26819936_x19.....................cUauucccggcgccggag... 1
Gi1_3831985_x19.................gguucgauucccggcgcUgg... 1
Gi3_26828410_x19.................gguucgauucccggcgAcg.. 1
Gi3_26815452_x19.................gAuucgauucccggcgccg.. 1
Gi3_26830880_x19..................Cuucgauucccggcgccggag... 1
Gi3_26822102_x19..................guucgauucccggcgAcgga.. 1
Gi2_10206845_x19.................gguucgGuucccggcgcc... 1
Gi3_26821722_x19..................guucgauuccUggcgccgga.. 1
Gi2_10196756_x19..................guucgauucccggcgccgCagc.. 1
Gi2_10215526_x18..................guucgauucccggcgUcgg... 1
Gi2_10218298_x18..........agauccggguucgauucccggcgccggagcC... 1
Gi3_26842750_x18..................guucgauucccggUgccggagc.. 1
Gi3_26859778_x18..................guucgauucccggcgcUgg... 1
Gi3_26850778_x18.................gguucgauucccggcgccgCagc.. 1
Gi3_26843128_x18..................guGcgauucccggcgccgg... 1
Gi1_3840465_x18..................guucgauucccggcgccggG.. 1
Gi1_3842463_x18...................Gucgauucccggcgccgg... 1
Gi2_10224454_x18.................gguuUgauucccggcgccgga.. 1
Gi3_26844622_x18...................uGcgauucccggcgccggagc.. 1
Gi5_8302311_x18..................guucgauucccggcgUcg.. 1
Gi3_26873890_x18....................uGgauucccggcgccggagc.. 1
Gi2_10218010_x18.................gguucgauucccggcgcUgga.. 1
G1a_5511635_x18.................Uguucgauucccggcgcc... 1
Gi4_506352_x18.................gguucgauucccggcgccggagcC... 1
Gi3_26846368_x18.................gguuUgauucccggcgccg.. 1
Gi2_10223176_x18..................guucAauucccggcgccggagc.. 1
Gi2_10224562_x18..................guucgauucccggcgcUgg... 1
Gi3_26859400_x18.................gguucgauucccggcgccUgagc.. 1
Gi3_26866942_x18..................guucgauucccggAgccgg... 1
Gi3_26870074_x18..................guucgauuccUggcgccggag... 1
Gi3_26896844_x17..................guucgauuUccggcgccgg... 1
Gi3_26878246_x17.................gguucgUuucccggcgccggagc.. 1
Gi5_8318266_x17..........aUauccggguucgauuccc... 1
Gi5_8310769_x17......caacagauccggguucgauucccggcgccg.. 0
Gi5_8315223_x17.................gAucgauucccggcgccgg.. 1
Gi1_3854747_x17.................gguucgauucccggUgccgg... 1
Gi3_26909917_x17..................guucgauucccggcgcAgg... 1
Gi4_508099_x17..................Uuucgauucccggcgccgg... 1
Gi1_3850973_x17..........aUauccggguucgauuccc... 1
Gi2_10243414_x17..................guucgauucUcggcgccgg... 1

```
Gi5_8319694_x17.................guucgauucccggcgcUgg.........................................................................    1
Gi2_10238654_x17................ggCucgauucccggcgcc...........................................................................    1
Gi1_3850905_x17.................Auucgauucccggcgccgga........................................................................    1
Gi2_10230664_x17................gguucgauucccggcgccggUgc......................................................................    1
Gi3_26878059_x17.....................gGuucccggcgccggagc......................................................................    1
Gi5_8310820_x17.................guucgauuUccggcgccgg.........................................................................    1
Gi3_26901825_x17................gguucgauucccggAgccgga........................................................................    1
Gi1_3848525_x17.................guucgauucccggcgcGgg.........................................................................    1
Gi3_26882904_x17.................guucgauucccggcgccUga........................................................................    1
Gi3_26912263_x17...............Ugguucgauucccggcgccggag.......................................................................    1
Gi3_26877923_x17.................guucgauAcccggcgccgg.........................................................................    1
Gi2_10229746_x17................gguucgauucccggcgAcg..........................................................................    1
Gi3_26882003_x17................gguucgGuucccggcgccg..........................................................................    1
Gi2_10246083_x17.................guucgauucccggcgccAgagc......................................................................    1
Gi3_26893376_x17.................guucgauucccggcgccgAa........................................................................    1
Gi3_26903763_x17.................guucgauucccggcgccggagcgA....................................................................    1
Gi1_3848746_x17.................guucgaCucccggcgccggagc......................................................................    1
Gi3_26882054_x17................gguucgauucccggGgccggagc......................................................................    1
Gi2_10232534_x17................gguucgauucccggcAcc...........................................................................    1
Gi2_10238671_x17.................guucgauucUcggcgccggagc......................................................................    1
Gi2_10239453_x17.................Auucgauucccggcgccgga........................................................................    1
Gi1_3846808_x17.................guucgauuAccggcgccgg.........................................................................    1
Gi1_3862977_x16.................guucgauuccAggcgccgg.........................................................................    1
Gi3_26935261_x16................gguucgauucccgAcgccggag.......................................................................    1
Gi2_10263641_x16.................guAcgauucccggcgccgg.........................................................................    1
Gi3_26932605_x16................gguucgauucccggcgccggaA.......................................................................    1
Gi3_26943453_x16................gguucgauCcccggcgccgg.........................................................................    1
Gi2_10261465_x16................gguucgauucccAgcgcc...........................................................................    1
Gi3_26916285_x16................gguucgauuccGggcgccggagc......................................................................    1
Gi3_26946093_x16.................guucgauucccggcgccggC........................................................................    1
Gi3_26920877_x16.................guucgauAcccggcgccgga........................................................................    1
Gi2_10267577_x16................Cguucgauucccggcgccg..........................................................................    1
Gi3_26925405_x16................gguucgauucccggAgccg..........................................................................    1
G1a_5537341_x16................gUuucgauucccggcgccgga........................................................................    1
Gi1_3857777_x16................gguucgCuucccggcgccgg.........................................................................    1
Gi5_8333760_x16................ggCucgauucccggcgccg..........................................................................    1
Gi3_26948301_x16................gAuucgauucccggcgccgg.........................................................................    1
Gi3_26919517_x16..............cUgguucgauucccggcgccgga........................................................................    1
Gi5_8335584_x16.................guucgauucccggUgccgg.........................................................................    1
Gi1_3856657_x16................gCucgauucccggcgccgga.........................................................................    1
Gi2_10252361_x16................gguucgUuucccggcgccggagc......................................................................    1
Gi1_3861793_x16................Uguucgauucccggcgcc...........................................................................    1
Gi1_3858289_x16................gguucgGuucccggcgccgg.........................................................................    1
Gi2_10258249_x16................gguucgaCucccggcgccg..........................................................................    1
Gi5_8323808_x16.................guucgauucUcggcgccg..........................................................................    1
Gi5_8337504_x16................Aguucgauucccggcgccggag.......................................................................    1
Gi3_26942765_x16.................gAucgauucccggcgccggagc......................................................................    1
```

Gi5_8331232_x16..................Auucgauucccggcgccgga...	1
Gi3_26941533_x16..................guucgauucUcggcgccgga...	1
Gi3_26919165_x16.................ggCucgauucccggcgccgg..	1
Gi3_26921565_x16.................ggAucgauucccggcgccg...	1
Gi2_10268377_x16..................guucgauucccggcgAcggagc...	1
Gi2_10269849_x16..................guucgauucccggcgGcggagc...	1
Gi1_3858945_x16.................gguucgauucccgAcgccg...	1
Gi2_10257705_x16..................guucgauucccggcgcUggagc...	1
Gi5_8336528_x16.................Aguucgauucccggcgccgg..	1
Gi2_10256521_x16.................gguucgauucccggcgccAga...	1
Gi5_8327520_x16..................guAcgauucccggcgccg...	1
Gi1_3861985_x16..................guucgauucAcggcgccgg..	1
Gi3_26935277_x16..................gAucgauucccggcgccgga...	1
Gi2_10263561_x16.................gguucgauuccGggcgccggagc...	1
Gi1_3870362_x15.................ggGucgauucccggcgccgg..	1
Gi2_10287895_x15..................guucAauucccggcgccgg..	1
Gi2_10287565_x15..................guGcgauucccggcgccggagc...	1
Gi2_10281325_x15.................gAuucgauucccggcgccggag..	1
Gi3_26953060_x15..................guucUauucccggcgccggagc...	1
Gi2_10287145_x15..................guucgauucccggcgccgA..	1
Gi3_26983810_x15.................gguucgauucccUgcgccggag..	1
Gi3_26981320_x15.................ggCucgauucccggcgcc..	1
Gi2_10274605_x15..................guCcgauucccggcgccg...	1
Gi5_8340467_x15..................guucgauucccggcgccCg..	1
Gi5_8338577_x15..................guucgauucccAgcgccgg..	1
Gi3_26985805_x15..................guucgauuUccggcgccggag..	1
Gi3_26966755_x15.................gCuucgauucccggcgccg...	1
Gi2_10291720_x15..................guucgauAcccggcgccggagc...	1
Gi5_8349707_x15..................guucgauucccggAgccgg..	1
Gi3_26960050_x15..................guucgauuAccggcgccggagc...	1
Gi3_26977105_x15.........cagauccggguucgauucccggcgccggagc...	0
Gi3_26953450_x15..................guucgauucccggcgcccCg...	1
Gi5_8354912_x15.................gguucgauucccggcgcUggagc...	1
Gi2_10275640_x15..................gCucgauucccggcgccg...	1
Gi3_26969215_x15..................guAcgauucccggcgccggagc...	1
G1a_5552324_x15.................gUuucgauucccggcgcc..	1
Gi5_8344007_x15.................gguucgauucccggUgccggagc...	1
Gi3_26986465_x15..................guucgauucccggcgcAggagc...	1
Gi3_26957575_x15..................Auucgauucccggcgccg...	1
Gi3_26975290_x15..................guucgauucUcggcgccggagc...	1
Gi5_8339057_x15.................gguucgauucccAgcgccgga...	1
Gi2_10292530_x15..................guucgauucccggcAccggagc...	1
Gi3_26978710_x15........acagauccggguucgauucccggcgccgga...	0
Gi5_8351162_x15cCcacccaacagauccgg...	1
Gi1_3870317_x15.................gCuucgauucccggcgccgg..	1
Gi2_10293895_x15.................gguucgGuucccggcgccggag..	1
Gi3_26972620_x15.................gguucgauuccAggcgccgga...	1

```
Gi3_26954470_x15.................gguucgauuGccggcgccggagc........................................................................   1
Gi5_8346857_x15.................gguucgauNcccggcgcc.............................................................................   1
Gi2_10273030_x15..................guucgGuucccggcgccgga..........................................................................   1
G1a_5544824_x15..................Uuucgauucccggcgccggagc........................................................................   1
Gi3_26952685_x15.................gguucgauuUccggcgccgga..........................................................................   1
Gi2_10291690_x15.................ggCucgauucccggcgccggag.........................................................................   1
Gi2_10284595_x15..................guucgauucccggUgccgg...........................................................................   1
Gi3_27029341_x14...................uucgauucccggcgccgC...........................................................................   1
Gi3_27021501_x14..................guGcgauucccggcgccggag.........................................................................   1
Gi5_8372561_x14..................guGcgauucccggcgccgg...........................................................................   1
Gi3_27010399_x14.................ggNucgauucccggcgccggagc........................................................................   1
Gi2_10316595_x14.................gAuucgauucccggcgcc.............................................................................   1
Gi3_27002125_x14.................gguucgauucccggcgAcgg...........................................................................   1
Gi5_8368865_x14.................ggCucgauucccggcgcc.............................................................................   1
Gi2_10297023_x14.................gguucgauCcccggcgccgga..........................................................................   1
Gi3_27005429_x14.................gCuucgauucccggcgccgg...........................................................................   1
Gi5_8363433_x14..................Cuucgauucccggcgccgga..........................................................................   1
Gi3_27000333_x14.................gguucgauucccggcgccCgagc........................................................................   1
Gi2_10296631_x14..................guucgauuNccggcgccg............................................................................   1
Gi3_27019345_x14..................guucgauuccAggcgccgg...........................................................................   1
Gi5_8360045_x14.................gAuucgauucccggcgccg............................................................................   1
Gi3_27018169_x14..................guuUgauucccggcgccggagc........................................................................   1
Gi5_8363685_x14.................gguucgauucccggcgccggagU........................................................................   1
Gi3_27015145_x14.................gguucgauAcccggcgccggag.........................................................................   1
Gi2_10306039_x14..................guucgauucccggAgccggagc........................................................................   1
Gi5_8365253_x14.................Cguucgauucccggcgccgga..........................................................................   1
Gi3_27016139_x14.................gguuGgauucccggcgccgga..........................................................................   1
Gi2_10298059_x14..................guucgauuUccggcgccgg...........................................................................   1
Gi5_8356405_x14.................gguucgauucccAgcgccg............................................................................   1
Gi3_27000277_x14...................uucgauucccggcgccggCgc........................................................................   1
Gi5_8362635_x14.................gguucgauucccggcgccggGg.........................................................................   1
Gi5_8363755_x14.................gguucAauucccggcgccg............................................................................   1
Gi2_10313193_x14..................guucgauucccggcgccA............................................................................   1
Gi3_27012821_x14..................gCucgauucccggcgccg............................................................................   1
Gi3_27005163_x14..................guucgauucccggcgccgAagc........................................................................   1
Gi1_3877780_x14.................ggCucgauucccggcgccggagc........................................................................   1
Gi5_8358995_x14..................gCucgauucccggcgccgga..........................................................................   1
Gi5_8359135_x14.................ggCucgauucccggcgccgga..........................................................................   1
Gi3_26999605_x14.................gguucgauuccUggcgccgg...........................................................................   1
Gi3_26999759_x14.................gguucgauucccggUgccg............................................................................   1
Gi5_8360829_x14.................gguucgauucccUgcgccggagc........................................................................   1
Gi2_10313039_x14..................guucgauucccggcgcUg............................................................................   1
Gi3_27015621_x14..................guGcgauucccggcgccggagc........................................................................   1
Gi3_27004211_x14.................gguucgauucccggcgUcggag.........................................................................   1
Gi2_10316175_x14..................guucgauucccggcgccgAagc........................................................................   1
Gi1_3875372_x14.................gguucgauucccggcAccgg...........................................................................   1
Gi5_8363377_x14.................gguucgCuucccggcgccg............................................................................   1
```

```
Gi3_27001719_x14.................gAuucgauucccggcgcc.............................................................................    1
Gi3_27022649_x14..................guucgauucccggcgGcggagc........................................................................    1
Gi3_27000123_x14.................gguucgauucccggcgccgAa..........................................................................    1
Gi3_27009573_x14..................guucgauucccggcgAcggagc........................................................................    1
Gi5_8383855_x13..................guucgauucUcggcgccgg...........................................................................    1
Gi5_8373858_x13.................Cguucgauucccggcgccgg...........................................................................    1
Gi5_8373910_x13.................ggGucgauucccggcgccg............................................................................    1
Gi1_3885592_x13..................guucgauucccggcgGcgg...........................................................................    1
Gi2_10336390_x13.................gguucgauucccggcgccgCagc........................................................................    1
Gi1_3891845_x13.................gguucgauNcccggcgccggagc........................................................................    1
Gi2_10332919_x13..........aUauccggguucgauucc....................................................................................    1
Gi3_27046542_x13.................gguuGgauucccggcgccggag.........................................................................    1
Gi3_27063065_x13.................gguucgauucccggcgccgA...........................................................................    1
Gi2_10330592_x13..................guucgauucccggcgccggaAc........................................................................    1
Gi3_27072802_x13.................gguucgauucccCgcgccggagc........................................................................    1
Gi5_8389601_x13.................gguucgaCucccggcgccg............................................................................    1
Gi5_8384674_x13.................gguucgauucccggcgAcggagc........................................................................    1
Gi3_27062818_x13.................gguucgauuccUggcgcc.............................................................................    1
Gi1_3884643_x13..................guucgauucccAgcgccgga..........................................................................    1
Gi3_27045437_x13..................guucgauucccggcgccAgagc........................................................................    1
Gi2_10322298_x13.................gguucgauucccggcAccgga..........................................................................    1
Gi2_10324222_x13.................gguucgauucccggcgccAgag.........................................................................    1
Gi1_3892261_x13..................guucgauucccggcgccggU..........................................................................    1
Gi3_27062961_x13..................guucgauCcccggcgccggag.........................................................................    1
Gi3_27042083_x13.................Cguucgauucccggcgcc.............................................................................    1
Gi5_8376926_x13.................gguucgauuUccggcgccg............................................................................    1
Gi3_27048310_x13.................gguucgauuccUggcgccg............................................................................    1
Gi5_8373351_x13.................gguucgauucccggcgccggG..........................................................................    1
Gi3_27043669_x13..................guucgauuccAggcgccgga..........................................................................    1
Gi2_10328811_x13.................ggCucgauucccggcgccg............................................................................    1
Gi5_8380410_x13..................guucgauucccggcgGcgg...........................................................................    1
Gi3_27077092_x13.................gguucgauucccggcgccggaUc........................................................................    1
Gi2_10322532_x13.................gguucgauucccggcgccggagcgA......................................................................    1
Gi4_514174_x13.................Uguucgauucccggcgccggagc........................................................................    1
Gi1_3885540_x13..................guuNgauucccggcgccgg...........................................................................    1
Gi3_27057319_x13..................guucgCuucccggcgccggagc........................................................................    1
Gi3_27067030_x13.................gguuAgauucccggcgccgga..........................................................................    1
Gi5_8373884_x13.................gguucgauucccggcgccAgagc........................................................................    1
Gi3_27052301_x13.................gguucgauucccggcgcAggag.........................................................................    1
Gi2_10322402_x13..................guucgauucccggcgccggagcU.......................................................................    1
Gi3_27044670_x13..................guucgauuUccggcgccggagc........................................................................    1
Gi5_8390368_x13..................guucgauucccgUcgccgg...........................................................................    1
Gi5_8376679_x13..................guucgauuNccggcgccgga..........................................................................    1
Gi2_10338912_x13..................guucgaGucccggcgccggagc........................................................................    1
Gi2_10338106_x13.................gCuucgauucccggcgccggag.........................................................................    1
Gi2_10332256_x13.................gguAcgauucccggcgccggagc........................................................................    1
Gi5_8382022_x13..................guucgauucccggcgccggCgc........................................................................    1
```

```
Gi3_27065327_x13..................guucgGuucccggcgccg............................................................................ 1
Gi5_8385636_x13..................guAcgauucccggcgccgg........................................................................... 1
Gi3_27059906_x13.................gguucUauucccggcgccggagc........................................................................ 1
Gi4_513719_x13..................guucgauucccggcgccg............................................................................ 0
Gi3_27039015_x13.................gguucgauuUccggcgccgg........................................................................... 1
Gi2_10321648_x13..................guucgauucccggcgccggagU........................................................................ 1
Gi3_27063494_x13....................uGgauucccggcgccggag......................................................................... 1
Gi3_27050338_x13..................guucgauucccggcgccggagA........................................................................ 1
G1a_5572419_x13.................gUuucgauucccggcgccgg........................................................................... 1
Gi3_27064742_x13Gucacccaacagauccgg.............................................................................................. 1
Gi3_27052756_x13.................gguucgauucccggcgUcg............................................................................ 1
Gi3_27043162_x13.................gguucgauucccggcgcGggagc........................................................................ 1
Gi3_27117285_x12.................gguucgauucAcggcgccggag......................................................................... 1
Gi3_27113469_x12....................uUgauucccggcgccggagc........................................................................ 1
Gi2_10345018_x12..................guucgauuNccggcgccggag......................................................................... 1
Gi2_10367230_x12..................guucgauucccAgcgccgg........................................................................... 1
Gi3_27110877_x12..................guucgauucccggcgccAgag......................................................................... 1
Gi3_27097029_x12.................gguucgauucccggcAcc............................................................................. 1
Gi5_8404434_x12..................gCucgauucccggcgccggagc........................................................................ 1
Gi3_27123429_x12..................guucgauucccggcgccggagU........................................................................ 1
Gi5_8401218_x12.................gUuucgauucccggcgccggagcgu...................................................................... 1
Gi3_27087333_x12......caacagauccggguucgauucccggcgccgg........................................................................... 0
Gi1_3899774_x12...................uGcgauucccggcgccgg........................................................................... 1
Gi3_27118293_x12..................guucUauucccggcgccggag......................................................................... 1
Gi2_10349386_x12..................gAucgauucccggcgccggagc........................................................................ 1
Gi2_10359070_x12..................Cuucgauucccggcgccggag......................................................................... 1
Gi5_8400042_x12.................gAuucgauucccggcgccgga.......................................................................... 1
Gi3_27131349_x12..................guucgauuUccggcgccgga.......................................................................... 1
Gi5_8393478_x12.................gguucgCuucccggcgccggagc........................................................................ 1
Gi2_10354906_x12..................guucgauucccAgcgccg............................................................................ 1
Gi2_10356202_x12......................gGuucccggcgccggagc........................................................................ 1
Gi5_8394774_x12..................guucgauucccggcgcAg............................................................................ 1
Gi3_27127785_x12..................guucgauucccggcgccggagcU....................................................................... 1
Gi3_27088305_x12.................gguucgauucccggcgcUgg........................................................................... 1
Gi3_27122361_x12.................gguucgauuUccggcgccg............................................................................ 1
Gi2_10350382_x12..................Auucgauucccggcgccg............................................................................ 1
Gi1_3897482_x12..................guucgaGucccggcgccgg........................................................................... 1
Gi5_8394810_x12..................guucgauucccgAcgccgg........................................................................... 1
Gi5_8400306_x12..................guucgauucccggcgAcg............................................................................ 1
Gi1_3895370_x12.................gguucgauucccAgcgccgg........................................................................... 1
Gi5_8402034_x12..................guucgauucccggcgcAgg........................................................................... 1
Gi5_8407494_x12..................guucgauucccggcgUcggagc........................................................................ 1
Gi3_27112941_x12.................gguucgaAucccggcgccggagc........................................................................ 1
Gi5_8403234_x12.................gguucgauucccggcgUcggagc........................................................................ 1
Gi3_27093009_x12.................gguucgauucccggcgccggU.......................................................................... 1
Gi2_10344394_x12..................guucgauucccggcAccgga.......................................................................... 1
Gi2_10353190_x12.................gguucgauuccUggcgcc............................................................................. 1
```

```
Gi2_10354426_x12.................gguucgGuucccggcgccgga..........................................................................   1
Gi3_27126693_x12..................guucgauucccCgcgccggagc........................................................................   1
Gi2_10364326_x12.................gguucgauucccggGgccggagc........................................................................   1
Gi1_3897854_x12..................guucgauucccUgcgccgg...........................................................................   1
Gi5_8395722_x12.................gguucgauucccggcgcUgga..........................................................................   1
Gi2_10356154_x12.................ggGucgauucccggcgccgga..........................................................................   1
Gi3_27117369_x12.................gguucgauucccggcgcAg............................................................................   1
Gi5_8404902_x12.................gguucgauucccggcgccggCg.........................................................................   1
Gi5_8407074_x12.................gguucgauucccggcgcAggagc........................................................................   1
Gi5_8404566_x12.................gguucgaCucccggcgccgg...........................................................................   1
Gi3_27124785_x12.................gguucgauucccggUgccgg...........................................................................   1
Gi1_3900650_x12..................guucgauucccCgcgccgg...........................................................................   1
Gi2_10344010_x12..................guuUgauucccggcgccg............................................................................   1
Gi2_10365874_x12.................gguNcgauucccggcgccggagc........................................................................   1
Gi5_8401914_x12..................Uuucgauucccggcgccggag.........................................................................   1
Gi3_27096909_x12..................guucgauucccggcgGcgg...........................................................................   1
Gi2_10363918_x12.................gguucgCuucccggcgccggag.........................................................................   1
Gi5_8431057_x11..................guucgauucAcggcgccg............................................................................   1
Gi3_27151873_x11..................guucgauucccggcgUcggagc........................................................................   1
Gi5_8426822_x11.................gguucgauucccggcgcUg............................................................................   1
Gi5_8420024_x11.................gguucgauucccggcgccA............................................................................   1
Gi2_10388832_x11.................ggAucgauucccggcgcc.............................................................................   1
Gi1_3905453_x11.................Aguucgauucccggcgccgga..........................................................................   1
Gi2_10385290_x11..................gCucgauucccggcgccggag.........................................................................   1
Gi3_27186776_x11...................uuUgauucccggcgccggagc........................................................................   1
Gi5_8429264_x11..................guucgauuUccggcgccg............................................................................   1
Gi3_27168615_x11....................ucgauuccNggcgccggagc........................................................................   1
Gi1_3909710_x11.................Aguucgauucccggcgccggagc........................................................................   1
Gi2_10369868_x11....................ucgauuccNggcgccggagc........................................................................   1
Gi3_27167878_x11..................guucgCuucccggcgccgg...........................................................................   1
Gi2_10381913_x11..................Auucgauucccggcgccggag.........................................................................   1
Gi3_27142325_x11.....ccaacagauccggguucgauucccggcgccgg...........................................................................   0
Gi1_3909963_x11..................guucgauucccggcgccAga..........................................................................   1
Gi3_27162972_x11.............Gccggguucgauucccggcgccgga..........................................................................   1
Gi5_8414458_x11.................gguucgauuUccggcgccggagc........................................................................   1
Gi5_8418066_x11.................gCuucgauucccggcgccg............................................................................   1
Gi3_27172443_x11..................guucgauucccggcgccgCagc........................................................................   1
Gi3_27173730_x11..................guucgauucccggcgccgU...........................................................................   1
Gi3_27156955_x11cucacccaacagauccggguucgauuccc...................................................................................   0
Gi2_10391307_x11..................Cuucgauucccggcgccg............................................................................   1
Gi2_10395916_x11..................guucgauucccggcgccggagcgA......................................................................   1
Gi5_8419232_x11cucacccaacagGuccgg..............................................................................................   1
Gi3_27179593_x11..................guucgauuAccggcgccgga..........................................................................   1
Gi2_10373432_x11.................gguucgauuUccggcgcc.............................................................................   1
Gi5_8418286_x11.................gguucgauuccUggcgccg............................................................................   1
Gi2_10376468_x11..................guucgauuccUggcgccggag.........................................................................   1
Gi1_3907752_x11..................guucAauucccggcgccgga..........................................................................   1
```

Gi3_27166690_x11....................Gcgauucccggcgccgga.. 1
Gi3_27178779_x11.................gguucgauuAccggcgccggag... 1
Gi2_10392935_x11.................gguucgaAucccggcgccggagc.. 1
Gi2_10379944_x11.................ggGucgauucccggcgcc... 1
Gi3_27138805_x11.................ggGucgauucccggcgcc... 1
Gi1_3908324_x11..................guucgauucccggcgccggagcU... 1
Gi5_8422455_x11..................gAucgauucccggcgccg.. 1
Gi2_10384201_x11..................guucgauucccggcgcAgg... 1
Gi5_8428120_x11.Gcacccaacagauccggguu... 1
Gi3_27157648_x11...................uGcgauucccggcgccgg... 1
G1a_5613319_x11..................Uuucgauucccggcgccgg... 1
Gi3_27180308_x11..................guuAgauucccggcgccgg... 1
Gi3_27164699_x11.................gguucgauAcccggcgccgga.. 1
Gi3_27169121_x11.................gguucgauucccAgcgccgg... 1
Gi3_27167482_x11.................gguucgauucccAgcgccg.. 1
Gi1_3907873_x11..................guucgauucccggcgcUgga.. 1
Gi3_27183982_x11..................guucgauucccggAgccggagc.. 1
Gi2_10373322_x11..................guGcgauucccggcgccg.. 1
Gi2_10394222_x11.................gguucgaCucccggcgccgga.. 1
Gi5_8420310_x11..................guucgauucccggcgccgA... 1
Gi3_27178009_x11................Ugguucgauucccggcgccgga.. 1
Gi3_27144360_x11..........aUauccggguucgauucc.. 1
Gi5_8423874_x11..................guucgauucccgAcgccgga.. 1
Gi2_10393540_x11.................gguucgauucccggcgUc... 1
Gi2_10376281_x11..................guucgauucccggcgccggCg... 1
Gi1_3909952_x11.................gguucgauuccUggcgccgg... 1
Gi2_10397588_x11.................gguucgauCcccggcgcc... 1
Gi3_27183586_x11..................guucgauucccggcAccggag... 1
Gi2_10371342_x11..................guucgauucccggUgccgga.. 1
Gi3_27167427_x11..................guucgauucccgUcgccgga.. 1
Gi3_27177382_x11..................guucgauuNccggcgccg.. 1
Gi1_3905156_x11.................gguucgauucccggcgcAgg... 1
Gi5_8419903_x11..................guuUgauucccggcgccgga.. 1
Gi2_10405312_x10..................guucgauucccggcgcAggagc.. 1
Gi5_8452576_x10.................gguucgauucccggAgccggagc.. 1
Gi5_8447786_x10.................gguucgauucccgAcgccggagc.. 1
Gi5_8435356_x10.................gguCcgauucccggcgccg.. 1
Gi5_8434636_x10.................ggAucgauucccggcgccg.. 1
Gi5_8447406_x10..................guucgCuucccggcgccgg... 1
Gi1_3916754_x10.................gguucAauucccggcgccgg... 1
Gi3_27204082_x10..................guucgauucccggcgcUggagc.. 1
Gi3_27209772_x10.................gguucgauucccggcAccg.. 1
Gi2_10412472_x10.................gguucAauucccggcgcc... 1
Gi3_27210902_x10..................guucgauAcccggcgccggagc.. 1
Gi2_10411892_x10..................guucgGuucccggcgccg.. 1
Gi3_27239182_x10....................Gcgauucccggcgccggag... 1
Gi3_27204172_x10.................gguucAauucccggcgccg.. 1

```
Gi3_27240502_x10..................Cuucgauucccggcgccg............................................................................    1
Gi5_8447646_x10..................guucgauucAcggcgccgg...........................................................................    1
Gi3_27206282_x10.................gguucgauucccgAcgccg............................................................................    1
Gi3_27197082_x10..................guAcgauucccggcgccggag.........................................................................    1
Gi4_520909_x10..................gGucgauucccggcgccgg...........................................................................    1
Gi3_27204492_x10..................guucgauucccgAcgccgga..........................................................................    1
Gi5_8449856_x10.................gguucgGuucccggcgccg............................................................................    1
Gi2_10409622_x10.................gguucgauucccggcgccggC..........................................................................    1
Gi1_3923774_x10.................gguucgauuUccggcgccgg...........................................................................    1
Gi2_10403512_x10.................gguucgauucccggcgccgUagc........................................................................    1
Gi2_10419612_x10.....................cUauucccggcgccggagcguuu....................................................................    1
Gi1_3928104_x10cGcacccaacagauccgg..............................................................................................    1
Gi3_27191172_x10.................gguucgCuucccggcgccgg...........................................................................    1
Gi2_10412292_x10.................gguucgUuucccggcgccggag.........................................................................    1
Gi2_10397762_x10..................guucgauuUccggcgccggagc........................................................................    1
Gi5_8452316_x10..................guucgauuNccggcgccggagc........................................................................    1
Gi2_10404252_x10.................gguucgauuUccggcgccgga..........................................................................    1
Gi5_8448436_x10..................guucgauucccggcgccggGgc........................................................................    1
Gi2_10417302_x10.................gguucgauucccggcgcGggagc........................................................................    1
Gi2_10410772_x10.................gguucgauucccggcgUcgga..........................................................................    1
Gi2_10403522_x10.................gguucgCuucccggcgcc.............................................................................    1
Gi3_27217412_x10.................gguucgauuccAggcgccggag.........................................................................    1
Gi2_10401452_x10..................guucgauucccggcAccg............................................................................    1
Gi2_10403692_x10..................guucgauucccgAcgccgg...........................................................................    1
Gi3_27190712_x10.................gguucgauucAcggcgccgga..........................................................................    1
Gi3_27220262_x10..................guucgauucccggcgccC............................................................................    1
Gi3_27209272_x10..................guucgauucccggcgccggUgc........................................................................    1
Gi2_10409192_x10.................gguuUgauucccggcgcc.............................................................................    1
Gi3_27220922_x10.................gguucgauucccUgcgccg............................................................................    1
Gi1_3923174_x10..................guucgaAucccggcgccgg...........................................................................    1
Gi3_27233622_x10................gCguucgauucccggcgccggagc........................................................................    1
Gi3_27205472_x10..................guuUgauucccggcgccggag.........................................................................    1
Gi1_3921274_x10..................guCcgauucccggcgccg............................................................................    1
Gi2_10407042_x10.................gguucgauuccUggcgccggag.........................................................................    1
Gi3_27226722_x10..................guucgauucccggcAccggagc........................................................................    1
Gi2_10413142_x10..................guucgauucccggcgccggGg.........................................................................    1
Gi2_10407172_x10..................guucgauucccgAcgccggagc........................................................................    1
Gi2_10427882_x10.................gguucgauucccggcgccCgagc........................................................................    1
Gi2_10419012_x10.................gCuucgauucccggcgcc.............................................................................    1
Gi3_27192892_x10..................guucgauucccgUcgccgg...........................................................................    1
Gi3_27188212_x10..................guucgCuucccggcgccgga..........................................................................    1
Gi1_3920724_x10.......aacagauccggguucgauucccggcgccggagc........................................................................    0
Gi2_10434306_x9.................gguucgauucccggcgccggaUc........................................................................    1
Gi3_27283429_x9..................guucgauucccggAgccgga..........................................................................    1
Gi2_10438842_x9..................guucgauucccggcgUcgga..........................................................................    1
Gi5_8459643_x9.................gguucgGuucccggcgccgg...........................................................................    1
Gi5_8453325_x9cucaccUaacagauccgg..............................................................................................    1
```

```
Gi5_8476581_x9..................guucgauucccAgcgccgga..........................................................................      1
Gi1_3931120_x9....................ucgauucccggcgccggagcC.......................................................................      1
Gi5_8458428_x9.................gguucgauucccggcgccgC...........................................................................      1
Gi2_10448607_x9..................guucgauucccggcgAcg............................................................................     1
Gi1_3932884_x9..................gCucgauucccggcgccggagc........................................................................      1
Gi3_27260965_x9..................guucgauucccAgcgccg............................................................................     1
Gi3_27256195_x9.................gguucgauucccggcgccgUagc........................................................................     1
Gi3_27281638_x9.....................Ggauucccggcgccggagc........................................................................     1
Gi2_10457346_x9.................gguucgauCcccggcgccg............................................................................     1
Gi3_27290737_x9.................gguucgauuccGggcgccgga..........................................................................     1
Gi2_10447860_x9..................guucgauucAcggcgccgg...........................................................................     1
Gi5_8460930_x9..................guucgauucccggcgcUgga..........................................................................      1
Gi3_27297991_x9.................ggAucgauucccggcgccgg...........................................................................     1
Gi2_10443045_x9..................guucgauucccggcgccggagA........................................................................     1
Gi5_8470668_x9.................gguucgauucccggcgccggaCc........................................................................      1
Gi4_521253_x9 ..................guucgauucccggcgccggagc........................................................................      0
Gi2_10443882_x9.................gguucAauucccggcgccggag.........................................................................     1
Gi1_3936475_x9.................gguucgauNcccggcgccg............................................................................      1
Gi3_27271405_x9..................guucgauucccCgcgccgg...........................................................................     1
Gi3_27296749_x9................ggguucgauucccggcgccggagcC.......................................................................     1
Gi3_27268903_x9.................gguGcgauucccggcgccggagc........................................................................     1
Gi5_8472180_x9.................gguucgauuccUggcgccgg...........................................................................      1
Gi5_8469930_x9.................gguucgauucccggcAccgga..........................................................................      1
Gi2_10447644_x9.................gguucgCuucccggcgccg............................................................................     1
Gi1_3932146_x9.................gguucgauucccggcgccggagcgA......................................................................      1
Gi3_27247375_x9.................gguucgauucccggcCccggagc........................................................................     1
Gi1_3936016_x9..................guuUgauucccggcgccgga..........................................................................      1
Gi3_27245107_x9.................ggGucgauucccggcgccgg...........................................................................     1
Gi5_8467230_x9.................gguucgCuucccggcgccggag.........................................................................      1
G1a_5648809_x9...acccaacagauccggguucgauuccc...................................................................................      0
Gi2_10446852_x9.................gguucgauucccggUgccg............................................................................     1
Gi5_8467374_x9..................guucgauucccCgcgccgg...........................................................................      1
Gi5_8458752_x9.................gguucgauucccggcgccAga..........................................................................      1
Gi3_27263017_x9..................guucgauucccCgcgccgga..........................................................................     1
Gi2_10450506_x9.................Cguucgauucccggcgccgga..........................................................................     1
Gi2_10434270_x9.................gguucgauucccCgcgccggagc........................................................................     1
Gi5_8454351_x9.................ggGucgauucccggcgccgg...........................................................................      1
Gi3_27302113_x9.................gguuAgauucccggcgccggag.........................................................................     1
Gi5_8463774_x9......................gGuucccggcgccggagc........................................................................      1
Gi2_10458642_x9.................gguucgauucccggAgccg............................................................................     1
Gi2_10432020_x9..................guucAauucccggcgccgga..........................................................................     1
Gi3_27249112_x9..................guGcgauucccggcgccg............................................................................     1
Gi5_8469489_x9..................guucgauAcccggcgccg............................................................................      1
Gi5_8461218_x9.................ggGucgauucccggcgcc.............................................................................      1
Gi3_27288757_x9..................guucAauucccggcgccggag.........................................................................     1
Gi5_8454072_x9..........Ggauccggguucgauuccc...................................................................................      1
G1a_5644426_x9..................guucgauucccggcgccggag.........................................................................      0
```

```
Gi5_8461515_x9..................guucUauucccggcgccgga..........................................................................     1
Gi2_10437555_x9..................guucgauucccggcgccCg...........................................................................     1
Gi5_8461047_x9.................Uguucgauucccggcgccggagcgu......................................................................     1
Gi5_8466123_x9..................guucgauucccggAgccg............................................................................     1
Gi5_8464125_x9...................Gucgauucccggcgccgg...........................................................................     1
Gi1_3947309_x8..................guucgauucccggcgccggGgc........................................................................     1
Gi3_27320697_x8..................guucgauucccggGgccgg...........................................................................     1
Gi4_525296_x8 .................gUuucgauucccggcgccggagc........................................................................     1
Gi5_8497897_x8..................guucgGuucccggcgccggagc........................................................................     1
Gi3_27331577_x8.................gguucgCuucccggcgcc.............................................................................     1
Gi3_27360993_x8..................guucgauucccggcgccggUg.........................................................................     1
Gi2_10487602_x8..................guucgauuccAggcgccgg...........................................................................     1
Gi2_10483890_x8.................ggCucgauucccggcgccgga..........................................................................     1
Gi4_526408_x8 .................gguucgauucccggcgccgga..........................................................................     0
Gi5_8493065_x8.................gguucgauucUcggcgccggagc........................................................................     1
Gi3_27359665_x8.................gguucgauuccGggcgccg............................................................................     1
Gi5_8496041_x8.................gguucgauucccggcgAcgga..........................................................................     1
Gi2_10487410_x8.................gguucgauucUcggcgcc.............................................................................     1
Gi5_8490161_x8.................ggGucgauucccggcgccggag.........................................................................     1
Gi5_8481609_x8........................................Uuuucccgccuuccuucgucu...................................................     1
Gi3_27362081_x8..................guucgauucccggcgcGgga..........................................................................     1
Gi2_10466082_x8.................gguucgauucccggcgUcg............................................................................     1
Gi3_27374497_x8..................gAucgauucccggcgccggag.........................................................................     1
Gi3_27328017_x8..................guucgaGucccggcgccgga..........................................................................     1
Gi3_27328089_x8..................guucgauucAcggcgccgg...........................................................................     1
Gi1_3942421_x8.................gguucgauucUcggcgccgg...........................................................................     1
Gi2_10487194_x8...................Gucgauucccggcgccgg...........................................................................     1
Gi3_27342481_x8.................gguucgauuccAggcgccg............................................................................     1
Gi5_8488025_x8.................ggAucgauucccggcgccgga..........................................................................     1
Gi2_10472418_x8.................gguCcgauucccggcgccggag.........................................................................     1
Gi3_27346385_x8..................guucgauucccggcgcccCgagc.......................................................................     1
Gi2_10492594_x8..................guucgauCcccggcgccgga..........................................................................     1
Gi1_3955053_x8.................gguucgauucccggcgccggagcA.......................................................................     1
Gi5_8496129_x8.................ggCucgauucccggcgccgg...........................................................................     1
Gi2_10495786_x8..................guucgauucAcggcgccggagc........................................................................     1
Gi2_10494178_x8.................gguucgauucccggcAccggag.........................................................................     1
Gi2_10479218_x8.................gguucgauucccggcgAcgga..........................................................................     1
Gi2_10479466_x8..................guucgauucccCgcgccggagc........................................................................     1
Gi5_8493233_x8.................gCuucgauucccggcgccgg...........................................................................     1
Gi2_10487810_x8..................guCcgauucccggcgccggag.........................................................................     1
Gi3_27356329_x8......................gauucccAgcgccggagc........................................................................     1
Gi5_8479537_x8.................gguucgauucccggAgccgga..........................................................................     1
Gi3_27359369_x8..................guucgauucccggcgccggaAc........................................................................     1
Gi1_3943421_x8.................gguucgauucccggcgccggU..........................................................................     1
Gi3_27363793_x8..................guucgauucccggcgGcgga..........................................................................     1
Gi2_10486802_x8....................ucgauucUcggcgccggagc........................................................................     1
Gi3_27364521_x8.................gguucgauucccggAgccggag.........................................................................     1
```

```
Gi3_27335497_x8.....................cgUuucccggcgccggagc.......................................................................    1
Gi3_27320833_x8.....................cgauucccggcgccggCgc.......................................................................    1
Gi5_8486097_x8..................guuUgauucccggcgccggagc.......................................................................    1
Gi2_10494842_x8....................ucgauucccggcgccggGgc.......................................................................    1
Gi5_8481825_x8.................ggCucgauucccggcgccggag........................................................................    1
Gi2_10472842_x8..................guucgauuNccggcgccgga.........................................................................    1
Gi3_27348209_x8.................gguuUgauucccggcgcc............................................................................    1
Gi2_10486738_x8.................gguucgauucccggcgGcggagc.......................................................................    1
Gi1_3946141_x8.................gguCcgauucccggcgccgg..........................................................................    1
Gi2_10476746_x8.................gguucgauNcccggcgccgg..........................................................................    1
Gi5_8501385_x8.................gguucgauuccAggcgccggagc.......................................................................    1
Gi2_10478338_x8.................gguucgauucccggAgcc............................................................................    1
Gi1_3947829_x8.................gguuUgauucccggcgccgg..........................................................................    1
Gi2_10481186_x8.................gguucgauucUcggcgccggag........................................................................    1
Gi5_8494609_x8.................gguucgauucccggcgUc............................................................................    1
Gi5_8493593_x8.................gguucgauucccggcgUcgga.........................................................................    1
Gi2_10477890_x8.................gguuUgauucccggcgccg...........................................................................    1
Gi3_27310729_x8.................gguucgauucccggcgccgCag........................................................................    1
Gi2_10467834_x8..................guucgauucccggcCccggagc.......................................................................    1
Gi2_10475850_x8.................gguucAauucccggcgccgga.........................................................................    1
Gi2_10497218_x8..................guucgauucccAgcgccgga.........................................................................    1
Gi5_8476993_x8..........agauccggNuucgauuccc..................................................................................    1
Gi5_8479161_x8cucGcccaacagauccgg.............................................................................................    1
Gi5_8486489_x8..................gGucgauucccggcgccggag........................................................................    1
Gi2_10469946_x8..................guucgUuucccggcgccgg..........................................................................    1
Gi2_10464722_x8.................gguucgGuucccggcgccg...........................................................................    1
Gi2_10482586_x8..................guucgauucccggcgccUgagc.......................................................................    1
Gi2_10466818_x8..................Cuucgauucccggcgccgga.........................................................................    1
Gi2_10480930_x8.................gguucgCuucccggcgccgga.........................................................................    1
Gi5_8487297_x8..................guucgauuccAggcgccg...........................................................................    1
Gi2_10480546_x8.................gguucgauucccggUgccgga.........................................................................    1
Gi1_3943365_x8.................gguucgauucccggcgccggagU.......................................................................    1
Gi2_10474298_x8.................gguucgauucccggcgccggagG.......................................................................    1
Gi2_10469098_x8.................gguucgauucccggcgccA...........................................................................    1
Gi5_8487249_x8..................guucgauucccggcgccggG.........................................................................    1
Gi2_10467634_x8..................guucgauucccgUcgccggagc.......................................................................    1
Gi2_10472362_x8.................gguucgauucccAgcgccgga.........................................................................    1
Gi5_8493825_x8.................ggGucgauucccggcgccgga.........................................................................    1
Gi2_10486098_x8.................gguucgaCucccggcgccgg..........................................................................    1
Gi1_3945037_x8.................gguucgauucccggcgccggG.........................................................................    1
Gi5_8498865_x8..................guucgUuucccggcgccg...........................................................................    1
Gi2_10488730_x8.................gguucgauucccggcAccgg..........................................................................    1
Gi5_8482345_x8.................gAuucgauucccggcgcc............................................................................    1
Gi5_8477785_x8..................guCcgauucccggcgccgga.........................................................................    1
Gi2_10493754_x8..................guucgauuAccggcgccggagc.......................................................................    1
G1a_5671057_x8.................Aguucgauucccggcgccggagc.......................................................................    1
Gi1_3943805_x8..................guucgUuucccggcgccgg..........................................................................    1
```

```
Gi2_10471362_x8.......aacagauccggguucgauucccggcgccgg..........................................................................    0
Gi3_27324385_x8...................Gucgauucccggcgccgg..........................................................................    1
Gi5_8523577_x7..................guucgauucccggcgccUg..........................................................................    1
Gi2_10523505_x7...................uGcgauucccggcgccgg..........................................................................    1
Gi3_27407679_x7.................gguucgauucccggcgcGggag........................................................................    1
Gi3_27409758_x7....................ucgGuucccggcgccggagc.......................................................................    1
Gi5_8527161_x7................Ugguucgauucccggcgccggagc.......................................................................    1
Gi2_10524779_x7.................gguucgauucccggUgccggag........................................................................    1
Gi1_3957976_x7..................Cuucgauucccggcgccgga.........................................................................    1
Gi2_10517688_x7.................gguucgauCcccggcgccggag........................................................................    1
Gi3_27441874_x7..................guucAauucccggcgccg...........................................................................    1
Gi2_10525038_x7..................guucUauucccggcgccg...........................................................................    1
Gi2_10501994_x7..................guucgCuucccggcgccgg..........................................................................    1
Gi5_8514904_x7..................Auucgauucccggcgccggagc.......................................................................    1
Gi5_8506868_x7.................gguucgCuucccggcgccgga.........................................................................    1
Gi5_8521162_x7.................gguucgauucccggcgccggUgc.......................................................................    1
Gi3_27427111_x7..................guucgauucccggcgcGggagc.......................................................................    1
Gi2_10501630_x7..................guucgauucccggUgccggag........................................................................    1
Gi3_27446081_x7.................gguucgauuccAggcgcc............................................................................    1
Gi3_27426285_x7..................guucgauuccUggcgccg...........................................................................    1
Gi2_10535055_x7....................ucgauuUccggcgccggagc.......................................................................    1
Gi3_27420545_x7.................gguucgauGcccggcgccggagc.......................................................................    1
Gi1_3962561_x7.................ggAucgauucccggcgccgg..........................................................................    1
Gi2_10499642_x7...................Gucgauucccggcgccggagc.......................................................................    1
Gi1_3963688_x7..................guuAgauucccggcgccgg..........................................................................    1
Gi3_27444233_x7..................guucgauucAcggcgccgga.........................................................................    1
Gi3_27405824_x7.................gguucgauucccggcgGcgga.........................................................................    1
Gi5_8506252_x7.................gguucgauucccggcgccgAagc.......................................................................    1
Gi1_3967076_x7.................gguucgauucccggcgccggGgc.......................................................................    1
Gi1_3958641_x7.................gguucgauucccggcAccggagc.......................................................................    1
G1a_5688204_x7..................gGucgauucccggcgccggagc.......................................................................    1
Gi5_8515569_x7..................guucgauucccggUgccgga.........................................................................    1
Gi3_27386308_x7.................gguucgauucccggcUccggagc.......................................................................    1
G1a_5682142_x7.................gguucgauNcccggcgccggagc.......................................................................    1
Gi3_27414959_x7.................gguucgCuucccggcgccg...........................................................................    1
Gi3_27423527_x7.....................cgauucccNgcgccggagc.......................................................................    1
Gi3_27447873_x7.................gguucgauucccggcgccgCa.........................................................................    1
Gi5_8529632_x7.................ggAucgauucccggcgccgg..........................................................................    1
Gi5_8507155_x7....................Gcgauucccggcgccggagc.......................................................................    1
Gi5_8514484_x7.................gguucgauucccggcAccg...........................................................................    1
Gi3_27445787_x7.................gguucgauucccggcgcUg...........................................................................    1
Gi5_8521729_x7.................gCuucgauucccggcgccggag........................................................................    1
Gi2_10499586_x7.................gguucgauucccggcgUcggag........................................................................    1
Gi3_27405733_x7..................guucgaGucccggcgccgg..........................................................................    1
Gi2_10525598_x7.................gguucgauucccggcgccC...........................................................................    1
Gi5_8526216_x7cucacUcaacagauccgg.............................................................................................    1
Gi2_10522161_x7.................gguCcgauucccggcgccg...........................................................................    1
```

```
Gi2_10509813_x7..................guuUgauucccggcgccgga..........................................................................   1
Gi3_27442532_x7.................gguucgauucAcggcgccg............................................................................   1
Gi2_10534873_x7..................guucCauucccggcgccg............................................................................   1
Gi2_10516162_x7.................Cguucgauucccggcgccgg...........................................................................   1
Gi3_27396451_x7..................guucgCuucccggcgccg............................................................................   1
Gi1_3962540_x7.................gguAcgauucccggcgccgg...........................................................................   1
Gi3_27425508_x7..................guCcgauucccggcgccg............................................................................   1
Gi5_8521813_x7.................gguucgauCcccggcgccg............................................................................   1
Gi5_8519272_x7.................gguCcgauucccggcgccgg...........................................................................   1
Gi1_3973922_x7...................uuUgauucccggcgccgg...........................................................................   1
Gi2_10536210_x7.................gguucgauucccggUgccgg...........................................................................   1
Gi2_10507881_x7..................guCcgauucccggcgccgga..........................................................................   1
Gi3_27447943_x7...................uucgauucNcggcgccggagc........................................................................   1
Gi5_8505811_x7..................guucgauucccgAcgccg............................................................................   1
Gi3_27453277_x7.................gguucgauucUcggcgccgg...........................................................................   1
Gi5_8511859_x7.................gguucgauucccggcgAcg............................................................................   1
Gi5_8516486_x7..................guucgauucccggcgccggC..........................................................................   1
Gi1_3961980_x7.................gguuGgauucccggcgccgg...........................................................................   1
Gi1_3961497_x7.................gUuucgauucccggcgcc.............................................................................   1
Gi3_27381107_x7.................gguucgauucccggcgUcgg...........................................................................   1
Gi3_27382605_x7...................uucgauucccggcgccAgagc........................................................................   1
Gi5_8521939_x7..................guucgauucccggcgGcg............................................................................   1
Gi2_10514692_x7..................guuAgauucccggcgccgg...........................................................................   1
Gi3_27451499_x7..................guucgauucccggUgccg............................................................................   1
Gi3_27446438_x7..................guucgauuAccggcgccgg...........................................................................   1
Gi3_27379581_x7.................gguucgauucccggcgUc.............................................................................   1
Gi2_10517387_x7.................ggGucgauucccggcgccg............................................................................   1
Gi3_27449252_x7..................guucgauucccggcgccggaCc........................................................................   1
Gi2_10525304_x7.................gguucgauucccggcgGcg............................................................................   1
Gi5_8512132_x7..................guuAgauucccggcgccgg...........................................................................   1
Gi3_27451051_x7.................gguCcgauucccggcgcc.............................................................................   1
Gi3_27381044_x7..................guucgauucccggcgcAggag.........................................................................   1
Gi1_3967965_x7..................guucgauucccggcgccUg...........................................................................   1
Gi2_10529371_x7.................gguucgaCucccggcgccggag.........................................................................   1
Gi1_3960671_x7..................guucCauucccggcgccgg...........................................................................   1
Gi3_27448888_x7.................gguucgauucccggcgccggagG........................................................................   1
Gi3_27434055_x7.................gguucgauucccggcgccggagcg.......................................................................   0
Gi2_10509617_x7..................guucgauuccUggcgccgga..........................................................................   1
Gi3_27424451_x7cucacccaNcagauccgg..............................................................................................   1
Gi3_27418193_x7.................Nguucgauucccggcgccggagc........................................................................   1
Gi5_8523892_x7..................guucgauucccggcgccAgagc........................................................................   1
Gi2_10529735_x7..................guucUauucccggcgccggag.........................................................................   1
Gi5_8528449_x7.................gguucgauucccAgcgcc.............................................................................   1
Gi3_27407525_x7..................guucgauucccggcgccggCg.........................................................................   1
Gi3_27425942_x7..................guucgauucccggcgccggagcgC......................................................................   1
Gi3_27448104_x7....................ucgaCucccggcgccggagc........................................................................   1
Gi5_8507673_x7..................guucAauucccggcgccggagc........................................................................   1
```

```
Gi5_8530185_x7.................Uguucgauucccggcgccggagcguu.....................................................................      1
Gi1_3973369_x7.................gguucgauucccggcgccggCgc........................................................................      1
Gi2_10522343_x7...................uGcgauucccggcgccggagc........................................................................      1
Gi3_27447810_x7.................gguucgauucUcggcgccg............................................................................      1
Gi3_27386875_x7..................guucgauucccggcgccggaA.........................................................................      1
Gi2_10512207_x7..................guucgauucccggcgccCgagc........................................................................      1
Gi5_8508282_x7.................gguucgauucccgAcgccgga..........................................................................      1
Gi5_8504460_x7.................gguuUgauucccggcgccg............................................................................      1
G1a_5701007_x7.................gguucgauucccggUgcc.............................................................................      1
Gi2_10503373_x7....................ucgaCucccggcgccggagc........................................................................      1
Gi3_27382640_x7..................guucgauucccggcgcUg............................................................................      1
Gi5_8512580_x7.................gguucgGuucccggcgccggag.........................................................................      1
Gi2_10508350_x7.................gguucgauucccUgcgccggag.........................................................................      1
Gi2_10515833_x7.................gguucgauuccUggcgccg............................................................................      1
Gi3_27423296_x7..................guuUgauucccggcgccg............................................................................      1
Gi3_27411417_x7..................guucgauucAcggcgccggagc........................................................................      1
Gi3_27416485_x7.................gguucgauucccggAgcc.............................................................................      1
Gi3_27394498_x7.................gguucgauucccggAgccgg...........................................................................      1
Gi1_3967475_x7.................ggCucgauucccggcgccgga..........................................................................      1
Gi5_8503165_x7..................guucgauuAccggcgccg............................................................................      1
Gi5_8529464_x7.................Uguucgauucccggcgccggagcguuu....................................................................      1
Gi5_8503200_x7..................guucgCuucccggcgccg............................................................................      1
Gi3_27445290_x7..................guucgauucccggcgccCga..........................................................................      1
Gi2_10568933_x6.................gguucgauucccggGgcc.............................................................................      1
Gi2_10549907_x6..................guucgauucccgAcgccgga..........................................................................      1
Gi5_8550985_x6.................gguucgauucccggcgcUgg...........................................................................      1
Gi3_27511798_x6..................guucgauucccggcgUcggag.........................................................................      1
Gi5_8541073_x6..................guucgauucccgUcgccg............................................................................      1
Gi2_10569353_x6....cUcaacagauccggguuc..........................................................................................      1
Gi2_10583597_x6.................gguCcgauucccggcgccgga..........................................................................      1
Gi3_27511150_x6.................gguuGgauucccggcgccg............................................................................      1
Gi1_3988279_x6...................Gucgauucccggcgccggagc........................................................................      1
Gi5_8558725_x6.................gguucgauucccUgcgccgg...........................................................................      1
Gi5_8532871_x6.................gguucgauucccggcgcAg............................................................................      1
Gi1_3992917_x6....................ucgauuAccggcgccggagc........................................................................      1
Gi3_27459346_x6.................gguucgauuccGggcgccggag.........................................................................      1
Gi3_27520444_x6......................gaCucccggcgccggagc........................................................................      1
Gi2_10587971_x6.................gguucgauucAcggcgccggag.........................................................................      1
Gi2_10574765_x6.................gguucgauucccggcgcA.............................................................................      1
Gi1_3981253_x6..................guuGgauucccggcgccgg...........................................................................      1
Gi5_8554057_x6.................gguGcgauucccggcgccg............................................................................      1
Gi2_10551425_x6.....................cgauucccggcgccggagc........................................................................      0
Gi3_27521662_x6.................gguucgGuucccggcgcc.............................................................................      1
Gi2_10574453_x6.................gguCcgauucccggcgccgg...........................................................................      1
Gi2_10558439_x6.....................cgauucccggcgccggagcguuG....................................................................      1
Gi3_27542590_x6....................ucgauucccggcgccggGgc........................................................................      1
Gi3_27525220_x6...................uucgauucccggcgccggC..........................................................................      1
```

```
Gi5_8535757_x6.................Cguucgauucccggcgccggag.........................................................................    1
Gi5_8545987_x6.................gguAcgauucccggcgccggagc........................................................................    1
Gi5_8551537_x6.................gguucgauucccAgcgccgg...........................................................................    1
Gi2_10567661_x6..................guucgauCcccggcgccg............................................................................    1
Gi1_3993187_x6..................gCucgauucccggcgccg............................................................................    1
Gi1_3986059_x6..................guucgauucccggcgccgU...........................................................................    1
Gi3_27501688_x6....................uGgauucccggcgccgga..........................................................................    1
Gi3_27507706_x6..................guAcgauucccggcgccg............................................................................    1
Gi3_27484174_x6.................gguucgauucccggcgccU............................................................................    1
Gi1_3980503_x6.................gguucgauCcccggcgccggagc........................................................................    1
Gi5_8549311_x6.................gguuAgauucccggcgccggagc........................................................................    1
Gi1_3980479_x6..................guucgauucccggcgccA............................................................................    1
Gi2_10585691_x6.................gguucgauucccggcgcAgga..........................................................................    1
Gi1_3992587_x6..................guAcgauucccggcgccgga..........................................................................    1
Gi5_8533621_x6..................guucgauCcccggcgccgga..........................................................................    1
Gi5_8554297_x6.................gguucgauucccggcgccggaAc........................................................................    1
Gi1_3985861_x6..................guCcgauucccggcgccggagc........................................................................    1
Gi5_8536891_x6.................gguucAauucccggcgccgg...........................................................................    1
Gi1_3992161_x6..................guucgauucccggcAccgga..........................................................................    1
Gi3_27495448_x6cCcacccaacagauccgg..............................................................................................    1
Gi1_3984025_x6..................guucgauuNccggcgccgga..........................................................................    1
Gi5_8550685_x6.............ucUggguucgauucccggc................................................................................    1
Gi5_8542951_x6cucacccaacagaCccgg..............................................................................................    1
Gi1_3984469_x6..................guuUgauucccggcgccggagc........................................................................    1
Gi2_10566755_x6..................guucgauucccUgcgccggagc........................................................................    1
Gi2_10575779_x6..................gAucgauucccggcgccgga..........................................................................    1
Gi3_27488302_x6....................ucgauucccggcgccggCgc........................................................................    1
Gi1_3986503_x6.................Aguucgauucccggcgccg............................................................................    1
Gi3_27527164_x6.................gguucgauucccgAcgccgg...........................................................................    1
Gi2_10576583_x6.................ggAucgauucccggcgccggag.........................................................................    1
Gi3_27510196_x6.................gguucgauucccAgcgcc.............................................................................    1
Gi5_8545291_x6Uucacccaacagauccgg..............................................................................................    1
Gi2_10569701_x6.................gguucgauucccggcAccg............................................................................    1
Gi3_27477280_x6................Ugguucgauucccggcgccg............................................................................    1
Gi2_10556015_x6..................guucgauucccggcAccggag.........................................................................    1
Gi2_10578713_x6.........Gagauccggguucgauucc....................................................................................    1
Gi3_27464092_x6..................guucgauucccggcgcAg............................................................................    1
Gi3_27541582_x6...................uucgauucNcggcgccgga..........................................................................    1
Gi3_27536362_x6..................guucgauucccggcgccUg...........................................................................    1
Gi3_27521374_x6..................guucgauucccggcCccgga..........................................................................    1
Gi3_27470788_x6.................gguucgaGucccggcgccggagc........................................................................    1
Gi3_27525706_x6..................guucgauucccUgcgccgg...........................................................................    1
Gi3_27518572_x6.................gguucgauuAccggcgccgga..........................................................................    1
Gi1_3988993_x6.................gguucgauucccggcgccCg...........................................................................    1
Gi5_8531353_x6.................gguucgauucAcggcgccggagc........................................................................    1
Gi5_8543503_x6.................gguucgauAcccggcgccggagc........................................................................    1
Gi3_27509530_x6........acagauccggguucgauucccggcgccggagcC.......................................................................    1
```

Gi3_27482182_x6.................gguucgauucccggcgccgU...	1
Gi3_27463690_x6.................gguucgaAucccggcgccgga..	1
Gi5_8532547_x6.................gguuUgauucccggcgccggagcguu...	1
Gi5_8548093_x6..................guucgauucccggcAccgga..	1
Gi2_10561487_x6..................guucUauucccggcgccgga..	1
Gi3_27487786_x6.................gguucUauucccggcgccgga..	1
Gi5_8543557_x6cuUacccaacagauccgg..	1
Gi2_10544189_x6.................gguuGgauucccggcgccg..	1
Gi5_8555773_x6.................gguucgauucccggcAccggag...	1
Gi3_27472150_x6..................guucgauucccgUcgccggagc..	1
Gi3_27548074_x6.....................cgauucccggcgccAgagc..	1
Gi2_10580399_x6..................guucgauucccggcgcAg..	1
Gi2_10562117_x6.................gguucgauucccAgcgccg..	1
Gi1_3991867_x6..................guucgauucccggUgccgga..	1
Gi5_8562331_x6..................guucgauucccCgcgccg..	1
Gi3_27509992_x6..................guucgauuccAggcgccggagc..	1
Gi1_3991351_x6......................gauucccggcgccggagc..	0
Gi5_8533807_x6.................gguucgauucccggcgccgAa..	1
Gi2_10580027_x6....................Gcgauucccggcgccggag...	1
Gi2_10567193_x6.................gguucgauucccggcgAcggag...	1
Gi2_10574429_x6....................Ccgauucccggcgccggagc..	1
Gi5_8561839_x6.................gCuucgauucccggcgccgga..	1
Gi1_3985681_x6..................guNcgauucccggcgccgg...	1
Gi3_27511738_x6..................guucgauucccggcAccg..	1
Gi2_10590599_x6.................gguucgauucccUgcgccg..	1
Gi3_27501868_x6.................gguucgauucccggGgccggag...	1
Gi3_27465052_x6..................guucgauucccggcgccggU..	1
Gi5_8560213_x6.................gUuucgauucccggcgccggagcguuu..	1
Gi3_27535378_x6.................gguuGgauucccggcgccgg...	1
Gi1_3993481_x6..................guucgauucccggcgUcg..	1
Gi2_10546109_x6.................gguucgauucccggcgccUgagc..	1
Gi2_10548545_x6..................guucgauuccAggcgccggagc..	1
Gi5_8547775_x6..................guucgauAcccggcgccgg...	1
Gi5_8548675_x6..................guucgauucccggGgccg..	1
Gi3_27478264_x6..................guucUauucccggcgccg..	1
Gi3_27531694_x6..................guuGgauucccggcgccggagc..	1
Gi2_10549547_x6.................gguucgauucccggcgccggaA...	1
Gi3_27484798_x6...................uCcgauucccggcgccgga..	1
Gi5_8562223_x6..................guucgaGucccggcgccgg...	1
Gi1_3980713_x6.Gcacccaacagauccgggu..	1
Gi5_8556733_x6.................gguucgauuccUggcgccgga..	1
Gi2_10578299_x6.....................cUauucccggcgccggagcguuuc...	1
Gi5_8547289_x6.................gguucgauucccggAgccg..	1
Gi3_27469624_x6.........Gagauccggguucgauuccc...	1
Gi2_10558097_x6.................gguucgauucccggcgcUggag...	1
Gi5_8563561_x6.................gguuUgauucccggcgccggag...	1
Gi1_3975169_x6..................Cuucgauucccggcgccg..	1

```
Gi3_27515596_x6......................gauucccggGgccggagc.......................................................................    1
Gi2_10589573_x6..................guucgauucccgUcgccgg..........................................................................    1
Gi1_3975235_x6..................guucgauucccggcgccggCgc.......................................................................    1
Gi5_8558587_x6cucacccaCcagauccgg.............................................................................................    1
Gi5_8544295_x6....ccUaacagauccggguuc.........................................................................................    1
Gi2_10569563_x6..................guucgauucccggcgccggC.........................................................................    1
Gi2_10585175_x6.................gguucgauucccggcgcU............................................................................    1
Gi1_3982381_x6..................guucgauuccUggcgccg...........................................................................    1
Gi5_8548669_x6.................gguuUgauucccggcgccgga.........................................................................    1
Gi2_10565009_x6.................gguucgauucccUgcgccgga.........................................................................    1
Gi5_8534767_x6..................guucgUuucccggcgccgg..........................................................................    1
G1a_5702458_x6.................Cguucgauucccggcgccggag........................................................................    1
G1a_5719582_x6..................Uuucgauucccggcgccg...........................................................................    1
Gi2_10576511_x6.................gCuucgauucccggcgccgga.........................................................................    1
Gi2_10563647_x6.................gguuUgauucccggcgccggag........................................................................    1
Gi1_3978103_x6.................Cguucgauucccggcgccggagc.......................................................................    1
Gi5_8531137_x6.................gguucgGuucccggcgccgga.........................................................................    1
Gi1_3990205_x6.................Aguucgauucccggcgccggag........................................................................    1
Gi4_530649_x6 ..................Uuucgauucccggcgccgga.........................................................................    1
Gi5_8550865_x6..................guucgaGucccggcgccg...........................................................................    1
Gi3_27509242_x6.......aacagauccggguucgauucccggcgccggagc.......................................................................    0
Gi2_10545881_x6..................guucgauucccggcgUcg...........................................................................    1
Gi5_8555503_x6..................guucgauucccAgcgccggagc.......................................................................    1
Gi2_10542875_x6..................guucgauucccggcgcUgga.........................................................................    1
Gi2_10630340_x5.................gguuGgauucccggcgccggag........................................................................    1
Gi2_10622855_x5.................gguucgauucccggcgccgCag........................................................................    1
Gi2_10599780_x5.................gguucgauucccggcgcAggag........................................................................    1
Gi1_4014305_x5..................guucgauuGccggcgccgg..........................................................................    1
Gi2_10598525_x5..................guucgauucccggcgccggUgc.......................................................................    1
Gi3_27655387_x5......Gaacagauccggguucgauuccc..................................................................................    1
Gi2_10602855_x5..................guuAgauucccggcgccggagc.......................................................................    1
Gi2_10622310_x5.................gCuucgauucccggcgccg...........................................................................    1
Gi3_27599387_x5...................uucgauucccggcgccggG.........................................................................    1
G1a_5735567_x5..................guCcgauucccggcgccgg..........................................................................    1
Gi5_8597282_x5cucacccaacagCuccgg.............................................................................................    1
Gi2_10594165_x5.................gguucgauuccAggcgcc............................................................................    1
Gi2_10645415_x5..................guucAauucccggcgccg...........................................................................    1
Gi5_8594042_x5.................gguucgauucccggUgccgga.........................................................................    1
Gi3_27611272_x5..................guucgUuucccggcgccgga.........................................................................    1
Gi2_10613545_x5....................ucgauucccggcgccggUgc.......................................................................    1
Gi3_27654967_x5.................gguucgauucccggcgcccCga........................................................................    1
Gi5_8575022_x5.................gguucgaCucccggcgccggag........................................................................    1
Gi5_8575227_x5..................guucgaAucccggcgccgg..........................................................................    1
Gi3_27622827_x5.................gguucgauucccggcCccgga.........................................................................    1
Gi3_27653367_x5...................uucgaCucccggcgccggagc.......................................................................    1
Gi1_4003445_x5.................gguucgauNcccggcgccggag........................................................................    1
Gi3_27650387_x5.................gguNcgauucccggcgccgga.........................................................................    1
```

```
Gi2_10604695_x5.................gguucgauucccggAgccggag.........................................................................    1
Gi2_10630375_x5.................gguucgauCcccggcgccgg...........................................................................    1
Gi3_27635117_x5.................gguucAauucccggcgcc.............................................................................    1
Gi3_27601762_x5.................gguucgauucccggGgccgga..........................................................................    1
Gi5_8592097_x5.................gguuGgauucccggcgccggagc........................................................................    1
Gi3_27551832_x5.................gguucgauCcccggcgcc.............................................................................    1
Gi1_4017635_x5..................guucgauucccggcgccggC..........................................................................    1
Gi3_27555052_x5..........agauccggguucgauucccggcgccgg............................................................................    0
Gi3_27644187_x5..................guucgUuucccggcgccggag.........................................................................    1
Gi3_27641547_x5....................ucUauucccggcgccggag.........................................................................    1
Gi3_27608742_x5.................ggAucgauucccggcgcc.............................................................................    1
Gi5_8577467_x5..................guAcgauucccggcgccggagc........................................................................    1
Gi1_4015470_x5..................guucgauucGcggcgccgg...........................................................................    1
Gi5_8576632_x5cucacccaGcagauccgg..............................................................................................    1
Gi1_4009640_x5....................ucgauuccNggcgccggagc........................................................................    1
G1a_5760217_x5.................ggCucgauucccggcgccggagc........................................................................    1
Gi2_10596120_x5..................guucgCuucccggcgccggagc........................................................................    1
Gi3_27610652_x5....................ucgauucUcggcgccggagc........................................................................    1
Gi5_8594457_x5..................guucgauuccUggcgccgga..........................................................................    1
Gi2_10614235_x5..................guucgauucccggUgccg............................................................................    1
Gi5_8565527_x5....cccaacagNuccggguuc..........................................................................................    1
Gi2_10617540_x5....................uUgauucccggcgccggagc........................................................................    1
Gi3_27574032_x5..Gacccaacagauccggguuc..........................................................................................    1
Gi1_3998275_x5..................guucgauucccggcgAcgga..........................................................................    1
Gi5_8601592_x5..................guucgauucccggcgccC............................................................................    1
Gi4_536278_x5 .................gguucgauucccggcgccg............................................................................    0
Gi2_10606795_x5.................gguucgauuccUggcgccgga..........................................................................    1
Gi5_8571622_x5.................gguucgauCcccggcgccggag.........................................................................    1
Gi5_8594797_x5..................guAcgauucccggcgccgga..........................................................................    1
Gi3_27584367_x5.................gguucgauucccgCcgccggagc........................................................................    1
Gi1_3995530_x5.................gguucgauucccggcgAcggagc........................................................................    1
Gi5_8591757_x5.................gguucgauucccggcAccgg...........................................................................    1
Gi2_10627645_x5..................guucgauAcccggcgccgg...........................................................................    1
Gi2_10602770_x5.................gguucgauucccgUcgccggagc........................................................................    1
Gi1_4006125_x5..................guucgauucccggcgccgAa..........................................................................    1
Gi2_10624170_x5.................gguucgauucccggcgccAg...........................................................................    1
Gi1_4011340_x5.................gguucgCuucccggcgccg............................................................................    1
Gi1_4005055_x5..........agUuccggguucgauuccc...................................................................................    1
Gi3_27595842_x5.................gguucgUuucccggcgccgga..........................................................................    1
Gi5_8598442_x5..................guucgaGucccggcgccggagc........................................................................    1
Gi3_27566502_x5................ggguucgauucccggcgUcg............................................................................    1
Gi1_4008040_x5..................guucgauucccggcgGcggagc........................................................................    1
Gi2_10615215_x5.................gguucgauucccgCcgccggagc........................................................................    1
Gi2_10626655_x5.................gguucgauucccggGgccgga..........................................................................    1
Gi3_27640812_x5....cccaacagaGccggguucgauuccc...................................................................................    1
Gi5_8592172_x5.................gguucgauucccggcgccggU..........................................................................    1
Gi1_4012755_x5...................uGcgauucccggcgccggagc........................................................................    1
```

Gi2_10626120_x5.................gguGcgauucccggcgccggagc...   1
Gi3_27572302_x5.................gguucgauucccUgcgcc..   1
Gi2_10616180_x5..................guucgauucccgUcgccg...   1
Gi5_8582857_x5.................gguucgauuAccggcgccggagc...   1
Gi1_4000445_x5.................gguucgauucccggcgccgA..   1
Gi5_8566167_x5................gUguucgauucccggcgccggagc...   1
Gi2_10628225_x5.................gguucgaGucccggcgccggagc...   1
Gi3_27642202_x5.................gguAcgauucccggcgccgga...   1
Gi5_8602037_x5.ucacccaacGgauccggguu..   1
Gi5_8588572_x5........acagauccggguucgauucccggcgccggagcC..   1
Gi2_10591920_x5.................gguucgauucccggcgcUgg..   1
Gi3_27567322_x5......................gauucccggcgccggCgc...   1
Gi2_10630885_x5....................ucgauucccggcgccggCgc...   1
Gi5_8586612_x5.................gguucgauucccggUgccgg..   1
Gi5_8584982_x5.................gguucgauucccggUgccg...   1
Gi5_8575312_x5..................guucgauuccUggcgccggagc...   1
Gi3_27593912_x5.................gguucgauuUccggcgcc..   1
Gi5_8600352_x5..................guucgauCcccggcgccggagc...   1
Gi3_27574737_x5..................guucgauucccggcgccgCa...   1
Gi5_8589032_x5.................gguucgauucccggcgccggagcU..   1
Gi1_4002880_x5..................guucgauucccggcgUcggagc...   1
Gi3_27607117_x5....................ucgauucccggcgccgAagc...   1
Gi1_4017240_x5..................guucgauuNccggcgccggagc...   1
Gi5_8596607_x5.................ggAucgauucccggcgcc..   1
Gi2_10627080_x5..................guucgauucccggAgccg...   1
Gi3_27550742_x5..................guucgaAucccggcgccggagc...   1
Gi1_3994800_x5cucacccaacagGuccgg...   1
Gi1_4011460_x5........aUagauccggguucgauuccc..   1
G1a_5753682_x5.................gguuUgauucccggcgccggagc...   1
Gi3_27618362_x5.Gcacccaacagauccggguuc...   1
Gi3_27581747_x5.................gguucgauucccggcgcAgg..   1
G1a_5763772_x5.................gGucgauucccggcgccg..   1
Gi2_10642120_x5..................guucgauucccggcgcGggagc...   1
Gi3_27589007_x5..................guucgauucccgUcgccggag..   1
Gi3_27586067_x5................gUguucgauucccggcgccggag..   1
Gi3_27595807_x5....................ucgauucccggUgccggagc...   1
Gi5_8591027_x5..................guucgauucccggcgccggU...   1
Gi5_8579392_x5..................guucgauuAccggcgccgg..   1
Gi5_8581037_x5.................gguucgauCcccggcgcc..   1
Gi2_10634505_x5..................guucgauucccggcgAcgga...   1
Gi3_27623577_x5.................gguucgauucccggcUccgga...   1
Gi3_27651732_x5..................guucgauucccggAgccggag..   1
Gi2_10645020_x5.................gguucgauuUccggcgccggag..   1
Gi2_10631330_x5.................gguucgauucccggGgccg...   1
Gi3_27619017_x5.................gguucgauucccggcgccCg..   1
Gi1_3995685_x5....................Gcgauucccggcgccggagc...   1
Gi3_27559772_x5.........cagauccgggGucgauuccc..   1

Gi2_10642000_x5.................ggNucgauucccggcgccggagc.. 1
Gi2_10615105_x5..........agUuccggguucgauucc.. 1
Gi2_10623165_x5..................gCucgauucccggcgccgga.. 1
Gi2_10646650_x5...............cUgguucgauucccggcgccgga.. 1
Gi1_3999215_x5...................uucgauucNcggcgccgg... 1
Gi1_4001215_x5.................gguucgauucccggAgccgg... 1
Gi1_4010650_x5..................guucgauucccggcgccggagcgC.. 1
Gi5_8577237_x5....cGcaacagauccggguuc.. 1
Gi3_27591427_x5....................ucgauuccNggcgccgga.. 1
Gi2_10643425_x5..................guucgauucccggcgccggaCc.. 1
Gi2_10642580_x5..................gAucgauucccggcgccgg... 1
Gi1_4015505_x5..................guucgauucccggGgccgg... 1
Gi3_27621692_x5.................gguuUgauucccggcgccgg... 1
Gi5_8590282_x5.................gguCcgauucccggcgccgga.. 1
Gi3_27651257_x5.................gguucAauucccggcgccgg... 1
Gi5_8597617_x5.................gguucgauucccAgcgccggag... 1
G1a_5752867_x5.................gguucgauucccggcgccggGgc.. 1
Gi3_27612727_x5.................gguucgauucGcggcgccggagc.. 1
Gi3_27549482_x5.................gCuucgauucccggcgcc... 1
Gi2_10605435_x5....................ucgauuccNggcgccggag... 1
Gi1_3996520_x5..................guucUauucccggcgccgga.. 1
Gi3_27577512_x5..................guucgauucccggcgUcg.. 1
Gi5_8569292_x5.................gguucgaCucccggcgccgga.. 1
Gi5_8592012_x5..................Cuucgauucccggcgccggagc.. 1
Gi2_10604880_x5.................gguucgauuccAggcgccgga.. 1
Gi5_8568342_x5.................gUuucgauucccggcgccggagcguu... 1
Gi5_8571147_x5..................guucgauucccggcgcAgga.. 1
Gi1_4003015_x5..................guucgauucccggcgcUg.. 1
Gi3_27588207_x5..........agUuccggguucgauuccc... 1
Gi2_10638655_x5..................guucgauucccggcgcUggag... 1
Gi3_27613417_x5.uGacccaacagauccggguuc.. 1
Gi1_3995375_x5.................gguucgauucccggcgccAga.. 1
Gi1_4005155_x5.................gguucgauNcccggcgccgga.. 1
Gi5_8590502_x5.................gguucgauucccggcgUcgg... 1
Gi1_3996170_x5.................gguucgauucccggcgccgC... 1
Gi2_10633035_x5..................guAcgauucccggcgccggag... 1
Gi2_10592830_x5..................guucgUuucccggcgccggagc.. 1
Gi3_27581737_x5..................guucgauucccggcgAcggag... 1
Gi1_4010410_x5..................Auucgauucccggcgccg.. 1
Gi3_27574422_x5...................uucgauucccggUgccggagc.. 1
Gi5_8590737_x5.................gAuucgauucccggcgccgg... 1
Gi3_27588697_x5..................guucgauucccgAcgccggag... 1
Gi3_27600652_x5....................ucgauuccNggcgccggag... 1
Gi3_27678893_x4..................guucgauucccggcgcccCgag.. 1
Gi3_27681081_x4....................ucgauuccUggcgccggagc.. 1
Gi5_8623915_x4.................gguucgauucccggAgccggag... 1
Gi1_4046500_x4cucacccaacGgauccgg.. 1

Gi2_10693463_x4.................gguucgauuccAggcgccg.. 1
Gi4_538906_x4 ....gguucgauucccggcgccggagcCu... 1
Gi1_4042944_x4.................gguucgauucccggcgcAggagc.. 1
Gi2_10655231_x4.................gguucgauucccggcUccggagc.. 1
Gi3_27766557_x4.....................cgUuucccggcgccggag... 1
Gi1_4033416_x4................ggguucgauucccggcgccgg... 0
Gi3_27696305_x4..................guucgauucccggAgccg.. 1
Gi2_10659599_x4..................guucgauuAccggcgccgg... 1
Gi3_27735613_x4..................guucgauucccggcgcUggag... 1
Gi3_27771121_x4................Cgguucgauucccggcgccggagc.. 1
Gi3_27721721_x4.................gguucgauuGccggcgccggag... 1
Gi1_4026548_x4..................guGcgauucccggcgccggagc.. 1
Gi3_27791545_x4..................guucgauuccGggcgccgga.. 1
Gi5_8616915_x4......................gauucccgNcgccggagc.. 1
Gi2_10706091_x4.................ggAucgauucccggcgccg.. 1
Gi3_27701357_x4..........Ggauccggguucgauucc.. 1
Gi3_27739361_x4.................Uguucgauucccggcgccggagcg... 1
Gi1_4028852_x4..................guucgGuucccggcgccgga.. 1
Gi5_8631527_x4...................uGcgauucccggcgccgg... 1
Gi3_27731089_x4....................Ccgauucccggcgccggagc.. 1
Gi5_8604751_x4.................gguucgauucccggcUccg.. 1
Gi2_10688451_x4..................guucgauucccggcgGcgg... 1
Gi4_539122_x4 ....guucgauucccggcgccggagcC... 1
Gi3_27743185_x4...................uucgauucccggcgccggagcCu.. 1
Gi1_4028916_x4.................gguucgauucccggcgccA.. 1
Gi5_8623479_x4.................gguCcgauucccggcgccggag... 1
G1a_5801486_x4.........cagauccggguucgauucccggcgccggag... 0
Gi3_27772357_x4..................guucgCuucccggcgccggag... 1
Gi3_27667689_x4......caacagauNcggguucga.. 1
Gi2_10677995_x4.................gguucgauucccgAcgccgg... 1
Gi2_10653131_x4..................guGcgauucccggcgccgga.. 1
Gi3_27761817_x4..................guucgauucccggGgccgga.. 1
Gi2_10695131_x4.................gguucgauucccgAcgccgga.. 1
Gi2_10703143_x4.................gguucgauucccggcCccggagc.. 1
Gi1_4029416_x4....................uGgauucccggcgccggagc.. 1
Gi5_8635099_x4cucacccaaUagauccgg.. 1
Gi1_4037048_x4..................guucgauCcccggcgccggagc.. 1
Gi3_27730749_x4......................Aauucccggcgccggagc.. 1
Gi2_10711727_x4..................guucAauucccggcgccggag... 1
Gi5_8619295_x4..................guucgauucAcggcgccggagc.. 1
Gi2_10681647_x4.................gguucCauucccggcgccggagc.. 1
G1a_5790914_x4.................gguucgGuucccggcgccggagc.. 1
Gi5_8640951_x4cucacccaacGgauccgg.. 1
Gi2_10689443_x4.................gguucUauucccggcgccggagc.. 1
Gi2_10695635_x4....................ucgauucccggUgccggagc.. 1
Gi1_4035960_x4..........agauccggguucgCuuccc... 1
Gi1_4030560_x4.................Cguucgauucccggcgccg.. 1

Gi5_8629787_x4.................gguucgauucccUgcgccgga...   1
Gi1_4039608_x4....................ucgauucccggcgccggagcCu...   1
G1a_5767494_x4.................gguucgauNcccggcgccgg..   1
Gi2_10665975_x4.................gguucgauucUcggcgccgga...   1
Gi5_8650211_x4.................gguucgauuccAggcgccg...   1
Gi3_27795865_x4.................gguucgauucccggcgccCgag..   1
Gi1_4043420_x4.................gguucgCuucccggcgccggagc...   1
Gi1_4017968_x4.................gguucgauuAccggcgccgg..   1
Gi3_27739557_x4.................gguucgauucccggcgccC...   1
Gi3_27753053_x4......caUcagauccggguucgauuccc..   1
Gi1_4047284_x4..................guucgGuucccggcgccggagc...   1
Gi2_10684211_x4.................gguucgauuccUggcgccgg..   1
Gi5_8627359_x4.................gguucgauucccggAgcc..   1
Gi2_10660771_x4..................guucgauucccggAgccgg..   1
Gi2_10670415_x4.............Gccggguucgauucccggcgccgga...   1
Gi3_27779217_x4..................guucgauAcccggcgccggag..   1
Gi3_27773113_x4................gUguucgauucccggcgccgga...   1
Gi1_4031772_x4..................guucgauucccggcgccgCagc...   1
Gi5_8647743_x4.ucacccaacagauccgggCu..   1
Gi1_4025808_x4..................guucgauucccAgcgccg...   1
Gi2_10712155_x4....................ucgaGucccggcgccggagc...   1
Gi1_4023416_x4cCcacccaacagauccgg...   1
Gi5_8620595_x4.................gguAcgauucccggcgccg...   1
Gi3_27792825_x4..................guucCauucccggcgccgg..   1
Gi3_27769641_x4.................gguucUauucccggcgccggag..   1
Gi3_27764721_x4......................gauucccggcgcUggagc...   1
Gi2_10685383_x4.................gguucgauucccgAcgccggag..   1
Gi3_27668109_x4..................guucgauucccggcgAcg...   1
Gi5_8632635_x4.................gguucgauucccggcgccU...   1
Gi5_8633523_x4..................guucAauucccggcgccgga...   1
Gi3_27680801_x4.................gguucgauucccggcCccggag..   1
G1a_5776354_x4.................gguucgauucccggcgccggagcCu...   1
Gi3_27734441_x4.....................cgauucccggcgccggagcA..   1
Gi3_27749101_x4...................uucgauCcccggcgccgga...   1
Gi5_8607155_x4....Gccaacagauccggguuc...   1
Gi2_10666835_x4....................Acgauucccggcgccggagc...   1
Gi2_10704539_x4................Agguucgauucccggcgccggagc...   1
Gi2_10661863_x4..................guucgauuAccggcgccg...   1
Gi2_10697727_x4..................guucgauucccCgcgccgg..   1
Gi3_27664693_x4.........cGgauccggguucgauucccggcgccgg..   1
Gi3_27778965_x4cuUacccaacagauccgg...   1
Gi2_10718583_x4.................ggAucgauucccggcgccgga...   1
Gi5_8623607_x4..................guucgauucccggcgcUggagc...   1
Gi2_10678799_x4...................uuUgauucccggcgccgg..   1
Gi3_27787413_x4....................ucgauucccgAcgccggagc...   1
Gi3_27683097_x4..................guNcgauucccggcgccgga...   1
Gi5_8648907_x4..................guucgauucccggcgcGg...   1

```
Gi3_27778913_x4..................guucgauucccggcgGcg...........................................................................     1
G1a_5786382_x4..................guucgaCucccggcgccgga.........................................................................     1
Gi5_8633619_x4..................guucgauucccggcgccggagcU......................................................................     1
Gi3_27730345_x4.................gguucgauuAccggcgccgg..........................................................................     1
G1a_5807290_x4..................guucgauucccggcgccggCgc.......................................................................     1
Gi3_27795609_x4...................uCcgauucccggcgccggagc.......................................................................     1
Gi2_10706115_x4.................gguucgauucccgAcgccg...........................................................................     1
G1a_5812030_x4..................guucgaCucccggcgccg...........................................................................     1
Gi5_8622683_x4..................guCcgauucccggcgccggagc.......................................................................     1
Gi2_10687567_x4..................guucCauucccggcgccgg..........................................................................     1
Gi1_4022432_x4.................gguucgauucccggcgcGgg..........................................................................     1
Gi5_8639583_x4.................gguuUgauucccggcgccgg..........................................................................     1
Gi3_27746029_x4.................gguucgUuucccggcgccggag........................................................................     1
Gi1_4024384_x4......................gauucccggcgccggagcC......................................................................     1
G1a_5787674_x4.................ggGucgauucccggcgccg...........................................................................     1
Gi5_8624019_x4..................guucgauucUcggcgccggagc.......................................................................     1
Gi5_8602619_x4..................guucgGuucccggcgccgga.........................................................................     1
Gi1_4039752_x4cucacccaNcagauccgg.............................................................................................     1
Gi2_10670995_x4..................guucgGuucccggcgccggag........................................................................     1
Gi2_10681731_x4..................guucgauucccUgcgccgg..........................................................................     1
Gi1_4031176_x4..................guucgauucccAgcgccggagc.......................................................................     1
Gi3_27771089_x4.................gguucgauAcccggcgccg...........................................................................     1
Gi3_27695869_x4.................gguucCauucccggcgccggagc.......................................................................     1
Gi3_27753009_x4......cGacagauccggguucgauuccc..................................................................................     1
G1a_5768570_x4.................Aguucgauucccggcgccggag........................................................................     1
Gi2_10657263_x4..................guucgauucccgAcgccg...........................................................................     1
Gi1_4034080_x4..................guucgauuNccggcgccg...........................................................................     1
G1a_5784282_x4.................gguucgauNcccggcgccggag........................................................................     1
Gi3_27778521_x4..................guucgauucccggcgccUgagc.......................................................................     1
Gi2_10682415_x4..................guucgauuUccggcgccgga.........................................................................     1
Gi5_8624351_x4.................gguucgauucccggcgccggUg........................................................................     1
Gi2_10666019_x4..................guucgauucccggcgcAgga.........................................................................     1
Gi1_4028676_x4.................gguucgauuccAggcgccgg..........................................................................     1
Gi5_8623143_x4.................gguCcgauucccggcgcc............................................................................     1
Gi5_8638887_x4..................guucgauuUccggcgccgga.........................................................................     1
Gi3_27685973_x4..........agauccggNuucgauuccc..................................................................................     1
Gi2_10700483_x4.................gguucgauucUcggcgccg...........................................................................     1
Gi5_8603079_x4.................gguucgauucccCgcgccg...........................................................................     1
Gi1_4036324_x4.................gCuucgauucccggcgccgga.........................................................................     1
Gi3_27784853_x4.................gguucgauuAccggcgcc............................................................................     1
Gi5_8619903_x4..................guuGgauucccggcgccg...........................................................................     1
Gi1_4037176_x4.................gguCcgauucccggcgccggagc.......................................................................     1
Gi3_27752297_x4..................guucgauucccggcgcGg...........................................................................     1
Gi1_4025512_x4..................guucgauuccUggcgccggagc.......................................................................     1
Gi1_4046608_x4.................gguucgauAcccggcgccgg..........................................................................     1
Gi5_8611723_x4...........gauccggguucgauuccc..................................................................................     0
Gi5_8642475_x4.................gguucgauucccggcgccgA..........................................................................     1
```

Gi1_4047016_x4.................gguucgauucccggcgcUggagc..	1
Gi1_4039380_x4..................guucgauucccggcgccggagU..	1
Gi5_8605199_x4cucacccaacCgauccgg..	1
Gi2_10677467_x4..................gAucgauucccggcgccg..	1
Gi3_27763597_x4.........cagauccggguucgGuuccc...	1
Gi5_8608147_x4cucacccGacagauccgg..	1
Gi5_8614263_x4.................gguucgauucccggcgAcgg...	1
Gi1_4043404_x4..................guucgauucccgCcgccgg...	1
Gi2_10660663_x4...................uucgauucNcggcgccgg...	1
Gi5_8612051_x4cucacccaacagauccAg..	1
Gi5_8609459_x4.................gguucgCuucccggcgccgg...	1
Gi3_27685073_x4.................gguucgauuAccggcgccg..	1
Gi1_4033656_x4..........agauccggNuucgauuccc...	1
Gi5_8611627_x4..................guucgauucccggcgccgAa..	1
Gi3_27781409_x4....................ucgauucccggcgccAga..	1
Gi3_27694377_x4..................guucgauucUcggcgccggag...	1
Gi3_27715681_x4.................gguucgauucccgAcgcc...	1
Gi1_4032704_x4.................gguucgauucccggAgccggagc..	1
Gi2_10672991_x4..................guAcgauucccggcgccgga..	1
Gi1_4043464_x4.................ggCucgauucccggcgccg..	1
Gi1_4025668_x4..................guucgauucccggcgUcgga..	1
Gi1_4022416_x4.................gguucgauucccAgcgccggagc..	1
Gi3_27713285_x4...............Gggguucgauucccggcgccgga..	1
Gi2_10721295_x4.........cagauccggguucgauucccggcgccgga..	0
Gi5_8632475_x4.................gguuUgauucccggcgcc...	1
Gi3_27672645_x4......................gauuUccggcgccggagc..	1
Gi3_27790573_x4..................guucgaGucccggcgccggag...	1
Gi3_27735701_x4..................guucgauuccGggcgccgg...	1
Gi2_10685011_x4.................gAuucgauucccggcgccg..	1
Gi2_10690695_x4.................gguucgGuucccggcgccgg...	1
Gi3_27683689_x4....................ucgGuucccggcgccgga..	1
Gi2_10723707_x4....................ucgauuccUggcgccggagc..	1
Gi1_4026372_x4.ucacccaacagauccgggC..	1
Gi3_27718133_x4.................gguucgauAcccggcgcc...	1
Gi2_10700555_x4.................gguucgauucccggcgccgAag...	1
Gi2_10657371_x4..................guucgauucccggcgccAgag...	1
Gi2_10717623_x4.................gguucgauuGccggcgccggagc..	1
Gi3_27754773_x4....................uUgauucccggcgccgga..	1
Gi3_27745305_x4.................gguucgaGucccggcgccgga..	1
Gi2_10702083_x4.................gguAcgauucccggcgccggag...	1
Gi2_10683207_x4cGcacccaacagauccgg..	1
Gi3_27737637_x4.................gguucgauucccggcgccggaC...	1
Gi2_10669571_x4................ggguucgauucccggcgccggagc..	0
Gi1_4040124_x4..................guCcgauucccggcgccgga..	1
Gi1_4018196_x4...................uucgauucccggcgcUgg...	1
Gi5_8615995_x4.................gAuucgauucccggcgccggag...	1
Gi5_8625803_x4.................gguucgCuucccggcgcc...	1

```
Gi2_10724311_x4.................gguucgauucccAgcgccgg...........................................................................    1
Gi2_10693895_x4..................guucgauucccggcgUcggag.........................................................................    1
Gi3_27780217_x4....................Ccgauucccggcgccgga..........................................................................    1
Gi5_8638219_x4..................guucgaAucccggcgccg............................................................................    1
Gi5_8644247_x4.................gguuGgauucccggcgccg............................................................................    1
G1a_5773650_x4..................Uuucgauucccggcgccgga..........................................................................    1
Gi3_27937088_x3..................Nuucgauucccggcgccggagc........................................................................    1
Gi5_8685113_x3..................guucgauucccggcgccggaAc........................................................................    1
Gi3_27970415_x3.............uGcggguucgauucccggcgccgga..........................................................................    1
Gi3_27834023_x3..................guuNgauucccggcgccgga..........................................................................    1
Gi3_27933464_x3....................ucgauucccggcgccggagcCu......................................................................    1
Gi3_27888362_x3.................gguucgauucccgCcgccgg...........................................................................    1
Gi2_10795600_x3.................gguucgauucccggcgccUgag.........................................................................    1
Gi2_10730596_x3..................guucgauucccggcgccC............................................................................    1
Gi3_27804551_x3.................gguucgauucccggGgccg............................................................................    1
G1a_5875426_x3.................gguCcgauucccggcgccgg...........................................................................    1
Gi2_10765123_x3..................guucCauucccggcgccgga..........................................................................    1
Gi1_4074720_x3............auccggguucgauucccggcgccAg...........................................................................    1
Gi1_4063590_x3..................guucgauucccggcgccCga..........................................................................    1
Gi3_27870452_x3.................gguucgauucccggcgcU.............................................................................    1
Gi1_4075323_x3....................ucgauucccggcgccggag.........................................................................    0
Gi2_10794589_x3....................ucgauucccggcgccgAagc........................................................................    1
Gi5_8664269_x3.................gguucgauuccUggcgccggag.........................................................................    1
Gi3_27912119_x3.......aacagauccggguucgauucccggcgccggagcC.......................................................................    1
Gi3_27835121_x3...................uucgauucccgAcgccggagc........................................................................    1
Gi3_27893261_x3..................guucgauuAccggcgccggag.........................................................................    1
Gi5_8686355_x3.......aacagauccggguucgauucccggcgccggagc........................................................................    0
Gi5_8675351_x3.................gguucgauucAcggcgccgga..........................................................................    1
Gi5_8661461_x3..................Auucgauucccggcgccggag.........................................................................    1
Gi2_10781692_x3..................guucgauucUcggcgccggag.........................................................................    1
Gi2_10786429_x3....................uGgauucccggcgccggag.........................................................................    1
Gi2_10790677_x3..................guucgauucccggGgccggagc........................................................................    1
Gi3_27917921_x3................ggguucgauucccggcgccgg...........................................................................    0
Gi1_4056333_x3.................gguucgauuccUggcgccg............................................................................    1
Gi3_27849311_x3..................guucgauucccggcgccggagG........................................................................    1
Gi2_10779409_x3..................guucgCuucccggcgccggag.........................................................................    1
Gi5_8699609_x3....................ucgauucccgAcgccgga..........................................................................    1
Gi5_8673644_x3.................gguucgauucccggcgcAggag.........................................................................    1
Gi2_10803289_x3.................gguucgauucAcggcgccg............................................................................    1
Gi5_8656658_x3.................gguNcgauucccggcgccggagc........................................................................    1
Gi2_10779490_x3..................guucgaGucccggcgccgg...........................................................................    1
Gi5_8669315_x3....cccaacagauccggguCc..........................................................................................    1
Gi3_27967724_x3....................ucUauucccggcgccgga..........................................................................    1
Gi5_8717162_x3Aucacccaacagauccgg..............................................................................................    1
Gi2_10748152_x3...................Gucgauucccggcgccggag.........................................................................    1
Gi3_27829070_x3.................gguucgauucccggcUccggag.........................................................................    1
Gi1_4057449_x3.................gguucgauucccggcgccAgagc........................................................................    1
```

```
Gi2_10792240_x3.................gguucgauucccggcgccgA...........................................................................     1
Gi2_10794754_x3.................gguucAauucccggcgccg............................................................................     1
Gi2_10798258_x3.................gguucgauucccggcgGcgg...........................................................................     1
Gi5_8675369_x3....................Gcgauucccggcgccgga..........................................................................     1
Gi3_27949205_x3..................guucgauAcccggcgccg............................................................................     1
Gi5_8709440_x3.................gguucgauAcccggcgccg............................................................................     1
Gi1_4085742_x3..................guucUauucccggcgccggagc........................................................................     1
Gi5_8666963_x3.................gguucgauucccggcgccggaUc........................................................................     1
Gi1_4076667_x3..................guucgauucccggAgccg............................................................................     1
Gi3_27818741_x3..................guucgUuucccggcgccggagc........................................................................     1
Gi5_8669591_x3.................gguucgauucccggAgccgg...........................................................................     1
Gi2_10730764_x3...................uCcgauucccggcgccgg...........................................................................     1
Gi1_4075806_x3..................Nuucgauucccggcgccgg...........................................................................     1
Gi2_10761703_x3..................guucgauucccggcgcGgga..........................................................................     1
Gi2_10749778_x3.................gguAcgauucccggcgccgga..........................................................................     1
Gi3_27911471_x3................gUguucgauucccggcgccggagc........................................................................     1
Gi5_8698340_x3.................gguucgauucccggcgccCg...........................................................................     1
Gi3_27886430_x3..................guuGgauucccggcgccgga..........................................................................     1
Gi5_8680985_x3........................................gGuucccgccuucuucgucu....................................................     1
Gi5_8658599_x3.....................cgauucccggcgccggagcCu......................................................................     1
Gi1_4083423_x3.................gguucgauucccggUgccggagc........................................................................     1
Gi5_8663858_x3.................gguucgauCcccggcgccgga..........................................................................     1
Gi3_27948005_x3..................guucgauucccggGgccggagc........................................................................     1
Gi5_8652935_x3..................guucgauuccAggcgccgga..........................................................................     1
Gi2_10767859_x3.................Nguucgauucccggcgccggagc........................................................................     1
Gi3_27975029_x3......................gUuucccggcgccggagc........................................................................     1
Gi2_10816255_x3....cccaacagaCccggguuc..........................................................................................     1
Gi4_550752_x3 .................ggGucgauucccggcgccgg...........................................................................     1
Gi3_27925016_x3.................gguucgauuGccggcgccgga..........................................................................     1
Gi3_27849674_x3.................gguucgauuGccggcgccgg...........................................................................     1
Gi1_4067886_x3............auccggguucgauuccAggcgccgg...........................................................................     1
Gi3_27859577_x3.............uccggguucgauucccggcgccgAa..........................................................................     1
Gi5_8679263_x3.................gguucgauucUcggcgcc.............................................................................     1
Gi5_8653355_x3.................gguucgauuAccggcgccgga..........................................................................     1
Gi2_10750684_x3.................gguucgauucccggAgccgga..........................................................................     1
Gi3_27877115_x3..................Nuucgauucccggcgccggag.........................................................................     1
Gi5_8652497_x3..................guucgauucccggcgccCga..........................................................................     1
Gi5_8682884_x3.........cagauccgggCucgauucccggcgccg............................................................................     1
Gi5_8672801_x3.................gguucgauucccgAcgccgg...........................................................................     1
Gi3_27936176_x3......................gauucccggcAccggagc........................................................................     1
Gi5_8712632_x3..................guuNgauucccggcgccgg...........................................................................     1
Gi5_8678831_x3.................gguucgaAucccggcgccg............................................................................     1
Gi1_4082253_x3..................guucgauCcccggcgccgga..........................................................................     1
Gi5_8676506_x3.Acacccaacagauccggguu...........................................................................................     1
Gi1_4058463_x3..................guucgauucccgUcgccgga..........................................................................     1
Gi3_27911921_x3.................gguucgauGcccggcgccg............................................................................     1
Gi2_10783765_x3..................guucgauucccggcgccgU...........................................................................     1
```

```
Gi2_10800736_x3.................gguucCauucccggcgccggag.........................................................................     1
Gi3_27926360_x3...................Aucgauucccggcgccggagc........................................................................     1
Gi1_4085529_x3..................guuUgauucccggcgccg............................................................................     1
Gi5_8708984_x3....................Ccgauucccggcgccggagc........................................................................     1
Gi1_4076907_x3.................gguucgauucccggUgccgga..........................................................................     1
Gi3_27845336_x3cucacccaacagGuccgg..............................................................................................     1
Gi1_4051002_x3..................guucgauuccGggcgccgg...........................................................................     1
Gi3_27922640_x3....................ucgauucccggUgccgga..........................................................................     1
Gi3_27911900_x3..................guucCauucccggcgccgga..........................................................................     1
Gi2_10756783_x3.................gguucgauAcccggcgcc.............................................................................     1
Gi5_8684030_x3.ucacccaacagauccgggGu...........................................................................................     1
Gi3_27867647_x3.................Nguucgauucccggcgccggag.........................................................................     1
G1a_5836006_x3.................gguucgauucccggcAccggagc........................................................................     1
Gi2_10762363_x3...................uucgaCucccggcgccggagc........................................................................     1
Gi3_27875945_x3..........agauccggguCcgauuccc...................................................................................     1
Gi2_10811437_x3..................guucgauucAcggcgccg............................................................................     1
Gi2_10759840_x3....................ucUauucccggcgccggagc........................................................................     1
Gi3_27937673_x3...........Uauccggguucgauucccggcgccgga..........................................................................     1
Gi4_547647_x3 .................gguucgauucccggcgccggag.........................................................................     0
Gi3_27827708_x3..................guucgUuucccggcgccgg...........................................................................     1
Gi2_10761202_x3.................gguucgauucUcggcgccgg...........................................................................     1
Gi3_27821375_x3....................ucgauCcccggcgccggagc........................................................................     1
Gi1_4082823_x3.................gAuucgauucccggcgccgga..........................................................................     1
G1a_5840212_x3.................gguucgGuucccggcgcc.............................................................................     1
Gi2_10743808_x3....................Gcgauucccggcgccgga..........................................................................     1
Gi3_27869762_x3......................gauucccgNcgccggagc........................................................................     1
Gi5_8672123_x3.................gguucgauucUcggcgccgg...........................................................................     1
Gi3_27896120_x3...................uucgauuccUggcgccgga..........................................................................     1
G1a_5850052_x3..................guucgaCucccggcgccggagc........................................................................     1
Gi3_27905309_x3.........cagauccgNguucgauuccc...................................................................................     1
Gi5_8693288_x3......................gauucccggcgccggGgc........................................................................     1
Gi5_8657537_x3....................Gcgauucccggcgccggag.........................................................................     1
Gi3_27890375_x3...............cggguucgauucccggcgccgC...........................................................................     1
G1a_5853931_x3..................guucgGuucccggcgccggagc........................................................................     1
Gi3_27813977_x3....................ucgaCucccggcgccgga..........................................................................     1
Gi3_27887441_x3cucacccGacagauccgg..............................................................................................     1
Gi1_4048029_x3.................gguucgauucccggcgccggagA........................................................................     1
G1a_5858230_x3..................gGucgauucccggcgccgg...........................................................................     1
Gi1_4080300_x3.................gguuAgauucccggcgccgg...........................................................................     1
Gi3_27821048_x3.................gguucgauucccgUcgccggagc........................................................................     1
Gi3_27884132_x3..................guucgaAucccggcgccgga..........................................................................     1
Gi1_4088190_x3.................gguucgauucUcggcgccgga..........................................................................     1
Gi2_10779472_x3.................gguucgauucccggcgcAg............................................................................     1
Gi1_4057824_x3..................guucgauCcccggcgccg............................................................................     1
Gi1_4088490_x3.................gguucgauucccggcgccggGg.........................................................................     1
Gi3_27834674_x3..........aUauccggguucgauucccggcgccggagc........................................................................     1
Gi5_8658845_x3.................gguucgauuccUggcgcc.............................................................................     1
```

```
Gi5_8663273_x3..................Uuucgauucccggcgccggagcguuucc..................................................................     1
Gi1_4081971_x3.................ggGucgauucccggcgccggagc........................................................................     1
Gi2_10824061_x3.................gguucgauucAcggcgcc.............................................................................     1
Gi5_8671412_x3..............ccggguucgauucccggcgccggC..........................................................................     1
Gi2_10804873_x3.................gguucgauucccggcgAcgg...........................................................................     1
Gi2_10796326_x3......................gUuucccggcgccggagc........................................................................     1
Gi3_27883871_x3...................uucgauucccggcgccggagcgA......................................................................     1
Gi2_10791523_x3.................gguucgauuUccggcgccgg...........................................................................     1
Gi2_10726432_x3.................gguucgauucGcggcgccggagc........................................................................     1
Gi5_8656268_x3..................guGcgauucccggcgccggagc........................................................................     1
Gi5_8701292_x3..................guucgauucccUgcgccgg...........................................................................     1
Gi3_27830687_x3.................gguucgauucccCgcgccgga..........................................................................     1
Gi2_10736971_x3.................gguucgauucccggcgccgC...........................................................................     1
Gi5_8683811_x3.................gCuucgauucccggcgcc.............................................................................     1
Gi2_10784854_x3.................gguucgauAcccggcgccg............................................................................     1
Gi5_8658503_x3.................ggAucgauucccggcgccggag.........................................................................     1
Gi5_8698208_x3cucaGccaacagauccgg..............................................................................................     1
Gi2_10785745_x3.................gguucgauucccggcgcGgga..........................................................................     1
Gi5_8672588_x3.................gguucgauucccCgcgccggagc........................................................................     1
Gi5_8657915_x3.................gguucgauucccUgcgccg............................................................................     1
Gi3_27955169_x3.................gguucUauucccggcgccg............................................................................     1
Gi1_4089084_x3..................guucgauuUccggcgccg............................................................................     1
Gi5_8692604_x3cucUcccaacagauccgg..............................................................................................     1
Gi5_8714339_x3....................ucUauucccggcgccggagc........................................................................     1
Gi5_8695913_x3.................gguucgauucccggcgccgCa..........................................................................     1
Gi1_4079901_x3.................ggAucgauucccggcgccggagc........................................................................     1
Gi3_27941390_x3...................uucgauCcccggcgccggagc........................................................................     1
Gi3_27818801_x3..................guucgauuccAggcgccg............................................................................     1
Gi5_8715362_x3..................guucgauucccggcgUcgga..........................................................................     1
Gi3_27931829_x3....................ucgauuUccggcgccggag.........................................................................     1
Gi2_10811659_x3.................gguucgauucccggcgGc.............................................................................     1
Gi5_8684414_x3..........agauccgggGucgauuccc...................................................................................     1
Gi3_27828017_x3..........aUauccggguucgauucccggcgccggag.........................................................................     1
G1a_5854144_x3.................gguucgauucccggcgccggCgc........................................................................     1
Gi2_10779790_x3.................gguucgauucccggcCccggag.........................................................................     1
Gi5_8688299_x3..................guucgCuucccggcgccggagc........................................................................     1
Gi2_10746955_x3..................guucgaGucccggcgccgga..........................................................................     1
Gi2_10745776_x3.................gguuAgauucccggcgccgga..........................................................................     1
Gi4_544908_x3 .................gguucgauNcccggcgccgg...........................................................................     1
Gi5_8702387_x3cucacccaacagaucUgg..............................................................................................     1
Gi1_4073718_x3..................guucgauuUccggcgccggagc........................................................................     1
Gi2_10743196_x3..................guucgauCcccggcgccggag.........................................................................     1
Gi5_8673065_x3....................ucgauucccggcgccggagcCu......................................................................     1
Gi5_8673704_x3.................gguucgauucccggcgccggagcgCu.....................................................................     1
Gi5_8711294_x3.................gguucgauucccggcgccAgag.........................................................................     1
Gi1_4065948_x3.................gguCcgauucccggcgccg............................................................................     1
Gi2_10751776_x3..................guucgauucccggcgccggU..........................................................................     1
```

```
Gi3_27958253_x3....................uUgauucccggcgccggag.........................................................................     1
Gi1_4074957_x3..................guucgauucccCgcgccgga..........................................................................     1
Gi3_27892247_x3.................gguucgauucccggcgcGgga..........................................................................     1
Gi2_10746802_x3.................gAuucgauucccggcgccgga..........................................................................     1
Gi3_27836450_x3..................guucgauucccggcgccgUag.........................................................................     1
Gi3_27812366_x3.................gguucgauucGcggcgccggag.........................................................................     1
Gi5_8666495_x3.ucacccaGcagauccggguu...........................................................................................     1
Gi5_8680913_x3..................guucgauucccggUgccggagc........................................................................     1
Gi5_8688164_x3..................guucgauucUcggcgccgga..........................................................................     1
Gi2_10816390_x3..................guuNgauucccggcgccggagc........................................................................     1
Gi3_27813704_x3.............uccgggCucgauucccggcgccgga..........................................................................     1
Gi3_27838577_x3........................uucccggcgccggagcCu......................................................................     1
Gi1_4079502_x3...................uucgaCucccggcgccgg...........................................................................     1
Gi5_8676110_x3....................ucgauuccNggcgccggag.........................................................................     1
Gi5_8711684_x3.................gguucAauucccggcgccgga..........................................................................     1
Gi2_10796617_x3..................guucgauucccggcgcGgg...........................................................................     1
Gi5_8694449_x3.................gguucgauucccggcgccCgagc........................................................................     1
Gi5_8697125_x3..................guucgauucccgUcgccggagc........................................................................     1
Gi2_10803223_x3..................guuGgauucccggcgccgga..........................................................................     1
Gi2_10769323_x3.......................auucccggcgccggagcCu......................................................................     1
Gi3_27813083_x3...............cggguucgNuucccggcgccgga..........................................................................     1
Gi2_10753882_x3.................gguucgauucccggcgccggaC.........................................................................     1
Gi2_10822213_x3.....................cgauucccNgcgccggagcguuu....................................................................     1
Gi3_27932771_x3.................gguucgauucccCgcgccg............................................................................     1
Gi5_8705831_x3..................gAucgauucccggcgccgga..........................................................................     1
Gi4_552126_x3 ..................guucgaCucccggcgccggagc........................................................................     1
Gi1_4071663_x3.................gguucgauucccggcgcUgga..........................................................................     1
Gi3_27947888_x3.................gguucgauucccgCcgccg............................................................................     1
Gi1_4048947_x3cuUacccaacagauccgg..............................................................................................     1
Gi2_10804204_x3..................guucgauucccggcgccgUagc........................................................................     1
G1a_5872276_x3.................gguucgaCucccggcgccggagc........................................................................     1
Gi3_27876740_x3..................guuAgauucccggcgccgga..........................................................................     1
Gi5_8713547_x3..................guucgauuGccggcgccgg...........................................................................     1
Gi1_4063098_x3.................gguucgauuccGggcgccgg...........................................................................     1
Gi1_4071303_x3cucacccaacagaucUgg..............................................................................................     1
G1a_5855329_x3.................Aguucgauucccggcgccgg...........................................................................     1
Gi1_4078809_x3.................gguucgauucccggcgccggagcCuu.....................................................................     1
Gi2_10755211_x3....cGcaacagauccggguuc..........................................................................................     1
Gi3_27939230_x3................ggguucgauucccggcgccggCgc........................................................................     1
Gi3_27963530_x3.................gguucgauucccggcgcA.............................................................................     1
Gi3_27940733_x3.................gguuAgauucccggcgcc.............................................................................     1
Gi3_27874355_x3.............uccggguuNgauucccggcgccgga..........................................................................     1
Gi2_10822219_x3.................gguucgauucccggcgccggagcg.......................................................................     0
Gi3_27944972_x3....................ucUauucccggcgccggagc........................................................................     1
Gi1_4077564_x3.................gguucgGuucccggcgccggagc........................................................................     1
Gi3_27881726_x3.................gguucgauucccgUcgccggag.........................................................................     1
Gi5_8716589_x3..................guucgauucccggAgccgga..........................................................................     1
```

```
Gi3_27822023_x3............auUcggguucgauucccggcgccgga.........................................................................     1
Gi3_27846707_x3....................Acgauucccggcgccgga.........................................................................     1
Gi3_27843638_x3.................gguAcgauucccggcgccgg..........................................................................     1
Gi1_4057752_x3.................gguucgaCucccggcgccgga.........................................................................     1
Gi1_4087377_x3.................gAuucgauucccggcgccggagc.......................................................................     1
Gi2_10791622_x3.................gguuAgauucccggcgccggag........................................................................     1
Gi1_4059540_x3.................gguucAauucccggcgccg...........................................................................     1
Gi2_10790146_x3.................gguucgauucccggcgccgAa.........................................................................     1
Gi5_8701718_x3.............Gccggguucgauucccggc...............................................................................     1
Gi3_27913643_x3..........agauccggguucgUuuccc..................................................................................     1
Gi5_8700377_x3...................uuUgauucccggcgccgg..........................................................................     1
Gi2_10776085_x3.................gguucUauucccggcgcc............................................................................     1
Gi3_27864263_x3..................guucgauucccggcgccU...........................................................................     1
Gi1_4072857_x3..................guucgauucccggcgccgCa.........................................................................     1
Gi3_27820343_x3....................ucAauucccggcgccggagc.......................................................................     1
Gi3_27864914_x3...................uucgaCucccggcgccgg..........................................................................     1
Gi1_4054593_x3cucacccGacagauccgg.............................................................................................     1
Gi1_4075113_x3..cacccaacagauccgggGucgauuccc..................................................................................     1
Gi3_27950240_x3............aGccggguucgauucccggcgccgga.........................................................................     1
Gi5_8669648_x3..................guucgauucccggcgccUgagc.......................................................................     1
Gi1_4073901_x3....................uUgauucccggcgccggagc.......................................................................     1
Gi2_10813018_x3....ccUaacagauccggguuc.........................................................................................     1
Gi1_4060719_x3.................gguucgauucAcggcgccgg..........................................................................     1
Gi2_10745017_x3.................gguucgauucccggcgccCgag........................................................................     1
Gi3_27869522_x3..................guucgauucccgCcgccgg..........................................................................     1
Gi2_10743235_x3.................gguucgauucccggcgcGgag.........................................................................     1
Gi5_8653064_x3......................Cauucccggcgccggagc.......................................................................     1
Gi5_8658149_x3..................guGcgauucccggcgccgga.........................................................................     1
Gi1_4089273_x3.................gguucgaCucccggcgccg...........................................................................     1
Gi5_8706995_x3.................gguucgauucccggcgcA............................................................................     1
Gi1_4063026_x3..................gAucgauucccggcgccgga.........................................................................     1
Gi2_10828222_x3.................gguucgauucccCgcgccgga.........................................................................     1
Gi3_27892118_x3..................guuNgauucccggcgccgg..........................................................................     1
Gi3_27816947_x3.................ggNucgauucccggcgcc............................................................................     1
Gi1_4057833_x3.................gguucgauucccUgcgccgg..........................................................................     1
Gi3_27954461_x3.................gguuAgauucccggcgccg...........................................................................     1
Gi2_10785715_x3....................ucgauucAcggcgccggagc.......................................................................     1
Gi3_27914537_x3..................guucgauucccUgcgccgga.........................................................................     1
Gi3_27868535_x3.........cagauccggguCcgauuccc..................................................................................     1
Gi5_8653847_x3...................uCcgauucccggcgccgg..........................................................................     1
Gi3_27909551_x3...................uucgauuccUggcgccggagc.......................................................................     1
Gi3_27935387_x3......................Cauucccggcgccggagc.......................................................................     1
Gi3_27827657_x3..................gAucgauucccggcgccg...........................................................................     1
Gi2_10776802_x3..................guucgauucccggAgccgga.........................................................................     1
Gi2_10778767_x3.................gguucgauuccAggcgccggag........................................................................     1
Gi5_8660549_x3..................guucgauuccAggcgccgg..........................................................................     1
Gi2_10821682_x3.................gguucgauucccUgcgcc............................................................................     1
```

```
Gi1_4055508_x3.................gguucgauucccgAcgccgg..........................................................................     1
Gi2_10823227_x3..................guucgauucccAgcgccggag........................................................................     1
Gi3_27834272_x3..................guuAgauucccggcgccggagc.......................................................................     1
Gi2_10784119_x3.................gguAcgauucccggcgccg...........................................................................     1
Gi3_27814679_x3....................ucgauucccggcgcAggagc.......................................................................     1
Gi3_27954134_x3..................guucgauucccUgcgccggagc.......................................................................     1
Gi3_27925910_x3.....................cgauuccUggcgccggagc.......................................................................     1
G1a_5845960_x3..................guucgauucccggcgccggagcCu.....................................................................     1
Gi3_27851090_x3...............Gggguucgauucccggcgccggag........................................................................     1
Gi3_27906098_x3...................uucgauuUccggcgccgga.........................................................................     1
Gi5_8680364_x3.................gguucgauucUcggcgccg...........................................................................     1
Gi1_4087308_x3..................guucgGuucccggcgccg...........................................................................     1
Gi2_10783159_x3..................guucgauuccGggcgccgg..........................................................................     1
Gi2_10790641_x3.................gguucgauuUccggcgccg...........................................................................     1
Gi1_4068717_x3.................gguucgauucccggcgUcgga.........................................................................     1
Gi2_10780225_x3......................Aauucccggcgccggagc.......................................................................     1
Gi5_8716610_x3.................gguucgauucccggcgcU............................................................................     1
Gi5_8692454_x3.................gguucgauucccggUgccggag........................................................................     1
Gi2_10801846_x3..................guucgaAucccggcgccggagc.......................................................................     1
Gi3_27842411_x3.................gguucgauuccGggcgccgg..........................................................................     1
Gi3_27964262_x3.................gguucgauucccggcgccgUa.........................................................................     1
Gi3_27913328_x3......caacagauNcgguucgauuccc...................................................................................     1
Gi5_8655605_x3..................guucgauucccggcgccAga.........................................................................     1
Gi3_27884855_x3...................uucgauuUccggcgccggagc.......................................................................     1
Gi1_4073733_x3...................uuUgauucccggcgccggagc.......................................................................     1
Gi2_10795651_x3.................gguucgauuAccggcgccgga.........................................................................     1
Gi4_548499_x3 .................gguucgauucccggcgcc............................................................................     0
Gi2_10788475_x3....................ucgauucccggcgccAgagc.......................................................................     1
Gi5_8652128_x3..................guucgauucccgUcgccgga.........................................................................     1
Gi3_27819824_x3.................gguuAgauucccggcgccgg..........................................................................     1
Gi1_4065504_x3..................guucAauucccggcgccg...........................................................................     1
Gi5_8655893_x3..................guucCauucccggcgccgg..........................................................................     1
Gi5_8653148_x3..................guucgauucccggcgcAggagc.......................................................................     1
Gi1_4048995_x3.................gguucgaCucccggcgccggagc.......................................................................     1
Gi2_10822114_x3.................gguuGgauucccggcgccgga.........................................................................     1
Gi2_10753012_x3................ggguucgauucccggcgccggagcC......................................................................     1
Gi1_4128985_x2............Cuccggguucgauucccggcgccgg..........................................................................     1
Gi3_28231734_x2.....................cgauucccggcgGcggagc.......................................................................     1
Gi3_28114184_x2.........cagauccggguucgauuUcc..................................................................................     1
Gi5_8808605_x2.ucacAcaacagauccggguu..........................................................................................     1
Gi5_8777695_x2.................gguucgauuccGggcgccg...........................................................................     1
Gi3_28086514_x2.........cagauccggguucgauCccc..................................................................................     1
Gi2_10975085_x2....................ucgauucccggcgccggG.........................................................................     1
Gi3_28062226_x2..................guucgauucccggcgGcggag........................................................................     1
Gi5_8800855_x2..................guucgauAcccggcgccggagc.......................................................................     1
G1a_5955404_x2...................uucgGuucccggcgccggagc.......................................................................     1
Gi2_10883591_x2....................ucgauucccggcgccUga.........................................................................     1
```

```
Gi2_10905121_x2.....................cgauucccgAcgccggagcguuu....................................................................    1
Gi1_4140307_x2..................guucgauucccgAcgccg............................................................................    1
Gi1_4147109_x2cucacccaacagauccgA..............................................................................................    1
Gi5_8808483_x2.................gguucgaGucccggcgccg............................................................................    1
Gi4_560646_x2 .................gguucgauucccUgcgccgg...........................................................................    1
Gi2_10932017_x2cucacccaacGgauccgg..............................................................................................    1
Gi3_28146306_x2.....................cgauucccggcgccggGgc........................................................................    1
Gi5_8759113_x2.....................cUauucccggcgccggagcguuuc...................................................................    1
Gi5_8718575_x2.................gguucgauucccggcgccggagG........................................................................    1
Gi1_4153047_x2..................guAcgauucccggcgccggagc........................................................................    1
Gi3_28144720_x2cucacccaacagaucUgg..............................................................................................    1
Gi5_8732521_x2.............uccggguucgGuucccggc................................................................................    1
Gi1_4111627_x2.................gguucgauucccggUgccg............................................................................    1
Gi2_10858823_x2..................guucgauucUcggcgccg............................................................................    1
Gi3_28057976_x2............auccUgguucgauucccggcgccgga..........................................................................    1
Gi5_8785603_x2....................ucgauucccgAcgccggag.........................................................................    1
Gi5_8729459_x2..................guucgauuccAggcgccggagc........................................................................    1
Gi3_28212126_x2......................gauucccgAcgccggagc........................................................................    1
Gi2_10832883_x2..................guucgauuGccggcgccgg...........................................................................    1
Gi5_8826375_x2..................Nuucgauucccggcgccgg...........................................................................    1
Gi2_10882565_x2......................gauucccggcgcUggagc........................................................................    1
Gi5_8812831_x2..................guucgauucccgAcgccggagc........................................................................    1
Gi1_4133053_x2.................ggGucgauucccggcgccg............................................................................    1
Gi2_10984659_x2..................guucgauucccggcgccggagG........................................................................    1
Gi3_28067814_x2.................gguucgauucccggcgGcggag.........................................................................    1
Gi5_8745705_x2.................gguucgauuAccggcgccggag.........................................................................    1
Gi5_8793103_x2.....................cgauucccggcgccggagcC.......................................................................    1
Gi2_10981085_x2..................guucgauucccggcgccUg...........................................................................    1
Gi5_8829199_x2....................Ccgauucccggcgccggag.........................................................................    1
G1a_5959402_x2.................ggCucgauucccggcgccg............................................................................    1
Gi1_4152497_x2cucaccUaacagauccgg..............................................................................................    1
Gi2_10908019_x2.................gguucgauuccAggcgccgg...........................................................................    1
Gi2_10838901_x2..................guucgauucccgAcgccggag.........................................................................    1
Gi5_8782019_x2.................gguucAauucccggcgccggag.........................................................................    1
Gi3_28079716_x2.................gguucgauucccggcgcGgg...........................................................................    1
Gi3_27984986_x2..........agauccggguucAauuccc...................................................................................    1
Gi3_27993194_x2.................gguucgaGucccggcgccggag.........................................................................    1
Gi3_28173668_x2...................uucgauucccggcgUcgga..........................................................................    1
Gi1_4114517_x2..................gAucgauucccggcgccggagc........................................................................    1
Gi4_565016_x2 cucacccaNcagauccggguucg.........................................................................................    1
Gi3_28023594_x2..Uacccaacagauccggguuc..........................................................................................    1
Gi3_28013354_x2...................uucgauucccAgcgccgga..........................................................................    1
Gi3_27989860_x2....cccaacagNuccggguucgauuccc...................................................................................    1
Gi5_8797229_x2.................gguucgauucccggcAcc.............................................................................    1
Gi2_10851903_x2.................gguAcgauucccggcgccgg...........................................................................    1
Gi2_10893279_x2..........agauccgggAucgauucc....................................................................................    1
Gi5_8719757_x2.................gguuUgauucccggcgccggagcgu......................................................................    1
```

```
Gi1_4119445_x2...................uucgauuccUggcgccgg..........................................................................      1
Gi1_4129275_x2.................gguucgauucccCgcgccggagc.......................................................................      1
Gi2_10911591_x2......................gauucccgUcgccggagc.......................................................................      1
Gi1_4147021_x2..................guucgauucccggcgcAg...........................................................................      1
Gi5_8754843_x2..........agUuccggguucgauuccc..................................................................................      1
Gi1_4106429_x2Aucacccaacagauccgg.............................................................................................      1
Gi2_10980421_x2..................guucgCuucccggcgccgga.........................................................................      1
Gi3_28154320_x2......caacagauccggguucgCuuccc..................................................................................      1
Gi5_8738921_x2..................guucgauucccggcgAcggagc.......................................................................      1
Gi3_28167666_x2.........cagauccggguucgauuccU..................................................................................      1
Gi3_28073120_x2.............uccggguucgauucccgAcgccgga.........................................................................      1
Gi1_4097929_x2..................guucgauucccggAgccgga.........................................................................      1
Gi5_8775529_x2cucacccUacagauccgg.............................................................................................      1
Gi3_28053262_x2...................Cucgauucccggcgccgga.........................................................................      1
Gi5_8772669_x2...................uucgauucccggcgccggagcC......................................................................      1
Gi1_4138577_x2..................guucgauuccUggcgccgga.........................................................................      1
Gi1_4106031_x2cucacccaNcagauccggguucga.......................................................................................      1
Gi1_4106033_x2.................gguucgauuccAggcgccgga.........................................................................      1
Gi4_564836_x2 ................gguucgauucccggcgccggCgc.......................................................................      1
Gi5_8789299_x2..................guucCauucccggcgccg...........................................................................      1
Gi2_10928843_x2...................uucgauucccggcgcAgg..........................................................................      1
Gi3_28146416_x2.................gguucgauucccggcgccggagcAu.....................................................................      1
Gi3_28174612_x2......................gauucccggcgccggagcCu.....................................................................      1
G1a_5925080_x2.................gguucgauucccAgcgccggagc.......................................................................      1
Gi5_8819591_x2.................gguucgauucccgAcgccggag........................................................................      1
Gi1_4125299_x2....................ucgauucccggcgccggaU........................................................................      1
Gi3_28027990_x2.....................cgauucccggcgccAgag........................................................................      1
G1a_5929114_x2.................gguucgauucccAgcgccggag........................................................................      1
Gi5_8757575_x2.................gguucgauucccUgcgccggag........................................................................      1
Gi2_10977695_x2..................guucgauucccggcgccggaA........................................................................      1
Gi5_8811325_x2..................guucgauuccGggcgccgg..........................................................................      1
Gi1_4141445_x2.................gguucgUuucccggcgccggagc.......................................................................      1
Gi5_8742179_x2.................gguucgauAcccggcgccggag........................................................................      1
Gi3_28016178_x2.....ccaacagauccggguucgauucccggcgccgga.........................................................................      0
Gi2_10861221_x2..................guucgauGcccggcgccggagc.......................................................................      1
G1a_5905292_x2.................gguucgauucccggcgccgC..........................................................................      1
Gi3_28096670_x2cuUacccaacagauccggguucgauu.....................................................................................      1
Gi3_28169190_x2...................uuUgauucccggcgccgga.........................................................................      1
G1a_5954434_x2..................gGucgauucccggcgccggag........................................................................      1
Gi3_28096924_x2..................guucgauucAcggcgccg...........................................................................      1
Gi5_8822577_x2.................gguucgauucAcggcgccg...........................................................................      1
Gi5_8757095_x2.....................Ggauucccggcgccggagcguuuc..................................................................      1
Gi3_28028530_x2cucacccaacagauccggguCcgauu.....................................................................................      1
Gi2_10991491_x2.................gguuAgauucccggcgccg...........................................................................      1
Gi1_4149379_x2.................gguucgauucccggcAccg...........................................................................      1
Gi1_4107127_x2.............Gccggguucgauucccggcgccgga.........................................................................      1
Gi2_10886403_x2..................guucgauAcccggcgccgga.........................................................................      1
```

```
Gi3_28084158_x2................Cgguucgauucccggcgccggag.........................................................................	1
Gi1_4148397_x2cucacccaacagCuccgg..............................................................................................	1
Gi3_28038396_x2....................ucgauucccggcgcGggagc........................................................................	1
Gi3_28134604_x2......cGacagauccggguucga........................................................................................	1
Gi5_8721603_x2....................uGgauucccggcgccggag.........................................................................	1
Gi3_28026106_x2..........Cgauccggguucgauuccc...................................................................................	1
Gi3_28141704_x2..................guucgauuccAggcgccggag.........................................................................	1
Gi3_28001584_x2Gucacccaacagauccggguucgauu......................................................................................	1
Gi5_8812521_x2..................guucgauucAcggcgccgga..........................................................................	1
Gi5_8745639_x2.................gguucgauucccggcgGcggagc........................................................................	1
Gi1_4129793_x2.................gguucgCuucccggcgccggag.........................................................................	1
Gi3_28218068_x2.................gguucgaAucccggcgccgg...........................................................................	1
Gi1_4134587_x2..................guucgauucccggcgccUga..........................................................................	1
G1a_5953700_x2..................guucgauucccggcgUcggagc........................................................................	1
Gi5_8812953_x2......................gaCucccggcgccggagc........................................................................	1
Gi5_8827803_x2......................gauucccAgcgccggagc........................................................................	1
Gi3_28194368_x2.................gguucgauucccggcgccggagcgC......................................................................	1
Gi5_8730235_x2....................ucgauuccNggcgccggagc........................................................................	1
Gi3_28210346_x2.................gguucgauucccggGgccgg...........................................................................	1
Gi1_4123847_x2......Gaacagauccggguucgauucccggc................................................................................	1
G1a_5934562_x2.................gguucUauucccggcgcc.............................................................................	1
G1a_5976424_x2.................gguucgCuucccggcgccg............................................................................	1
Gi2_10922353_x2.....................cgauucccggcgccggag.........................................................................	0
Gi2_10988729_x2.....................cgauuUccggcgccggagcguuuc...................................................................	1
Gi5_8745417_x2.................gguucgauucccgAcgccg............................................................................	1
Gi2_10840491_x2..................guucgauucccggAgccggag.........................................................................	1
Gi1_4095373_x2....................ucgauCcccggcgccggagc........................................................................	1
Gi1_4118757_x2...................Cucgauucccggcgccggagc........................................................................	1
Gi5_8817329_x2....cccaacGgauccggguuc..........................................................................................	1
Gi3_28241752_x2.........cagauccgAguucgauuccc...................................................................................	1
Gi3_28076800_x2..cacccaacagauccggguCc..........................................................................................	1
Gi1_4151871_x2................ggguucgaNucccggcgccgga..........................................................................	1
Gi3_28000816_x2..................guucgauucccggcgccgUa..........................................................................	1
Gi1_4134717_x2..................guucgauucccggUgccg............................................................................	1
Gi1_4123089_x2cucaGccaacagauccgg..............................................................................................	1
Gi3_28181902_x2.................gguucgauucccggcgccUg...........................................................................	1
Gi2_10964051_x2..................guucgauucccggcgccggUg.........................................................................	1
Gi2_10974063_x2.............uccggguuNgauucccggcgccgga..........................................................................	1
Gi5_8731855_x2........Gcagauccggguucgauucccggcgc..............................................................................	1
Gi2_10926921_x2..................guucgauucccggcgccCga..........................................................................	1
Gi4_554144_x2 cGcacccaacagauccggguucg..........................................................................................	1
Gi5_8808045_x2cucacccaacagauAcgg..............................................................................................	1
Gi3_27997574_x2......................gauucccggcgccggagU........................................................................	1
Gi3_28241054_x2...................uucgauucccggcgccAga..........................................................................	1
Gi5_8765215_x2..................guuNgauucccggcgccg............................................................................	1
Gi5_8763795_x2.................gguucgauucccgAcgcc.............................................................................	1
Gi1_4120257_x2................ggguucgauCcccggcgccgga..........................................................................	1
```

```
Gi3_28051586_x2....................ucgauucccggcgccggaA........................................................................     1
Gi2_10835117_x2....................ucgauCcccggcgccggagc.......................................................................     1
Gi3_28251356_x2.....................cgauCcccggcgccggagc.......................................................................     1
Gi3_28236582_x2..................guucgauCcccggcgccg...........................................................................     1
Gi3_28021150_x2..................gNucgauucccggcgccgga.........................................................................     1
Gi2_10836725_x2......................gauucccgNcgccggagc.......................................................................     1
Gi3_27980764_x2Uucacccaacagauccgg.............................................................................................     1
Gi5_8746429_x2cucacccaacagauUcgg.............................................................................................     1
Gi3_28162802_x2......................gauucAcggcgccggagc.......................................................................     1
Gi1_4153353_x2.................ggGucgauucccggcgccgga.........................................................................     1
Gi3_28199080_x2.................gguucgauucccggcgccUgag........................................................................     1
Gi3_28016184_x2.................Nguucgauucccggcgccgga.........................................................................     1
Gi3_28108166_x2....................ucgauucccggcgccggagcU......................................................................     1
Gi2_10831301_x2..................guucgauucAcggcgccgga.........................................................................     1
Gi2_10913593_x2..........agauccggguCcgauucc...................................................................................     1
G1a_5954188_x2..................guucgGuucccggcgccgg..........................................................................     1
Gi3_28094734_x2.ucacccaaNagauccggguuc.........................................................................................     1
Gi2_10890015_x2..................guGcgauucccggcgccggag........................................................................     1
G1a_5954946_x2.................gguucgGuucccggcgccgga.........................................................................     1
Gi1_4145965_x2cucacccaacagaucAgg.............................................................................................     1
Gi1_4153575_x2....................ucgauucccggcgccggCgc.......................................................................     1
Gi3_28194068_x2....................ucgauucccggcgAcggagc.......................................................................     1
Gi1_4138575_x2..................guucgauucAcggcgccgga.........................................................................     1
G1a_5965278_x2.................gguucgauucccggcgUcggagc.......................................................................     1
Gi3_28022676_x2...................uucgauucccggcgccggagcA......................................................................     1
Gi1_4152189_x2.................gguucgauucccUgcgccg...........................................................................     1
Gi1_4093929_x2..................guucUauucccggcgccg...........................................................................     1
G1a_5955874_x2.................gguucgauucccggcgccggCg........................................................................     1
Gi2_10960141_x2...................uucgauucccggcAccgga.........................................................................     1
Gi2_10854593_x2................Cgguucgauucccggcgccg...........................................................................     1
Gi3_28012736_x2.................ggNucgauucccggcgccg...........................................................................     1
Gi3_28178876_x2cAcacccaacagauccgg.............................................................................................     1
Gi5_8811253_x2.....................cgUuucccggcgccggagcguuuc..................................................................     1
Gi1_4148939_x2..................guGcgauucccggcgccgga.........................................................................     1
Gi3_28140802_x2.................gguucCauucccggcgccgga.........................................................................     1
Gi3_28002086_x2..................guucgauucccggGgccggag........................................................................     1
Gi2_10886137_x2.................gAuucgauucccggcgccgg..........................................................................     1
G1a_5974960_x2.................ggCucgauucccggcgccggag........................................................................     1
Gi1_4106883_x2cucacccaCcagauccgg.............................................................................................     1
Gi1_4150583_x2..................guucgauucccgAcgccgga.........................................................................     1
G1a_5889316_x2..................guucgaCucccggcgccgg..........................................................................     1
Gi5_8756105_x2...........Uauccggguucgauucccggcgccgga.........................................................................     1
Gi5_8791119_x2..............Gcggguucgauucccggcgccgga.........................................................................     1
Gi2_10873443_x2.................gguucgUuucccggcgccg...........................................................................     1
Gi1_4123129_x2..cacccaacNgauccggguucgauuccc..................................................................................     1
Gi3_28198142_x2...................uucgauucccggGgccgga.........................................................................     1
Gi3_28117386_x2.................gguucgauucccggcUccg...........................................................................     1
```

```
Gi1_4147217_x2..................guucgauucccggUgccggagc........................................................................     1
Gi3_28017590_x2...........gauccgggNucgauuccc...................................................................................     1
G1a_5966336_x2.................gguucgauucccggcgccAgagc........................................................................     1
Gi4_554608_x2 ..................guucgaCucccggcgccgg...........................................................................     1
Gi5_8770651_x2..................guucgauucccggcgccgAagc........................................................................     1
Gi2_10947541_x2....Uccaacagauccggguuc..........................................................................................     1
Gi2_10964813_x2.ucacccaacagauccggguu...........................................................................................     0
Gi3_28119816_x2...................Cucgauucccggcgccggagc........................................................................     1
Gi2_10924103_x2...................uucgauucNcggcgccggagc........................................................................     1
Gi3_28247670_x2.........cagauccAgguucgauuccc...................................................................................     1
Gi1_4156275_x2.ucacccaacagaCccgggu............................................................................................     1
Gi2_10952571_x2..................guucgauucccggcgccggaUc........................................................................     1
Gi3_28150320_x2................ggguucgauucccggcgccggaA.........................................................................     1
Gi3_28229644_x2.....................cgauucccggcgccggagcCu......................................................................     1
Gi1_4121371_x2.................gguucgauucccggcgccAgag.........................................................................     1
Gi4_558418_x2 .................gguucgauucccggAgccgg...........................................................................     1
Gi5_8765217_x2..................guucgauucccgCcgccg............................................................................     1
Gi5_8719451_x2.................gguucgauucccggcgGcgga..........................................................................     1
Gi4_557714_x2 ..................guucgauCcccggcgccgg...........................................................................     1
Gi3_28078074_x2..................guucgauuGccggcgccgg...........................................................................     1
Gi3_27994084_x2...................uucgCuucccggcgccgga..........................................................................     1
Gi5_8798343_x2.................gguucgauuUccggcgccgga..........................................................................     1
Gi3_28107384_x2....................ucgauucccggcCccggagc........................................................................     1
Gi3_28018856_x2..................guucgaAucccggcgccgg...........................................................................     1
Gi5_8764685_x2..................guucgauAcccggcgccgga..........................................................................     1
Gi2_10830241_x2.................gguucgauucccggcUccggag.........................................................................     1
Gi1_4118337_x2..caUccaacagauccggguucgauuccc...................................................................................     1
Gi2_10973233_x2..................guucgauucccggGgccgg...........................................................................     1
G1a_5894674_x2.................gguucgauucccggcgUc.............................................................................     1
Gi3_28229778_x2................gggGucgauucccggcgccggagc........................................................................     1
Gi3_28130614_x2..........agauccggguucgCuuccc...................................................................................     1
Gi5_8763973_x2.................gguucgauucccggcgccggagcAu......................................................................     1
Gi5_8724119_x2.................Nguucgauucccggcgccggagc........................................................................     1
Gi5_8780981_x2.................gguucgauucccggcgGcgg...........................................................................     1
Gi3_28185408_x2...................uucgauucUcggcgccgg...........................................................................     1
Gi1_4135867_x2.................Cguucgauucccggcgccgga..........................................................................     1
Gi2_10869407_x2..................guucCauucccggcgccggagc........................................................................     1
Gi3_28051810_x2.................gguucgauucccggcgccUga..........................................................................     1
Gi5_8803327_x2..........agaucUggguucgauuccc...................................................................................     1
Gi1_4120567_x2..................guucgauucccggcgcAgga..........................................................................     1
Gi3_28204358_x2.....................cgaCucccggcgccggagc........................................................................     1
Gi2_10952191_x2.................gguucgauucccggcgccCg...........................................................................     1
Gi3_28149672_x2................gggGucgauucccggcgccg............................................................................     1
Gi4_556180_x2 ..................Auucgauucccggcgccgg...........................................................................     1
Gi1_4145737_x2...............cggguucgauucccggcgccgg...........................................................................     0
Gi2_10831105_x2...................uGcgauucccggcgccgga..........................................................................     1
Gi5_8730009_x2.................gguucgauAcccggcgccgg...........................................................................     1
```

```
Gi3_27984222_x2..................guucgauucccggcgccggaU..........................................................................     1
Gi5_8826953_x2....................uGgauucccggcgccggagc.........................................................................     1
Gi5_8762963_x2.................gguucgauucccggcgcAgga...........................................................................     1
Gi3_28169138_x2...................uAcgauucccggcgccggagc.........................................................................     1
Gi4_560504_x2 .................gguCcgauucccggcgccggagc.........................................................................     1
Gi2_10935317_x2..................guucgauucccggcgccgAag..........................................................................     1
Gi3_28154844_x2...............cggguucgauucccAgcgccgga...........................................................................     1
Gi3_28204134_x2....................ucgauucccggcgccggagU.........................................................................     1
Gi3_28152912_x2.................gguucgauucccCgcgccggag..........................................................................     1
Gi1_4103481_x2.................gguucgauucccggcgcU..............................................................................     1
Gi2_10912907_x2.................gguNcgauucccggcgcc..............................................................................     1
Gi5_8754887_x2...................uucgaCucccggcgccgg............................................................................     1
Gi1_4091359_x2..................Cuucgauucccggcgccggagc.........................................................................     1
Gi2_10919851_x2......................gauucccggcgccggagcCu.......................................................................     1
G1a_5966242_x2...................uucgaCucccggcgccggagc.........................................................................     1
Gi1_4153439_x2.................gguucgauucccgAcgccgga...........................................................................     1
Gi5_8748139_x2.........Gagauccggguucgauucccggcgccg.............................................................................     1
Gi2_10914825_x2....................uUgauucccggcgccggag..........................................................................     1
Gi3_28160534_x2...................uucgGuucccggcgccggagc.........................................................................     1
Gi3_28140736_x2....................ucgaGucccggcgccggag..........................................................................     1
Gi2_10869631_x2.................gguucgauucccggGgccggag..........................................................................     1
Gi3_28226426_x2...................uucgauucccggcgGcgga...........................................................................     1
Gi3_28236300_x2............Guccggguucgauucccggcgccggagc.........................................................................     1
Gi2_10925471_x2.................gguucgauucccggcgccCga...........................................................................     1
G1a_5952986_x2..................guucgauucccggcgccggagU.........................................................................     1
G1a_5909814_x2.................gguucgauucccggcgccggC...........................................................................     1
Gi3_28141006_x2................ggguCcgauucccggcgccg.............................................................................     1
Gi5_8766929_x2.........cagauccgggGucgauucccggcgccg.............................................................................     1
Gi3_28053326_x2.................gguucgauucccgCcgccgga...........................................................................     1
Gi2_10976811_x2.................gguucgauucccggcgccUga...........................................................................     1
Gi2_10890847_x2..................guucgauucccggcgccCgag..........................................................................     1
Gi5_8810763_x2.................gguuGgauucccggcgccgg............................................................................     1
Gi1_4135311_x2.................gguucgauNcccggcgcc..............................................................................     1
Gi3_28166684_x2..................guucgauucccgUcgccg.............................................................................     1
Gi5_8785053_x2.................gguuGgauucccggcgccgga...........................................................................     1
Gi2_10922863_x2..................guucgauAcccggcgccg.............................................................................     1
Gi2_10921797_x2...................uuUgauucccggcgccgga...........................................................................     1
Gi5_8752639_x2.................ggGucgauucccggcgccggagcguu......................................................................     1
Gi1_4095171_x2.................gguucgauuccUggcgccggagc.........................................................................     1
Gi1_4106855_x2.................gguucgauucccggcgAcg.............................................................................     1
Gi1_4158449_x2.................gguucgauCcccggcgccgga...........................................................................     1
G1a_5936950_x2.................Cguucgauucccggcgccgga...........................................................................     1
Gi1_4142901_x2.................gguuUgauucccggcgccgga...........................................................................     1
Gi3_28175422_x2...................uucgauucccggcgcUggagc.........................................................................     1
Gi3_28108420_x2.........cagauccgggCucgauuccc....................................................................................     1
Gi3_28254678_x2.....................Ugauucccggcgccggagc.........................................................................     1
Gi2_10990005_x2.................gguucgauuccGggcgcc..............................................................................     1
```

```
Gi3_28180718_x2..........aCauccggguucgauucc....................................................................................    1
Gi2_10897531_x2..................guucgauAcccggcgccggag.........................................................................    1
Gi5_8774561_x2.................gguucgauNcccggcgccggagcgu......................................................................    1
Gi3_28224872_x2...................uucAauucccggcgccggagc........................................................................    1
Gi3_28083252_x2..................guucgauucAcggcgccggag.........................................................................    1
Gi5_8739787_x2..................guuGgauucccggcgccgg...........................................................................    1
Gi2_10969409_x2..................guuGgauucccggcgccgg...........................................................................    1
Gi5_8820787_x2.................gguucgauucccggcgUcggag.........................................................................    1
Gi1_4104675_x2.................gguucgauucccggcgccggCg.........................................................................    1
Gi5_8824933_x2.................gguucgauCcccggcgccgg...........................................................................    1
Gi5_8730799_x2..................guucgauucGcggcgccg............................................................................    1
Gi3_28014692_x2..cacccaacNgauccggguuc..........................................................................................    1
Gi3_28133342_x2....................ucgauucccggcgccgCagc........................................................................    1
Gi4_560328_x2 cuUacccaacagauccggguucg.........................................................................................    1
Gi4_564144_x2 ..................guucgauucccggcgccggagcA.......................................................................    1
Gi5_8718507_x2..................guucgauucccggcgccggagA........................................................................    1
G1a_5938360_x2.................gguNcgauucccggcgccg............................................................................    1
Gi5_8741773_x2...................uucgauucccggcgccggagc........................................................................    0
Gi3_28150636_x2......................gaGucccggcgccggagc........................................................................    1
Gi1_4131383_x2..................guucgauucccggcgAcggagc........................................................................    1
Gi3_28247674_x2...................uucgauucccggcgccgAa..........................................................................    1
Gi3_28130308_x2.....................cCauucccggcgccggagc........................................................................    1
Gi5_8731359_x2.................gguucgauucccggcgccC............................................................................    1
Gi1_4099341_x2.................gguucgauuccUggcgccgga..........................................................................    1
Gi5_8790799_x2..................guucUauucccggcgccggag.........................................................................    1
Gi2_10883617_x2.................ggAucgauucccggcgccgg...........................................................................    1
Gi1_4101425_x2.................gguucgauucccggcgGcgg...........................................................................    1
Gi1_4107525_x2...................uCcgauucccggcgccgg...........................................................................    1
Gi1_4155703_x2..................guucgauuUccggcgccgga..........................................................................    1
Gi3_28166900_x2.....................cgauucAcggcgccggagc........................................................................    1
Gi2_10871971_x2..................guucgauucccggcgccgCag.........................................................................    1
Gi3_28174380_x2....................ucgauucccggcAccgga..........................................................................    1
Gi2_10940445_x2..................guuNgauucccggcgccgg...........................................................................    1
Gi3_28115858_x2.............uUcggguucgauucccggcgccgga..........................................................................    1
Gi5_8801793_x2.................ggCucgauucccggcgccggagcguu.....................................................................    1
Gi2_10904063_x2......................gauucccggUgccggagc........................................................................    1
Gi4_559956_x2 ..................guucgauucccggcgUcgg...........................................................................    1
Gi5_8795627_x2......caacagauccggguucgauucccggcgccggagc........................................................................    0
Gi1_4143387_x2.................gguuGgauucccggcgccg............................................................................    1
Gi1_4156181_x2..................guucgauucccggcgccUgagc........................................................................    1
Gi2_10869777_x2..................guucgauucGcggcgccggagc........................................................................    1
Gi5_8822741_x2..................guucgauucccggcgccgCa..........................................................................    1
Gi2_10906983_x2.................gguucgauucccggcUcc.............................................................................    1
Gi3_27992666_x2..................guucgauucccggcgccggagcAu......................................................................    1
Gi3_28128564_x2...................uucgauucccggcgccggag.........................................................................    0
Gi3_28091478_x2cucacccaacCgauccgg..............................................................................................    1
Gi3_28132424_x2...........gauccggguucgGuccc....................................................................................    1
```

Gi5_8746507_x2..................guucgauucccggcCccggagc.. 1
Gi5_8753995_x2..................guucgauucccgCcgccgg... 1
G1a_5885002_x2.................ggAucgauucccggcgccggagc.. 1
Gi3_28006938_x2...............cAgguucgauucccggcgccgga.. 1
Gi3_28147408_x2.........cagGuccggguucgauuccc... 1
Gi1_4141471_x2..................guucgauucccggcgccggagA.. 1
Gi5_8808217_x2.................gguucgauucccggcgAcggag... 1
Gi1_4148503_x2..................Uuucgauucccggcgccggag... 1
Gi1_4142627_x2.................gguucgauucccAgcgccgga.. 1
Gi3_28234392_x2.................gguucgauucccggcgccggaU... 1
Gi1_4108265_x2.................gCuucgauucccggcgccggagc.. 1
Gi3_28174226_x2......................gauucccggcgccgAagc.. 1
Gi1_4132611_x2....................ucgGuucccggcgccggagc.. 1
Gi3_28239860_x2...........gauccggguucgaCuccc... 1
Gi5_8743865_x2......................Aauucccggcgccggagc.. 1
Gi5_8784921_x2..................guucgauucccggcgccggagcgC.. 1
Gi1_4118663_x2.................gguucgauucccggcAccgga.. 1
Gi3_28079074_x2....................ucgauuccAggcgccgga.. 1
Gi2_10888181_x2.................gguucgUuucccggcgccgga.. 1
Gi3_28147456_x2...................uuUgauucccggcgccgg... 1
Gi3_28168954_x2..........agauccggguucgauCccc... 1
Gi5_8738045_x2....................uUgauucccggcgccggagc.. 1
Gi2_10853421_x2...............cggguucgauucccggcgccggagc.. 0
Gi5_8779869_x2.................gguucgGuucccggcgcc... 1
Gi1_4100149_x2cAcacccaacagauccgg.. 1
Gi3_28164796_x2.................gguucgauuccGggcgcc... 1
Gi5_8735137_x2..................guucgauucccggcAccggagc.. 1
Gi5_8723867_x2.................gguucgauucccgCcgccggagc.. 1
Gi1_4151121_x2.ucacccaaNagauccgggu.. 1
Gi3_28147466_x2....................ucgauucccggcgcUggagc.. 1
Gi1_4154003_x2..................guucgauAcccggcgccggagc.. 1
Gi3_28078132_x2......................gauAcccggcgccggagc.. 1
G1a_5911486_x2.................gguucgauucccggcgccggUgc.. 1
Gi2_10846981_x2....................ucgauucccggcAccggagc.. 1
Gi2_10929055_x2..................guucgauucccggcgAcggag... 1
Gi2_10950493_x2.................gguucgauucccggGgccgg... 1
Gi3_28048188_x2.................gguucCauucccggcgccgg... 1
Gi3_28233852_x2................gUguucgauucccggcgccg.. 1
Gi1_4142341_x2..................guucgauucccggcgccAgag... 1
Gi1_4145897_x2...acccaacaNauccggguucgauucccg.. 1
Gi3_28104480_x2...................uucgauucccAgcgccggagc.. 1
Gi3_28231186_x2....................ucgauAcccggcgccggagc.. 1
Gi2_10847523_x2..................guucgauucccgCcgccggagc.. 1
Gi5_8801251_x2........................uucccggcgccggagcCu.. 1
G1a_5921074_x2.................gguucgauucccggcgccggagA.. 1
Gi3_28230940_x2....................ucgauucccgCcgccggagc.. 1
Gi3_28077110_x2....................ucgauuAccggcgccgga.. 1

```
Gi3_28040844_x2....................ucgauucccggcgccggaAc.......................................................................	1
Gi1_4094027_x2.................gguucgauucccggcgccU...........................................................................	1
Gi5_8744671_x2..................guucgauucccggcgAcgga.........................................................................	1
Gi2_10948143_x2................Cgguucgauucccggcgccggagc.......................................................................	1
Gi3_28139806_x2.......Gacagauccggguucgauucccggcgccgga.........................................................................	1
Gi3_28217236_x2...................uucgauucccggAgccgga.........................................................................	1
Gi3_28052776_x2cucacccaaUagauccgg.............................................................................................	1
Gi1_4118677_x2..................guucgauucccgUcgccg...........................................................................	1
G1a_5930474_x2.................gguucgauucccggcgAc............................................................................	1
Gi3_28164826_x2..................guucgauucGcggcgccgga.........................................................................	1
Gi2_10948229_x2.......................auucccggcgccggagcgA.....................................................................	1
G1a_5882038_x2..................Auucgauucccggcgccggagc.......................................................................	1
Gi4_556122_x2 ..................guucgauuNccggcgccgg..........................................................................	1
Gi3_28065536_x2..........agaucUggguucgauuccc..................................................................................	1
Gi2_10924427_x2..................guucgCuucccggcgccg...........................................................................	1
Gi2_10991033_x2.................gguuGgauucccggcgcc............................................................................	1
Gi1_4127477_x2...................uucgauucccAgcgccgg..........................................................................	1
Gi1_4139407_x2.................gguucgauucccggcgUcgg..........................................................................	1
Gi2_10918135_x2.................gguucgauucccggcgUcgg..........................................................................	1
Gi1_4140105_x2.....................cgauucccggcgccggagcC......................................................................	1
Gi3_28005272_x2..................guucgauucccggcUccgg..........................................................................	1
Gi2_10833539_x2..................guucgauucUcggcgccgga.........................................................................	1
Gi5_8754285_x2.................gguucgauucccggGgccg...........................................................................	1
Gi1_4148309_x2......caGcagauccggguucgauu.....................................................................................	1
Gi3_28024186_x2.................gguucgauucccUgcgccgg..........................................................................	1
Gi1_4100751_x2.................gguucgauucccggcgccgAa.........................................................................	1
Gi1_4100873_x2..................guucgauucccggcAccggagc.......................................................................	1
Gi3_28041284_x2.....................cgauucccggcgccggaAc.......................................................................	1
Gi3_28203820_x2.....................cgauucccggcgcGggagc.......................................................................	1
Gi2_10980637_x2.............uccggguucgauucccggc...............................................................................	0
G1a_5913426_x2...................Gucgauucccggcgccggagc.......................................................................	1
Gi3_28171068_x2.................gguucgauucccggcgGcg...........................................................................	1
Gi2_10947365_x2..................guucgauuAccggcgccggag........................................................................	1
Gi3_28150738_x2..................guuAgauucccggcgccggag........................................................................	1
Gi4_553470_x2 ..................guucgauucccggcgccAg..........................................................................	1
Gi3_28089238_x2...................uucgauucAcggcgccgga.........................................................................	1
Gi3_28051960_x2.................gguucgauucUcggcgcc............................................................................	1
Gi2_10897681_x2...................uuUgauucccggcgccggag........................................................................	1
Gi1_4131255_x2...................uucgauucccggcgccggCgc.......................................................................	1
Gi3_28239946_x2....................ucgauucccggcgccggC.........................................................................	1
Gi5_8789659_x2..................guucgaAucccggcgccgga.........................................................................	1
Gi2_10978487_x2......................gaAucccggcgccggagc.......................................................................	1
Gi2_10858249_x2...................uucgauucccggcgccggCg........................................................................	1
Gi2_10868905_x2.................gguucgauucccggcgccggU.........................................................................	1
G1a_5911236_x2...................uGcgauucccggcgccggagc.......................................................................	1
Gi5_8772665_x2..........agauccggguucgCuuccc..................................................................................	1
Gi1_4116277_x2...................uucgauucccggcgccggaU........................................................................	1
```

```
Gi2_10922609_x2......................gauucccggcgccggagU........................................................................    1
Gi2_10831947_x2...................Gucgauucccggcgccgga..........................................................................    1
Gi2_10959617_x2......................gauucccggcgccggUgc........................................................................    1
Gi3_28254224_x2..................guucgaGucccggcgccg............................................................................    1
G1a_5904370_x2..................gCucgauucccggcgccgg...........................................................................    1
Gi2_10853099_x2....................ucgauucccgCcgccggag.........................................................................    1
Gi3_28063662_x2cGcacccaacagauccggguucgauu......................................................................................    1
Gi2_10868073_x2..................guucgauuccAggcgccggag.........................................................................    1
Gi1_4138259_x2.................gguucgauucccUgcgccgga..........................................................................    1
Gi2_10945881_x2...................uucgaCucccggcgccgg...........................................................................    1
G1a_5930724_x2.................ggGucgauucccggcgccggagc........................................................................    1
G1a_5899828_x2.................gguuUgauucccggcgccgg...........................................................................    1
Gi5_8718193_x2.......................auucccggcgccggagcA.......................................................................    1
Gi5_8823847_x2...acccaacaNauccggguucgauuccc...................................................................................    1
Gi2_10856589_x2.....................cgauucccggcgccggagcgCuuc...................................................................    1
Gi2_10940653_x2...................Cucgauucccggcgccggagc........................................................................    1
Gi1_4153405_x2..........agauccgggCucgauuccc...................................................................................    1
Gi1_4118443_x2...................uucgGuucccggcgccgg...........................................................................    1
Gi3_28135542_x2..................guucgauucGcggcgccgg...........................................................................    1
Gi3_28098530_x2.....................cgauucccggcgccggGg.........................................................................    1
Gi5_8732703_x2.................gguucgauucccggcgcAgg...........................................................................    1
G1a_5897474_x2..................guucgauucccggcgcUggagc........................................................................    1
Gi1_4112609_x2.................gguucgauucccggcgUcggagc........................................................................    1
Gi5_8802831_x2.................gguuAgauucccggcgccg............................................................................    1
Gi5_8782065_x2...................uucgauucccggcgccggagcCu......................................................................    1
Gi5_8728773_x2..................guucgauucccggcgcGgg...........................................................................    1
Gi5_8723713_x2......................gUuucccggcgccggagc........................................................................    1
Gi3_28190458_x2..................guucgauucccggcgcGgg...........................................................................    1
Gi5_8823089_x2.............uUcggguucgauucccggc................................................................................    1
Gi2_10984877_x2....cccaacGgauccggguuc..........................................................................................    1
Gi5_8745723_x2.................gguucgauAcccggcgcc.............................................................................    1
Gi3_28160030_x2........Gcagauccggguucgauuccc...................................................................................    1
Gi2_10951235_x2....................ucgauucccggcgccggagU........................................................................    1
Gi3_28145154_x2...............cggguucgGuucccggcgccgga..........................................................................    1
Gi3_28175786_x2..........Ggauccggguucgauuccc...................................................................................    1
G1a_5977690_x2.................gguucgauNcccggcgccgga..........................................................................    1
Gi5_8748009_x2.ucacccaacagGuccggguu...........................................................................................    1
Gi3_28243286_x2....................ucgauucccggcgcAgga..........................................................................    1
Gi5_8747675_x2.................gguucgauucAcggcgccgg...........................................................................    1
Gi3_28030324_x2....................ucgauucccggcgccggagcgA......................................................................    1
Gi1_4145445_x2...................uucgauucUcggcgccgg...........................................................................    1
Gi5_8789839_x2...............cggguucgauucccggUgcc.............................................................................    1
Gi1_4124623_x2cucacccaacagUuccgg..............................................................................................    1
Gi3_28071682_x2.................gguGcgauucccggcgccg............................................................................    1
Gi3_28133844_x2..................gNucgauucccggcgccggagc........................................................................    1
Gi2_10884795_x2....................ucgauucccggcgAcggagc........................................................................    1
Gi2_10852143_x2.....................cgauucccggcgccggGgcguuu....................................................................    1
```

```
Gi1_4122019_x2...................uucgauucccggcgccgga..........................................................................     0
Gi3_28146992_x2......caacagauccggguucgauuccA...................................................................................    1
Gi2_10872295_x2..................gCuucgauucccggcgccgg..........................................................................    1
G1a_5951118_x2.................gguucgauucccggAgccggagc........................................................................     1
G1a_5946596_x2.................gguucgauucccggcgcAg............................................................................     1
Gi2_10830341_x2.................ggGucgauucccggcgccgg...........................................................................    1
Gi1_4092719_x2.................gguucgauucccggcgccUg...........................................................................     1
Gi2_10858231_x2.............uccgAguucgauucccggcgccgga..........................................................................    1
Gi2_10973467_x2......caacagauccggguucgauuccc...................................................................................    0
Gi1_4143113_x2...................uucgauucNcggcgccggagc........................................................................     1
G1a_5900034_x2.................Aguucgauucccggcgccgga..........................................................................     1
Gi5_8734655_x2.................gguucgauucccggcgccgAag.........................................................................     1
Gi3_28056430_x2...................uucgGuucccggcgccgg...........................................................................    1
Gi2_10965185_x2....................ucgGuucccggcgccggagc........................................................................    1
Gi3_28034016_x2.................gguucgUuucccggcgcc.............................................................................    1
Gi3_28059142_x2.....................cgauucccAgcgccggag.........................................................................    1
Gi3_28211802_x2............auccggguucgauucccggcg...............................................................................    0
Gi3_28028268_x2...................uuGgauucccggcgccgg...........................................................................    1
Gi1_4096007_x2.Gcacccaacagauccggguucga........................................................................................     1
Gi3_28253028_x2................gggCucgauucccggcgccggag.........................................................................    1
Gi4_564000_x2 ..................gCucgauucccggcgccgga..........................................................................     1
Gi2_10850305_x2..................guucgaGucccggcgccg............................................................................    1
Gi3_28162628_x2.................gguuGgauucccggcgcc.............................................................................    1
Gi3_28257636_x2..................guuNgauucccggcgccg............................................................................    1
Gi3_28128796_x2cucacccaacagaCccgg..............................................................................................    1
Gi3_28038154_x2......caacagauccggUuucga........................................................................................    1
Gi5_8737139_x2.................gguucgauucccggcgccgCagc........................................................................     1
Gi4_562044_x2 .................ggCucgauucccggcgccgg...........................................................................     1
Gi5_8788193_x2.................gguuAgauucccggcgccgga..........................................................................     1
Gi1_4114789_x2.................gguuUgauucccggcgccggagc........................................................................     1
Gi1_4118461_x2.................ggAucgauucccggcgccgga..........................................................................     1
Gi3_28236100_x2......caacagauccggguucAauuccc...................................................................................    1
Gi1_4127809_x2..................guucgauucUcggcgccgga..........................................................................     1
Gi2_10893647_x2.................gguucgaAucccggcgccgga..........................................................................    1
Gi3_28257730_x2.................gguGcgauucccggcgccgga..........................................................................    1
Gi4_553262_x2 .................gguucgauNcccggcgccggagc........................................................................     1
Gi3_28149006_x2Aucacccaacagauccgg..............................................................................................    1
Gi5_8731585_x2..................guucgaAucccggcgccggagc........................................................................     1
Gi3_28086048_x2.....................cgauucccggAgccggagc........................................................................    1
Gi2_10955977_x2...................uucgaCucccggcgccgga..........................................................................    1
Gi2_10982175_x2...................Cucgauucccggcgccgg...........................................................................    1
Gi1_4091203_x2..................guucgauucccggcCccgga..........................................................................     1
Gi3_28096882_x2..................guucgauucccggcgccUgag.........................................................................    1
Gi5_8759301_x2..................guucgauucccUgcgccg............................................................................     1
Gi5_8722591_x2....................ucgauucccggcgccggCgc........................................................................     1
Gi3_27997186_x2.........cagauccggguucgauucUc...................................................................................    1
Gi1_4125441_x2..................guucgauucccggcgGcgga..........................................................................     1
```

```
Gi1_4143699_x2..........agauccggguucgauucc......................................................................................      0
Gi5_8770111_x2..................guucUauucccggcgccggagc..........................................................................      1
Gi1_4129393_x2............auUcggguucgauucccggcgccgga............................................................................      1
Gi2_10973301_x2..................guucgauucccggcgGcg..............................................................................      1
Gi1_4118543_x2..................guucgauucccggcgccC..............................................................................      1
Gi4_555552_x2 ..................Uuucgauucccggcgccg..............................................................................      1
Gi2_10849895_x2..........agauccggNuucgauucc......................................................................................      1
Gi1_4110277_x2.................gguucgauucccggcgccggC............................................................................      1
Gi3_28207296_x2.................gguNcgauucccggcgccggag...........................................................................      1
Gi3_27995030_x2......................gauucccggcgccgUagc..........................................................................      1
Gi3_28240662_x2.................gguNcgauucccggcgccgg.............................................................................      1
Gi3_28146482_x2..................guucgauucccUgcgccg..............................................................................      1
Gi5_8766899_x2...................gCucgauucccggcgccggag..........................................................................      1
Gi1_4101387_x2.................gguucgauucccggcgccggaA...........................................................................      1
G1a_5898620_x2.................gguucgauucccUgcgccggagc..........................................................................      1
Gi5_8950806_x1..................guucgauuUccggcgccggagc..........................................................................      1
Gi5_8916664_x1..cacccaacagauccAgguucga..........................................................................................      1
Gi2_11323640_x1................gUguucgauucccggcgccggag...........................................................................      1
Gi1_4174174_x1......caacagUccggguucgauucccggc...................................................................................      1
Gi5_8868571_x1..................guuNgauucccggcgccgga............................................................................      1
Gi1_4325900_x1......caacagGuccggguucgauucccggc..................................................................................      1
Gi2_11157373_x1..........agauccgggCucgauucc......................................................................................      1
Gi1_4244347_x1......................Uauucccggcgccggagc..........................................................................      1
Gi2_11031798_x1.................gguucgauucccggcgcG...............................................................................      1
Gi2_11338905_x1.................gguucgauucccggcgcGg..............................................................................      1
G1a_6027245_x1.................gguuAgauucccggcgccg..............................................................................      1
Gi3_28607525_x1................ggguucgauucccggcgAcggagc..........................................................................      1
Gi3_28525479_x1.......aGcagauccggguucgauucc......................................................................................      1
Gi3_28920241_x1......caacagauccggguucgGuuccc.....................................................................................      1
Gi3_28596304_x1...................uucgauucccggcgccggUgc..........................................................................      1
Gi2_11158769_x1...................uucgauucccggcgccAga............................................................................      1
Gi2_11337343_x1.................gguucgauucGcggcgcc...............................................................................      1
Gi3_28519263_x1.........cagauccggguucgauucccggcAccgg.............................................................................      1
Gi5_9138174_x1................ggguucgauucccggcgAcg..............................................................................      1
Gi3_28979015_x1...................Gucgauucccggcgccggag...........................................................................      1
Gi1_4323646_x1..................Nuucgauucccggcgccgga............................................................................      1
Gi3_28321592_x1.............Accggguucgauucccggcgccgga............................................................................      1
Gi1_4352148_x1.................gguucgauCcccggcgccg..............................................................................      1
Gi5_9075623_x1..................guucgaCucccggcgccggagcgu........................................................................      1
Gi2_11354537_x1....................ucgauuccAggcgccggagc..........................................................................      1
Gi1_4274271_x1...................uucgauucccggcgccggagcgA........................................................................      1
G1a_6038980_x1.................gguucgauucccggUgccggagc..........................................................................      1
Gi3_28923891_x1..........Ugauccggguucgauuccc.....................................................................................      1
Gi3_28548837_x1...............cgggCucgauucccggcgccgga............................................................................      1
Gi5_8834483_x1.................gguucgauucccggGgccggag...........................................................................      1
Gi1_4167359_x1................gUguucgauucccggcgccgg.............................................................................      1
Gi3_28845590_x1................ggguuUgauucccggcgccg..............................................................................      1
```

```
Gi2_11400291_x1.............uccgggCucgauucccggcgccgga...........................................................................     1
Gi4_576983_x1 ..................guucAauucccggcgccgg............................................................................     1
Gi5_9026824_x1..........agauAcggguucgauuccc....................................................................................     1
Gi1_4366842_x1cGcacccaacagauccggguucga.........................................................................................     1
Gi5_8842286_x1.................gguucgauuccAggcgccggagcguu......................................................................     1
Gi5_8887075_x1.................gguucgUuucccggcgccgg............................................................................     1
Gi5_8960424_x1.................gguucgauucAcggcgcc..............................................................................     1
Gi3_28281794_x1................ggguucgauuccUggcgccgga...........................................................................     1
Gi2_11193403_x1....cccaacagCuccggguuc...........................................................................................     1
Gi3_28870627_x1..................guucCauucccggcgccggagc.........................................................................     1
Gi3_28516877_x1..........agauccggguCcgauucccggcgccggag..........................................................................     1
Gi3_28488094_x1.........Gagauccggguucgauucccggcgccgg............................................................................     1
Gi2_11144500_x1..................guucgauucccggGgccggag..........................................................................     1
Gi3_28668874_x1......caacagauccggguucgauucccggcgccggagc.........................................................................     0
Gi3_28552813_x1..................guuNgauucccggcgccggag..........................................................................     1
G1a_6330265_x1.................gguucgauucccggcgcUgga...........................................................................     1
Gi2_11263507_x1.................gguucUauucccggcgccg.............................................................................     1
Gi5_8941812_x1.................gguucgauucccggcgccggagcguuC.....................................................................     1
Gi1_4192941_x1......Aaacagauccggguucgauucccggc.................................................................................     1
G1a_6343388_x1.................gguucUauucccggcgccg.............................................................................     1
Gi5_9018245_x1.................gguucgauucccggcgccAgagcguu......................................................................     1
Gi3_28440405_x1.....................cgNuucccggcgccggagc.........................................................................     1
Gi2_11201441_x1..................guucgauucccggcgccgUag..........................................................................     1
Gi3_28500151_x1................ggguucgauucccggcgA...............................................................................     1
G1a_6330986_x1..................guucgauucccCgcgccg.............................................................................     1
Gi3_28771493_x1......caGcagauccggguucgauuccc....................................................................................     1
Gi4_597138_x1 ..................guucgauucccggUgccgga...........................................................................     1
Gi2_11361890_x1..............ccggguucgauucccggcgUcgga...........................................................................     1
Gi3_28625165_x1cucacccaacagauUcgg...............................................................................................     1
Gi2_11235190_x1..................guucgauucccggcgccUgag..........................................................................     1
Gi1_4224993_x1cucacccaacagauccggguucg..........................................................................................     0
Gi3_28819375_x1..........agauccgggCucgauuccc....................................................................................     1
Gi3_28766235_x1......caacagauccggguucgC.........................................................................................     1
Gi3_28311531_x1.........Aagauccggguucgauuccc....................................................................................     1
Gi3_28336392_x1....cccaacCgauccggguucgauuccc....................................................................................     1
Gi2_11375064_x1.....ccaacagaNccggguucgauucccggcgccggagc.........................................................................     1
Gi2_11271302_x1................Ugguucgauucccggcgcc..............................................................................     1
Gi3_28572412_x1..................guucgauuGccggcgccgga...........................................................................     1
Gi2_11127044_x1....................ucgauucccggcgccggagcA........................................................................     1
G1a_6047477_x1.................gCuucgauucccggcgccggag..........................................................................     1
Gi3_28789606_x1.................gguucgauucccggcUcc..............................................................................     1
Gi1_4284495_x1....................ucgauucccggcgccggagU.........................................................................     1
Gi3_28767525_x1....................ucgCuucccggcgccggagc.........................................................................     1
Gi1_4358312_x1.ucacccCacagauccgggu.............................................................................................     1
Gi1_4237244_x1...................uucgauucccggcgccAg............................................................................     1
Gi3_28573061_x1.............uccggguucgauucccggcAccgga...........................................................................     1
Gi3_28888618_x1....................ucgauucccggcAccggagc.........................................................................     1
```

```
Gi2_11055804_x1.......aacagauccggguucgauCcccggcgccg............................................................................     1
Gi1_4342867_x1.....................cgauucccggcgccggagcU.......................................................................     1
Gi4_584974_x1 .................Cuucgauucccggcgccgg...........................................................................     1
Gi3_28709223_x1....................Acgauucccggcgccggag.........................................................................     1
Gi3_28822165_x1..................guuGgauucccggcgccgg...........................................................................     1
Gi4_585899_x1 ................Aguucgauucccggcgccggagc........................................................................     1
Gi3_28876647_x1..........aAauccggguucgauucccggcgccggag.........................................................................     1
Gi1_4257203_x1................Ugguucgauucccggcgccgga..........................................................................     1
Gi4_572180_x1 ................gguucgauucccggcgGcggagc........................................................................     1
Gi1_4373113_x1....cccaacUgauccggguuc..........................................................................................     1
Gi3_28329919_x1..cUcccaacagauccggguuc..........................................................................................     1
Gi5_8997540_x1.ucUcccaacagauccggguu...........................................................................................     1
Gi5_9062050_x1.................gguucgauucUcggcgccggagcgu......................................................................     1
Gi3_28582943_x1................ggguucgauucccggAgccgga..........................................................................     1
Gi5_8956571_x1................ggguucgauuccUggcgccggagc........................................................................     1
G1a_6322399_x1.................gguucgGuucccggcgccg............................................................................     1
G1a_6169776_x1.................gguucgaCucccggcgccgga..........................................................................     1
Gi1_4330992_x1..................guucgaGucccggcgccg............................................................................     1
Gi3_28493974_x1..cacccaacagGuccggguuc..........................................................................................     1
Gi5_9122889_x1.................gguGcgauucccggcgccggagc........................................................................     1
Gi4_606205_x1 .................guucgauucccggcgcAgg...........................................................................     1
G1a_6035669_x1.................gguucgauucccUgcgccgga..........................................................................     1
Gi1_4179955_x1.................gguucgauucccggcgcUg............................................................................     1
Gi3_28590145_x1..................guucgauuGccggcgccggag.........................................................................     1
Gi5_8992512_x1.................gguucgauuccGggcgccgga..........................................................................     1
Gi3_28489770_x1...................uucgauuccAggcgccgga..........................................................................     1
Gi3_28587312_x1......................gauucccggcgccggUgc........................................................................     1
Gi3_28971467_x1....................ucgauucccggcgccCga..........................................................................     1
Gi5_9077938_x1......................gauucccggcgccggagcCu......................................................................     1
Gi1_4375213_x1..................guucgauucccggcgccggagcg.......................................................................     0
Gi2_11072067_x1................ggguucgauucccggcgccg............................................................................     0
Gi1_4215423_x1.ucacccaacagauccgCgu............................................................................................     1
Gi2_11119670_x1...................uCcgauucccggcgccggagc........................................................................     1
Gi3_28276348_x1....................ucgaCucccggcgccggag.........................................................................     1
Gi3_28963298_x1.....................cgauucccggcgUcggagc........................................................................     1
Gi3_28280924_x1..................guucgauucccggcCccggagc........................................................................     1
Gi2_11394025_x1...................uucgauucccggcgccgA...........................................................................     1
Gi3_28428452_x1....................ucgaGucccggcgccggagc........................................................................     1
Gi5_9079622_x1....cccaacagauccgggCuc..........................................................................................     1
Gi1_4348091_x1.................gguucgauucccggcgccCga..........................................................................     1
Gi3_28954306_x1....................ucgauucUcggcgccggag.........................................................................     1
Gi3_28833541_x1.....................cgauGcccggcgccggagc........................................................................     1
Gi4_608663_x1 ................gguucgauucccggcgccgA...........................................................................     1
Gi3_28746704_x1..........aCauccggguucgauucccggcgccgg...........................................................................     1
Gi3_28721884_x1..cacccaacagauccAgguuc..........................................................................................     1
G1a_6296102_x1.................gguAcgauucccggcgccggagc........................................................................     1
Gi3_28419247_x1.ucacccaacagauccggguuU..........................................................................................     1
```

```
Gi3_28438278_x1......................gauucccggcgccggagcU...................................................................     1
Gi1_4171949_x1...................uucgauucccgAcgccgg.......................................................................     1
G1a_6183397_x1..................guucgauucccggcgccggagcU...................................................................     1
Gi5_8848946_x1....................ucgauucUcggcgccggag.....................................................................     1
Gi5_8932518_x1................ggguucgauuUccggcgccggagc....................................................................     1
Gi5_9053971_x1........acagauccggguucgauucGcggcgc..........................................................................     1
Gi4_610398_x1 cucacccaacagauccggguCcg.....................................................................................     1
Gi5_9051740_x1...................uucgauucccggUgccgg.......................................................................     1
Gi5_8875925_x1...................Cucgauucccggcgccggag.....................................................................     1
Gi5_9038163_x1.................gguucCauucccggcgccggagc....................................................................     1
Gi3_28361222_x1..........Ugauccggguucgauucccggcgccggag.....................................................................     1
Gi3_28781900_x1........acagauccggguucgauucccggcgccggG......................................................................     1
Gi3_28331776_x1....................ucgauucccggcgGcggag.....................................................................     1
Gi1_4169482_x1..................guucgauucccUgcgccg........................................................................     1
G1a_6055536_x1.................gguucgauucccgAcgcc.........................................................................     1
Gi5_9075210_x1.ucacccaacagauccggguC.......................................................................................     1
Gi2_11309983_x1.....................Ugauucccggcgccggagc....................................................................     1
Gi3_28770490_x1..................guucgauucccggcgcGggag.....................................................................     1
Gi3_28940332_x1..........Ugauccggguucgauucc................................................................................     1
Gi3_28332991_x1............aucUggguucgauucccggcgccggagc....................................................................     1
G1a_6328472_x1...................uucgauucNcggcgccggagc....................................................................     1
Gi1_4206857_x1...................uucgauucccggGgccgg.......................................................................     1
G1a_6201526_x1.................gAuucgauucccggcgccggag.....................................................................     1
Gi1_4172732_x1........Ucagauccggguucgauuccc...............................................................................     1
Gi5_9107136_x1.............ucAggguucgauucccggcgccg........................................................................     1
G1a_6346247_x1.................gguucgauucccgAcgccgg.......................................................................     1
Gi1_4380338_x1..................guucgaGucccggcgccggagc....................................................................     1
Gi1_4202460_x1.............uccAgguucgauucccggcgccgga......................................................................     1
Gi1_4159212_x1..........Ggauccggguucgauuccc...............................................................................     1
Gi2_11330651_x1.............uccggguucgauucccggcgUcgga......................................................................     1
Gi1_4388966_x1....................ucgauucccggcgUcggagc....................................................................     1
G1a_6100783_x1.................gguucgauucccgAcgccg........................................................................     1
G1a_6004502_x1................ggguucgauucccggUgc..........................................................................     1
Gi3_28962457_x1....Gccaacagauccggguucgauuccc...............................................................................     1
Gi1_4380831_x1..................guucCauucccggcgccgga......................................................................     1
Gi5_8892467_x1....cUcaacagauccggguuc......................................................................................     1
Gi3_28426008_x1....................ucgauuAccggcgccggagc....................................................................     1
Gi1_4170449_x1....................ucgaCucccggcgccggagc....................................................................     1
Gi2_11284605_x1.........cagauccggCuucgauucccggcgccggag.....................................................................     1
G1a_6115971_x1..................Auucgauucccggcgccgga......................................................................     1
Gi3_28599906_x1............auccggguNcgauucccggcg...........................................................................     1
Gi1_4349058_x1..................guucgGuucccggcgccggag.....................................................................     1
Gi5_9084857_x1.............uGcggguucgauucccggc............................................................................     1
Gi5_9035518_x1................ggguucgauucccggcgcc.........................................................................     0
Gi1_4241720_x1.................gguucgauucUcggcgccggagc....................................................................     1
Gi2_11379186_x1.....................cgauucccggcgccggagcguuuA...............................................................     1
Gi5_9116559_x1..................guucgauucccUgcgccggagc....................................................................     1
```

G1a_6256295_x1..................guucgauucUcggcgccgga.. 1
Gi3_28552136_x1....................ucgauucccggcgccggU.. 1
Gi5_8834311_x1..................guucgauucccggcgccggagU.. 1
Gi1_4237845_x1cucacccaacagauccAgguucgauucc.. 1
Gi1_4280231_x1..................guucgauGcccggcgccg.. 1
Gi5_9027678_x1...acccaacagauccggAuucgauucc.. 1
Gi3_28375014_x1..................guucgauucccggcgccggagcg... 0
Gi5_9025560_x1.................gguucgauucccggcgccggaC... 1
Gi3_28728018_x1................ggguucgauucAcggcgccggagc.. 1
Gi2_11180345_x1................Ugguucgauucccggcgccggagc.. 1
Gi5_9067346_x1..................guucgauucccggcgGcgga.. 1
Gi3_28367049_x1......caacagauccgAguucga.. 1
Gi1_4326623_x1..................guucgauAcccggcgccg.. 1
Gi2_11136551_x1..................guucgaAucccggcgccggag... 1
Gi4_571954_x1 ....guAcgauucccggcgccgg... 1
G1a_6020043_x1..................guucgauCcccggcgccggagc.. 1
Gi4_603804_x1 cucacccaacagauccgg.. 0
Gi2_11097809_x1...................uucgauucAcggcgccgg... 1
Gi5_9015306_x1..................guucgauGcccggcgccgg... 1
Gi1_4266472_x1....................ucgaGucccggcgccggagc.. 1
Gi1_4259666_x1.................gguucgauucccggcUccgg... 1
Gi3_28864523_x1....................ucgauucccggAgccgga.. 1
Gi5_8872085_x1.................gguucgauucccgUcgccggag... 1
Gi5_9130071_x1..........agauccggguCcgauuccc... 1
Gi5_9005679_x1.................gguucgauucccggcgccggagcgAu... 1
Gi3_28941386_x1cucacccaacagauccgggCucgauu.. 1
Gi5_8916686_x1...................uucgauucccggcgccAg... 1
Gi1_4234736_x1.................gguucgauucccggcgccgCagc.. 1
Gi3_28565667_x1.......................auucccggcgccggagcCu.. 1
Gi5_8884803_x1..................guucgauucccggcgccggagcAuuucc.. 1
Gi2_11398309_x1cucacccaacagaCccgg.. 1
Gi3_28519129_x1.....................cgauAcccggcgccggagc.. 1
Gi4_577047_x1 ....gguucgauNcccggcgcc... 1
Gi3_28343661_x1...................uucgaCucccggcgccggag... 1
Gi2_10999928_x1.................gguucgauucGggcgccgga... 1
Gi3_28682260_x1.................gguucgauucGcggcgccg.. 1
Gi2_11031964_x1.................gguucgauucccgCcgccgg... 1
Gi3_28608323_x1...........gGuccggguucgauucccggcgccgga.. 1
Gi2_11146961_x1.................gguucgauucccggcgccggagcgC.. 1
Gi1_4347531_x1...................uucgauucccggcgccggagcU... 1
Gi5_8935087_x1.................gguAcgauucccggcgccgg... 1
Gi2_11061560_x1.................gguucgauucccggcgccggagcAu.. 1
Gi3_28269161_x1.....ccaacagauccggguucgCuuccc... 1
Gi2_10994031_x1.................gguucgauucccggcgcAgg... 1
Gi5_8902831_x1.........cagauccggguucgauCcccggcgccg.. 1
Gi1_4340501_x1.................gguNcgauucccggcgccg.. 1
Gi5_9011318_x1.................gguucgauucccggcgccgUagc.. 1

```
Gi3_28774530_x1.............uccgAguucgauucccggcgccgga..........................................................................     1
Gi3_28860126_x1....cccaacagauccggguucgauuGcc...................................................................................     1
Gi5_8877369_x1..................guucgauucccggcgccgU...........................................................................     1
Gi5_8956102_x1.....................cgauucccggcgccggagcA.......................................................................     1
Gi2_11193724_x1......................gauuccAggcgccggagc........................................................................     1
Gi3_28322090_x1................ggguucgauuccGggcgccgga..........................................................................     1
Gi2_11018820_x1...................uAcgauucccggcgccggagc........................................................................     1
Gi4_610798_x1 .................gguucgauucccggcgccgC...........................................................................     1
Gi3_28584641_x1................ggguucgauucccggcgcc.............................................................................     0
Gi5_8840835_x1.............uccggguucgauucUcggc................................................................................     1
Gi5_9056090_x1.......aacagauccgggCucgauucccggcgccggagc........................................................................     1
Gi3_28405878_x1.........cagauccggguucgauucccggcgccg............................................................................     0
Gi5_9049110_x1.ucacccaacagauccAgguu...........................................................................................     1
Gi3_28349661_x1.ucacccaacagauccggAuuc..........................................................................................     1
G1a_6177820_x1...................uucgauucccggcgccgAagc........................................................................     1
Gi2_11266359_x1.....................cgauucccggcgccggagcCu......................................................................     1
Gi1_4282852_x1.................ggAucgauucccggcgccggag.........................................................................     1
Gi3_28920230_x1cGcacccaacagauccggguucgauucc....................................................................................     1
Gi5_8935297_x1..................guucgauucccCgcgccgga..........................................................................     1
Gi3_28893665_x1...............cggguucgaCucccggcgccgga..........................................................................     1
Gi2_11024313_x1.................gguucgauucccggcgccggagcguuA....................................................................     1
Gi1_4212124_x1.......aacagauccggguucgCuucccggcgccgg...........................................................................     1
Gi2_11088551_x1.....................cgauucccggcgccggagcU.......................................................................     1
Gi3_28755984_x1cucacccaacagaucAgg..............................................................................................     1
Gi2_11088895_x1..................Nuucgauucccggcgccggagc........................................................................     1
Gi3_28845316_x1...acccaGcagauccggguucgauuccc...................................................................................     1
Gi3_28281122_x1...................uucgauucccggcgcUgg...........................................................................     1
Gi2_11357110_x1...................uucgGuucccggcgccgg...........................................................................     1
Gi2_11021314_x1....................ucgauucccAgcgccggagc........................................................................     1
Gi3_28630962_x1..................Nuucgauucccggcgccgg...........................................................................     1
Gi1_4306787_x1cucacccaGcagauccggguucga........................................................................................     1
G1a_6325166_x1.................ggAucgauucccggcgccggag.........................................................................     1
Gi3_28606103_x1.....Gcaacagauccggguucgauucccggcgccggagc........................................................................     1
Gi5_8867078_x1....cccGacagauccggguuc..........................................................................................     1
Gi1_4325094_x1..................guAcgauucccggcgccg............................................................................     1
Gi2_11100270_x1.........cagaGccggguucgauucccggcgccggag.........................................................................     1
G1a_6021407_x1.................gguucgauucccggcgccggagU........................................................................     1
Gi2_11020624_x1..................guucgauuccAggcgccgga..........................................................................     1
Gi3_28815093_x1....................ucgauGcccggcgccggag.........................................................................     1
Gi1_4361395_x1...................uucgauucccggcUccgg...........................................................................     1
Gi1_4241313_x1.........cCgauccggguucgauucccggcgcc.............................................................................     1
Gi1_4380293_x1cucacccaacGgauccggguucgauucc....................................................................................     1
Gi5_9087436_x1.......................auucccggcgccggagcgA......................................................................     1
Gi1_4213027_x1.................gguucgauucccggcgccggaCc........................................................................     1
Gi5_9127316_x1....cccaacagauccggguucgau.......................................................................................     0
G1a_6278848_x1.................gguucgauucccggcCccggagc........................................................................     1
Gi5_8857528_x1.................gguucgGuucccggcgccggagcguu.....................................................................     1
```

```
Gi3_28478583_x1.....ccGacagauccggguucgauucccggcgccgg..........................................................................     1
Gi5_9048280_x1..................guucgGuucccggcgccggag........................................................................     1
Gi3_28900399_x1............auccggguucgaGucccggcgccgga.........................................................................     1
Gi5_8849782_x1.................gguucgauucccggcgccggGgcguuu...................................................................     1
G1a_6020147_x1.................gguucgauuccAggcgccggag........................................................................     1
Gi2_11395991_x1....................ucgauucccggUgccggag........................................................................     1
Gi4_592464_x1 ..................guucgauucccggcgccgA..........................................................................     1
Gi2_11014138_x1..................guucgauucccgUcgccggag........................................................................     1
G1a_6295680_x1...................uucgauucccggcgccggagcCu.....................................................................     1
Gi3_28403535_x1..........aUauccggguucgauucccggcgccgg..........................................................................     1
G1a_6002738_x1.................gCuucgauucccggcgccgg..........................................................................     1
Gi5_9106722_x1.....................cgauucccggcgcUggagcguuuc..................................................................     1
Gi4_570145_x1 ..................gGucgauucccggcgccgga.........................................................................     1
Gi2_11279226_x1.....................cgauucccAgcgccggagcguuu...................................................................     1
Gi4_587679_x1 ......................gauucccggcgccggagcC......................................................................     1
Gi5_9077616_x1...................uucgauucccggcgccggagcguuucc.................................................................     0
Gi1_4261491_x1.................gguucgauuccUggcgccggag........................................................................     1
Gi1_4353672_x1..........agauAcggguucgauuccc..................................................................................     1
Gi2_10997619_x1....................ucgauucccggcgccggagcU......................................................................     1
Gi5_9125235_x1........................................guuucccgccCuccuucgucu..................................................     1
Gi1_4240444_x1.................gguucgauucccggcgAcggag........................................................................     1
G1a_5996456_x1................ggguucgauucccggcgccggagcC......................................................................     1
Gi5_8944125_x1................ggguucgauucccggcgA.............................................................................     1
Gi2_11278841_x1.................gguGcgauucccggcgccggag........................................................................     1
Gi3_28973498_x1....................ucgauuccGggcgccggagc.......................................................................     1
Gi5_8862066_x1....................uGgauucccggcgccgga.........................................................................     1
Gi5_8935263_x1....cccaacagauUcggguuc.........................................................................................     1
Gi1_4296745_x1.................gguucgauucccggcgccggaC........................................................................     1
Gi3_28968747_x1...................uucgauucccggcAccgga.........................................................................     1
Gi4_570127_x1 ..................guCcgauucccggcgccg...........................................................................     1
Gi3_28579118_x1....................ucgauucccAgcgccggagc.......................................................................     1
G1a_6159216_x1.................gguucgauucccAgcgccgga.........................................................................     1
Gi1_4327807_x1.ucaNccaacagauccgggu...........................................................................................     1
Gi2_11138380_x1....................ucgauucccggcgcGggagc.......................................................................     1
G1a_6071562_x1.................Aguucgauucccggcgccg...........................................................................     1
G1a_6235090_x1.................gguucgauucccggcAccgg..........................................................................     1
Gi5_8950064_x1.................gguuGgauucccggcgcc............................................................................     1
Gi1_4240262_x1...................uucgauuccGggcgccgg..........................................................................     1
Gi5_9125988_x1..................guCcgauucccggcgccggag........................................................................     1
Gi3_28392848_x1.................gguucgauucccggcgccggagcUu.....................................................................     1
Gi1_4301381_x1..................guucgauucccggcgccggaCc.......................................................................     1
Gi1_4359562_x1......caCcagauccggguucgauucccggc...............................................................................     1
Gi2_11129664_x1................ggguucgauucccggcgAcgga.........................................................................     1
G1a_6114946_x1..................guucgauucccggcgccgA..........................................................................     1
Gi1_4165188_x1cucacccaacaAauccgg.............................................................................................     1
Gi2_11000175_x1.................gguucUauucccggcgccggag........................................................................     1
Gi5_8973379_x1....................ucgauucccggcgAcggagc.......................................................................     1
```

Gi3_28475828_x1.........cagauccggguucgCuuccc...   1
Gi5_8848803_x1..................guGcgauucccggcgccggag...   1
Gi2_11231128_x1..........agauccggguucgauucccggcgccggag...   0
Gi5_8970092_x1...................uucgauucccggcgcUgg...   1
Gi2_11275391_x1....................ucgauucccggcgccggagA..   1
Gi2_11061990_x1....................ucgauuccNggcgccgga..   1
Gi5_9135537_x1.ucacccGacagauccggguu...   1
Gi2_11366978_x1....................ucgauAcccggcgccggagc..   1
G1a_6320710_x1..................guAcgauucccggcgccgg...   1
Gi1_4382342_x1..........agauccggguucgauCccc...   1
Gi5_9062397_x1....................ucgauucccggUgccggag...   1
Gi5_8952778_x1.................gguucgauucccggcgccUgag...   1
Gi2_11319370_x1....................ucgGuucccggcgccggag...   1
Gi3_28795804_x1cucGcccaacagauccgg..   1
Gi5_9026658_x1........aGagauccggguucgauucccggcgc..   1
G1a_6176978_x1.................gguucgauCcccggcgccgga..   1
G1a_6274430_x1.................gguucgCuucccggcgccggagc..   1
Gi5_9067315_x1....cccaacagauccgAguuc..   1
Gi1_4327164_x1............auccggguucgauucccggcgccggU..   1
Gi5_9051218_x1.ucacccaacaAauccggguu...   1
Gi2_11037821_x1...................uucAauucccggcgccggag...   1
Gi2_11045741_x1..................guucgauucccggcgccgAa..   1
Gi1_4365740_x1....................ucgUuucccggcgccggagc..   1
Gi4_612542_x1 ....gauucccggcgccggagc..   0
Gi5_8837373_x1..................Nuucgauucccggcgccg..   1
Gi3_28872991_x1..................guucgauucccggcUccgga..   1
Gi5_9056183_x1...acccaacagauccggguucgauCcc..   1
Gi2_11133986_x1...............cggguucgauucccAgcgccgga..   1
Gi3_28428151_x1..................guucgauucccgCcgccgga..   1
Gi5_8977529_x1.................gguucgauuUccggcgcc...   1
Gi3_28524515_x1.............uccggguucUauucccggcgccgga..   1
Gi1_4162336_x1.............uccggguucgauucccggcgccgg...   0
Gi2_11246227_x1.................gguNcgauucccggcgccg..   1
Gi3_28339293_x1.ucacccUacagauccggguuc..   1
Gi1_4371333_x1...................uucgauuccUggcgccggagc..   1
Gi4_598390_x1 ....gguucgauucccggGgccgg...   1
Gi5_9112221_x1.................gguucgauucccggcgccggCgcguu...   1
Gi2_11066010_x1...................uucgauucccggcgAcggagc..   1
Gi1_4191855_x1Gucacccaacagauccggguucgauucc..   1
Gi3_28680340_x1..................Nuucgauucccggcgccg..   1
Gi5_9129461_x1.................gguucgauucccgCcgccgg...   1
Gi5_8843127_x1........Ucagauccggguucgauucccggcgc..   1
Gi3_28652401_x1....................ucgauucccggcgccggagA..   1
Gi2_10996523_x1.................gguucgauucccCgcgccg..   1
Gi1_4271575_x1.................gAuucgauucccggcgccggag...   1
Gi5_8892905_x1..........agauccggguucgaCuccc...   1
Gi5_9070968_x1....................ucgaCucccggcgccggagc..   1

```
Gi5_8895369_x1......................gauucccggcgcAggagc........................................................................	1
Gi3_28467228_x1.................gguucgauucccgCcgccggag.........................................................................	1
Gi2_11273501_x1.................gguucgauucccgCcgccggag.........................................................................	1
Gi4_574948_x1 .................gguucgauCcccggcgccggagc........................................................................	1
Gi1_4389669_x1.................gguucUauucccggcgccgg...........................................................................	1
Gi3_28778243_x1....cccaacagaucUggguucgauuccc...................................................................................	1
Gi3_28336947_x1..................guucgauucccggcgccgAag.........................................................................	1
Gi1_4272720_x1.................gCuucgauucccggcgccg............................................................................	1
Gi2_11248818_x1.......aacUgauccggguucgauucccggcgccg............................................................................	1
Gi3_28381888_x1....................ucgauucccUgcgccggagc........................................................................	1
Gi1_4234834_x1............auccggguucgauucccggcgcUgga..........................................................................	1
Gi3_28712821_x1..................guucgauucccCgcgccg............................................................................	1
Gi2_11189622_x1...................uucgauucccggcgUcgg...........................................................................	1
Gi2_11268373_x1....................ucgauucccggcgccggaAc........................................................................	1
Gi2_11180185_x1....Gccaacagauccggguuc..........................................................................................	1
Gi5_9122369_x1.ucacccaacagauccgCguu...........................................................................................	1
Gi1_4271800_x1cucacccaacUgauccgg..............................................................................................	1
Gi4_604592_x1 .................gguucgauuUccggcgccgg...........................................................................	1
Gi3_28676654_x1....cGcaacagauccggguucgauuccc...................................................................................	1
Gi5_8885001_x1..................Uuucgauucccggcgccggagcguu.....................................................................	1
Gi3_28294130_x1..................guucgauucccggcgccgCag.........................................................................	1
Gi5_8913613_x1..cacccaGcagauccggguucga........................................................................................	1
Gi5_8866384_x1....................ucgauucccggcgccggC..........................................................................	1
Gi3_28447617_x1...................uucgauucccgAcgccgga..........................................................................	1
Gi3_28686517_x1cucacccaacagauccUg..............................................................................................	1
Gi1_4214623_x1.....................cgauucccggcgccggagcguuuc...................................................................	0
Gi2_11154278_x1.................gguucgauuAccggcgccg............................................................................	1
Gi5_8902008_x1.ucacUcaacagauccggguu...........................................................................................	1
Gi3_28821354_x1...............cggguucgauucccggcgccggagcA.......................................................................	1
Gi1_4301285_x1.................gguucgauuUccggcgccggagc........................................................................	1
Gi2_11077824_x1.......aUcagauccggguucgauucccggcgccg............................................................................	1
Gi2_11396827_x1..................guucgauucccCgcgccggag.........................................................................	1
Gi5_8997089_x1..............ccggguucgGuucccggcgccgga..........................................................................	1
Gi5_8873427_x1cucacccaacagauccUg..............................................................................................	1
Gi3_28459116_x1...................uucNauucccggcgccgga..........................................................................	1
Gi5_8925876_x1.................gguucgauucccggcgcGggagc........................................................................	1
Gi3_28446139_x1...............Uggguucgauucccggcgccggagc........................................................................	1
Gi4_579019_x1 ..................gGucgauucccggcgccggagc........................................................................	1
G1a_6250668_x1..................guucgauucccggUgccgg...........................................................................	1
Gi3_28881241_x1......................gauucccggcgccggagG........................................................................	1
Gi1_4175616_x1.................gguCcgauucccggcgccgga..........................................................................	1
Gi1_4159385_x1............aGccggguucgauucccggcgccgga..........................................................................	1
Gi3_28481643_x1.............Cccggguucgauucccggcgccgga..........................................................................	1
Gi5_9045279_x1.................gAuucgauucccggcgccggagcguuu....................................................................	1
Gi3_28538480_x1.................gguucgauucccggcgGc.............................................................................	1
Gi3_28644290_x1................gggGucgauucccggcgccgga..........................................................................	1
Gi2_11308489_x1.....................cgUuucccggcgccggagcguuuc...................................................................	1
```

```
Gi2_11039943_x1..................guucgauucccUgcgccg............................................................................     1
Gi2_11154360_x1.....................Ggauucccggcgccggagcguuuc...................................................................     1
G1a_5986143_x1.................gCuucgauucccggcgcc.............................................................................     1
G1a_6321473_x1...................uucgauucccggAgccggagc........................................................................     1
Gi1_4240077_x1.........cagauccgNguucgauucccggcgcc.............................................................................     1
Gi2_11130540_x1....................ucgauucccgAcgccgga..........................................................................     1
Gi3_28586589_x1.............uccggguucgauucccggcgccggagc........................................................................     0
Gi5_9103529_x1..................guucgauucGcggcgccgg...........................................................................     1
Gi1_4241493_x1............auccggguCcgauucccggcgccgg...........................................................................     1
G1a_6104029_x1.................gguucgauucccCgcgccggagc........................................................................     1
Gi5_9017302_x1..............Ucggguucgauucccggcgccgga..........................................................................     1
Gi1_4175261_x1.................gguucgauucccggcgccgAag.........................................................................     1
Gi2_11241331_x1.......................auucccggcgccggagcC.......................................................................     1
Gi5_9101242_x1.............uccggguucgauucccUggc...............................................................................     1
Gi3_28319896_x1Gucacccaacagauccggguucgauuccc...................................................................................     1
G1a_6186067_x1.................gguuAgauucccggcgccggagc........................................................................     1
Gi3_28448279_x1..................guucCauucccggcgccggag.........................................................................     1
Gi3_28683435_x1........acagauccggguucgauCcccggcgccggagc........................................................................     1
Gi1_4368857_x1.................ggNucgauucccggcgccgg...........................................................................     1
Gi2_11056791_x1..........agauccggAuucgauucc....................................................................................     1
Gi4_609748_x1 .................gguucgauucccggcgccggagcCu......................................................................     1
Gi2_11294708_x1cCcacccaacagauccgg..............................................................................................     1
Gi3_28439530_x1.............uccggguucgauCcccggcgccgga..........................................................................     1
Gi2_11383158_x1..................guucgauucccggcgccgUa..........................................................................     1
Gi2_11016792_x1.........cagauccggguucgauuccc...................................................................................     0
Gi3_28970858_x1....................ucgauucccggcgcUgga..........................................................................     1
Gi2_11295088_x1.................gguNcgauucccggcgccgga..........................................................................     1
Gi1_4295070_x1......caacagauccggguucgauucccUggc...............................................................................     1
Gi3_28361274_x1..........agauccggguucgauucUc...................................................................................     1
Gi2_11365551_x1......................gauuccUggcgccggagc........................................................................     1
Gi5_9011185_x1....................ucgauCcccggcgccggagc........................................................................     1
Gi3_28723782_x1...............Aggguucgauucccggcgccgga..........................................................................     1
Gi5_8884316_x1...............cggguucgauucccggUgc..............................................................................     1
Gi3_28914915_x1...............cggguucgauucccggcAccgga..........................................................................     1
Gi2_11289243_x1.................gguucgauucccgCcgcc.............................................................................     1
G1a_6154963_x1...................uuUgauucccggcgccggagc........................................................................     1
Gi3_28607845_x1...............cggNuucgauucccggcgccgga..........................................................................     1
Gi1_4222424_x1..................guucgauuccAggcgccggagc........................................................................     1
Gi2_11082774_x1..................guuGgauucccggcgccggag.........................................................................     1
Gi4_567782_x1 Gucacccaacagauccggguucg.........................................................................................     1
Gi5_8942041_x1........acagGuccggguucgauucccggcgc..............................................................................     1
Gi5_9097109_x1........aUagauccggguucgauucccggcgc..............................................................................     1
Gi3_28883816_x1.................gguucgauucccggNgccggagc........................................................................     1
Gi2_11151932_x1.............uccggguucgauuccUggcgccgga..........................................................................     1
Gi2_11321425_x1..........agauccggguucUauucc....................................................................................     1
Gi3_28350522_x1..........agauccggguucUauucccggcgccgg...........................................................................     1
Gi4_572457_x1 .................gguucgCuucccggcgccgg...........................................................................     1
```

Gi3_28353381_x1.............uccggguucgauucccggcUccgga.. 1
Gi3_28917137_x1....................ucgauucGcggcgccggagc.. 1
Gi1_4332510_x1.................gguucgaGucccggcgccgga.. 1
Gi2_11222279_x1......................gauuUccggcgccggagc.. 1
Gi2_11107148_x1.......aacagauccggguuAgauucccggcgccg.. 1
Gi3_28480317_x1...................uucgauucccgAcgccgg... 1
Gi5_9001980_x1..................guucgauGcccggcgccg.. 1
Gi2_11282668_x1.............uccgggAucgauucccggcgccgga.. 1
Gi4_593486_x1 ....Cguucgauucccggcgccgg... 1
Gi5_8901041_x1.................gguucgauucccggcgccgCag... 1
Gi3_28425353_x1......caacagauccgggCucgauuccc... 1
Gi3_28399980_x1..................guucgauucccCgcgccggag... 1
Gi3_28897627_x1.........cagaucUggguucgauuccc... 1
Gi3_28348367_x1.................gguucgaAucccggcgcc... 1
Gi2_11013948_x1..................guucgauuGccggcgccgga.. 1
Gi2_11405600_x1...............cggguucgauucccggcgccggU.. 1
Gi5_8873261_x1cucaUccaacagauccgg.. 1
Gi5_9120349_x1....................ucgauuAccggcgccggagc.. 1
Gi2_11333413_x1..................guucgaAucccggcgccgga.. 1
Gi5_8890193_x1..................guucgauucccggcgccggGg... 1
Gi1_4217158_x1cucacccaacagauccggguucAa.. 1
Gi3_28943427_x1......caacagaucUggguucgauuccc... 1
Gi2_11331612_x1...................uucAauucccggcgccgg... 1
Gi2_11314643_x1...................uucgauucccggcgccggUg... 1
Gi3_28806175_x1................ggguucgGuucccggcgccgga.. 1
Gi2_11038569_x1.................gguucgauucccggcgccgUa.. 1
Gi3_28585848_x1............Uuccggguucgauucccggcgccgga.. 1
Gi3_28813952_x1....................ucgGuucccggcgccggag... 1
Gi3_28489861_x1..........agauccggguuAgauucccggcgccggag... 1
Gi3_28332252_x1..........aCauccggguucgauucccggcgccggag... 1
Gi3_28446037_x1....cccaacagGuccggguucgauuccc... 1
Gi3_28369938_x1...........gaucUggguucgauuccc... 1
Gi3_28547069_x1......................gauucccUgcgccggagc.. 1
Gi3_28435967_x1......caacagauccgggGucga.. 1
Gi3_28271680_x1.................gguucgauucccggGgcc... 1
Gi3_28987072_x1.......aaUagauccggguucgauucc.. 1
Gi5_8965334_x1.................gguucgauuUccggcgccggagcguu... 1
Gi3_28897993_x1...........gaGccggguucgauuccc... 1
Gi5_9076870_x1...................uucgauucccggcgccgC... 1
Gi5_8857640_x1.................gguucgUuucccggcgccggag... 1
Gi3_28502031_x1....................ucgauucccggcgccggGg... 1
Gi5_8937867_x1..................guucCauucccggcgccggag... 1
Gi3_28584321_x1..........agauccggguucgauucccggcgccggGg... 1
Gi1_4364029_x1cucacccaacagauccggguucgauuUc.. 1
Gi2_11168535_x1...................uucgauucccggcgccUga.. 1
Gi1_4278672_x1......caGcagauccggguucgauucccggc.. 1
Gi1_4324906_x1.........cagauccggguucgCuucccggcgcc... 1

```
G1a_6152621_x1.................Cguucgauucccggcgccgg...........................................................................   1
Gi1_4221503_x1..........agauccggguucgauucccggcgccggagc........................................................................   0
Gi2_11261593_x1.........cagauccggguucgauucccggcgccggagc........................................................................   0
Gi3_28328436_x1..cGcccaacagauccggguuc..........................................................................................   1
Gi3_28449308_x1...................uucgaCucccggcgccgga..........................................................................   1
G1a_6005304_x1...................uucgauuccUggcgccggagc........................................................................   1
Gi3_28712356_x1..................guucgauuccGggcgccggag.........................................................................   1
Gi2_11319089_x1.................gguucgauucccCgcgccggag.........................................................................   1
Gi1_4313092_x1.................gguucgauucccAgcgccggag.........................................................................   1
Gi3_28890204_x1.............ucAggguucgauucccggcgccgga..........................................................................   1
Gi2_11288815_x1.................gguAcgauucccggcgcc.............................................................................   1
Gi3_28572390_x1.................gguucgauAcccggcgccgg...........................................................................   1
Gi2_11397560_x1..................guucgauucccggcgGcgga..........................................................................   1
Gi1_4201107_x1..................guucgauucUcggcgccggagc........................................................................   1
Gi1_4186633_x1.uGacccaacagauccgggu............................................................................................   1
Gi1_4159395_x1..................guuNgauucccggcgccggagc........................................................................   1
Gi3_28501653_x1......cUacagauccggguucga........................................................................................   1
Gi3_28759870_x1..........agauccggguucgauuccU...................................................................................   1
Gi1_4225088_x1.................gguucgauucccggAgccg............................................................................   1
Gi3_28362273_x1cucaGccaacagauccgg..............................................................................................   1
G1a_6355749_x1..................guucgauAcccggcgccggagc........................................................................   1
Gi2_11199524_x1...............Gggguucgauucccggcgccgga..........................................................................   1
Gi1_4323896_x1............auccggAuucgauucccggcgccgg...........................................................................   1
Gi2_11133795_x1......................gauucAcggcgccggagc........................................................................   1
Gi3_28662191_x1........acagauccggguucgauucccgUcgccgg...........................................................................   1
Gi5_9103469_x1..........aCauccggguucgauuccc...................................................................................   1
Gi3_28439206_x1.........cagauccggguucgauuGcc...................................................................................   1
Gi5_8933169_x1..cacccGacagauccggguucga........................................................................................   1
G1a_6220951_x1...................uAcgauucccggcgccggagc........................................................................   1
Gi1_4364403_x1.ucUcccaacagauccgggu............................................................................................   1
Gi4_574850_x1 ..................guucgGuucccggcgccgga..........................................................................   1
Gi1_4336294_x1.................gguuAgauucccggcgccggag.........................................................................   1
Gi3_28426142_x1.............uccggguucgCuucccggcgccgga..........................................................................   1
Gi2_11358108_x1..........agauccggguucgGuucc....................................................................................   1
Gi2_11210225_x1....................ucAauucccggcgccggagc........................................................................   1
Gi3_28868060_x1.........cagaGccggguucgauuccc...................................................................................   1
G1a_6201313_x1...................uucgauucccggcgccggCgc........................................................................   1
Gi3_28284791_x1...........gauccgAguucgauuccc...................................................................................   1
Gi3_28659909_x1...................uucgauucNcggcgccgg...........................................................................   1
Gi5_9017239_x1..................guucUauucccggcgccggagcguuucc..................................................................   1
Gi3_28519252_x1cucacccaNcagauccggguucgauu......................................................................................   1
Gi3_28529026_x1..................guucgauGcccggcgccgga..........................................................................   1
Gi3_28494870_x1................ggguucgauucccggcgccggGgc........................................................................   1
Gi2_11384950_x1..........agaCccggguucgauucc....................................................................................   1
Gi1_4331175_x1.................gguucgauucccggcgccUga..........................................................................   1
Gi5_8887625_x1......caacagauccgggCucgauucccggcgccg............................................................................   1
Gi5_8877325_x1..................guucgUuucccggcgccggagc........................................................................   1
```

```
Gi5_9128815_x1..................guucgauucccggAgccggagc........................................................................    1
Gi1_4249212_x1.................gguucgaCucccggcgccggag.........................................................................    1
Gi3_28473247_x1.............uUcggguucgauucccggcgccg............................................................................    1
Gi3_28714050_x1.........cagauccggguucgaCucccggcgccgg...........................................................................    1
Gi2_11386665_x1....................ucgauucccggGgccgga..........................................................................    1
Gi5_8925769_x1..................guucgaAucccggcgccggagcguuu....................................................................    1
Gi1_4376981_x1...................uucgGuucccggcgccggagc........................................................................    1
Gi2_11311669_x1......................gauucccggcgcGggagc........................................................................    1
Gi1_4339970_x1..................guucgauucccggcgccAgagc........................................................................    1
Gi1_4158953_x1.................gguucgGuucccggcgccg............................................................................    1
Gi5_8888853_x1..................guucgauucccggcgGcggagc........................................................................    1
Gi5_8940077_x1...................uucgauucccggcgccgga..........................................................................    0
Gi5_9000256_x1.....................cgauucccggcgccggagcguu.....................................................................    0
Gi2_11325663_x1.............uGcggguucgauucccggcgccgga..........................................................................    1
Gi1_4248954_x1.ucacccUacagauccgggu............................................................................................    1
Gi3_28940951_x1.............uccggguucgauuccAggcgccgga..........................................................................    1
Gi2_11368171_x1.................gguucgauAcccggcgccggag.........................................................................    1
Gi2_11302589_x1...............cggguucgauucAcggcgccgga..........................................................................    1
Gi3_28643184_x1...................uucgauucccggcgcAggagc........................................................................    1
Gi5_9104398_x1..cUcccaacagauccggguucga........................................................................................    1
Gi5_9019263_x1.................gguucgauucUcggcgccgga..........................................................................    1
Gi1_4264589_x1....................ucgauucccggUgccggagc........................................................................    1
Gi5_9011958_x1.......aacagauccggguucgauucccggcgccgg...........................................................................    0
Gi1_4191986_x1............aCccggguucgauucccggcgccgg...........................................................................    1
Gi2_11291518_x1cucacccaacagauccgC..............................................................................................    1
Gi3_28714124_x1..cacccaacagauccgAguuc..........................................................................................    1
Gi3_28603845_x1.............uccggguucgauucccggc................................................................................    0
Gi3_28580361_x1....................ucgauucccggcgccggagcA.......................................................................    1
Gi4_598994_x1 .................gguucgauucccggUgccgg...........................................................................    1
Gi2_11000590_x1......................gauuAccggcgccggagc........................................................................    1
Gi5_9124832_x1.................gguucgauucccggGgccggagc........................................................................    1
Gi1_4201748_x1...................uucgauucccggcgccggagcA.......................................................................    1
G1a_6053965_x1.................gguucgGuucccggcgccgg...........................................................................    1
Gi5_9133714_x1....................ucgauucccggUgccggagc........................................................................    1
Gi3_28910686_x1...........Aauccggguucgauucccggcgccggag.........................................................................    1
Gi4_595660_x1 cucacccaacagauccggguucA.........................................................................................    1
Gi5_8970024_x1........acagauccggguucgauucccggAgc..............................................................................    1
Gi5_9051916_x1..................guNcgauucccggcgccggagc........................................................................    1
Gi4_569927_x1 .................gguucgauucccggcgccggagcA.......................................................................    1
Gi3_28792058_x1..................guucgaAucccggcgccg............................................................................    1
Gi2_11237360_x1.................gguucgauuGccggcgccggag.........................................................................    1
G1a_6096632_x1..................Uuucgauucccggcgccggag.........................................................................    1
Gi4_589114_x1 .................gguucgauucccggcgccggaAc........................................................................    1
Gi5_9053569_x1..................guNcgauucccggcgccg............................................................................    1
Gi3_28630680_x1..................guucgauucccggcgccgUagc........................................................................    1
Gi1_4324529_x1.................gguucAauucccggcgccggagc........................................................................    1
Gi3_28846220_x1................ggguucgaNucccggcgccggagc........................................................................    1
```

```
Gi3_28529579_x1...................uucgauuAccggcgccggagc........................................................................     1
Gi1_4225951_x1.ucacAcaacagauccgggu............................................................................................     1
Gi3_28527212_x1.................gguucCauucccggcgcc.............................................................................     1
Gi1_4215116_x1.................gguucgauucccggGgccgga..........................................................................     1
Gi3_28509361_x1..................gCucgauucccggcgccggagcg.......................................................................     1
Gi5_9110155_x1.................gguucgauucUcggcgccggag.........................................................................     1
G1a_6286866_x1.................Cguucgauucccggcgccg............................................................................     1
Gi3_28555333_x1....................ucgauucccggcgccggaC.........................................................................     1
Gi3_28783069_x1....................ucgauucccggcUccggagc........................................................................     1
Gi1_4365183_x1cucacccaacagauccggguucgauuU.....................................................................................     1
Gi3_28795788_x1......................gaAucccggcgccggagc........................................................................     1
Gi2_11372011_x1.....................cgauucccggUgccggagcguuu....................................................................     1
Gi5_8909169_x1......................gauucccggcgccgAagc........................................................................     1
Gi2_11103065_x1....cccaacagauccggguGc..........................................................................................     1
Gi3_28427369_x1.ucacccaacagGuccggguuc..........................................................................................     1
Gi3_28376183_x1.................gguucgUuucccggcgccgg...........................................................................     1
Gi3_28376579_x1..................guucgauuUccggcgccg............................................................................     1
Gi3_28335349_x1........acagauccNgguucgauucccggcgccgga..........................................................................     1
Gi3_28378752_x1...........gauccggguucgauuccA...................................................................................     1
Gi3_28684600_x1...................uucgauuUccggcgccgg...........................................................................     1
Gi1_4202196_x1.....................cgauucccggcgccggag.........................................................................     0
Gi2_11226528_x1..........agGuccggguucgauucc....................................................................................     1
Gi4_585429_x1 .................Aguucgauucccggcgccgg...........................................................................     1
Gi3_28311070_x1....................ucgauucccggcgcUggag.........................................................................     1
G1a_6327152_x1.................gguucgauuccUggcgccgga..........................................................................     1
Gi2_11022829_x1....cccaCcagauccggguuc..........................................................................................     1
Gi5_9097006_x1.................gguucgUuucccggcgccgga..........................................................................     1
Gi3_28420422_x1..........agauccggguucgauucccggcgccggaA.........................................................................     1
Gi5_9129558_x1.................gguucgaGucccggcgccgga..........................................................................     1
Gi3_28419933_x1..........agauccggNuucgauucc....................................................................................     1
Gi3_28381752_x1....................ucgauucccggAgccggag.........................................................................     1
Gi4_592725_x1 ..................guucgauucGcggcgccggagc........................................................................     1
G1a_6024901_x1.................gguucgauuAccggcgccggag.........................................................................     1
G1a_6126895_x1..................guucgauuNccggcgccgg...........................................................................     1
Gi2_11377995_x1..................guucgauuccGggcgccg............................................................................     1
Gi3_28282524_x1.uUacccaacagauccggguuc..........................................................................................     1
Gi2_11361035_x1..................guucgauuGccggcgccg............................................................................     1
Gi1_4277007_x1..................guucgauucccggcUccgg...........................................................................     1
Gi1_4316704_x1.................gguucgauucccggcgccgUag.........................................................................     1
Gi5_9001791_x1.....................cUauucccggcgccggagcguuucc..................................................................     1
Gi2_11135237_x1..................guucgauuccUggcgccg............................................................................     1
Gi2_11121073_x1.................gguucgauucccgAcgcc.............................................................................     1
Gi5_8841715_x1....Accaacagauccggguucgauucc....................................................................................     1
Gi5_8921644_x1..................guucgauucccggcgcUggag.........................................................................     1
G1a_6212261_x1..................guucgauucccggcgAcg............................................................................     1
Gi2_11051947_x1....cccaacaUauccggguuc..........................................................................................     1
Gi5_8920249_x1.................gguucgauucccggcCccggagc........................................................................     1
```

```
Gi1_4311669_x1.................gguucgauuccAggcgccg............................................................................     1
Gi5_9047258_x1.................gguucgauucccgAcgccggagcgu......................................................................     1
Gi1_4264243_x1.............uccggguucgauucccAgcgccgga..........................................................................     1
Gi3_28467549_x1.................gguucgUuucccggcgccg............................................................................     1
Gi3_28782586_x1..................guucgaAucccggcgccggag.........................................................................     1
Gi2_11114347_x1.....................cCauucccggcgccggagcguuu....................................................................     1
Gi3_28901503_x1......caacagauccggguCcga........................................................................................     1
Gi3_28618792_x1.................gguucgauucAcggcgcc.............................................................................     1
Gi5_8940859_x1......................gaGucccggcgccggagc........................................................................     1
G1a_6044083_x1..................guucAauucccggcgccggag.........................................................................     1
Gi3_28269762_x1..cacccaacagauccgggGuc..........................................................................................     1
Gi3_28872324_x1.............ucUggguucgauucccggcgccgga..........................................................................     1
Gi1_4312696_x1........................uucccggcgccggagcgA......................................................................     1
Gi5_8974423_x1..cacccaacagauccgggAucga........................................................................................     1
Gi1_4185539_x1......................gauucccggcgccggagU........................................................................     1
Gi3_28846649_x1.....................cgauucccggcgccggagcgC......................................................................     1
Gi5_8953659_x1..................guucgauucccUgcgccgga..........................................................................     1
Gi5_8834769_x1...aGccaacagauccggguucgauuccc...................................................................................     1
Gi2_11000963_x1..................guucgauuGccggcgccggagc........................................................................     1
Gi3_28495574_x1..........agauGcggguucgauucc....................................................................................     1
Gi3_28646600_x1...................uucgauAcccggcgccgga..........................................................................     1
Gi1_4175368_x1.................gguucgauucccggcAccggag.........................................................................     1
Gi5_9118019_x1.............uccggguucgauucccggA................................................................................     1
Gi4_568672_x1 ................gUuucgauucccggcgccgga..........................................................................     1
Gi1_4199628_x1..................guucgauucccggcgcccCgagc.......................................................................     1
Gi3_28368155_x1.................ggNucgauucccggcgccgga..........................................................................     1
Gi1_4323736_x1.....................cgauucccggcgccggagcguuA....................................................................     1
Gi1_4274502_x1......caacUgauccggguucgauucccggc................................................................................     1
Gi4_589084_x1 ................gguucgauucccggcAccgg...........................................................................     1
Gi3_28923520_x1.....................cCauucccggcgccggag.........................................................................     1
Gi5_8956080_x1.................gguucgauucccggcCccgga..........................................................................     1
Gi1_4289002_x1cucacccaacagaucGgg..............................................................................................     1
Gi5_8964200_x1.................ggNucgauucccggcgccg............................................................................     1
G1a_6158177_x1.................gguucgauucccggcgcAgga..........................................................................     1
Gi1_4351216_x1.................gguAcgauucccggcgccggagc........................................................................     1
Gi1_4229021_x1....................ucgauucccggcgccggGgc........................................................................     1
Gi4_595029_x1 .................guucgauucccggcgAcgg...........................................................................     1
Gi4_586628_x1 cucacccaacagGuccggguucg.........................................................................................     1
Gi1_4253110_x1..................guucAauucccggcgccggagc........................................................................     1
Gi2_11373735_x1....................Acgauucccggcgccggag.........................................................................     1
Gi1_4315660_x1...............cUgguucgauucccggcgccgg...........................................................................     1
Gi1_4269826_x1..cGcccaacagauccggguucgauuccc...................................................................................     1
Gi3_28297674_x1..................guNcgauucccggcgccgg...........................................................................     1
Gi1_4214366_x1.................gguucgauuAccggcgccggag.........................................................................     1
Gi5_8891075_x1cucacccaaAagauccgg...............................................................................................     1
Gi3_28874542_x1.................gguucgauucAcggcgccgg...........................................................................     1
Gi1_4388420_x1.................gguucgaAucccggcgccgg...........................................................................     1
```

```
Gi3_28716628_x1...................uucgauucccggcgccUgagc........................................................................ 1
Gi3_28868449_x1............auccgggCucgauucccggcgccgga.......................................................................... 1
Gi2_11031774_x1.....................cAauucccggcgccggagcguuuc................................................................... 1
Gi3_28318453_x1.................gguNcgauucccggcgccg............................................................................ 1
Gi2_11295613_x1..................guuGgauucccggcgccggagc........................................................................ 1
Gi2_11350215_x1.................gguucgauucccggUgccggagcguu..................................................................... 1
Gi5_9030840_x1......................gauucccggcgccggagA........................................................................ 1
Gi1_4179217_x1..........agauccggguCcgauuccc................................................................................... 1
Gi5_8959505_x1..................guucgauucccggcgcGgga.......................................................................... 1
Gi3_28742968_x1....cccNacagauccggguucgauuccc................................................................................... 1
Gi5_8988260_x1.ucacccaaNagauccggguu........................................................................................... 1
Gi1a_6130507_x1................gguucgauucccggcAccg............................................................................ 1
Gi2_11311502_x1.............uccggguucgCuucccggcgccgga.......................................................................... 1
Gi3_28605773_x1.................gguGcgauucccggcgccggag......................................................................... 1
Gi2_11024176_x1................ggguucgauucccggUgcc............................................................................. 1
Gi5_8915546_x1.................gguucgUuucccggcgccggagc........................................................................ 1
Gi3_28573501_x1.ucacccaGcagauccggguuc.......................................................................................... 1
Gi1_4261801_x1..................guucgauucccggAgccggagc........................................................................ 1
Gi5_8877020_x1...................uucgauucccggcgccggagcgA...................................................................... 1
Gi5_8843088_x1................Agguucgauucccggcgccgg........................................................................... 1
Gi3_28403797_x1..........agauccggguucgauuGc.................................................................................... 1
Gi5_8949105_x1.................gguucgauucccggcgccggCgcguuu.................................................................... 1
Gi5_8884712_x1...................uucgauuAccggcgccgg........................................................................... 1
Gi3_28335134_x1......caacagGuccggguucga........................................................................................ 1
Gi1a_6024655_x1.................guucgauucccggcgccggC.......................................................................... 1
Gi5_9020591_x1........acagauccgAguucgauucccggcgc.............................................................................. 1
Gi3_28323193_x1...........Cauccggguucgauuccc................................................................................... 1
Gi2_11157678_x1.................gguucgauucccgUcgccggag......................................................................... 1
Gi1_4194882_x1.............uGcggguucgauucccggcgccgga.......................................................................... 1
Gi3_28438328_x1....................ucgauucAcggcgccggagc........................................................................ 1
Gi2_11282194_x1...................uucgauucNcggcgccggag......................................................................... 1
Gi5_8863732_x1.................gguucgauucccggcgccggagcguC..................................................................... 1
Gi5_8975422_x1..................guucgaCucccggcgccggagcguu..................................................................... 1
Gi1_4308261_x1.................gguucgauucccAgcgccg............................................................................ 1
Gi3_28411947_x1...............cggguucgauucccggcgccgCa.......................................................................... 1
Gi1a_6305575_x1.................guucgauucccgCcgccgg........................................................................... 1
Gi5_9012390_x1.................gguucgauucccggcgcUggagcguu..................................................................... 1
Gi5_8965890_x1.....................Agauucccggcgccggagcguuuc................................................................... 1
Gi5_9114512_x1.................gguucgauucccggcgccggagcgCuu.................................................................... 1
Gi4_604906_x1 ..................guucgauucccAgcgccgg........................................................................... 1
Gi5_9003046_x1..................guucgauucccggcgccUga.......................................................................... 1
Gi4_609235_x1 ....................Gcgauucccggcgccggagc........................................................................ 1
Gi4_612632_x1 ..................gGucgauucccggcgccg............................................................................ 1
Gi3_28418808_x1..................guucgauuccGggcgccg............................................................................ 1
Gi2_11326604_x1.................Nguucgauucccggcgccg............................................................................ 1
Gi5_8934544_x1..................guucgauCcccggcgccggag......................................................................... 1
Gi2_11234487_x1....................ucgauucccggcgccgUagc........................................................................ 1
```

Gi3_28941279_x1.................gguucgauucccggcgccgUag...   1
Gi5_8880505_x1....................ucgauucccggUgccgga..   1
Gi3_28715958_x1................ggguCcgauucccggcgccggag...   1
Gi2_11271529_x1.........cagauccggguucgCuucccggcgccggag...   1
Gi5_8861140_x1.............uccggguucgauucccCgc..   1
Gi2_11009016_x1...................uGcgauucccggcgccggag...   1
Gi5_9110145_x1.................gguucgauuccAggcgccgga..   1
Gi3_28983925_x1..........agauccggAuucgauucccggcgccggagc..   1
G1a_6317276_x1.................gguucgauucccggAgccg..   1
Gi2_11381308_x1..................guucgauucccggcgccU..   1
Gi3_28670617_x1....cccaacagauccggguuc..   0
Gi1_4165399_x1..................Nuucgauucccggcgccggagc..   1
Gi5_8876818_x1....cccaacagaucGggguuc..   1
Gi5_8872269_x1..................guucgauucccggcgUcggag...   1
Gi3_28594514_x1................ggguucgauucccggcgccggG..   1
Gi3_28557731_x1.........cagauccggguucgaCuccc...   1
Gi5_9050029_x1...........gauccggguucgauucccggUgccgga..   1
Gi3_28395763_x1......caacagaCccggguucga..   1
Gi1_4247162_x1.................gguucgauucccggAgccggag...   1
Gi3_28534528_x1........aUagauccggguucgauu..   1
G1a_6015802_x1.................gguucgauucccggcgUcg..   1
Gi1_4313075_x1..................guucgauucccggcAccg..   1
Gi2_11284343_x1....................ucgaAucccggcgccggagc..   1
Gi3_28951653_x1..........agauccAgguucgauuccc...   1
Gi3_28912144_x1............auccggguuAgauucccggcgccgga..   1
Gi1_4194051_x1..................guGcgauucccggcgccg..   1
Gi5_8990492_x1.................gguCcgauucccggcgccggagcguu...   1
Gi1_4242195_x1................ggguucgauucccggcgccgU...   1
Gi2_11053413_x1.................ggCucgauucccggcgccgg...   1
Gi5_8840570_x1..................guucgauucccggcgGcggag...   1
Gi3_28943001_x1............auccggguucgauucccggcAccgga..   1
Gi1_4207462_x1...................uucgauucccggcgccggagcCu..   1
Gi4_594417_x1 cucacccaacagauccggguuUg...   1
Gi5_8852998_x1.................gguucAauucccggcgcc...   1
Gi5_8899033_x1.............uccggguuNgauucccggc..   1
G1a_6239732_x1.................gguucgauucccggcgccUgagc..   1
Gi4_582858_x1 ....ggAucgauucccggcgccgg...   1
Gi5_9045643_x1..........agauccggguucgUuuccc...   1
Gi5_8908215_x1.................gguucgUuucccggcgccg..   1
Gi5_8862734_x1..................guucgauucccggcgccAgag...   1
Gi1_4369953_x1.................gguucgauuccAggcgccggagc..   1
Gi3_28434038_x1cucacccaacagauccAg..   1
G1a_6031264_x1..................guucgauucccggcgcAggagc..   1
Gi1_4193613_x1.................gguucgauucccggcgccgAagc..   1
Gi3_28876879_x1.................Aguucgauucccggcgccggagcg...   1
Gi5_8872509_x1................ggguucgCuucccggcgccggagc..   1
Gi3_28469741_x1.................Nguucgauucccggcgcc...   1

```
Gi1_4323888_x1..................gGucgauucccggcgccggag.........................................................................      1
Gi5_8911690_x1......caacagauccggguucgauucccggcgccgg...........................................................................      0
Gi2_11403150_x1.....................cgauucccggcgccggagcguuC....................................................................      1
Gi5_8897780_x1.................gguuAgauucccggcgcc.............................................................................      1
Gi3_28593613_x1....................Ncgauucccggcgccggagc........................................................................      1
Gi3_28615613_x1..........Ugauccggguucgauucccggcgccgg...........................................................................      1
Gi1_4176742_x1.......aacagauccggguucgauucccggUgccgg...........................................................................      1
Gi5_9013166_x1..........Cgauccggguucgauuccc...................................................................................      1
Gi1_4248429_x1..................guucgauuccGggcgccg............................................................................      1
Gi4_582552_x1 .................gguCcgauucccggcgccgg...........................................................................      1
Gi5_9086160_x1...................uucgauucccggcgccggagcAu......................................................................      1
Gi3_28737614_x1....................ucAauucccggcgccgga..........................................................................      1
Gi3_28725190_x1..................guucgauuGccggcgccg............................................................................      1
Gi2_11218470_x1................ggguucgauucccggcgccggagcgA......................................................................      1
Gi5_9128802_x1.................gguucgauucccgCcgccgga..........................................................................      1
Gi5_9131642_x1.................gguucgauucccggcgccggaA.........................................................................      1
Gi2_11007588_x1..................guucgaGucccggcgccggag.........................................................................      1
Gi1_4172795_x1..................guucgUuucccggcgccg............................................................................      1
Gi3_28552848_x1..................guucgauucccggcUccg............................................................................      1
Gi1_4290332_x1..................guucgauucccggcgccggaC.........................................................................      1
Gi5_8952807_x1...................uucgauCcccggcgccgg...........................................................................      1
Gi3_28467988_x1.....................cgauucccggcgccggagcguu.....................................................................      0
Gi3_28555814_x1...................uucgauucUcggcgccggagc........................................................................      1
Gi3_28987564_x1.................gguucgauGcccggcgcc.............................................................................      1
Gi5_8899476_x1.................gguucgauucccggcgUcg............................................................................      1
Gi1_4297345_x1...................uucgauucccggcgccggGgc........................................................................      1
Gi3_28838892_x1............auccgAguucgauucccggcgccgga..........................................................................      1
Gi1_4186097_x1..........agauccggguucgaCuccc...................................................................................      1
Gi1_4264100_x1.......aacagauccggguucgauucccAgcgccgg...........................................................................      1
Gi3_28743439_x1........acagauccggguucgauucccggcgccgAa..........................................................................      1
Gi3_28366159_x1...........gauccggguucgauucccggcgccggagcC.......................................................................      1
Gi2_11292939_x1..................guucgauuUccggcgccggag.........................................................................      1
Gi3_28827616_x1..........agauccggguAcgauuccc...................................................................................      1
Gi2_11264536_x1......................gauucccggcgccgAagc........................................................................      1
Gi2_11351729_x1.............uccggguucgauucccggcgcUgga..........................................................................      1
Gi2_11301148_x1...................uucAauucccggcgccggagc........................................................................      1
Gi5_9030671_x1.................gguucgauucccggGgcc.............................................................................      1
Gi1_4388547_x1.......Gacagauccggguucgauucccggcgccgg...........................................................................      1
Gi3_28968230_x1.ucacccaacCgauccggguuc..........................................................................................      1
Gi1_4202183_x1....................ucgauucccAgcgccggagc........................................................................      1
Gi4_598497_x1 .................gguucgauucccgAcgccgg...........................................................................      1
Gi3_28756476_x1.................gguucgauucccgCcgcc.............................................................................      1
Gi2_11144303_x1..................guucCauucccggcgccggag.........................................................................      1
Gi3_28608683_x1................Cgguucgauucccggcgccg............................................................................      1
Gi3_28975968_x1............auccggguucAauucccggcgccgga..........................................................................      1
Gi1_4338121_x1............auccggguucgGuucccggcgccgg...........................................................................      1
Gi2_11016230_x1...................uucgauucccggcgccAg...........................................................................      1
```

```
Gi2_11376939_x1.................ggNucgauucccggcgccgga..........................................................................     1
G1a_6302868_x1............auccggguucgaCucccggc................................................................................     1
Gi2_11403411_x1cucacccaacUgauccgg..............................................................................................     1
Gi5_9007389_x1..................guucgauucccggcgccgUagc........................................................................     1
Gi1_4171915_x1..................guucgauucUcggcgccg............................................................................     1
Gi2_11304308_x1..................guucgauuGccggcgccggag.........................................................................     1
Gi5_8979444_x1...................Cucgauucccggcgccgg...........................................................................     1
Gi3_28282996_x1.................gguucgauucccggcUccgg...........................................................................     1
Gi5_9077290_x1...................uucgaCucccggcgccggagc........................................................................     1
Gi1_4341708_x1.................gguucgauucccggcgccggagcgG......................................................................     1
Gi5_9000220_x1..................guucgaCucccggcgccggagcguuuc...................................................................     1
Gi5_9035748_x1.................gNuucgauucccggcgccggagc........................................................................     1
G1a_6271337_x1...Gcccaacagauccggguucgauuccc...................................................................................     1
Gi1_4280151_x1..................guucgUuucccggcgccgga..........................................................................     1
Gi1_4333951_x1..................gAucgauucccggcgccg............................................................................     1
Gi3_28323073_x1....cccaacagauccgggCucgauuccc...................................................................................     1
Gi2_11075509_x1...................uucgaAucccggcgccggag.........................................................................     1
Gi2_11311014_x1...................uucgauucccggcgccUgag.........................................................................     1
G1a_6322424_x1....................ucgauucccggcgccggagcCu......................................................................     1
Gi3_28369503_x1............auccggguucgauucccggcUccgga..........................................................................     1
Gi2_11212311_x1.................gguucgauuccGggcgccggag.........................................................................     1
Gi3_28851276_x1......caacagauccggguucAa........................................................................................     1
Gi5_8994404_x1..................Cuucgauucccggcgccggag.........................................................................     1
Gi2_11258334_x1....cccaacagauGcggguuc..........................................................................................     1
Gi5_9116846_x1........................................guuucccgccuuccuucgCcu...................................................     1
Gi3_28665053_x1..........agauccggguucgauuAcc...................................................................................     1
Gi5_8916156_x1..................guucgauuNccggcgccggag.........................................................................     1
Gi2_11239010_x1..................guucgauucccCgcgccgga..........................................................................     1
Gi2_11071155_x1cucacccaacagauccCg..............................................................................................     1
Gi2_11387804_x1......................gaCucccggcgccggagc........................................................................     1
Gi4_591759_x1 ..................guucgauucccggcgccgC...........................................................................     1
Gi3_28554081_x1................ggguucgGuucccggcgccg............................................................................     1
Gi3_28681081_x1......caacagauccggguucgGuucccggcgccgg...........................................................................     1
Gi5_9000909_x1..................guucgauucccggcgccggaUc........................................................................     1
Gi5_9096651_x1...acccaacagauccggguucgaCuccc...................................................................................     1
Gi2_11297972_x1.................ggNucgauucccggcgccggag.........................................................................     1
Gi3_28578239_x1..cacccaacagaucAggguuc..........................................................................................     1
Gi4_569785_x1 cCcacccaacagauccggguucg.........................................................................................     1
Gi1_4275504_x1.................gguucgauucccgAcgcc.............................................................................     1
Gi3_28759523_x1................gAguucgauucccggcgccggagc........................................................................     1
Gi5_8903697_x1.................gguucgauucccggcUccggagc........................................................................     1
Gi2_11168067_x1..................gNucgauucccggcgccggagc........................................................................     1
G1a_6262994_x1.................gguAcgauucccggcgccggag.........................................................................     1
Gi2_11086499_x1...............cggguucgaGucccggcgccgga..........................................................................     1
Gi5_9092387_x1.................gguucgauucccgCcgccg............................................................................     1
Gi2_11219074_x1....cccaacagGuccggguuc..........................................................................................     1
Gi3_28544967_x1............Guccggguucgauucccggcgccgga..........................................................................     1
```

```
Gi1_4224178_x1.................gguucgauucccCgcgccgg...........................................................................     1
Gi2_11055791_x1.................gUuucgauucccggcgccggagcguuu....................................................................     1
Gi1_4328204_x1.................gguucgauucccCgcgcc.............................................................................     1
G1a_6153473_x1.................gguucgauucAcggcgccgga..........................................................................     1
Gi2_11271428_x1.................gguucgaGucccggcgccg............................................................................     1
Gi1_4374162_x1...acccaacagauccggguucgauucUcg..................................................................................     1
Gi1_4283066_x1.................gAuucgauucccggcgcc.............................................................................     1
Gi3_28845642_x1..........agaucAggguucgauucc....................................................................................     1
G1a_6138565_x1.................gguucgauucccggcgccggagcU.......................................................................     1
Gi2_11390964_x1.......aacagauccggguucgGuucccggcgccg............................................................................     1
Gi2_11274941_x1..................guucgauucccggcCccgga..........................................................................     1
Gi2_11294474_x1...................uucgauucccggcAccgg...........................................................................     1
Gi2_11394664_x1.................gguucgauuccGggcgccg............................................................................     1
Gi2_11204160_x1.................gguucgauucccCgcgccgg...........................................................................     1
Gi5_8968586_x1.................gguucgauGcccggcgccgg...........................................................................     1
Gi3_28725258_x1cucacccaNcagauccggguucgauucc....................................................................................     1
Gi3_28383338_x1...............cgUguucgauucccggcgccgga..........................................................................     1
Gi3_28593556_x1............auccAgguucgauucccggcgccgga..........................................................................     1
G1a_6029100_x1.................gguucgauucccAgcgccg............................................................................     1
G1a_6258115_x1..................Auucgauucccggcgccgg...........................................................................     1
Gi3_28658183_x1...............cggguucgauucccggcgccggG..........................................................................     1
Gi3_28671000_x1.................gguucgauucccggcgccggagcgG......................................................................     1
Gi4_572129_x1 .................gguuAgauucccggcgccggagc........................................................................     1
Gi1_4234343_x1.................gguucUauucccggcgccg............................................................................     1
Gi5_9087095_x1.................gguucgCuucccggcgccggagcg.......................................................................     1
Gi3_28344005_x1....cccaacagauccUgguucgauuccc...................................................................................     1
Gi3_28420742_x1cucacccaacagauccgC..............................................................................................     1
Gi5_8850989_x1.................gguucgauucccggcgcUggag.........................................................................     1
G1a_6104292_x1.................gguucgauuccUggcgcc.............................................................................     1
G1a_6280602_x1.................gguucgGuucccggcgccggag.........................................................................     1
Gi5_9038510_x1.................gguucgaAucccggcgccgg...........................................................................     1
Gi1_4367709_x1.........cGgauccggguucgauucccggcgcc.............................................................................     1
Gi2_11388017_x1..................gNucgauucccggcgccgg...........................................................................     1
Gi3_28394896_x1...................uucgauucccUgcgccgga..........................................................................     1
Gi3_28516924_x1...........gauccggguucgauCccc...................................................................................     1
Gi4_600480_x1 ..................guucgUuucccggcgccg............................................................................     1
Gi5_9104674_x1..................guucgauucccgCcgccgga..........................................................................     1
Gi5_8971807_x1.........cagauccgNguucgauucccggcgccg............................................................................     1
Gi1_4236171_x1..........aAauccggguucgauuccc...................................................................................     1
Gi2_11132369_x1......................gauucUcggcgccggagc........................................................................     1
Gi5_9022843_x1....cccaacagauccAgguuc..........................................................................................     1
Gi3_28724358_x1..cacccGacagauccggguuc..........................................................................................     1
Gi3_28505496_x1......Uaacagauccggguucga........................................................................................     1
Gi1_4217533_x1.................gguCcgauucccggcgcc.............................................................................     1
Gi2_11039119_x1............auccggguucgauucccggcgccggagc........................................................................     0
Gi3_28584662_x1......................gauuccAggcgccggagc........................................................................     1
Gi2_11134767_x1cucacccaacagGuccgg..............................................................................................     1
```

```
Gi4_575170_x1 .................ggCucgauucccggcgccggagc........................................................................     1
Gi4_590903_x1 .................gguucgaCucccggcgccgg...........................................................................     1
Gi2_11099575_x1..................guucgauuccAggcgccg............................................................................     1
Gi5_9001238_x1.................gguNcgauucccggcgccg............................................................................     1
Gi3_28444929_x1.............uGcggguucgauucccggcgccg............................................................................     1
Gi5_8913058_x1....................ucgauucccggcgccggGg.........................................................................     1
Gi2_11301120_x1.....................cgauucccNgcgccggagcguuuc...................................................................     1
Gi5_9045519_x1.......aacagauccgggGucgauucccggcgccggagc........................................................................     1
Gi2_11168775_x1....cccaacagauccgggCuc..........................................................................................     1
Gi3_28921023_x1...................uucAauucccggcgccgga..........................................................................     1
Gi2_11330095_x1.................gguGcgauucccggcgccgga..........................................................................     1
Gi3_28441267_x1...................uucgauucccAgcgccgg...........................................................................     1
Gi2_11252634_x1.................gguucgauucccggcgccU............................................................................     1
Gi3_28931829_x1......caacagauccUgguucgauuccc...................................................................................     1
Gi1_4218397_x1.............uccggguucgauGcccggcgccgga..........................................................................     1
Gi2_11106519_x1.ucacccaacagauccggguuc..........................................................................................     0
Gi5_9080958_x1.................gCuucgauucccggcgccggagcgu......................................................................     1
Gi3_28617586_x1.............uccggguucgauucccggcgccggC..........................................................................     1
Gi5_8968028_x1.................gguucgauuccGggcgccggagc........................................................................     1
G1a_6301306_x1.................gguucgauCcccggcgccggagc........................................................................     1
Gi5_9082997_x1..................guucgauucccggcgccggagcg.......................................................................     0
G1a_6044462_x1....................ucgauucccggcgccggagc........................................................................     0
Gi3_28471226_x1...............cggAuucgauucccggcgccgga..........................................................................     1
Gi2_11358279_x1..................guucgauucccggcgccggagcgC......................................................................     1
Gi3_28803494_x1...........gauccggguucgauucAc...................................................................................     1
Gi1_4354265_x1........................................guuucccgccuuccuucgucuu..................................................     0
Gi4_583134_x1 ..................guucgauuccUggcgccgg...........................................................................     1
Gi5_8981725_x1...................Gucgauucccggcgccggagc........................................................................     1
Gi1_4329538_x1..................guucgauuccAggcgccg............................................................................     1
Gi3_28344517_x1..cacccaacUgauccggguuc..........................................................................................     1
G1a_6026247_x1..................guucUauucccggcgccgg...........................................................................     1
Gi2_11112269_x1..........agaucGggguucgauucc....................................................................................     1
Gi1_4211252_x1................gUguucgauucccggcgccgga..........................................................................     1
Gi2_11120609_x1....................ucgauuccAggcgccggag.........................................................................     1
Gi3_28831761_x1...........Aauccggguucgauucccggcgccgga..........................................................................     1
Gi3_28955418_x1......................gauuAccggcgccggagc........................................................................     1
Gi1_4267734_x1............auccgAguucgauucccggcgccgg...........................................................................     1
Gi3_28809496_x1.....................cgauucccAgcgccggagc........................................................................     1
Gi5_9106455_x1............auccggguucgauucccggcgccggagc........................................................................     0
Gi3_28310326_x1...........gauccggguCcgauuccc...................................................................................     1
Gi2_11303749_x1..................guucgaAucccggcgccgg...........................................................................     1
Gi3_28558286_x1..................guucgauucccgAcgccg............................................................................     1
Gi3_28692740_x1cucacccaacaAauccgg..............................................................................................     1
Gi1_4319907_x1..................guuNgauucccggcgccgga..........................................................................     1
Gi1_4347019_x1..................guucgauuAccggcgccg............................................................................     1
Gi3_28281105_x1cucacccaacagauccAgguucgauu......................................................................................     1
Gi2_11077411_x1cAcacccaacagauccgg..............................................................................................     1
```

```
Gi1_4193508_x1......caacagauccggguucgauucUcggc................................................................................ 1
Gi5_9073493_x1.........cagauccggguucgauucccggAgccg............................................................................ 1
Gi2_11053440_x1....................ucgauucccggcgcUggagc........................................................................ 1
Gi3_28955718_x1..caUccaacagauccggguuc.......................................................................................... 1
G1a_6016120_x1...................uucgauucccggcgccggGgc........................................................................ 1
Gi2_11222589_x1....................ucgauucccAgcgccgga.......................................................................... 1
Gi4_595506_x1 .................gguuUgauucccggcgccgg........................................................................... 1
Gi1_4338588_x1.........cagauccggguucgauuccc................................................................................... 0
Gi3_28383516_x1.................gguucgauucccCgcgccgg........................................................................... 1
Gi1_4320439_x1............auccAgguucgauucccggcgccgg........................................................................... 1
Gi3_28964329_x1................ggguGcgauucccggcgccg............................................................................ 1
Gi1_4220275_x1..................guuGgauucccggcgccggagc........................................................................ 1
Gi2_11059354_x1.................gguucgaAucccggcgcc............................................................................. 1
Gi3_28283115_x1..........aAauccggguucgauucc.................................................................................... 1
Gi5_8985201_x1...........gGuccggguucgauucccggcgccgga.......................................................................... 1
Gi2_11185963_x1.................gguucgauucccggcgccggagcAuuuc................................................................... 1
Gi2_11218958_x1......................gauucccggcAccggagc........................................................................ 1
Gi2_11328523_x1.........cagauccgNguucgauuccc................................................................................... 1
Gi5_8957435_x1..................guucgauuccGggcgccg............................................................................ 1
Gi5_9099443_x1........acagauccggguucAauucccggcgc.............................................................................. 1
Gi5_8984487_x1..Uacccaacagauccggguucga........................................................................................ 1
Gi2_11059842_x1.................gguucgauucccggcgccgCa.......................................................................... 1
Gi3_28633401_x1................ggguucgauucccggGgccgga.......................................................................... 1
Gi2_11134506_x1....cccaacagauccgggGuc.......................................................................................... 1
G1a_6047473_x1..................guucgauucccggcgccgCagc........................................................................ 1
Gi2_11294532_x1...................uucgauucccgAcgccgga.......................................................................... 1
G1a_6170784_x1..................guucgauucccggcgccAg........................................................................... 1
Gi1_4205118_x1..................guucgauucccgUcgccggagc........................................................................ 1
Gi3_28636057_x1................gggCucgauucccggcgccg............................................................................ 1
Gi2_11407350_x1....................ucgauucccCgcgccggagc........................................................................ 1
Gi2_11222338_x1..................guNcgauucccggcgccggagc........................................................................ 1
Gi5_9119606_x1.ucaccUaacagauccggguu........................................................................................... 1
Gi3_28827725_x1.............uccggguucgauAcccggcgccgga.......................................................................... 1
G1a_6160626_x1.................gAuucgauucccggcgccggagc........................................................................ 1
Gi4_581032_x1 .................gCuucgauucccggcgccggagc........................................................................ 1
Gi3_28784225_x1...................uucgUuucccggcgccgg........................................................................... 1
Gi5_8958892_x1....................ucgGuucccggcgccggag......................................................................... 1
Gi3_28816522_x1...................uAcgauucccggcgccgg........................................................................... 1
Gi5_9050165_x1..................guuAgauucccggcgccg............................................................................ 1
Gi5_9084990_x1..................guucgauucccggcgccgCagc........................................................................ 1
Gi1_4376683_x1.................gguucgauuUccggcgccg............................................................................ 1
Gi1_4308228_x1................Agguucgauucccggcgccg............................................................................ 1
Gi3_28627834_x1...................uNcgauucccggcgccggagc........................................................................ 1
G1a_6100600_x1.................gguucgauucccAgcgccgg........................................................................... 1
G1a_6353152_x1.................gguucAauucccggcgccggagc........................................................................ 1
Gi4_604495_x1 ......................gGuucccggcgccggagc........................................................................ 1
Gi2_11033012_x1.....................cgaCucccggcgccggagcguuu.................................................................... 1
```

Gi3_28596156_x1.....................cgaNucccggcgccggagc... 1
Gi5_8941816_x1.................gguucUauucccggcgccgg.. 1
Gi1_4308627_x1..................guucgauucccggGgccg... 1
Gi1_4374259_x1cucaccAaacagauccgg... 1
Gi2_11312496_x1....................ucgauAcccggcgccggag.. 1
Gi5_8840045_x1....................ucgauucccggcgccgAa... 1
Gi1_4358284_x1.................gguuAgauucccggcgccg... 1
Gi1_4335481_x1.................gguNcgauucccggcgccgg.. 1
Gi3_28284425_x1........acagauccggguucgaCucccggcgccgg.. 1
Gi3_28402350_x1............auccggguNcgauucccggcgccggagc... 1
Gi2_11303602_x1.................gguucgauucccggcgccggagcguuu... 0
Gi3_28546806_x1.....................cgauucccgAcgccggagc... 1
Gi1_4217748_x1cucacccaaAagauccgg... 1
Gi2_11100601_x1....................ucgauucccggcUccggag.. 1
Gi1_4257778_x1.................gguucgauucccggcgcAg... 1
Gi1_4201497_x1.......aacaUauccggguucgauucccggcgccgg.. 1
Gi3_28686905_x1...................uucgauucccggcAccgg.. 1
Gi5_8876071_x1..................guucgaGucccggcgccgga... 1
Gi2_11269785_x1..................guucgauucccggcgGcggag.. 1
Gi5_8904963_x1.....................cgauucccggcgccggagcg.. 0
Gi3_28748580_x1.....................cgauucccggcgccggagU... 1
Gi5_8865448_x1.............uccggguucgauuAccggc... 1
Gi3_28563903_x1................ggguucgauucccggcgccggGg.. 1
Gi3_28682632_x1cucacccaacagaucccCg.. 1
Gi3_28559884_x1....ccUaacagauccggguucgauuccc.. 1
Gi5_9095907_x1..................guNcgauucccggcgccgg.. 1
Gi3_28341037_x1..................guucgauucccgCcgccggagc... 1
Gi1_4247730_x1...................uucgCuucccggcgccgg.. 1
Gi3_28576477_x1....................ucAauucccggcgccggag.. 1
Gi3_28960986_x1....................ucgauucAcggcgccggag.. 1
Gi3_28782911_x1................ggguucgGuucccggcgccggag.. 1
Gi1_4218610_x1...................uucgauuccAggcgccgg.. 1
Gi3_28584570_x1...................Cucgauucccggcgccgg.. 1
Gi2_11218230_x1.................gguucgauucccggAgccgg.. 1
G1a_6151198_x1.................gguucgauuUccggcgccggagc... 1
Gi3_28604818_x1....................ucgauCcccggcgccggag.. 1
Gi2_11300518_x1.........Gagauccggguucgauucccggcgccggag.. 1
Gi5_8867507_x1........acagaucGggguucgauucccggcgc... 1
Gi1_4377282_x1.ucacccaacagauccggAu... 1
Gi3_28346100_x1............auccggguucgauucccggcC.. 1
Gi5_9066493_x1..................guucgauucccggcgccgUa... 1
Gi3_28337109_x1..................guucgauGcccggcgccgg.. 1
Gi2_11238491_x1..................guucgauucccggcgccggaC.. 1
Gi5_9119065_x1...acccaacagauccgggCucgauucc... 1
Gi2_11298164_x1....................uGgauucccggcgccgga... 1
Gi5_8974533_x1....cccaacagCuccggguuc... 1
Gi3_28352573_x1.....................Ggauucccggcgccggag.. 1

```
Gi2_11299054_x1....................ucgauuAccggcgccggagc........................................................................     1
Gi2_11164530_x1..................guucgauucccUgcgccgga..........................................................................     1
Gi5_8895238_x1.................gguucgauCcccggcgccggagcguuu....................................................................     1
Gi2_11231686_x1......................gauucccggcgccggCgc........................................................................     1
G1a_6308753_x1..................guucgauuNccggcgccggagc........................................................................     1
Gi3_28728177_x1..........agauccgggCucgauucc....................................................................................     1
Gi2_11092858_x1..................guucgauucccggcUccgga..........................................................................     1
Gi1_4307504_x1...................Aucgauucccggcgccgg...........................................................................     1
Gi2_11275583_x1.........cagauccggguucgauucccggcgccggCg.........................................................................     1
Gi5_8878709_x1cucacccaacagauccCg..............................................................................................     1
Gi2_11182677_x1...............Uggguucgauucccggcgccggagc........................................................................     1
Gi2_11311702_x1..................guucgauucccgUcgccgga..........................................................................     1
Gi5_9082065_x1.................gguucgauuGccggcgccgg...........................................................................     1
Gi1_4171366_x1.................gguucgauucccggcgcAgga..........................................................................     1
Gi4_606396_x1 ..................guucgaGucccggcgccgg...........................................................................     1
Gi1_4364259_x1.................gguucgauucAcggcgccggag.........................................................................     1
Gi1_4228899_x1.................gguucgauuccGggcgccggag.........................................................................     1
Gi4_594121_x1 .................gguucgaCucccggcgccggagc........................................................................     1
G1a_6344008_x1.................gguucgauucccggcgccA............................................................................     1
Gi2_11273164_x1...................uucgauucccggcgccgC...........................................................................     1
Gi3_28647113_x1................ggguucgauucccggcgUcgga..........................................................................     1
Gi3_28688297_x1.................gguAcgauucccggcgcc.............................................................................     1
Gi5_8946289_x1.uGacccaacagauccggguu...........................................................................................     1
Gi5_9082625_x1...................uucgCuucccggcgccgg...........................................................................     1
Gi5_8865141_x1..................guuNgauucccggcgccggagc........................................................................     1
Gi3_28562024_x1...................uucgauuccAggcgccggagc........................................................................     1
Gi2_11144917_x1.....................cgauucccggcgccggaAcguuu....................................................................     1
Gi5_8889832_x1..................guucgauucccCgcgccggagc........................................................................     1
Gi4_575202_x1 .....................cgauucccggcgccggagcC.......................................................................     1
Gi3_28526016_x1.....................cgauucccggcgUcggag.........................................................................     1
Gi1_4368827_x1.................gguucgauucccggcgccCgagc........................................................................     1
Gi5_8893376_x1.................gguuGgauucccggcgccggag.........................................................................     1
Gi3_28482553_x1.Ccacccaacagauccggguuc..........................................................................................     1
Gi2_11278047_x1..............ccCgguucgauucccggcgccggagc........................................................................     1
Gi3_28459091_x1...................uucgGuucccggcgccgga..........................................................................     1
Gi3_28619795_x1...............cggguucgauuGccggcgccgga..........................................................................     1
G1a_6174982_x1.................gguucgauucccCgcgccggag.........................................................................     1
Gi3_28530015_x1.....ccaacagauccggguucgauucccggcgccggagc........................................................................     0
Gi1_4257465_x1.................gguucgauAcccggcgccggag.........................................................................     1
Gi3_28663955_x1......caUcagauccggguucgauucccggcgccgg...........................................................................     1
Gi3_28876102_x1.............uccggguucgauucccggUgccgga..........................................................................     1
Gi3_28881903_x1.............Cccggguucgauucccggcgccg............................................................................     1
Gi5_9126598_x1....cccaGcagauccggguucgau.......................................................................................     1
Gi3_28984545_x1.ucacccaacagauccgAguuc..........................................................................................     1
Gi2_11058683_x1.........cagauccggguucgauucccggcgccggagcC.......................................................................     1
Gi5_9120741_x1.................gguNcgauucccggcgcc.............................................................................     1
Gi3_28577470_x1..........agauccggguuAgauuccc...................................................................................     1
```

```
G1a_6310241_x1..................Cuucgauucccggcgccg.............................................................................    1
Gi5_9124154_x1..................guuAgauucccggcgccgga...........................................................................    1
Gi4_573755_x1 ...................uucgauucccggcgccgg............................................................................    0
Gi3_28709885_x1..................guucgauuGccggcgccggagc.........................................................................    1
Gi2_11209690_x1..................guucgauuccGggcgccgga...........................................................................    1
Gi2_11130143_x1.........cagauccggguucgauucccggcgccAgag..........................................................................    1
Gi3_28545311_x1.......aacagauccggguucgauucccggcgUcgga...........................................................................    1
Gi2_11244713_x1....................ucgauucccggcgccAga...........................................................................    1
Gi3_28373286_x1............auccgggGucgauucccggcgccgga...........................................................................    1
Gi3_28963190_x1cucacccaacGgauccgg...............................................................................................    1
Gi5_8994262_x1................ggguucgauucccggcgccggagcC........................................................................    1
Gi5_9069420_x1.ucacccUacagauccggguu............................................................................................    1
Gi1_4355106_x1.................gCuucgauucccggcgccggag..........................................................................    1
Gi4_569538_x1 ..................guucUauucccggcgccgga...........................................................................    1
Gi3_28740307_x1......................gauucccggcgccggagcgA.......................................................................    1
Gi2_11010459_x1....................ucgaCucccggcgccgga...........................................................................    1
Gi5_9119908_x1...................uucgauucccggcgcAgg............................................................................    1
Gi3_28908752_x1cucacccaCcagauccgg...............................................................................................    1
Gi4_570039_x1 ..................guucgauucccggcAccggag..........................................................................    1
Gi3_28423031_x1.........cagauccggguucgauucccggcgccgC............................................................................    1
Gi5_8961465_x1.................gguuUgauucccggcgccggagcguuu.....................................................................    1
Gi5_9107189_x1cAcacccaacagauccgg...............................................................................................    1
Gi2_11358228_x1....................ucgauuccGggcgccggagc.........................................................................    1
Gi3_28905466_x1cucUcccaacagauccgg...............................................................................................    1
Gi1_4278280_x1cucacccaacagauccggguucgauucA.....................................................................................    1
G1a_6202501_x1.................gguucgauNcccggcgcc..............................................................................    1
Gi5_8985112_x1....................ucgauucccggAgccggag..........................................................................    1
Gi2_11285736_x1.....................cgauucccggcgccggCgcguuu.....................................................................    1
Gi3_28313780_x1....cccaacagauccggguucgauucUc....................................................................................    1
Gi2_11287437_x1...........gauccggguucgauucccggcgccgga...........................................................................    0
Gi1_4266129_x1.................gguucgauucccggcCccggagc.........................................................................    1
G1a_6079268_x1.................gguucgauuAccggcgccggagc.........................................................................    1
Gi3_28903629_x1.............uccggguucgaCucccggcgccgga...........................................................................    1
Gi3_28552888_x1cucacccaacagaucGgg...............................................................................................    1
Gi2_11046574_x1.................gguucgauGcccggcgcc..............................................................................    1
Gi2_11362987_x1.................gguucgauucccCgcgcc..............................................................................    1
Gi2_11060389_x1....................ucgauucccggcgGcggagc.........................................................................    1
Gi2_11009191_x1....cccaacagNuccggguuc...........................................................................................    1
Gi2_11160678_x1.............uccggguucgauucccggcgccggagc.........................................................................    0
Gi3_28507336_x1....................ucgauucccggcgAcgga...........................................................................    1
Gi5_8948057_x1................Cgguucgauucccggcgccggagc.........................................................................    1
Gi5_8907107_x1.................gguuAgauucccggcgccgg............................................................................    1
Gi5_8907632_x1.ucacccaCcagauccggguu............................................................................................    1
Gi1_4218050_x1.................gguucgauucccggcgccgCag..........................................................................    1
Gi2_11037431_x1..........agauccggguucgCuuccc....................................................................................    1
Gi1_4350415_x1...................uucgauucccggcgccAgagc.........................................................................    1
Gi1_4295786_x1..................guuAgauucccggcgccgga...........................................................................    1
```

```
Gi1_4316550_x1.................gguucgauAcccggcgccggagc........................................................................     1
Gi3_28688159_x1.....................cAauucccggcgccggag.........................................................................     1
Gi5_9017324_x1..cacccaaUagauccggguucga........................................................................................     1
Gi1_4317254_x1.................gguuAgauucccggcgccggagc........................................................................     1
Gi5_9019155_x1...................uucgauucccggcgccgA...........................................................................     1
G1a_6108774_x1.................gguucgauGcccggcgccgga..........................................................................     1
Gi2_11059441_x1.................gguucgauucAcggcgccgg...........................................................................     1
Gi3_28890051_x1..................guucgauucccUgcgccggag.........................................................................     1
Gi1_4175714_x1.................gguucgauucccggcgGcggagc........................................................................     1
Gi3_28765865_x1......................gauucccgUcgccggagc........................................................................     1
Gi2_11000074_x1....................ucgaGucccggcgccggag.........................................................................     1
Gi2_11111681_x1..................guucgauuccGggcgccggagc........................................................................     1
Gi5_8838262_x1.................gguucgauAcccggcgccgga..........................................................................     1
G1a_6035510_x1..................guucgaCucccggcgccggag.........................................................................     1
Gi3_28543581_x1......caacGgauccggguucga........................................................................................     1
Gi5_9038197_x1.................gguucUauucccggcgcc.............................................................................     1
Gi2_11215724_x1..................guuNgauucccggcgccggag.........................................................................     1
Gi1_4202829_x1.................gguuGgauucccggcgccggagc........................................................................     1
Gi3_28609483_x1....................ucgauucccggcgccggUgc........................................................................     1
Gi5_8857226_x1..................guucgauucccggcgccggagcgu......................................................................     0
Gi2_11360205_x1.....................cgauucccggcgcUggagcguuu....................................................................     1
Gi1_4275075_x1.........cagauccgggCucgauucccggcgcc.............................................................................     1
Gi3_28915508_x1......................gauucccggcgccAgagc........................................................................     1
Gi4_594361_x1 Aucacccaacagauccggguucg.........................................................................................     1
G1a_6084214_x1.................gguucgauucccggcgUcggag.........................................................................     1
Gi3_28565650_x1......caacagauccggguucgauCccc...................................................................................     1
Gi5_9127215_x1.................gguucUauucccggcgccg............................................................................     1
Gi3_28873117_x1cucGcccaacagauccggguucgauu......................................................................................     1
Gi5_9107706_x1..................guucgauuccGggcgccgga..........................................................................     1
Gi3_28987095_x1....cccaacagauccggguucgaCuccc...................................................................................     1
Gi1_4262903_x1..................guucgCuucccggcgccggagc........................................................................     1
Gi1_4228821_x1cGcacccaacagauccggguucgauucc....................................................................................     1
Gi3_28405141_x1............auccggguCcgauucccggcgccggagc........................................................................     1
Gi1_4191676_x1....................ucgauucccggcgccgga..........................................................................     0
Gi5_8902325_x1................Agguucgauucccggcgccggag.........................................................................     1
Gi3_28492981_x1.............uccggguucgGuucccggcgccg............................................................................     1
Gi5_9063088_x1..................guucgauucccggcCccgga..........................................................................     1
G1a_6087353_x1.................gguucgauucccCgcgccgga..........................................................................     1
Gi1_4220382_x1..................guuAgauucccggcgccggagc........................................................................     1
Gi3_28550482_x1cucaUccaacagauccgg..............................................................................................     1
Gi5_8923941_x1cucacAcaacagauccgg..............................................................................................     1
Gi3_28370921_x1................ggguuAgauucccggcgccggagc........................................................................     1
Gi4_586758_x1 .................gguucgauucUcggcgccgg...........................................................................     1
Gi3_28412858_x1.....................cgauucccggcgccggagcU.......................................................................     1
Gi3_28702826_x1.....................cgGuucccggcgccggag.........................................................................     1
Gi2_11122381_x1...................uucgauucccggUgccggag.........................................................................     1
Gi5_9048020_x1.................gguucgauucccgUcgccggagc........................................................................     1
```

Gi2_11114223_x1..........Ggauccggguucgauucc... 1
Gi2_11272735_x1...............cggguucgauCcccggcgccgga... 1
Gi3_28628330_x1.............uccggguucgauucccggcgccggG... 1
Gi3_28751554_x1....................Acgauucccggcgccggagc... 1
Gi2_11134309_x1...............cgggGucgauucccggcgccgga... 1
Gi1_4166191_x1cucacccaacagauccggguucgG... 1
Gi2_11164250_x1.................gguucgauucccggcgccggaU.. 1
Gi5_9018585_x1.................gguucgaAucccggcgccggag.. 1
Gi2_11228306_x1....................ucAauucccggcgccggag.. 1
Gi2_11227077_x1..................guucgauucccggcgcGg... 1
Gi2_11289923_x1....................ucgNuucccggcgccggagc... 1
Gi3_28538696_x1...........gauccggguucgauAccc.. 1
Gi5_8915832_x1....cccaacagauccggguAc... 1
Gi5_8833470_x1........acUgauccggguucgauucccggcgc... 1
Gi5_8891167_x1cucacccaUcagauccgg... 1
Gi5_8947925_x1..................guucgauucccggcgccgAag.. 1
Gi5_8906643_x1...........gauccggguucgCuucccggcgccgga... 1
Gi3_28962402_x1....................ucgauucccggcgccggCg.. 1
Gi1_4367466_x1...acccaacaAauccggguucgauuccc.. 1
Gi3_28324364_x1.................gguucgauucccggcgcGg... 1
Gi4_583265_x1 ....guucgauucUcggcgccgga... 1
Gi3_28767597_x1......................gauucccggcgUcggagc... 1
Gi4_595786_x1 ....guucgCuucccggcgccgg.. 1
Gi5_9068058_x1.....................cgauucccCgcgccggagcguuuc.. 1
Gi2_11372612_x1...........gauccggguucgauuccc.. 0
Gi2_11022110_x1.....................cgauuccUggcgccggagcguuuc.. 1
G1a_6299274_x1.................gguucgauucccUgcgccggag.. 1
G1a_6130492_x1..................guucUauucccggcgccggagc... 1
Gi1_4195490_x1..................guucgauucccggcgccggaAc... 1
Gi2_11261799_x1.................gguucgauuAccggcgccggag.. 1
Gi2_11237992_x1...................uucgauuccUggcgccggagc... 1
Gi5_9088187_x1..............ccggguucgauucccggcgccggU... 1
Gi3_28973006_x1.........cagauccgggGucgauucccggcgccgg.. 1
Gi2_11070776_x1....................ucgauucccggcgccUgag.. 1
Gi1_4323829_x1.................gguucgauGcccggcgccgga... 1
Gi3_28378571_x1................ggguucgGuucccggcgccggagc... 1
Gi3_28466710_x1.................Nguucgauucccggcgccg... 1
Gi2_11324069_x1...............cAgguucgauucccggcgccgga... 1
Gi3_28985193_x1................ggguucgauucccggcgccggaCc... 1
Gi3_28678017_x1................ggguucgauucccggcgccAgagc... 1
Gi5_9129758_x1.................gguucgauucccggcgcGg... 1
Gi3_28742472_x1......................gauuccGggcgccggagc... 1
Gi3_28632715_x1cucacccaacagauccggCu... 1
Gi2_11386878_x1..................guucgauucGcggcgccg... 1
Gi1_4278256_x1..................guucgauuGccggcgccggagc... 1
Gi4_588102_x1 ....guucgauucccggcgccggag.. 0
Gi2_11115133_x1.................gguuAgauucccggcgcc.. 1

```
Gi1_4274785_x1......caacagauccgAguucgauucccggc................................................................................      1
Gi2_11355533_x1.............uccggguucgauAcccggcgccgga..........................................................................     1
Gi1_4352544_x1....cccaacagauccggguuc..........................................................................................      0
Gi3_28628149_x1.................gUuucgauucccggcgccggagcg.......................................................................     1
Gi5_8921422_x1...acccaacagGuccggguucgauucc....................................................................................      1
Gi3_28944552_x1...................Aucgauucccggcgccgga..........................................................................     1
Gi3_28980890_x1..........agGuccggguucgauuccc...................................................................................     1
Gi1_4160066_x1.................gguucgUuucccggcgccggag.........................................................................      1
Gi5_8982493_x1.................gguucgauucccggcgGcg............................................................................      1
Gi3_28440888_x1.........cagauccggguucgauucccggcgGcgg...........................................................................     1
Gi2_11291313_x1.................gguucgauucccggcCccgga..........................................................................     1
G1a_6239905_x1.................gguucgauuccUggcgccggagc........................................................................      1
Gi2_11005910_x1.........caUauccggguucgauucccggcgccgga..........................................................................     1
Gi5_8947288_x1.................gguucgaAucccggcgccggagc........................................................................      1
Gi2_11054287_x1.................gguucgauucccgUcgccg............................................................................     1
Gi5_9045459_x1................Agguucgauucccggcgccggagc........................................................................      1
Gi5_8857061_x1..................guucgauucccggcgccggCg.........................................................................      1
Gi5_8907661_x1.................Aguucgauucccggcgccggagcguu.....................................................................      1
Gi5_8906375_x1cucacccaacagauccgA..............................................................................................      1
Gi3_28837471_x1......................gauCccggcgccggagc.........................................................................     1
Gi5_8893444_x1....................ucgauucccggcgccgCagc........................................................................      1
Gi3_28787178_x1....................ucgauuAccggcgccggag.........................................................................     1
Gi2_11271538_x1....................ucgauucAcggcgccgga..........................................................................     1
Gi2_11390696_x1..........agauccggguucAauucc....................................................................................     1
Gi1_4354254_x1..................guucgCuucccggcgccg............................................................................      1
Gi5_9050629_x1...acccCacagauccggguucgauuccc...................................................................................      1
Gi2_11110040_x1..................guucgauucccggGgccg............................................................................     1
Gi1_4250698_x1.........cagauccggguucgauucccggcgAc.............................................................................      1
Gi5_8981188_x1..................guucgauuGccggcgccggagc........................................................................      1
Gi1_4390598_x1..................guucgauGcccggcgccgg...........................................................................      1
Gi3_28689156_x1....................ucgauucccgAcgccggag.........................................................................     1
Gi3_28424338_x1...........gauccggguucgauucccggU................................................................................     1
Gi2_11089352_x1......................Cauucccggcgccggagc........................................................................     1
G1a_6183164_x1.................Aguucgauucccggcgcc.............................................................................      1
Gi5_9048238_x1.................gguucgaAucccggcgccgga..........................................................................      1
Gi3_28398881_x1.ucacccaacagauccggguCc..........................................................................................     1
Gi4_592962_x1 .................gAuucgauucccggcgccggagc........................................................................      1
Gi3_28624239_x1cCcacccaacagauccggguucgauucc....................................................................................     1
Gi2_11362498_x1.................gguucgauucccUgcgccgg...........................................................................     1
Gi5_9023466_x1.................gguucgauuccAggcgccgg...........................................................................      1
G1a_6272640_x1..................guCcgauucccggcgccggagc........................................................................      1
Gi4_575011_x1 .......................auucccggcgccggagcC.......................................................................      1
Gi2_11276863_x1cucacccaaUagauccgg..............................................................................................     1
G1a_6340938_x1.................gguCcgauucccggcgccggagc........................................................................      1
G1a_6280899_x1.................gguucgauucccggcgccggagcgA......................................................................      1
Gi1_4190703_x1..........agauccggguucgauuAcc...................................................................................      1
G1a_6098600_x1.................gguucgauucccggcAcc.............................................................................      1
```

```
Gi2_11365591_x1....................ucgauucccggcgccggGg.........................................................................  1
Gi3_28728864_x1................Agguucgauucccggcgccgga..........................................................................  1
Gi3_28582693_x1...............Uggguucgauucccggcgccggag.........................................................................  1
Gi5_8886831_x1.................gguucgauuccUggcgccggagcguu.....................................................................  1
Gi3_28399065_x1.............uccggguuUgauucccggcgccgga..........................................................................  1
Gi2_11271890_x1....................ucgauucccggcgccgAa..........................................................................  1
Gi2_11386236_x1..........agaNccggguucgauucc....................................................................................  1
Gi3_28974909_x1...................uucgauucccggAgccggagc........................................................................  1
Gi3_28835913_x1.............uccggguucgauucccggcgccAga..........................................................................  1
G1a_6216070_x1.................Cguucgauucccggcgcc.............................................................................  1
Gi5_8878632_x1cucacccaacaAauccgg..............................................................................................  1
Gi1_4374816_x1....................Ccgauucccggcgccggagc........................................................................  1
Gi3_28625718_x1................ggguucgaCucccggcgccggag.........................................................................  1
Gi1_4295209_x1.................gguGcgauucccggcgccgg...........................................................................  1
Gi2_11124518_x1.................gguucgauAcccggcgccgga..........................................................................  1
Gi3_28607644_x1cucacccaacUgauccgg..............................................................................................  1
Gi3_28652680_x1.....................cgauucccggcgccggCg.........................................................................  1
Gi1_4356054_x1cucacccaacagauccggguucgauuccU...................................................................................  1
Gi1_4346213_x1..................guucgaAucccggcgccg............................................................................  1
Gi1_4356775_x1..................guuAgauucccggcgccg............................................................................  1
Gi2_11015154_x1...acccaacagauccggguucgauucc....................................................................................  0
Gi3_28528581_x1.....................cAauucccggcgccggagc........................................................................  1
Gi3_28851294_x1..caccUaacagauccggguuc..........................................................................................  1
Gi1_4203173_x1Nucacccaacagauccgg..............................................................................................  1
Gi1_4207197_x1.................gguucgauCcccggcgccggag.........................................................................  1
Gi3_28462976_x1.................gguuNgauucccggcgccggagc........................................................................  1
Gi5_9135834_x1..................guucgauuGccggcgccgga..........................................................................  1
Gi5_8853453_x1..................guucgauucccgUcgccggag.........................................................................  1
Gi1_4186427_x1..................guucAauucccggcgccggag.........................................................................  1
G1a_6336195_x1..................gGucgauucccggcgccgga..........................................................................  1
Gi5_8957526_x1.......aacagauccggAuucgauucccggcgccggagc........................................................................  1
Gi2_11173314_x1.................gNuucgauucccggcgccggagc........................................................................  1
Gi2_11118619_x1.....................cgUuucccggcgccggagcguuu....................................................................  1
Gi1_4321648_x1..cacccaacagauccggguucgCuuccc...................................................................................  1
Gi1_4223970_x1..................guucgauucccggcgcGgga..........................................................................  1
Gi3_28811273_x1.................gguucCauucccggcgccggag.........................................................................  1
Gi2_11041101_x1.................gguucgauucAcggcgccgga..........................................................................  1
Gi5_8965383_x1.................gguucgauucccUgcgcc.............................................................................  1
Gi1_4349061_x1........Gcagauccggguucgauuccc...................................................................................  1
Gi1_4316738_x1........................uucccggcgccggagcCu......................................................................  1
Gi5_9020413_x1...acccaacagauccggguucgauucUc...................................................................................  1
Gi3_28865077_x1..........agauccgggAucgauuccc...................................................................................  1
Gi1_4343697_x1cucacccaacagauccggU.............................................................................................  1
Gi3_28439388_x1.................gNuucgauucccggcgccggagc........................................................................  1
Gi4_596185_x1 ...................ucgauucccggcgccggagcC.......................................................................  1
Gi5_9112113_x1..............ccgggCucgauucccggcgccgga..........................................................................  1
Gi1_4280852_x1.............uccggguucgaCucccggcgccgga..........................................................................  1
```

Gi4_612650_x1 ....guucgauucccggcgUcgga.. 1
Gi1_4378852_x1..........agauccgguucgauucUc.. 1
Gi1_4195659_x1............Uuccgguucgauucccggcgccgga... 1
Gi3_28418507_x1...................uucgauuGccggcgccggagc.. 1
Gi2_11150518_x1.................gguucgauucccggcgGcggag... 1
Gi5_9082398_x1...................uucgGuucccggcgccgg... 1
Gi1_4329162_x1............Guccgguucgauucccggcgccgga... 1
Gi5_8871802_x1cucacccaacagaucAgg... 1
Gi3_28829414_x1cucacccaacagauccgA.. 1
Gi3_28675635_x1......caacagauccAgguucga.. 1
Gi3_28419469_x1.............uccgguucgauucccggAgccgga... 1
Gi3_28686329_x1..........agCuccgguucgauucc... 1
Gi5_8918949_x1....................ucgGuucccggcgccggagc.. 1
G1a_6037754_x1.................gguucgauucccggcgccgAag... 1
Gi3_28391790_x1.................Uguucgauucccggcgccggagcguu... 1
Gi2_11295437_x1.......aacagauccggguAcgauucccggcgccg.. 1
Gi4_571218_x1 ....gguucgauucccggcgcA... 1
Gi3_28333127_x1........Ucagauccggguucgauuccc... 1
Gi2_11264212_x1.................gguucgauucccggcgccggUg... 1
G1a_6083325_x1.................gguucgauucccggAgccgg... 1
G1a_6330116_x1..................guucgauucccggcgGcggagc.. 1
Gi3_28632935_x1.ucaccUaacagauccggguuc.. 1
Gi3_28310905_x1....................ucgauucUcggcgccgga.. 1
Gi3_28315274_x1.........cagaGccggguucgauucccggcgccgg... 1
G1a_6289377_x1.................gguucgauucccggcgccggGg... 1
Gi2_11034387_x1.............uccggguucgaCucccggcgccgga.. 1
Gi3_28972842_x1......................gauucccCgcgccggagc.. 1
Gi2_11062455_x1..................guucgauucccggcgccggagcg... 0
Gi3_28293012_x1....................ucgauuccAggcgccggag... 1
Gi2_11331587_x1..................guNcgauucccggcgccgg... 1
Gi2_11346832_x1..........agauccggguucgCuucc.. 1
Gi5_8954286_x1......................gauucccggcgccggaUc.. 1
G1a_6279999_x1..................guucgauucccAgcgccggagc.. 1
Gi2_11299356_x1..........agauccgAguucgauucc.. 1
Gi1_4328694_x1.....................cgauucccggcgccggagc.. 0
Gi2_11043616_x1..................guucgUuucccggcgccgga.. 1
Gi5_9068649_x1.................gguucgauucGcggcgccggagc.. 1
Gi3_28912503_x1......................gauucccggcgccggaAc.. 1
Gi2_11223709_x1....................ucgauuUccggcgccggag... 1
Gi2_11161412_x1cucacccaacagauccAg.. 1
Gi3_28458350_x1..................guucgauucccggcUccggag... 1
Gi4_591325_x1 ....guucgauucUcggcgccgg... 1
Gi1_4197287_x1.......................auucccggcgccggagcU... 1
Gi2_11340941_x1...................uucgCuucccggcgccgg... 1
G1a_6257853_x1.................gguucgauCcccggcgccggag... 1
Gi5_9086464_x1.................Aguucgauucccggcgccggagcgu.. 1
Gi3_28894200_x1cucacccaGcagauccgg.. 1

```
Gi3_28422376_x1.............uccggguucgauucccAgcgccgga..........................................................................     1
Gi4_579742_x1 ..................guCcgauucccggcgccgg...........................................................................      1
Gi2_11012929_x1..................guAcgauucccggcgccg............................................................................     1
>GLCHR05_6862
The candidate has been discarded because the structure is inconsistent with Dicer processing:
the candidate mature sequence does not form part of a likely miRNA duplex
exp           nnnnnnnnnnnnnnnnnnnnnnnnnnnnnnnnnnnnnnnnnnnnnnnnnnnnnnnnnnnnnnnnnnnnnnnnnnnnnnnnnnnnnnnnnnnnnnnnnnnnnnnnnnnnnnnn
pri_seq       aucuggcgcgcccccaguccggcguaguauaacggcuagaaugguugguucucacccaacagauccggguucgauucccggcgccggagcguuucccgccuuccuucgucuu
pri_struct    ....(((((((......(((((((((((......)))).....(((((.......)))))....(((((.......))))))))))))))).....))))............      #MM



Gi3_8900428_x63185...................................................................gguucgauucccggcgccggagc......................  0
Gi3_9682832_x43166.................uccggcguaguauaacggcuaga........................................................................  0
Gi3_9927636_x38745...................................................................gguucgauucccggcgccggagcC.....................  1
Gi2_2216035_x30808...................................................................gguucgauucccggcgccggagc......................  0
Gi1_1331494_x26933....................................................................guucgauucccggcgccgg.........................  0
Gi3_12301936_x14323...................................................................gguucgauucccggcgccgga........................ 0
Gi3_12467744_x13384....................................................................guucgauucccggcgccgg......................... 0
Gi3_12507553_x13080....................................................................guucgauucccggcgccggagcC..................... 1
Gi2_2855789_x12876...................................................................gguucgauucccggcgccggagcC.....................  1
Gi3_12546417_x12822...................................................................gguucgauucccggcgccggag....................... 0
Gi3_12730796_x11530....................................................................guucgauucccggcgccgga........................ 0
Gi3_12799683_x11385.................uccggcguaguauaacggcua.......................................................................... 0
Gi5_3589931_x10963....................................................................guucgauucccggcgccgg.........................  0
Gi2_3032616_x10345....................................................................guucgauucccggcgccggagc......................  0
Gi5_3654433_x9879....................................................................guucgauucccggcgccg..........................   0
Gi3_13160908_x9669....................................................................guucgauucccggcgccggagc......................  0
Gi2_3177371_x8982....................................................................guucgauucccggcgccgg.........................   0
Gi3_13264247_x8954.................uccggcguaguauaacgg.............................................................................  0
Gi2_3195303_x8882.................uccggcguaguauaacggcuaga........................................................................   0
Gi5_3728878_x8684...................................................................gguucgauucccggcgccggagc......................   0
Gi3_13351844_x8572.................uccggcguaguauaacggc............................................................................  0
Gi5_3795072_x7593.................uccggcguaguauaacggcuaga........................................................................   0
Gi5_3810050_x7380.................uccggcguaguauaacggcuag.........................................................................   0
Gi3_13864308_x6910...................................................................gguucgauucccggcgccg..........................  0
Gi5_3872205_x6628..............................................gguucucacccaacagauccg.............................................   0
Gi2_3537957_x6599....................................................................guucgauucccggcgccggagcC.....................   1
Gi3_14018747_x6489...................................................................Uguucgauucccggcgccggagc......................  1
Gi3_14430267_x5377....................................................................guucgauucccggcgccggag.......................  0
Gi5_3990121_x5185...................................................................gguucgauucccggcgccggagcC.....................   1
Gi3_14604826_x4914...................................................................gguucgauucccggcgccgg.........................  0
Gi1_1946380_x4673...................................................................gguucgauucccggcgccgg.........................   0
Gi3_14790128_x4604...................................................................gUuucgauucccggcgccggagc......................  1
Gi1_1960321_x4547.................uccggcguaguauaacgg.............................................................................   0
Gi2_3825436_x4527...................................................................gguucgauucccggcgccggag.......................   0
Gi5_4103580_x4349...............................................guucucacccaacagauccg.............................................   0
```

```
Gi2_3883487_x4338...................................................................gguucgauucccggcgcc...........................  0
Gi5_4133188_x4096...................................................................gguucgauucccggcgccg..........................  0
Gi3_15101518_x4074.................uccggcguaguauaacggcuag.........................................................................  0
Gi1_1995350_x4001.................uccggcguaguauaacggcuag.........................................................................  0
Gi3_15217882_x3936..............................................gguucucacccaacagauccg.............................................  0
Gi5_4205184_x3893.................uccggcguaguauaacggcua..........................................................................  0
Gi5_4228222_x3725.................uccggcguaguauaacgg.............................................................................  0
Gi3_15427175_x3678.................uccggcguaguauaacggcuagaau......................................................................  0
Gi5_4301044_x3513.................uccggcguaguauaacggcuagaau......................................................................  0
Gi3_15580261_x3458...................................................................gguucgauucccggcgcc...........................  0
Gi2_4129219_x3450...................................................................gguucgauucccggcgccg..........................  0
Gi5_4325292_x3416.................uccggcguaguauaacggc............................................................................  0
Gi5_4342263_x3360...................................................................gguucgauucccggcgccgga........................  0
Gi1_2046517_x3321.............................................ugguucucacccaacagauccgg............................................  0
Gi1_2049838_x3306...............................................guucucacccaacagauccg.............................................  0
Gi1_2059731_x3261................................................uucucacccaacagauccg.............................................  0
Gi2_4175715_x3240...................................................................Uguucgauucccggcgccggagc......................  1
Gi3_15854949_x3080................................................uucucacccaacagauccg.............................................  0
Gi2_4263674_x3014.................uccggcguaguauaacggc............................................................................  0
Gi2_4266688_x3008....................................................................guucgauucccggcgccg..........................  0
Gi1_2084611_x2882................................................uucucacccaacagauccgg............................................  0
Gi3_16166426_x2766.................uccggcguaguauaacgA.............................................................................  1
Gi2_4338576_x2763...................................................................gguucgauucccggcgccgga........................  0
Gi3_16196749_x2745....................................................................guucgauucccggcgccg..........................  0
Gi5_4464014_x2733................................................uucucacccaacagauccg.............................................  0
Gi5_4466747_x2732....................................................................guucgauucccggcgccgga........................  0
Gi1_2098525_x2672....................................................................Uuucgauucccggcgccgg.........................  1
Gi1_2106457_x2553....................................................................guucgauucccggcgccggagcC.....................  1
Gi3_16419246_x2520.................uccggcguaguauaacggcuagaa.......................................................................  0
Gi1_2118934_x2435.................uccggcguaguauaacggcua..........................................................................  0
Gi5_4584884_x2359...................................................................gguucgauucccggcgccgg.........................  0
Gi2_4550526_x2301...................................................................gUuucgauucccggcgccggagc......................  1
Gi3_16719768_x2271......................................................................ucgauucccggcgccggagcC.....................  1
Gi1_2160670_x2236....................................................................guucgauucccggcgccgga........................  0
Gi1_2162906_x2212..............................................gguucucacccaacagauccg.............................................  0
Gi2_4595781_x2187.................uccggcguaguauaacggcua..........................................................................  0
Gi2_4659874_x2088....................................................................guucgauucccggcgccgga........................  0
Gi5_4689295_x2063....................................................................guucgauucccggcgccggagcC.....................  1
Gi2_4678540_x2059....................................................................guucgauucccggcgccggag.......................  0
Gi1_2196056_x1931.................uccggcguaguauaacggcuaga........................................................................  0
Gi5_4746918_x1924...................................................................gguucgauucccggcgcc...........................  0
Gi3_17173976_x1910...............................................guucucacccaacagauccg.............................................  0
Gi3_17258833_x1863................................................uucucacccaacagauccgg............................................  0
Gi5_4797320_x1809....................................................................guucgauucccggcgccggagc......................  0
Gi5_4829364_x1752...................................................................gguucgauucccggcgccggag.......................  0
Gi1_2219646_x1714...............................................guucucacccaacagauccgg............................................  0
Gi2_4889877_x1702.................uccggcguaguauaacgg.............................................................................  0
```

Gi5_4856838_x1692..cucacccaacagauccgg.. 0
Gi3_17723670_x1584...Uguucgauucccggcgccgga........................ 1
Gi2_5042785_x1544..gguucucacccaacagauccg... 0
Gi1_2263260_x1508..guucgauucccggcgccg.......................... 0
Gi2_5126744_x1455.................uccggcguaguauaacggcuag... 0
Gi5_4974773_x1454...ugguucucacccaacagauccg... 0
Gi1_2279516_x1437..gGucgauucccggcgccgg......................... 1
Gi3_18002792_x1423..ucgauucccggcgccggagc...................... 0
Gi2_5159750_x1419.................uccggcguaguauaacggcuagaau.. 0
Gi2_5190590_x1386..uucucacccaacagauccg... 0
Gi3_18114895_x1376...Uguucgauucccggcgccggag....................... 1
Gi1_2308841_x1354...ugguucucacccaacagauccg... 0
Gi3_18209951_x1340.................Gccggcguaguauaacggcuaga.. 1
Gi2_5265076_x1323..ucgauucccggcgccggagc...................... 0
Gi3_18253831_x1317..Uuucgauucccggcgccgg......................... 1
Gi2_5286105_x1300...guucucacccaacagauccg... 0
Gi1_2332554_x1264...gguucgauucccggcgccggagc...................... 0
Gi5_5082952_x1247...guucucacccaacagauccgg.. 0
Gi1_2340050_x1226..guucgauucccggcgccggagc...................... 0
Gi3_18631876_x1148..Uuucgauucccggcgccgga........................ 1
Gi5_5178308_x1083..Uuucgauucccggcgccgg......................... 1
Gi5_5191186_x1059..Uuucgauucccggcgccg.......................... 1
Gi3_18881639_x1044...gUuucgauucccggcgccgga........................ 1
Gi2_5612893_x1032..Uuucgauucccggcgccggagc...................... 1
Gi3_18985137_x1003..Uuucgauucccggcgccggagc...................... 1
Gi5_5228053_x994.................uccggcguaguauaacggcuagaa... 0
Gi2_5713753_x968.................uccggcguaguauaacggcuagaa... 0
Gi3_19108956_x964...gUuucgauucccggcgccggag....................... 1
Gi3_19131046_x956...ugguucucacccaacagauccg... 0
Gi1_2424457_x950.................uccggcguaguauaacggc.. 0
Gi5_5277356_x931...Uguucgauucccggcgccggagc...................... 1
Gi3_19223083_x923...uucgauucccggcgccggagc...................... 0
Gi3_19277081_x906..gauucccggcgccggagc...................... 0
Gi2_5857907_x868..Uuucgauucccggcgccgg......................... 1
Gi5_5341340_x840...ucucacccaacagauccg... 0
G1a_3705913_x820.................uccggcguaguauaacggcuaga.. 0
Gi2_5947887_x817...ugguucucacccaacagauccg... 0
Gi1_2480982_x805...gguucgauucccggcgccg.......................... 0
Gi3_19615623_x803...Uguucgauucccggcgccg.......................... 1
G1a_3739453_x774...gguucgauucccggcgccggagc...................... 0
Gi3_19734571_x772...guucucacccaacagauccgg.. 0
Gi2_6110175_x725...gguucgauucccggcgccgg......................... 0
Gi5_5431764_x722..Uguucucacccaacagauccg... 1
Gi5_5453834_x702..uucucacccaacagauccgg.. 0
Gi3_20044512_x691..gGucgauucccggcgccgg......................... 1
Gi3_20062420_x686..guucgauucccggcgccgC......................... 1
Gi2_6221840_x679.................uccggcguaguauaacgA... 1

```
Gi5_5504875_x652...................................................................gUuucgauucccggcgccggagc......................    1
Gi1_2562309_x634...................................................................gguucgauucccggcgccgga........................    0
Gi3_20331714_x633...................................................................Aguucgauucccggcgccggagc......................   1
Gi3_20356944_x628.......................................................................cgauucccggcgccggagc......................   0
G1a_3873470_x626.................uccggcguaguauaacggcuag.........................................................................    0
Gi3_20450237_x607.....................................................................uucgauucccggcgccgga........................   0
Gi3_20477458_x603.....................................................................uucgauucccggcgccggagcC.....................   1
Gi3_20490687_x600....................................................................gGucgauucccggcgccgga........................   1
Gi3_20592804_x574........................................................................gauucccggcgccggagcC.....................   1
Gi3_20641249_x565...................................................................gguucgauucccggcgccgC.........................   1
Gi3_20654213_x562....................................................................guucgaCucccggcgccggagc......................   1
Gi2_6524433_x562....................................................................gGucgauucccggcgccggagc......................    1
Gi3_20695535_x555.................uGcggcguaguauaacggcuaga........................................................................   1
Gi3_20711587_x552...................................................................gguucgauNcccggcgccggagc......................   1
Gi3_20742876_x547...................................................................gUuucgauucccggcgccg..........................   1
Gi5_5635437_x543....................................................................gGucgauucccggcgccgg.........................    1
Gi3_20766281_x542......................................................................ucgauucccggcgccggag.......................   0
Gi1_2621476_x538.................uccggcguaguauaacggcuagaau......................................................................    0
Gi3_20813076_x534....................................................................Uuucgauucccggcgccggag.......................   1
Gi1_2629414_x520...................................................................Uguucgauucccggcgccgg.........................    1
Gi3_20890919_x518......................................................................ucgauucccggcgccgga........................   0
Gi3_20912056_x514...................................................................Uguucgauucccggcgccgg.........................   1
Gi5_5680282_x510..............................................gUuucucacccaacagauccg.............................................    1
Gi3_20940158_x508..................................................cucacccaacagauccgg............................................   0
Gi1_2635580_x506.................uccggcguaguauaacggcu...........................................................................    0
Gi2_6718036_x497........................................................................gauucccggcgccggagc......................    0
Gi2_6721016_x496....................................................................gGucgauucccggcgccgg.........................    1
Gi3_21017851_x495.........................................................................auucccggcgccggagcC.....................   1
Gi3_21069318_x486...................................................................gguucgauucccggcgccggCgc......................   1
Gi2_6751449_x486...................................................................Uguucgauucccggcgcc...........................    1
Gi2_6769824_x481...................................................................Uguucgauucccggcgccggag.......................    1
Gi2_6779412_x478......................................................................ucgauucccggcgccggagcC.....................    1
Gi1_2664335_x471..................................................cucacccaacagauccgg............................................    0
Gi3_21175959_x470.................................................ucucacccaacagauccg.............................................   0
Gi5_5742002_x468....................................................................gGucgauucccggcgccg..........................    1
Gi5_5754997_x462...............................................Uuucucacccaacagauccg.............................................    1
Gi3_21241144_x461....................................................................gGucgauucccggcgccggagc......................   1
Gi3_21272709_x455..............................................Uguucucacccaacagauccg.............................................   1
Gi1_2680859_x446.................................................ucucacccaacagauccg.............................................    0
Gi1_2686601_x437.....................................................................uucgauucccggcgccgg.........................    0
Gi5_5802448_x432...................................................................Uguucgauucccggcgccg..........................    1
Gi3_21433943_x429...................................................................ggCucgauucccggcgccggagc......................   1
Gi5_5815709_x425.................uccggcguaguauaacggcu...........................................................................    0
Gi3_21502240_x419.................uccggcguaguauaacAgcuaga........................................................................   1
Gi2_7002965_x415...................................................................Uguucgauucccggcgccg..........................    1
Gi3_21540181_x414.................uccggcguaguauaacggcu...........................................................................   0
G1a_4132002_x412...................................................................gguucgauucccggcgccggag.......................    0
```

```
Gi3_21582156_x408................ucUggcguaguauaacggcuaga....................................................................... 1
Gi3_21603665_x404................uccggcguaguauaacggcuGga....................................................................... 1
Gi1_2728696_x387................................................ucucacccaacagauccgg........................................... 0
Gi3_21757565_x383................uccggcguaguauaacggcuagG....................................................................... 1
Gi3_21767117_x381..................................................................Uguucgauucccggcgcc.......................... 1
Gi3_21824415_x373..................................................................gUuucgauucccggcgccgg........................ 1
Gi5_5902217_x373..................................................................Uguucgauucccggcgccgga....................... 1
Gi3_21849616_x369......................................................................cgauucccggcgccggagcC.................... 1
Gi3_21850354_x369..................................................................gguucgauucccggcgccggagcCu................... 1
Gi1_2749901_x367..................................................................gguucgauucccggcgccggag...................... 0
Gi5_5915080_x362.......................................................................gauucccggcgccggagc..................... 0
Gi3_21945585_x355............................................ugguucucacccaacagauc.............................................. 0
Gi3_21982977_x351................Gccggcguaguauaacggcua......................................................................... 1
Gi1_2772066_x347..............................................Uuucucacccaacagauccg............................................ 1
Gi2_7279530_x347.......................................................................gauucccggcgccggagcC.................... 1
Gi3_22011911_x347.................ccggcguaguauaacggc........................................................................... 0
Gi3_22045248_x341..........................................................cagauccggguucgauuccc................................ 0
Gi1_2780262_x336..................................................................gUuucgauucccggcgccgg........................ 1
Gi5_5989302_x333.............................................gguucucacccaacagauccgg........................................... 0
Gi2_7348465_x332..................................................................Aguucgauucccggcgccggagc..................... 1
Gi2_7360380_x330..................................................................gUuucgauucccggcgccggag...................... 1
Gi5_6005812_x327..................................................................gUuucgauucccggcgccg......................... 1
Gi3_22176246_x324................uccggcguaguauaacggcCaga....................................................................... 1
Gi3_22209728_x320................uccggcguaguauaacggcuaA........................................................................ 1
Gi2_7428877_x314...................................................................Uuucgauucccggcgccg......................... 1
Gi3_22313956_x308..................................................................gUuucgauucccggcgcc.......................... 1
Gi3_22310260_x308..................................................................gguucgauucccggcgccggGgc..................... 1
Gi2_7451906_x308...................................................................guucgaCucccggcgccggagc..................... 1
Gi1_2813435_x304................uccggcguaguauaacgA............................................................................ 1
Gi3_22350015_x303..............................................................uccggguucgauucccggcgccgga....................... 0
Gi3_22355469_x303.............................................gUuucucacccaacagauccg............................................ 1
Gi3_22369960_x301...................................................................gGucgauucccggcgccggag...................... 1
G1a_4343709_x299..................................................................gguucgauucccggcgccggagcC.................... 1
Gi3_22406772_x297..................................................................gguucgauuccUggcgccggagc..................... 1
Gi3_22426894_x295...................................................................Uuucgauucccggcgccg......................... 1
Gi3_22440421_x293..................................................................Cguucgauucccggcgccggagc..................... 1
Gi3_22470199_x290....................................................................uucgauucccggcgccgg........................ 0
Gi1_2833307_x289.................................................Uucacccaacagauccgg........................................... 1
Gi1_2837629_x286..................................................................gguucgauucccggcgccggagcC.................... 1
Gi2_7584647_x283..................................................................gUuucgauucccggcgcc.......................... 1
Gi3_22550896_x282................Gccggcguaguauaacgg............................................................................ 1
Gi2_7604625_x280....................................................................uucgauucccggcgccgg........................ 0
Gi2_7623265_x277..................................................................Uguucgauucccggcgccgga....................... 1
Gi3_22591439_x277................Gccggcguaguauaacggc........................................................................... 1
Gi2_7633467_x275..................................................................gguucgauNcccggcgccggagc..................... 1
Gi3_22625362_x274................uccggcguNguauaacggcuaga....................................................................... 1
Gi3_22667789_x270...................................................................guucgauucccggcgccggagcCu................... 1
```

```
Gi1_2865516_x267....................................................................guucgauucccggcgccggagcCu....................	1
Gi5_6179994_x266....................................................................Uuucgauucccggcgccgga........................	1
Gi3_22720450_x265...................................................................gguucgaCucccggcgccggagc......................	1
Gi3_22715945_x265............................................................agauccggguucgauuccc.................................	0
G1a_4422156_x265...................................................................gguucgauucccggcgccgga........................	0
Gi2_7692801_x265...................................................................gUuucgauucccggcgccg..........................	1
Gi1_2868710_x265......................................................................ucgauucccggcgccggagc......................	0
Gi2_7696235_x264.................Gccggcguaguauaacggcuaga........................................................................	1
Gi1_2876324_x260....................................................................guucgaCucccggcgccgg.........................	1
Gi5_6222628_x254............................................................agauccggguucgauuccc.................................	0
Gi5_6228460_x253......................................................cccaacagauccggguuc........................................	0
Gi1_2891471_x253..............................................Uguucucacccaacagauccg.............................................	1
Gi5_6235510_x251.................................................ucucacccaacagauccgg............................................	0
Gi3_22899164_x248....................................................................guucgaCucccggcgccgga........................	1
Gi5_6245734_x248...................................................................Uguucgauucccggcgcc...........................	1
Gi1_2903961_x247.............................................uUguucucacccaacagauccgg............................................	1
Gi5_6257064_x245..................ccggcguaguauaacggcuag.........................................................................	0
Gi4_339684_x242...................................................................gguucgauucccggcgccgg.........................	0
Gi3_22994850_x240...................................................................gguucgGuucccggcgccggagc......................	1
Gi1_2928825_x236.....................................................................uucgauucccggcgccggagcC.....................	1
Gi3_23052063_x235....................................................cacccaacagauccggguuc........................................	0
Gi3_23081319_x233.................uccggcguaguauaacgAcuaga........................................................................	1
Gi5_6308565_x232...................................................................Uguucgauucccggcgccgg.........................	1
Gi5_6317809_x230......................................................................ucgauucccggcgccggag.......................	0
Gi2_7899826_x230.....................................................................uucgauucccggcgccggagcC.....................	1
Gi1_2938596_x230...................................................................Cguucgauucccggcgccgg.........................	1
Gi5_6326282_x228......................................................................ucgauucccggcgccggagc......................	0
Gi5_6331966_x226....................................................................guucgauucccggcgccggag.......................	0
Gi3_23176138_x225.................uUcggcguaguauaacggcuaga........................................................................	1
Gi2_7938475_x225...................................................................gguucgauucccggcgccggCgc......................	1
Gi3_23193875_x224...................................................................gguucgauuccUggcgccggag.......................	1
Gi3_23200799_x223....................................................................guucgaCucccggcgccgg.........................	1
Gi5_6359700_x221..................ccggcguaguauaacggcua..........................................................................	0
G1a_4538766_x220................................................uucucacccaacagauccg.............................................	0
Gi1_2963766_x219....................................................................gCucgauucccggcgccgg.........................	1
G1a_4546649_x218...................................................................gguucgauucccggcgccgg.........................	0
G1a_4549477_x217...............................................guucucacccaacagauccg.............................................	0
Gi2_7994399_x217....................................................................Uuucgauucccggcgccggag.......................	1
Gi1_2970519_x216.................uccggcguaguauaacggcuagaa.......................................................................	0
Gi3_23309690_x215.................uccggcguaguauaGcggcuaga........................................................................	1
Gi3_23304530_x215.................................................ucucacccaacagauccgg............................................	0
Gi2_8017908_x214.....................................................................uucgauucccggcgccggagc......................	0
G1a_4557874_x214...................................................................gguucgauucccggcgccg..........................	0
Gi5_6398702_x214...................................................................gUuucgauucccggcgccgga........................	1
Gi5_6408310_x213.................Gccggcguaguauaacggcuag.........................................................................	1
Gi5_6417005_x211......................................................................ucgauucccggcgccggagcC.....................	1
Gi3_23359579_x211..................ccggcguaguauaacggcuaga........................................................................	0
```

```
Gi1_2985636_x210.....................................................................Uuucgauucccggcgccgga........................   1
Gi3_23382327_x210.....................................................................guucgauucccggcgccgA.........................   1
Gi3_23458130_x204.................uccggcguGguauaacggcuaga.........................................................................   1
Gi5_6461919_x203....................................................................Uguucgauucccggcgccggag.......................   1
Gi5_6465567_x202..............................................ugguucucacccaacagauccgggu..........................................   0
Gi1_3010075_x201......................................................................uucgauucccggcgccggagc......................   0
Gi3_23506713_x201.........................................................caacagauccggguucgauuccc.................................   0
Gi2_8127732_x200....................................................................ggCucgauucccggcgccggagc......................   1
Gi3_23516931_x200....................................................................gguCcgauucccggcgccggagc......................   1
Gi2_8144076_x198.....................................................................Uuucgauucccggcgccgga........................   1
Gi3_23560200_x197..............................................................gauccggguucgauuccc.................................   0
Gi3_23573973_x196....................................................................gCuucgauucccggcgccggagc......................   1
Gi3_23572013_x196.................uccggcguaguGuaacggcuaga.........................................................................   1
Gi5_6501422_x196...............................................gguucucacccaacagauc...............................................   0
Gi3_23604809_x194.................uGcggcguaguauaacggcua...........................................................................   1
Gi5_6522813_x193.................Gccggcguaguauaacggcuaga.........................................................................   1
Gi3_23634928_x192...............................................gguucucacccaacagaucc..............................................   0
Gi5_6534139_x191................................................gGucucacccaacagauccg.............................................   1
G1a_4632800_x191.....................................................................guucgauucccggcgccggagcC.....................   1
Gi3_23641643_x191.....................................................................guucgauucccggUgccggag.......................   1
Gi3_23661655_x190................................................Uuucucacccaacagauccg.............................................   1
Gi2_8225747_x189...............................................Uguucucacccaacagauccg.............................................   1
Gi3_23694905_x188...................................................................Agguucgauucccggcgccggagc......................   1
Gi3_23697725_x188....................................................................ggGucgauucccggcgccggagc......................   1
G1a_4644493_x187.....................................................................guucgauucccggcgccggagc......................   0
Gi5_6557180_x187....................................................ucacccaacagauccggguu.........................................   0
Gi3_23707837_x187.................Cccggcguaguauaacggcuaga.........................................................................   1
Gi3_23739640_x185.................uccggcguaguauaacggA.............................................................................   1
Gi2_8259226_x185.......................................................cccaacagauccggguuc........................................   0
Gi3_23749033_x184....................................................................gAuucgauucccggcgccggagc......................   1
G1a_4658536_x183....................................................................gguucgauucccggcgcc...........................   0
Gi1_3057106_x183...............................................gguucucacccaacagauccgg............................................   0
Gi4_367620_x179.....................................................................guucgauucccggcgccgg.........................   0
Gi1_3070143_x179.............................................................agauccggguucgauuccc.................................   0
Gi5_6615364_x179.....................................................................Uuucgauucccggcgccggagc......................   1
Gi5_6612679_x179....................ggcguaguauaacggcuag..........................................................................   0
Gi1_3071396_x179...............................................gUuucucacccaacagauccg.............................................   1
Gi3_23840974_x178.............................................uugguucucacccaacagaucc..............................................   0
Gi5_6632435_x177....................................................................gguucgauucccggcgccggagcCu....................   1
Gi3_23865777_x176........................................................................cgauucccggcgccggag.......................   0
Gi5_6646480_x175..............................................ugguucucacccaacagauccgg............................................   0
Gi1_3086057_x175.....................................................................Uuucgauucccggcgccg..........................   1
Gi1_3085182_x175................................................Uuucucacccaacagauccgg............................................   1
Gi3_23894898_x174...............................................gguucucacccaacagauccgggu..........................................   0
Gi1_3095764_x172..............................................Ggguucucacccaacagauccgg............................................   1
Gi3_23948305_x171.....................................................................guucgaCucccggcgccggag.......................   1
Gi2_8390578_x170....................................................................gUuucgauucccggcgccgga........................   1
```

```
Gi3_23962653_x170.................ucUggcguaguauaacggcua...........................................................................   1
Gi5_6685354_x169....................................................................gUuucgauucccggcgccgg.........................   1
Gi1_3103256_x169....................................................ucacccaacagauccgggu..........................................   0
Gi3_24013811_x167.................uccgUcguaguauaacggcuaga.........................................................................   1
Gi2_8419886_x166.....................................................................gGucgauucccggcgccg..........................   1
Gi3_24044896_x165.................uccggcguaguauaacggcuaAa.........................................................................   1
Gi2_8436782_x165....................................................................gguucgauucccggcgccggGgc......................   1
Gi5_6719756_x164.........................................................................gauucccggcgccggagcC.....................   1
Gi3_24072831_x163....................................................................gguucgauucccggUgccggagc......................   1
G1a_4721617_x163.....................................................................guucgauucccggcgccgg.........................   0
Gi5_6738710_x162.....................................................................guucgaCucccggcgccgg.........................   1
Gi3_24090728_x162..................................................................cggguucgauucccggcgccgga........................   0
Gi3_24111706_x161.....................................................................gGucgauucccggcgccg..........................   1
Gi2_8472510_x161.......................................................................ucgauucccggcgccggag.......................   0
Gi3_24113960_x161....................................................................gguucgauCcccggcgccggagc......................   1
Gi5_6747445_x161.................................................uucucacccaacagauccggguu.........................................   0
Gi5_6756601_x160......................................................................uucgauucccggcgccgg.........................   0
Gi5_6755161_x160................................................Uuucucacccaacagauccgg............................................   1
Gi3_24132052_x160...............................................gguucucacccaacagauccgg............................................   0
Gi1_3143858_x159................................................gGucucacccaacagauccg.............................................   1
Gi3_24150185_x159....................................................ucacccaacagauccggguuc........................................   0
Gi3_24143030_x159.................uccggcguaguauaaUggcuaga.........................................................................   1
Gi3_24142553_x159....................................................................gguucgauucccAgcgccggagc......................   1
Gi5_6778507_x158................................................................uccggguucgauucccggc..............................   0
Gi2_8512592_x157....................................................................gguucgaCucccggcgccggagc......................   1
G1a_4740628_x157..................................................ucucacccaacagauccg.............................................   0
Gi2_8518708_x156........................................................................cgauucccggcgccggagcC.....................   1
Gi1_3155014_x156................................................guucucacccaacaCauccgg............................................   1
Gi1_3162430_x154....................................................................gguucgauucccggcgccggagcCu....................   1
Gi5_6811730_x153.....................................................................guucgauucccggcgccggaCc......................   1
Gi3_24232226_x153.................uccggcguagCauaacggcuaga.........................................................................   1
Gi1_3167020_x152.....................................................................Auucgauucccggcgccgg.........................   1
Gi3_24258258_x151.................uccggUguaguauaacggc.............................................................................   1
Gi3_24263996_x151....................................................................gguucgauucccggcgccggagcA.....................   1
Gi3_24257654_x151....................................................................Aguucgauucccggcgccggag.......................   1
Gi2_8579332_x150.............................................................agauccggguucgauucc..................................   0
Gi3_24312302_x148....................................................................gguucgauucccggcgAcggagc......................   1
Gi2_8603634_x148......................................agaaugguugguucucacc........................................................   0
Gi3_24331334_x147...............................................................auccggguucgauucccggcgccgga........................   0
Gi1_3193841_x145.....................................................................guucgauuNccggcgccgg.........................   1
Gi2_8651444_x144................................................Uuucucacccaacagauccg.............................................   1
Gi1_3205093_x143..................ccggcguaguauaacggcua...........................................................................   0
Gi3_24417877_x142.....................................................................guucgauucccggcgccggaC.......................   1
Gi3_24415889_x142....................................................................gguucAauucccggcgccggagc......................   1
Gi2_8670143_x142....................................................................Cguucgauucccggcgccggagc......................   1
Gi1_3216716_x141.................Gccggcguaguauaacgg..............................................................................   1
Gi2_8681736_x141..............................................ugguucucacccaacagaucc..............................................   0
```

```
G1a_4799734_x141.................uccggcguaguauaacgg.............................................................................   0
Gi3_24449273_x140...............................................guucucacccaacagauccggg...........................................   0
Gi5_6930592_x139.........................................................................auucccggcgccggagcC.....................   1
Gi2_8698787_x139.................uccggcguaguauaacggcuaAaa.......................................................................   1
Gi5_6938237_x139....................................................................guucgauucccggcgccggagcCu....................   1
Gi3_24512108_x136...................................................................ggAucgauucccggcgccggagc......................   1
Gi2_8736673_x136....................................................................gGucgauucccggcgccgga........................   1
Gi2_8744776_x135....................................................................guuUgauucccggcgccggag.......................   1
Gi3_24559584_x134................................................uucucacccaacagauccggguuc........................................   0
Gi5_6979068_x134...................................................................gguucgauuUccggcgccgg.........................   1
Gi3_24551410_x134..................................................................ggguucgauucccggcgccggagc......................   0
Gi1_3244829_x134....................................................................Uuucgauucccggcgccggagc......................   1
Gi5_6975584_x134...................................................................gUuucgauucccggcgccggag.......................   1
Gi1_3250029_x133..................ccggcguaguauaacggcuaga........................................................................   0
Gi3_24613453_x131...................................................................gguuUgauucccggcgccggagc......................   1
Gi5_7003636_x131....................................................................gGucgauucccggcgccgga........................   1
Gi1_3258716_x131..................................................cucacccaacagauccCg............................................   1
Gi5_7008202_x130...................................................................gUuucgauucccggcgcc...........................   1
G1a_4841853_x129.................uccggcguaguauaacggc............................................................................   0
Gi3_24642742_x129.................uccggcguaguauCacggcuaga........................................................................   1
Gi1_3271991_x127....................................................................guCcgauucccggcgccgg.........................   1
Gi5_7036657_x127....................................................................guucgaCucccggcgccg..........................   1
Gi3_24687352_x126...................................................................Aguucgauucccggcgccgga........................   1
Gi3_24694660_x126...................................................................gguucgauucccggcgccggagA......................   1
Gi5_7068052_x124..................ccggcguaguauaacggcuaga........................................................................   0
Gi3_24739411_x123...................................................................gguucgauNcccggcgccgga........................   1
Gi3_24759981_x122.................uGcggcguaguauaacggc............................................................................   1
Gi3_24749611_x122...................................................................gguucgauucccggcgcUggagc......................   1
Gi2_8905500_x121...................................................................gguCcgauucccggcgccggagc......................   1
Gi5_7102542_x121............................................uugguucucacccaacagauccg.............................................   0
Gi1_3298259_x121..................ccggcguaguauaacggc............................................................................   0
Gi5_7117155_x119.................Gccggcguaguauaacgg.............................................................................   1
Gi3_24812582_x119........................................................caacagauccggguucga......................................   0
Gi1_3311683_x118...................................................................Uguucgauucccggcgccggagc......................   1
Gi3_24828166_x118...............................................guucucacccaacagauccgggu..........................................   0
Gi4_406299_x117.................uccggcguaguauaacggcuaga........................................................................   0
Gi2_8951030_x117...................................................................Uguucgauucccggcgccgg.........................   1
Gi3_24835557_x117...................................................................gguucgauNcccggcgccggag.......................   1
Gi5_7131131_x117...................................................................Cguucgauucccggcgcc...........................   1
Gi3_24857697_x116.................uccggUguaguauaacggcuaga........................................................................   1
Gi3_24876850_x115.................uccggcguGguauaacggcua..........................................................................   1
Gi1_3326847_x115................................................Gucucacccaacagauccg.............................................   1
Gi5_7166130_x114.................Gccggcguaguauaacggc............................................................................   1
Gi1_3331080_x114.................Gccggcguaguauaacggcuag.........................................................................   1
Gi3_24942169_x111...................................................................gguucgauucccggcgccggaCc......................   1
Gi2_9037156_x110......................................................................ucgauucccggcgccgga........................   0
Gi5_7202873_x110.............................................uUguucucacccaacagauccg.............................................   1
```

```
Gi3_24965152_x110.................uGcggcguaguauaacgg.............................................................................   1
Gi5_7222001_x108.................Gccggcguaguauaacggcuagaau......................................................................   1
Gi5_7223837_x108................................................Gucucacccaacagauccg.............................................   1
Gi3_24998583_x108.................uccggcguaguauaacggUuaga........................................................................   1
Gi3_24991239_x108.................Gccggcguaguauaacggcuagaau......................................................................   1
Gi1_3358450_x107................................................Gucucacccaacagauccgg............................................   1
Gi3_25019293_x107....................................................................guucgaCucccggcgccg..........................   1
Gi3_25009663_x107........................................................................Uauucccggcgccggagc......................   1
Gi5_7233480_x107.................uGcggcguaguauaacggcuaga........................................................................   1
Gi3_25015334_x107..................................................................ggguucgauucccggcgccggag.......................   0
Gi2_9076486_x106...............aguccggcguaguauaacgg.............................................................................   0
Gi4_415438_x105...................................................................gguucgauucccggcgccggagc......................   0
G1a_4940392_x105.....................................................................uucgauucccggcgccggagc......................   0
Gi5_7247474_x105.................................................ucucacccaacagauccgggu..........................................   0
Gi1_3372775_x105....................................................................gGucgauucccggcgccgga........................   1
Gi2_9109341_x104....................................................................gGucgauucccggcgccggag.......................   1
Gi5_7261905_x104.................Gccggcguaguauaacggcua..........................................................................   1
Gi3_25095319_x103..................ccggcguaguauaacggcuag.........................................................................   0
Gi3_25091096_x103.................Gccggcguaguauaacggcuag.........................................................................   1
Gi2_9115834_x103...................................................................gguucgGuucccggcgccggagc......................   1
Gi3_25104011_x102...................................................................gguAcgauucccggcgccggagc......................   1
Gi3_25119990_x101...................................................................gguucgCuucccggcgccggagc......................   1
Gi5_7296674_x101...................................................................gguucgauucccggcgccggagcgu....................   0
Gi2_9144974_x100.......................................................................cgauucccggcgccggagcguuA..................   1
Gi3_25149226_x100.................uccggcguaguauaacggcuagC........................................................................   1
Gi2_9150774_x100.................uGcggcguaguauaacggcuaga........................................................................   1
Gi3_25155666_x99................................................Gucucacccaacagauccg.............................................   1
Gi1_3403571_x98...................................................................gUuucgauucccggcgccggagc......................   1
Gi3_25180281_x98....................ggcguaguauaacggcuaga........................................................................   0
Gi1_3407858_x97.............................................uUguucucacccaacagauccg.............................................   1
Gi3_25193808_x97................................................uGcucacccaacagauccg.............................................   1
Gi1_3410380_x97...................................................................gguucgauucccggcgccggagcU.....................   1
Gi2_9186957_x97....................................................................guucgaCucccggcgccgg.........................   1
Gi1_3412405_x96...................................................................gguucgauucccggcgcc...........................   0
Gi1_3412885_x96...................................................................gguucgauucccggcgccAg.........................   1
Gi3_25219584_x95.................uccggcguaguaCaacggcuaga........................................................................   1
Gi5_7369994_x94....................................................................gCucgauucccggcgccg..........................   1
Gi3_25254537_x94...................................................................gguucgauucUcggcgccggagc......................   1
Gi3_25246829_x94............................................................agauccggguucgauucc..................................   0
Gi1_3431858_x93....................................................................Cuucgauucccggcgccgg.........................   1
Gi3_25280139_x92...............................................gGucucacccaacagauccg.............................................   1
Gi3_25304938_x91...................................................................gguucgauucccggcgUcggagc......................   1
Gi3_25293472_x91....................................................................Auucgauucccggcgccgga........................   1
Gi1_3443389_x90..................................................................Agguucgauucccggcgccgg.........................   1
Gi3_25328468_x89...............................................Uuucucacccaacagauccgg............................................   1
Gi5_7424212_x89..........................................................acagauccggguucgauucccggcgc............................   0
Gi2_9277568_x89...................................................................gAuucgauucccggcgccggagc......................   1
```

```
Gi1_3455056_x88...................................................................guucgGuucccggcgccgg.........................     1
Gi3_25349424_x88................uccggcguaguauaacggcuagaaA......................................................................     1
Gi5_7436018_x88...................................................................gGucgauucccggcgccggagc......................     1
Gi1_3460306_x87...................ggcguaguauaacggcuag.........................................................................     0
Gi5_7437946_x87................uGcggcguaguauaacggcuag.........................................................................     1
Gi1_3459958_x87..............................................gGucucacccaacagauccgg............................................     1
Gi2_9296624_x87..................................................................ggGucgauucccggcgccggagc......................     1
Gi3_25380784_x86................uccggcgCaguauaacggcuaga........................................................................     1
Gi1_3464465_x86..................................................................Uguucgauucccggcgccg..........................     1
Gi2_9316258_x86..................cggcguaguauaacggcua..........................................................................     0
Gi3_25413852_x85.....................................................cccaacagauccggguucgauuccc.................................     0
Gi3_25408497_x85................uccggcguaguauaacggcuagaauA.....................................................................     1
Gi2_9323159_x85................Gccggcguaguauaacggc............................................................................     1
G1a_5035827_x85................uccggcguaguauaacggcua..........................................................................     0
Gi3_25425115_x84..........................................................cagauccggguucgauucccggcgccgg.........................     0
Gi3_25418899_x84...................................................................Auucgauucccggcgccggagc......................     1
Gi3_25424863_x84..................................................................gguucgauucccggcgcAggagc......................     1
Gi3_25423855_x84..................................................................gguucgauucccggcAccggagc......................     1
Gi4_435328_x83...........................................uugguucucacccaacaga.................................................     0
Gi2_9348290_x83.................................................cucacccaacagauccgg............................................     0
G1a_5050925_x83..................................................................Uguucgauucccggcgccggagc......................     1
Gi3_25442693_x83................Gccggcguaguauaacggcuagaa.......................................................................     1
Gi5_7492827_x83..................................................................Aguucgauucccggcgccggagc......................     1
Gi1_3484149_x82...................................................................guucAauucccggcgccgg.........................     1
Gi5_7506722_x82..................................................................gguucgauucccggcgccggagcguu...................     0
Gi1_3491081_x81...................................................................guucgauucccggcgccAg.........................     1
Gi3_25469201_x81..................................................................gguucgauucccUgcgccggagc......................     1
Gi3_25483376_x81..................................................................gguucgauucccggcgccAgagc......................     1
Gi3_25483214_x81..................................................................gguucgauucccggcgccggG........................     1
Gi3_25500696_x80................ucUggcguaguauaacggc............................................................................     1
Gi2_9378699_x80....................................................................uucgauucccggcgccggag.......................     0
Gi3_25486936_x80..................................................................ggCucgauucccggcgccgga........................     1
Gi2_9385499_x80.............................................gUuucucacccaacagauccg.............................................     1
Gi3_25504765_x79...................................................................guucgauucccggcgccggGg.......................     1
Gi3_25536986_x78............................................uUguucucacccaacagauccg.............................................     1
Gi1_3506059_x78...............................................uGcucacccaacagauccg.............................................     1
Gi1_3501457_x78...................................................................guuUgauucccggcgccgg.........................     1
Gi1_3501301_x78................uccggcguaguauaacggcuagaauU.....................................................................     1
Gi1_3510223_x77...................................................................gGucgauucccggcgccg..........................     1
Gi5_7559961_x77......................................................................cgauucccggcgccggagcguuuc.................     0
Gi4_442433_x77...................................................................guucgauucccggcgccgga........................     0
Gi1_3506604_x77...................................................................guucUauucccggcgccgg.........................     1
Gi2_9414179_x77...................................................................Auucgauucccggcgccgg.........................     1
Gi2_9417028_x77..................................................................ggAucgauucccggcgccggagc......................     1
Gi3_25563010_x76.............................................Cguucucacccaacagauccg.............................................     1
Gi2_9423957_x76................uccggcguaguauaacggcu...........................................................................     0
Gi1_3511686_x76...............................................uGcucacccaacagauccgg............................................     1
```

Gi3_25568178_x76...gguucgauucccggcgccggagU...................... 1
Gi3_25594935_x75..guucgauuNccggcgccgga........................ 1
Gi2_9443182_x75...gguuUgauucccggcgccggagc...................... 1
Gi2_9445432_x75...................cggcguaguauaacggcuaga.. 0
Gi5_7589840_x74..guucgaCucccggcgccggagc...................... 1
Gi5_7588434_x74...................cggcguaguauaacggcuaga.. 0
Gi3_25627916_x73.................uccggcguUguauaacggcuaga.. 1
Gi3_25620397_x73..guucgauuNccggcgccgg......................... 1
Gi2_9465654_x73.................uccggcguaguauaacggcuGga.. 1
Gi5_7599143_x73.................uccggcguaguauaacggcuagG.. 1
Gi3_25621492_x73..Auucgauucccggcgccgg......................... 1
Gi3_25652418_x72...gguucgauuUccggcgccggagc...................... 1
Gi5_7616829_x72..uucucacccaacagauccggguuc.. 0
Gi3_25641906_x72.................ucUggcguaguauaacgg... 1
G1a_5103639_x72.................uccggcguaguauaacggcuagaa... 0
Gi5_7618053_x72....................ggcguaguauaacggcuaga.. 0
Gi3_25636938_x72....................ggcguaguauaacggcuag... 0
Gi3_25635066_x72...ugguucucacccaacagauccgg.. 0
Gi2_9477623_x72...cgauucccggcgccggagcguuuc................. 0
Gi3_25652994_x72.................uccggcguNguauaacggcua.. 1
Gi3_25661387_x71...Cguucgauucccggcgccgga........................ 1
Gi1_3536906_x71.................uccggcguaguauaacggcuagaU... 1
Gi2_9490621_x71...gCuucgauucccggcgccggagc...................... 1
Gi1_3536125_x71...Uguucgauucccggcgccgga........................ 1
Gi1_3538184_x71..gGucgauucccggcgccggagc...................... 1
Gi1_3539746_x71..guucgauucccggUgccgg......................... 1
Gi3_25659541_x71..ggguucgauucccggcgccgga........................ 0
G1a_5110245_x71...cgauucccggcgccggagcC..................... 1
Gi4_448843_x70..cucacccaacagauccggguucg....................................... 0
Gi2_9500831_x70...guucucacccaacagauccgg.. 0
Gi1_3542696_x70....................ggcguaguauaacggcua.. 0
Gi3_25690730_x70..guucgGuucccggcgccgga........................ 1
Gi2_9499641_x70..uucucacccaacagauccgg.. 0
Gi2_9498941_x70...gguucgauucccggcgAcggagc...................... 1
Gi5_7641508_x70..gguucucacccaacagauccggguu... 0
G1a_5117872_x70..guucgauucccggcgccg.......................... 0
Gi3_25682750_x70.................uccggcguCguauaacggcuaga.. 1
G1a_5115352_x70.................uccggcguaguauaacggcuagaau.. 0
Gi5_7642558_x70.................uccggcguaguauaacggcuGga.. 1
Gi1_3541786_x70.................Gccggcguaguauaacggcua.. 1
Gi5_7651960_x69..Aguucucacccaacagauccg... 1
Gi2_9507881_x69...gguucgauucccggcgccggagA...................... 1
Gi2_9515471_x69.................ucUggcguaguauaacggcuaga.. 1
Gi3_25693495_x69..gCucgauucccggcgccgg......................... 1
Gi5_7645819_x69.................uGcggcguaguauaacggcuagaau.. 1
Gi3_25706398_x69..guucgauucccggcgccggG........................ 1
Gi2_9511814_x69...gUuucgauucccggcgccgg......................... 1

```
Gi1_3549294_x69.................Gccggcguaguauaacggcuaga........................................................................ 1
Gi3_25697014_x69...................................................................ggCucgauucccggcgccggag....................... 1
Gi3_25713635_x68.................uccggcguaguauaacggcuUga........................................................................ 1
Gi5_7667663_x68....................................................................Auucgauucccggcgccg.......................... 1
Gi5_7659095_x68...................................................................gguucgauNcccggcgccggagc...................... 1
Gi2_9519724_x68...................................................................gguucgauucccAgcgccggagc...................... 1
Gi5_7664807_x68.............................................Ggguucucacccaacagauccg............................................. 1
Gi3_25725127_x68...................................................................Aguucgauucccggcgccg.......................... 1
Gi2_9536503_x67...................................................................gguucgauCcccggcgccggagc...................... 1
Gi5_7668269_x67...................................................................gguucgauucccggcgccAg......................... 1
Gi3_25740233_x67.................Accggcguaguauaacggcuaga........................................................................ 1
Gi2_9542198_x67..................ccggcguaguauaacggcua.......................................................................... 0
Gi5_7691722_x66...........................................................cagauccggguucgauucccggcgccg.......................... 0
Gi1_3559898_x66....................................................................guucgauucccAgcgccgg......................... 1
Gi2_9546257_x66...................................................................gguucAauucccggcgccggagc...................... 1
Gi1_3561746_x66....................................................................guucgauCcccggcgccgg......................... 1
Gi5_7680238_x66..................................................................ggguucgauucccggcgccggagc...................... 0
Gi5_7687168_x66......................................................................ucgauucccggcgccgga........................ 0
Gi2_9554639_x66.................Gccggcguaguauaacggcua.......................................................................... 1
Gi1_3561548_x66.............................................Ggguucucacccaacagauccg............................................. 1
Gi3_25773912_x65.................uccAgcguaguauaacggcuaga........................................................................ 1
Gi2_9562796_x65...............................................gGucucacccaacagauccg............................................. 1
Gi3_25769037_x65....................................................................gCucgauucccggcgccggagc...................... 1
Gi5_7698366_x65................................................................ccggguucgauucccggcgccgga........................ 0
Gi3_25791259_x64...................................................................Cguucgauucccggcgccggag....................... 1
Gi5_7709861_x64...................cggcguaguauaacggcuag......................................................................... 0
Gi1_3572008_x64........................................................caacagauccggguucgauucccggc.............................. 0
Gi2_9578297_x64.......................................................................cgauucccggcgccggagcguuu.................. 0
Gi2_9577273_x64....................................................................Auucgauucccggcgccggagc...................... 1
Gi2_9571449_x64...............aguccggcguaguauaacggcuaga........................................................................ 0
Gi2_9571513_x64................................................uucucacccaacagauccggguucg....................................... 0
Gi1_3573800_x64....................................................................guucgauucccggcgcUgg......................... 1
Gi5_7726656_x63....................................................................Auucgauucccggcgccgg......................... 1
Gi3_25805856_x63...................................................................gguucgauucccggcgccggagcgA.................... 1
Gi1_3575068_x63...................................................................gUuucgauucccggcgccg.......................... 1
Gi2_9582921_x63....................................................................guucgaCucccggcgccg.......................... 1
Gi5_7721679_x63...................................................................gguucgauucccggcgccggagcguuu.................. 0
Gi3_25815684_x63....................ggcguaguauaacggcua.......................................................................... 0
Gi3_25813101_x63.................uccggcguaguauaCcggcuaga........................................................................ 1
Gi3_25813920_x63....................................................................guucgauucccggcgccggCgc...................... 1
Gi3_25833619_x62...................................................................gguucgauucccggcgccgAagc...................... 1
Gi3_25833123_x62...................................................................gguucgauucccggUgcc........................... 1
Gi2_9593361_x62...................................................................gguucgauucccggcgccggGg....................... 1
Gi3_25836223_x62...................................................................gguucgauucccggcgAc........................... 1
Gi3_25826303_x62....................................................................guucgGuucccggcgccggagc...................... 1
Gi3_25822955_x62.................uccggcguGguauaacgg............................................................................. 1
Gi3_25830829_x62.................uccggcguaguauaacggcuCga........................................................................ 1
```

G1a_5165112_x62..uucucacccaacagauccgg.. 0
Gi5_7745403_x61....................ggcguaguauaacggcuagaau.. 0
Gi1_3588651_x61...cagauccggguucgauucccggcgcc........................... 0
Gi5_7741133_x61...................cggcguaguauaacggcuagaau.. 0
Gi1_3588041_x61..guucgauuccUggcgccgg......................... 1
Gi1_3585662_x61..cacccaacagauccggguucgauuccc................................. 0
Gi3_25847533_x61...gguucgauucccggcgccggGg....................... 1
Gi5_7750771_x61...gGucucacccaacagauccgg.. 1
Gi3_25845764_x61.................uccggcguaguauaacggcCa.. 1
Gi5_7750649_x61..ggCucucacccaacagauccg... 1
Gi2_9613011_x61.................uccggcguaguauaacggcuagG.. 1
Gi3_25842104_x61.................uccggcguaguauaGcggcua.. 1
Gi2_9614597_x61...gguucgauuccUggcgccggagc...................... 1
Gi3_25870549_x60...gguucgauuccAggcgccggagc...................... 1
Gi5_7764076_x60..uGcucacccaacagauccg... 1
Gi5_7760416_x60.................ucUggcguaguauaacggcuaga.. 1
Gi3_25867609_x60...gguucgauNcccggcgccg.......................... 1
Gi2_9637625_x59.................Gccggcguaguauaacgg... 1
Gi5_7768413_x59...cgauucccggcgccggagcguuuA................. 1
Gi2_9636740_x59..guucgauucccggcgccggagcCu.................... 1
Gi3_25881254_x59.................uccggcguGguauaacggc.. 1
Gi2_9631725_x59.................uccggcguNguauaacggcuaga.. 1
Gi3_25895650_x59...................cggcguaguauaacggcuaga.. 0
Gi2_9646919_x58...Aguucgauucccggcgccggag....................... 1
Gi3_25900812_x58..Gucucacccaacagauccgg.. 1
Gi5_7786211_x58.................uccggcguNguauaacggcuag... 1
Gi3_25908468_x58.................uccgAcguaguauaacggcuaga.. 1
Gi3_25913050_x58..gCucgauucccggcgccgga........................ 1
Gi5_7785515_x58...gguucgauucccggcgccggCgc...................... 1
Gi5_7790155_x58.................ucUggcguaguauaacggcuag... 1
Gi2_9663188_x57..guucgauuNccggcgccggagc...................... 1
Gi1_3611657_x57.................uGcggcguaguauaacgg... 1
Gi2_9655550_x57...uUguucucacccaacagauccg... 1
Gi3_25948550_x56..guCcgauucccggcgccgg......................... 1
Gi2_9670070_x56...uucgauucccggcgccgga........................ 0
Gi2_9670686_x56..gCucgauucccggcgccggagc...................... 1
Gi2_9671918_x56..Uauucccggcgccggagc...................... 1
Gi3_25954822_x56...gguucgaCucccggcgccgga........................ 1
Gi3_25942054_x56...gguucgauucccggcgccggaAc...................... 1
Gi3_25956726_x56...Aguucgauucccggcgccgg......................... 1
Gi3_25940150_x56..guucgauucccggcgccggagcA..................... 1
Gi5_7823094_x55.................uGcggcguaguauaacgg... 1
Gi3_25969945_x55...gguucgauucccggcgcUggag....................... 1
Gi5_7824359_x55..gCucgauucccggcgccgg......................... 1
Gi1_3622888_x55.................uGcggcguaguauaacggcuag... 1
Gi3_25976215_x55.................uccggcguNguauaacgg... 1
Gi1_3627210_x54.................uccggcguGguauaacggcuag... 1

Gi2_9702216_x54...gguucgauucccggcAccggagc...................... 1
Gi2_9697464_x54..Gucucacccaacagauccg... 1
Gi3_25983306_x54...cUauucccggcgccggagc...................... 1
Gi3_26015306_x53................Cuccggcguaguauaacggcua.. 1
Gi3_26000095_x53...................cggcguaguauaacggcuagaau.. 0
Gi3_25997127_x53.................uccggcguaguGuaacggcua.. 1
Gi3_26016048_x53...gguucgauucccggAgccggagc...................... 1
Gi3_26031092_x52..Gcgauucccggcgccggagc...................... 1
Gi1_3633854_x52..guucgauucccggcAccgg......................... 1
Gi3_26032808_x52...cgauucccggcgccggagcguuucA................ 1
Gi3_26033432_x52..Cuucgauucccggcgccgga........................ 1
Gi3_26030988_x52...Ggguucucacccaacagauccg... 1
Gi5_7864261_x51...ggCucgauucccggcgccggagc...................... 1
Gi5_7872115_x51...guCcucacccaacagauccg... 1
Gi5_7874053_x51...gguucgauucccggcgccggagcA..................... 1
Gi1_3640695_x51..guucgauucccggcgcAgg......................... 1
Gi3_26052100_x51...gguucgauucccgAcgccggagc...................... 1
Gi3_26049448_x51..Cuucgauucccggcgccgg......................... 1
Gi1_3642664_x50..guucgauucUcggcgccgg......................... 1
Gi3_26067836_x50...ugguucucacccaUcagauc... 1
Gi2_9747145_x50...gguucgauucccggcgcAggagc...................... 1
Gi3_26069236_x50...Cguucgauucccggcgccg.......................... 1
Gi3_26057686_x50...gguuGgauucccggcgccggagc...................... 1
Gi3_26077436_x50...gguucgauucccggcgccggagcU..................... 1
Gi3_26058286_x50...gCuucgauucccggcgccggag....................... 1
Gi3_26077036_x50..guucgauuNccggcgccggagc...................... 1
Gi3_26076736_x50.................uccggcguNguauaacggc.. 1
Gi3_26064286_x50...gguucgauNcccggcgccgg......................... 1
Gi3_26057186_x50..Aguucucacccaacagauccg... 1
Gi3_26079272_x49..guucgGuucccggcgccgg......................... 1
Gi2_9757944_x49..guucgauucccggcgccAga........................ 1
Gi1_3652936_x49...gguucgauCcccggcgccgg......................... 1
Gi3_26081820_x49...gAuucgauucccggcgccggag....................... 1
Gi5_7894955_x49.......................guaguauaacggcuagaau.. 0
Gi2_9755445_x49...gguucgauucccggcgccggagcCu.................... 1
Gi1_3647644_x49..auccggguucgauucccggcgccgga........................ 0
Gi5_7897160_x49..Uauucccggcgccggagc...................... 1
Gi3_26081134_x49.................uccggcguaguauGacggcuaga.. 1
Gi5_7894759_x49.................uccggcguaguauaacAgcuaga.. 1
Gi1_3647301_x49...gguucgauNcccggcgccgg......................... 1
Gi3_26116112_x48.................uccggcguaguauaacAgc.. 1
Gi3_26110784_x48.................uccggcguaguCuaacggcuaga.. 1
Gi3_26101520_x48.................uccggcguaguauaUcggcuaga.. 1
Gi3_26103488_x48.................uccggcguaguauCacggcua.. 1
Gi2_9772973_x48...gguucgCuucccggcgccggagc...................... 1
Gi2_9777389_x48..guCcgauucccggcgccgg......................... 1
Gi3_26097632_x48.................uccggcguaguauaacAgcua.. 1

```
Gi2_9775181_x48.................uccggcguaguauaacAgcuaga........................................................................     1
Gi2_9773741_x48...................................................................Aguucgauucccggcgcc...........................     1
Gi3_26113280_x48...................................................................gguucgauCcccggcgccgga........................     1
Gi1_3657785_x48...................................................................Aguucgauucccggcgccgg.........................     1
Gi1_3653081_x48....................................................................guucgauucccggcgccggagcA.....................     1
Gi2_9784902_x47....................................................................guucgauucccggcgccggCgc......................     1
Gi1_3662555_x47....................................................................guucgauucccggcgUcgg.........................     1
Gi3_26130456_x47...................................................................gguucgGuucccggcgccggag.......................     1
Gi2_9784714_x47...................................................................gguucgauucccggcgccggagU......................     1
Gi5_7923906_x47...................................................................gguucgauucccCgcgccggag.......................     1
Gi2_9786124_x47.................Gccggcguaguauaacggcuag.........................................................................     1
Gi3_26125662_x47...............................................gGucucacccaacagauccgg............................................     1
Gi2_9805034_x46...................................................................gguucgauucccggUgccggagc......................     1
Gi5_7940367_x46....................................................................guucgGuucccggcgccg..........................     1
Gi3_26145785_x46.................uccggcguaguauaGcggc............................................................................     1
Gi2_9796478_x46....................................................................guucgauucccggcgccggGgc......................     1
Gi5_7931489_x46.................uGcggcguaguauaacggc............................................................................     1
G1a_5280140_x46...................................................................gUuucgauucccggcgccggagc......................     1
Gi3_26153651_x46...................................................................gguucgauuUccggcgccggag.......................     1
Gi5_7937975_x46....................................................................Cuucgauucccggcgccgg.........................     1
Gi3_26142795_x46.................uGcggcguaguauaacggcuag.........................................................................     1
Gi3_26153973_x46..................................................................ggguucgauucccggcgccg..........................     0
Gi5_7938021_x46....................................................................guucgauuNccggcgccg..........................     1
Gi3_26152501_x46...................................................................gguucgauCcccggcgccggag.......................     1
Gi3_26161378_x45.................uccggcguaguauCacgg.............................................................................     1
Gi2_9814704_x45...................................................................gguucgauucccggcgcUggagc......................     1
Gi5_7946502_x45....................................................................guCcgauucccggcgccgg.........................     1
Gi2_9812634_x45.................uccggcguaguauaacggcuagC........................................................................     1
Gi2_9809439_x45......................................................................Gcgauucccggcgccggagc......................     1
Gi3_26169523_x45.................uccggcguaguauaacggcuG..........................................................................     1
Gi2_9809574_x45...................................................................gguucgauucccgAcgccggagc......................     1
Gi3_26181005_x44.................uccggcguaguauaacggcuagU........................................................................     1
Gi1_3679485_x44....................................................................guGcgauucccggcgccgg.........................     1
Gi3_26186901_x44...................................................................gguucgauucccAgcgccgga........................     1
Gi5_7956443_x44................................................Cucucacccaacagauccg.............................................     1
Gi2_9825942_x44...................................................................gguucgauucccggcgccggagcA.....................     1
Gi2_9830694_x44.................uccggcguaguauaGcggcuaga........................................................................     1
Gi2_9841797_x43....................................................................guCcgauucccggcgccggagc......................     1
Gi5_7971399_x43.................uccggcguaguauaacggcCaga........................................................................     1
Gi5_7973936_x43....................................................................guucgauuNccggcgccgg.........................     1
Gi5_7971012_x43.................uccggcguNguauaacggcuaga........................................................................     1
G1a_5301606_x43...................................................................Uguucgauucccggcgccggag.......................     1
Gi2_9846570_x43.................uccggcguaguauaacggcCaga........................................................................     1
Gi5_7984822_x42....................................................cacccaacagauccggguucga......................................     0
Gi2_9852685_x42...................................................................gguucgauucUcggcgccggagc......................     1
Gi3_26228227_x42.................uccgUcguaguauaacggcua..........................................................................     1
Gi5_7990408_x42.................uccggcguaguauaacgAcua..........................................................................     1
```

Gi3_26226589_x42.................uGcggcguaguauaacggcuagaau..   1
Gi2_9855499_x42.................Gccggcguaguauaacggcuagaau..   1
Gi2_9856885_x42.................uccggcguGguauaacggc..   1
Gi2_9858523_x42...gguucgauucccggcgccAgagc......................   1
Gi2_9851257_x42..cggguucgauucccggcgccgga.........................   0
Gi5_7979992_x42.................uGcggcguaguauaacggcua..   1
Gi2_9849745_x42....................ggcguaguauaacggcuaga..   0
Gi3_26229739_x42.................uccggcguaguauaGcgg...   1
Gi3_26228521_x42..guCcgauucccggcgccggagc......................   1
Gi1_3688259_x42...ugguucucNcccaacagauccgg..   1
Gi2_9855625_x42.................uccggUguaguauaacggcuagaa...   1
Gi5_7989610_x42...gguucgauucccggcgccggagcgA....................   1
Gi5_8004564_x41..gguucucacccCacagauccg...   1
Gi5_8000628_x41.................Cccggcguaguauaacggcuag...   1
Gi3_26256018_x41.................uccggcguagCauaacggcua..   1
Gi5_7998250_x41..gguucucacccaacGgauccg...   1
Gi5_7996774_x41...............aguccggcguaguauaacggcuagaaugg..   0
Gi1_3695561_x41..................ccggcguaguauaacggcu...   0
Gi3_26241340_x41.................Cccggcguaguauaacggcua..   1
Gi5_7996569_x41...gguucgauNcccggcgccg..........................   1
Gi5_7993863_x41...Aguucgauucccggcgccg..........................   1
Gi1_3695766_x41..guAcgauucccggcgccgg.........................   1
Gi3_26260364_x41..auccggguucgauucccggcgccggagc......................   0
Gi2_9863452_x41..guucgaCucccggcgccggag.......................   1
Gi3_26252492_x41...gguucgauucccggcgccggUgc......................   1
Gi5_7993658_x41...guucucacccCacagauccg...   1
Gi2_9877330_x40...Ggguucucacccaacagauccg...   1
Gi3_26276090_x40..guucgauucccggcgccAga........................   1
Gi1_3697317_x40...gUuucgauucccggcgccgga........................   1
Gi3_26276010_x40...gguucgauAcccggcgccggagc......................   1
Gi1_3698477_x40..guucgauucccgAcgccgg.........................   1
Gi3_26266650_x40.................uUcggcguaguauaacggc..   1
Gi3_26263370_x40...uUguucucacccaacagauc...   1
Gi5_8014854_x40.................uccggcguaguauaacggcCag...   1
Gi3_26263930_x40...gCuucgauucccggcgccgga........................   1
Gi3_26275650_x40.................uUcggcguaguauaacgg...   1
Gi3_26282010_x40.................uccggAguaguauaacggcuaga..   1
Gi5_8026280_x39..guucgauucccggcAccgg.........................   1
Gi3_26299059_x39..Cuucgauucccggcgccggagc......................   1
Gi1_3704321_x39...guugguucucacccaacagauccgggu..   0
Gi3_26283693_x39...gguucgaCucccggcgccggag.......................   1
Gi3_26305026_x39..agauccggguucgauucccggcgccggagcC.....................   1
Gi2_9893236_x39..Cuucgauucccggcgccgg.........................   1
Gi3_26305572_x39...gguucgauucAcggcgccggagc......................   1
Gi3_26327082_x38.................uUcggcguaguauaacggcua..   1
Gi2_9912081_x38..guucgaCucccggcgccgga........................   1
Gi2_9905051_x38...uccggguucgauucccggcgccgga........................   0

```
Gi3_26320546_x38.................uccggUguaguauaacggcua..........................................................................    1
Gi1_3714126_x38.................uccggcguNguauaacggcuag.........................................................................    1
Gi2_9913069_x38...................................................................gguucgauNcccggcgccggag.......................    1
Gi3_26326702_x38....................................................................guucgauucccggUgccgg.........................    1
Gi3_26320318_x38...................................................................gguucgauucccggcAccgg.........................    1
Gi3_26318608_x38...................................................................gguucgGuucccggcgccgga........................    1
Gi1_3709984_x38...................................................................gUuucgauucccggcgccggag.......................    1
Gi3_26311426_x38...................................................................gguucgauucccggcgccggCg.......................    1
Gi2_9911093_x38....................................................................guucgauuNccggcgccgg.........................    1
Gi5_8033481_x38.....................................................acccaacagauccggguucgauucc..................................    0
Gi2_9909383_x38....................................................................guucgGuucccggcgccggagc......................    1
Gi5_8031125_x38....................................................................guucgauucccggcgccA..........................    1
Gi1_3713404_x38.................uGcggcguaguauaacggcua..........................................................................    1
Gi3_26331161_x37....................................................................Auucgauucccggcgccggag.......................    1
Gi1_3717732_x37....................................................................guucgauucccggcgAcgg.........................    1
Gi5_8052664_x37...................................................................Cguucgauucccggcgccggagc......................    1
Gi2_9924698_x37...................................................................gguucgauucccggcgAc...........................    1
Gi5_8043118_x37..............................................gguucCcacccaacagauccg.............................................    1
Gi3_26335638_x37...................................................................gguucgauucccggcgccA..........................    1
Gi5_8046744_x37...................................................................gguucgauNcccggcgccgga........................    1
Gi1_3718472_x37.................uccggcguGguauaacggcua..........................................................................    1
Gi3_26345147_x37...................................................................ggAucgauucccggcgccgga........................    1
Gi3_26349402_x37...................................................................gguucgauucccggcgccgAag.......................    1
G1a_5352502_x37.................Gccggcguaguauaacggcuaga........................................................................    1
Gi2_9921627_x37.................uUcggcguaguauaacggcuaga........................................................................    1
Gi2_9916965_x37...................................................................Aguucgauucccggcgccg..........................    1
Gi3_26332530_x37.................uccggcAuaguauaacggcuaga........................................................................    1
Gi2_9923107_x37...................................................................gguucgauNcccggcgcc...........................    1
Gi1_3715031_x37....................................................................guucgauucccggcgccggag.......................    0
Gi5_8065660_x36..............................................Uguucucacccaacagauccg.............................................    1
Gi5_8055436_x36..........................................................................................guuucccgccuuccuucgucu.    0
Gi1_3721587_x36..................................................cucacccaacagauccggguucgauucc..................................    0
Gi3_26352767_x36...................................................................gguucgauucccggUgccgga........................    1
Gi3_26352083_x36....................................................................guucgauucccggcgccggGgc......................    1
Gi3_26358887_x36.................uccgUcguaguauaacgg.............................................................................    1
Gi3_26372855_x36...................................................................gguucgauucccggcgccggC........................    1
Gi2_9939771_x36...................................................................gguucgauucccggcgccggaAc......................    1
Gi5_8063536_x36.................uccggcguaguauaGcggcuag.........................................................................    1
Gi5_8058424_x36..................................................cucacccCacagauccg.............................................    1
Gi3_26353271_x36................................................uGcucacccaacagauccgg............................................    1
Gi2_9939159_x36...................................................................gguucgauucccggcgccgAagc......................    1
Gi3_26361443_x36...................................................................gguCcgauucccggcgccggag.......................    1
Gi2_9929187_x36...................................................................gguucgauucccggAgccggagc......................    1
Gi5_8061016_x36..............................................gguucucaNccaacagauccg.............................................    1
Gi3_26359031_x36...................................................................gguucgauucccggcAccgga........................    1
Gi3_26393525_x35....................................................................guucgauucccAgcgccgg.........................    1
Gi1_3728247_x35....................................................................guucgauucccggAgccgg.........................    1
```

Gi3_26382465_x35.................uccggcguaAuauaacggcuaga..	1
G1a_5365600_x35..guucgauucccggcgccgga........................	0
Gi5_8068229_x35..guucgaCucccggcgccgga........................	1
Gi1_3728352_x35.................uccggcguaguauaGcgg...	1
Gi3_26394540_x35..guucAauucccggcgccgg.........................	1
Gi5_8072429_x35..Cuucgauucccggcgccg..........................	1
Gi2_9949979_x35...gguuGgauucccggcgccggagc......................	1
Gi3_26378230_x35.................uccggcguaguauaacggcuagaaC..	1
Gi2_9948054_x35.................uGcggcguaguauaacggcua..	1
Gi5_8076769_x35.................uccggcguaguGuaacggcuaga..	1
G1a_5364060_x35...ugguucucacccaacagauccg...	0
Gi3_26394120_x35...uccggguucgauucccggcgccg..........................	0
Gi3_26381415_x35..agauccggguucgauucccggcgccggagc......................	0
Gi3_26384355_x35...ggCucgauucccggcgccg..........................	1
Gi5_8077714_x35..guucUauucccggcgccgg.........................	1
Gi5_8069594_x35.................uccggcguaguauaacAgcuag...	1
Gi3_26377950_x35..guCcgauucccggcgccgga........................	1
Gi1_3735733_x34.................ucUggcguaguauaacgg...	1
Gi1_3737467_x34.................uccggcguNguauaacgg...	1
Gi1_3735189_x34..................ccggcguaguauaacggcuag...	0
Gi5_8088935_x34...guucucacNcaacagauccg...	1
Gi3_26414972_x34..guucgauucccggUgccgga........................	1
Gi1_3735325_x34...guucucacccCacagauccg...	1
Gi5_8090499_x34.................uccggcguGguauaacggcuag...	1
Gi2_9958755_x34...Aguucgauucccggcgccgga........................	1
Gi3_26398108_x34..uCcucacccaacagauccg...	1
Gi5_8084005_x34.................uccggcguaguauaacgAcuaga..	1
Gi5_8085025_x34..cGcacccaacagauccgg..	1
Gi5_8085535_x34..guCcgauucccggcgccg..........................	1
Gi2_9967459_x34...gguucgauuUccggcgccggagc......................	1
Gi2_9965147_x34...gguucgauNcccggcgccg..........................	1
Gi2_9956477_x34...gguucgauucccUgcgccggagc......................	1
Gi5_8085263_x34.................ucUggcguaguauaacggcua..	1
Gi3_26413068_x34...gguucgauucccAgcgccggag.......................	1
Gi5_8088731_x34..gguucucacccGacagauccg...	1
Gi2_9982581_x33...gguucgauAcccggcgccggagc......................	1
Gi3_26434465_x33..guucgauucccggcgccA..........................	1
Gi3_26424796_x33.................uccggcguaguauaUcggcua..	1
Gi5_8099352_x33.................uUcggcguaguauaacggcuag...	1
Gi3_26436412_x33.................ucUggcguaguauaacggcuag...	1
Gi3_26426050_x33..guucUauucccggcgccgg.........................	1
Gi3_26430076_x33......................cguaguauaacggcuaga..	0
Gi3_26424631_x33.................uccgUcguaguauaacggc..	1
Gi3_26436082_x33.................uccggcguaguauUacggcuaga..	1
Gi2_9980106_x33...gguucgauucccggcgUcggagc......................	1
Gi3_26424136_x33...gguCcgauucccggcgccgga........................	1
Gi1_3738717_x33...uccggguucgauucccggcgccgga........................	0

```
Gi5_8097372_x33...................................................................gguucgauucccggcgccggGgc......................	1
Gi2_9978951_x33...................................................................gguucgauucccggcgccggCg.......................	1
Gi1_3742776_x33..............................................Aguucucacccaacagauccg.............................................	1
Gi5_8105061_x33...................................................................gguucgGuucccggcgccggagc......................	1
Gi5_8099451_x33....................................................................guucgauucccggcgcUg..........................	1
Gi5_8099088_x33.................uUcggcguaguauaacggcuaga........................................................................	1
Gi1_3743568_x33................................................uucucaccNaacagauccg.............................................	1
Gi3_26426809_x33...................................................................Aguucgauucccggcgcc...........................	1
Gi3_26442022_x33....................................................................guucgauucccggcgccAg.........................	1
Gi3_26428129_x33...................................................................gguucgauuccUggcgccgga........................	1
Gi3_26469278_x32...................................................................gguucgauucccggcgcUgga........................	1
Gi3_26460286_x32...................................................................gAuucgauucccggcgccgga........................	1
Gi5_8108550_x32...................................................................gAuucgauucccggcgccggagc......................	1
Gi3_26452766_x32.................ucAggcguaguauaacggcuaga........................................................................	1
Gi2_9993405_x32.................uGcggcguaguauaacggc............................................................................	1
Gi2_9996477_x32.................uccggcguaguGuaacggcuaga........................................................................	1
Gi3_26450590_x32....................................................................guuUgauucccggcgccgga........................	1
Gi2_9997213_x32.................uccggcguGguauaacggcuaga........................................................................	1
Gi1_3747552_x32.............................................ugguucucacccaacagaucc..............................................	0
Gi5_8111366_x32..................................................cucacccaNcagauccg.............................................	1
Gi5_8114374_x32.................uccggcguaguauaGcgg.............................................................................	1
Gi3_26457758_x32...................................................................gguucgauuAccggcgccggagc......................	1
Gi3_26457918_x32...................................................................ggGucgauucccggcgccgga........................	1
Gi5_8116710_x32.................uccggcguaguGuaacggcuag.........................................................................	1
Gi3_26455070_x32.................Cccggcguaguauaacggc............................................................................	1
Gi1_3745344_x32....................................................................guucgauucccgUcgccgg.........................	1
Gi3_26466494_x32...................................................................ggGucgauucccggcgccggag.......................	1
Gi1_3744160_x32.................uccggcguaguauaacAgcuag.........................................................................	1
Gi3_26468638_x32................................................uucucacccaCcagauccg.............................................	1
Gi3_26446270_x32..........................................................acagauccggguucgauucccggcgccgg.........................	0
Gi3_26487574_x31...................................................................gguuAgauucccggcgccggagc......................	1
Gi3_26478336_x31....................................................................guucUauucccggcgccgga........................	1
Gi5_8126192_x31...................................................................gguucgaCucccggcgccggagc......................	1
Gi2_10008326_x31...............................................guucucacccaacagauccgggu..........................................	0
Gi5_8127401_x31.................uUcggcguaguauaacggcuagaau......................................................................	1
Gi3_26472911_x31....................................................................guuUgauucccggcgccgg.........................	1
Gi5_8130315_x31....................................................................guucgauucccggcAccg..........................	1
Gi5_8120085_x31.................uccggcguaguauaGcggcuaga........................................................................	1
Gi1_3754020_x31................................................uuUucacccaacagauccg.............................................	1
Gi2_10011860_x31....................................................................guucgauucccggcgccgC.........................	1
Gi1_3754578_x31..................................................................ggguucgauucccggcgccgga........................	0
Gi5_8118814_x31.................uccggcguGguauaacggcua..........................................................................	1
Gi4_490729_x31.................uccggcguaguauaacggcuag.........................................................................	0
Gi3_26491728_x31....................................................................guucgauuccUggcgccgg.........................	1
Gi3_26471795_x31.................uccggcguaguGuaacgg.............................................................................	1
Gi3_26473655_x31....................................................................guucgauCcccggcgccgg.........................	1
Gi3_26477902_x31............................................................agauccggguucgauucccggcgccggag.......................	0
```

```
Gi2_9999863_x31...................................................................Cuucgauucccggcgccggagc.....................     1
Gi3_26480072_x31..................................................................gguucgaCucccggcgccgg........................     1
Gi2_10012480_x31.................Cccggcguaguauaacggcuaga......................................................................     1
Gi2_10002994_x31.................uccggcguaguauCacggcuaga......................................................................     1
Gi3_26475019_x31...................................................................guucgauCcccggcgccgga.......................     1
Gi5_8141425_x30..................................................................gguucgauucccggcgccggC.......................     1
Gi5_8133235_x30.................uccggcguaguauaacggcuagaCu....................................................................     1
Gi2_10024765_x30...............................................uucucacccaacGgauccg............................................     1
Gi1_3758915_x30.............................................................auccggguucgauucccggcgccgg........................     0
Gi3_26516520_x30...................................................................guucgauucccggcgUcgga.......................     1
Gi1_3756935_x30.................uccggcguaguauaGcggcuag.......................................................................     1
Gi2_10027315_x30.................Gccggcguaguauaacggcuagaa.....................................................................     1
Gi1_3761195_x30.................Gccggcguaguauaacggc..........................................................................     1
Gi3_26506200_x30..................................................................gguucAauucccggcgccgga.......................     1
Gi1_3756845_x30........................................................aacagauccggguucgauucccggcgccgg........................     0
Gi5_8141005_x30.................uccggcguaguauaacggcuagaaug...................................................................     0
Gi3_26512770_x30...................................................................guucgauucccAgcgccgga.......................     1
Gi5_8140135_x30.............................................gguucucGcccaacagauccg............................................     1
Gi5_8137855_x30...................................................................guucgauuccUggcgccg.........................     1
Gi3_26513970_x30.................uccggcguaguauUacgg...........................................................................     1
Gi2_10028005_x30..................................................................Cguucgauucccggcgcc..........................     1
Gi5_8136295_x30.................ucUggcguaguauaacggc..........................................................................     1
G1a_5399946_x30..................................................................Uguucgauucccggcgccg.........................     1
Gi1_3758705_x30.................................................cucacccaacagauccggguucga.....................................     0
Gi2_10018675_x30...................................................................guucgauucccAgcgccggagc.....................     1
Gi5_8140915_x30.................uccggcguaguauaacggcuagC......................................................................     1
Gi3_26528828_x29...................................................................guucAauucccggcgccgga.......................     1
Gi2_10032948_x29..........................................................cagauccggguucgauucccggcgccggag......................     0
Gi5_8148972_x29..............................................gCucucacccaacagauccg............................................     1
Gi5_8148740_x29....................................................acccaacagauccggguucgauuccc................................     0
Gi2_10036109_x29.................uccggcguGguauaacggcua........................................................................     1
Gi5_8143462_x29.................uccgUcguaguauaacggcuaga......................................................................     1
Gi1_3764610_x29...................................................................guucgauucccggcgccggagcgA...................     1
Gi3_26542980_x29..................................................................gguucgauucccggcgAcggag......................     1
Gi5_8145608_x29.................ucUggcguaguauaacgg...........................................................................     1
Gi1_3767597_x29...................................................................guucgauuUccggcgccgg........................     1
Gi3_26538456_x29.................uccggcguaguauaacgAcua........................................................................     1
Gi5_8150915_x29................................................Gcucacccaacagauccg............................................     1
Gi1_3764407_x29....................................................acccaacagauccggguucgauucccg...............................     0
Gi1_3767858_x29...................................................................guucgauucccggcgccCg........................     1
Gi3_26539384_x29.................uccggcguagCauaacggc..........................................................................     1
Gi5_8151785_x29.................uccggcguGguauaacggcuaga......................................................................     1
Gi5_8143752_x29.................ucUggcguaguauaacggcuagaau....................................................................     1
Gi3_26539645_x29....................................................................Gucgauucccggcgccggagc.....................     1
Gi3_26538688_x29..................................................................gguucgauucccggcgGcggagc.....................     1
Gi5_8154772_x29.............................................gguCcucacccaacagauccg............................................     1
Gi2_10029874_x29..................................................................gguCcgauucccggcgcc..........................     1
```

Gi5_8144825_x29..guucgGuucccggcgccgg.........................   1
Gi3_26527030_x29..guucgauucccggcgcUgga........................   1
Gi3_26537209_x29..guucgauuNccggcgccggag.......................   1
Gi5_8153061_x29.................uccggcguaguauaacggUuag...   1
Gi5_8152887_x29..guucgauuccUggcgccgg.........................   1
Gi3_26523260_x29.................uccggcgCaguauaacgg...   1
Gi2_10057461_x28.................uccggcguaguauaacggcuaAa..   1
Gi2_10045757_x28...gguuAgauucccggcgccggagc......................   1
Gi5_8163133_x28..gguucucacccaCcagauccg...   1
Gi5_8162657_x28...gCuucgauucccggcgccggagc......................   1
Gi5_8158877_x28..uucucaccNaacagauccg...   1
Gi3_26554693_x28...gguucgauucccggcgAcgga........................   1
Gi2_10055053_x28...gguucgauucccggcgccggG........................   1
Gi3_26559173_x28.................uccggcguaguauCacggc..   1
Gi3_26555953_x28..guucgauuccUggcgccggagc......................   1
Gi5_8156945_x28..guucgauucccggcgUcgg.........................   1
Gi3_26556541_x28..guucgauucccggcAccgga........................   1
Gi3_26554441_x28..cucacccaacagauccggguucgauucc..................................   0
Gi5_8160949_x28...gguucgauNcccggcgccggag.......................   1
Gi2_10045729_x28..guucgauuccUggcgccggagc......................   1
Gi3_26547861_x28..guucgGuucccggcgccggag.......................   1
Gi1_3770940_x28...Uguucgauucccggcgccggag.......................   1
Gi3_26545705_x28.................uccggcguaguauaacggcuGg...   1
Gi5_8166913_x28...gguucgauucccggcgAc...........................   1
Gi2_10046429_x28...gguucgauucAcggcgccggagc......................   1
Gi3_26545733_x28.................Cccggcguaguauaacgg...   1
Gi1_3779598_x27.................uccggcguaguauaacggcCag...   1
Gi3_26582799_x27...Gcucacccaacagauccg...   1
Gi2_10059887_x27.................ucUggcguaguauaacggc..   1
Gi3_26577156_x27..guucgaGucccggcgccggagc......................   1
Gi3_26582178_x27.................ucUggcguaguauaacggcuagaa...   1
Gi2_10066853_x27....................Cgcguaguauaacggcuaga..   1
G1a_5426177_x27...Uguucgauucccggcgccgg.........................   1
Gi5_8169947_x27...gauccggguucgauucccggcgccgga........................   0
Gi3_26588604_x27...gguucgauucccggcAccggag.......................   1
Gi2_10059158_x27..guucUauucccggcgccgg.........................   1
Gi3_26590710_x27...aacagauccggguucgauucccggcgccgga........................   0
Gi3_26578533_x27..cucacccaacagauccggguucgauu....................................   0
Gi1_3780354_x27...gguucgauucccggcgccgU.........................   1
Gi3_26586768_x27....................Ugcguaguauaacggcuaga..   1
Gi5_8178101_x27..gguucucacccaGcagauccg...   1
Gi5_8170649_x27..guucgauCcccggcgccg..........................   1
Gi3_26585094_x27...ggAucgauucccggcgccggag.......................   1
Gi5_8171027_x27.................uccggcguaguauaaUggcuaga..   1
Gi3_26592168_x27..guucgauucccAgcgccggagc......................   1
Gi3_26588037_x27.................uccggcguaguauaacggcuGgaa...   1
Gi3_26587578_x27...gguucgauucccggcgccAga........................   1

```
Gi1_3776601_x27....................................................................guucgaCucccggcgccgga........................ 1
Gi3_26593599_x27.................ucUggcguaguauaacggcuagaau...................................................................... 1
Gi5_8180396_x27.................uccggcguaguauaacAgcua.......................................................................... 1
Gi2_10067744_x27....................................................................guucgauucccggcAccgg......................... 1
Gi3_26600531_x26..........................................................acagauccggguucgauucccggcgccggagc...................... 0
Gi5_8189398_x26.................uccggcguaguauCacggcuaga........................................................................ 1
Gi5_8187812_x26.................uccgUcguaguauaacggcuag......................................................................... 1
Gi5_8194208_x26...................................................................gguucgauucccggUgcc........................... 1
Gi1_3786287_x26.................uccggcguaguGuaacggcuaga........................................................................ 1
Gi5_8186200_x26....................................................................guucgauucccggUgccg.......................... 1
Gi1_3785741_x26................................................uucucacccaCcagauccg............................................. 1
Gi1_3781945_x26....................................................................guucgauucccggcgccgA......................... 1
Gi3_26619511_x26.................uccggcguaguauaaUggcua.......................................................................... 1
Gi3_26620993_x26.................uccggcguaguaCaacgg............................................................................. 1
Gi1_3784623_x26.............................................ugguucucGcccaacagauccgg............................................ 1
Gi5_8186590_x26....................................................................guucgauucccAgcgccg.......................... 1
Gi3_26621123_x26.....................................................................Gucgauucccggcgccgga........................ 1
Gi5_8191868_x26..................................................Gucacccaacagauccgg............................................ 1
Gi5_8183444_x26....................................................................guGcgauucccggcgccg.......................... 1
Gi3_26601935_x26...................................................................gguucgauucUcggcgccggag....................... 1
Gi2_10082815_x26................................................uGcucacccaacagauccg............................................. 1
Gi1_3786209_x26.................uccggUguaguauaacggcuag......................................................................... 1
Gi2_10076679_x26....................................................................guucgGuucccggcgccgg......................... 1
Gi3_26619537_x26.................uccggcguaguauaGcggcuag......................................................................... 1
Gi3_26596267_x26....................................................................guucgauucccggcgAcgg......................... 1
Gi1_3784987_x26.................uGcggcguaguauaacggcuaga........................................................................ 1
Gi1_3790881_x25....................................................................gAucgauucccggcgccgg......................... 1
Gi5_8198434_x25...............................................guucucacccaacagGuccg............................................. 1
Gi5_8201084_x25..............................................Cguucucacccaacagauccg............................................. 1
Gi3_26629968_x25...............................................Uuucucacccaacagauccggg........................................... 1
Gi5_8196184_x25.................uccggcguNguauaacggcuagaau...................................................................... 1
Gi1_3792481_x25................................................uuUcacccaacagauccgg............................................. 1
Gi3_26640343_x25....................................................................guucgauucGcggcgccggagc...................... 1
Gi5_8203184_x25...................................................................gguuUgauucccggcgccggagc...................... 1
Gi2_10097647_x25...................................................................gguucgauucccggUgcc........................... 1
Gi2_10104647_x25....................................................................guucgauucccggcgccAg......................... 1
Gi3_26629143_x25.................uccggAguaguauaacggcua.......................................................................... 1
Gi5_8201009_x25...............................................guucucacccaCcagauccg............................................. 1
Gi3_26638018_x25...................................................................gguucgauuccAggcgccgg......................... 1
Gi5_8200709_x25................................................uuUcacccaacagauccg.............................................. 1
Gi5_8207059_x25....................................................................guucgauucccggcgccAg......................... 1
Gi3_26649193_x25...................................................................gguucgauucccggUgccggag....................... 1
Gi5_8199184_x25.................uccggcguaguauaacggcuagaaA...................................................................... 1
Gi2_10100297_x25.........................................................aacagauccggguucgauucccggcgccg.......................... 0
Gi5_8196384_x25..............................................gguucucacUcaacagauccg............................................. 1
Gi3_26640368_x25.................uccggcguaguGuaacggc............................................................................ 1
Gi1_3790031_x25...............................................guucucacNcaacagauccg............................................. 1
```

Gi2_10100072_x25...gguucgauNcccggcgccgga........................ 1
G1a_5444687_x25...gUuucgauucccggcgccggag....................... 1
Gi2_10095222_x25..guuUgauucccggcgccggagc...................... 1
Gi5_8196534_x25.................uccggcguaguauaacgAcuag... 1
Gi3_26645368_x25..guucgauucccggcAccgg......................... 1
Gi3_26624893_x25..guucgauucUcggcgccg.......................... 1
Gi2_10097697_x25.................uccggcguaguauaacgAcuaga.. 1
Gi1_3790131_x25..guucgaCucccggcgccg.......................... 1
G1a_5450987_x25...Uguucgauucccggcgccgga........................ 1
Gi3_26633193_x25...gguucgauNcccggcgcc........................... 1
Gi3_26647618_x25...gguuUgauucccggcgccggag....................... 1
Gi2_10090447_x25..guucgauuccUggcgccgg......................... 1
Gi3_26646318_x25.................uccggcguaguauaacAgcuagaau.. 1
Gi5_8197809_x25...Aguucgauucccggcgccgga........................ 1
Gi3_26621768_x25...gguucgCuucccggcgccgga........................ 1
Gi1_3795686_x24.................uccggcguaguauaacggcuagG.. 1
Gi5_8218152_x24.................uccggcguGguauaacgg... 1
Gi3_26665520_x24...gguucgaCucccggcgccg.......................... 1
Gi5_8207976_x24.................Gccggcguaguauaacggcuagaa... 1
Gi3_26675528_x24...gguucgauucccggcgccggUg....................... 1
Gi3_26674952_x24...gguucgauucccggcgUcgga........................ 1
Gi3_26650424_x24.................ucGggcguaguauaacggcuaga.. 1
Gi1_3795806_x24.................uccggcguaguauaacggcuGga.. 1
Gi3_26671712_x24...gguuUgauucccggcgccgga........................ 1
Gi2_10115803_x24..guucgauucccggUgccggagc...................... 1
Gi3_26679536_x24.................uccggcguagCauaacgg... 1
Gi5_8217048_x24....................Ugcguaguauaacggcuag... 1
Gi3_26660288_x24..gguucucaNccaacagauccg... 1
Gi1_3795614_x24...ugguucucNcccaacagauccg... 1
Gi3_26675624_x24..guucgauCcccggcgccggagc...................... 1
Gi3_26668208_x24.................uccggcguaguauGacgg... 1
Gi5_8207304_x24.................uccggcguaguauaacggcuGgaau.. 1
Gi3_26658896_x24..guucgauucccggcgUcgg......................... 1
Gi5_8208528_x24..gguucucacccaacagGuccg... 1
Gi2_10120123_x24.................uccggcguaguauaacggUuaga.. 1
Gi5_8209344_x24.................uccggcguaguaCaacggcuaga.. 1
Gi3_26661824_x24..gguCcucacccaacagauccg... 1
Gi5_8218584_x24..guucgauucccggcgccggagcguuucc................ 0
Gi3_26664320_x24.................uUcggcguaguauaacggcuag... 1
Gi3_26675888_x24.................uccgCcguaguauaacggcuaga.. 1
Gi3_26666672_x24..aUauccggguucgauuccc................................. 1
Gi5_8219064_x24...uUguucucacccaacagauccgggu.. 1
Gi1_3798686_x24...gguucgaCucccggcgccgg......................... 1
Gi2_10111627_x24.................uccggcguNguauaacggc.. 1
Gi5_8207880_x24.................uccggcguagCauaacggcuaga.. 1
Gi1_3794750_x24.................ucUggcguaguauaacggcuag... 1
Gi3_26663672_x24...gguucAauucccggcgccggag....................... 1

```
Gi3_26663600_x24.................uccggcguGguauaacggcuag.........................................................................    1
Gi3_26687230_x23...................................................................ggGucgauucccggcgccg..........................    1
Gi5_8221616_x23...............................................guucucacccaGcagauccg.............................................    1
Gi1_3800485_x23....................................................................guucgauucccggcgccggaU.......................    1
Gi4_499091_x23...................................................................gUuucgauucccggcgccgg.........................    1
Gi5_8223571_x23....................................................................guucgauucccggcgccggagcgA....................    1
Gi3_26697948_x23...................................................................gguucgauucUcggcgccgga........................    1
Gi3_26708827_x23................................................uuUucacccaacagauccg.............................................    1
Gi2_10130648_x23...............................................guucucacccCacagauccg.............................................    1
Gi5_8227987_x23.................uccggcguNguauaacggcua..........................................................................    1
Gi5_8225756_x23...............................................Auucucacccaacagauccg.............................................    1
Gi5_8228700_x23....................................................................guucAauucccggcgccg..........................    1
Gi3_26708482_x23..............................................gguucucacccaGcagauccg.............................................    1
Gi5_8229942_x23...................................................................Cguucgauucccggcgccg..........................    1
Gi2_10137065_x23....................................................................gCucgauucccggcgccgg.........................    1
Gi5_8225825_x23.................uccggUguaguauaacggcuag.........................................................................    1
Gi1_3799979_x23....................................................................guucgCuucccggcgccgg.........................    1
Gi5_8223433_x23................................................Gucucacccaacagauccgg............................................    1
Gi5_8228263_x23...................................................................gguucgauuccUggcgccggagc......................    1
Gi5_8222398_x23....................................................................guucgauucccggcgccggagcA.....................    1
Gi2_10128003_x23....................................................................guucUauucccggcgccggagc......................    1
Gi5_8232656_x23.................uccggcguaguauaacggcuagaaugg....................................................................    0
Gi3_26687529_x23...................................................................gguAcgauucccggcgccggag.......................    1
Gi5_8231069_x23....................................................................guucUauucccggcgccg..........................    1
Gi5_8226906_x23..............................................Uguucucacccaacagauc...............................................    1
Gi2_10122253_x23.................uccggcguaguauaacggcuGgaau......................................................................    1
Gi3_26684470_x23.................uccggcguaguauaacggcuagaCu......................................................................    1
Gi3_26686057_x23.................uccggcguNguauaacggcuag.........................................................................    1
Gi2_10126186_x23.................uccggcguNguauaacggcua..........................................................................    1
Gi3_26689323_x23.................uccggcguaguauaacggUua..........................................................................    1
Gi5_8230356_x23.................uccggcguaguauaacggcuGg.........................................................................    1
Gi4_499827_x23...................................................................Uguucgauucccggcgccgg.........................    1
Gi3_26682400_x23.................uccggcguNguauaacggcuagaa.......................................................................    1
Gi3_26697833_x23...................................................................gguucgauCcccggcgccg..........................    1
Gi1_3804694_x23...............................................guucucacccCacagauccgg............................................    1
Gi1_3805131_x23...............................................guCcucacccaacagauccg.............................................    1
Gi5_8220144_x23....................................................................guuUgauucccggcgccgg.........................    1
Gi3_26688150_x23.................uccggcguaguauaacAg.............................................................................    1
Gi5_8227343_x23...................................................................gguucgauucccAgcgccggagc......................    1
Gi3_26701122_x23....................................................................guucgauucccgAcgccggagc......................    1
Gi1_3813437_x22...................................................................gAuucgauucccggcgccgg.........................    1
Gi3_26729320_x22.................uccggcguaguauaacggAuaga........................................................................    1
Gi3_26737284_x22.................uccggcguaguauaacUgcuaga........................................................................    1
Gi3_26717924_x22.....................................................................uGcgauucccggcgccgga........................    1
Gi3_26730772_x22...................................................................gguucgauucccUgcgccgga........................    1
Gi2_10151036_x22......................................................................uGgauucccggcgccggagc......................    1
Gi5_8238293_x22.................uccggcguaguauCacggcuag.........................................................................    1
```

```
Gi3_26740848_x22...................................................................gguCcgauucccggcgccg..........................	1
Gi1_3811083_x22................................................uucucacccaCcagauccgg............................................	1
Gi2_10142500_x22...................cggcguaguauaacggcuagaau......................................................................	0
Gi3_26721092_x22..............................................Uguucucacccaacagauccgg............................................	1
Gi3_26731828_x22...................................................................gguCcgauucccggcgccgg.........................	1
Gi1_3808399_x22................................................uucucaccUaacagauccgg............................................	1
Gi2_10139948_x22....................................................................guucgauucccggcgccggG........................	1
Gi3_26716120_x22....................................................................gCucgauucccggcgccggag.......................	1
Gi3_26719574_x22..............................................ggCucucacccaacagauccg.............................................	1
Gi3_26713986_x22................................................uuUucacccaacagauccgg............................................	1
Gi3_26726196_x22...................................................................Cguucgauucccggcgccgg.........................	1
Gi3_26722500_x22.................uccggcguaguUuaacggcuaga........................................................................	1
Gi3_26728792_x22...................................................................gguNcgauucccggcgccggagc......................	1
Gi3_26729452_x22.................uccggcguaguauaacggcuC..........................................................................	1
Gi5_8240427_x22...................................................................gguucgauNcccggcgccgg.........................	1
Gi2_10147758_x22...................................................................Cguucgauucccggcgccggag.......................	1
Gi5_8242539_x22....................ggcguaguauaacggcuagaa.......................................................................	0
Gi3_26709784_x22....................................................................guAcgauucccggcgccgg.........................	1
Gi3_26725954_x22...................................................................gguucgauucccggcgcAgga........................	1
Gi3_26717066_x22...................................................................gguucgGuucccggcgccgg.........................	1
Gi5_8243045_x22..................ccggcguaguauaacggcuagaau......................................................................	0
Gi3_26718562_x22...............................................guucucacccCacagauccg.............................................	1
Gi2_10138452_x22....................................................................guuUgauucccggcgccgg.........................	1
Gi3_26727098_x22....................................................................guucAauucccggcgccggagc......................	1
Gi1_3808971_x22.................uUcggcguaguauaacggcuag.........................................................................	1
Gi3_26728088_x22....................................................................guucgauucccggcgcAgga........................	1
Gi3_26727450_x22..................................................cGcacccaacagauccgg............................................	1
Gi3_26725008_x22.................uccggcguaguaCaacggcua..........................................................................	1
Gi5_8248083_x22..............................................gguucucacccaacagCuccg.............................................	1
Gi2_10140696_x22....................................................................guGcgauucccggcgccgg.........................	1
Gi3_26718166_x22....................................................................guAcgauucccggcgccgga........................	1
Gi3_26711566_x22....................................................................guucgauucccAgcgccggag.......................	1
Gi3_26725778_x22.................uccggcguaguauaacggGuaga........................................................................	1
Gi1_3807937_x22..............................................Uguucucacccaacagauccgg............................................	1
Gi3_26726042_x22.................uccggUguaguauaacgg.............................................................................	1
Gi5_8234575_x22...................................................................Aguucgauucccggcgcc...........................	1
Gi1_3812227_x22....................................................................guucgauucccggcgccgC.........................	1
Gi1_3810995_x22.................uccggUguaguauaacgg.............................................................................	1
G1a_5485777_x21...................................................................gUuucgauucccggcgccg..........................	1
Gi1_3818985_x21..................................................Gucacccaacagauccgg............................................	1
Gi5_8254014_x21...................................................................gguucgauucccggcgccggagA......................	1
Gi1_3820980_x21.................Gccggcguaguauaacggcuagaau......................................................................	1
G1a_5481220_x21...............................................Uuucucacccaacagauccg.............................................	1
Gi3_26764221_x21.................uccggcguaguauaaUgg.............................................................................	1
Gi5_8251242_x21.................uccggcgCaguauaacggcuaga........................................................................	1
Gi1_3813861_x21................................................uucucacccGacagauccgg............................................	1
Gi3_26754267_x21....................................................................guGcgauucccggcgccgga........................	1
```

```
Gi3_26768379_x21.................uccggcguaguauaaGggcuaga........................................................................    1
Gi3_26758068_x21.................uccggcguaguauaacAgcuag.........................................................................    1
Gi5_8251956_x21...................................................................ggAucgauucccggcgccggagc......................    1
Gi3_26768988_x21.................uccggcguaguGuaacggcuag.........................................................................    1
Gi5_8255085_x21....................................................................guucgaCucccggcgccggag.......................    1
Gi5_8262477_x21.................uccggcguaguauaacggcCagaau......................................................................    1
Gi2_10166227_x21.................uccgUcguaguauaacggcuaga........................................................................    1
Gi2_10172401_x21.................uGcggcguaguauaacgg.............................................................................    1
Gi2_10171771_x21...................................................................gguucgauucccAgcgccggag.......................    1
Gi1_3816633_x21.................uUcggcguaguauaacggcua..........................................................................    1
Gi5_8252838_x21...................................................................gguCcgauucccggcgccggagc......................    1
Gi5_8250171_x21...............................................guucucacccGacagauccg.............................................    1
Gi2_10157638_x21.................uccggcguagCauaacggcuaga........................................................................    1
Gi3_26762877_x21.................uccggcguaguaCaacggc............................................................................    1
Gi5_8257437_x21...................................................................gguucgauCcccggcgccggagc......................    1
Gi5_8253804_x21..............................................gguucucacccaacagaCccg.............................................    1
Gi2_10171645_x21.................uccggcguaguauaacggcA...........................................................................    1
Gi1_3818649_x21................................................uucucacccaacGgauccgg............................................    1
Gi3_26742633_x21.................uccggcguNguauaacggcuagaau......................................................................    1
Gi3_26769219_x21.................uccggcguGguauaacggcuagaau......................................................................    1
Gi1_3820434_x21.................uUcggcguaguauaacgg.............................................................................    1
Gi3_26765145_x21...........................................................cagauccggguucgauucccggcgccggagcC.....................    1
Gi3_26764158_x21.................uccggcgCaguauaacggcua..........................................................................    1
Gi3_26755359_x21.................uccggcguaguauaaAggcuaga........................................................................    1
Gi5_8254728_x21..............................................gAuucucacccaacagauccg.............................................    1
Gi5_8253741_x21....................................................................guucgauucccggcgccgC.........................    1
Gi2_10171897_x21....................................................................guucgauucccggcgUcggagc......................    1
Gi2_10157743_x21...................................................................gguucgauuccAggcgccggagc......................    1
Gi5_8258970_x21...................................................................ggGucgauucccggcgccggagc......................    1
Gi1_3814533_x21...................................................................gguucgauucccggcgccggaU.......................    1
Gi5_8254392_x21.............................................ugguucucacccaacagauccggguucga......................................    0
Gi2_10163098_x21...................................................................gguucgauucccggcgcUg..........................    1
Gi2_10155790_x21.................uccggcguGguauaacgg.............................................................................    1
Gi5_8255211_x21...................................................................gguucAauucccggcgccggagc......................    1
Gi3_26767035_x21.................uccggcguaguauaacCgcuaga........................................................................    1
Gi1_3820035_x21................................................uucucacccaacagauccggguucgau.....................................    0
Gi3_26755716_x21.............................................................Uauccggguucgauuccc.................................    1
Gi2_10186169_x20...................................................................gguucgauucccggcgccggaCc......................    1
Gi5_8268609_x20....................................................................guucgauucccggcgAcgg.........................    1
Gi2_10183269_x20.................Cccggcguaguauaacggc............................................................................    1
Gi3_26801825_x20....................................................................gAucgauucccggcgccgg.........................    1
Gi1_3821370_x20.................uccggcguaguaCaacgg.............................................................................    1
Gi3_26804425_x20..................................................................Ugguucgauucccggcgccggagc......................    1
Gi3_26803925_x20...................................................................gguucgauucccggcgccAgag.......................    1
Gi5_8271389_x20....................................................................guuUgauucccggcgccg..........................    1
Gi3_26799545_x20...................................................................gguucgauucccgAcgccgga........................    1
Gi5_8265869_x20.................uccggcCuaguauaacggc............................................................................    1
```

```
Gi1_3825750_x20...................cggcguaguauaacggcu............................................................................    0
Gi1_3827030_x20................................................uucucaccNaacagauccgg.............................................    1
Gi3_26786165_x20.............................................................gauccggguucgauucccggcgccgga.........................    0
Gi2_10181629_x20....................................................................guucgauCcccggcgccggagc.......................    1
Gi1_3825530_x20...................................................................ggCucgauucccggcgccgg..........................    1
Gi3_26795625_x20.................uAcggcguaguauaacggcuaga.........................................................................    1
Gi3_26802185_x20...................................................................gguucgCuucccggcgccggag........................    1
Gi2_10187089_x20...................................................................ggGucgauucccggcgccggag........................    1
Gi1_3825930_x20.................Cccggcguaguauaacgg..............................................................................    1
Gi5_8270349_x20.................Cccggcguaguauaacggc.............................................................................    1
Gi5_8265889_x20...............aguccggcguaguauaacggcu............................................................................    0
Gi1_3823770_x20...................................................................gguucgauucccggcgAcgg..........................    1
Gi1_3824130_x20.............................................ugguucucacccaacGgauccgg.............................................    1
Gi5_8276429_x20....................................................................guucAauucccggcgccgg..........................    1
Gi2_10189489_x20.................uccggcguaguauaacgAcua...........................................................................    1
Gi5_8272489_x20.................uccggcguaguauaacggUuaga.........................................................................    1
Gi2_10175769_x20...................................................................Aguucgauucccggcgccgg..........................    1
Gi3_26780245_x20.............................................ugguucucaccUaacagauc................................................    1
Gi2_10183169_x20....................................................................guucgauCcccggcgccgg..........................    1
Gi2_10185989_x20...................................................................gguucgauucccggcgccggagcU......................    1
Gi1_3821550_x20....................................................................guucgauAcccggcgccgg..........................    1
Gi5_8274529_x20..............................................gCuucucacccaacagauccg..............................................    1
Gi5_8271009_x20.................Cccggcguaguauaacggcuaga.........................................................................    1
Gi5_8276009_x20.................Cccggcguaguauaacggcua...........................................................................    1
Gi3_26772925_x20....................ggcguaguauaacggcuagaau.......................................................................    0
Gi3_26793365_x20.................uccAgcguaguauaacggcua...........................................................................    1
Gi2_10173549_x20....................................................................guucgauucccggcgccggagcA......................    1
Gi1_3827510_x20.................................................Gcucacccaacagauccg..............................................    1
Gi2_10208726_x19.................uccggcguaguauaaUggcuaga.........................................................................    1
Gi3_26830880_x19....................................................................Cuucgauucccggcgccggag........................    1
Gi3_26810854_x19................................................Cucucacccaacagauccg..............................................    1
Gi5_8291903_x19...................................................................gguucgauucccggcAccggagc.......................    1
Gi3_26808840_x19...................................................................gguucgauucccggcgccAg..........................    1
Gi2_10199340_x19....................................................................guucgauucccggcgAcgg..........................    1
Gi2_10192918_x19.................uUcggcguaguauaacggc.............................................................................    1
Gi3_26822102_x19....................................................................guucgauucccggcgAcgga.........................    1
Gi2_10196756_x19....................................................................guucgauucccggcgccgCagc.......................    1
Gi1_3833676_x19.................ucUggcguaguauaacggcuaga.........................................................................    1
Gi3_26836029_x19.................uccggcguaguauaacggcuagGau.......................................................................    1
Gi3_26808612_x19...............Gguccggcguaguauaac................................................................................    1
Gi3_26806161_x19.................uccggcguaguauaacgAc.............................................................................    1
Gi5_8280085_x19.................uccggcguagCauaacgg..............................................................................    1
Gi3_26835193_x19.................uccggcguaguaGaacggcuaga.........................................................................    1
Gi1_3831985_x19...................................................................gguucgauucccggcgcUgg..........................    1
Gi5_8287020_x19....................................................................guucgauCcccggcgccgg..........................    1
Gi5_8291884_x19.................uccggcguGguauaacggc.............................................................................    1
Gi3_26820943_x19.................uccggUguaguauaacggcuag..........................................................................    1
```

Gi3_26832894_x19..guucgauucUcggcgccgg.........................   1
Gi3_26816193_x19.................uccggcguaguauaaUggc..   1
Gi3_26805230_x19..guCcgauucccggcgccggag.......................   1
Gi1_3830807_x19...Auucucacccaacagauccg...   1
Gi1_3834930_x19..Auucgauucccggcgccggagc......................   1
Gi2_10206845_x19...gguucgGuucccggcgcc...........................   1
Gi3_26828410_x19...gguucgauucccggcgAcg..........................   1
Gi3_26820183_x19..guucgauucccgAcgccgg.........................   1
Gi3_26821722_x19..guucgauuccUggcgccgga........................   1
Gi1_3830617_x19.................uccgUcguaguauaacgg...   1
Gi5_8283296_x19..................ccggcguaguauaacggcu...   0
Gi1_3830427_x19...ucacccaacagauccggguucga......................................   0
Gi3_26819936_x19...cUauucccggcgccggag.......................   1
Gi5_8285652_x19.................uccggcguaguauaacggcuagaGu..   1
Gi2_10199530_x19..guAcgauucccggcgccggagc......................   1
Gi3_26815452_x19...gAuucgauucccggcgccg..........................   1
Gi2_10206731_x19...gguucgauuAccggcgccggagc......................   1
Gi3_26854252_x18.....................gcguaguauaacggcuaga..   0
Gi5_8302311_x18..guucgauucccggcgUcg..........................   1
Gi3_26850778_x18...gguucgauucccggcgccgCagc......................   1
Gi3_26873890_x18..uGgauucccggcgccggagc......................   1
Gi1_3838593_x18..uucucacccaacGgauccg...   1
Gi5_8303733_x18.................uccggcguaguaCaacggc..   1
Gi5_8299737_x18.................uccggUguaguauaacggcua..   1
Gi2_10224454_x18...gguuUgauucccggcgccgga........................   1
Gi1_3837495_x18..Cucucacccaacagauccg...   1
Gi5_8300925_x18.................uUcggcguaguauaacgg...   1
Gi1_3840465_x18..guucgauucccggcgccggG........................   1
Gi1_3843633_x18..uucCcacccaacagauccg...   1
Gi3_26842750_x18..guucgauucccggUgccggagc......................   1
Gi4_506352_x18...gguucgauucccggcgccggagcC.....................   1
Gi1_3843435_x18.................uccgUcguaguauaacggcuag...   1
Gi3_26859400_x18...gguucgauucccggcgccUgagc......................   1
Gi5_8297415_x18.................uccggcguaguauaacggcuCga..   1
G1a_5511635_x18...Uguucgauucccggcgcc...........................   1
Gi2_10223590_x18.................uccggcguaguauaacggcuagaaugguugg..   0
Gi2_10226272_x18.................uccggcgCaguauaacggcuaga..   1
Gi3_26870074_x18..guucgauuccUggcgccggag.......................   1
Gi5_8296209_x18..ggAucucacccaacagauccg...   1
Gi2_10215526_x18..guucgauucccggcgUcgg.........................   1
Gi3_26866942_x18..guucgauucccggAgccgg.........................   1
Gi5_8294553_x18.................uccAgcguaguauaacggcuag...   1
Gi1_3842463_x18...Gucgauucccggcgccgg.........................   1
Gi1_3841635_x18.................uccggcguaguauaacggcuGg...   1
Gi1_3842319_x18.................uccggcguNguauaacggcua..   1
Gi3_26844622_x18...uGcgauucccggcgccggagc......................   1
Gi1_3838809_x18..gUuucucacccaacagauccgg..   1

Gi2_10218010_x18...gguucgauucccggcgcUgga........................ 1
Gi3_26865556_x18.................uccggcguaguCuaacggcua.. 1
Gi3_26839870_x18..Uguucucacccaacagauccgggu.. 1
Gi2_10223176_x18..guucAauucccggcgccggagc...................... 1
Gi2_10224562_x18..guucgauucccggcgcUgg......................... 1
Gi5_8297325_x18..uCcucacccaacagauccg... 1
Gi3_26846368_x18...gguuUgauucccggcgccg.......................... 1
Gi3_26859778_x18..guucgauucccggcgcUgg......................... 1
Gi2_10218298_x18..agauccggguucgauucccggcgccggagcC..................... 1
Gi5_8296497_x18..uucucacccaCcagauccg... 1
Gi3_26843128_x18..guGcgauucccggcgccgg......................... 1
Gi1_3843075_x18...ugguCcucacccaacagauccg... 1
Gi2_10230664_x17...gguucgauucccggcgccggUgc...................... 1
Gi5_8319660_x17..gUuucucacccaacagauccgg.. 1
Gi1_3850905_x17..Auucgauucccggcgccgga........................ 1
Gi1_3851194_x17..uCcucacccaacagauccg... 1
Gi3_26878059_x17..gGuucccggcgccggagc...................... 1
Gi5_8315104_x17.................uccggcguaguauaacggcuagaaugguu.. 0
Gi1_3853115_x17.................ucUggcguaguauaacggcua.. 1
Gi2_10246083_x17..guucgauucccggcgccAgagc...................... 1
Gi2_10232534_x17...gguucgauucccggcAcc........................... 1
Gi2_10238654_x17...ggCucgauucccggcgcc........................... 1
Gi2_10238671_x17..guucgauucUcggcgccggagc...................... 1
Gi3_26898238_x17.................uccggcguagAauaacggcuaga.. 1
Gi1_3850973_x17..aUauccggguucgauuccc................................. 1
Gi1_3847335_x17...uAguucucacccaacagauccgg.. 1
Gi3_26882054_x17...gguucgauucccggGgccggagc...................... 1
Gi3_26896538_x17.................uccggcgCaguauaacggc.. 1
Gi5_8321479_x17.................uccggcguNguauaacggc.. 1
Gi3_26876937_x17.................uccggcguaguauaacggcCagaau.. 1
Gi3_26882904_x17..guucgauucccggcgccUga........................ 1
Gi3_26882003_x17...gguucgGuucccggcgccg.......................... 1
Gi2_10232636_x17.................uccggUguaguauaacggcuaga.. 1
Gi5_8319592_x17.................uccgUcguaguauaacgg... 1
Gi3_26893376_x17..guucgauucccggcgccgAa........................ 1
Gi3_26907112_x17.....................gcguaguauaacggcuagaa... 0
Gi2_10240048_x17.................uccggcguaguauaacAgc.. 1
Gi4_508099_x17..Uuucgauucccggcgccgg......................... 1
Gi1_3848661_x17..ggCucucacccaacagauccg... 1
Gi3_26889806_x17..uucucacccaacagaCccg... 1
Gi3_26909917_x17..guucgauucccggcgcAgg......................... 1
Gi3_26878246_x17...gguucgUuucccggcgccggagc...................... 1
Gi5_8308066_x17.................uccggcguaguauaaUggcuag... 1
Gi5_8311296_x17.................uccggcguaguauaGcggc.. 1
Gi1_3850395_x17...guucucacccaacagGuccg... 1
Gi5_8310820_x17..guucgauuUccggcgccgg......................... 1
Gi5_8315223_x17..gAucgauucccggcgccgg......................... 1

Gi3_26896844_x17...guucgauuUccggcgccgg........................ 1
Gi5_8310769_x17...caacagauccggguucgauucccggcgccg......................... 0
Gi1_3848746_x17...guucgaCucccggcgccggagc..................... 1
Gi3_26877923_x17...guucgauAcccggcgccgg........................ 1
Gi1_3846808_x17...guucgauuAccggcgccgg........................ 1
Gi2_10243414_x17...guucgauucUcggcgccgg........................ 1
Gi1_3847658_x17.................uccggcguaguauaacggcuaU... 1
Gi5_8308746_x17..guucucGcccaacagauccg.. 1
Gi5_8311126_x17.................uccggcguaguauaGcggcuagaau.. 1
Gi3_26903763_x17...guucgauucccggcgccggagcgA................... 1
Gi2_10247120_x17.................uccggcguUguauaacggcuaga.. 1
Gi2_10238093_x17.................uGcggcguaguauaacggcuag... 1
Gi2_10239453_x17...Auucgauucccggcgccgga....................... 1
Gi5_8313914_x17.................Gccggcguaguauaacggcu... 1
Gi5_8316906_x17.................uccggcguaguauaacggcuCg... 1
Gi3_26912263_x17...Ugguucgauucccggcgccggag...................... 1
Gi5_8319694_x17...guucgauucccggcgcUgg........................ 1
Gi4_506824_x17.................uccggcguaguauaacggc.. 0
Gi5_8318266_x17...aUauccggguucgauuccc................................ 1
Gi3_26892560_x17...gguucucacccaacagCuccg.. 1
Gi2_10229746_x17..gguucgauucccggcgAcg......................... 1
Gi1_3854747_x17..gguucgauucccggUgccgg........................ 1
Gi3_26901825_x17..gguucgauucccggAgccgga....................... 1
Gi1_3848525_x17...guucgauucccggcgcGgg........................ 1
Gi3_26935261_x16..gguucgauucccgAcgccggag...................... 1
Gi2_10257705_x16...guucgauucccggcgcUggagc..................... 1
Gi3_26916285_x16..gguucgauuccGggcgccggagc..................... 1
Gi3_26946093_x16...guucgauucccggcgccggC....................... 1
G1a_5537341_x16..gUuucgauucccggcgccgga....................... 1
Gi2_10267577_x16..Cguucgauucccggcgccg......................... 1
Gi5_8336528_x16..Aguucgauucccggcgccgg........................ 1
Gi3_26942973_x16...gguucucacccCacagauccg.. 1
Gi1_3862977_x16...guucgauuccAggcgccgg........................ 1
Gi5_8335344_x16.................uccAgcguaguauaacgg... 1
Gi3_26943453_x16..gguucgauCcccggcgccgg........................ 1
Gi2_10268377_x16...guucgauucccggcgAcggagc..................... 1
Gi3_26942173_x16.................uccAgcguaguauaacgg... 1
Gi2_10261641_x16.................uGcggcguaguauaacggcuagaau.. 1
Gi2_10269849_x16...guucgauucccggcgGcggagc..................... 1
Gi2_10261465_x16..gguucgauuccccAgcgcc......................... 1
Gi3_26926957_x16...uucucaccNaacagauccg.. 1
Gi3_26921389_x16.................uGcggcguaguauaacggcuagaa... 1
Gi1_3861857_x16..gCucucacccaacagauccg.. 1
Gi3_26931325_x16.................uccggcguaguauaacgCcuaga.. 1
Gi3_26942061_x16...gUuucucacccaacagaucc... 1
Gi3_26922189_x16...Uguucucacccaacagaucc... 1
Gi3_26936845_x16...gguucCcacccaacagauccg.. 1

Gi3_26942765_x16..gAucgauucccggcgccggagc...................... 1
Gi1_3858497_x16..gguucucacccaCcagauccg... 1
Gi5_8325104_x16.................uccggcguagCauaacggcuag... 1
Gi2_10256553_x16.................uccggcCuaguauaacggcuaga.. 1
Gi5_8323536_x16..gguucucacccaacagaucUg... 1
Gi3_26950013_x16..gguucucacccaacGgauccg... 1
Gi2_10259961_x16.................uccgUcguaguauaacggc.. 1
Gi2_10252361_x16...gguucgUuucccggcgccggagc...................... 1
Gi5_8327520_x16..guAcgauucccggcgccg.......................... 1
Gi3_26921565_x16...ggAucgauucccggcgccg.......................... 1
Gi1_3861985_x16..guucgauucAcggcgccgg......................... 1
Gi3_26919165_x16...ggCucgauucccggcgccgg......................... 1
Gi2_10256521_x16...gguucgauucccggcgccAga........................ 1
Gi5_8323808_x16..guucgauucUcggcgccg.......................... 1
Gi3_26935277_x16..gAucgauucccggcgccgga........................ 1
Gi1_3861793_x16...Uguucgauucccggcgcc........................... 1
Gi1_3861281_x16..uucucGcccaacagauccg... 1
Gi2_10263641_x16..guAcgauucccggcgccgg......................... 1
Gi1_3856657_x16..gCucgauucccggcgccgga........................ 1
Gi3_26948301_x16...gAuucgauucccggcgccgg......................... 1
Gi1_3860929_x16...guucucacccaacGgauccg... 1
Gi2_10260585_x16..gguucucaNccaacagauccg... 1
Gi5_8337504_x16...Aguucgauucccggcgccggag....................... 1
Gi5_8335312_x16.................uccggcguNguauaacgg... 1
Gi3_26920877_x16..guucgauAcccggcgccgga........................ 1
Gi5_8333760_x16...ggCucgauucccggcgccg.......................... 1
Gi3_26944653_x16.................uccggcguaguauaacggcuGgaau.. 1
Gi1_3858945_x16...gguucgauucccgAcgccg.......................... 1
Gi1_3858289_x16...gguucgGuucccggcgccgg......................... 1
Gi5_8333488_x16.................uccggcguaguauaacggcuaAa.. 1
Gi2_10263561_x16...gguucgauuccGggcgccggagc...................... 1
Gi3_26941533_x16..guucgauucUcggcgccgga........................ 1
Gi3_26945757_x16.................uccggcguaguauaGcggcuagaau.. 1
Gi2_10266009_x16.................uccggcguaguauaacggcuCga.. 1
Gi1_3857777_x16...gguucgCuucccggcgccgg......................... 1
Gi2_10267337_x16.....................................aUaaugguugguucucacc.. 1
Gi2_10258249_x16...gguucgaCucccggcgccg.......................... 1
Gi3_26930301_x16..uucucacccaacagGuccg... 1
Gi3_26919517_x16...cUgguucgauucccggcgccgga........................ 1
Gi5_8324224_x16.................uccggcguaguauaacAgc.. 1
Gi1_3855969_x16..uucucacccaacagGuccg... 1
Gi3_26925405_x16...gguucgauucccggAgccg.......................... 1
Gi3_26932605_x16...gguucgauucccggcgccggaA....................... 1
Gi5_8331232_x16..Auucgauucccggcgccgga........................ 1
Gi3_26947453_x16.................uccggcguCguauaacgg... 1
Gi5_8335584_x16..guucgauucccggUgccgg......................... 1
Gi1_3862593_x16...ugguucucacccaCcagauccgg.. 1

```
Gi1_3860049_x16................................................uCcucacccaacagauccgg............................................ 1
Gi1_3857521_x16.................uccggcguGguauaacgg............................................................................. 1
Gi2_10275640_x15....................................................................gCucgauucccggcgccg.......................... 1
Gi3_26969215_x15....................................................................guAcgauucccggcgccggagc...................... 1
Gi2_10287145_x15....................................................................guucgauucccggcgccgA......................... 1
Gi5_8346857_x15...................................................................gguucgauNcccggcgcc........................... 1
Gi3_26981320_x15...................................................................ggCucgauucccggcgcc........................... 1
Gi1_3867872_x15...............................................guucucacNcaacagauccgg............................................ 1
Gi2_10279780_x15.................ucUggcguaguauaacggcua.......................................................................... 1
Gi5_8350472_x15.................uUcggcguaguauaacggcua.......................................................................... 1
Gi2_10287895_x15....................................................................guucAauucccggcgccgg......................... 1
Gi2_10273030_x15....................................................................guucgGuucccggcgccgga........................ 1
G1a_5544824_x15....................................................................Uuucgauucccggcgccggagc...................... 1
Gi3_26960050_x15....................................................................guucgauuAccggcgccggagc...................... 1
Gi1_3872252_x15..............................................gguucucacccGacagauccg............................................. 1
Gi5_8338427_x15................................................uucucacccaacGgauccg............................................. 1
Gi5_8344892_x15.................................................ucucacccGacagauccgg............................................ 1
Gi3_26955550_x15.................Gccggcguaguauaacggcu........................................................................... 1
Gi5_8345297_x15..............................................ggGucucacccaacagauccg............................................. 1
Gi5_8351162_x15..................................................cCcacccaacagauccgg............................................ 1
Gi1_3866552_x15.............................................uggCucucacccaacagauccgg............................................ 1
Gi3_26975290_x15....................................................................guucgauucUcggcgccggagc...................... 1
Gi3_26954470_x15...................................................................gguucgauuGccggcgccggagc...................... 1
Gi3_26968030_x15................................................uucucacccaacGgauccg............................................. 1
Gi3_26988265_x15..............................................gguucucacccGacagauccg............................................. 1
Gi2_10281595_x15.................uccAgcguaguauaacggcuaga........................................................................ 1
G1a_5552324_x15...................................................................gUuucgauucccggcgcc........................... 1
Gi1_3869582_x15.................uccggcguaguauaacggcuG.......................................................................... 1
Gi1_3870362_x15...................................................................ggGucgauucccggcgccgg......................... 1
Gi3_26977105_x15...........................................................cagauccggguucgauucccggcgccggagc...................... 0
Gi3_26983810_x15...................................................................gguucgauucccUgcgccggag....................... 1
Gi5_8341367_x15.................uccggcguagCauaacggc............................................................................ 1
Gi2_10292530_x15....................................................................guucgauucccggcAccggagc...................... 1
Gi3_26953060_x15....................................................................guucUauucccggcgccggagc...................... 1
Gi2_10288225_x15.............................................uUguucucacccaacagaucc.............................................. 1
Gi2_10281325_x15...................................................................gAuucgauucccggcgccggag....................... 1
Gi2_10291690_x15...................................................................ggCucgauucccggcgccggag....................... 1
Gi3_26957575_x15....................................................................Auucgauucccggcgccg.......................... 1
Gi1_3870467_x15.................uccggcguaguauaacAg............................................................................. 1
Gi3_26977270_x15..............................................gguucucacUcaacagauccg............................................. 1
Gi3_26953450_x15....................................................................guucgauucccggcgccCg......................... 1
Gi5_8339057_x15...................................................................gguucgauucccAgcgccgga........................ 1
Gi3_26978710_x15..........................................................acagauccggguucgauucccggcgccgga........................ 0
Gi3_26988595_x15...............................................guucucacNcaacagauccg............................................. 1
Gi3_26961715_x15.................uccAgcguaguauaacggc............................................................................ 1
Gi4_510572_x15.................uccggcguaguauaacggcuagaau...................................................................... 0
Gi1_3865502_x15.................uccggcgCaguauaacgg............................................................................. 1
```

```
Gi5_8351762_x15.............................................gUuucucacccaacagauc...............................................    1
Gi3_26985805_x15...................................................................guucgauuUccggcgccggag.......................    1
Gi5_8352632_x15.................uccggcguaguauCacgg............................................................................    1
Gi2_10291720_x15...................................................................guucgauAcccggcgccggagc......................    1
Gi1_3870317_x15..................................................................gCuucgauucccggcgccgg.........................    1
Gi1_3868877_x15.................uccggcguaguauaacgAcuag........................................................................    1
Gi1_3867737_x15.............................................Cguucucacccaacagauccg.............................................    1
Gi3_26972620_x15..................................................................gguucgauuccAggcgccgga........................    1
Gi3_26986555_x15............................................Ggguucucacccaacagauc...............................................    1
Gi5_8349707_x15...................................................................guucgauucccggAgccgg.........................    1
Gi1_3872102_x15.................uccggcguaguGuaacggcuag........................................................................    1
Gi3_26987620_x15.................uccggcguaguauaacgAcuagaau.....................................................................    1
Gi3_26992975_x15.................uUcggcguaguauaacggcuagaau.....................................................................    1
Gi5_8338577_x15...................................................................guucgauucccAgcgccgg.........................    1
Gi5_8354912_x15..................................................................gguucgauucccggcgcUggagc......................    1
Gi2_10293895_x15..................................................................gguucgGuucccggcgccggag.......................    1
Gi3_26957860_x15...............................................uucCcacccaacagauccg.............................................    1
Gi2_10277185_x15.................uccggcguaguaCaacggcuaga.......................................................................    1
Gi2_10274605_x15...................................................................guCcgauucccggcgccg..........................    1
Gi2_10284595_x15...................................................................guucgauucccggUgccgg.........................    1
Gi3_26966755_x15..................................................................gCuucgauucccggcgccg..........................    1
Gi2_10291435_x15............................................ugguucucNcccaacagauccg.............................................    1
Gi2_10294195_x15...............................................uucucacccaCcagauccg.............................................    1
Gi5_8340467_x15...................................................................guucgauucccggcgcccCg........................    1
Gi3_26986465_x15...................................................................guucgauucccggcgcAggagc......................    1
Gi5_8344007_x15..................................................................gguucgauucccggUgccggagc......................    1
Gi2_10287565_x15...................................................................guGcgauucccggcgccggagc......................    1
Gi3_26966170_x15.................uccggcguaguGuaacggcuagaau.....................................................................    1
Gi3_26952685_x15..................................................................gguucgauuUccggcgccgga........................    1
Gi1_3867422_x15.................uccggcguaguauaacggcC..........................................................................    1
Gi5_8359135_x14..................................................................ggCucgauucccggcgccgga........................    1
Gi3_27009573_x14...................................................................guucgauucccggcgAcggagc......................    1
Gi1_3873118_x14.............................................gguucucacccaacagaCccg.............................................    1
Gi3_27012527_x14.................uccggcguaguauaCcggcua.........................................................................    1
Gi5_8360829_x14..................................................................gguucgauucccUgcgccggagc......................    1
Gi5_8363755_x14..................................................................gguucAauucccggcgccg..........................    1
Gi2_10313039_x14...................................................................guucgauucccggcgcUg..........................    1
Gi3_27007347_x14.................Cccggcguaguauaacggcuagaau.....................................................................    1
Gi5_8359835_x14.................uccggcguaguauaacAgcuagaau.....................................................................    1
Gi3_26998135_x14...............................................uucucaccNaacagauccgg............................................    1
Gi2_10316595_x14..................................................................gAuucgauucccggcgcc...........................    1
Gi5_8368683_x14.................uccgUcguaguauaacggcua.........................................................................    1
Gi5_8365309_x14......................cguaguauaacggcuagaaugguugguuc............................................................    0
Gi5_8363433_x14...................................................................Cuucgauucccggcgccgga........................    1
Gi3_27011939_x14...............................................uucucacccaGcagauccg.............................................    1
Gi3_27000277_x14....................................................................uucgauucccggcgccggCgc......................    1
Gi3_27005163_x14...................................................................guucgauucccggcgccgAagc......................    1
```

```
Gi3_27016139_x14...................................................................gguuGgauucccggcgccgga........................    1
Gi5_8363685_x14...................................................................gguucgauucccggcgccggagU......................    1
Gi3_26999759_x14...................................................................gguucgauucccggUgccg..........................    1
Gi3_27004211_x14...................................................................gguucgauucccggcgUcggag.......................    1
Gi5_8356405_x14...................................................................gguucgauucccAgcgccg..........................    1
Gi3_27036425_x14.............................................ugguucucacccaacagauccggguucga......................................    0
Gi3_27012821_x14....................................................................gCucgauucccggcgccg..........................    1
Gi1_3882092_x14...............................................guucucacccaacagGuccgg............................................    1
Gi3_27000333_x14...................................................................gguucgauucccggcgccCgagc......................    1
Gi3_27002125_x14...................................................................gguucgauucccggcgAcgg.........................    1
Gi2_10308545_x14.................uccggUguaguauaacggcua..........................................................................    1
Gi1_3875372_x14...................................................................gguucgauucccggcAccgg.........................    1
Gi5_8368865_x14...................................................................ggCucgauucccggcgcc...........................    1
Gi5_8371357_x14.................uccggcguaguauaGcggcua..........................................................................    1
Gi5_8366779_x14.................uccggcguaguaCaacggcuag.........................................................................    1
Gi2_10313193_x14....................................................................guucgauucccggcgccA..........................    1
Gi2_10304737_x14.................uccggcguaguauaacggcuagGau......................................................................    1
Gi1_3877304_x14................................................uucucacccaacagGuccg.............................................    1
Gi3_27021501_x14....................................................................guGcgauucccggcgccggag.......................    1
Gi2_10301433_x14.................uccggcguaguGuaacggc............................................................................    1
G1a_5558613_x14................................................Gucucacccaacagauccg.............................................    1
Gi3_27036271_x14.................uccggcguaguauaacggcuaUa........................................................................    1
Gi1_3873146_x14................................................uucucGcccaacagauccgg............................................    1
Gi3_27008271_x14...............................................guucucacccaacagauccggguucgau.....................................    0
Gi1_3878970_x14.............................................ugguucucacccCacagauccgg............................................    1
Gi3_27018169_x14....................................................................guuUgauucccggcgccggagc......................    1
Gi2_10308713_x14.................ucUggcguaguauaacggcuagaau......................................................................    1
Gi2_10316175_x14....................................................................guucgauucccggcgccgAagc......................    1
Gi5_8362635_x14...................................................................gguucgauucccggcgccggGg.......................    1
Gi3_27023615_x14................................................uucucGcccaacagauccg.............................................    1
Gi1_3877780_x14...................................................................ggCucgauucccggcgccggagc......................    1
Gi5_8358995_x14....................................................................gCucgauucccggcgccgga........................    1
Gi3_27010399_x14...................................................................ggNucgauucccggcgccggagc......................    1
Gi1_3876772_x14..............................................gguucucacccaacagCuccg.............................................    1
Gi2_10296631_x14....................................................................guucgauuNccggcgccg..........................    1
G1a_5559747_x14.................uccggcguaguauaacgAcuagaau......................................................................    1
Gi3_27021445_x14.................uccggcguaguauaGcggcuagaa.......................................................................    1
Gi3_27015145_x14...................................................................gguucgauAcccggcgccggag.......................    1
Gi2_10305815_x14.....................gcguaguauaacggcuagaa.......................................................................    0
Gi5_8362173_x14.................uccggcguaguauaacggcCa..........................................................................    1
Gi5_8369299_x14.................Cccggcguaguauaacgg.............................................................................    1
Gi1_3874140_x14................................................Cucucacccaacagauccgg............................................    1
Gi2_10297023_x14...................................................................gguucgauCcccggcgccgga........................    1
Gi5_8367703_x14...............................................guucucacccaacCgauccg.............................................    1
Gi5_8365687_x14.................uGcggcguaguauaacggcuagaa.......................................................................    1
Gi5_8372561_x14....................................................................guGcgauucccggcgccgg.........................    1
Gi3_27006577_x14................................................uCcucacccaacagauccgg............................................    1
```

```
Gi3_27037237_x14..............................................gguucucacccaCcagauccg.............................................    1
Gi3_27005429_x14...................................................................gCuucgauucccggcgccgg.........................    1
Gi3_27000123_x14...................................................................gguucgauucccggcgccgAa........................    1
Gi3_27029817_x14.................uccggcgGaguauaacggcuaga........................................................................    1
Gi5_8359107_x14.................uccggcgCaguauaacggcuagaau......................................................................    1
Gi5_8363377_x14...................................................................gguucgCuucccggcgccg..........................    1
Gi5_8365253_x14...................................................................Cguucgauucccggcgccgga........................    1
Gi3_27002881_x14.................uccNgcguaguauaacggcuaga........................................................................    1
Gi3_27014207_x14.................uccggcguUguauaacggc............................................................................    1
Gi2_10303701_x14.................uccggcguGguauaacggcuag.........................................................................    1
Gi2_10306039_x14....................................................................guucgauucccggAgccggagc......................    1
Gi3_27005527_x14.................uccUgcguaguauaacggcuaga........................................................................    1
Gi3_27019345_x14....................................................................guucgauuccAggcgccgg.........................    1
Gi5_8360045_x14...................................................................gAuucgauucccggcgccg..........................    1
Gi3_27024385_x14.................uccggcguaguauaCcgg.............................................................................    1
Gi1_3880440_x14.............................................ugguucucacccaacagaCccg.............................................    1
Gi3_27022649_x14....................................................................guucgauucccggcgGcggagc......................    1
Gi3_27006661_x14...............................................guCcucacccaacagauccg.............................................    1
Gi3_27001719_x14...................................................................gAuucgauucccggcgcc...........................    1
Gi3_27029341_x14.....................................................................uucgauucccggcgccgC.........................    1
Gi3_26999605_x14...................................................................gguucgauuccUggcgccgg.........................    1
Gi2_10298059_x14....................................................................guucgauuUccggcgccgg.........................    1
Gi3_27015621_x14....................................................................guGcgauucccggcgccggagc......................    1
Gi3_27039015_x13...................................................................gguucgauuUccggcgccgg.........................    1
Gi3_27075623_x13...............Gguccggcguaguauaacggcuagaau......................................................................    1
Gi1_3886073_x13.................Gccggcguaguauaacggcu...........................................................................    1
Gi3_27084086_x13..............................................gguucucacccaacagaucUg.............................................    1
Gi3_27063494_x13......................................................................uGgauucccggcgccggag.......................    1
Gi1_3882914_x13...............................................guucucGcccaacagauccg.............................................    1
Gi5_8373884_x13...................................................................gguucgauucccggcgccAgagc......................    1
Gi1_3885072_x13.................uccggcguaguauaacggcuagU........................................................................    1
Gi3_27044670_x13....................................................................guucgauuUccggcgccggagc......................    1
Gi5_8373143_x13.................uccggcguaguauaacggcuagGau......................................................................    1
Gi3_27064742_x13..................................................Gucacccaacagauccgg............................................    1
Gi3_27038209_x13.................uccggcguaguauaacggcuaAaau......................................................................    1
Gi1_3889024_x13.............................................ugguucucacccGacagauccgg............................................    1
Gi2_10338106_x13...................................................................gCuucgauucccggcgccggag.......................    1
Gi3_27065496_x13..............................................gUuucucacccaacagauccgg............................................    1
Gi5_8380410_x13....................................................................guucgauucccggcgGcgg.........................    1
Gi2_10332919_x13............................................................aUauccggguucgauucc..................................    1
Gi4_514174_x13...................................................................Uguucgauucccggcgccggagc......................    1
Gi3_27065327_x13....................................................................guucgGuucccggcgccg..........................    1
Gi2_10338912_x13....................................................................guucgaGucccggcgccggagc......................    1
Gi1_3891182_x13...............................................guucucacccGacagauccgg............................................    1
Gi1_3887464_x13.............................................ugguucCcacccaacagauccgg............................................    1
Gi3_27072802_x13...................................................................gguucgauucccCgcgccggagc......................    1
Gi5_8373910_x13...................................................................ggGucgauucccggcgccg..........................    1
```

```
Gi5_8373351_x13...................................................................gguucgauucccggcgccggG........................ 1
Gi3_27069760_x13.................uccggcguaguauaCcggc............................................................................ 1
Gi3_27067030_x13...................................................................gguuAgauucccggcgccgga........................ 1
Gi1_3891559_x13....................Ugcguaguauaacggcuag......................................................................... 1
Gi3_27043669_x13....................................................................guucgauuccAggcgccgga........................ 1
Gi2_10321648_x13....................................................................guucgauucccggcgccggagU...................... 1
Gi1_3892261_x13....................................................................guucgauucccggcgccggU........................ 1
Gi2_10322532_x13...................................................................gguucgauucccggcgccggagcgA.................... 1
Gi3_27067238_x13.................uccggcguaguauaacgUcuaga........................................................................ 1
Gi3_27052301_x13...................................................................gguucgauucccggcgcAggag....................... 1
Gi3_27046542_x13...................................................................gguuGgauucccggcgccggag....................... 1
Gi3_27062818_x13...................................................................gguucgauuccUggcgcc........................... 1
Gi5_8389601_x13...................................................................gguucgaCucccggcgccg.......................... 1
Gi3_27047153_x13.................uccggcAuaguauaacggcua.......................................................................... 1
Gi2_10322298_x13...................................................................gguucgauucccggcAccgga........................ 1
G1a_5572419_x13...................................................................gUucgauucccggcgccgg.......................... 1
Gi3_27060023_x13.................uccggcguaguauaacggcuagaC....................................................................... 1
Gi5_8382022_x13....................................................................guucgauucccggcgccggCgc...................... 1
Gi2_10336663_x13................................................Cucucacccaacagauccg............................................. 1
Gi2_10330592_x13....................................................................guucgauucccggcgccggaAc...................... 1
Gi5_8385987_x13.................uccggcguaguauaacggcuagaaC...................................................................... 1
Gi3_27056617_x13.................uccggcguaguauaacggAua.......................................................................... 1
Gi1_3891845_x13...................................................................gguucgauNcccggcgccggagc...................... 1
Gi1_3888413_x13.............................................ugUuucucacccaacagauccg............................................. 1
Gi3_27079484_x13..............................................ggGucucacccaacagauccg............................................. 1
Gi5_8376679_x13....................................................................guucgauuNccggcgccgga........................ 1
Gi3_27077092_x13...................................................................gguucgauucccggcgccggaUc...................... 1
Gi3_27050338_x13....................................................................guucgauucccggcgccggagA...................... 1
Gi1_3892781_x13..............................................gguucucaNccaacagauccg............................................. 1
Gi2_10322402_x13....................................................................guucgauucccggcgccggagcU..................... 1
Gi2_10336390_x13...................................................................gguucgauucccggcgccgCagc...................... 1
Gi2_10328811_x13...................................................................ggCucgauucccggcgccg.......................... 1
Gi3_27059763_x13.................uccggcguaguauaacggU............................................................................ 1
Gi3_27075558_x13.................uccggcguCguauaacggc............................................................................ 1
Gi3_27058840_x13.................uccggcguaguauaacggcCag......................................................................... 1
Gi3_27057319_x13....................................................................guucgCuucccggcgccggagc...................... 1
Gi3_27063065_x13...................................................................gguucgauucccggcgccgA......................... 1
Gi3_27048310_x13...................................................................gguucgauuccUggcgccg.......................... 1
Gi1_3885592_x13....................................................................guucgauucccggcgGcgg......................... 1
Gi3_27059906_x13...................................................................gguucUauucccggcgccggagc...................... 1
Gi2_10324222_x13...................................................................gguucgauucccggcgccAgag....................... 1
Gi3_27042083_x13...................................................................Cguucgauucccggcgcc........................... 1
Gi2_10342500_x13.................uccggcguaguGuaacggcuagaau...................................................................... 1
Gi1_3885293_x13...............................................guucucacccaCcagauccg............................................. 1
Gi3_27045437_x13....................................................................guucgauucccggcgccAgagc...................... 1
Gi2_10324599_x13.................uccggcguagCauaacggcua.......................................................................... 1
Gi1_3884643_x13....................................................................guucgauucccAgcgccgga........................ 1
```

```
Gi2_10328044_x13.................uccggcguaguauaacAgcuagaau......................................................................   1
Gi5_8380748_x13................................................uucucacccaacagGuccg.............................................   1
Gi5_8373858_x13...................................................................Cguucgauucccggcgccgg.........................   1
G1a_5576280_x13.................uccggcguaguauaacggcu...........................................................................   0
Gi3_27043162_x13...................................................................gguucgauucccggcgcGggagc......................   1
Gi3_27052756_x13...................................................................gguucgauucccggcgUcg..........................   1
Gi3_27051573_x13.................uccgUcguaguauaacggcuag.........................................................................   1
Gi3_27062961_x13....................................................................guucgauCcccggcgccggag.......................   1
Gi3_27059126_x13..............................................gCuucucacccaacagauccg.............................................   1
Gi1_3885540_x13....................................................................guuNgauucccggcgccgg.........................   1
Gi2_10333738_x13.................uccggcguaguGuaacggcua..........................................................................   1
Gi3_27077807_x13...............................................Auucucacccaacagauccg.............................................   1
Gi3_27063572_x13..............................................gguucucGcccaacagauccg.............................................   1
Gi5_8376926_x13...................................................................gguucgauuUccggcgccg..........................   1
Gi5_8385636_x13....................................................................guAcgauucccggcgccgg.........................   1
Gi5_8383855_x13....................................................................guucgauucUcggcgccgg.........................   1
Gi5_8384674_x13...................................................................gguucgauucccggcgAcggagc......................   1
Gi5_8382412_x13..............................................gguucucaccUaacagauccg.............................................   1
Gi2_10332256_x13...................................................................gguAcgauucccggcgccggagc......................   1
Gi5_8381086_x13...............................................guucCcacccaacagauccg.............................................   1
Gi4_513719_x13....................................................................guucgauucccggcgccg..........................   0
Gi5_8390368_x13....................................................................guucgauucccgUcgccgg.........................   1
Gi2_10367230_x12....................................................................guucgauucccAgcgccgg.........................   1
Gi3_27097293_x12..............................................gguucucacccaacagaCccg.............................................   1
Gi2_10356154_x12...................................................................ggGucgauucccggcgccgga........................   1
Gi5_8399538_x12.................Accggcguaguauaacggcuag.........................................................................   1
Gi1_3902258_x12..............................................gguucucGcccaacagauccg.............................................   1
Gi2_10359070_x12....................................................................Cuucgauucccggcgccggag.......................   1
Gi3_27124785_x12...................................................................gguucgauucccggUgccgg.........................   1
Gi3_27096909_x12....................................................................guucgauucccggcgGcgg.........................   1
Gi1_3896822_x12.................uccggcguaguGuaacgg.............................................................................   1
Gi5_8406918_x12.................uccggcguaguauaacggcuG..........................................................................   1
Gi3_27106473_x12.................Accggcguaguauaacggc............................................................................   1
Gi3_27088305_x12...................................................................gguucgauucccggcgcUgg.........................   1
Gi3_27092649_x12.................uccggcAuaguauaacgg.............................................................................   1
Gi5_8401914_x12....................................................................Uuucgauucccggcgccggag.......................   1
Gi5_8404434_x12....................................................................gCucgauucccggcgccggagc......................   1
Gi2_10361794_x12..............................................Aguucucacccaacagauccg.............................................   1
Gi5_8400042_x12...................................................................gAuucgauucccggcgccgga........................   1
Gi5_8407074_x12...................................................................gguucgauucccggcgcAggagc......................   1
Gi2_10344394_x12....................................................................guucgauucccggcAccgga........................   1
Gi2_10349386_x12....................................................................gAucgauucccggcgccggagc......................   1
Gi5_8393478_x12...................................................................gguucgCuucccggcgccggagc......................   1
Gi2_10364326_x12...................................................................gguucgauucccggGgccggagc......................   1
Gi1_3900650_x12....................................................................guucgauucccCgcgccgg.........................   1
Gi2_10359730_x12................................................uucucacccaacagGuccg.............................................   1
Gi3_27117369_x12...................................................................gguucgauucccggcgcAg..........................   1
```

```
Gi3_27094065_x12.................uccggcguaguCuaacggc............................................................................    1
Gi1_3899774_x12.....................................................................uGcgauucccggcgccgg.........................    1
Gi5_8401218_x12...................................................................gUuucgauucccggcgccggagcgu....................    1
Gi2_10353190_x12...................................................................gguucgauuccUggcgcc...........................    1
Gi3_27112941_x12...................................................................gguucgaAucccggcgccggagc......................    1
Gi2_10345018_x12....................................................................guucgauuNccggcgccggag.......................    1
Gi1_3897482_x12....................................................................guucgaGucccggcgccgg.........................    1
Gi5_8395722_x12...................................................................gguucgauucccggcgcUgga........................    1
Gi1_3896510_x12.................uccggUguaguauaacggcua..........................................................................    1
Gi4_515028_x12.................uccggcguaguauaacggcuagaa.......................................................................    0
Gi5_8393466_x12..............................................gguucucacccaacagaucAg.............................................    1
Gi5_8401998_x12...............................................guucucaccUaacagauccg.............................................    1
Gi5_8401710_x12.................................................uGucacccaacagauccg.............................................    1
Gi2_10356202_x12........................................................................gGuucccggcgccggagc......................    1
Gi5_8400198_x12.................uccggcgCaguauaacggcuag.........................................................................    1
Gi3_27101517_x12..............................................gguucucacccaacagGuccg.............................................    1
Gi3_27104649_x12................................................uucCcacccaacagauccgg............................................    1
Gi3_27110877_x12....................................................................guucgauucccggcgccAgag.......................    1
Gi1_3898454_x12...............................................guucucacccaacGgauccgg............................................    1
Gi3_27122361_x12...................................................................gguucgauuUccggcgccg..........................    1
Gi3_27126693_x12....................................................................guucgauucccCgcgccggagc......................    1
Gi3_27131349_x12....................................................................guucgauuUccggcgccgga........................    1
Gi3_27103185_x12.................uccggcguaguauaacAgcuagaa.......................................................................    1
Gi2_10363918_x12...................................................................gguucgCuucccggcgccggag.......................    1
Gi3_27118293_x12....................................................................guucUauucccggcgccggag.......................    1
Gi1_3901406_x12.................uccggcguaguauaacggU............................................................................    1
Gi3_27117957_x12...............................................guucucacccaacCgauccg.............................................    1
Gi5_8396058_x12....................ggcguaguauaacggcua..........................................................................    0
Gi3_27126453_x12..............................................gUuucucacccaacagauccgggu..........................................    1
Gi3_27109329_x12.................uccggcguaguauaaAggcua..........................................................................    1
Gi5_8394810_x12....................................................................guucgauucccgAcgccgg.........................    1
Gi3_27102801_x12.................uccggcguUguauaacggcua..........................................................................    1
Gi2_10347886_x12.................uGcggcguaguauaacggcuagaa.......................................................................    1
Gi1_3894194_x12.............................................uCguucucacccaacagauccgg............................................    1
Gi2_10344010_x12....................................................................guuUgauucccggcgccg..........................    1
Gi5_8406114_x12............................................Gugguucucacccaacagauccg.............................................    1
Gi3_27101049_x12.............................................ugguucucNcccaacagauccg.............................................    1
Gi5_8404902_x12...................................................................gguucgauucccggcgccggCg.......................    1
Gi5_8404566_x12...................................................................gguucgaCucccggcgccgg.........................    1
Gi3_27113469_x12......................................................................uUgauucccggcgccggagc......................    1
Gi2_10365874_x12...................................................................gguNcgauucccggcgccggagc......................    1
Gi1_3895478_x12.................uccggcguaguauaacggcuagaaA......................................................................    1
Gi5_8402034_x12....................................................................guucgauucccggcgcAgg.........................    1
Gi1_3898814_x12.................uccggcguaguauaaUggcuaga........................................................................    1
Gi3_27087333_x12........................................................caacagauccggguucgauucccggcgccgg.........................    0
Gi1_3897170_x12................................................uucCcacccaacagauccgg............................................    1
Gi1_3895370_x12...................................................................gguucgauucccAgcgccgg.........................    1
```

```
Gi1_3895874_x12................................................uucucacccaacaAauccgg.............................................     1
Gi5_8400306_x12....................................................................guucgauucccggcgAcg...........................     1
Gi2_10354426_x12...................................................................gguucgGuucccggcgccgga.........................     1
Gi3_27108045_x12................................................uucucacccaGcagauccgg.............................................     1
Gi5_8392722_x12...............................................guucucacccaacagaCccg..............................................     1
Gi3_27103977_x12............................................Gugguucucacccaacagaucc...............................................     1
Gi5_8408574_x12.................uUcggcguaguauaacggc.............................................................................     1
Gi5_8406570_x12.................uccggcguaguGuaacggcuagaau.......................................................................     1
Gi5_8392854_x12.................uccggcguaguauCacggcua...........................................................................     1
Gi3_27117285_x12...................................................................gguucgauucAcggcgccggag........................     1
Gi3_27105573_x12.................uccggcguaguauaacggcuagCa........................................................................     1
Gi3_27093009_x12...................................................................gguucgauucccggcgccggU.........................     1
Gi5_8401206_x12.................uccggcguaguaCaacggcua...........................................................................     1
Gi5_8394774_x12....................................................................guucgauucccggcgcAg...........................     1
Gi1_3897854_x12....................................................................guucgauucccUgcgccgg..........................     1
Gi3_27097029_x12...................................................................gguucgauucccggcAcc............................     1
Gi2_10348270_x12................................................uCcucacccaacagauccg..............................................     1
Gi5_8392002_x12.................uccggUguaguauaacgg..............................................................................     1
Gi2_10354906_x12....................................................................guucgauucccAgcgccg...........................     1
Gi5_8404698_x12................................................uucCcacccaacagauccg..............................................     1
Gi1_3900710_x12.................uccggcguaguauaacAgcuaga.........................................................................     1
Gi3_27113829_x12.................Accggcguaguauaacggcua...........................................................................     1
Gi1_3902114_x12.................uccggcguaguauaGcggcua...........................................................................     1
Gi3_27123429_x12....................................................................guucgauucccggcgccggagU.......................     1
Gi2_10350382_x12....................................................................Auucgauucccggcgccg...........................     1
Gi5_8400258_x12.................uccggcguaguauaacgAc.............................................................................     1
Gi5_8403234_x12...................................................................gguucgauucccggcgUcggagc.......................     1
Gi1_3899882_x12..................ccggcguGguauaacggcuag..........................................................................     1
Gi3_27127785_x12....................................................................guucgauucccggcgccggagcU......................     1
Gi5_8407494_x12....................................................................guucgauucccggcgUcggagc.......................     1
Gi5_8404746_x12.................uccggUguaguauaacggcuaga.........................................................................     1
Gi2_10355794_x12.................uccggcguaguauaacggcuagGa........................................................................     1
Gi1_3893738_x12...............................................guucucacccaacagauccAg.............................................     1
Gi3_27148716_x11.................uccggUguaguauaacggcuagaa........................................................................     1
Gi5_8412951_x11...............................................guucucacccaacagauccA..............................................     1
Gi3_27147528_x11.................uccggGguaguauaacggcuaga.........................................................................     1
Gi3_27173730_x11....................................................................guucgauucccggcgccgU..........................     1
Gi5_8429264_x11....................................................................guucgauuUccggcgccg...........................     1
Gi5_8425271_x11..............................................gguucucaUccaacagauccg..............................................     1
Gi5_8419232_x11..................................................cucacccaacagGuccgg.............................................     1
Gi5_8429451_x11.................uUcggcguaguauaacggcuagaa........................................................................     1
Gi3_27179087_x11.................uccggcguaguauCacggcuag..........................................................................     1
Gi2_10395388_x11................................................uuUucacccaacagauccg..............................................     1
Gi1_3910568_x11.............................................ugguucucacccaacagGuccgg.............................................     1
Gi3_27164699_x11...................................................................gguucgauAcccggcgccgga.........................     1
Gi2_10376468_x11....................................................................guucgauuccUggcgccggag........................     1
G1a_5613319_x11....................................................................Uuucgauucccggcgccgg..........................     1
```

Gi1_3909710_x11...Aguucgauucccggcgccggagc...................... 1
Gi2_10392935_x11...gguucgaAucccggcgccggagc...................... 1
Gi3_27183982_x11..guucgauucccggAgccggagc...................... 1
Gi1_3907873_x11..guucgauucccggcgcUgga........................ 1
Gi2_10393540_x11...gguucgauucccggcgUc........................... 1
Gi3_27173664_x11.................Accggcguaguauaacgg... 1
Gi1_3908324_x11..guucgauucccggcgccggagcU..................... 1
Gi1_3905453_x11...Aguucgauucccggcgccgga........................ 1
Gi2_10397588_x11...gguucgauCcccggcgcc........................... 1
Gi3_27137221_x11...Uuucucacccaacagauccgggu.. 1
Gi3_27162972_x11...Gccggguucgauucccggcgccgga........................ 1
Gi3_27169121_x11...gguucgauucccAgcgccgg......................... 1
Gi3_27157802_x11...ugguucucacccaacGgauccg... 1
Gi2_10389547_x11..................ccggcguaguauaacggcuagaau.. 0
Gi3_27145449_x11..gguucucacccaacagauccA... 1
Gi5_8420024_x11...gguucgauucccggcgccA.......................... 1
Gi2_10392671_x11..ggCucucacccaacagauccg... 1
Gi1_3915661_x11.................uccggcguagCauaacgg... 1
Gi2_10388832_x11...ggAucgauucccggcgcc........................... 1
Gi1_3909963_x11..guucgauucccggcgccAga........................ 1
Gi5_8420563_x11.................uccggcguaguaCaacgg... 1
Gi3_27177382_x11..guucgauuNccggcgccg.......................... 1
Gi3_27183586_x11..guucgauucccggcAccggag....................... 1
Gi5_8413479_x11....................gUcguaguauaacggcuag... 1
Gi5_8431057_x11..guucgauucAcggcgccg.......................... 1
Gi5_8418451_x11.................uccggUguaguauaacggc.. 1
Gi3_27138805_x11...ggGucgauucccggcgcc........................... 1
Gi3_27143898_x11.................uccggcguaguaAaacggcuaga.. 1
Gi5_8418330_x11...guGcucacccaacagauccg... 1
Gi5_8410553_x11.................Accggcguaguauaacggcuaga.. 1
Gi3_27178779_x11...gguucgauuAccggcgccggag....................... 1
Gi5_8424138_x11...Gcucacccaacagauccgg.. 1
Gi3_27157648_x11...uGcgauucccggcgccgg......................... 1
Gi3_27167482_x11...gguucgauucccAgcgccg.......................... 1
Gi2_10395916_x11..guucgauucccggcgccggagcgA.................... 1
Gi2_10379944_x11...ggGucgauucccggcgcc........................... 1
Gi3_27167427_x11..guucgauucccgUcgccgga........................ 1
Gi2_10391230_x11...................cUgcguaguauaacggcua.. 1
Gi3_27150520_x11.................uccggcguaguauGacggc.. 1
Gi2_10394222_x11...gguucgaCucccggcgccgga........................ 1
Gi5_8428120_x11...Gcacccaacagauccggguu... 1
Gi2_10391307_x11..Cuucgauucccggcgccg.......................... 1
Gi1_3907752_x11..guucAauucccggcgccgga........................ 1
Gi5_8414458_x11...gguucgauuUccggcgccggagc...................... 1
Gi2_10384421_x11.................uUcggcguaguauaacgg... 1
Gi3_27156955_x11..cucacccaacagauccggguucgauuccc................................. 0
Gi5_8420310_x11..guucgauucccggcgccgA......................... 1

```
Gi2_10369868_x11......................................................................ucgauuccNggcgccggagc......................    1
Gi3_27151818_x11.................uccggcguaguauaacggcuagGa.......................................................................    1
Gi1_3909952_x15...................................................................gguucgauuccUggcgccgg.........................    1
Gi3_27178009_x11..................................................................Ugguucgauucccggcgccgga........................    1
Gi2_10396785_x11.................uccggcguaguauaacggcuGg.........................................................................    1
Gi3_27166690_x11......................................................................Gcgauucccggcgccgga........................    1
Gi5_8413666_x11.................uccgAcguaguauaacgg.............................................................................    1
Gi1_3910612_x11.................Cccggcguaguauaacggcua..........................................................................    1
Gi3_27168615_x11......................................................................ucgauuccNggcgccggagc......................    1
Gi2_10371342_x11....................................................................guucgauucccggUgccgga........................    1
Gi2_10385290_x11....................................................................gCucgauucccggcgccggag.......................    1
Gi3_27151873_x11....................................................................guucgauucccggcgUcggagc......................    1
Gi3_27144360_x11............................................................aUauccggguucgauucc..................................    1
Gi5_8422455_x11....................................................................gAucgauucccggcgccg..........................    1
Gi5_8418671_x11.................uccggcguaguauaacggcuagaauggu...................................................................    0
Gi5_8419903_x11....................................................................guuUgauucccggcgccgga........................    1
Gi2_10376281_x11....................................................................guucgauucccggcgccggCg.......................    1
Gi3_27184686_x11.................uccggAguaguauaacgg.............................................................................    1
Gi1_3908104_x11................................................uucucacccaGcagauccg.............................................    1
Gi5_8423874_x11....................................................................guucgauucccgAcgccgga........................    1
Gi5_8426338_x11.................uccggcguaguauaacAg.............................................................................    1
Gi3_27180517_x11...............................................gCucucacccaacagauccg.............................................    1
Gi5_8426822_x11...................................................................gguucgauucccggcgcUg..........................    1
Gi3_27156548_x11.................uccggcguaguauaaUggcuag.........................................................................    1
Gi5_8415030_x11...............................................guucucacccaacGgauccg.............................................    1
Gi2_10383827_x11.................uccggcguaguauaGcgg.............................................................................    1
Gi1_3914561_x11.................................................Gcucacccaacagauccgg............................................    1
Gi2_10381913_x11....................................................................Auucgauucccggcgccggag.......................    1
Gi5_8418066_x11...................................................................gCuucgauucccggcgccg..........................    1
Gi3_27145086_x11.................uccggcguaguauaacggcuagCau......................................................................    1
Gi3_27183509_x11.................uccggcguaguauaacgAcuag.........................................................................    1
Gi5_8410355_x11..............................................Uguucucacccaacagauccggguu.........................................    1
Gi3_27169869_x11...............................................guucucacccaacagGuccg.............................................    1
Gi3_27179593_x11....................................................................guucgauuAccggcgccgga........................    1
Gi2_10373322_x11....................................................................guGcgauucccggcgccg..........................    1
Gi3_27178471_x11..............................................gguucucacccaacagaucAg.............................................    1
Gi3_27149948_x11....................gUcguaguauaacggcua..........................................................................    1
G1a_5609469_x11.................Gccggcguaguauaacggcuag.........................................................................    1
Gi5_8418286_x11...................................................................gguucgauuccUggcgccg..........................    1
Gi2_10377293_x11.................uccggcguaguauaGcggc............................................................................    1
Gi3_27167878_x11....................................................................guucgCuucccggcgccgg.........................    1
Gi2_10373432_x11...................................................................gguucgauuUccggcgcc...........................    1
Gi5_8410993_x11.................uccggcguaguGuaacggcua..........................................................................    1
Gi3_27172443_x11....................................................................guucgauucccggcgccgCagc......................    1
Gi2_10378382_x11.................uccgAcguaguauaacggcuaga........................................................................    1
Gi1_3910887_x11................................................uAcucacccaacagauccg.............................................    1
Gi3_27165073_x11.................uccggcguaguauaacggUuag.........................................................................    1
```

```
Gi3_27142325_x11.......................................................ccaacagauccggguucgauucccggcgccgg.........................   0
Gi2_10377172_x11.................uccggcguaguauCacggc............................................................................   1
Gi1_3915683_x11......................cguaguauaacggcuagaau......................................................................   0
Gi2_10384201_x11....................................................................guucgauucccggcgcAgg.........................   1
Gi3_27144250_x11.................uccggcguaguauCacggcuagaau......................................................................   1
Gi3_27186776_x11.....................................................................uuUgauucccggcgccggagc......................   1
Gi5_8428219_x11...............................................guucucacUcaacagauccg.............................................   1
Gi5_8427636_x11.................uccggcguaguGuaacggc............................................................................   1
Gi1_3905156_x11...................................................................gguucgauucccggcgcAgg.........................   1
Gi2_10387710_x11.................uccggcguaguauaacAgcuag.........................................................................   1
Gi2_10384608_x11.................uccgUcguaguauaacggcua..........................................................................   1
Gi3_27133789_x11.................uccggcguaguaGaacggcua..........................................................................   1
Gi2_10397500_x11.................uccggcguCguauaacggcuaga........................................................................   1
Gi3_27180308_x11....................................................................guuAgauucccggcgccgg.........................   1
Gi3_27225642_x10.................uccAgcguaguauaacggcuag.........................................................................   1
Gi5_8444156_x10...............................................Cuucucacccaacagauccg.............................................   1
Gi3_27192892_x10....................................................................guucgauucccgUcgccgg.........................   1
Gi1_3921274_x10....................................................................guCcgauucccggcgccg..........................   1
Gi2_10413142_x10....................................................................guucgauucccggcgccggGg.......................   1
Gi2_10414892_x10..............................................ggGucucacccaacagauccg.............................................   1
Gi3_27235662_x10.................uccggcCuaguauaacggcuaga........................................................................   1
Gi4_520909_x10....................................................................gGucgauucccggcgccgg.........................   1
Gi2_10415822_x10...............................................Auucucacccaacagauccg.............................................   1
Gi2_10415192_x10...............................................guucucacNcaacagauccg.............................................   1
Gi3_27205472_x10....................................................................guuUgauucccggcgccggag.......................   1
Gi2_10419012_x10...................................................................gCuucgauucccggcgcc...........................   1
Gi2_10412482_x10.................uccggcguaguCuaacggcuaga........................................................................   1
Gi1_3924494_x10.............................................ugguCcucacccaacagauccg.............................................   1
Gi1_3926694_x10...............................................guucCcacccaacagauccg.............................................   1
Gi2_10407042_x10...................................................................gguucgauuccUggcgccggag.......................   1
Gi2_10419612_x10.......................................................................cUauucccggcgccggagcguuu..................   1
Gi3_27209772_x10...................................................................gguucgauucccggcAccg..........................   1
Gi1_3927194_x10..............................................gguCcucacccaacagauccg.............................................   1
Gi2_10412472_x10...................................................................gguucAauucccggcgcc...........................   1
Gi1_3924034_x10....................Ugcguaguauaacggcua..........................................................................   1
Gi2_10403692_x10....................................................................guucgauucccgAcgccgg.........................   1
Gi5_8448436_x10....................................................................guucgauucccggcgccggGgc......................   1
Gi5_8436516_x10..............................................gguucucacAcaacagauccggguu.........................................   1
Gi1_3927214_x10.................uccggcguaguauCacggcuaga........................................................................   1
Gi5_8447406_x10....................................................................guucgCuucccggcgccgg.........................   1
Gi5_8435356_x10...................................................................gguCcgauucccggcgccg..........................   1
Gi3_27190342_x10.................uccggcguCguauaacggcua..........................................................................   1
Gi5_8452316_x10....................................................................guucgauuNccggcgccggagc......................   1
Gi1_3920084_x10.............................................Cgguucucacccaacagauccgg............................................   1
Gi2_10427882_x10...................................................................gguucgauucccggcgccCgagc......................   1
Gi2_10405312_x10....................................................................guucgauucccggcgcAggagc......................   1
Gi5_8431736_x10.................uccggcguaguauaacggcuagCau......................................................................   1
```

```
Gi3_27239032_x10.................uccggcgCaguauaacggcuagaau......................................................................   1
Gi1_3928104_x10..................................................cGcacccaacagauccgg............................................   1
Gi5_8433596_x10................................................uucucacccaGcagauccg.............................................   1
Gi1_3926114_x10.................Cccggcguaguauaacggcuag.........................................................................   1
Gi5_8442986_x10.................uccgAcguaguauaacggcuagaau......................................................................   1
Gi2_10407172_x10....................................................................guucgauucccgAcgccggagc......................   1
Gi3_27220262_x10....................................................................guucgauucccggcgccC..........................   1
Gi2_10411892_x10....................................................................guucgGuucccggcgccg..........................   1
Gi3_27220922_x10...................................................................gguucgauucccUgcgccg..........................   1
Gi2_10415872_x10.................uUcggcguaguauaacggcua..........................................................................   1
Gi2_10411452_x10.................uccggcguaguauaacggcCa..........................................................................   1
Gi3_27239182_x10......................................................................Gcgauucccggcgccggag.......................   1
Gi2_10399502_x10...............................................guucucacccaacagGuccg.............................................   1
Gi5_8452576_x10...................................................................gguucgauucccggAgccggagc......................   1
Gi3_27188212_x10....................................................................guucgCuucccggcgccgga........................   1
Gi3_27198502_x10..................ccggcguaguauaacggcuagaau......................................................................   0
Gi3_27226722_x10....................................................................guucgauucccggcAccggagc......................   1
Gi3_27190712_x10...................................................................gguucgauucAcggcgccgga........................   1
Gi3_27240502_x10....................................................................Cuucgauucccggcgccg..........................   1
Gi5_8439896_x10.................uccggcAuaguauaacggcuagaau......................................................................   1
Gi3_27204172_x10...................................................................gguucAauucccggcgccg..........................   1
Gi3_27233622_x10..................................................................gCguucgauucccggcgccggagc......................   1
Gi3_27191172_x10...................................................................gguucgCuucccggcgccgg.........................   1
Gi3_27205302_x10.................uccggcguaguauaacggcuaCa........................................................................   1
Gi2_10401452_x10....................................................................guucgauucccggcAccg..........................   1
Gi1_3920724_x10.........................................................aacagauccggguucgauucccggcgccggagc......................   0
Gi2_10397762_x10....................................................................guucgauuUccggcgccggagc......................   1
Gi3_27217962_x10.................uccggAguaguauaacggc............................................................................   1
Gi5_8434636_x10...................................................................ggAucgauucccggcgccg..........................   1
Gi2_10417612_x10.................uccggcguaguauaGcggcua..........................................................................   1
Gi1_3917484_x10................................................uucucacccaacagauAcgg............................................   1
Gi3_27217682_x10.............................................uggCucucacccaacagauccg.............................................   1
Gi3_27231662_x10..............................................gguucAcacccaacagauccg.............................................   1
Gi2_10423752_x10.................uccggcguaguGuaacgg.............................................................................   1
Gi2_10409482_x10.................uccggcguaguauaacgAc............................................................................   1
Gi5_8434526_x10................................................uGcucacccaacagauccgg............................................   1
Gi5_8452586_x10.............................................uggCucucacccaacagauccg.............................................   1
Gi3_27240962_x10..............................................gguucucaccUaacagauccg.............................................   1
Gi5_8435706_x10.................uccggcguaguCuaacggcuag.........................................................................   1
Gi1_3917414_x10..................ccggcguaguauaacggcuagaa.......................................................................   0
Gi1_3923774_x10...................................................................gguucgauuUccggcgccgg.........................   1
Gi1_3926524_x10...............................................guucucacccaCcagauccgg............................................   1
Gi3_27206282_x10...................................................................gguucgauucccgAcgccg..........................   1
Gi2_10403512_x10...................................................................gguucgauucccggcgccgUagc......................   1
Gi5_8449706_x10.................................................ucucacccNacagauccg.............................................   1
Gi3_27205052_x10.................uUcggcguaguauaacggcuagaa.......................................................................   1
Gi2_10409192_x10...................................................................gguuUgauucccggcgcc...........................   1
```

```
Gi1_3926564_x10...............................................guucucacccaGcagauccg.............................................    1
Gi3_27217412_x10...................................................................gguucgauuccAggcgccggag.......................    1
Gi1_3923174_x10....................................................................guucgaAucccggcgccgg.........................    1
Gi3_27209962_x10.................uccggcguagGauaacggcuaga........................................................................    1
Gi3_27189252_x10.................uccggcguaguGuaacggcuagaa.......................................................................    1
Gi1_3920124_x10.............................................ugguucucacUcaacagauccgg............................................    1
Gi1_3928064_x10.................uccggcguaguauaacgAcuaga........................................................................    1
Gi2_10412842_x10..............................................gguuUucacccaacagauccg.............................................    1
Gi5_8447646_x10....................................................................guucgauucAcggcgccgg.........................    1
Gi3_27209272_x10....................................................................guucgauucccggcgccggUgc......................    1
Gi3_27197082_x10....................................................................guAcgauucccggcgccggag.......................    1
Gi1_3917474_x10.................uccggcguaguauCacggcuag.........................................................................    1
Gi5_8448566_x10.................uccggcguaguauaCcggcuaga........................................................................    1
Gi3_27222292_x10.................uccggcguaguauaaUggcuagaau......................................................................    1
Gi2_10403602_x10..............................................gguucCcacccaacagauccg.............................................    1
Gi1_3925234_x10...............................................gCucucacccaacagauccgg............................................    1
Gi1_3927234_x10.................uccggcguGguauaacggcuaga........................................................................    1
Gi2_10410772_x10...................................................................gguucgauucccggcgUcgga........................    1
Gi3_27222132_x10.................uccggcguaguauaaUggcuagaa.......................................................................    1
Gi2_10404252_x10...................................................................gguucgauuUccggcgccgga........................    1
Gi5_8448506_x10.................uccggcguaguauGacggcuagaau......................................................................    1
Gi5_8449856_x10...................................................................gguucgGuucccggcgccg..........................    1
Gi3_27243812_x10.................uccgUcguaguauaacggcuagaa.......................................................................    1
Gi5_8447776_x10.................ucUggcguaguauaacggcuagaa.......................................................................    1
Gi3_27204492_x10....................................................................guucgauucccgAcgccgga........................    1
Gi1_3916754_x10...................................................................gguucAauucccggcgccgg.........................    1
Gi5_8451806_x10.................uccggcguaAuauaacggcuaga........................................................................    1
Gi3_27204082_x10....................................................................guucgauucccggcgcUggagc......................    1
Gi5_8447786_x10...................................................................gguucgauucccgAcgccggagc......................    1
Gi4_520139_x10..............................................gguucucacccaacagauccg.............................................    0
Gi3_27210902_x10....................................................................guucgauAcccggcgccggagc......................    1
Gi5_8439926_x10.............................................Cgguucucacccaacagauccg.............................................    1
Gi3_27221662_x10...............................................gGucucacccaacagauccgggu..........................................    1
Gi1_3917614_x10.................uccggcguaguauaaUggcua..........................................................................    1
Gi2_10403522_x10...................................................................gguucgCuucccggcgcc...........................    1
Gi2_10427752_x10.................uccggAguaguauaacggcuaga........................................................................    1
Gi2_10412292_x10...................................................................gguucgUuucccggcgccggag.......................    1
Gi2_10417302_x10...................................................................gguucgauucccggcgcGggagc......................    1
Gi2_10409622_x10...................................................................gguucgauucccggcgccggC........................    1
Gi2_10412052_x10.................uccggcguaguauaacggcuagaaC......................................................................    1
Gi1_3922454_x10.................uccgUcguaguauaacggcua..........................................................................    1
Gi3_27302113_x9...................................................................gguuAgauucccggcgccggag.......................    1
Gi2_10437555_x9....................................................................guucgauucccggcgcccCg........................    1
Gi5_8472180_x9...................................................................gguucgauuccUggcgccgg.........................    1
Gi5_8466564_x9.................uccggcguaguGuaacggcuagaa.......................................................................    1
Gi2_10446852_x9...................................................................gguucgauucccggUgccg..........................    1
Gi5_8458914_x9..............................................gguucucacccaacUgauccg.............................................    1
```

Gi3_27285409_x9...guucucacccaCcagauccg...   1
Gi3_27297991_x9...ggAucgauucccggcgccgg.........................   1
Gi5_8461047_x9...Uguucgauucccggcgccggagcgu....................   1
Gi5_8454072_x9..Ggauccggguucgauuccc.................................   1
Gi2_10434306_x9...gguucgauucccggcgccggaUc......................   1
Gi1_3936475_x9...gguucgauNcccggcgccg..........................   1
Gi3_27247375_x9...gguucgauucccggcCccggagc......................   1
Gi2_10446348_x9.................uccggcguaAuauaacggcuaga..   1
Gi2_10447644_x9...gguucgCuucccggcgccg..........................   1
Gi3_27248194_x9....................Ugcguaguauaacggcuag...   1
Gi3_27249112_x9..guGcgauucccggcgccg..........................   1
Gi3_27260965_x9..guucgauucccAgcgccg..........................   1
Gi5_8466123_x9..guucgauucccggAgccg..........................   1
Gi3_27273718_x9..uucucacccGacagauccg...   1
Gi1_3932884_x9..gCucgauucccggcgccggagc......................   1
Gi5_8465142_x9...guucucacccaacagaucUg...   1
Gi3_27296749_x9..ggguucgauucccggcgccggagcC.....................   1
Gi5_8473674_x9.................uccgAcguaguauaacggcuaga..   1
Gi5_8470929_x9.................Accggcguaguauaacgg...   1
Gi2_10450506_x9...Cguucgauucccggcgccgga........................   1
Gi1_3931039_x9...ugguucucGcccaacagauccg...   1
Gi3_27245278_x9..uucucUcccaacagauccg...   1
Gi1_3940336_x9..uucucaccUaacagauccg...   1
Gi2_10447860_x9..guucgauucAcggcgccgg.........................   1
Gi5_8462433_x9.................uccggcguaguauaacggcuC..   1
G1a_5644372_x9.................uGcggcguaguauaacggcuaga..   1
Gi5_8465457_x9.................uccggUguaguauaacggcuagaau..   1
Gi2_10448607_x9..guucgauucccggcgAcg..........................   1
Gi1_3932569_x9.................uccggcguagCauaacggcuag...   1
Gi5_8460930_x9..guucgauucccggcgcUgga........................   1
Gi5_8476581_x9..guucgauucccAgcgccgga........................   1
Gi5_8468166_x9...guucucacccaacagaucAg...   1
Gi1_3939337_x9.................uccggcguGguauaacggc..   1
Gi5_8463774_x9..gGuucccggcgccggagc......................   1
Gi1_3933739_x9...guucucacccaacCgauccgg..   1
Gi5_8461218_x9...ggGucgauucccggcgcc...........................   1
Gi5_8461515_x9..guucUauucccggcgccgga........................   1
Gi1_3931111_x9..uucucacccaacagaucUg...   1
Gi1_3931120_x9..ucgauucccggcgccggagcC.....................   1
Gi5_8453280_x9...ugguucucNcccaacagauccg...   1
Gi2_10439625_x9.................ucUggcguaguauaacgg...   1
Gi5_8459643_x9...gguucgGuucccggcgccgg.........................   1
Gi5_8455422_x9...Ggguucucacccaacagauccgg..   1
Gi5_8465079_x9.................uccggcguaguauaCcggc..   1
G1a_5650780_x9.................uGcggcguaguauaacggcuag...   1
Gi3_27271405_x9..guucgauucccCgcgccgg.........................   1
Gi3_27245107_x9...ggGucgauucccggcgccgg.........................   1

```
Gi3_27303679_x9................................................uucucacccaCcagauccgg............................................    1
Gi5_8473449_x9....................Ugcguaguauaacggcuaga........................................................................    1
Gi5_8458428_x9...................................................................gguucgauucccggcgccgC.........................    1
Gi5_8458752_x9...................................................................gguucgauucccggcgccAga........................    1
Gi5_8466249_x9.................uccggcgCaguauaacggc............................................................................    1
Gi2_10432020_x9....................................................................guucAauucccggcgccgga........................    1
Gi3_27290737_x9...................................................................gguucgauuccGgcgccgga.........................    1
Gi2_10454124_x9.................uccggcguaguauaacggcuagaCu......................................................................    1
Gi3_27270208_x9.................uccgAcguaguauaacgg.............................................................................    1
G1a_5644426_x9....................................................................guucgauucccggcgccggag.......................    0
Gi5_8473503_x9.................uccggcguaguCuaacggcua..........................................................................    1
Gi5_8453325_x9..................................................cucaccUaacagauccgg............................................    1
Gi4_521253_x9 ....................................................................guucgauucccggcgccggagc......................    0
Gi1_3932146_x9...................................................................gguucgauucccggcgccggagcgA....................    1
Gi1_3929473_x9.................uccggcguaguauCacggcua..........................................................................    1
Gi2_10438842_x9....................................................................guucgauucccggcgUcgga........................    1
Gi3_27256195_x9...................................................................gguucgauucccggcgccgUagc......................    1
Gi5_8453658_x9.................uccggcguaguauaacggcuaA.........................................................................    1
Gi3_27264709_x9.................uccggcgAaguauaacggcuaga........................................................................    1
Gi3_27268903_x9...................................................................gguGcgauucccggcgccggagc......................    1
Gi5_8463549_x9.................uccggcguaguauaaUggc............................................................................    1
Gi2_10447014_x9...............................................gCucucacccaacagauccg.............................................    1
Gi3_27257707_x9..............................................gguucucacccaaUagauccg.............................................    1
Gi2_10458642_x9...................................................................gguucgauucccggAgccg..........................    1
Gi5_8453289_x9..............................................gguucucCcccaacagauccg.............................................    1
Gi1_3933055_x9.................uccggcgCaguauaacggcuag.........................................................................    1
Gi3_27258040_x9.................Cccggcguaguauaacggcuag.........................................................................    1
Gi2_10451217_x9.................uccggcguaguauaacAg.............................................................................    1
Gi1_3937897_x9.................uccggcguaguauGacgg.............................................................................    1
Gi2_10457940_x9.................uccggcguaguauaUcggcuaga........................................................................    1
Gi1_3929077_x9..............................................gguucucacccaacGgauccg.............................................    1
Gi5_8469930_x9...................................................................gguucgauucccggcAccgga........................    1
G1a_5648809_x9.....................................................acccaacagauccggguucgauuccc.................................    0
Gi2_10434270_x9...................................................................gguucgauucccCgcgccggagc......................    1
Gi5_8467374_x9....................................................................guucgauucccCgcgccgg.........................    1
Gi5_8454351_x9...................................................................ggGucgauucccggcgccgg.........................    1
Gi3_27263017_x9....................................................................guucgauucccCgcgccgga........................    1
Gi2_10443882_x9...................................................................gguucAauucccggcgccggag.......................    1
Gi1_3934828_x9...............................................guucucaUccaacagauccg.............................................    1
Gi5_8455944_x9.................uccggcguaguauaacggcuagU........................................................................    1
Gi5_8459157_x9..............................................gguucAcacccaacagauccg.............................................    1
Gi1_3933244_x9................................................uucucacccaacagaucUgg............................................    1
Gi2_10455438_x9.................uccggcgCaguauaacggcua..........................................................................    1
Gi1_3941641_x9................................................uucucacUcaacagauccg.............................................    1
Gi2_10457346_x9...................................................................gguucgauCcccggcgccg..........................    1
Gi5_8471865_x9.................uccggcguagCauaacggcuagaau......................................................................    1
Gi2_10443045_x9....................................................................guucgauucccggcgccggagA......................    1
```

```
Gi3_27306514_x9...............................................guucucacccaGcagauccg.............................................     1
Gi1_3935098_x9.................uccggUguaguauaacggc............................................................................     1
Gi5_8470668_x9...................................................................gguucgauucccggcgccggaCc......................     1
Gi3_27283429_x9....................................................................guucgauucccggAgccgga........................     1
Gi2_10461621_x9.................uccggcguagCauaacggc............................................................................     1
Gi1_3936016_x9....................................................................guuUgauucccggcgccgga........................     1
Gi5_8469489_x9....................................................................guucgauAcccggcgccg..........................     1
Gi1_3934774_x9.................uccggcguaguauCacgg.............................................................................     1
Gi5_8455935_x9.................uccggcguaguauGacgg.............................................................................     1
Gi5_8464125_x9.....................................................................Gucgauucccggcgccgg.........................     1
Gi5_8463207_x9.............................................Ggguucucacccaacagauccgggu..........................................     1
Gi5_8467230_x9...................................................................gguucgCuucccggcgccggag.......................     1
Gi5_8464548_x9...............................................guucucacccCacagauccg.............................................     1
Gi3_27281638_x9.......................................................................Ggauucccggcgccggagc......................     1
Gi3_27288757_x9....................................................................guucAauucccggcgccggag.......................     1
Gi3_27257527_x9.................uccggcguaguauaacgC.............................................................................     1
Gi2_10440687_x9...................cggcguaguauaacggcuagaaC......................................................................     1
Gi1_3940768_x9.................uccAgcguaguauaacgg.............................................................................     1
Gi2_10495458_x8.................uccggUguaguauaacgg.............................................................................     1
Gi5_8479161_x8..................................................cucGcccaacagauccg.............................................     1
Gi5_8499257_x8..............................................gguucuUacccaacagauccg.............................................     1
Gi3_27328089_x8....................................................................guucgauucAcggcgccgg.........................     1
Gi2_10487194_x8.....................................................................Gucgauucccggcgccgg.........................     1
Gi3_27350665_x8.................uccggcguUguauaacggcuagaau......................................................................     1
Gi2_10494178_x8...................................................................gguucgauucccggcAccggag.......................     1
Gi2_10474298_x8...................................................................gguucgauucccggcgccggagG......................     1
Gi1_3947829_x8...................................................................gguuUgauucccggcgccgg.........................     1
Gi1_3949949_x8.................uccgAcguaguauaacgg.............................................................................     1
Gi1_3946485_x8..............................................gguucucacccCacagauccg.............................................     1
Gi3_27324993_x8..............................................ggAucucacccaacagauccg.............................................     1
Gi1_3947997_x8.................uccggcguaguGuaacggcua..........................................................................     1
Gi1_3951533_x8.................uGcggcguaguauaacggc............................................................................     1
Gi2_10486098_x8...................................................................gguucgaCucccggcgccgg.........................     1
Gi3_27359369_x8....................................................................guucgauucccggcgccggaAc......................     1
Gi3_27360993_x8....................................................................guucgauucccggcgccggUg.......................     1
Gi5_8498865_x8....................................................................guucgUuucccggcgccg..........................     1
Gi2_10474722_x8.................uccggcguUguauaacggc............................................................................     1
Gi1_3952509_x8.................uccggcguaguauaacggUuaga........................................................................     1
Gi2_10475530_x8.................uccggcguaguauaacggcuaAaau......................................................................     1
Gi2_10487810_x8....................................................................guCcgauucccggcgccggag.......................     1
Gi2_10478338_x8...................................................................gguucgauucccggAgcc...........................     1
Gi1_3943093_x8.................................................ucucGcccaacagauccg.............................................     1
Gi4_524184_x8 ...............................................guucucacccaacagauccg.............................................     0
Gi2_10479218_x8...................................................................gguucgauucccggcgAcgga........................     1
Gi2_10480930_x8...................................................................gguucgCuucccggcgccgga........................     1
Gi3_27317417_x8.................uccggcguaguCuaacggcuag.........................................................................     1
Gi2_10488106_x8..............................................gguucucacccGacagauccg.............................................     1
```

Gi3_27317057_x8...gGucucacccaacagauccggg... 1
Gi3_27330617_x8...ugguucucGcccaacagauccg... 1
Gi1_3950797_x8..gAuucucacccaacagauccg... 1
Gi3_27341417_x8.................uGcggcguaguauaacggcu... 1
Gi5_8490161_x8...ggGucgauucccggcgccggag....................... 1
Gi5_8479537_x8...gguucgauucccggAgccgga........................ 1
Gi2_10479466_x8..guucgauucccCgcgccggagc...................... 1
Gi2_10494842_x8..ucgauucccggcgccggGgc...................... 1
Gi3_27341601_x8.................uccggcguaguauGacggcuagaau.. 1
Gi2_10488730_x8...gguucgauucccggcAccgg......................... 1
Gi2_10466818_x8..Cuucgauucccggcgccgga........................ 1
Gi1_3954701_x8.................uccggcguaguCuaacgg... 1
Gi2_10476746_x8...gguucgauNcccggcgccgg......................... 1
G1a_5671057_x8...Aguucgauucccggcgccggagc...................... 1
Gi5_8479753_x8...guucucacccaacUgauccg... 1
Gi1_3947309_x8..guucgauucccggcgccggGgc...................... 1
Gi3_27346385_x8..guucgauucccggcgcccCgagc...................... 1
Gi1_3954109_x8....................gUcguaguauaacggcuag... 1
Gi1_3943317_x8.................uccggcguaguauaacggcuaA... 1
Gi2_10475850_x8...gguucAauucccggcgccgga........................ 1
Gi5_8496129_x8...ggCucgauucccggcgccgg......................... 1
Gi3_27356329_x8..gauucccAgcgccggagc...................... 1
Gi1_3950469_x8.................uccggcguaguauaacggcuaAaau.. 1
Gi2_10497218_x8..guucgauucccAgcgccgga........................ 1
Gi3_27348209_x8...gguuUgauucccggcgcc........................... 1
Gi2_10481186_x8...gguucgauucUcggcgccggag....................... 1
Gi1_3949389_x8..gguucucacccaGcagauccg... 1
Gi3_27361169_x8.................uccggcUuaguauaacggcuaga.. 1
Gi2_10467834_x8..guucgauucccggcCccggagc...................... 1
Gi5_8487249_x8..guucgauucccggcgccggG........................ 1
Gi2_10464722_x8...gguucgGuucccggcgccg.......................... 1
Gi4_526408_x8 ....gguucgauucccggcgccgga........................ 0
Gi5_8479385_x8.................uccggcguaguauCacggcuagaau.. 1
Gi1_3957061_x8...ugguucucacccaacagauAcgg.. 1
Gi1_3955053_x8...gguucgauucccggcgccggagcA..................... 1
Gi5_8488025_x8...ggAucgauucccggcgccgga........................ 1
Gi2_10497954_x8.................uccggcguaguauGacggcuaga.. 1
Gi3_27332217_x8..uucucacccaacagGuccgg.. 1
Gi2_10481602_x8.................uccggcguaguauaacggcuagU.. 1
Gi1_3942421_x8...gguucgauucUcggcgccgg......................... 1
Gi2_10495786_x8..guucgauucAcggcgccggagc...................... 1
Gi2_10482514_x8.................uccggcguaguauaacggcuC.. 1
Gi2_10494290_x8.................uccggcguaguauaacAgcua.. 1
Gi5_8476993_x8..agauccggNuucgauuccc................................. 1
Gi2_10482586_x8..guucgauucccggcgccUgagc...................... 1
Gi3_27337049_x8...guucucaccUaacagauccg... 1
Gi5_8494881_x8.................uccggcguaguauaaAggcuag... 1

```
Gi5_8494609_x8...................................................................gguucgauucccggcgUc...........................      1
Gi1_3945037_x8...................................................................gguucgauucccggcgccggG........................      1
Gi5_8481825_x8...................................................................ggCucgauucccggcgccggag.......................      1
Gi2_10492602_x8.................................................ucucacccaacagauccg.............................................      0
Gi1_3946141_x8...................................................................gguCcgauucccggcgccgg.........................      1
Gi2_10483890_x8...................................................................ggCucgauucccggcgccgga........................      1
Gi2_10467634_x8....................................................................guucgauucccgUcgccggagc......................      1
Gi5_8486761_x8.................uccggcguaguCuaacggcuaga........................................................................      1
Gi2_10487602_x8....................................................................guucgauuccAggcgccgg.........................      1
Gi2_10487410_x8...................................................................gguucgauucUcggcgcc...........................      1
Gi2_10462786_x8.................ucUggcguaguauaacggcuag.........................................................................      1
Gi2_10483818_x8.................uccggcguGguauaacggcuagaau......................................................................      1
Gi5_8483369_x8.................uccggcguaguauaaUggcuagaau......................................................................      1
Gi3_27317361_x8.................uccggcguaguaCaacggcuagaa.......................................................................      1
Gi5_8482345_x8..................................................................gAuucgauucccggcgcc............................      1
Gi5_8485265_x8.................uccggcguaguauGacggcuag.........................................................................      1
Gi1_3954469_x8...............................................guucucaccUaacagauccg.............................................      1
Gi5_8493593_x8...................................................................gguucgauucccggcgUcgga........................      1
Gi1_3944557_x8..............................................gguucucacccaacagGuccg.............................................      1
Gi2_10472362_x8...................................................................gguucgauucccAgcgccgga........................      1
Gi3_27363793_x8....................................................................guucgauucccggcgGcgga........................      1
Gi5_8498585_x8..............................................gguucucacAcaacagauccg.............................................      1
Gi3_27342481_x8...................................................................gguucgauuccAggcgccg..........................      1
Gi3_27347513_x8.................uccggcguaguauaaAgg.............................................................................      1
Gi2_10477890_x8...................................................................gguuUgauucccggcgccg..........................      1
Gi3_27324385_x8.....................................................................Gucgauucccggcgccgg.........................      1
Gi2_10486738_x8...................................................................gguucgauucccggcgGcggagc......................      1
Gi2_10471362_x8.........................................................aacagauccggguucgauucccggcgccgg.........................      0
Gi3_27318697_x8..............................................gguucucacccaacagauAcg.............................................      1
Gi5_8486489_x8....................................................................gGucgauucccggcgccggag.......................      1
Gi5_8488249_x8.................uccggcguagCauaacggcua..........................................................................      1
Gi5_8493065_x8...................................................................gguucgauucUcggcgccggagc......................      1
Gi5_8483649_x8.....................gcguaguauaacggcuaga........................................................................      0
Gi5_8477473_x8.................uccAgcguaguauaacggc............................................................................      1
Gi1_3955573_x8.................uccggcguaguauaacggcuaAa........................................................................      1
Gi4_525296_x8 ...................................................................gUuucgauucccggcgccggagc.....................      1
Gi1_3942557_x8.................uccggcguaguauaacggcCa..........................................................................      1
Gi1_3952357_x8..............................................gguucucacccaacagauAcg.............................................      1
G1a_5654737_x8...............................................gGucucacccaacagauccg.............................................      1
Gi5_8488777_x8.................uccggcguCguauaacgg.............................................................................      1
Gi1_3943805_x8....................................................................guucgUuucccggcgccgg.........................      1
Gi5_8491289_x8.................uccggcguaguauCacggc............................................................................      1
Gi5_8493825_x8...................................................................ggGucgauucccggcgccgga........................      1
Gi5_8481609_x8..........................................................................................Uuuucccgccuuccuucgucu.      1
Gi2_10492594_x8....................................................................guucgauCcccggcgccgga........................      1
Gi5_8488537_x8...............................................uucucaccAaacagauccg..............................................      1
Gi1_3943365_x8...................................................................gguucgauucccggcgccggagU......................      1
```

```
Gi2_10472842_x8....................................................................guucgauuNccggcgccgga........................     1
Gi3_27328017_x8....................................................................guucgaGucccggcgccgga........................     1
Gi3_27335497_x8.......................................................................cgUuucccggcgccggagc......................     1
Gi2_10495498_x8.................uccggUguaguauaacggc............................................................................     1
Gi3_27369817_x8.................ucGggcguaguauaacggcua..........................................................................     1
Gi5_8497897_x8....................................................................guucgGuucccggcgccggagc......................     1
Gi3_27364521_x8...................................................................gguucgauucccggAgccggag.......................     1
Gi3_27361201_x8................................................uucucacccaacagauAcg.............................................     1
Gi5_8493233_x8...................................................................gCuucgauucccggcgccgg.........................     1
Gi2_10489418_x8.................uccggcguaguauaGcggcuagaau......................................................................     1
Gi5_8476985_x8..............................................gguucucacccaacagauAcg.............................................     1
Gi2_10469098_x8...................................................................gguucgauucccggcgccA..........................     1
Gi2_10472418_x8...................................................................gguCcgauucccggcgccggag.......................     1
Gi5_8477785_x8....................................................................guCcgauucccggcgccgga........................     1
Gi3_27331577_x8...................................................................gguucgCuucccggcgcc...........................     1
Gi5_8492497_x8.................................................ucCcacccaacagauccg.............................................     1
Gi5_8500185_x8.................Cccggcguaguauaacggcuagaa.......................................................................     1
Gi1_3948949_x8.................uccggcguaguauaacAgcua..........................................................................     1
Gi3_27358193_x8.................uccggcgCaguauaacggcuagaa.......................................................................     1
Gi3_27310729_x8...................................................................gguucgauucccggcgccgCag.......................     1
Gi1_3943637_x8.................uccggcguaguauaacggUuag.........................................................................     1
Gi2_10480546_x8...................................................................gguucgauucccggUgccgga........................     1
Gi5_8487297_x8....................................................................guucgauuccAggcgccg..........................     1
Gi2_10469946_x8....................................................................guucgUuucccggcgccgg.........................     1
Gi3_27354289_x8...............................................guucucacccaacGgauccg.............................................     1
Gi3_27320833_x8.......................................................................cgauucccggcgccggCgc......................     1
Gi1_3943421_x8...................................................................gguucgauucccggcgccggU........................     1
Gi5_8488329_x8.................uccggcguaguauaaUggcua..........................................................................     1
Gi3_27353129_x8.................uccggcguaguauaacggcCagaa.......................................................................     1
Gi2_10493754_x8....................................................................guucgauuAccggcgccggagc......................     1
Gi2_10466082_x8...................................................................gguucgauucccggcgUcg..........................     1
Gi5_8488553_x8.................uccggcguaguauGacggcuaga........................................................................     1
Gi3_27366841_x8.................uccgUcguaguauaacggcuagaau......................................................................     1
Gi1_3952669_x8.................uccggcguNguauaacggcuaga........................................................................     1
Gi3_27366081_x8.................uccgAcguaguauaacggcua..........................................................................     1
Gi3_27359665_x8...................................................................gguucgauuccGggcgccg..........................     1
Gi2_10498522_x8.................uccggcguNguauaacggcuagaa.......................................................................     1
Gi5_8501385_x8...................................................................gguucgauuccAggcgccggagc......................     1
Gi2_10486802_x8......................................................................ucgauucUcggcgccggagc......................     1
Gi3_27323897_x8.............................................ugguucucacccaacagGuccg.............................................     1
Gi5_8496041_x8...................................................................gguucgauucccggcgAcgga........................     1
Gi3_27374497_x8....................................................................gAucgauucccggcgccggag.......................     1
Gi3_27362081_x8....................................................................guucgauucccggcgcGgga........................     1
Gi5_8482833_x8.................uccggcguaguCuaacgg.............................................................................     1
Gi5_8486097_x8....................................................................guuUgauucccggcgccggagc......................     1
Gi3_27320697_x8....................................................................guucgauucccggGgccgg.........................     1
Gi1_3952253_x8.................Cccggcguaguauaacggcuaga........................................................................     1
```

```
Gi1_3943245_x8.................ucUggcguaguauaacggc............................................................................      1
Gi5_8504495_x7.................uccggcguaguauaCcgg.............................................................................      1
Gi1_3962561_x7...................................................................ggAucgauucccggcgccgg.........................      1
Gi1_3973922_x7.....................................................................uuUgauucccggcgccgg.........................      1
Gi5_8503221_x7.................uccggcguCguauaacggcuaga........................................................................      1
Gi5_8514904_x7....................................................................Auucgauucccggcgccggagc......................      1
Gi1_3970982_x7...............................................Auucucacccaacagauccgg............................................      1
Gi3_27424451_x7..................................................cucacccaNcagauccgg............................................      1
Gi3_27386875_x7....................................................................guucgauucccggcgccggaA.......................      1
Gi3_27449252_x7....................................................................guucgauucccggcgccggaCc......................      1
Gi2_10537645_x7................................................uucucaccNaacagauccg.............................................      1
Gi1_3962106_x7...............................................guucucGcccaacagauccgg............................................      1
Gi3_27432732_x7.................uccggcguGguauaacggcuagaa.......................................................................      1
Gi2_10536210_x7...................................................................gguucgauucccggUgccgg.........................      1
Gi1_3973369_x7...................................................................gguucgauucccggcgccggCgc......................      1
Gi3_27435749_x7................................................uucuUacccaacagauccg.............................................      1
Gi3_27447810_x7...................................................................gguucgauucUcggcgccg..........................      1
Gi3_27385167_x7...............................................guucucacccaacagaCccg.............................................      1
Gi2_10538863_x7.................uccggcguaguauaacggcuGgaa.......................................................................      1
Gi2_10503373_x7......................................................................ucgaCucccggcgccggagc......................      1
Gi5_8511789_x7...............................................guuUucacccaacagauccg.............................................      1
Gi2_10505571_x7.................uccggcguNguauaacggcuagaau......................................................................      1
G1a_5682142_x7...................................................................gguucgauNcccggcgccggagc......................      1
Gi5_8525586_x7...............................................guucAcacccaacagauccg.............................................      1
Gi1_3969239_x7..............................................gguucucacccaacagaucAg.............................................      1
Gi5_8518145_x7.................uccggcAuaguauaacggcuaga........................................................................      1
Gi3_27416485_x7...................................................................gguucgauucccggAgcc...........................      1
Gi2_10525304_x7...................................................................gguucgauucccggcgGcg..........................      1
Gi2_10529371_x7...................................................................gguucgaCucccggcgccggag.......................      1
Gi2_10512207_x7....................................................................guucgauucccggcgccCgagc......................      1
Gi5_8512531_x7................................................uGcucacccaacagauccggguuc........................................      1
Gi3_27448104_x7......................................................................ucgaCucccggcgccggagc......................      1
Gi1_3965249_x7................................................uucucacccUacagauccgg............................................      1
Gi3_27451499_x7....................................................................guucgauucccggUgccg..........................      1
Gi1_3968728_x7.................uccggcgCaguauaacggcua..........................................................................      1
Gi5_8530668_x7.................................................Ccucacccaacagauccg.............................................      1
Gi5_8516255_x7.................uccggcguaguauaCcggcua..........................................................................      1
Gi3_27407007_x7.................ucUggcguaguauaacggcu...........................................................................      1
Gi3_27423996_x7...............................................guucucacccCacagauccgg............................................      1
Gi5_8511635_x7...............................................guucucacNcaacagauccgg............................................      1
Gi3_27425508_x7....................................................................guCcgauucccggcgccg..........................      1
Gi5_8515625_x7..............................................gguucucacccaacCgauccg.............................................      1
Gi2_10517387_x7...................................................................ggGucgauucccggcgccg..........................      1
Gi1_3967076_x7...................................................................gguucgauucccggcgccggGgc......................      1
Gi5_8526174_x7..............................................gguucucUcccaacagauccg.............................................      1
Gi5_8504649_x7.............................................ugguucucGcccaacagauccg.............................................      1
Gi3_27444058_x7..............................................gguAcucacccaacagauccg.............................................      1
```

```
Gi1_3960223_x7.............................................ugguucucacccaacGgauccg.............................................     1
Gi3_27445787_x7...................................................................gguucgauucccggcgcUg..........................     1
Gi3_27411053_x7.................uccggcguaAuauaacggc............................................................................     1
Gi5_8503165_x7....................................................................guucgauuAccggcgccg..........................     1
Gi2_10525598_x7...................................................................gguucgauucccggcgccC..........................     1
Gi3_27382640_x7....................................................................guucgauucccggcgcUg..........................     1
Gi5_8512132_x7....................................................................guuAgauucccggcgccgg.........................     1
Gi2_10515805_x7.................uccggcguaguauaacggcuaUa........................................................................     1
Gi2_10525038_x7....................................................................guucUauucccggcgccg..........................     1
Gi1_3963856_x7.............................................ugguucucacAcaacagauccgg............................................     1
Gi1_3970065_x7.................Gccggcguaguauaacggcuagaa.......................................................................     1
Gi3_27407525_x7....................................................................guucgauucccggcgccggCg.......................     1
Gi2_10504444_x7.................uccgUcguaguauaacgg.............................................................................     1
Gi1_3957976_x7....................................................................Cuucgauucccggcgccgga........................     1
Gi1_3963688_x7....................................................................guuAgauucccggcgccgg.........................     1
Gi1_3967559_x7.................Accggcguaguauaacgg.............................................................................     1
Gi3_27409758_x7......................................................................ucgGuucccggcgccggagc......................     1
Gi2_10501630_x7....................................................................guucgauucccggUgccggag.......................     1
Gi3_27427447_x7............................................ugguucucacAcaacagauc................................................     1
Gi2_10513705_x7.................Accggcguaguauaacggcuaga........................................................................     1
Gi5_8505391_x7...................cUgcguaguauaacggcuag.........................................................................     1
Gi2_10537638_x7.................uccgAcguaguauaacggc............................................................................     1
Gi5_8505384_x7....................gUcguaguauaacggcuaga........................................................................     1
Gi5_8524718_x7.................Cccggcguaguauaacggcuagaau......................................................................     1
Gi3_27447943_x7.....................................................................uucgauucNcggcgccggagc......................     1
Gi3_27422820_x7.................uccggcguaguauUacggcua..........................................................................     1
Gi3_27425942_x7....................................................................guucgauucccggcgccggagcgC....................     1
Gi1_3960671_x7....................................................................guucCauucccggcgccgg.........................     1
Gi3_27448888_x7...................................................................gguucgauucccggcgccggagG......................     1
Gi1_3972865_x7.............................................ugguucucacccaCcagauccg.............................................     1
Gi2_10524779_x7...................................................................gguucgauucccggUgccggag.......................     1
Gi3_27393791_x7.................uccgCcguaguauaacggc............................................................................     1
Gi1_3958641_x7...................................................................gguucgauucccggcAccggagc......................     1
Gi2_10509813_x7....................................................................guuUgauucccggcgccgga........................     1
Gi3_27414959_x7...................................................................gguucgCuucccggcgccg..........................     1
Gi3_27382605_x7.....................................................................uucgauucccggcgccAgagc......................     1
Gi3_27405824_x7...................................................................gguucgauucccggcgGcgga........................     1
Gi3_27412663_x7.................uccggcguagAauaacgg.............................................................................     1
Gi5_8521813_x7...................................................................gguucgauCcccggcgccg..........................     1
Gi5_8529464_x7...................................................................Uguucgauucccggcgccggagcguuu..................     1
Gi3_27395842_x7.................ucNggcguaguauaacggcuaga........................................................................     1
Gi5_8506868_x7...................................................................gguucgCuucccggcgccgga........................     1
Gi2_10535055_x7......................................................................ucgauuUccggcgccggagc......................     1
Gi3_27390683_x7...............................................Cuucucacccaacagauccg.............................................     1
Gi5_8516486_x7....................................................................guucgauucccggcgccggC........................     1
Gi1_3958452_x7................................................uucucacccaacagaucAgg............................................     1
Gi3_27420545_x7...................................................................gguucgauGcccggcgccggagc......................     1
```

Gi5_8511397_x7..gguuUucacccaacagauccg...   1
Gi5_8504460_x7...gguuUgauucccggcgccg..........................   1
Gi3_27422295_x7.................uccggcguaAuauaacgg...   1
Gi3_27408729_x7...ugguucCcacccaacagauccg...   1
Gi3_27381044_x7..guucgauucccggcgcAggag.......................   1
Gi5_8507288_x7...guucucacccaacagauAcg...   1
Gi1_3967475_x7...ggCucgauucccggcgccgga........................   1
Gi5_8507155_x7..Gcgauucccggcgccggagc......................   1
Gi3_27405733_x7..guucgaGucccggcgccgg.........................   1
Gi2_10503198_x7..gguucucacccaacagaucUg...   1
Gi3_27434055_x7...gguucgauucccggcgccggagcg.....................   0
Gi5_8507673_x7..guucAauucccggcgccggagc......................   1
Gi3_27431339_x7..uGgguucucacccaacagaucc..   1
Gi5_8527399_x7.................uccggcguaguauaacggcCagaa...   1
Gi3_27398712_x7..uucucacccaacGgauccgg..   1
Gi2_10527012_x7.................uccggcguaguauaaUggc..   1
Gi3_27396451_x7..guucgCuucccggcgccg..........................   1
Gi5_8521729_x7...gCuucgauucccggcgccggag.......................   1
Gi1_3968938_x7.................uccggcguaguauaacggcCaga..   1
Gi5_8503200_x7..guucgCuucccggcgccg..........................   1
Gi1_3971206_x7..uAcucacccaacagauccgg..   1
Gi5_8516479_x7...guucucacccaacagGuccgg..   1
Gi3_27407679_x7...gguucgauucccggcgcGggag.......................   1
Gi3_27431668_x7..uucucaccAaacagauccg...   1
Gi5_8506252_x7...gguucgauucccggcgccgAagc......................   1
Gi5_8528162_x7.................uccgUcguaguauaacggcuagaau..   1
Gi1_3967720_x7.................uccggcguaguauaacggUua..   1
Gi3_27426285_x7..guucgauuccUggcgccg..........................   1
Gi5_8527224_x7.................uccAgcguaguauaacggcuaga..   1
Gi3_27416478_x7.................uccggcguaguauaacgAcuagaa...   1
Gi2_10517688_x7...gguucgauCcccggcgccggag.......................   1
Gi2_10537610_x7.................uUcggcguaguauaacggcuag...   1
Gi1_3961238_x7...guCcucacccaacagauccgg..   1
Gi3_27444233_x7..guucgauucAcggcgccgga........................   1
Gi3_27445290_x7..guucgauucccggcgccCga........................   1
Gi2_10535097_x7.................uccggcguaguauaacggcCagaau..   1
Gi3_27436547_x7.................uccggcguaAuauaacggcua..   1
Gi2_10499642_x7...Gucgauucccggcgccggagc......................   1
Gi3_27441874_x7..guucAauucccggcgccg..........................   1
Gi5_8510725_x7...ugguucucacccaacGgauccg...   1
Gi5_8508282_x7...gguucgauucccgAcgccgga........................   1
Gi5_8523577_x7..guucgauucccggcgccUg.........................   1
Gi2_10534873_x7..guucCauucccggcgccg..........................   1
Gi3_27394498_x7...gguucgauucccggAgccgg.........................   1
G1a_5682051_x7.................uccggcguGguauaacggcua..   1
Gi1_3960720_x7.................uccggcguagCauaacggcua..   1
Gi2_10499586_x7...gguucgauucccggcgUcggag.......................   1

```
Gi1_3968056_x7................................................uucucacccaacCgauccgg............................................	1
Gi3_27379581_x7....................................................................gguucgauucccggcgUc..........................	1
Gi3_27423296_x7.....................................................................guuUgauucccggcgccg.........................	1
G1a_5701007_x7....................................................................gguucgauucccggUgcc..........................	1
Gi3_27436477_x7.................uccggcguaguaCaacggcuagaau......................................................................	1
Gi3_27407336_x7.................uccggcguaUuauaacggcuaga........................................................................	1
Gi2_10516162_x7....................................................................Cguucgauucccggcgccgg........................	1
Gi2_10525864_x7.................uccggcgCaguauaacggc............................................................................	1
Gi1_3959887_x7.................uccggcguaguauaacggcuagC........................................................................	1
Gi2_10514335_x7.................uccggcguNguauaacgg.............................................................................	1
Gi1_3967965_x7.....................................................................guucgauucccggcgccUg........................	1
Gi3_27446438_x7.....................................................................guucgauuAccggcgccgg........................	1
Gi3_27456651_x7.................uccAgcguaguauaacggcuagaau......................................................................	1
Gi5_8521162_x7....................................................................gguucgauucccggcgccggUgc.....................	1
Gi1_3972557_x7.................uccggcguaguauaacAgc............................................................................	1
Gi3_27411417_x7.....................................................................guucgauucAcggcgccggagc.....................	1
Gi1_3961497_x7....................................................................gUucgauucccggcgcc...........................	1
Gi2_10522161_x7....................................................................gguCcgauucccggcgccg.........................	1
Gi2_10508679_x7.................uccggcguNguauaacggcuag.........................................................................	1
Gi3_27393896_x7.............................................ugguCcucacccaacagauccg.............................................	1
Gi1_3973243_x7................................................uucucacccaacagauccAg............................................	1
Gi5_8527161_x7...................................................................Ugguucgauucccggcgccggagc.....................	1
Gi5_8512076_x7..............................................gguuGucacccaacagauccg.............................................	1
Gi2_10529735_x7.....................................................................guucUauucccggcgccggag......................	1
Gi5_8519342_x7.............................................ugguucucacccaCcagauccg.............................................	1
Gi1_3968819_x7...............................................guGcucacccaacagauccg.............................................	1
Gi1_3958823_x7................................................uucucacccaacagaCccg.............................................	1
Gi1_3962540_x7....................................................................gguAcgauucccggcgccgg........................	1
Gi5_8530185_x7....................................................................Uguucgauucccggcgccggagcguu..................	1
Gi2_10520985_x7..............................................gCuucucacccaacagauccg.............................................	1
Gi2_10520656_x7................................................uucucacccaacCgauccg.............................................	1
Gi5_8507519_x7.................uccggcguaguauaGcggcuagaa.......................................................................	1
Gi2_10522343_x7......................................................................uGcgauucccggcgccggagc.....................	1
Gi3_27411676_x7.................uccggcguaguauGacggcua..........................................................................	1
Gi1_3967139_x7................................................uucucacccaacagaAccg.............................................	1
Gi3_27432172_x7................................................uucucacccaacagaucUg.............................................	1
Gi3_27386308_x7....................................................................gguucgauucccggcUccggagc.....................	1
Gi1_3961980_x7....................................................................gguuGgauucccggcgccgg........................	1
Gi1_3959257_x7.............................................uCguucucacccaacagauccg.............................................	1
Gi5_8515569_x7.....................................................................guucgauucccggUgccgga.......................	1
Gi5_8518824_x7.................uccggcguaguCuaacggcuagaau......................................................................	1
Gi4_526928_x7 .................uccggcguaguauaacgg.............................................................................	0
Gi3_27386826_x7...............................................guucucacccaacagaucUg.............................................	1
Gi2_10501994_x7.....................................................................guucgCuucccggcgccgg........................	1
Gi5_8530850_x7.............................................ugguucucaccUaacagauccg.............................................	1
Gi4_528594_x7 .................uccggcguaguauaacggcuGga........................................................................	1
Gi5_8523892_x7.....................................................................guucgauucccggcgccAgagc.....................	1
```

```
Gi2_10502547_x7.................uccggcguaguauaacggcuagCau.....................................................................    1
Gi5_8529632_x7...................................................................ggAucgauucccggcgccgg........................    1
Gi3_27447873_x7...................................................................gguucgauucccggcgccgCa.......................    1
Gi2_10534418_x7.................uccggcguaguaCaacggcua.........................................................................    1
Gi1_3972046_x7................................................uucucacccaGcagauccgg...........................................    1
Gi3_27381107_x7...................................................................gguucgauucccggcgUcgg........................    1
G1a_5687742_x7.................................................Gcucacccaacagauccg............................................    1
Gi3_27402597_x7................................................uucucacccaacagauccgC...........................................    1
Gi2_10515833_x7...................................................................gguucgauuccUggcgccg.........................    1
Gi5_8530052_x7.................uccggcguaguauaacggGuag........................................................................    1
Gi2_10530001_x7.................uccggcguaguauaCcggcuaga.......................................................................    1
Gi5_8526216_x7..................................................cucacUcaacagauccgg...........................................    1
Gi2_10508350_x7...................................................................gguucgauucccUgcgccggag......................    1
Gi5_8512580_x7...................................................................gguucgGuucccggcgccggag......................    1
Gi2_10509617_x7....................................................................guucgauuccUggcgccgga.......................    1
Gi2_10512956_x7.................uccggcguaguauaacggcuaA........................................................................    1
Gi5_8521939_x7....................................................................guucgauucccggcgGcg.........................    1
Gi2_10507881_x7....................................................................guCcgauucccggcgccgga.......................    1
Gi3_27419789_x7.................uccCgcguaguauaacggcuaga.......................................................................    1
Gi2_10516757_x7..............................................gguucucacccaacagCuccg............................................    1
Gi5_8528449_x7...................................................................gguucgauucccAgcgcc..........................    1
Gi1_3973075_x7..................ccggcguaguauaacggcuagaau.....................................................................    0
Gi3_27451051_x7...................................................................gguCcgauucccggcgcc..........................    1
Gi3_27423527_x7.......................................................................cgauucccNgcgccggagc.....................    1
Gi5_8511859_x7...................................................................gguucgauucccggcgAcg.........................    1
Gi5_8505811_x7....................................................................guucgauucccgAcgccg.........................    1
Gi2_10514692_x7....................................................................guuAgauucccggcgccgg........................    1
Gi5_8519272_x7...................................................................gguCcgauucccggcgccgg........................    1
Gi5_8514484_x7...................................................................gguucgauucccggcAccg.........................    1
Gi5_8516521_x7.................uccggcguaguCuaacggc...........................................................................    1
Gi3_27453277_x7...................................................................gguucgauucUcggcgccgg........................    1
Gi3_27446081_x7...................................................................gguucgauuccAggcgcc..........................    1
Gi3_27435574_x7.................uccggcguagCauaacggcuag........................................................................    1
Gi2_10523505_x7.....................................................................uGcgauucccggcgccgg........................    1
Gi3_27427111_x7....................................................................guucgauucccggcgcGgagc......................    1
Gi3_27442532_x7...................................................................gguucgauucAcggcgccg.........................    1
Gi3_27437044_x7.................uccggcguaguUuaacgg............................................................................    1
Gi3_27416835_x7.................uccggAguaguauaacggcuagaa......................................................................    1
Gi3_27418193_x7...................................................................Nguucgauucccggcgccggagc.....................    1
G1a_5688204_x7...................................................................gGucgauucccggcgccggagc......................    1
Gi2_10508567_x7....................Ugcguaguauaacggcuaga.......................................................................    1
Gi5_8545291_x6..................................................Uucacccaacagauccgg...........................................    1
Gi3_27518824_x6.................uccggcgCaguauaacggcuag........................................................................    1
Gi5_8542951_x6..................................................cucacccaacagaCccgg...........................................    1
Gi5_8538079_x6................................................Cucucacccaacagauccgg...........................................    1
Gi2_10570907_x6.................uccgUcguaguauaacggcuag........................................................................    1
Gi1_3979981_x6..............................................gguucucacccUacagauccg............................................    1
```

```
Gi5_8536669_x6.................uccggcguaAuauaacggcua..........................................................................      1
Gi5_8537881_x6.................uccggcguaguauaacgAcuagaau......................................................................      1
Gi3_27521662_x6...................................................................gguucgGuucccggcgcc...........................      1
Gi3_27462982_x6..................ccggcguaguauaacggcua..........................................................................      0
Gi2_10548773_x6................................................uucucacccaacagaCccg.............................................      1
Gi2_10569563_x6....................................................................guucgauucccggcgccggC........................      1
Gi5_8532547_x6...................................................................gguuUgauucccggcgccggagcguu...................      1
Gi5_8558725_x6...................................................................gguucgauucccUgcgccgg.........................      1
Gi2_10545881_x6....................................................................guucgauucccggcgUcg..........................      1
Gi5_8553223_x6.................ucAggcguaguauaacggcuag.........................................................................      1
Gi5_8536429_x6...............................................guCcucacccaacagauccgg............................................      1
Gi5_8554213_x6.................uccggcguaguauUacggcuag.........................................................................      1
Gi2_10585175_x6...................................................................gguucgauucccggcgcU...........................      1
Gi2_10545575_x6..............................................gguucucacccCacagauccg.............................................      1
Gi5_8546413_x6...............................................guucucaccAaacagauccg.............................................      1
Gi1_3990703_x6..............................................gguucucacUcaacagauccg.............................................      1
Gi1_3984469_x6....................................................................guuUgauucccggcgccggagc......................      1
Gi3_27491716_x6................................................uucucacccaacagauccgA............................................      1
Gi3_27532678_x6................................................uucucacccaacagaucUgg............................................      1
Gi1_3987019_x6...............................................guucucacccaacCgauccg.............................................      1
Gi1_3975613_x6.............................................ugguucucacccaacagaucUgg............................................      1
Gi5_8536891_x6...................................................................gguucAauucccggcgccgg.........................      1
Gi5_8550865_x6....................................................................guucgaGucccggcgccg..........................      1
Gi5_8554297_x6...................................................................gguucgauucccggcgccggaAc......................      1
Gi5_8534767_x6....................................................................guucgUuucccggcgccgg.........................      1
Gi5_8535937_x6.................uccggcguaguauaaGggcuaga........................................................................      1
Gi1_3987505_x6..................Gcggcguaguauaacggcuaga........................................................................      1
Gi1_3978553_x6..............................................ggAucucacccaacagauccg.............................................      1
Gi3_27541582_x6.....................................................................uucgauucNcggcgccgga........................      1
Gi1_3980713_x6...................................................Gcacccaacagauccgggu..........................................      1
Gi3_27538150_x6.................uccAgcguaguauaacggcuagaa.......................................................................      1
G1a_5705266_x6.................uccggcguaguauCacggcuaga........................................................................      1
Gi3_27524956_x6..............................................gAuucucacccaacagauccg.............................................      1
Gi2_10576511_x6...................................................................gCuucgauucccggcgccgga........................      1
Gi1_3993481_x6....................................................................guucgauucccggcgUcg..........................      1
Gi5_8555773_x6...................................................................gguucgauucccggcAccggag.......................      1
Gi2_10568933_x6...................................................................gguucgauucccggGgcc...........................      1
Gi5_8545813_x6..................Ucggcguaguauaacggcuaga........................................................................      1
Gi3_27467050_x6...............................................guGcucacccaacagauccg.............................................      1
Gi5_8550685_x6...............................................................ucUggguucgauucccggc..............................      1
Gi5_8531065_x6.......................Uuaguauaacggcuagaau......................................................................      1
Gi2_10551671_x6.................uccgUcguaguauaacggcuagaa.......................................................................      1
Gi3_27468004_x6..............................................gguucucacccaacagauccUggu..........................................      1
Gi5_8549311_x6...................................................................gguuAgauucccggcgccggagc......................      1
Gi1_3990541_x6.............................................ugguuAucacccaacagauccgg............................................      1
Gi1_3992857_x6.............................................ugguuUucacccaacagauccg.............................................      1
Gi1_3992917_x6......................................................................ucgauuAccggcgccggagc......................      1
```

Gi3_27478312_x6..uAcucacccaacagauccg... 1
Gi5_8557789_x6..gguucucacccaacagauccA... 1
Gi5_8560399_x6...ugguucCcacccaacagauccg... 1
Gi3_27465052_x6..guucgauucccggcgccggU........................ 1
Gi3_27477280_x6..Ugguucgauucccggcgccg.......................... 1
Gi5_8533417_x6..uucucacAcaacagauccg... 1
Gi2_10554221_x6.................uccggcguaguauaGcggcuag... 1
Gi1_3976795_x6..gguucucacccaacagauccA... 1
Gi5_8544061_x6.................uccggcguaguaCaacggcuagaau.. 1
Gi2_10551461_x6..gguucucacccaacGgauccg... 1
Gi2_10580399_x6..guucgauucccggcgcAg.......................... 1
Gi1_3989029_x6...ugguucucaUccaacagauccgg.. 1
Gi3_27521374_x6..guucgauucccggcCccgga........................ 1
Gi3_27495448_x6..cCcacccaacagauccgg.. 1
Gi5_8547775_x6..guucgauAcccggcgccgg......................... 1
Gi5_8542771_x6...guAcucacccaacagauccg... 1
Gi1_3980503_x6...gguucgauCcccggcgccggagc...................... 1
Gi1_3983311_x6.................uccggcguaguauaGcggcuaga.. 1
Gi2_10562117_x6...gguucgauucccAgcgccg.......................... 1
Gi5_8531629_x6...guucucacccUacagauccg... 1
Gi1_3988279_x6...Gucgauucccggcgccggagc...................... 1
G1a_5719582_x6..Uucgauucccggcgccg........................... 1
Gi5_8535979_x6...uAguucucacccaacagauccg... 1
Gi2_10586483_x6...guucCcacccaacagauccg... 1
Gi5_8534227_x6.................uccggcguaguauaaAggcuaga.. 1
Gi3_27515596_x6..gauucccggGgccggagc...................... 1
Gi1_3991123_x6.................uccggcguaguauaUcgg... 1
Gi3_27536362_x6..guucgauucccggcgccUg......................... 1
Gi3_27531646_x6...guucucacccaacagauAcg... 1
Gi5_8548093_x6..guucgauucccggcAccgga........................ 1
Gi1_3990955_x6.................uccggcguaguaCaacggcuaga.. 1
Gi2_10585691_x6...gguucgauucccggcgcAgga........................ 1
Gi1_3989257_x6.................uccggcguaguauaacAgcuagaau.. 1
Gi5_8555047_x6.................uccggcguaguauaacUgcuaga.. 1
Gi1_3985957_x6...Agguucucacccaacagauccgg.. 1
Gi3_27527164_x6...gguucgauucccgAcgccgg......................... 1
Gi3_27501688_x6..uGgauucccggcgccgga........................ 1
Gi1_3985681_x6..guNcgauucccggcgccgg......................... 1
Gi5_8550985_x6...gguucgauucccggcgcUgg......................... 1
Gi2_10542875_x6..guucgauucccggcgcUgga........................ 1
Gi1_3992587_x6..guAcgauucccggcgccgga........................ 1
Gi3_27478264_x6..guucUauucccggcgccg.......................... 1
Gi3_27484126_x6....................Ugcguaguauaacggcua.. 1
Gi1_3991351_x6..gauucccggcgccggagc...................... 0
Gi5_8545987_x6...gguAcgauucccggcgccggagc...................... 1
Gi2_10566947_x6.................uccggcguaguauaacggcuagaC... 1
Gi3_27511738_x6..guucgauucccggcAccg.......................... 1

```
Gi5_8531137_x6....................................................................gguucgGuucccggcgccgga........................    1
Gi2_10588361_x6..................uccggcguaguauCacggcuagaau......................................................................    1
Gi1_3990067_x6..................uccggcguaguauaacggcuagGau......................................................................    1
Gi2_10581317_x6..................Cccggcguaguauaacggcuagaau......................................................................    1
Gi3_27511798_x6.....................................................................guucgauucccggcgUcggag.......................    1
G1a_5716528_x6..................Gccggcguaguauaacgg.............................................................................    1
Gi2_10574453_x6....................................................................gguCcgauucccggcgccgg.........................    1
Gi5_8558587_x6...................................................cucacccaCcagauccgg............................................    1
Gi3_27525706_x6.....................................................................guucgauucccUgcgccgg.........................    1
Gi5_8538685_x6..................uccAgcguaguauaacggcuagaau......................................................................    1
Gi2_10565009_x6....................................................................gguucgauucccUgcgccgga........................    1
Gi5_8542441_x6..................uccgAcguaguauaacggcuag.........................................................................    1
Gi1_3975235_x6.....................................................................guucgauucccggcgccggCgc......................    1
Gi3_27533494_x6..................uccggcgGaguauaacggcua..........................................................................    1
Gi2_10575779_x6.....................................................................gAucgauucccggcgccgga........................    1
Gi2_10546109_x6....................................................................gguucgauucccggcgccUgagc......................    1
Gi5_8561119_x6.....................Ugcguaguauaacggcuagaau......................................................................    1
Gi1_3991867_x6.....................................................................guucgauucccggUgccgga........................    1
G1a_5726536_x6..................uccggcguaguauaacggcuagG........................................................................    1
Gi3_27488302_x6.......................................................................ucgauucccggcgccggCgc......................    1
Gi3_27535378_x6....................................................................gguuGgauucccggcgccgg.........................    1
Gi5_8544295_x6.......................................................ccUaacagauccggguuc........................................    1
Gi2_10578197_x6..................uccggcguaguaCaacgg.............................................................................    1
Gi5_8544565_x6..................uccggcguaguauaacggcAag.........................................................................    1
Gi1_3990013_x6..................uccggcguaguauaacggcuagaaC......................................................................    1
Gi2_10590599_x6....................................................................gguucgauucccUgcgccg..........................    1
Gi2_10566755_x6.....................................................................guucgauucccUgcgccggagc......................    1
Gi3_27527308_x6..................uccggcguaAuauaacggcuagaau......................................................................    1
Gi5_8543557_x6...................................................cuUacccaacagauccgg............................................    1
Gi1_3977671_x6................................................guucucacccaacagauAcgg............................................    1
Gi3_27519514_x6.............................................uugguCcucacccaacagaucc..............................................    1
Gi2_10558439_x6........................................................................cgauucccggcgccggagcguuG..................    1
Gi3_27509530_x6...........................................................acagauccggguucgauucccggcgccggagcC.....................    1
Gi1_3987037_x6...............................................gguucucacccaacagauccgU............................................    1
Gi5_8544787_x6................................................guucucacccaacagCuccg.............................................    1
Gi1_3980473_x6..................uccggcguaguGuaacggcu...........................................................................    1
Gi3_27475666_x6..............................................ugguucucGcccaacagauc...............................................    1
Gi5_8556079_x6...............................................gguucucacccaacagauUcg.............................................    1
Gi1_3978493_x6..............................................ugguucucacccaacagCuccgg............................................    1
Gi2_10551425_x6........................................................................cgauucccggcgccggagc......................    0
Gi2_10574765_x6....................................................................gguucgauucccggcgcA...........................    1
Gi3_27465964_x6...................ccggcguaguauaCcggc............................................................................    1
Gi3_27544918_x6..................uccggcAuaguauaacggc............................................................................    1
Gi2_10578713_x6............................................................Gagauccggguucgauucc..................................    1
Gi5_8551537_x6....................................................................gguucgauucccAgcgccgg.........................    1
Gi5_8562907_x6..................uccUgcguaguauaacggcuag.........................................................................    1
Gi2_10583597_x6....................................................................gguCcgauucccggcgccgga........................    1
```

```
Gi1_3980131_x6...............................................guucucacccGacagauccg.............................................     1
Gi5_8548669_x6...................................................................gguuUgauucccggcgccgga........................     1
Gi5_8539447_x6...............................................guucucaUccaacagauccg.............................................     1
Gi3_27510196_x6...................................................................gguucgauucccAgcgcc...........................     1
Gi3_27459700_x6.................uccggcguagCauaacggcuagaau......................................................................     1
Gi2_10543823_x6.................Cccggcguaguauaacggcua..........................................................................     1
Gi3_27509182_x6.................uccggcguaguUuaacggcua..........................................................................     1
Gi2_10574855_x6.................uccggcguaguauaaAggcuaga........................................................................     1
Gi3_27463690_x6...................................................................gguucgaAucccggcgccgga........................     1
Gi5_8547289_x6...................................................................gguucgauucccggAgccg..........................     1
Gi1_3986503_x6...................................................................Aguucgauucccggcgccg..........................     1
Gi5_8548675_x6....................................................................guucgauucccggGgccg..........................     1
Gi5_8562469_x6...............................................guucucacccaacagauUcg.............................................     1
Gi4_530649_x6 ....................................................................Uuucgauucccggcgccgga........................     1
Gi2_10578299_x6.......................................................................cUauucccggcgccggagcguuuc.................     1
Gi1_3984025_x6....................................................................guucgauuNccggcgccgga........................     1
Gi3_27490564_x6..............................................gguucucacccaacCgauccg.............................................     1
Gi1_3985861_x6....................................................................guCcgauucccggcgccggagc......................     1
Gi5_8543503_x6...................................................................gguucgauAcccggcgccggagc......................     1
Gi3_27484798_x6.....................................................................uCcgauucccggcgccggga.......................     1
Gi1_3975169_x6....................................................................Cuucgauucccggcgccg..........................     1
Gi5_8561839_x6...................................................................gCuucgauucccggcgccgga........................     1
Gi2_10558097_x6...................................................................gguucgauucccggcgcUggag.......................     1
Gi1_3980905_x6...............................................guucucacccaacagaucUg.............................................     1
Gi2_10553141_x6.................uccggcAuaguauaacggcuaga........................................................................     1
Gi3_27493318_x6...............................................guucucacccaacagaucAg.............................................     1
Gi1_3981253_x6....................................................................guuGgauucccggcgccgg.........................     1
Gi3_27491776_x6.................uccggGguaguauaacgg.............................................................................     1
Gi1_3976639_x6.................uccggcguGguauaacggcu...........................................................................     1
Gi2_10563647_x6...................................................................gguuUgauucccggcgccggag.......................     1
Gi3_27541906_x6.................uAcggcguaguauaacgg.............................................................................     1
Gi2_10569353_x6......................................................cUcaacagauccggguuc........................................     1
Gi2_10541777_x6..............................................gguucucGcccaacagauccg.............................................     1
Gi1_3980479_x6....................................................................guucgauucccggcgccA..........................     1
Gi3_27509242_x6.........................................................aacagauccggguucgauucccggcgccggagc......................     0
Gi5_8531617_x6..............................................gguucucacccaUcagauccg.............................................     1
Gi2_10570739_x6.................Accggcguaguauaacggc............................................................................     1
Gi3_27547384_x6................................................uucAcacccaacagauccgg............................................     1
Gi5_8533591_x6.................uccggcguaguauaacggcuGgaa.......................................................................     1
Gi5_8556055_x6.................uccggcAuaguauaacggcuag.........................................................................     1
Gi5_8548897_x6.................uccggcguaguGuaacgg.............................................................................     1
Gi5_8542105_x6...............................................guucucacccaCcagauccgg............................................     1
Gi5_8544871_x6...............................................Auucucacccaacagauccgg............................................     1
Gi1_3986059_x6....................................................................guucgauucccggcgccgU.........................     1
Gi1_3992161_x6....................................................................guucgauucccggcAccgga........................     1
G1a_5723356_x6.................uccggcguagCauaacggcuaga........................................................................     1
Gi1_3986179_x6.................uccggUguaguauaacggcuagaau......................................................................     1
```

Gi2_10567661_x6..guucgauCcccggcgccg.......................... 1
Gi2_10548545_x6..guucgauuccAggcgccggagc...................... 1
Gi2_10549547_x6...gguucgauucccggcgccggaA....................... 1
Gi1_3989989_x6...ugguucucacccaacagauccAg.. 1
Gi3_27509992_x6..guucgauuccAggcgccggagc...................... 1
Gi2_10547255_x6...............Gguccggcguaguauaacgg... 1
Gi3_27482182_x6...gguucgauucccggcgccgU......................... 1
Gi3_27476716_x6.................uccggcguaguauaacggUuagaau.. 1
Gi2_10586393_x6..uucucacccGacagauccg... 1
Gi5_8555503_x6..guucgauucccAgcgccggagc...................... 1
Gi5_8535757_x6...Cguucgauucccggcgccggag....................... 1
Gi1_3993703_x6.................uccggcguaguauaacggcuagaGu.. 1
Gi1_3978925_x6...uGucacccaacagauccg... 1
Gi5_8531353_x6...gguucgauucAcggcgccggagc...................... 1
Gi3_27542590_x6..ucgauucccggcgccggGgc...................... 1
Gi2_10547435_x6.................uccggcguaguauaacggU.. 1
Gi2_10576583_x6...ggAucgauucccggcgccggag....................... 1
Gi3_27481582_x6...............Gguccggcguaguauaacggcuagaa... 1
Gi1_3975787_x6.................uccgAcguaguauaacggcua.. 1
Gi5_8552635_x6.................uccggUguaguauaacggcuagaa... 1
Gi1_3983947_x6..gguucucacccaacagaucUg... 1
Gi3_27498148_x6..Cucucacccaacagauccgg.. 1
Gi3_27531694_x6..guuGgauucccggcgccggagc...................... 1
Gi2_10589573_x6..guucgauucccgUcgccgg......................... 1
Gi3_27520810_x6.................uccggcguaguauaacggcAaga.. 1
Gi5_8563561_x6...gguuUgauucccggcgccggag....................... 1
Gi2_10564247_x6...guCcucacccaacagauccg... 1
Gi1_3976723_x6...guucCcacccaacagauccgg.. 1
Gi2_10556015_x6..guucgauucccggcAccggag....................... 1
Gi1_3988993_x6...gguucgauucccggcgccCg......................... 1
Gi2_10549907_x6..guucgauucccgAcgccgga........................ 1
Gi5_8533621_x6..guucgauCcccggcgccgga........................ 1
Gi1_3993187_x6..gCucgauucccggcgccg.......................... 1
G1a_5730928_x6...guucucacccCacagauccg... 1
Gi3_27511150_x6...gguuGgauucccggcgccg.......................... 1
Gi5_8556733_x6...gguucgauuccUggcgccgga........................ 1
Gi3_27487786_x6...gguucUauucccggcgccgga........................ 1
Gi3_27518572_x6...gguucgauuAccggcgccgga........................ 1
G1a_5702458_x6...Cguucgauucccggcgccggag....................... 1
Gi3_27472150_x6..guucgauucccgUcgccggagc...................... 1
Gi5_8532835_x6.................uccggcguCguauaacggcuag... 1
Gi3_27520444_x6..gaCucccggcgccggagc...................... 1
Gi1_3977395_x6...guucucacccaacagauAcg... 1
Gi5_8560951_x6..uucucacccaacagauAcg... 1
Gi3_27515602_x6...Gcucacccaacagauccgg.. 1
Gi5_8549155_x6...ucucGcccaacagauccg... 1
Gi3_27534442_x6.................uccggcguaguCuaacgg... 1

```
Gi3_27507706_x6....................................................................guAcgauucccggcgccg..........................     1
Gi2_10580027_x6......................................................................Gcgauucccggcgccggag.......................     1
Gi3_27459346_x6...................................................................gguucgauuccGggcgccggag.......................     1
Gi5_8532871_x6...................................................................gguucgauucccggcgcAg..........................     1
Gi2_10574429_x6......................................................................Ccgauucccggcgccggagc......................     1
Gi2_10587971_x6...................................................................gguucgauucAcggcgccggag.......................     1
Gi1_3990205_x6...................................................................Aguucgauucccggcgccggag.......................     1
Gi5_8542753_x6.................uccggcguUguauaacggcuaga........................................................................     1
Gi5_8533807_x6...................................................................gguucgauucccggcgccgAa........................     1
Gi5_8562223_x6....................................................................guucgaGucccggcgccgg.........................     1
Gi5_8554093_x6.............................................uUguucucacccaacagauccgg............................................     1
Gi3_27548074_x6.......................................................................cgauucccggcgccAgagc......................     1
Gi3_27470788_x6...................................................................gguucgaGucccggcgccggagc......................     1
Gi2_10566929_x6...............................................guucucacccaacGgauccg.............................................     1
Gi5_8551669_x6.............................................ugguucucacccaacagaucUg.............................................     1
Gi3_27539296_x6..................Gcggcguaguauaacggc............................................................................     1
Gi3_27517384_x6.................uccgAcguaguauaacggc............................................................................     1
Gi2_10561487_x6....................................................................guucUauucccggcgccgga........................     1
Gi5_8537029_x6.................uccggcguaguauaacggcuUg.........................................................................     1
Gi3_27489004_x6.............................................ugguucucNcccaacagauc...............................................     1
Gi3_27521212_x6.................ucAggcguaguauaacgg.............................................................................     1
Gi2_10580909_x6.................uccggcguaguauaUcggcuagaau......................................................................     1
Gi1_3978103_x6...................................................................Cguucgauucccggcgccggagc......................     1
Gi2_10544189_x6...................................................................gguuGgauucccggcgccg..........................     1
Gi5_8562331_x6....................................................................guucgauucccCgcgccg..........................     1
Gi5_8560213_x6...................................................................gUuucgauucccggcgccggagcguuu..................     1
Gi5_8541073_x6....................................................................guucgauucccgUcgccg..........................     1
Gi3_27484174_x6...................................................................gguucgauucccggcgccU..........................     1
Gi3_27525220_x6.....................................................................uucgauucccggcgccggC........................     1
Gi5_8563153_x6.............................................ugguucucacccGacagauccg.............................................     1
Gi2_10567193_x6...................................................................gguucgauucccggcgAcggag.......................     1
Gi3_27501868_x6...................................................................gguucgauucccggGgccggag.......................     1
Gi3_27523864_x6...................cUgcguaguauaacggcuagaau......................................................................     1
Gi3_27469624_x6...........................................................Gagauccggguucgauuccc.................................     1
Gi3_27494680_x6................................................uucucacAcaacagauccgg............................................     1
G1a_5726860_x6.................ucUggcguaguauaacggcuaga........................................................................     1
Gi2_10588049_x6...............................................guucucacAcaacagauccg.............................................     1
Gi5_8554057_x6...................................................................gguGcgauucccggcgccg..........................     1
Gi3_27464092_x6....................................................................guucgauucccggcgcAg..........................     1
Gi1_3982381_x6....................................................................guucgauuccUggcgccg..........................     1
Gi2_10569701_x6...................................................................gguucgauucccggcAccg..........................     1
Gi1_3999010_x5.................uccggcguaguauaaUggcuag.........................................................................     1
Gi3_27595842_x5...................................................................gguucgUuucccggcgccgga........................     1
Gi2_10631330_x5...................................................................gguucgauucccggGgccg..........................     1
Gi2_10606795_x5...................................................................gguucgauuccUggcgccgga........................     1
Gi2_10623935_x5.................uccggcguagCauaacggcuagaa.......................................................................     1
Gi1_4008040_x5....................................................................guucgauucccggcgGcggagc......................     1
```

```
Gi2_10627080_x5....................................................................guucgauucccggAgccg..........................     1
Gi1_4004905_x5....................gUcguaguauaacggcua..........................................................................     1
Gi1_4006725_x5................................................uucAcacccaacagauccg.............................................     1
Gi1_4011145_x5.............................................uggCucucacccaacagauccg.............................................     1
Gi2_10605435_x5......................................................................ucgauuccNggcgccggag.......................     1
Gi3_27581747_x5...................................................................gguucgauucccggcgcAgg.........................     1
Gi1_3999765_x5.................uccggcguaguauaacggcuCg.........................................................................     1
Gi1_4010535_x5.............................................ugguucucacccaacUgauccgg............................................     1
Gi1_4007875_x5................................................uucucacccaacagaCccgg............................................     1
Gi1_4016545_x5...............................................guucucacccaacagaCccg.............................................     1
Gi1_4002010_x5.................uccggcguaguauaacgAc............................................................................     1
Gi3_27574422_x5.....................................................................ucgauucccggUgccggagc.......................     1
Gi3_27581737_x5....................................................................guucgauucccggcgAcggag.......................     1
Gi3_27599387_x5.....................................................................ucgauucccggcgccggG.........................     1
Gi2_10645415_x5....................................................................guucAauucccggcgccg..........................     1
Gi3_27604427_x5................guccggcguaguauaacggcua..........................................................................     0
Gi3_27640892_x5................................................uucucacccUacagauccgg............................................     1
Gi5_8580147_x5..............................................gguucucaAccaacagauccg.............................................     1
Gi5_8597617_x5...................................................................gguucgauucccAgcgccggag.......................     1
Gi5_8592172_x5...................................................................gguucgauucccggcgccggU........................     1
Gi3_27583507_x5...............................................guucucacAcaacagauccg.............................................     1
Gi3_27559677_x5.................uccggcguUguauaacggcuagaa.......................................................................     1
Gi3_27549482_x5...................................................................gCuucgauucccggcgcc...........................     1
Gi2_10628225_x5...................................................................gguucgaGucccggcgccggagc......................     1
Gi1_4017635_x5....................................................................guucgauucccggcgccggC........................     1
Gi3_27636442_x5...............................................Cuucucacccaacagauccgg............................................     1
Gi3_27619477_x5................................................Aucucacccaacagauccg.............................................     1
Gi1_4004260_x5.............................................ugguucucacccaacagaucAgg............................................     1
Gi5_8595782_x5...............................................gCucucacccaacagauccgg............................................     1
Gi5_8594042_x5...................................................................gguucgauucccggUgccgga........................     1
Gi1_4013905_x5.................uccggcguaguauaacggcuagaC.......................................................................     1
Gi5_8594797_x5....................................................................guAcgauucccggcgccgga........................     1
Gi5_8571147_x5....................................................................guucgauucccggcgcAgga........................     1
Gi3_27555052_x5............................................................agauccggguucgauucccggcgccgg.........................     0
Gi3_27614012_x5...............................................guucucacccaacGgauccgg............................................     1
Gi5_8590737_x5...................................................................gAuucgauucccggcgccgg.........................     1
Gi2_10623165_x5....................................................................gCucgauucccggcgccgga........................     1
Gi3_27621692_x5...................................................................gguuUgauucccggcgccgg.........................     1
Gi2_10615215_x5...................................................................gguucgauucccgCcgccggagc......................     1
Gi1_4006035_x5...............................................guucucacccaacagaucUgg............................................     1
Gi1_4004885_x5................................................uucucacccaacagauUcg.............................................     1
Gi3_27651257_x5...................................................................gguucAauucccggcgccgg.........................     1
Gi3_27640812_x5......................................................cccaacagaGccggguucgauuccc.................................     1
Gi3_27600207_x5.................uccggcguaguauUacggc............................................................................     1
Gi5_8573337_x5...................cUgcguaguauaacggcuagaau......................................................................     1
Gi1_3999595_x5...............................................gAucucacccaacagauccg.............................................     1
G1a_5752502_x5.................uccggcguaguauaGcggcuag.........................................................................     1
```

```
Gi3_27604932_x5.................................................ucucacccaacagaCccg.............................................  1
Gi3_27593912_x5...................................................................gguucgauuUccggcgcc...........................  1
Gi1_4016980_x5.................uccggcguaguauUacgg.............................................................................  1
Gi2_10629720_x5.................uccggcguaguauUacggcuaga........................................................................  1
Gi5_8597282_x5..................................................cucacccaacagCuccgg............................................  1
Gi1_4011795_x5.............................................ugguucucacccaacagGuccg.............................................  1
Gi1_4014305_x5....................................................................guucgauuGccggcgccgg.........................  1
Gi1_4008310_x5...............................................guucuUacccaacagauccg.............................................  1
Gi5_8593517_x5.............................................ugguucucacccaacagaCccg.............................................  1
Gi2_10648380_x5.................Cccggcguaguauaacggcuag.........................................................................  1
Gi1_4016230_x5...........................................Uuugguucucacccaacagauccgggu..........................................  1
Gi1_3995650_x5.................ucGggcguaguauaacgg.............................................................................  1
Gi5_8568342_x5...................................................................gUuucgauucccggcgccggagcguu...................  1
Gi2_10602855_x5....................................................................guuAgauucccggcgccggagc......................  1
Gi2_10594165_x5...................................................................gguucgauuccAggcgcc...........................  1
Gi2_10645020_x5...................................................................gguucgauuUccggcgccggag.......................  1
Gi2_10626655_x5...................................................................gguucgauucccggGgccgga........................  1
Gi2_10642120_x5....................................................................guucgauucccggcgcGggagc......................  1
Gi2_10628090_x5.....................................agaaugguugguCcucacc........................................................  1
Gi5_8577957_x5.................uccggcguaguauaacggAuagaau......................................................................  1
G1a_5753682_x5...................................................................gguuUgauucccggcgccggagc......................  1
Gi1_4011125_x5.................uccggcguaguauaCcgg.............................................................................  1
Gi1_4017165_x5................................................uucucacAcaacagauccgg............................................  1
Gi2_10617425_x5.................uccggcguaguaCaacggc............................................................................  1
Gi2_10617540_x5......................................................................uUgauucccggcgccggagc......................  1
Gi2_10604695_x5...................................................................gguucgauucccggAgccggag.......................  1
Gi2_10643425_x5....................................................................guucgauucccggcgccggaCc......................  1
Gi2_10633100_x5.................uccggcguaguauaaUgg.............................................................................  1
Gi5_8588232_x5....................Ugcguaguauaacggcuagaa.......................................................................  1
Gi5_8579392_x5....................................................................guucgauuAccggcgccgg.........................  1
Gi3_27644187_x5....................................................................guucgUuucccggcgccggag.......................  1
Gi2_10626120_x5...................................................................gguGcgauucccggcgccggagc......................  1
Gi3_27622827_x5...................................................................gguucgauucccggcCccgga........................  1
Gi1_4004970_x5.................uccggcguaguaCaacggcuag.........................................................................  1
Gi1_4005055_x5............................................................agUuccggguucgauuccc.................................  1
Gi3_27601762_x5...................................................................gguucgauucccggGgccgga........................  1
Gi2_10648035_x5.................uccggcguaguauaacggcuagUau......................................................................  1
Gi5_8591757_x5...................................................................gguucgauucccggcAccgg.........................  1
Gi3_27621612_x5.................uccggcguaAuauaacggcuag.........................................................................  1
Gi2_10592560_x5................................................uucucacccaacagauccC.............................................  1
Gi3_27608552_x5.................uccggcgGaguauaacgg.............................................................................  1
Gi3_27586067_x5..................................................................gUguucgauucccggcgccggag.......................  1
Gi3_27588697_x5....................................................................guucgauucccgAcgccggag.......................  1
Gi1_4017240_x5....................................................................guucgauuNccggcgccggagc......................  1
Gi2_10615105_x5............................................................agUuccggguucgauucc..................................  1
Gi5_8596682_x5.................uccggcguaAuauaacggcuag.........................................................................  1
Gi2_10599780_x5...................................................................gguucgauucccggcgcAggag.......................  1
```

```
Gi1_4006810_x5.................uccggcguaguUuaacgg.............................................................................    1
Gi3_27566502_x5..................................................................ggguucgauucccggcgUcg..........................    1
Gi5_8571622_x5...................................................................gguucgauCcccggcgccggag.......................    1
Gi1_4001675_x5.................................................Ccucacccaacagauccgg............................................    1
Gi3_27583602_x5.................uccggcguaguauaacggcuaC.........................................................................    1
Gi3_27641547_x5......................................................................ucUauucccggcgccggag.......................    1
Gi3_27630192_x5.................uccggcguaguauaUcgg.............................................................................    1
Gi5_8590502_x5...................................................................gguucgauucccggcgUcgg.........................    1
Gi2_10623980_x5.................uccggcguUguauaacggcuagaau......................................................................    1
Gi1_4011340_x5...................................................................gguucgCuucccggcgccg..........................    1
Gi5_8585457_x5.................uccggcguaguauaacggcC...........................................................................    1
Gi3_27574032_x5....................................................Gacccaacagauccggguuc........................................    1
Gi3_27551882_x5.................uccggcguaguUuaacggc............................................................................    1
Gi3_27617207_x5..............................................gguucucacccaacagauUcg.............................................    1
Gi5_8576487_x5................................................uuUucacccaacagauccgg............................................    1
Gi1_3996660_x5................................................uucucacccaacagCuccg.............................................    1
Gi2_10598525_x5....................................................................guucgauucccggcgccggUgc......................    1
Gi2_10592830_x5....................................................................guucgUuucccggcgccggagc......................    1
Gi2_10591920_x5...................................................................gguucgauucccggcgcUgg.........................    1
Gi5_8575727_x5..............................................gguucucacccaacagauccggA...........................................    1
Gi1_4014530_x5..............................................gCuucucacccaacagauccg.............................................    1
Gi3_27635172_x5..............................................gguucucacccaacUgauccg.............................................    1
Gi3_27606822_x5.................ucAggcguaguauaacggcuagaa.......................................................................    1
Gi2_10633035_x5....................................................................guAcgauucccggcgccggag.......................    1
Gi2_10636470_x5.................uccCgcguaguauaacggcuaga........................................................................    1
Gi2_10624170_x5...................................................................gguucgauucccggcgccAg.........................    1
Gi1_3995530_x5...................................................................gguucgauucccggcgAcggagc......................    1
Gi1_3998275_x5....................................................................guucgauucccggcgAcgga........................    1
Gi2_10642580_x5....................................................................gAucgauucccggcgccgg.........................    1
Gi1_4011460_x5..........................................................aUagauccggguucgauuccc.................................    1
Gi1_4011240_x5...............................................guucucacccaacagauccggU...........................................    1
Gi3_27642202_x5...................................................................gguAcgauucccggcgccgga........................    1
Gi5_8566407_x5................................................uucucacUcaacagauccg.............................................    1
Gi5_8599527_x5.................uccggcguaguauaCcggcuag.........................................................................    1
Gi1_3994800_x5..................................................cucacccaacagGuccgg............................................    1
Gi3_27566402_x5...............................................guucucacccGacagauccg.............................................    1
Gi2_10603410_x5.................uccggcguaguauaaUggcua..........................................................................    1
Gi1_4012755_x5.....................................................................uGcgauucccggcgccggagc......................    1
Gi5_8592097_x5...................................................................gguuGgauucccggcgccggagc......................    1
Gi4_536278_x5 ...................................................................gguucgauucccggcgccg..........................    0
Gi3_27605667_x5................................................uGcucacccaacagauccggguuc........................................    1
Gi3_27559772_x5...........................................................cagauccgggGucgauuccc.................................    1
Gi5_8566427_x5................................................uucucGcccaacagauccg.............................................    1
Gi3_27551832_x5...................................................................gguucgauCcccggcgcc...........................    1
Gi3_27651732_x5....................................................................guucgauucccggAgccggag.......................    1
Gi3_27608742_x5...................................................................ggAucgauucccggcgcc...........................    1
Gi3_27585937_x5................................................uucucacccaacagauUcg.............................................    1
```

Gi3_27653367_x5...uucgaCucccggcgccggagc......................   1
Gi2_10616180_x5..guucgauucccgUcgccg..........................   1
Gi5_8590282_x5...gguCcgauucccggcgccgga........................   1
Gi3_27577512_x5..guucgauucccggcgUcg..........................   1
Gi1_4002880_x5..guucgauucccggcgUcggagc......................   1
Gi5_8596177_x5...ucucacccaacCgauccg...   1
Gi3_27633117_x5.................Cccggcguaguauaacggcuagaa...   1
Gi2_10630885_x5..ucgauucccggcgccggCgc......................   1
Gi3_27650387_x5...gguNcgauucccggcgccgga........................   1
Gi1_3995685_x5..Gcgauucccggcgccggagc......................   1
Gi1_4007795_x5...ugguucuUacccaacagauccgg..   1
Gi5_8592582_x5..gguucuAacccaacagauccg...   1
G1a_5760217_x5...ggCucgauucccggcgccggagc......................   1
Gi3_27606677_x5...ugguucucacccGacagauccg...   1
Gi5_8567237_x5.................uccggcguNguauaacggcuagaa...   1
Gi1_4005990_x5.................uccAgcguaguauaacggcuag...   1
Gi1_4005280_x5.................uccggcguaguauGacggcuag...   1
Gi5_8591027_x5..guucgauucccggcgccggU........................   1
Gi3_27561792_x5...uGucacccaacagauccg...   1
Gi1_4001215_x5...gguucgauucccggAgccgg.........................   1
Gi1_3997850_x5...guucucacccaaUagauccg...   1
Gi2_10620790_x5.................uccggcguaguauaacggcCag...   1
G1a_5752867_x5...gguucgauucccggcgccggGgc......................   1
Gi3_27591482_x5.................uccggUguaguauaacggcuagaau..   1
Gi1_4014775_x5.................uccggcguaguauaacggcuagCau..   1
Gi3_27612727_x5...gguucgauucGcggcgccggagc......................   1
Gi5_8568887_x5.................uccNgcguaguauaacggcuag...   1
Gi2_10638655_x5..guucgauucccggcgcUggag.......................   1
Gi5_8592012_x5..Cuucgauucccggcgccggagc......................   1
Gi5_8586612_x5...gguucgauucccggUgccgg.........................   1
Gi2_10617660_x5.................uccggcgCaguauaacgg...   1
Gi5_8591902_x5.................uccggcAuaguauaacggcuagaa...   1
Gi5_8579307_x5...guucucacccaaUagauccg...   1
Gi2_10602770_x5...gguucgauucccgUcgccggagc......................   1
Gi2_10605510_x5.................uccNgcguaguauaacggcuaga..   1
Gi5_8580837_x5.................uccggcguaguauaCcggcuagaau..   1
Gi3_27611272_x5..guucgUuucccggcgccgga........................   1
Gi5_8598052_x5...uCguucucacccaacagauccg...   1
Gi2_10606495_x5..gguucucacccaCcagauccg...   1
Gi1_4015505_x5..guucgauucccggGgccgg.........................   1
Gi1_3994160_x5...Cgguucucacccaacagauccg...   1
Gi1_4005095_x5...Cuucucacccaacagauccg...   1
Gi3_27613417_x5...uGacccaacagauccggguuc..   1
Gi3_27572302_x5...gguucgauucccUgcgcc...........................   1
Gi3_27596067_x5..uucucaccUaacagauccgg..   1
Gi5_8570782_x5...guucuUacccaacagauccg...   1
Gi5_8598692_x5..uucucacccGacagauccg...   1

Gi1_4010130_x5..uucucacccaacagauAcg.. 1
Gi1_3997125_x5...guucAcacccaacagauccg.. 1
Gi5_8588572_x5..acagauccggguucgauucccggcgccggagcC.................... 1
Gi5_8581037_x5...gguucgauCcccggcgcc.......................... 1
Gi1_4017445_x5....................ggcguaguauaacggcuagaau... 0
Gi2_10634505_x5..guucgauucccggcgAcgga....................... 1
Gi5_8589032_x5...gguucgauucccggcgccggagcU.................... 1
Gi1_4005840_x5..................cGggcguaguauaacggcua... 1
Gi3_27600652_x5..ucgauuccNggcgccggag...................... 1
Gi1_4014320_x5..uucucacccaacCgauccg.. 1
Gi3_27610652_x5..ucgauucUcggcgccggagc..................... 1
Gi5_8576632_x5..cucacccaGcagauccgg... 1
Gi5_8577237_x5..cGcaacagauccggguuc....................................... 1
Gi2_10591075_x5.................uccggGguaguauaacggcuaga... 1
Gi1_4010650_x5..guucgauucccggcgccggagcgC................... 1
Gi3_27585387_x5.................uccggcguaguauaacggcuaAaa.. 1
Gi5_8600352_x5..guucgauCcccggcgccggagc..................... 1
Gi3_27655387_x5..Gaacagauccggguucgauuccc................................ 1
Gi2_10642000_x5...ggNucgauucccggcgccggagc..................... 1
Gi1_4000445_x5...gguucgauucccggcgccgA........................ 1
Gi1_4015315_x5..uucucacccaacagaucAg.. 1
Gi5_8575312_x5..guucgauuccUggcgccggagc..................... 1
Gi3_27630187_x5..uucucacccGacagauccgg... 1
Gi1_4010015_x5...guGcucacccaacagauccgg... 1
Gi1_4002305_x5.................uccgUcguaguauaacggcuaga... 1
Gi2_10604880_x5...gguucgauuccAggcgccgga....................... 1
Gi1_4009640_x5..ucgauuccNggcgccggagc..................... 1
Gi3_27588207_x5..agUuccggguucgauuccc................................ 1
Gi1_4003010_x5..uucucacccaacagauccgA... 1
Gi1_3995895_x5...ugguAcucacccaacagauccgg... 1
Gi1_4008185_x5.................uccAgcguaguauaacggcua... 1
Gi5_8601592_x5..guucgauucccggcgccC......................... 1
Gi3_27654967_x5...gguucgauucccggcgccCga....................... 1
Gi5_8574987_x5.................uccggcguaguauaacggUuagaau... 1
Gi3_27567322_x5..gauucccggcgccggCgc..................... 1
Gi5_8588937_x5.................uccAgcguaguauaacggcua... 1
Gi5_8570127_x5..uucucacccaCcagauccgg... 1
Gi3_27562322_x5.................uccggcguaguauaacggAuagaau... 1
Gi5_8596607_x5...ggAucgauucccggcgcc.......................... 1
Gi2_10622310_x5...gCuucgauucccggcgccg......................... 1
Gi5_8571647_x5....................gUcguaguauaacggcuagaau... 1
Gi1_4000140_x5...uggAucucacccaacagauccgg... 1
Gi1_4010410_x5..Auucgauucccggcgccg......................... 1
Gi2_10613545_x5..ucgauucccggcgccggUgc..................... 1
Gi5_8593232_x5.................uccggcguaAuauaacggcuagaau... 1
Gi5_8587617_x5.................uccggcguaguauaacggcuagaC.. 1
Gi1_3997310_x5.................uccgUcguaguauaacggc... 1

Gi3_27656182_x5..gguucucacccaacaAauccg... 1
Gi2_10630205_x5...guucucacccaacCgauccg... 1
Gi1_3993955_x5.................uccggcguaguauaCcggcuag... 1
Gi5_8566167_x5..gUguucgauucccggcgccggagc...................... 1
Gi3_27584367_x5...gguucgauucccgCcgccggagc...................... 1
Gi5_8598067_x5.................uccggAguaguauaacggcuagaau.. 1
Gi5_8582857_x5...gguucgauuAccggcgccggagc...................... 1
Gi1_4015470_x5..guucgauucGcggcgccgg......................... 1
Gi2_10605985_x5.................uccggcguaguauaacggcuUga.. 1
Gi5_8598202_x5...Cuucucacccaacagauccgg.. 1
G1a_5746312_x5.................Gccggcguaguauaacggcua.. 1
Gi1_3996275_x5.................uccggcguaguauaaUggcuagaau.. 1
Gi2_10602395_x5.................uccggcguaguauaacgAcuagaau.. 1
Gi1_4006845_x5...uggGucucacccaacagauccgg.. 1
Gi1_4012760_x5..uucucacccaacagauccA... 1
Gi5_8567422_x5.................uccggcguaguauaacggcuUga.. 1
Gi3_27589007_x5..guucgauucccgUcgccggag....................... 1
Gi1_4011940_x5.................uccggcguUguauaacgg... 1
Gi1_4016660_x5...guucucacccaacagaucAg... 1
Gi2_10644650_x5..gguucucacccaacagaucAg... 1
Gi2_10622855_x5...gguucgauucccggcgccgCag....................... 1
Gi2_10646650_x5...cUgguucgauucccggcgccgga........................ 1
G1a_5760192_x5.................uccggcgCaguauaacggcuaga.. 1
Gi5_8600132_x5.................uccggcgCaguauaacgg... 1
Gi2_10632270_x5.................uccggUguaguauaacggcuag... 1
Gi5_8602037_x5...ucacccaacGgauccggguu... 1
Gi5_8584982_x5...gguucgauucccggUgccg.......................... 1
Gi2_10596120_x5..guucgCuucccggcgccggagc...................... 1
Gi3_27619017_x5...gguucgauucccggcgcccCg........................ 1
Gi1_3995460_x5..uuAucacccaacagauccg... 1
G1a_5739327_x5.................ucUggcguaguauaacggcuag... 1
Gi2_10610005_x5...Uuucucacccaacagauccgggu.. 1
Gi3_27604532_x5...guCcucacccaacagauccgg.. 1
Gi1_4003445_x5...gguucgauNcccggcgccggag....................... 1
Gi4_535038_x5 ....Gugguucucacccaacaga.. 1
Gi3_27626252_x5.................uccggcguaguauaaGgg... 1
Gi2_10628725_x5.................uccggcguaguCuaacggcua.. 1
Gi5_8572457_x5...ugguucucacccaGcagauccg... 1
Gi3_27567232_x5..uucucacccaacagauccC... 1
Gi2_10648950_x5.................uccggcguaguauCacggcua.. 1
Gi2_10646330_x5.................uccggcguaguCuaacggc.. 1
G1a_5735567_x5..guCcgauucccggcgccgg......................... 1
Gi5_8594457_x5..guucgauuccUggcgccgga........................ 1
Gi3_27591427_x5..ucgauuccNggcgccgga........................ 1
Gi3_27647232_x5.................uccgAcguaguauaacggcuagaau.. 1
Gi5_8598442_x5..guucgaGucccggcgccggagc...................... 1
Gi1_4003600_x5.................uGcggcguaguauaacggcuagaau.. 1

Gi3_27623577_x5...gguucgauucccggcUccgga......................... 1
Gi3_27561947_x5.................uccggcgAaguauaacggcuagaau... 1
Gi1_4006125_x5..guucgauucccggcgccgAa......................... 1
Gi5_8575227_x5..guucgaAucccggcgccgg.......................... 1
Gi5_8573097_x5.................uccggcguCguauaacggc... 1
Gi2_10630340_x5...gguuGgauucccggcgccggag........................ 1
Gi3_27595807_x5..ucgauucccggUgccggagc....................... 1
Gi3_27618362_x5...Gcacccaacagauccggguuc... 1
Gi5_8581882_x5...Agguucucacccaacagauccg.. 1
Gi2_10601940_x5.................ucUggcguaguauaacggcuagaa.. 1
Gi1_4011745_x5..uucuGacccaacagauccg.. 1
Gi5_8568272_x5.................uccggcguaguauaacggU... 1
Gi1_3999215_x5...uucgauucNcggcgccgg.......................... 1
Gi3_27607117_x5..ucgauucccggcgccgAagc....................... 1
Gi2_10603755_x5...guucucacccaCcagauccg.. 1
Gi3_27635117_x5...gguucAauucccggcgcc............................ 1
Gi2_10607020_x5...guucucacccaacagaCccg.. 1
Gi2_10614235_x5..guucgauucccggUgccg........................... 1
Gi5_8573662_x5...ugguCcucacccaacagauccg.. 1
Gi2_10622525_x5.................uccggcguGguauaacggcuagaa.. 1
Gi1_3995375_x5...gguucgauucccggcgccAga......................... 1
Gi1_3996170_x5...gguucgauucccggcgccgC.......................... 1
Gi5_8571332_x5.................uccggcguUguauaacggcuag.. 1
G1a_5740252_x5.................uccggcguNguauaacggcuaga... 1
Gi5_8565527_x5..cccaacagNuccggguuc... 1
Gi1_3996520_x5..guucUauucccggcgccgga......................... 1
Gi1_4013950_x5...ugguucucacccaGcagauccgg... 1
Gi5_8575022_x5...gguucgaCucccggcgccggag........................ 1
Gi5_8569292_x5...gguucgaCucccggcgccgga......................... 1
Gi5_8598422_x5.................uccggcguaguauGacggcua... 1
Gi5_8577467_x5..guAcgauucccggcgccggagc....................... 1
G1a_5747067_x5.................uccggcguaguauaacggcuagC... 1
Gi3_27574737_x5..guucgauucccggcgccgCa......................... 1
Gi5_8590952_x5..gguucucacccaaAagauccg.. 1
Gi1_4015240_x5..uucucacAcaacagauccg.. 1
Gi5_8576682_x5..uGgguucucacccaacagauccg.. 1
Gi1_4005155_x5...gguucgauNcccggcgccgga......................... 1
Gi5_8578112_x5..gguucucaccAaacagauccg.. 1
Gi5_8601712_x5...ugguucucacccaacagauAcg.. 1
Gi2_10627645_x5..guucgauAcccggcgccgg.......................... 1
Gi2_10630375_x5...gguucgauCcccggcgccgg.......................... 1
Gi1_4003015_x5..guucgauucccggcgcUg........................... 1
Gi3_27598827_x5....................gUcguaguauaacggcuag.. 1
Gi2_10592435_x5..gguCcucacccaacagauccg.. 1
Gi3_27605632_x5.................uccggcguaguauaacggcuCg.. 1
Gi3_27574097_x5.................uccggcguaguaCaacggcuag.. 1
Gi5_8582642_x5.................uccggcgCaguauaacggcua... 1

```
Gi1_4009905_x5................................................uucucacccGacagauccg.............................................      1
Gi3_27550742_x5....................................................................guucgaAucccggcgccggagc......................      1
G1a_5763772_x5....................................................................gGucgauucccggcgccg..........................      1
Gi5_8637731_x4.................uccggcguCguauaacggcua..........................................................................      1
Gi3_27753009_x4........................................................cGacagauccggguucgauuccc.................................      1
Gi5_8642743_x4.................uccggcguaAuauaacgg.............................................................................      1
Gi3_27772193_x4.................................................Ccucacccaacagauccg.............................................      1
Gi1_4029016_x4.................................................ucucacccNacagauccg.............................................      1
Gi1_4026056_x4..............................................ggGucucacccaacagauccg.............................................      1
Gi1_4043464_x4...................................................................ggCucgauucccggcgccg..........................      1
Gi5_8636555_x4.................uccggcguagAauaaacggcuag........................................................................      1
Gi3_27686413_x4.............................................ugguucucacccaacagaCccg.............................................      1
Gi2_10666019_x4....................................................................guucgauucccggcgcAgga........................      1
Gi2_10714383_x4................................................uucCcacccaacagauccg.............................................      1
G1a_5806874_x4.................uccggcguaguauaacAgc............................................................................      1
Gi1_4043600_x4.................uccggcguUguauaacggcuaga........................................................................      1
Gi3_27737637_x4...................................................................gguucgauucccggcgccggaC.......................      1
Gi5_8642943_x4.................Accggcguaguauaacggcua..........................................................................      1
Gi1_4045912_x4.................ucGggcguaguauaacggcuag.........................................................................      1
Gi5_8609195_x4...............................................guucucacccaacagauAcgg............................................      1
Gi2_10690695_x4...................................................................gguucgGuucccggcgccgg.........................      1
Gi1_4037508_x4.................uccggcguaguauaacggAua..........................................................................      1
Gi2_10660663_x4.....................................................................uucgauucNcggcgccgg.........................      1
Gi1_4021276_x4.................uccggcguaguauaCcggc............................................................................      1
Gi1_4040124_x4....................................................................guCcgauucccggcgccgga........................      1
Gi2_10723707_x4......................................................................ucgauuccUggcgccggagc......................      1
Gi5_8611723_x4.............................................................gauccggguucgauuccc.................................      0
Gi3_27733425_x4.................uccgCcguaguauaacggcua..........................................................................      1
Gi3_27777017_x4...............................................guucucacccaacagCuccg.............................................      1
Gi3_27723821_x4..................ccggcguaguauaacggcuagG........................................................................      1
G1a_5790914_x4...................................................................gguucgGuucccggcgccggagc......................      1
Gi2_10671651_x4.................uccggcguaguauaacggAuaga........................................................................      1
Gi1_4025668_x4....................................................................guucgauucccggcgUcgga........................      1
Gi3_27735613_x4....................................................................guucgauucccggcgcUggag.......................      1
Gi5_8640951_x4..................................................cucacccaacGgauccgg............................................      1
Gi5_8624019_x4....................................................................guucgauucUcggcgccggagc......................      1
Gi2_10711235_x4................................................uucucacccaacaAauccgg............................................      1
Gi3_27680801_x4...................................................................gguucgauucccggcCccggag.......................      1
Gi2_10679567_x4.................uccggcguaguauaacggcCagaa.......................................................................      1
Gi2_10681647_x4...................................................................gguucCauucccggcgccggagc......................      1
Gi2_10658783_x4.................uccggcguaguauaaUggcuagaau......................................................................      1
Gi1_4029512_x4.............................................ugguucucacccaacagauccUg............................................      1
Gi5_8645679_x4.................uccggcguaguaGaacggcua..........................................................................      1
Gi5_8636847_x4................................................uucucacccaacagauccgAguuc........................................      1
Gi2_10724311_x4...................................................................gguucgauucccAgcgccgg.........................      1
Gi3_27696025_x4...............................................guucucacccaacagauccggguucga......................................      0
Gi5_8638887_x4....................................................................guucgauuUccggcgccgga........................      1
```

```
Gi2_10704539_x4..................................................................Agguucgauucccggcgccggagc......................      1
G1a_5776354_x4...................................................................gguucgauucccggcgccggagcCu....................      1
Gi5_8627359_x4...................................................................gguucgauucccggAgcc...........................      1
Gi2_10684211_x4...................................................................gguucgauuccUggcgccgg.........................      1
Gi1_4040728_x4...............................................guucucCcccaacagauccgg............................................      1
Gi3_27770765_x4..................cUggcguaguauaacggc............................................................................      1
Gi3_27687713_x4.................uccggcguaguauaacggcuagaauU.....................................................................      1
Gi3_27680249_x4...............Gguccggcguaguauaacggcuagaaugg....................................................................      1
Gi2_10709991_x4.................uccAgcguaguauaacgg.............................................................................      1
Gi2_10665471_x4.................uccggcguaguauaCcggcua..........................................................................      1
Gi3_27719873_x4.................uccggcguaguauUacggcu...........................................................................      1
Gi3_27730345_x4...................................................................gguucgauuAccggcgccgg.........................      1
Gi5_8618779_x4.................uccggcguGguauaacggcuagaau......................................................................      1
Gi3_27793401_x4.................uccggcguaguauaacggcuCgaa.......................................................................      1
Gi3_27790573_x4....................................................................guucgaGucccggcgccggag.......................      1
Gi3_27757405_x4.................uccggcguaguauaCcggcuag.........................................................................      1
Gi1_4028852_x4....................................................................guucgGuucccggcgccgga........................      1
Gi5_8612143_x4................................................uucucacccaacagaucAg.............................................      1
Gi1_4019692_x4.................................................uGucacccaacagauccg.............................................      1
Gi3_27739557_x4...................................................................gguucgauucccggcgcccC.........................      1
Gi5_8636211_x4.................ucNggcguaguauaacggcuaga........................................................................      1
Gi5_8635967_x4................................................uucucaUccaacagauccg.............................................      1
Gi3_27667689_x4........................................................caacagauNcggguucga......................................      1
Gi5_8649707_x4................................................uucucaccUaacagauccg.............................................      1
Gi2_10666835_x4......................................................................Acgauucccggcgccggagc......................      1
G1a_5793414_x4.................uccggcguaguauaacggcuGga........................................................................      1
Gi5_8623915_x4...................................................................gguucgauucccggAgccggag.......................      1
Gi2_10693463_x4...................................................................gguucgauuccAggcgccg..........................      1
Gi2_10672991_x4....................................................................guAcgauucccggcgccgga........................      1
Gi5_8607155_x4......................................................Gccaacagauccggguuc........................................      1
Gi1_4021920_x4.................uccggcguaguauaaUggc............................................................................      1
Gi1_4041980_x4.................uccggcguaguCuaacggcua..........................................................................      1
Gi3_27734861_x4.................uccggcguaguCuaacggcuagaau......................................................................      1
Gi3_27717393_x4.................uccggcguaguauaacggcGa..........................................................................      1
Gi5_8647855_x4.................uccggcguUguauaacggcua..........................................................................      1
Gi5_8608251_x4.................ucGggcguaguauaacggcuaga........................................................................      1
Gi5_8631527_x4.....................................................................uGcgauucccggcgccgg.........................      1
Gi3_27754773_x4......................................................................uUgauucccggcgccgga........................      1
Gi2_10678799_x4.....................................................................uuUgauucccggcgccgg.........................      1
G1a_5767494_x4...................................................................gguucgauNcccggcgccgg.........................      1
Gi3_27663033_x4..............................................gguucucaccAaacagauccg.............................................      1
Gi2_10663483_x4...............................................guucucacUcaacagauccg.............................................      1
Gi3_27778965_x4..................................................cuUacccaacagauccgg............................................      1
Gi2_10655315_x4.................uccggcguagCauaacggcuag.........................................................................      1
Gi2_10657263_x4....................................................................guucgauucccgAcgccg..........................      1
Gi1_4031480_x4.................uccggcguaguCuaacggcuag.........................................................................      1
Gi1_4043404_x4....................................................................guucgauucccgCcgccgg.........................      1
```

```
Gi1_4037432_x4.................uccggcguaguauaacAgcuagaa.......................................................................      1
Gi5_8638903_x4.................................................ucAcacccaacagauccg.............................................      1
Gi3_27791545_x4....................................................................guucgauuccGggcgccgga........................      1
Gi3_27711361_x4.................uccggcguaguauaUcggcuagaa.......................................................................      1
Gi3_27738193_x4.................uccggcguCguauaacggcuagaau......................................................................      1
Gi5_8602619_x4....................................................................guucgGuucccggcgccgga........................      1
Gi1_4018196_x4.....................................................................uucgauucccggcgcUgg.........................      1
Gi2_10690995_x4.................uccggcguaguauaacggcuagaUu......................................................................      1
Gi3_27781409_x4......................................................................ucgauucccggcgccAga........................      1
Gi2_10675863_x4.................uccggcguaguauaacgAcuag.........................................................................      1
Gi2_10701467_x4.................uccggcguaguGuaacggcuag.........................................................................      1
Gi3_27664693_x4...........................................................cGgauccggguucgauucccggcgccgg.........................      1
Gi2_10669531_x4.............................................ugguucucGcccaacagauccg.............................................      1
Gi2_10714971_x4.................uccggcguaguauaacggUuag.........................................................................      1
Gi2_10712155_x4......................................................................ucgaGucccggcgccggagc......................      1
Gi1_4031972_x4.................uUcggcguaguauaacggcuaga........................................................................      1
Gi5_8629787_x4...................................................................gguucgauucccUgcgccgga........................      1
Gi1_4034080_x4....................................................................guucgauuNccggcgccg..........................      1
Gi5_8619207_x4..................Gcggcguaguauaacggcua..........................................................................      1
Gi3_27681081_x4......................................................................ucgauuccUggcgccggagc......................      1
Gi3_27740305_x4.............................................ugguucucacccaacagaCc...............................................      1
Gi5_8650211_x4...................................................................gguucgauuccAggcgccg..........................      1
Gi1_4036624_x4.................uccggAguaguauaacggcuag.........................................................................      1
Gi2_10655231_x4...................................................................gguucgauucccggcUccggagc......................      1
Gi1_4031772_x4....................................................................guucgauucccggcgccgCagc......................      1
Gi2_10723675_x4.............................................uAguucucacccaacagauccg.............................................      1
Gi2_10703143_x4...................................................................gguucgauucccggcCccggagc......................      1
Gi2_10652575_x4.................Gccggcguaguauaacggcu...........................................................................      1
Gi2_10695131_x4...................................................................gguucgauucccgAcgccgga........................      1
Gi2_10718439_x4.............................................ugguucucacccaGcagauccg.............................................      1
Gi2_10670903_x4.................uccggAguaguauaacgg.............................................................................      1
Gi3_27744169_x4.............................................ugguucucacccaacCgauccg.............................................      1
Gi3_27685973_x4............................................................agauccggNuucgauuccc.................................      1
Gi5_8621407_x4...............................................guucucacccaacagauUcgg............................................      1
Gi1_4039380_x4....................................................................guucgauucccggcgccggagU......................      1
Gi5_8602203_x4.................uccggcCuaguauaacgg.............................................................................      1
Gi1_4037176_x4...................................................................gguCcgauucccggcgccggagc......................      1
Gi3_27772357_x4....................................................................guucgCuucccggcgccggag.......................      1
Gi1_4033128_x4.............................................ugguuUucacccaacagauccgg............................................      1
Gi5_8615995_x4...................................................................gAuucgauucccggcgccggag.......................      1
Gi3_27731089_x4......................................................................Ccgauucccggcgccggagc......................      1
Gi1_4023416_x4..................................................cCcacccaacagauccgg............................................      1
Gi2_10653131_x4....................................................................guGcgauucccggcgccgga........................      1
Gi3_27764721_x4........................................................................gauucccggcgcUggagc......................      1
Gi2_10650611_x4.................uccggcgAaguauaacggcuaga........................................................................      1
Gi1_4025384_x4.................uccggcguNguauaacggcu...........................................................................      1
Gi1_4039176_x4.............................................ugguucucaccUaacagauccgg............................................      1
```

```
Gi5_8636191_x4................................................uucucacccCacagauccg.............................................     1
Gi2_10681731_x4....................................................................guucgauucccUgcgccgg.........................     1
Gi3_27731845_x4.................uccggcguaguaAaacgg.............................................................................     1
Gi5_8618571_x4................................................uucucacccaacagaucUg.............................................     1
Gi5_8620595_x4...................................................................gguAcgauucccggcgccg..........................     1
Gi2_10661571_x4.................uccggcguaguauaacggA............................................................................     1
Gi2_10660771_x4....................................................................guucgauucccggAgccgg.........................     1
Gi2_10700483_x4...................................................................gguucgauucUcggcgccg..........................     1
Gi1_4045972_x4.................uccAgcguaguauaacggcuaga........................................................................     1
Gi1_4046608_x4...................................................................gguucgauAcccggcgccgg.........................     1
Gi2_10685383_x4...................................................................gguucgauucccgAcgccggag.......................     1
Gi3_27677373_x4..................cGggcguaguauaacggcuaga........................................................................     1
Gi1_4025080_x4.............................................ugguucucacccaGcagauccg.............................................     1
Gi1_4026508_x4...............................................guucucaccUaacagauccgg............................................     1
Gi5_8642555_x4.................uGcggcguaguauaacggcu...........................................................................     1
Gi3_27746597_x4.................uccggcguNguauaacggcu...........................................................................     1
Gi3_27779217_x4....................................................................guucgauAcccggcgccggag.......................     1
Gi3_27672645_x4........................................................................gauuUccggcgccggagc......................     1
Gi3_27722585_x4.................uccggcgAaguauaacgg.............................................................................     1
Gi2_10711727_x4....................................................................guucAauucccggcgccggag.......................     1
Gi2_10670995_x4....................................................................guucgGuucccggcgccggag.......................     1
Gi3_27718133_x4...................................................................gguucgauAcccggcgcc...........................     1
G1a_5784282_x4...................................................................gguucgauNcccggcgccggag.......................     1
Gi5_8644247_x4...................................................................gguuGgauucccggcgccg..........................     1
Gi3_27665241_x4................................................uucucacccaacagauccA.............................................     1
Gi5_8625943_x4..............................................gguNcucacccaacagauccg.............................................     1
Gi5_8604751_x4...................................................................gguucgauucccggcUccg..........................     1
Gi3_27762773_x4................................................uucucacccaacUgauccgg............................................     1
Gi1_4030560_x4...................................................................Cguucgauucccggcgccg..........................     1
Gi3_27734441_x4.......................................................................cgauucccggcgccggagcA.....................     1
Gi1_4039424_x4................................................uucucaccAaacagauccg.............................................     1
Gi2_10695635_x4......................................................................ucgauucccggUgccggagc......................     1
G1a_5801486_x4...........................................................cagauccggguucgauucccggcgccggag.......................     0
Gi1_4026748_x4.................uccggUguaguauaacggcuaga........................................................................     1
Gi1_4033416_x4..................................................................ggguucgauucccggcgccgg.........................     0
Gi1_4022416_x4...................................................................gguucgauucccAgcgccggagc......................     1
Gi2_10659599_x4....................................................................guucgauuAccggcgccgg.........................     1
Gi1_4028676_x4...................................................................gguucgauuccAggcgccgg.........................     1
Gi5_8608015_x4.................uccggAguaguauaacggcuag.........................................................................     1
Gi3_27700177_x4....................Ugcguaguauaacggcuagaau......................................................................     1
Gi1_4039752_x4..................................................cucacccaNcagauccgg............................................     1
Gi5_8616915_x4........................................................................gauucccgNcgccggagc......................     1
Gi2_10690783_x4.................uccggcguaguauCacggcuagaa.......................................................................     1
Gi1_4024132_x4.................uccggcguaguauaacAgcu...........................................................................     1
Gi5_8618407_x4...............................................guucucacccaacaAauccg.............................................     1
G1a_5770894_x4.................uccgUcguaguauaacggcuaga........................................................................     1
Gi3_27678893_x4....................................................................guucgauucccggcgccCgag.......................     1
```

Gi1_4029248_x4...ugguucucacccaacagauccgC.. 1
Gi2_10683207_x4..cGcacccaacagauccgg.. 1
Gi3_27789605_x4.................uccUgcguaguauaacggcua.. 1
G1a_5768298_x4.................uUcggcguaguauaacggcuag... 1
Gi2_10693895_x4..guucgauucccggcgUcggag....................... 1
Gi3_27713285_x4...Gggguucgauucccggcgccgga........................ 1
Gi3_27787413_x4..ucgauucccgAcgccggagc...................... 1
Gi3_27761817_x4..guucgauucccggGccgga......................... 1
Gi1_4046468_x4..uucucacccaaUagauccg... 1
Gi2_10684895_x4.................uccggcguaguauaacggcuU.. 1
Gi1_4032704_x4...gguucgauucccggAgccggagc...................... 1
Gi2_10687567_x4..guucCauucccggcgccgg......................... 1
Gi5_8607547_x4.................uccggcguaguUuaacggcuaga.. 1
Gi1_4027888_x4.................ucUggcguaguauaacggcuagaau.. 1
Gi3_27734069_x4.................uccggcCuaguauaacggcua.. 1
Gi3_27687081_x4..uucucacUcaacagauccgg.. 1
G1a_5786382_x4..guucgaCucccggcgccgga........................ 1
Gi1_4021644_x4...ugguucucacccaUcagauccgg.. 1
Gi1_4026196_x4...guucucacccaacagauccgU.. 1
Gi5_8632635_x4...gguucgauucccggcgccU.......................... 1
Gi3_27773113_x4..gUguucgauucccggcgccgga........................ 1
G1a_5789218_x4...uGucacccaacagauccg... 1
Gi5_8608147_x4..cucacccGacagauccgg.. 1
Gi5_8614263_x4...gguucgauucccggcgAcgg......................... 1
Gi2_10683051_x4.................uccggcguaguauaacCgcuaga.. 1
Gi1_4018652_x4.................uccggcgGaguauaacgg... 1
Gi3_27746029_x4...gguucgUuucccggcgccggag....................... 1
Gi5_8637387_x4...ucucaccUaacagauccg... 1
Gi1_4039608_x4..ucgauucccggcgccggagcCu.................... 1
Gi3_27795865_x4...gguucgauucccggcgccCgag....................... 1
Gi1_4024384_x4..gauucccggcgccggagcC..................... 1
G1a_5807306_x4.................uccggcguGguauaacggcuag... 1
Gi3_27690365_x4.................uccggcguaguauaacggcuaUaa... 1
G1a_5778542_x4..uucucaccNaacagauccg... 1
Gi1_4026548_x4..guGcgauucccggcgccggagc...................... 1
Gi1_4017968_x4...gguucgauuAccggcgccgg......................... 1
Gi2_10670063_x4...............aguccggcNuaguauaacggcuaga.. 1
Gi2_10674299_x4.................uccggcguaguauaacgC... 1
G1a_5797642_x4...uUguucucacccaacagauccg... 1
Gi3_27715681_x4...gguucgauucccgAcgcc........................... 1
Gi4_544242_x4 ....uGgguucucacccaacaga... 1
Gi5_8646987_x4..gguucucacccaacaAauccg... 1
Gi1_4033572_x4...ugguucucaccUaacagauccg... 1
Gi1_4044292_x4..uucAcacccaacagauccgg.. 1
Gi1_4025512_x4..guucgauuccUggcgccggagc...................... 1
Gi1_4029828_x4...ugguucucacccaacagaucUg... 1
Gi5_8622307_x4.................ucAggcguaguauaacgg... 1

Gi2_10662051_x4.................ucAggcguaguauaacggcuaga.. 1
Gi1_4047284_x4..guucgGuucccggcgccggagc...................... 1
Gi3_27780217_x4..Ccgauucccggcgccgga........................ 1
Gi5_8622683_x4..guCcgauucccggcgccggagc...................... 1
Gi1_4047544_x4..gguuGucacccaacagauccg... 1
Gi3_27685289_x4...uCguucucacccaacagauccg... 1
Gi5_8631027_x4.................ucAggcguaguauaacggcuaga.. 1
Gi2_10662863_x4..Cguucucacccaacagauccg... 1
Gi1_4037048_x4..guucgauCcccggcgccggagc...................... 1
Gi2_10721295_x4...cagauccggguucgauucccggcgccgga........................ 0
Gi3_27695869_x4...gguucCauucccggcgccggagc...................... 1
Gi5_8633619_x4..guucgauucccggcgccggagcU..................... 1
G1a_5805290_x4..uGcucacccaacagauccg... 1
Gi1_4031176_x4..guucgauucccAgcgccggagc...................... 1
Gi2_10667263_x4...ugUuucucacccaacagauccg... 1
Gi1_4045956_x4...guucucacccaacagauUcg... 1
Gi1_4021344_x4.................uccggcguaguauCacggcuagaau.. 1
Gi2_10677239_x4..uucucaccUaacagauccg... 1
Gi5_8631047_x4...guucuAacccaacagauccgg.. 1
Gi5_8624183_x4...guucucacAcaacagauccg... 1
Gi5_8633971_x4...guucuAacccaacagauccg... 1
Gi3_27679433_x4.................uccggcguaguauaacggcGag... 1
Gi2_10650651_x4................aUuccggcguaguauaacgg.. 1
Gi5_8638095_x4.................uccgCcguaguauaacggcuaga.. 1
Gi2_10722963_x4..gguucucacUcaacagauccg... 1
Gi3_27786237_x4....................ggcguGguauaacggcua.. 1
Gi5_8649635_x4...guucucacccGacagauccgg.. 1
Gi5_8611627_x4..guucgauucccggcgccgAa........................ 1
G1a_5768570_x4...Aguucgauucccggcgccggag....................... 1
Gi1_4027556_x4...uUucacccaacagauccg... 1
Gi1_4044148_x4...ucucacccaacagCuccg... 1
Gi5_8625755_x4...guucucacccaacGgauccgg.. 1
Gi2_10669571_x4..ggguucgauucccggcgccggagc...................... 0
Gi2_10717675_x4..gguucuUacccaacagauccg... 1
Gi2_10692995_x4..gguucucacccaacagauc... 0
Gi3_27786177_x4.................uccggcguaguauaacggcuUgaa... 1
Gi2_10659299_x4..gguucucacccaGcagauccg... 1
Gi1_4026924_x4...guucucaccAaacagauccg... 1
Gi3_27733409_x4.................Accggcguaguauaacggcuagaau.. 1
G1a_5778154_x4.................uccggcguaguaCaacggcuaga.. 1
Gi3_27763597_x4...cagauccggguucgGuuccc................................. 1
Gi5_8638219_x4..guucgaAucccggcgccg.......................... 1
Gi2_10718583_x4...ggAucgauucccggcgccgga........................ 1
Gi3_27683689_x4..ucgGuucccggcgccgga........................ 1
G1a_5812030_x4..guucgaCucccggcgccg.......................... 1
Gi1_4020584_x4...ugguucCcacccaacagauccg... 1
Gi1_4046500_x4..cucacccaacGgauccgg.. 1

Gi1_4046344_x4..uucucacccaacagauUcgg.. 1
Gi1_4043420_x4...gguucgCuucccggcgccggagc...................... 1
G1a_5791562_x4.................uccggcguaguauaGcggcuaga.. 1
Gi2_10683295_x4.....................gcguaguauaacggcuagaau.. 0
Gi1_4021004_x4.................Cccggcguaguauaacggcuagaau.. 1
Gi5_8636739_x4.................uccggcAuaguauaacggc.. 1
Gi3_27734973_x4..gguucucacAcaacagauccg... 1
Gi3_27768121_x4...guucAcacccaacagauccg... 1
Gi2_10712851_x4..gguucucacccaacagaCccg... 1
Gi1_4042952_x4...uAguucucacccaacagauccg... 1
Gi5_8633523_x4..guucAauucccggcgccgga........................ 1
Gi5_8648907_x4..guucgauucccggcgcGg.......................... 1
Gi3_27720869_x4..uucucacccaacagaCccgg.. 1
Gi5_8623135_x4..uucucacccaacagCuccg... 1
Gi1_4033656_x4..agauccggNuucgauuccc................................. 1
Gi3_27793233_x4...Cgguucucacccaacagauccgg.. 1
Gi5_8647743_x4...ucacccaacagauccgggCu... 1
Gi3_27685181_x4..................ccggcguaguauaGcggcuaga.. 1
Gi2_10668255_x4.................uccggcguaguauaCcgg... 1
Gi5_8609459_x4...gguucgCuucccggcgccgg......................... 1
Gi1_4046576_x4...guuUucacccaacagauccg... 1
Gi5_8621483_x4.................uccggcguaguauaacggcuagCa... 1
Gi2_10697727_x4..guucgauucccCgcgccgg......................... 1
Gi5_8634519_x4..................ccggcguaguauaacggc.. 0
Gi2_10702083_x4...gguAcgauucccggcgccggag....................... 1
Gi3_27713213_x4...ugguucucacccaGcagauccg... 1
G1a_5812070_x4.................uccggcguGguauaacggcuaga.. 1
Gi3_27788509_x4...ugguucucacccaCcagauccg... 1
Gi1_4038864_x4...ugguucucacccaacagauccgU.. 1
Gi3_27771089_x4...gguucgauAcccggcgccg.......................... 1
Gi3_27750101_x4..uuUucacccaacagauccggguuc.. 1
Gi1_4021520_x4.................uccggcguaguauaacgUcuag... 1
G1a_5794926_x4.................uccggcguaguauaacggcCaga.. 1
Gi1_4025804_x4.................uccggcguaguauaacggcuaAaa... 1
Gi2_10700555_x4...gguucgauucccggcgccgAag....................... 1
Gi3_27672201_x4...uggGucucacccaacagauccg... 1
G1a_5773650_x4..Uuucgauucccggcgccgga........................ 1
Gi1_4029416_x4..uGgauucccggcgccggagc...................... 1
Gi3_27735701_x4..guucgauuccGggcgccgg......................... 1
Gi5_8635099_x4..cucacccaaUagauccgg.. 1
G1a_5784406_x4.................uccggcguaguauaacAgcuag... 1
Gi5_8620599_x4.................uccggcguagCauaacggcuagaa... 1
Gi3_27766449_x4.................uccggcguaguauGacggcuag... 1
Gi3_27755897_x4.................uccUgcguaguauaacgg... 1
Gi5_8606315_x4...ugguGcucacccaacagauccg... 1
Gi3_27706561_x4...guucucacccaGcagauccgg.. 1
Gi3_27761645_x4...uUucacccaacagauccg... 1

Gi1_4047016_x4...gguucgauucccggcgcUggagc......................   1
Gi3_27705597_x4...ugguucCcacccaacagauc...   1
Gi2_10689443_x4...gguucUauucccggcgccggagc......................   1
Gi1_4019520_x4...ugguucucCcccaacagauccgg..   1
Gi3_27740821_x4.................uccggcguaguaGaacgg...   1
Gi3_27693585_x4.................uccggcguUguauaacgg...   1
Gi3_27752297_x4..guucgauucccggcgcGg..........................   1
Gi3_27793009_x4..uucucacccaacaAauccg...   1
Gi3_27701357_x4..Ggauccggguucgauucc..................................   1
Gi5_8644263_x4.................uccggAguaguauaacggcua..   1
Gi1_4037732_x4...ugAuucucacccaacagauccgg..   1
Gi5_8605199_x4..cucacccaacCgauccgg..   1
Gi5_8639407_x4...ugguucucacccaCcagauccgg..   1
Gi3_27677229_x4.................uccggcguCguauaacggcuag...   1
Gi2_10654171_x4...Cuucucacccaacagauccgg..   1
G1a_5787674_x4...ggGucgauucccggcgccg..........................   1
Gi3_27766557_x4...cgUuucccggcgccggag.......................   1
Gi5_8627543_x4...ucGcacccaacagauccg...   1
Gi5_8611827_x4.................uccggcguaguauUacggcuaga..   1
Gi3_27779881_x4.................uccUgcguaguauaacggc..   1
Gi1_4033028_x4..uucucacUcaacagauccgg..   1
Gi1_4042808_x4...ugguucucacccaacagauccgA..   1
Gi3_27740369_x4..................ccggcguGguauaacggc..   1
Gi2_10677467_x4..gAucgauucccggcgccg..........................   1
Gi3_27749661_x4..uucuGacccaacagauccg...   1
Gi5_8605863_x4...guucucacAcaacagauccgg..   1
Gi3_27685073_x4...gguucgauuAccggcgccg..........................   1
Gi3_27778913_x4..guucgauucccggcgGcg..........................   1
Gi2_10682415_x4..guucgauuUccggcgccgga........................   1
Gi5_8623607_x4..guucgauucccggcgcUggagc......................   1
Gi1_4029940_x4.................uUcggcguaguauaacggcuagaau..   1
Gi1_4043972_x4...guucucacccaacagCuccgg..   1
Gi5_8625803_x4...gguucgCuucccggcgcc...........................   1
Gi5_8630763_x4.................uccggcguaguauaacggcuagaG...   1
Gi3_27730749_x4..Aauucccggcgccggagc......................   1
Gi3_27745305_x4...gguucgaGucccggcgccggga.......................   1
Gi3_27743185_x4...uucgauucccggcgccggagcCu....................   1
Gi2_10716583_x4...guucucacccaacagauccA...   1
G1a_5772702_x4..uucucGcccaacagauccg...   1
Gi2_10672067_x4.................uccggcguCguauaacggcua..   1
Gi1_4038728_x4.................uGcggcguaguauaacggcu...   1
Gi2_10685011_x4...gAuucgauucccggcgccg..........................   1
G1a_5768546_x4.................uccggcguaguauaacggcuGg...   1
Gi2_10661671_x4...ugguucucacccaacagGuccg...   1
Gi5_8623143_x4...gguCcgauucccggcgcc...........................   1
Gi3_27695253_x4.................uccggcguaguauGacggcuagaa...   1
G1a_5807290_x4..guucgauucccggcgccggCgc......................   1

```
Gi2_10670415_x4...............................................................Gccggguucgauucccggcgccgga........................   1
Gi5_8634911_x4..................cGggcguaguauaacggcuaga........................................................................   1
Gi3_27749101_x4.....................................................................uucgauCcccggcgccgga........................   1
Gi5_8636855_x4.................uccggcguaguaCaacggcuagaa.......................................................................   1
Gi2_10717975_x4.................uccggcguCguauaacggc............................................................................   1
Gi1_4029868_x4.................uccggcguaguauaacgAcuagaau......................................................................   1
Gi3_27769641_x4...................................................................gguucUauucccggcgccggag.......................   1
Gi5_8617559_x4.................uccgAcguaguauaacggc............................................................................   1
Gi3_27730525_x4......................cUuaguauaacggcuaga........................................................................   1
Gi2_10706091_x4...................................................................ggAucgauucccggcgccg..........................   1
Gi5_8621807_x4................................................uucucaccNaacagauccgg............................................   1
Gi5_8621503_x4.................uccUgcguaguauaacgg.............................................................................   1
Gi5_8632299_x4.................uccgAcguaguauaacggcu...........................................................................   1
Gi1_4036960_x4.................uccggcguaguauaacggcuagaCu......................................................................   1
Gi1_4025808_x4....................................................................guucgauucccAgcgccg..........................   1
Gi3_27696305_x4....................................................................guucgauucccggAgccg..........................   1
Gi5_8628479_x4...................cUgcguaguauaacggcuaga........................................................................   1
Gi3_27713505_x4.................uccggcguaguauaCcggcuagaau......................................................................   1
Gi1_4036324_x4...................................................................gCuucgauucccggcgccgga........................   1
Gi1_4028916_x4...................................................................gguucgauucccggcgccA..........................   1
Gi5_8619903_x4....................................................................guuGgauucccggcgccg..........................   1
Gi5_8617791_x4.................uccggAguaguauaacggcuaga........................................................................   1
Gi3_27785053_x4..............................................gguucucacGcaacagauccg.............................................   1
Gi2_10657371_x4....................................................................guucgauucccggcgccAgag.......................   1
Gi2_10661863_x4....................................................................guucgauuAccggcgccg..........................   1
Gi2_10706115_x4...................................................................gguucgauucccgAcgccg..........................   1
Gi2_10700299_x4.................Accggcguaguauaacggcuagaau......................................................................   1
Gi4_539122_x4 ....................................................................guucgauucccggcgccggagcC.....................   1
Gi1_4023456_x4.................uccggcguaguaGaacggcuag.........................................................................   1
Gi3_27725249_x4.............................................uUguucucacccaacagauccg.............................................   1
Gi2_10677995_x4...................................................................gguucgauucccgAcgccgg.........................   1
Gi1_4025360_x4.................uccggcguCguauaacggcua..........................................................................   1
Gi3_27749889_x4..............................................ggCucucacccaacagauccgg............................................   1
Gi5_8603079_x4...................................................................gguucgauucccCgcgccg..........................   1
Gi3_27668109_x4....................................................................guucgauucccggcgAcg..........................   1
Gi3_27784729_x4................................................uucucacccaUcagauccgg............................................   1
Gi1_4028920_x4.................................................ucAcacccaacagauccgg............................................   1
Gi5_8650035_x4.............................................ugguucucacccaacUgauccg.............................................   1
Gi2_10669251_x4................................................uucucacccaacagaucUg.............................................   1
Gi3_27778521_x4....................................................................guucgauucccggcgccUgagc......................   1
Gi3_27792825_x4....................................................................guucCauucccggcgccgg.........................   1
Gi5_8639583_x4...................................................................gguuUgauucccggcgccgg.........................   1
Gi5_8644719_x4.................uccggcguaguauaacggGuaga........................................................................   1
Gi3_27739361_x4...................................................................Uguucgauucccggcgccggagcg.....................   1
Gi5_8623479_x4...................................................................gguCcgauucccggcgccggag.......................   1
Gi5_8621983_x4..............................................Aguucucacccaacagauccgg............................................   1
Gi5_8642475_x4...................................................................gguucgauucccggcgccgA.........................   1
```

```
Gi1_4022432_x4...................................................................gguucgauucccggcgcGgg.........................      1
Gi1_4040120_x4..............................................gguucucaccUaacagauccg.............................................      1
Gi3_27739937_x4.................uccgAcguaguauaacggcuagaa.......................................................................      1
Gi3_27784853_x4...................................................................gguucgauuAccggcgcc...........................      1
Gi4_538906_x4 ....................................................................guucgauucccggcgccggagcCu....................      1
Gi5_8643103_x4....................ggUguaguauaacggcuag.........................................................................      1
Gi3_27713121_x4..................ccggcguaguauaacAgcuaga........................................................................      1
Gi2_10722047_x4...............aUuccggcguaguauaacggcuaga........................................................................      1
Gi3_27753053_x4........................................................caUcagauccggguucgauuccc.................................      1
Gi5_8609083_x4.................uccggGguaguauaacggcuag.........................................................................      1
Gi1_4026372_x4...................................................ucacccaacagauccgggC..........................................      1
Gi5_8647391_x4...............................................guucucacccaacUgauccgg............................................      1
Gi3_27771121_x4..................................................................Cgguucgauucccggcgccggagc......................      1
Gi3_27721721_x4...................................................................gguucgauuGccggcgccggag.......................      1
Gi3_27704205_x4.................uccggcguaguauaacggcGaga........................................................................      1
Gi1_4031320_x4.................uccggcguaguauaacgCcuaga........................................................................      1
Gi3_27725409_x4...................Gggcguaguauaacggcuaga........................................................................      1
Gi5_8624351_x4...................................................................gguucgauucccggcgccggUg.......................      1
Gi2_10674607_x4.................uUcggcguaguauaacggcuagaau......................................................................      1
Gi5_8632475_x4...................................................................gguuUgauucccggcgcc...........................      1
Gi5_8631839_x4...............................................guucucaccUaacagauccgg............................................      1
Gi5_8609607_x4.................uccggcguaguUuaacggcuag.........................................................................      1
Gi5_8603259_x4.................uccggcgGaguauaacggcuag.........................................................................      1
Gi5_8619295_x4....................................................................guucgauucAcggcgccggagc......................      1
Gi5_8612051_x4..................................................cucacccaacagauccAg............................................      1
Gi2_10653287_x4.................uccggcguaguauaacUgcuaga........................................................................      1
Gi2_10688451_x4....................................................................guucgauucccggcgGcgg.........................      1
Gi3_27756361_x4.................Accggcguaguauaacggcuagaa.......................................................................      1
Gi1_4027604_x4...............................................guAcucacccaacagauccg.............................................      1
Gi5_8644299_x4.................uccggcguaguauaaAggcua..........................................................................      1
Gi3_27683097_x4....................................................................guNcgauucccggcgccgga........................      1
Gi2_10717623_x4...................................................................gguucgauuGccggcgccggagc......................      1
Gi3_27694377_x4....................................................................guucgauucUcggcgccggag.......................      1
Gi3_27795609_x4.....................................................................uCcgauucccggcgccggagc......................      1
Gi2_10720347_x4...............................................guucucacccaacagaucAg.............................................      1
Gi1_4042944_x4...................................................................gguucgauucccggcgcAggagc......................      1
Gi2_10665975_x4...................................................................gguucgauucUcggcgccgga........................      1
Gi1_4035960_x4............................................................agauccggguucgCuccc..................................      1
Gi5_8673704_x3...................................................................gguucgauucccggcgccggagcgCu...................      1
Gi5_8682932_x3.................uccggcguaguauaacUgcua..........................................................................      1
Gi5_8694299_x3.............................................ugguucucacccaacagaAccg.............................................      1
Gi2_10815952_x3..............................................gguucucacAcaacagauccg.............................................      1
Gi3_27892118_x3....................................................................guuNgauucccggcgccgg.........................      1
Gi1_4049865_x3................................................uucucacccaacUgauccg.............................................      1
Gi3_27935387_x3........................................................................Cauucccggcgccggagc......................      1
G1a_5855329_x3...................................................................Aguucgauucccggcgccgg.........................      1
Gi3_27978053_x3..................ccggcguaNuauaacggcuaga........................................................................      1
```

```
Gi5_8703620_x3.................................................ucucacccaacagaucUg.............................................     1
Gi5_8651117_x3................................................Gucucacccaacagauccggguuc........................................     1
Gi1_4071303_x3..................................................cucacccaacagaucUgg............................................     1
Gi2_10795318_x3................................................Gucucacccaacagauccgg............................................     1
Gi3_27859106_x3.....................Ucguaguauaacggcuaga........................................................................     1
Gi3_27823085_x3................................................uucucacAcaacagauccg.............................................     1
Gi2_10751776_x3....................................................................guucgauucccggcgccggU........................     1
Gi2_10765123_x3....................................................................guucCauucccggcgccgga........................     1
Gi3_27950240_x3..............................................................aGccggguucgauucccggcgccgga........................     1
Gi2_10822495_x3.................uccggcguaguaGaacggcua..........................................................................     1
Gi1_4069920_x3...............................................guucucacUcaacagauccg.............................................     1
Gi1_4057752_x3...................................................................gguucgaCucccggcgccgga........................     1
Gi3_27845336_x3..................................................cucacccaacagGuccgg............................................     1
Gi3_27941390_x3.....................................................................uucgauCcccggcgccggagc......................     1
Gi2_10816390_x3....................................................................guuNgauucccggcgccggagc......................     1
Gi5_8683016_x3.............................................ugguucuUacccaacagauccg.............................................     1
Gi5_8653355_x3...................................................................gguucgauuAccggcgccgga........................     1
Gi5_8697125_x3....................................................................guucgauucccgUcgccggagc......................     1
Gi5_8667383_x3.................uccggcgGaguauaacggcuaga........................................................................     1
Gi5_8698208_x3..................................................cucaGccaacagauccgg............................................     1
Gi1_4069074_x3.................uccggcguaguauaacggGuagaau......................................................................     1
Gi5_8692274_x3.................uccggcAuaguauaacgg.............................................................................     1
Gi2_10794589_x3......................................................................ucgauucccggcgccgAagc......................     1
Gi2_10743235_x3...................................................................gguucgauucccggcgcGggag.......................     1
Gi2_10781692_x3....................................................................guucgauucUcggcgccggag.......................     1
Gi3_27823253_x3..................Ucggcguaguauaacggcua..........................................................................     1
Gi5_8664656_x3.................................................ucucacccNacagauccgg............................................     1
Gi2_10821514_x3..............................................gguucucacccaaUagauccg.............................................     1
Gi5_8705780_x3................................................Aucucacccaacagauccg.............................................     1
Gi3_27894176_x3.................uccNgcguaguauaacggcuag.........................................................................     1
Gi2_10826089_x3..............................................gguucucacccaacUgauccg.............................................     1
Gi1_4066989_x3...............................................guuUucacccaacagauccgg............................................     1
Gi2_10745017_x3...................................................................gguucgauucccggcgccCgag.......................     1
Gi1_4055508_x3...................................................................gguucgauucccgAcgccgg.........................     1
Gi5_8657537_x3......................................................................Gcgauucccggcgccggag.......................     1
Gi1_4085529_x3....................................................................guuUgauucccggcgccg..........................     1
Gi2_10762363_x3.....................................................................uucgaCucccggcgccggagc......................     1
Gi3_27870452_x3...................................................................gguucgauucccggcgcU...........................     1
Gi2_10749043_x3...............aguccggcguaguauaGcggcuaga........................................................................     1
Gi1_4055634_x3.................................................ucucacccaacagauAcg.............................................     1
Gi5_8686355_x3.........................................................aacagauccggguucgauucccggcgccggagc......................     0
Gi1_4067886_x3..............................................................auccggguucgauuccAggcgccgg.........................     1
Gi5_8709440_x3...................................................................gguucgauAcccggcgccg..........................     1
Gi2_10792609_x3.................uccggcguaguauaacggcAa..........................................................................     1
Gi3_27829070_x3...................................................................gguucgauucccggcUccggag.......................     1
Gi1_4073733_x3.....................................................................uuUgauucccggcgccggagc......................     1
Gi3_27912119_x3.........................................................aacagauccggguucgauucccggcgccggagcC.....................     1
```

Gi5_8717162_x3..Aucacccaacagauccgg.. 1
Gi5_8661260_x3.................uccggcguaguaGaacggcuaga.. 1
G1a_5840212_x3...gguucgGuucccggcgcc........................... 1
Gi3_27869762_x3..gauucccgNcgccggagc...................... 1
Gi3_27970415_x3...uGcggguucgauucccggcgccgga........................ 1
Gi3_27896066_x3..................ccggcguGguauaacggcua.. 1
Gi5_8669315_x3..cccaacagauccggguCc.. 1
Gi5_8671166_x3..gguucucacccaacagGuccgg.. 1
Gi3_27869522_x3..guucgauucccgCcgccgg......................... 1
Gi5_8699177_x3...guuNucacccaacagauccg... 1
Gi5_8655155_x3.................uccggcguaguauaacUgcuag... 1
Gi5_8672801_x3...gguucgauucccgAcgccgg......................... 1
Gi1_4079256_x3.................uccggcAuaguauaacggcuag... 1
Gi3_27816947_x3...ggNucgauucccggcgcc........................... 1
Gi1_4079190_x3...ugguucucacccaacagaCccgg.. 1
Gi1_4089102_x3...ugguucuUacccaacagauccg... 1
Gi5_8715362_x3..guucgauucccggcgUcgga........................ 1
Gi3_27899402_x3..Aguucucacccaacagauccgggu.. 1
Gi1_4051395_x3...guucGcacccaacagauccgg.. 1
Gi1_4075257_x3...ucucacccaacagauccA... 1
Gi5_8666963_x3...gguucgauucccggcgccggaUc...................... 1
Gi1_4057833_x3...gguucgauucccUgcgccgg......................... 1
Gi3_27945962_x3...ugguucucacccaacagaucUg... 1
Gi3_27881726_x3...gguucgauucccgUcgccggag....................... 1
Gi5_8702807_x3..uucucCcccaacagauccg... 1
Gi1_4051002_x3..guucgauuccGggcgccgg......................... 1
Gi2_10804873_x3...gguucgauucccggcgAcgg......................... 1
Gi2_10801846_x3..guucgaAucccggcgccggagc...................... 1
Gi3_27959249_x3..gguucucacccUacagauccg... 1
Gi3_27818801_x3..guucgauuccAggcgccg.......................... 1
Gi3_27905579_x3.................uccggcguCguauaacggcuagaa... 1
Gi3_27886160_x3.................ucGggcguaguauaacgg... 1
Gi5_8655605_x3..guucgauucccggcgccAga........................ 1
Gi2_10726432_x3...gguucgauucGcggcgccggagc...................... 1
Gi1_4064631_x3.................uccggcguaguauCacggcu... 1
Gi5_8658845_x3...gguucgauuccUggcgcc........................... 1
Gi2_10815724_x3...guucucacccaacagaucUg... 1
Gi1_4083897_x3...................cggcguaguauaacggcuagaauU... 1
Gi1_4087308_x3..guucgGuucccggcgccg.......................... 1
Gi1_4057188_x3.................uccggcguaguauaacggcuGgaau.. 1
Gi5_8679263_x3...gguucgauucUcggcgcc........................... 1
Gi3_27857555_x3.................uccggcguUguauaacggcuag... 1
Gi2_10779409_x3..guucgCuucccggcgccggag....................... 1
Gi2_10730596_x3..guucgauucccggcgccC.......................... 1
Gi5_8659796_x3..uucucacccaacagaucUgg.. 1
Gi1_4056333_x3...gguucgauuccUggcgccg.......................... 1
Gi1_4059471_x3...ucucacccGacagauccg... 1

```
Gi2_10759840_x3......................................................................ucUauucccggcgccggagc......................    1
Gi5_8692985_x3.................uccggcguaguauaacggcuaCaau......................................................................    1
Gi1_4075299_x3...............................................gAucucacccaacagauccgg............................................    1
Gi1_4057344_x3.................uccggcguUguauaacggcuag.........................................................................    1
Gi3_27925016_x3...................................................................gguucgauuGccggcgccgga........................    1
Gi1_4048947_x3..................................................cuUacccaacagauccgg............................................    1
Gi3_27834674_x3............................................................aUauccggguucgauucccggcgccggagc......................    1
Gi3_27970070_x3................................................uucucacUcaacagauccg.............................................    1
Gi5_8670206_x3....................gUcguaguauaacggcuagaa.......................................................................    1
Gi1_4058496_x3..............................................gguucCcacccaacagauccgg............................................    1
Gi3_27927506_x3................................................uucucacccUacagauccg.............................................    1
Gi5_8652335_x3..................ccggcguaNuauaacggcuaga........................................................................    1
Gi3_27967724_x3......................................................................ucUauucccggcgccgga........................    1
Gi5_8711294_x3...................................................................gguucgauucccggcgccAgag.......................    1
Gi5_8702387_x3..................................................cucacccaacagaucUgg............................................    1
Gi2_10743196_x3....................................................................guucgauCcccggcgccggag.......................    1
Gi3_27805592_x3.................ucNggcguaguauaacggcua..........................................................................    1
Gi3_27827708_x3....................................................................guucgUuucccggcgccgg.........................    1
Gi3_27890375_x3.................................................................cggguucgauucccggcgccgC.........................    1
Gi2_10744741_x3.................uccggAguaguauaacggc............................................................................    1
Gi5_8708840_x3....................ggcguaguauaacggcuagG........................................................................    1
Gi3_27911921_x3...................................................................gguucgauGcccggcgccg..........................    1
Gi2_10811863_x3................................................uucucacAcaacagauccg.............................................    1
Gi1_4064877_x3.............................................ugguucucaccAaacagauccgg............................................    1
G1a_5853619_x3.................uUcggcguaguauaacggcuaga........................................................................    1
Gi5_8693198_x3....................Agcguaguauaacggcuaga........................................................................    1
Gi5_8656268_x3....................................................................guGcgauucccggcgccggagc......................    1
Gi1_4064754_x3.................uccgCcguaguauaacggcuaga........................................................................    1
Gi1_4054572_x3.................................................ucucacccGacagauccg.............................................    1
Gi5_8680883_x3....................ggcgCaguauaacggcuag.........................................................................    1
Gi5_8709635_x3.............................................ugguucucacccaacagGuccg.............................................    1
Gi1_4082253_x3....................................................................guucgauCcccggcgccgga........................    1
Gi5_8701718_x3...............................................................Gccggguucgauucccggc..............................    1
Gi3_27835121_x3.....................................................................uucgauucccgAcgccggagc......................    1
Gi1_4075113_x3....................................................cacccaacagauccgggGucgauuccc.................................    1
Gi3_27954461_x3...................................................................gguuAgauucccggcgccg..........................    1
Gi5_8708984_x3......................................................................Ccgauucccggcgccggagc......................    1
Gi1_4080930_x3.................Accggcguaguauaacggcua..........................................................................    1
Gi1_4060236_x3.............................................Ggguucucacccaacagaucc..............................................    1
Gi3_27944390_x3.................uccggcguaCuauaacggcuaga........................................................................    1
Gi1_4077060_x3................................................uucucacccaacUgauccgg............................................    1
Gi4_549507_x3 .................................................ucucacccaacagauccg............................................    0
Gi3_27890729_x3.................uccggcguaguauaaAggc............................................................................    1
Gi1_4052205_x3................................................uucucacccaacagauccgC............................................    1
Gi3_27851090_x3.................................................................Gggguucgauucccggcgccggag.......................    1
Gi2_10784437_x3.................uccAgcguaguauaacggcuag.........................................................................    1
Gi1_4060665_x3.................uccggcguCguauaacgg.............................................................................    1
```

Gi2_10743808_x3..Gcgauucccggcgccgga........................ 1
Gi3_27841136_x3....................ggUguaguauaacggcua.. 1
Gi5_8693645_x3...guucGcacccaacagauccg... 1
Gi5_8711072_x3.................ucAggcguaguauaacggcua.. 1
Gi2_10736971_x3...gguucgauucccggcgccgC......................... 1
Gi5_8667827_x3...ucucacccaacagauccgggG.. 1
Gi2_10783381_x3.................uccggcguagAauaacgg... 1
Gi5_8686904_x3.................uccAgcguaguauaacggcuagaa... 1
Gi3_27955472_x3..uAcucacccaacagauccgg.. 1
Gi5_8695475_x3.................uccggcguagGauaacggcuaga.. 1
Gi5_8660549_x3..guucgauuccAggcgccgg......................... 1
Gi2_10774735_x3..uGcucacccaacagauccgg.. 1
Gi5_8672081_x3...ugguucucacAcaacagauccg... 1
Gi2_10790146_x3...gguucgauucccggcgccgAa........................ 1
Gi3_27963113_x3..................Acggcguaguauaacggc.. 1
Gi5_8655068_x3.................uUcggcguaguauaacggcu... 1
Gi5_8652935_x3..guucgauuccAggcgccgga........................ 1
Gi3_27888362_x3...gguucgauucccgCcgccgg......................... 1
Gi3_27922772_x3.................uccggcguaguauaacggAuag... 1
Gi1_4065948_x3...gguCcgauucccggcgccg.......................... 1
Gi5_8669642_x3..uGcucacccaacagauccggguu... 1
Gi3_27814679_x3..ucgauucccggcgcAggagc...................... 1
Gi3_27813704_x3...uccgggCucgauucccggcgccgga........................ 1
Gi5_8673644_x3...gguucgauucccggcgcAggag....................... 1
G1a_5858230_x3..gGucgauucccggcgccgg......................... 1
Gi2_10772911_x3.................uccggcguaguauaacggcuG.. 1
Gi3_27838577_x3..uucccggcgccggagcCu.................... 1
Gi2_10799161_x3.................uccggcgAaguauaacgg... 1
Gi3_27947888_x3...gguucgauucccgCcgccg.......................... 1
Gi5_8691176_x3.................uccggcgAaguauaacgg... 1
Gi5_8712632_x3..guuNgauucccggcgccgg......................... 1
Gi2_10828222_x3...gguucgauucccCgcgccgga........................ 1
Gi2_10783765_x3..guucgauucccggcgccgU......................... 1
Gi1_4068717_x3...gguucgauucccggcgUcgga........................ 1
Gi1_4064415_x3.................Accggcguaguauaacggcuaga.. 1
Gi1_4061523_x3..................ccggcguGguauaacggcua.. 1
Gi3_27933464_x3..ucgauucccggcgccggagcCu.................... 1
Gi1_4076667_x3..guucgauucccggAgccg.......................... 1
Gi2_10734631_x3.................Accggcguaguauaacggcuagaa... 1
Gi5_8652134_x3.................uccggcgAaguauaacggcuag... 1
Gi5_8661995_x3...guucucacccaaGagauccg... 1
Gi5_8673773_x3...Cgguucucacccaacagauccgg.. 1
Gi3_27828017_x3..aUauccggguucgauucccggcgccggag....................... 1
Gi2_10823227_x3..guucgauucccAgcgccggag....................... 1
Gi2_10792240_x3...gguucgauucccggcgccgA......................... 1
Gi1_4059540_x3...gguucAauucccggcgccg.......................... 1
Gi5_8700377_x3...uuUgauucccggcgccgg......................... 1

```
Gi5_8653064_x3........................................................................Cauucccggcgccggagc......................      1
Gi4_551532_x3 ................................................uucucacccaacagauccgg............................................      0
Gi3_27925592_x3.................uccNgcguaguauaacggcua..........................................................................      1
Gi3_27940733_x3...................................................................gguuAgauucccggcgcc...........................      1
Gi3_27920717_x3..............................................ggAucucacccaacagaucc..............................................      1
Gi3_27967733_x3.................uccggcgAaguauaacggcua..........................................................................      1
Gi2_10791622_x3...................................................................gguuAgauucccggcgccggag.......................      1
G1a_5845960_x3....................................................................guucgauucccggcgccggagcCu....................      1
Gi1_4057098_x3.................uccggcguaguauaacggAuag.........................................................................      1
Gi2_10813858_x3..................ccggcguaguauaacggcuaga........................................................................      0
Gi3_27865328_x3....................ggcguaguauaGcggcuagaau......................................................................      1
Gi2_10796617_x3....................................................................guucgauucccggcgcGgg.........................      1
Gi2_10800736_x3...................................................................gguucCauucccggcgccggag.......................      1
Gi2_10785715_x3......................................................................ucgauucAcggcgccggagc......................      1
Gi5_8668376_x3.................uccggcguUguauaacggcuagaau......................................................................      1
Gi2_10814320_x3.................uccggcguaguCuaacggcuagaa.......................................................................      1
Gi5_8692454_x3...................................................................gguucgauucccggUgccggag.......................      1
Gi2_10759252_x3..................Gcggcguaguauaacggcua..........................................................................      1
Gi5_8689922_x3.................uccggcguagAauaacggcuagaau......................................................................      1
Gi3_27945524_x3...............................................guucucacccaacagauccA.............................................      1
Gi5_8701349_x3.................uccggcguaguauaacggcAagaau......................................................................      1
Gi5_8710403_x3.................................................ucuUacccaacagauccg.............................................      1
Gi1_4058463_x3....................................................................guucgauucccgUcgccgga........................      1
Gi2_10821295_x3.................uccgAcguaguauaacggcua..........................................................................      1
Gi5_8671748_x3................................................uAcucacccaacagauccg.............................................      1
Gi1_4075866_x3.............................................ugUuucucacccaacagauccg.............................................      1
Gi3_27804551_x3...................................................................gguucgauucccggGgccg..........................      1
Gi5_8663876_x3................................................uucuUacccaacagauccg.............................................      1
Gi5_8688164_x3....................................................................guucgauucUcggcgccgga........................      1
Gi3_27878051_x3..............................................gguuGucacccaacagauccg.............................................      1
Gi2_10763971_x3.................uccgAcguaguauaacgg.............................................................................      1
Gi1_4061901_x3....................ggUguaguauaacggcuag.........................................................................      1
Gi4_549306_x3 .................Gccggcguaguauaacggcuag.........................................................................      1
Gi3_27834272_x3....................................................................guuAgauucccggcgccggagc......................      1
Gi2_10765033_x3................................................uucucacccaacagauAcg.............................................      1
Gi5_8705831_x3....................................................................gAucgauucccggcgccgga........................      1
Gi3_27812366_x3...................................................................gguucgauucGcggcgccggag.......................      1
Gi2_10791523_x3...................................................................gguucgauuUccggcgccgg.........................      1
Gi3_27884132_x3....................................................................guucgaAucccggcgccgga........................      1
Gi3_27818741_x3....................................................................guucgUuucccggcgccggagc......................      1
Gi5_8675351_x3...................................................................gguucgauucAcggcgccgga........................      1
Gi1_4081536_x3...............................................Cuucucacccaacagauccgg............................................      1
Gi2_10776085_x3...................................................................gguucUauucccggcgcc...........................      1
Gi5_8667017_x3..............................................gguucucacccaaUagauccg.............................................      1
Gi3_27849311_x3....................................................................guucgauucccggcgccggagG......................      1
Gi5_8661062_x3................................................Gucucacccaacagauccggguu.........................................      1
Gi5_8684414_x3............................................................agauccgggGucgauuccc.................................      1
```

Gi5_8701292_x3..guucgauucccUgcgccgg......................... 1
Gi2_10788433_x3..ggAucucacccaacagauccg... 1
G1a_5825536_x3.................uccggcguaguGuaacggcuaga.. 1
Gi3_27876740_x3..guuAgauucccggcgccgga........................ 1
Gi2_10761202_x3...gguucgauucUcggcgccgg......................... 1
Gi1_4079502_x3...uucgaCucccggcgccgg......................... 1
Gi1_4076907_x3...gguucgauucccggUgccgga........................ 1
Gi5_8676506_x3...Acacccaacagauccggguu... 1
Gi1_4053543_x3.................uUcggcguaguauaacggc.. 1
Gi5_8718017_x3.................uccggcguaguUuaacggc.. 1
Gi1_4089273_x3...gguucgaCucccggcgccg.......................... 1
Gi3_27957008_x3...guucucacccaacagauAcgg.. 1
Gi3_27961436_x3..uucucaccUaacagauccg... 1
G1a_5874976_x3.................uccggcguaguauaaUggcuag... 1
Gi3_27841766_x3...guucCcacccaacagauccg... 1
Gi5_8700323_x3.................uccUgcguaguauaacggcuaga.. 1
Gi3_27958253_x3..uUgauucccggcgccggag....................... 1
Gi2_10803223_x3..guuGgauucccggcgccgga........................ 1
Gi2_10803289_x3...gguucgauucAcggcgccg.......................... 1
Gi3_27949205_x3..guucgauAcccggcgccg.......................... 1
Gi5_8672588_x3...gguucgauucccCgcgccggagc...................... 1
Gi2_10735141_x3.................uccggcguaguaCaacggcuagaau.. 1
Gi3_27917921_x3..ggguucgauucccggcgccgg......................... 0
Gi2_10814698_x3.................uccggcguaguauCacgg... 1
Gi3_27824288_x3.................uccggcguagAauaacggcua.. 1
Gi4_551673_x3 ....uccggcguaguauaacggcua.. 0
Gi5_8683721_x3...................cggcguGguauaacggcua.. 1
Gi3_27906098_x3...uucgauuUccggcgccgga........................ 1
Gi2_10816714_x3..gguucucacccUacagauccg... 1
Gi3_27877115_x3..Nuucgauucccggcgccggag....................... 1
Gi2_10817857_x3.................uccggcgGaguauaacggcuaga.. 1
Gi5_8669591_x3...gguucgauucccggAgccgg......................... 1
Gi3_27798860_x3..uucucacccaacagaucAgg.. 1
Gi5_8652497_x3..guucgauucccggcgcccCga....................... 1
Gi3_27978362_x3.................uccggcguaguauaacCg... 1
Gi3_27963530_x3...gguucgauucccggcgcA........................... 1
Gi1_4083423_x3...gguucgauucccggUgccggagc...................... 1
Gi5_8688872_x3...................Gggcguaguauaacggcuag... 1
Gi5_8657915_x3...gguucgauucccUgcgccg.......................... 1
Gi2_10725721_x3.................uccggcguaguauaacggcuUgaau.. 1
Gi1_4078875_x3...guucucacccaaAagauccgg.. 1
Gi1_4065267_x3.................uccggcguaguauaacggcAa.. 1
G1a_5850052_x3..guucgaCucccggcgccggagc...................... 1
Gi5_8675003_x3...ugUuucucacccaacagauccg... 1
Gi3_27843638_x3...gguAcgauucccggcgccgg......................... 1
Gi3_27911900_x3..guucCauucccggcgccgga........................ 1
Gi3_27925910_x3...cgauuccUggcgccggagc...................... 1

```
Gi2_10795651_x3...................................................................gguucgauuAccggcgccgga........................     1
Gi5_8664269_x3....................................................................gguucgauuccUggcgccggag.......................     1
Gi3_27821048_x3...................................................................gguucgauucccgUcgccggagc......................     1
Gi5_8668877_x3..................Ucggcguaguauaacggcuag.........................................................................     1
Gi1_4060656_x3.................uccggcguaguCuaacggc............................................................................     1
Gi5_8661461_x3....................................................................Auucgauucccggcgccggag.......................     1
Gi2_10761232_x3.................uccggcguaguauaacggUua..........................................................................     1
Gi5_8663273_x3....................................................................Uuucgauucccggcgccggagcguuucc................     1
Gi2_10822213_x3.......................................................................cgauucccNgcgccggagcguuu..................     1
Gi1_4058412_x3.................................................ucucacccaacGgauccg.............................................     1
Gi3_27842411_x3...................................................................gguucgauuccGggcgccgg.........................     1
G1a_5853931_x3....................................................................guucgGuucccggcgccggagc......................     1
Gi1_4073901_x3......................................................................uUgauucccggcgccggagc......................     1
Gi1_4074957_x3....................................................................guucgauucccCgcgccgga........................     1
Gi2_10796326_x3........................................................................gUuucccggcgccggagc......................     1
Gi5_8708177_x3...............................................gAucucacccaacagauccg.............................................     1
Gi3_27813083_x3.................................................................cggguucgNuucccggcgccgga........................     1
Gi1_4057590_x3.............................................ugguucucacccaacagauc...............................................     0
Gi5_8712719_x3................................................uucucacAcaacagauccgg............................................     1
Gi1_4060890_x3.................uccggcguCguauaacggcuag.........................................................................     1
Gi3_27826490_x3..................ccggcguaNuauaacggc............................................................................     1
Gi5_8657486_x3................................................uucucacccaacagaCccgg............................................     1
Gi3_27932771_x3...................................................................gguucgauucccCgcgccg..........................     1
Gi5_8716610_x3...................................................................gguucgauucccggcgcU...........................     1
Gi1_4074375_x3.............................................ugguucucacccaacagauccA.............................................     1
Gi3_27828476_x3...............................................Auucucacccaacagauccgg............................................     1
Gi2_10753882_x3...................................................................gguucgauucccggcgccggaC.......................     1
Gi3_27846707_x3......................................................................Acgauucccggcgccgga........................     1
Gi2_10809373_x3.................uUcggcguaguauaacggcuagaa.......................................................................     1
Gi3_27877406_x3...............................................guucucacccaacagauUcg.............................................     1
Gi5_8673065_x3......................................................................ucgauucccggcgccggagcCu....................     1
Gi4_547647_x3 ...................................................................gguucgauucccggcgccggag.......................     0
Gi1_4049922_x3.................ucUggcguaguauaacggcu...........................................................................     1
Gi1_4056942_x3...............................................guucucacccaacagCuccg.............................................     1
Gi2_10755211_x3......................................................cGcaacagauccggguuc........................................     1
Gi2_10773238_x3.................uccggcguaguauaacggUuagaau......................................................................     1
Gi1_4057824_x3....................................................................guucgauCcccggcgccg..........................     1
Gi3_27839147_x3..................ccggcguaguauaacggcCaga........................................................................     1
Gi3_27949622_x3.................uccggcguaguauaacggcuaCaau......................................................................     1
Gi1_4072857_x3....................................................................guucgauucccggcgccgCa........................     1
Gi2_10779490_x3....................................................................guucgaGucccggcgccgg.........................     1
Gi1_4074498_x3................................................Aucucacccaacagauccg.............................................     1
G1a_5843290_x3.................uccgUcguaguauaacggcuag.........................................................................     1
Gi4_550752_x3 ...................................................................ggGucgauucccggcgccgg.........................     1
Gi2_10750684_x3...................................................................gguucgauucccggAgccgga........................     1
Gi1_4075806_x3....................................................................Nuucgauucccggcgccgg.........................     1
Gi5_8683811_x3...................................................................gCuucgauucccggcgcc...........................     1
```

```
Gi2_10799182_x3.................uccggcguaguauaacggcuagCa.......................................................................     1
Gi2_10769530_x3.................uccggcguaguaGaacggc............................................................................     1
Gi1_4085451_x3................................................uucucaAccaacagauccg.............................................     1
Gi2_10782331_x3.............................................ugguucucacccGacagauccg.............................................     1
Gi2_10824061_x3...................................................................gguucgauucAcggcgcc...........................     1
G1a_5875426_x3...................................................................gguCcgauucccggcgccgg.........................     1
Gi3_27874355_x3...............................................................uccggguuNgauucccggcgccgga........................     1
Gi2_10729030_x3.................uccggcAuaguauaacggc............................................................................     1
Gi2_10753012_x3..................................................................ggguucgauucccggcgccggagcC.....................     1
Gi3_27960371_x3...............................................guucucGcccaacagauccg.............................................     1
Gi2_10795600_x3...................................................................gguucgauucccggcgccUgag.......................     1
Gi3_27937088_x3....................................................................Nuucgauucccggcgccggagc......................     1
Gi2_10776802_x3....................................................................guucgauucccggAgccgga........................     1
Gi2_10783849_x3.................uccggcguCguauaacggcuagaa.......................................................................     1
Gi2_10817479_x3.................uccggcguaguUuaacggc............................................................................     1
Gi5_8714924_x3.................uccggcguaguauGacggc............................................................................     1
Gi3_27967205_x3...................cUgcguaguauaacggcuaga........................................................................     1
Gi1_4082823_x3...................................................................gAuucgauucccggcgccgga........................     1
Gi3_27976247_x3.............................................ugguCcucacccaacagauccgg............................................     1
Gi5_8694740_x3................................................uucucacccaacagauccA.............................................     1
Gi1_4081971_x3...................................................................ggGucgauucccggcgccggagc......................     1
Gi3_27813977_x3......................................................................ucgaCucccggcgccgga........................     1
Gi1_4087377_x3...................................................................gAuucgauucccggcgccggagc......................     1
Gi2_10813018_x3......................................................ccUaacagauccggguuc........................................     1
Gi2_10767859_x3...................................................................Nguucgauucccggcgccggagc......................     1
Gi2_10754527_x3.....................................agaaugguugguucCcacc........................................................     1
Gi3_27871745_x3.................uccggcguaguauaacCgcuagaau......................................................................     1
Gi1_4065120_x3.................................................ucucacccNacagauccgg............................................     1
Gi2_10776658_x3.............................................uCguucucacccaacagauccg.............................................     1
Gi5_8705954_x3...............................................guucucacccaacagaucUgg............................................     1
Gi5_8691035_x3..............................................gCuucucacccaacagauccg.............................................     1
Gi1_4051380_x3.................uccggcguaguauaacggcuagCa.......................................................................     1
Gi3_27890927_x3.................Cccggcguaguauaacggcu...........................................................................     1
Gi3_27923198_x3.................uccNgcguaguauaacggc............................................................................     1
Gi5_8652026_x3..............................................gguucucacccaacagUuccg.............................................     1
Gi2_10741264_x3.................uccggcguagAauaacggcuag.........................................................................     1
Gi1_4077711_x3..............................................gguucucCcccaacagauccg.............................................     1
Gi3_27843773_x3.................uccggcguaguauaacggcAa..........................................................................     1
Gi5_8714861_x3.................uccggcguaguauaUcggcuaga........................................................................     1
Gi5_8669648_x3....................................................................guucgauucccggcgccUgagc......................     1
Gi3_27816380_x3.................Nccggcguaguauaacggcuaga........................................................................     1
Gi2_10825423_x3.................uccggcguUguauaacggcua..........................................................................     1
Gi3_27893051_x3.................uAcggcguaguauaacggc............................................................................     1
Gi2_10803628_x3.............................................ugguucCcacccaacagauccg.............................................     1
Gi2_10779790_x3...................................................................gguucgauucccggcCccggag.......................     1
Gi3_27892247_x3...................................................................gguucgauucccggcgcGgga........................     1
Gi3_27849806_x3.................uccggcguagAauaacggc............................................................................     1
```

Gi5_8686973_x3.................uccggcguaguauaaAggc.. 1
Gi2_10799380_x3.................uccggcguaAuauaacggc.. 1
G1a_5836006_x3...gguucgauucccggcAccggagc...................... 1
Gi2_10759366_x3...gGucucacccaacagauccgg.. 1
Gi4_552126_x3 ....guucgaCucccggcgccggagc...................... 1
Gi3_27906026_x3..Gucucacccaacagauccggguuc.. 1
Gi5_8684423_x3.................uccggcguaguauaacggAuaga.. 1
Gi5_8699609_x3..ucgauucccgAcgccgga........................ 1
Gi3_27913643_x3..agauccggguucgUuuccc................................. 1
Gi2_10824511_x3...uggCucucacccaacagauccg... 1
Gi1_4082856_x3..................ccggcguaNuauaacggcuag... 1
Gi5_8697452_x3....................gUcguaguauaacggcua.. 1
G1a_5864050_x3.................Accggcguaguauaacggcuag... 1
Gi2_10730764_x3...uCcgauucccggcgccgg......................... 1
Gi5_8676110_x3..ucgauuccNggcgccggag....................... 1
Gi1_4074720_x3..auccggguucgauucccggcgccAg......................... 1
Gi3_27821375_x3..ucgauCcccggcgccggagc...................... 1
Gi5_8669372_x3..gguucucacccaacagaAccg... 1
Gi2_10792870_x3...ugguucucacccaCcagauccg... 1
Gi1_4078809_x3...gguucgauucccggcgccggagcCuu................... 1
Gi3_27974477_x3..gguuUucacccaacagauccg... 1
Gi2_10761703_x3..guucgauucccggcgcGgga........................ 1
Gi5_8675369_x3..Gcgauucccggcgccgga........................ 1
Gi2_10780225_x3..Aauucccggcgccggagc...................... 1
Gi2_10804204_x3..guucgauucccggcgccgUagc...................... 1
Gi2_10760845_x3..uucucacccaUcagauccg... 1
G1a_5853448_x3..uGcucacccaacagauccgg.. 1
Gi1_4049988_x3...guAcucacccaacagauccgg.. 1
Gi2_10783159_x3..guucgauuccGggcgccgg......................... 1
Gi2_10769323_x3...auucccggcgccggagcCu.................... 1
Gi1_4079901_x3...ggAucgauucccggcgccggagc...................... 1
Gi3_27849674_x3...gguucgauuGccggcgccgg......................... 1
Gi3_27849536_x3.................uccggcguGguauaacggcu... 1
Gi3_27883871_x3...uucgauucccggcgccggagcgA.................... 1
Gi3_27827657_x3..gAucgauucccggcgccg.......................... 1
Gi1_4048995_x3...gguucgaCucccggcgccggagc...................... 1
Gi3_27948239_x3.................uccggcguaguauaacgCc.. 1
Gi1_4071780_x3.................Cccggcguaguauaacggc.. 1
Gi2_10788475_x3..ucgauucccggcgccAgagc...................... 1
Gi5_8666495_x3...ucacccaGcagauccggguu... 1
Gi1_4075323_x3..ucgauucccggcgccggag....................... 0
Gi5_8698340_x3...gguucgauucccggcgccCg......................... 1
Gi3_27864263_x3..guucgauucccggcgccU.......................... 1
Gi3_27860786_x3.................uccggcguaguauaacggcuagaU... 1
Gi5_8716589_x3..guucgauucccggAgccgga........................ 1
Gi3_27936176_x3..gauucccggcAccggagc...................... 1
Gi4_544908_x3 ....gguucgauNcccggcgccgg......................... 1

Gi3_27893876_x3...guucucCcccaacagauccg... 1
Gi5_8677196_x3...ucucacccaacagGuccgggu.. 1
Gi1_4074180_x3...ucAcacccaacagauccg... 1
Gi5_8667416_x3..uucucacccaacUgauccg... 1
Gi3_27832424_x3...ugguucucacccaacGgauccgg.. 1
Gi5_8684228_x3.................uccggcguaguauaacUgcuagaau.. 1
Gi5_8658149_x3..guGcgauucccggcgccgga........................ 1
Gi3_27834023_x3..guuNgauucccggcgccgga........................ 1
Gi1_4080300_x3...gguuAgauucccggcgccgg......................... 1
Gi5_8700176_x3..ggCucucacccaacagauccgg.. 1
Gi3_27805868_x3..guuUcacccaacagauccgg.. 1
Gi1_4051707_x3.................ucUggcguaguauaacggcuagaa... 1
G1a_5837905_x3.................uccggcguGguauaacggc.. 1
Gi5_8682884_x3...cagauccgggCucgauucccggcgccg.......................... 1
Gi2_10771150_x3.................uccgCcguaguauaacggcuaga.. 1
Gi3_27937673_x3...Uauccggguucgauucccggcgccgga........................ 1
Gi3_27836138_x3..uucucacccaaUagauccgg.. 1
Gi5_8653148_x3..guucgauucccggcgcAggagc...................... 1
Gi3_27829607_x3...ugUuucucacccaacagauc... 1
Gi2_10759411_x3.................uccggcguaguauaacggcuCg... 1
Gi3_27830687_x3...gguucgauucccCgcgccgga........................ 1
Gi5_8699711_x3.................uccggcguaguauaacggcuagUau.. 1
Gi3_27818648_x3.................ucAggcguaguauaacggc.. 1
Gi5_8653427_x3..uucucacccaCcagauccggguu... 1
Gi1_4080075_x3..uucucCcccaacagauccg... 1
Gi2_10749778_x3...gguAcgauucccggcgccgga........................ 1
Gi1_4089084_x3..guucgauuUccggcgccg.......................... 1
Gi5_8674208_x3...................cggcguaguauaacggcuagaGu.. 1
Gi3_27887441_x3..cucacccGacagauccgg.. 1
Gi3_27974735_x3...ugUuucucacccaacagauccg... 1
Gi2_10821682_x3...gguucgauucccUgcgcc........................... 1
Gi1_4077450_x3..uucucacccaacagauccgU.. 1
Gi3_27844193_x3...ugguucucacccaacagCuccg... 1
Gi2_10822219_x3...gguucgauucccggcgccggagcg..................... 0
Gi1_4064262_x3.................uccggcguaguauaacggcuGgaa... 1
Gi2_10740964_x3..gguucucacccaacagauAcg... 1
Gi1_4065225_x3.................uccggcguaguauaacggcuUgaau.. 1
Gi5_8665940_x3...Gcucacccaacagauccgggu.. 1
Gi5_8685422_x3.................uccggcguagAauaacggcuaga.. 1
Gi2_10784119_x3...gguAcgauucccggcgccg.......................... 1
Gi2_10804822_x3.................uccggcguaguauGacggcuagaau.. 1
Gi5_8688299_x3..guucgCuucccggcgccggagc...................... 1
Gi2_10784854_x3...gguucgauAcccggcgccg.......................... 1
Gi1_4059102_x3.................uccggcguaguauaaUgg... 1
Gi2_10746955_x3..guucgaGucccggcgccgga........................ 1
Gi2_10751710_x3.................Accggcguaguauaacggcuag... 1
Gi5_8711684_x3...gguucAauucccggcgccgga........................ 1

```
Gi3_27798551_x3.............................................Cgguucucacccaacagauc...............................................     1
Gi3_27896120_x3.....................................................................uucgauuccUggcgccgga........................     1
Gi5_8657960_x3.................uccggcguaguauaacgCc............................................................................     1
Gi1_4077123_x3..............................................gguucucacAcaacagauccg.............................................     1
Gi5_8705318_x3.................................................Acucacccaacagauccg.............................................     1
Gi1_4055982_x3.................uccggcguagCauaacggcuaga........................................................................     1
Gi5_8663615_x3.................................................ucucacccGacagauccg.............................................     1
Gi3_27804740_x3.............................................ugguucucacccaacGgauc...............................................     1
Gi5_8668661_x3.................uccggcguaguauaacggAua..........................................................................     1
Gi5_8652128_x3....................................................................guucgauucccgUcgccgga........................     1
Gi1_4077942_x3.................uccggcguaguauUacggcuaga........................................................................     1
Gi5_8658503_x3...................................................................ggAucgauucccggcgccggag.......................     1
Gi5_8672123_x3...................................................................gguucgauucUcggcgccgg.........................     1
Gi2_10744147_x3.................uccggcguaAuauaacggcua..........................................................................     1
Gi1_4054593_x3..................................................cucacccGacagauccgg............................................     1
Gi1_4077459_x3.................ucAggcguaguauaacggcuag.........................................................................     1
Gi5_8655893_x3....................................................................guucCauucccggcgccgg.........................     1
Gi5_8678831_x3...................................................................gguucgaAucccggcgccg..........................     1
Gi2_10790641_x3...................................................................gguucgauuUccggcgccg..........................     1
Gi3_27867647_x3...................................................................Nguucgauucccggcgccggag.......................     1
Gi4_548499_x3 ...................................................................gguucgauucccggcgcc...........................     0
Gi5_8653847_x3.....................................................................uCcgauucccggcgccgg.........................     1
Gi2_10751311_x3...............................................guucucacccaGcagauccg.............................................     1
Gi5_8659568_x3.............................................ugguucucacccaacagauccgggC..........................................     1
Gi3_27926360_x3.....................................................................Aucgauucccggcgccggagc......................     1
Gi3_27822023_x3..............................................................auUcggguucgauucccggcgccgga........................     1
Gi5_8689874_x3.............................................ugguucucacccaacagCuccg.............................................     1
Gi3_27797651_x3.............................................ugguucucNcccaacagauccgg............................................     1
Gi3_27909551_x3.....................................................................uucgauuccUggcgccggagc......................     1
Gi2_10816255_x3......................................................cccaacagaCccggguuc........................................     1
Gi5_8689247_x3..............................................Aguucucacccaacagauccggguu.........................................     1
Gi1_4071663_x3...................................................................gguucgauucccggcgcUgga........................     1
Gi1_4052016_x3.................uccggcguaguauaacgAcua..........................................................................     1
Gi5_8671412_x3................................................................ccggguucgauucccggcgccggC........................     1
Gi3_27821294_x3.................uAcggcguaguauaacggcuag.........................................................................     1
Gi3_27930038_x3..............................................gguucucaUccaacagauccg.............................................     1
Gi5_8695913_x3...................................................................gguucgauucccggcgccgCa........................     1
Gi2_10744057_x3................................................Gucucacccaacagauccggguucg.......................................     1
Gi2_10754875_x3.................uccAgcguaguauaacggc............................................................................     1
Gi5_8656658_x3...................................................................gguNcgauucccggcgccggagc......................     1
Gi2_10744978_x3.................uccggcguaguCuaacggcuag.........................................................................     1
Gi1_4062306_x3.................uccggcAuaguauaacggcua..........................................................................     1
Gi5_8715050_x3...............................................guucucacccaacagaCccgg............................................     1
Gi5_8702369_x3..................ccggcguaguauaacggcCaga........................................................................     1
Gi5_8703722_x3..............................................gguucucacccaacagaGccg.............................................     1
Gi5_8692604_x3..................................................cucUcccaacagauccgg............................................     1
Gi1_4061478_x3...............................................guucucacAcaacagauccg.............................................     1
```

Gi3_27863351_x3.................ucAggcguaguauaacggcuag... 1
Gi2_10785745_x3...gguucgauucccggcgcGgga........................ 1
Gi5_8703035_x3.................uccggcAuaguauaacggcua.. 1
Gi1_4088490_x3...gguucgauucccggcgccggGg....................... 1
Gi5_8678750_x3.................uccggcguCguauaacggcuagaau.. 1
Gi5_8706569_x3...guucucacccaGcagauccgg.. 1
Gi1_4088190_x3...gguucgauucUcggcgccgga........................ 1
Gi1_4060851_x3...guucuAacccaacagauccgg.. 1
Gi3_27975029_x3..gUuucccggcgccggagc...................... 1
Gi2_10822264_x3...guucucacccaaAagauccg... 1
Gi5_8713547_x3..guucgauuGccggcgccgg......................... 1
Gi3_27911471_x3..gUguucgauucccggcgccggagc...................... 1
Gi1_4047990_x3..uucGcacccaacagauccg... 1
Gi2_10822114_x3...gguuGgauucccggcgccgga........................ 1
Gi1_4077564_x3...gguucgGuucccggcgccggagc...................... 1
Gi1_4058406_x3...guucucacccUacagauccg... 1
Gi2_10745776_x3...gguuAgauucccggcgccgga........................ 1
Gi2_10794754_x3...gguucAauucccggcgccg.......................... 1
Gi3_27948005_x3..guucgauucccggGgccggagc...................... 1
Gi3_27914537_x3..guucgauucccUgcgccgga........................ 1
Gi1_4060380_x3..uucucaUccaacagauccgg.. 1
Gi5_8714744_x3.................uccggcguaguaGaacggcuagaau.. 1
Gi3_27893261_x3..guucgauuAccggcgccggag....................... 1
Gi5_8680985_x3..gGuucccgccuuccuucgucu. 1
Gi1_4083678_x3.................uGcggcguaguauaacggcuagaa... 1
Gi3_27886430_x3..guuGgauucccggcgccgga........................ 1
Gi1_4056021_x3.................ucAggcguaguauaacgg... 1
Gi5_8692295_x3...................cggcguaguauaacggcuCga.. 1
Gi5_8715074_x3.................uccggcguaguUuaacgg... 1
Gi2_10790677_x3..guucgauucccggGgccggagc...................... 1
Gi1_4063719_x3..................ccggUguaguauaacggcua.. 1
Gi3_27884855_x3...uucgauuUccggcgccggagc...................... 1
Gi5_8683430_x3.................uccggcguaguauaacggcuaAaau.. 1
Gi3_27804281_x3...ucucacccaacagGuccg... 1
G1a_5815924_x3.................uccggcgCaguauaacggcuag... 1
Gi5_8661392_x3.................uccgUcguaguauaacggc.. 1
Gi3_27900356_x3...guucucacUcaacagauccgg.. 1
Gi5_8709053_x3.................uccgCcguaguauaacggcua.. 1
Gi1_4085040_x3..................ccggcguaguauaacggcCaga.. 1
Gi2_10773382_x3.................uccggcguaguauaGcggcuagaa... 1
Gi3_27808544_x3...guucucacccaacagaucccC.. 1
Gi2_10748152_x3...Gucgauucccggcgccggag....................... 1
Gi2_10786429_x3..uGgauucccggcgccggag....................... 1
Gi1_4060719_x3...gguucgauucAcggcgccgg......................... 1
Gi5_8671859_x3.................uccggcguaUuauaacggcuag... 1
Gi5_8706995_x3...gguucgauucccggcgcA........................... 1
Gi2_10797733_x3..gAuucucacccaacagauccg... 1

```
Gi3_27955169_x3...................................................................gguucUauucccggcgccg..........................     1
Gi3_27875945_x3............................................................agauccggguCcgauuccc.................................     1
Gi3_27906479_x3.................uccgAcguaguauaacggcuag.........................................................................     1
Gi3_27868535_x3...........................................................cagauccggguCcgauuccc.................................     1
Gi2_10801753_x3.................uccggcguaguauaacggcuagaGu......................................................................     1
Gi5_8704106_x3.................uccggcguaguauaaAggcuagaau......................................................................     1
Gi3_27901448_x3.............................................ugguucAcacccaacagauccg.............................................     1
Gi3_27905078_x3...............................................guucucacccUacagauccg.............................................     1
Gi2_10783561_x3................................................uucucacccCacagauccg.............................................     1
Gi1_4060953_x3................................................uucuUacccaacagauccg.............................................     1
Gi1_4073718_x3....................................................................guucgauuUccggcgccggagc......................     1
Gi1_4070532_x3.............................................ugguucucacccaacCgauccgg............................................     1
Gi2_10779721_x3................................................uucuUacccaacagauccg.............................................     1
Gi5_8683466_x3.................uccggcguUguauaacggc............................................................................     1
Gi5_8690372_x3.................uccggcguaguauaacggcuaC.........................................................................     1
Gi1_4063026_x3....................................................................gAucgauucccggcgccgga........................     1
Gi3_27922640_x3......................................................................ucgauucccggUgccgga........................     1
Gi5_8681225_x3....................ggcguaguNuaacggcuag.........................................................................     1
Gi2_10752562_x3.................Cccggcguaguauaacggcuagaa.......................................................................     1
Gi3_27819824_x3...................................................................gguuAgauucccggcgccgg.........................     1
Gi2_10762984_x3...............................................guucucGcccaacagauccg.............................................     1
Gi3_27931829_x3......................................................................ucgauuUccggcgccggag.......................     1
Gi1_4065504_x3....................................................................guucAauucccggcgccg..........................     1
Gi1_4073493_x3...............................................guucucacccaGcagauccgg............................................     1
Gi3_27820343_x3......................................................................ucAauucccggcgccggagc......................     1
Gi5_8663858_x3...................................................................gguucgauCcccggcgccgga........................     1
Gi3_27905309_x3...........................................................cagauccgNguucgauuccc.................................     1
Gi2_10778767_x3...................................................................gguucgauuccAggcgccggag.......................     1
Gi1_4063590_x3....................................................................guucgauucccggcgcccCga.......................     1
Gi1_4048029_x3...................................................................gguucgauucccggcgccggagA......................     1
Gi2_10746802_x3...................................................................gAuucgauucccggcgccgga........................     1
Gi3_27874574_x3.................uccggcguaguauaacggcAag.........................................................................     1
Gi5_8692001_x3...............................................guucucacccaacCgauccgg............................................     1
Gi2_10811659_x3...................................................................gguucgauucccggcgGc...........................     1
Gi2_10811437_x3....................................................................guucgauucAcggcgccg..........................     1
Gi3_27939230_x3..................................................................ggguucgauucccggcgccggCgc......................     1
Gi2_10756783_x3...................................................................gguucgauAcccggcgcc...........................     1
Gi3_27913328_x3........................................................caacagauNcggguucgauuccc.................................     1
Gi5_8684030_x3...................................................ucacccaacagauccgggGu.........................................     1
Gi1_4063806_x3..............................................gguucAcacccaacagauccg.............................................     1
Gi1_4057449_x3...................................................................gguucgauucccggcgccAgagc......................     1
Gi1_4073049_x3................................................uucucacccaacaAauccg.............................................     1
Gi2_10781722_x3.................uccAgcguaguauaacggcuagaau......................................................................     1
Gi3_27978749_x3.................uccggcAuaguauaacggcuag.........................................................................     1
Gi1_4088958_x3...............................................guucucaUccaacagauccgg............................................     1
Gi5_8694449_x3...................................................................gguucgauucccggcgccCgagc......................     1
Gi3_27796832_x3...............................................guucucacccaacagGuccggg...........................................     1
```

Gi1_4064547_x3.................uccgCcguaguauaacgg... 1
Gi5_8715332_x3...ucucacccaacagGuccg... 1
Gi1_4087317_x3.................uccggcguaguauaacggUuagaau.. 1
Gi1_4085415_x3.................uccggcguaguauaacggcuUg... 1
Gi1_4085742_x3..guucUauucccggcgccggagc...................... 1
G1a_5822464_x3.................Gccggcguaguauaacggc.. 1
G1a_5854144_x3...gguucgauucccggcgccggCgc...................... 1
Gi1_4078194_x3.................uccggcAuaguauaacgg... 1
Gi3_27864914_x3...uucgaCucccggcgccgg......................... 1
Gi3_27815537_x3..uucucacccaacagaucAg... 1
Gi3_27969116_x3..Aguucucacccaacagaucc.. 1
Gi2_10779472_x3...gguucgauucccggcgcAg.......................... 1
Gi3_27954134_x3..guucgauucccUgcgccggagc...................... 1
Gi3_27837659_x3..gguucucacccaacagauAcgg.. 1
Gi5_8680364_x3...gguucgauucUcggcgccg.......................... 1
Gi2_10798258_x3...gguucgauucccggcgGcgg......................... 1
Gi2_10780153_x3..gguucucCcccaacagauccg... 1
Gi3_27887174_x3..uucucacccaacagCuccg... 1
Gi3_27964262_x3...gguucgauucccggcgccgUa........................ 1
Gi1_4085181_x3.................uccggcguaguauGacggcuagaau.. 1
Gi5_8680913_x3..guucgauucccggUgccggagc...................... 1
Gi2_10748833_x3.................uccggcguaguauaacggcuCgaa... 1
Gi1_4074489_x3.................uccgAcguaguauaacggcuag... 1
Gi5_8706638_x3.................uccggcUuaguauaacgg... 1
Gi3_27822218_x3.................uccggcguaguauaacggcuagaG... 1
Gi1_4057173_x3.................uccggcguaguauaCcggcuaga.. 1
Gi5_8691923_x3.................uccggcguagGauaacggcuag... 1
Gi1_4056513_x3..uucucacccaacagCuccgg.. 1
Gi5_8714339_x3..ucUauucccggcgccggagc...................... 1
G1a_5872276_x3...gguucgaCucccggcgccggagc...................... 1
Gi3_27939362_x3.................uccggcguaCuauaacggcua.. 1
Gi1_4063098_x3...gguucgauuccGggcgccgg......................... 1
Gi3_27836450_x3..guucgauucccggcgccgUag....................... 1
Gi3_27881060_x3..................Ucggcguaguauaacggcuaga.. 1
G1a_5829127_x3...ucucacccaacagGuccg... 1
Gi5_8654009_x3..................ccggcguaguauaGcggcua.. 1
Gi3_27960362_x3.................uccgCcguaguauaacgg... 1
Gi3_27944972_x3..ucUauucccggcgccggagc...................... 1
Gi5_8665886_x3..Nguucucacccaacagauccg... 1
Gi3_27859094_x3...guucucGcccaacagauccgg.. 1
Gi5_8658599_x3...cgauucccggcgccggagcCu.................... 1
Gi3_27859577_x3...uccggguucgauucccggcgccgAa........................ 1
Gi5_8685113_x3..guucgauucccggcgccggaAc...................... 1
Gi2_10730029_x3..gguucucacccaacagGuccg... 1
Gi5_8693288_x3..gauucccggcgccggGgc...................... 1
Gi3_28079716_x2...gguucgauucccggcgcGgg......................... 1
Gi1_4133185_x2....................gUcguaguauaacggcuagaau.. 1

```
Gi2_10980637_x2................................................................uccggguucgauucccggc..............................     0
Gi5_8822941_x2..................Acggcguaguauaacggcuag..........................................................................     1
Gi5_8826375_x2.....................................................................Nuucgauucccggcgccgg.........................     1
Gi1_4143699_x2.............................................................agauccggguucgauucc..................................     0
Gi3_28028530_x2...................................................cucacccaacagauccggguCcgauu....................................     1
Gi5_8723713_x2.........................................................................gUuucccggcgccggagc......................     1
Gi1_4125441_x2.....................................................................guucgauucccggcgGcgga........................     1
Gi5_8752453_x2..................ccUgcguaguauaacggcua...........................................................................     1
Gi1_4147217_x2.....................................................................guucgauucccggUgccggagc......................     1
Gi4_564144_x2 .....................................................................guucgauucccggcgccggagcA.....................     1
Gi5_8792971_x2..................ccggcguCguauaacggcuaga.........................................................................     1
Gi1_4152177_x2..............................................uggGucucacccaacagauccg.............................................     1
Gi5_8721245_x2.................................................uucCcacccaacagauccgg............................................     1
Gi3_28022676_x2......................................................................uucgauucccggcgccggagcA.....................     1
Gi2_10990929_x2...............aguccggcguaguauaacAg..............................................................................     1
Gi1_4129793_x2....................................................................gguucgCuucccggcgccggag.......................     1
Gi2_10924103_x2......................................................................uucgauucNggcgccggagc.......................     1
Gi2_10922261_x2.................uccggcgGaguauaacggcua...........................................................................     1
Gi2_10933207_x2..............................................ugguucucaccUaacagaucc..............................................     1
G1a_5894674_x2....................................................................gguucgauucccggcgUc...........................     1
Gi1_4135515_x2.................uccggcguaguauaacggcuC...........................................................................     1
Gi2_10922863_x2.....................................................................guucgauAcccggcgccg..........................     1
Gi1_4125299_x2.......................................................................ucgauucccggcgccggaU.......................     1
Gi5_8752639_x2....................................................................ggGucgauucccggcgccggagcguu...................     1
Gi5_8753319_x2..................cUggcguaguauaacggcuag..........................................................................     1
G1a_5954188_x2.....................................................................guucgGuucccggcgccgg.........................     1
Gi2_10874447_x2.................uccggcguagCauaacggcuagaau.......................................................................     1
Gi1_4147393_x2..............................................ugguucucUcccaacagauccg.............................................     1
Gi4_560646_x2 ....................................................................gguucgauucccUgcgccgg.........................     1
Gi3_28198142_x2......................................................................uucgauucccggGgccgga........................     1
Gi5_8812953_x2.........................................................................gaCucccggcgccggagc......................     1
Gi3_28044874_x2.................uccggcCuaguauaacggc.............................................................................     1
G1a_5959402_x2....................................................................ggCucgauucccggcgccg..........................     1
Gi2_10890015_x2.....................................................................guGcgauucccggcgccggag.......................     1
Gi3_28133844_x2.....................................................................gNucgauucccggcgccggagc......................     1
Gi1_4106855_x2....................................................................gguucgauucccggcgAcg..........................     1
Gi5_8747139_x2.................................................uucucacccaacGgauccggguuc........................................     1
Gi5_8787061_x2....................ggcguaguauCacggcuaga.........................................................................     1
Gi5_8779869_x2....................................................................gguucgGuucccggcgcc...........................     1
Gi2_10867705_x2.................uccggcguaguauUacggcua...........................................................................     1
Gi1_4131383_x2.....................................................................guucgauucccggcgAcggagc......................     1
Gi3_28084690_x2................................................guucucacNcaacagauccgg............................................     1
Gi2_10886109_x2.................................................uucucacccUacagauccg.............................................     1
Gi1_4100149_x2...................................................cAcacccaacagauccgg............................................     1
Gi3_28253028_x2...................................................................gggCucgauucccggcgccggag.......................     1
Gi1_4094725_x2................................................guucucacccaacagauGcg.............................................     1
Gi3_28254678_x2........................................................................Ugauucccggcgccggagc......................     1
```

```
Gi3_28098530_x2.......................................................................cgauucccggcgccggGg.......................     1
Gi2_10835159_x2...............................................guucucacNcaacagauccgg............................................     1
Gi3_28207296_x2...................................................................gguNcgauucccggcgccggag.......................     1
Gi2_10851117_x2.............................................ugguucucacccaacagaucAg.............................................     1
Gi2_10842717_x2..............................................gguucAcacccaacagauccg.............................................     1
Gi3_28230496_x2............................................Cugguucucacccaacagaucc..............................................     1
Gi5_8818071_x2.................uccggcguaguauaacggcuagaGugguu..................................................................     1
Gi1_4118663_x2...................................................................gguucgauucccggcAccgga........................     1
Gi3_28146898_x2.................uccggcguaCuauaacggc............................................................................     1
Gi1_4129347_x2.................................................ucCcacccaacagauccg.............................................     1
Gi5_8759113_x2.......................................................................cUauucccggcgccggagcguuuc.................     1
Gi3_28172804_x2...............................................guucucacUcaacagauccggg...........................................     1
Gi3_28134252_x2................................................uucucacccCacagauccg.............................................     1
Gi3_28134604_x2........................................................cGacagauccggguucga......................................     1
Gi1_4149101_x2.................uccCgcguaguauaacggcuag.........................................................................     1
Gi1_4100309_x2.................uccggcguagCauaacggcuagaa.......................................................................     1
Gi5_8741773_x2.....................................................................uucgauucccggcgccggagc......................     0
Gi3_28133342_x2......................................................................ucgauucccggcgccgCagc......................     1
Gi1_4102229_x2..................cUggcguaguauaacggc............................................................................     1
Gi3_28048188_x2...................................................................gguucCauucccggcgccgg.........................     1
Gi1_4106031_x2..................................................cucacccaNcagauccggguucga......................................     1
Gi3_28210084_x2...........................................uuggCucucacccaacagaucc...............................................     1
Gi1_4121287_x2............................................uugguucucacccaacagauccgg............................................     0
Gi5_8738045_x2......................................................................uUgauucccggcgccggagc......................     1
G1a_5941946_x2.................uGcggcguaguauaacgg.............................................................................     1
Gi3_28038906_x2.................................................uGucacccaacagauccgg............................................     1
Gi2_10850087_x2..................ccggcguaguauaacggcuag.........................................................................     0
Gi3_28096924_x2....................................................................guucgauucAcggcgccg..........................     1
Gi2_10904063_x2........................................................................gauucccggUgccggagc......................     1
Gi1_4104675_x2...................................................................gguucgauucccggcgccggCg.......................     1
Gi5_8806185_x2.................................................ucucaccUaacagauccgg............................................     1
Gi3_28034016_x2...................................................................gguucgUuucccggcgcc...........................     1
G1a_5978784_x2.................uccggcguaguauaacggcuaA.........................................................................     1
Gi3_28175422_x2.....................................................................uucgauucccggcgcUggagc......................     1
Gi3_28076800_x2....................................................cacccaacagauccggguCc........................................     1
Gi1_4152189_x2...................................................................gguucgauucccUgcgccg..........................     1
Gi3_28089238_x2.....................................................................uucgauucAcggcgccgga........................     1
Gi3_28065536_x2............................................................agaucUggguucgauuccc.................................     1
Gi3_28136160_x2...............................................guucucacccaacGgauccggg...........................................     1
Gi5_8742179_x2...................................................................gguucgauAcccggcgccggag.......................     1
Gi2_10885251_x2.............................................ugguucucacccaacagauccgg............................................     0
G1a_5904370_x2....................................................................gCucgauucccggcgccgg.........................     1
Gi3_27993528_x2................................................uucAcacccaacagauccg.............................................     1
G1a_5899828_x2...................................................................gguuUgauucccggcgccgg.........................     1
Gi3_28000050_x2...............................................guucucaUccaacagauccg.............................................     1
Gi5_8771865_x2.................uccggcguaguaGaacgg.............................................................................     1
Gi5_8789659_x2....................................................................guucgaAucccggcgccgga........................     1
```

```
Gi2_10918135_x2...................................................................gguucgauucccggcgUcgg.........................     1
Gi1_4132839_x2.................uccggAguaguauaacggcuagaau......................................................................     1
Gi3_28203820_x2.......................................................................cgauucccggcgcGggagc......................     1
Gi2_10981625_x2................................................uucucaccAaacagauccg.............................................     1
Gi3_28231734_x2.......................................................................cgauucccggcgGcggagc......................     1
Gi4_554144_x2 ..................................................cGcacccaacagauccggguucg.......................................     1
Gi5_8765217_x2....................................................................guucgauucccgCcgccg..........................     1
Gi3_28010828_x2.................uccggcguagAauaacggcuagaa.......................................................................     1
Gi5_8728071_x2.................uccNgcguaguauaacgg.............................................................................     1
G1a_5939658_x2...............................................guucucacccaacGgauccg.............................................     1
Gi2_10890847_x2....................................................................guucgauucccggcgcccCgag......................     1
G1a_5881486_x2.................uGcggcguaguauaacggcua..........................................................................     1
Gi3_28233852_x2..................................................................gUguucgauucccggcgccg..........................     1
Gi3_28174146_x2.................................................ucAcacccaacagauccg.............................................     1
Gi3_28231186_x2......................................................................ucgauAcccggcgccggagc......................     1
Gi1_4141445_x2...................................................................gguucgUuucccggcgccggagc......................     1
Gi3_28181902_x2...................................................................gguucgauucccggcgccUg.........................     1
Gi5_8720173_x2..............................................gguucucacccUacagauccg.............................................     1
Gi2_10977773_x2...............aguccggcguaguGuaacgg.............................................................................     1
Gi3_28140802_x2...................................................................gguucCauucccggcgccgga........................     1
Gi1_4135609_x2................................................uucuGacccaacagauccg.............................................     1
Gi5_8763973_x2...................................................................gguucgauucccggcgccggagcAu....................     1
Gi5_8812521_x2....................................................................guucgauucAcggcgccgga........................     1
Gi3_28114810_x2.............................................ugguucucacccaacagauAcg.............................................     1
Gi2_10906983_x2...................................................................gguucgauucccggcUcc...........................     1
Gi3_28001604_x2..............................................gguucucacccCacagauccgggu..........................................     1
Gi4_564836_x2 ...................................................................gguucgauucccggcgccggCgc......................     1
Gi5_8734655_x2...................................................................gguucgauucccggcgccgAag.......................     1
Gi3_28250398_x2.................uccggcguaguauaacCgcua..........................................................................     1
Gi3_28107384_x2......................................................................ucgauucccggcCccggagc......................     1
Gi2_10978851_x2.................uccggcguaguauUacggc............................................................................     1
Gi2_10897907_x2.................ucGggcguaguauaacggcuaga........................................................................     1
Gi3_28149672_x2..................................................................gggGucgauucccggcgccg..........................     1
Gi2_10849895_x2............................................................agauccggNuucgauucc..................................     1
Gi5_8743879_x2.................ucAggcguaguauaacggc............................................................................     1
Gi3_28017590_x2.............................................................gauccgggNucgauuccc.................................     1
Gi1_4142341_x2....................................................................guucgauucccggcgccAgag.......................     1
Gi3_28082106_x2..................ccggcguaguauaacggU............................................................................     1
Gi3_28130878_x2.................ucGggcguaguauaacggc............................................................................     1
Gi3_28194068_x2......................................................................ucgauucccggcgAcggagc......................     1
Gi1_4118443_x2.....................................................................uucgGuucccggcgccgg.........................     1
Gi2_10858823_x2....................................................................guucgauucUcggcgccg..........................     1
Gi2_10853099_x2......................................................................ucgauucccgCcgccggag.......................     1
Gi2_10963211_x2.................ucGggcguaguauaacggcua..........................................................................     1
Gi1_4153439_x2...................................................................gguucgauucccgAcgccgga........................     1
Gi4_562044_x2 ...................................................................ggCucgauucccggcgccgg.........................     1
Gi1_4128047_x2.................uAcggcguaguauaacgg.............................................................................     1
```

```
Gi1_4091035_x2.............................................ugguucucacccUacagauccgg............................................      1
Gi1_4114517_x2....................................................................gAucgauucccggcgccggagc......................      1
Gi2_10928843_x2.....................................................................uucgauucccggcgcAgg.........................      1
Gi3_28019476_x2...............aguccggcguaguauaacA..............................................................................      1
G1a_5934562_x2...................................................................gguucUauucccggcgcc...........................      1
Gi2_10952571_x2....................................................................guucgauucccggcgccggaUc......................      1
Gi2_10941671_x2...................cggcguaguauaGcggcuaga........................................................................      1
Gi5_8728773_x2....................................................................guucgauucccggcgcGgg.........................      1
Gi2_10869631_x2...................................................................gguucgauucccggGgccggag.......................      1
Gi2_10868905_x2...................................................................gguucgauucccggcgccggU........................      1
Gi1_4119237_x2.................uccggAguaguauaacggcua..........................................................................      1
Gi5_8819693_x2.................uccggcguaguGuaacggcu...........................................................................      1
Gi3_28035968_x2..................ccggcguaguaCaacggc............................................................................      1
Gi3_27984986_x2............................................................agauccggguucAauuccc.................................      1
Gi1_4103171_x2.................uccggcguaguauaacggAuaga........................................................................      1
Gi2_10852143_x2.......................................................................cgauucccggcgccggGgcguuu..................      1
Gi1_4116457_x2..............................................gguucucaccAaacagauccg.............................................      1
Gi3_28154066_x2..............................................gguucucaNccaacagauccgggu..........................................      1
Gi3_27997186_x2...........................................................cagauccggguucgauucUc.................................      1
Gi2_10933965_x2.............................................ugguucuUacccaacagauccg.............................................      1
G1a_5889500_x2.................uccggAguaguauaacggcuag.........................................................................      1
Gi2_10990005_x2...................................................................gguucgauuccGggcgcc...........................      1
Gi1_4101697_x2.................uccggcAuaguauaacggcuaga........................................................................      1
Gi5_8767991_x2................................................uucuGacccaacagauccg.............................................      1
G1a_5943254_x2.................................................ucucacccNacagauccg.............................................      1
Gi3_28213472_x2...........................................guugguucucacccaacagauccggg...........................................      0
Gi4_558114_x2 .................uccgUcguaguauaacggcuaga........................................................................      1
Gi3_28051586_x2......................................................................ucgauucccggcgccggaA.......................      1
G1a_5942324_x2.................Gccggcguaguauaacggcuagaa.......................................................................      1
Gi1_4158449_x2...................................................................gguucgauCcccggcgccgga........................      1
Gi2_10853421_x2.................................................................cggguucgauucccggcgccggagc......................      0
Gi3_28247670_x2...........................................................cagauccAgguucgauuccc.................................      1
Gi3_28249020_x2................................................uucucacccaacagauUcgg............................................      1
Gi1_4134717_x2....................................................................guucgauucccggUgccg..........................      1
Gi1_4106429_x2..................................................Aucacccaacagauccgg............................................      1
Gi1_4095373_x2......................................................................ucgauCcccggcgccggagc......................      1
Gi1_4137261_x2.................uccggcgCaguauaacggcuaga........................................................................      1
Gi5_8780779_x2.............................................ugguucucacccUacagauccg.............................................      1
Gi3_28236496_x2................................................uucucaccAaacagauccgg............................................      1
Gi1_4140037_x2.................uccggcguNguauaacggcuagaau......................................................................      1
Gi3_28128564_x2.....................................................................uucgauucccggcgccggag.......................      0
Gi1_4145965_x2..................................................cucacccaacagaucAgg............................................      1
Gi2_10980227_x2.................uccggcguaguauaacggcuagaU.......................................................................      1
Gi2_10857755_x2.................uccggcguaguaGaacggcuag.........................................................................      1
Gi3_28247674_x2.....................................................................uucgauucccggcgccgAa........................      1
Gi1_4092719_x2...................................................................gguucgauucccggcgccUg.........................      1
Gi5_8784113_x2............................................ugAuucucacccaacagauccg..............................................      1
```

Gi1_4150379_x2...ugAuucucacccaacagauccg... 1
Gi2_10882565_x2..gauucccggcgcUggagc...................... 1
Gi3_28246232_x2...ugAuucucacccaacagauc... 1
Gi2_10954911_x2.................Cccggcguaguauaacgg... 1
Gi2_10982499_x2.....................Ucguaguauaacggcuagaa... 1
Gi5_8748139_x2...Gagauccggguucgauucccggcgccg.......................... 1
G1a_5925080_x2...gguucgauucccAgcgccggagc...................... 1
G1a_5949928_x2.................uccggcguaguUuaacggcuag... 1
Gi1_4151559_x2.................uccggcguaguauaacggcuCga.. 1
Gi3_28229254_x2...ucGcacccaacagauccg... 1
Gi3_28234392_x2...gguucgauucccggcgccggaU....................... 1
Gi5_8827813_x2..................Gcggcguaguauaacggcuagaau.. 1
Gi1_4111627_x2...gguucgauucccggUgccg.......................... 1
Gi3_28108420_x2...cagauccgggCucgauuccc................................. 1
Gi3_28040844_x2..ucgauucccggcgccggaAc...................... 1
Gi2_10947365_x2..guucgauuAccggcgccggag....................... 1
Gi2_10883591_x2..ucgauucccggcgccUga........................ 1
Gi3_28216684_x2..gguucucacccaCcagauccgggu.. 1
Gi5_8762963_x2...gguucgauucccggcgcAgga........................ 1
Gi4_566220_x2 ....Uguucucacccaacagauccg... 1
Gi1_4128245_x2...uggAucucacccaacagauccg... 1
Gi3_27993486_x2...guucucacccaCcagauccgg.. 1
Gi1_4091359_x2..Cuucgauucccggcgccggagc...................... 1
Gi2_10893647_x2...gguucgaAucccggcgccgga........................ 1
Gi5_8823847_x2...acccaacaNauccggguucgauuccc................................. 1
G1a_5939924_x2.................uccggcguaguauGacggcuaga.. 1
G1a_5921894_x2.................uccggcguaguauaacggcuagGa... 1
Gi3_28018490_x2...guucucacccaacaAauccgg.. 1
Gi3_28102444_x2....................gUcguaguauaacggcuaga.. 1
Gi2_10846981_x2..ucgauucccggcAccggagc...................... 1
Gi5_8757575_x2...gguucgauucccUgcgccggag....................... 1
Gi3_28212126_x2..gauucccgAcgccggagc...................... 1
Gi1_4125009_x2...ucGcacccaacagauccgg.. 1
Gi2_10972823_x2..gguucucacccaaAgauccg.. 1
Gi2_10948143_x2..Cgguucgauucccggcgccggagc...................... 1
Gi1_4091203_x2..guucgauucccggcCccgga........................ 1
Gi5_8724307_x2.................uccggcguaguauaacggA.. 1
Gi2_10962109_x2.................uccggcguaguauaacggGua.. 1
Gi1_4148503_x2..Uuucgauucccggcgccggag....................... 1
Gi2_10992029_x2.................uccggcguaguaAaacggcuaga.. 1
Gi3_27983194_x2..uucucCcccaacagauccgg.. 1
Gi2_10874581_x2.................uccggcguaguaCaacggcuagaa... 1
Gi3_28228472_x2.................ucAggcguaguauaacggcuagaau.. 1
Gi5_8721603_x2..uGgauucccggcgccggag....................... 1
Gi1_4103481_x2...gguucgauucccggcgcU........................... 1
Gi5_8772157_x2...............aUuccggcguaguauaacggcuagaaugg.. 1
Gi2_10929055_x2..guucgauucccggcgAcggag....................... 1

```
Gi5_8731915_x2..............................................gguAcucacccaacagauccg.............................................      1
Gi1_4142901_x2...................................................................gguuUgauucccggcgccgga........................      1
G1a_5930724_x2...................................................................ggGucgauucccggcgccggagc......................      1
Gi3_28137472_x2..................cUggcguaguauaacggcuaga........................................................................      1
Gi1_4105631_x2................................................uucNcacccaacagauccg.............................................      1
Gi5_8721061_x2.................................................ucucacccaacGgauccg.............................................      1
Gi1_4155703_x2....................................................................guucgauuUccggcgccgga........................      1
Gi1_4115253_x2.................uccggAguaguauaacgg.............................................................................      1
Gi3_28086048_x2.......................................................................cgauucccggAgccggagc......................      1
Gi3_28162802_x2........................................................................gauucAcggcgccggagc......................      1
Gi3_28027976_x2.................uccggcguaguaGaacggc............................................................................      1
Gi3_28114184_x2...........................................................cagauccggguucgauuUcc.................................      1
Gi3_28218068_x2...................................................................gguucgaAucccggcgccgg.........................      1
Gi5_8822741_x2....................................................................guucgauucccggcgccgCa........................      1
Gi1_4118673_x2.................uccggcguaguauaUcggcuagaau......................................................................      1
Gi2_10988729_x2.......................................................................cgauuUccggcgccggagcguuuc.................      1
Gi2_10959557_x2.................uccggcguNguauaacggcu...........................................................................      1
Gi3_28236582_x2....................................................................guucgauCcccggcgccg..........................      1
Gi4_562006_x2 .................ucUggcguaguauaacggcuaga........................................................................      1
Gi2_10978487_x2........................................................................gaAucccggcgccggagc......................      1
Gi1_4156275_x2...................................................ucacccaacagaCccgggu..........................................      1
Gi1_4118337_x2....................................................caUccaacagauccggguucgauuccc.................................      1
Gi1_4110277_x2...................................................................gguucgauucccggcgccggC........................      1
Gi5_8731855_x2..........................................................Gcagauccggguucgauucccggcgc............................      1
Gi2_10978229_x2................................................uucucacccaacUgauccg.............................................      1
Gi3_28171068_x2...................................................................gguucgauucccggcgGcg..........................      1
Gi5_8729115_x2..............................................Cguucucacccaacagauccgg............................................      1
Gi5_8730009_x2...................................................................gguucgauAcccggcgccgg.........................      1
Gi2_10889355_x2.................uccggcguaguauaUcggc............................................................................      1
Gi3_28200538_x2.................uccggcguaguauaacggcuagUa.......................................................................      1
Gi4_564492_x2 .................uccggcguaguauaacggcuaAaau......................................................................      1
G1a_5920410_x2......................cguaguauaacggcuaga........................................................................      0
Gi5_8743365_x2.................................................ucCcacccaacagauccgg............................................      1
Gi3_28229644_x2.......................................................................cgauucccggcgccggagcCu....................      1
G1a_5918792_x2.............................................Ggguucucacccaacagauccg.............................................      1
Gi1_4100969_x2..............................................gguucuUacccaacagauccg.............................................      1
Gi2_10832883_x2....................................................................guucgauuGccggcgccgg.........................      1
Gi3_28257636_x2....................................................................guuNgauucccggcgccg..........................      1
Gi3_28009626_x2.............................................uAguucucacccaacagauccg.............................................      1
Gi2_10888181_x2...................................................................gguucgUuucccggcgccgga........................      1
G1a_5966336_x2...................................................................gguucgauucccggcgccAgagc......................      1
Gi5_8820989_x2.................uccggcguaguauaacCgcuag.........................................................................      1
Gi5_8827803_x2........................................................................gauucccAgcgccggagc......................      1
Gi3_28144720_x2..................................................cucacccaacagaucUgg............................................      1
Gi2_10976811_x2...................................................................gguucgauucccggcgccUga........................      1
Gi2_10833051_x2.................uAcggcguaguauaacggcuaga........................................................................      1
Gi4_560504_x2 ...................................................................gguCcgauucccggcgccggagc......................      1
```

```
Gi3_28226266_x2.................uccggcgAaguauaacggcuag.........................................................................     1
Gi3_28115858_x2...............................................................uUcggguucgauucccggcgccgga........................     1
Gi1_4147955_x2.................uccggcguaguauaGcggcu...........................................................................     1
Gi2_10944459_x2.....................................agaaugguNgguucucacc........................................................     1
Gi3_27985310_x2.................uccggcguaguauaacggcC...........................................................................     1
Gi3_28157448_x2...............................................guucucacccaacagaucAgg............................................     1
Gi1_4130011_x2...............................................guucucacccaacaAauccg.............................................     1
Gi2_10964813_x2...................................................ucacccaacagauccggguu.........................................     0
Gi3_28014692_x2....................................................cacccaacNgauccggguuc........................................     1
Gi1_4145445_x2.....................................................................uucgauucUcggcgccgg.........................     1
Gi4_556180_x2 ....................................................................Auucgauucccggcgccgg.........................     1
Gi5_8770111_x2....................................................................guucUauucccggcgccggagc......................     1
Gi2_10885075_x2..................ccggcguaguauaacggcCa..........................................................................     1
Gi1_4145883_x2...............................................uucucacGcaacagauccg..............................................     1
Gi1_4157677_x2...............................................guucucacccaacagauccU.............................................     1
Gi3_28097608_x2.....................Ucguaguauaacggcuagaa.......................................................................     1
Gi2_10890487_x2.................uccggAguaguauaacggcua..........................................................................     1
Gi2_10944301_x2.............................................Agguucucacccaacagauccg.............................................     1
Gi3_27980764_x2..................................................Uucacccaacagauccgg............................................     1
Gi2_10838125_x2..................ccggUguaguauaacggcua..........................................................................     1
Gi3_28240662_x2...................................................................gguNcgauucccggcgccgg.........................     1
Gi2_10919851_x2........................................................................gauucccggcgccggagcCu....................     1
Gi3_28180718_x2............................................................aCauccggguucgauucc..................................     1
Gi2_10868063_x2.................Accggcguaguauaacggcua..........................................................................     1
Gi2_10991033_x2...................................................................gguuGgauucccggcgcc...........................     1
Gi2_10847113_x2.............................................Ggguucucacccaacagaucc..............................................     1
Gi1_4123089_x2..................................................cucaGccaacagauccgg............................................     1
Gi5_8746507_x2....................................................................guucgauucccggcCccggagc......................     1
Gi3_28189706_x2...............................................guucucGcccaacagauccggg...........................................     1
Gi2_10948995_x2.................uccggcguUguauaacgg.............................................................................     1
Gi1_4147109_x2..................................................cucacccaacagauccgA............................................     1
Gi3_28229778_x2..................................................................gggGucgauucccggcgccggagc......................     1
Gi5_8777695_x2...................................................................gguucgauuccGggcgccg..........................     1
Gi3_28164600_x2................................................uucucCcccaacagauccg.............................................     1
Gi5_8829203_x2..................ccggcguGguauaacggcuag.........................................................................     1
Gi3_28119816_x2.....................................................................Cucgauucccggcgccggagc......................     1
Gi2_10848903_x2.................uGcggcguaguauaacggcu...........................................................................     1
Gi3_28001188_x2..............................................gguucuAacccaacagauccg.............................................     1
G1a_5909814_x2...................................................................gguucgauucccggcgccggC........................     1
Gi5_8757095_x2.......................................................................Ggauucccggcgccggagcguuuc.................     1
Gi5_8770651_x2....................................................................guucgauucccggcgccgAagc......................     1
Gi2_10974063_x2...............................................................uccggguuNgauucccggcgccgga........................     1
G1a_5953700_x2....................................................................guucgauucccggcgUcggagc......................     1
Gi3_28132424_x2.............................................................gauccggguucgGuuccc.................................     1
Gi5_8723877_x2..................ccggcguaguauaaGggcuag.........................................................................     1
Gi3_28056046_x2...............................................guucucacGcaacagauccg.............................................     1
Gi5_8764301_x2................................................uucucaccNaacagauccggguu.........................................     1
```

```
G1a_5965936_x2.................uccggcgCaguauaacggcua..........................................................................      1
Gi1_4150583_x2....................................................................guucgauucccgAcgccgga........................      1
Gi5_8745723_x2...................................................................gguucgauAcccggcgcc...........................      1
Gi5_8718575_x2...................................................................gguucgauucccggcgccggagG......................      1
Gi3_28027990_x2.......................................................................cgauucccggcgccAgag.......................      1
Gi3_28141006_x2..................................................................ggguCcgauucccggcgccg..........................      1
Gi3_28007004_x2.................................................uUucacccaacagauccgg............................................      1
Gi3_28146992_x2........................................................caacagauccggguucgauuccA.................................      1
Gi5_8737139_x2...................................................................gguucgauucccggcgccgCagc......................      1
G1a_5965278_x2...................................................................gguucgauucccggcgUcggagc......................      1
Gi1_4140307_x2....................................................................guucgauucccgAcgccg..........................      1
Gi3_28160534_x2.....................................................................uucgGuucccggcgccggagc......................      1
Gi5_8811289_x2.................uccggcguaguauaUcggc............................................................................      1
Gi1_4109757_x2.............................................ugguGcucacccaacagauccgg............................................      1
Gi1_4132611_x2......................................................................ucgGuucccggcgccggagc......................      1
Gi1_4157141_x2.................uccgCcguaguauaacggcuag.........................................................................      1
Gi3_27988624_x2..............................................gguucucacccaacGgauccgggu..........................................      1
Gi3_28174612_x2........................................................................gauucccggcgccggagcCu....................      1
Gi3_28238266_x2.................ucNggcguaguauaacgg.............................................................................      1
Gi3_28226426_x2.....................................................................uucgauucccggcgGcgga........................      1
Gi3_28109010_x2....................ggUguaguauaacggcuag.........................................................................      1
Gi5_8740929_x2..............................................gAuucucacccaacagauc...............................................      1
Gi5_8744349_x2.............................................ugguucucacccGacagauccgg............................................      1
Gi5_8738131_x2.................................................ucucacccaacagCuccg.............................................      1
Gi2_10831999_x2...............................................guucucacccaaGagauccgg............................................      1
Gi1_4093929_x2....................................................................guucUauucccggcgccg..........................      1
Gi2_10913593_x2............................................................agauccggguCcgauucc..................................      1
Gi5_8793103_x2.......................................................................cgauucccggcgccggagcC.....................      1
Gi5_8719451_x2...................................................................gguucgauucccggcgGcgga........................      1
Gi2_10984659_x2....................................................................guucgauucccggcgccggagG......................      1
G1a_5911344_x2.................uccggcguaguauaaAggcuag.........................................................................      1
Gi4_561578_x2 .................Gccggcguaguauaacggcuagaa.......................................................................      1
Gi1_4134087_x2.................uccggcguaguauCacggcuagaa.......................................................................      1
Gi2_10854593_x2..................................................................Cgguucgauucccggcgccg..........................      1
Gi3_28251546_x2.............................................ugguucucacccaacagauccA.............................................      1
Gi4_555552_x2 ....................................................................Uuucgauucccggcgccg..........................      1
Gi2_10864803_x2.................uccggcguCguauaacggcuag.........................................................................      1
Gi3_28030324_x2......................................................................ucgauucccggcgccggagcgA....................      1
Gi1_4115131_x2.............................................ugguucucCcccaacagauccg.............................................      1
Gi3_28204134_x2......................................................................ucgauucccggcgccggagU......................      1
Gi2_10836725_x2........................................................................gauucccgNcgccggagc......................      1
Gi2_10857619_x2.................uccggcguaguUuaacggcuag.........................................................................      1
Gi2_10940653_x2.....................................................................Cucgauucccggcgccggagc......................      1
Gi5_8731585_x2....................................................................guucgaAucccggcgccggagc......................      1
Gi3_28202388_x2.................uccggcguaguauCacggcuagaa.......................................................................      1
Gi5_8734339_x2..............................................gguucucacccCacagauccgg............................................      1
Gi3_27998018_x2.................uccggcUuaguauaacggc............................................................................      1
```

Gi3_28056430_x2..uucgGuucccggcgccgg.........................   1
Gi4_553470_x2 ....guucgauucccggcgccAg.........................   1
Gi2_10856589_x2..cgauucccggcgccggagcgCuuc.................   1
Gi3_28155778_x2...............Uguccggcguaguauaacggcuaga...   1
Gi4_565016_x2 ....cucacccaNcagauccggguucg.......................................   1
Gi2_10859405_x2...uucucacccaacagauccA...   1
Gi3_28013498_x2.................uccggcguaguauaacggGua...   1
Gi2_10960141_x2..uucgauucccggcAccgga........................   1
Gi5_8754285_x2..gguucgauucccggGgccg..........................   1
Gi2_10922609_x2...gauucccggcgccggagU......................   1
Gi1_4119445_x2..uucgauuccUggcgccgg.........................   1
Gi5_8744545_x2.................uccggcguaAuauaacggc...   1
Gi5_8813375_x2..uUucacccaacagauccg...   1
Gi1_4147083_x2....................ggcguaguNuaacggcuag..   1
Gi5_8732703_x2..gguucgauucccggcgcAgg.........................   1
Gi5_8789587_x2...uucucaGccaacagauccg...   1
Gi1_4147021_x2...guucgauucccggcgcAg..........................   1
Gi5_8785991_x2..................ccggcguaNuauaacggcua...   1
Gi3_28184974_x2.................uccggcguaguauaaAggcuagaau...   1
Gi1_4150551_x2..ugguucucaAccaacagauccgg..   1
Gi2_10869407_x2...guucCauucccggcgccggagc......................   1
Gi3_28086514_x2..cagauccggguucgauCccc.................................   1
Gi2_10851449_x2..Uuucucacccaacagauccgg..   1
Gi3_28057528_x2.................uccggAguaguauaacggcu..   1
Gi1_4114805_x2..Ccucacccaacagauccg...   1
Gi1_4123129_x2...cacccaacNgauccggguucgauuccc.................................   1
Gi2_10981085_x2...guucgauucccggcgccUg.........................   1
Gi5_8769191_x2..................cGggcguaguauaacggcuag..   1
Gi3_28224340_x2..guucucacccaacagauccgC..   1
Gi1_4134357_x2..guucucCcccaacagauccg...   1
Gi3_28178876_x2...cAcacccaacagauccgg..   1
Gi1_4099341_x2..gguucgauuccUggcgccgga........................   1
Gi3_28251356_x2..cgauCcccggcgccggagc......................   1
Gi3_27990064_x2.................uccggcguaguUuaacggcuagaa..   1
Gi1_4128663_x2.................uccggcguaguauaUcggcua...   1
Gi1_4151389_x2.................uccggcguagAauaacggc...   1
Gi3_28079074_x2...ucgauuccAggcgccgga........................   1
Gi1_4153745_x2...gguucucaUccaacagauccg...   1
Gi2_10835087_x2.................uccggcguaguauGacggcuag..   1
G1a_5900034_x2..Aguucgauucccggcgccgga........................   1
Gi2_10911591_x2...gauucccgUcgccggagc......................   1
Gi1_4109713_x2...uucucUcccaacagauccgg..   1
Gi2_10861221_x2...guucgauGcccggcgccggagc......................   1
Gi5_8760729_x2.................uccggcguaguauaacggUua...   1
Gi2_10918679_x2.................uccggcguUguauaacggcuagaa..   1
Gi1_4117015_x2..................cGggcguaguauaacggcu..   1
Gi2_10897681_x2..uuUgauucccggcgccggag.......................   1

```
Gi2_10975085_x2......................................................................ucgauucccggcgccggG........................     1
Gi3_28187862_x2..............................................gguucucacccaUcagauccg.............................................     1
Gi5_8817329_x2......................................................cccaacGgauccggguuc........................................     1
Gi5_8766929_x2...........................................................cagauccgggGucgauucccggcgccg..........................     1
Gi5_8743151_x2.................uccggcguagGauaacggc............................................................................     1
Gi3_28217236_x2.....................................................................uucgauucccggAgccgga........................     1
Gi2_10964545_x2..............................................gguucucUcccaacagauccg.............................................     1
Gi3_27994084_x2.....................................................................uucgCuucccggcgccgga........................     1
Gi3_28185408_x2.....................................................................uucgauucUcggcgccgg.........................     1
Gi1_4089331_x2.................uUcggcguaguauaacggcuagaa.......................................................................     1
Gi3_27980270_x2..............................................gguNcucacccaacagauccg.............................................     1
Gi2_10932017_x2..................................................cucacccaacGgauccgg............................................     1
Gi3_28044656_x2.................uccggcguaguauaGcggcu...........................................................................     1
Gi3_28123760_x2.................uccggcguaguauaUcggcuag.........................................................................     1
Gi2_10886137_x2...................................................................gAuucgauucccggcgccgg.........................     1
Gi5_8794179_x2.............................................ugguAcucacccaacagauccg.............................................     1
G1a_5897474_x2....................................................................guucgauucccggcgcUggagc......................     1
Gi3_28141704_x2....................................................................guucgauuccAggcgccggag.......................     1
Gi5_8829587_x2.............................................ugguucucacccaGcagauccgggu..........................................     1
Gi1_4093469_x2.................uccggcguaguauaacggUu...........................................................................     1
Gi1_4105501_x2.................uccggcguNguauaacggc............................................................................     1
Gi3_28183034_x2.................uccggcguaguauaUcggc............................................................................     1
Gi1_4136671_x2.....................gcguGguauaacggcuag.........................................................................     1
Gi1_4107931_x2................................................uucucaUccaacagauccg.............................................     1
Gi2_10861727_x2................................................uucucacccaCcagauccgg............................................     1
Gi1_4122639_x2..............................................Aguucucacccaacagauccgg............................................     1
G1a_5885892_x2.................uccAgcguaguauaacggcuag.........................................................................     1
Gi5_8817405_x2.................uccggcguaguauaacggcuagaaG......................................................................     1
Gi2_10930629_x2.................uccggcguagGauaacggc............................................................................     1
Gi1_4124243_x2..................ccggcguaNuauaacggc............................................................................     1
Gi3_28117386_x2...................................................................gguucgauucccggcUccg..........................     1
Gi5_8798379_x2.................................................ucucacccaacaUauccg.............................................     1
Gi2_10937331_x2.............................................ugguucucacccaacagauccC.............................................     1
Gi1_4140105_x2.......................................................................cgauucccggcgccggagcC.....................     1
Gi5_8785053_x2...................................................................gguuGgauucccggcgccgga........................     1
Gi1_4124623_x2..................................................cucacccaacagUuccgg............................................     1
G1a_5946596_x2...................................................................gguucgauucccggcgcAg..........................     1
Gi1_4142993_x2..................ccggcguGguauaacggcuaga........................................................................     1
Gi2_10945881_x2.....................................................................uucgaCucccggcgccgg.........................     1
Gi2_10988189_x2...............................................guucucacccaacaAauccg.............................................     1
Gi1_4103203_x2...........................................gGugguucucacccaacagauccgggu..........................................     1
Gi1_4155181_x2................................................uucucUcccaacagauccg.............................................     1
Gi3_27984222_x2....................................................................guucgauucccggcgccggaU.......................     1
Gi2_10886261_x2...............................................guucucacccCacagauccgg............................................     1
Gi5_8729459_x2....................................................................guucgauuccAggcgccggagc......................     1
Gi1_4125815_x2..............................................gguucucacccaaUagauccg.............................................     1
Gi2_10847523_x2....................................................................guucgauucccgCcgccggagc......................     1
```

```
Gi1_4099169_x2................................................uucucaccAaacagauccgg............................................      1
Gi3_28242240_x2...............................................guucuAacccaacagauccgg............................................      1
Gi1_4107127_x2...............................................................Gccggguucgauucccggcgccgga........................      1
Gi1_4122019_x2.....................................................................uucgauucccggcgccgga........................      0
Gi1_4135311_x2...................................................................gguucgauNcccggcgcc...........................      1
Gi5_8828365_x2.................................................uGucacccaacagauccgggu..........................................      1
Gi5_8820837_x2.....................Ucguaguauaacggcuaga........................................................................      1
Gi5_8739787_x2....................................................................guuGgauucccggcgccgg.........................      1
Gi3_28211802_x2..............................................................auccggguucgauucccggcg.............................      0
Gi1_4145897_x2.....................................................acccaacaNauccggguucgauucccg................................      1
Gi3_28184814_x2.................uccggcguagGauaacggcua..........................................................................      1
Gi2_10878373_x2...............................................guGcucacccaacagauccg.............................................      1
Gi2_10925909_x2...............agAccggcguaguauaacgg.............................................................................      1
Gi1_4104341_x2.................uccggcguaguauaaGggcuag.........................................................................      1
Gi3_28005272_x2....................................................................guucgauucccggcUccgg.........................      1
Gi3_28053262_x2.....................................................................Cucgauucccggcgccgga........................      1
Gi1_4156181_x2....................................................................guucgauucccggcgccUgagc......................      1
Gi5_8744671_x2....................................................................guucgauucccggcgAcgga........................      1
Gi1_4095231_x2...............................................guucucacccaacagauccC.............................................      1
Gi1_4113121_x2.................uccggcUuaguauaacggcuag.........................................................................      1
G1a_5908756_x2................................................uuUucacccaacagauccg.............................................      1
Gi5_8808045_x2..................................................cucacccaacagauAcgg............................................      1
Gi5_8802339_x2.................uccggcguaguauaacggUuagaa.......................................................................      1
Gi4_564000_x2 ....................................................................gCucgauucccggcgccgga........................      1
Gi3_28094166_x2..............................................gguucucacccaCcagauccgg............................................      1
Gi5_8723201_x2.................uccgAcguaguauaacggcua..........................................................................      1
Gi3_28059142_x2.......................................................................cgauucccAgcgccggag.......................      1
G1a_5936410_x2.................uccggcguaguCuaacggcuag.........................................................................      1
Gi5_8745705_x2...................................................................gguucgauuAccggcgccggag.......................      1
Gi1_4143113_x2.....................................................................uucgauucNcggcgccggagc......................      1
Gi2_10893279_x2............................................................agauccgggAucgauucc..................................      1
Gi3_28162628_x2...................................................................gguuGgauucccggcgcc...........................      1
Gi1_4153575_x2......................................................................ucgauucccggcgccggCgc......................      1
G1a_5898620_x2...................................................................gguucgauucccUgcgccggagc......................      1
Gi5_8719757_x2...................................................................gguuUgauucccggcgccggagcgu....................      1
Gi1_4121371_x2...................................................................gguucgauucccggcgccAgag.......................      1
Gi1_4116841_x2..............................................gguucucacccaacaAauccg.............................................      1
Gi5_8768605_x2.................uccggcguaguauaaGggcuagaa.......................................................................      1
Gi2_10851903_x2...................................................................gguAcgauucccggcgccgg.........................      1
Gi5_8774561_x2...................................................................gguucgauNcccggcgccggagcgu....................      1
Gi1_4154957_x2...............................................guucucacAcaacagauccgg............................................      1
Gi1_4158569_x2.................uccggcguaguauaaAggcua..........................................................................      1
Gi3_28125892_x2................................................uucucaUccaacagauccg.............................................      1
Gi5_8764685_x2....................................................................guucgauAcccggcgccgga........................      1
Gi1_4149059_x2.................uccgAcguaguauaacggcuagaau......................................................................      1
G1a_5956184_x2.................uccAgcguaguauaacgg.............................................................................      1
Gi2_10920215_x2.................uccggcguaguauaacggcC...........................................................................      1
```

```
Gi5_8829307_x2.................uccggcguaguauaacggcuagGa.......................................................................   1
Gi2_10959413_x2................................................uucucacGcaacagauccg.............................................   1
Gi5_8735337_x2.............................................ugguucucacUcaacagauccg.............................................   1
Gi1_4092761_x2.................uccggcguagCauaacggcuagaau......................................................................   1
Gi2_10905121_x2.......................................................................cgauucccgAcgccggagcguuu..................   1
Gi3_28150636_x2........................................................................gaGucccggcgccggagc......................   1
Gi2_10935317_x2....................................................................guucgauucccggcgccgAag.......................   1
G1a_5952986_x2....................................................................guucgauucccggcgccggagU......................   1
Gi5_8828401_x2.................uccggcguaguUuaacggcua..........................................................................   1
Gi3_28147466_x2......................................................................ucgauucccggcgcUggagc......................   1
Gi3_28091478_x2..................................................cucacccaacCgauccgg............................................   1
Gi1_4143385_x2.................................................ucGcacccaacagauccg.............................................   1
Gi5_8802831_x2...................................................................gguuAgauucccggcgccg..........................   1
Gi3_28073174_x2............................................uugguuGucacccaacagaucc..............................................   1
Gi5_8786395_x2.................................................ucucaccAaacagauccg.............................................   1
Gi1_4095171_x2...................................................................gguucgauuccUggcgccggagc......................   1
Gi5_8731359_x2...................................................................gguucgauucccggcgccC..........................   1
Gi2_10977695_x2....................................................................guucgauucccggcgccggaA.......................   1
Gi2_10848597_x2................................................uucucacUcaacagauccg.............................................   1
Gi3_28077110_x2......................................................................ucgauuAccggcgccgga........................   1
G1a_5954434_x2....................................................................gGucgauucccggcgccggag.......................   1
Gi5_8791119_x2................................................................Gcggguucgauucccggcgccgga........................   1
Gi2_10883617_x2...................................................................ggAucgauucccggcgccgg.........................   1
Gi3_28136020_x2................................................uucGcacccaacagauccg.............................................   1
Gi5_8750825_x2..................Ucggcguaguauaacggcua..........................................................................   1
Gi3_28236100_x2........................................................caacagauccggguucAauuccc.................................   1
Gi1_4107657_x2.............................................ugguucucacccUacagauccg.............................................   1
Gi1_4101425_x2...................................................................gguucgauucccggcgGcgg.........................   1
Gi1_4143387_x2...................................................................gguuGgauucccggcgccg..........................   1
Gi5_8730121_x2..................ccggcguaNuauaacggcuag.........................................................................   1
Gi3_28257730_x2...................................................................gguGcgauucccggcgccgga........................   1
Gi2_10926921_x2....................................................................guucgauucccggcgccCga........................   1
Gi3_28078132_x2........................................................................gauAcccggcgccggagc......................   1
Gi2_10884795_x2......................................................................ucgauucccggcgAcggagc......................   1
Gi3_28199080_x2...................................................................gguucgauucccggcgccUgag.......................   1
Gi1_4123331_x2................................................uucucacGcaacagauccgg............................................   1
Gi5_8766899_x2....................................................................gCucgauucccggcgccggag.......................   1
G1a_5917286_x2.................uccggcguaguauaacggGuagaa.......................................................................   1
Gi3_28133550_x2................................................uucucacccaacCgauccg.............................................   1
Gi1_4151535_x2................................................uucuUacccaacagauccgg............................................   1
Gi3_28046310_x2.................................................ucucacccaacCgauccg.............................................   1
Gi5_8789839_x2.................................................................cggguucgauucccggUgcc...........................   1
G1a_5927864_x2.................ucAggcguaguauaacggcuag.........................................................................   1
Gi2_10846363_x2.................uccggcguaguauaacggAuagaau......................................................................   1
G1a_5911236_x2.....................................................................uGcgauucccggcgccggagc......................   1
Gi4_556122_x2 ....................................................................guucgauuNccggcgccgg.........................   1
Gi2_10955977_x2.....................................................................uucgaCucccggcgccgga.......................   1
```

```
Gi5_8730235_x2......................................................................ucgauuccNggcgccggagc......................      1
Gi2_10926639_x2.................uccggcguaguGuaacggcu...........................................................................      1
Gi1_4128275_x2.................ucAggcguaguauaacggcua..........................................................................      1
Gi3_28229662_x2.................uccggcguaguauaacgU.............................................................................      1
Gi5_8788193_x2...................................................................gguuAgauucccggcgccgga........................      1
Gi5_8777755_x2.................uccggcguaguauaacAgcuagaa.......................................................................      1
Gi1_4148939_x2....................................................................guGcgauucccggcgccgga........................      1
Gi2_10835117_x2......................................................................ucgauCcccggcgccggagc......................      1
Gi3_28082596_x2.............................................ggAucucacccaacagauccgg.............................................      1
Gi3_28154844_x2.................................................................cggguucgauucccAgcgccgga........................      1
Gi3_28019352_x2..................ccggcguaguauGacggcuaga........................................................................      1
Gi3_27997574_x2........................................................................gauucccggcgccggagU......................      1
Gi5_8763795_x2...................................................................gguucgauucccgAcgcc...........................      1
Gi2_10973049_x2.................uccggcguaAuauaacggcuagaau......................................................................      1
G1a_5976028_x2.................uccggcguaguauaacgAcuag.........................................................................      1
Gi2_10986739_x2.................uccAgcguaguauaacggcua..........................................................................      1
Gi2_10842275_x2.................uccggcguaguauaaGggcuaga........................................................................      1
G1a_5915830_x2..............................................gCucucacccaacagauccg..............................................      1
Gi3_28204358_x2.......................................................................cgaCucccggcgccggagc......................      1
Gi2_10953299_x2.............................................ugguucucacccaacGgauccg.............................................      1
Gi3_28166900_x2.......................................................................cgauucAcggcgccggagc......................      1
Gi1_4153405_x2............................................................agauccgggCucgauuccc.................................      1
Gi2_10983297_x2...................cggcguaguauaacggcuag.........................................................................      0
Gi2_10865509_x2...................cggcguaguauaGcggcua..........................................................................      1
Gi3_28241054_x2.....................................................................uucgauucccggcgccAga........................      1
Gi1_4148421_x2.................uccggcguaguauaacgUc............................................................................      1
Gi5_8816703_x2...............................................guucucaGccaacagauccg.............................................      1
G1a_5977690_x2...................................................................gguucgauNcccggcgccgga........................      1
Gi3_28063086_x2.................ucGggcguaguauaacggcuagaau......................................................................      1
Gi5_8770649_x2.................uccggcgGaguauaacgg.............................................................................      1
Gi3_28060026_x2................................................uucucacccaacCgauccgg............................................      1
G1a_5976424_x2...................................................................gguucgCuucccggcgccg..........................      1
Gi1_4141471_x2....................................................................guucgauucccggcgccggagA......................      1
Gi3_28212010_x2...............Uguccggcguaguauaacggcuagaa.......................................................................      1
Gi1_4129275_x2...................................................................gguucgauucccCgcgccggagc......................      1
Gi1_4127809_x2....................................................................guucgauucUcggcgccgga........................      1
Gi2_10914271_x2.............................................uCguucucacccaacagaucc..............................................      1
Gi5_8803873_x2.....................gcguaguauaacggcuag.........................................................................      0
Gi2_10868131_x2.................uccggcguagGauaacggcua..........................................................................      1
G1a_5931608_x2.................uccggUguaguauaacggcuaga........................................................................      1
Gi3_28118232_x2...............................................guucucacAcaacagauccgg............................................      1
Gi3_28012268_x2....................ggcguaguauaacggcuagG........................................................................      1
Gi2_10949289_x2................................................uucucacccaaUagauccg.............................................      1
Gi3_28063662_x2..................................................cGcacccaacagauccggguucgauu....................................      1
Gi3_28224872_x2.....................................................................uucAauucccggcgccggagc......................      1
Gi2_10930853_x2................................................uuUucacccaacagauccggguucg.......................................      1
Gi4_554608_x2 ....................................................................guucgaCucccggcgccgg.........................      1
```

```
Gi1_4118677_x2....................................................................guucgauucccgUcgccg..........................     1
G1a_5898922_x2.................uccggcguaguauaGcgg.............................................................................     1
Gi2_10921797_x2.....................................................................uuUgauucccggcgccgga........................     1
Gi1_4154003_x2....................................................................guucgauAcccggcgccggagc......................     1
Gi5_8743487_x2.................uccggcgCaguauaacggcuagaa.......................................................................     1
Gi5_8800855_x2....................................................................guucgauAcccggcgccggagc......................     1
Gi1_4092961_x2.................uccggcguaguauaacggcuUga........................................................................     1
Gi1_4128985_x2..............................................................Cuccggguucgauucccggcgccgg.........................     1
Gi5_8789299_x2....................................................................guucCauucccggcgccg..........................     1
Gi3_28027372_x2.................................................ucucacccaacGgauccg.............................................     1
Gi4_553262_x2 ...................................................................gguucgauNcccggcgccggagc.....................     1
Gi1_4155657_x2.................uccggcguaguauaacCgcuag.........................................................................     1
Gi1_4109943_x2..............................................gguucucacGcaacagauccg.............................................     1
Gi2_10951235_x2......................................................................ucgauucccggcgccggagU......................     1
Gi2_10865455_x2.................uAcggcguaguauaacggcuagaa.......................................................................     1
Gi3_28166684_x2....................................................................guucgauucccgUcgccg..........................     1
Gi5_8822577_x2...................................................................gguucgauucAcggcgccg..........................     1
Gi1_4123745_x2.................uccggcguaguUuaacggcuag.........................................................................     1
Gi1_4109933_x2.................uccUgcguaguauaacggcuaga........................................................................     1
Gi5_8718193_x2.........................................................................auucccggcgccggagcA.....................     1
Gi3_28204080_x2..............................................Nguucucacccaacagauccg.............................................     1
Gi1_4150149_x2..............................................gguucucacccaacCgauccg.............................................     1
Gi1_4138259_x2...................................................................gguucgauucccUgcgccgga........................     1
Gi1_4105643_x2................................................uucucacccCacagauccgg............................................     1
Gi5_8808483_x2...................................................................gguucgaGucccggcgccg..........................     1
Gi5_8797229_x2...................................................................gguucgauucccggcAcc...........................     1
Gi5_8782575_x2...............................................gAucucacccaacagauccgg............................................     1
Gi5_8770777_x2.................ucUggcguaguauaacggcu...........................................................................     1
Gi1_4150117_x2.................uccggcguaguauaacggcuagGa.......................................................................     1
Gi3_28007158_x2..............................................gguucCcacccaacagauccgg............................................     1
Gi3_28236116_x2................................................uucucacccaacagauAcgg............................................     1
Gi2_10871971_x2....................................................................guucgauucccggcgccgCag.......................     1
Gi2_10833539_x2....................................................................guucgauucUcggcgccgga........................     1
Gi5_8755189_x2................................................Aucucacccaacagauccgg............................................     1
Gi2_10938311_x2................................................uAcucacccaacagauccg.............................................     1
Gi5_8799241_x2...............................................guucucacccaaAagauccgg............................................     1
Gi5_8798375_x2...............................................guucucacccaacagauccgA............................................     1
Gi1_4133053_x2...................................................................ggGucgauucccggcgccg..........................     1
Gi1_4147755_x2....................ggUguaguauaacggcua..........................................................................     1
Gi2_10952191_x2...................................................................gguucgauucccggcgccCg.........................     1
Gi3_28141668_x2..................ccggcguaguCuaacggcuag.........................................................................     1
Gi1_4093181_x2..................ccggcguaNuauaacggcua..........................................................................     1
Gi3_28008200_x2..................ccggcguaguauaGcggcuag.........................................................................     1
Gi3_28016028_x2.................uccggcguaguauaacggUuagaa.......................................................................     1
G1a_5897486_x2.................uccggcguNguauaacggcuag.........................................................................     1
Gi3_28024186_x2...................................................................gguucgauucccUgcgccgg.........................     1
Gi5_8790001_x2..............................................gguucucCcccaacagauccgg............................................     1
```

Gi4_559956_x2 ....guucgauucccggcgUcgg......................... 1
Gi3_28177218_x2..................ccggcguaguauCacggc.. 1
Gi3_28130614_x2..agauccggguucgCuuccc................................. 1
Gi1_4120257_x2...ggguucgauCcccggcgccgga......................... 1
Gi3_28239388_x2...gCucucacccaacagauccgg.. 1
Gi2_10968989_x2...ugAuucucacccaacagauccg... 1
Gi3_28254224_x2..guucgaGucccggcgccg.......................... 1
Gi1_4135867_x2...Cguucgauucccggcgccgga........................ 1
Gi5_8820439_x2..uucucacAcaacagauccggguu... 1
Gi5_8801251_x2..uucccggcgccggagcCu.................... 1
Gi3_28071682_x2...gguGcgauucccggcgccg.......................... 1
Gi5_8748173_x2...guucCcacccaacagauccgg.. 1
Gi3_28058190_x2..gguucucCcccaacagauccg... 1
Gi5_8756105_x2...Uauccggguucgauucccggcgccgga........................ 1
Gi1_4126001_x2...ugguucAcacccaacagauccgg.. 1
Gi5_8795605_x2...ugguucucacccaacCgauccg... 1
Gi1_4143197_x2..uucucaAccaacagauccgg.. 1
Gi3_28068438_x2...Cgguucucacccaacagauccg... 1
Gi3_28149690_x2.................uccgCcguaguauaacggcuagaau.. 1
Gi3_28053326_x2...gguucgauucccgCcgccgga........................ 1
Gi2_10896883_x2.................Nccggcguaguauaacggcuaga.. 1
Gi3_28214384_x2...Nuucucacccaacagauccg... 1
G1a_5954946_x2...gguucgGuucccggcgccgga........................ 1
Gi3_28161772_x2..gguucucacccaacagauccgggC.. 1
Gi1_4138577_x2..guucgauuccUggcgccgga........................ 1
Gi5_8805879_x2.................uccggcguaguauaaGgg... 1
Gi2_10831105_x2...uGcgauucccggcgccgga........................ 1
Gi3_28243286_x2..ucgauucccggcgcAgga........................ 1
Gi3_28146306_x2...cgauucccggcgccggGgc...................... 1
Gi2_10973233_x2..guucgauucccggGgccgg......................... 1
Gi3_28134518_x2.................uccggcguaguauaacggAuagaa... 1
G1a_5936588_x2.................uccggcguaguauaacggcCag... 1
Gi3_28028268_x2...uuGgauucccggcgccgg......................... 1
Gi3_28187008_x2..................Gcggcguaguauaacggcuaga.. 1
Gi3_27996894_x2...guucucacccaacagaCccgg.. 1
Gi1_4095731_x2..gguucucacccaacagUuccg... 1
Gi1_4148309_x2..caGcagauccggguucgauu.................................... 1
Gi2_10935313_x2.................uccggcguaguauaacggUuagaa... 1
Gi1_4142627_x2...gguucgauucccAgcgccgga........................ 1
Gi2_10930911_x2.................uccggcguaguauaacggcG... 1
Gi2_10982175_x2...Cucgauucccggcgccgg......................... 1
Gi3_28175786_x2..Ggauccggguucgauuccc................................. 1
Gi1_4129393_x2..auUcggguucgauucccggcgccgga........................ 1
Gi2_10840491_x2..guucgauucccggAgccggag....................... 1
Gi5_8774761_x2.................uccggcguaguaAaacgg... 1
Gi3_28055514_x2....................Agcguaguauaacggcuagaau.. 1
Gi5_8760047_x2..................Acggcguaguauaacggcua.. 1

```
G1a_5941006_x2...............................................guuUucacccaacagauccg.............................................      1
Gi5_8797827_x2............................................uCgguucucacccaacagauccg.............................................      1
Gi1_4097929_x2....................................................................guucgauucccggAgccgga........................      1
Gi2_10872691_x2.................uccggcguaguauaCcggcuagaa.......................................................................      1
Gi5_8747733_x2.................Accggcguaguauaacggc............................................................................      1
Gi2_10844215_x2.................uccggcguagCauaacgg.............................................................................      1
Gi2_10969409_x2....................................................................guuGgauucccggcgccgg.........................      1
Gi2_10869777_x2....................................................................guucgauucGcggcgccggagc......................      1
Gi1_4122539_x2.................Accggcguaguauaacggc............................................................................      1
Gi3_28094734_x2...................................................ucacccaaNagauccggguuc........................................      1
Gi5_8760681_x2.................uccggcguaguauaacgCcuagaau......................................................................      1
Gi3_28038396_x2......................................................................ucgauucccggcgcGggagc......................      1
Gi1_4153631_x2................................................uucucacccaUcagauccg.............................................      1
Gi1_4105013_x2.................uccggcguaguGuaacggc............................................................................      1
G1a_5974960_x2...................................................................ggCucgauucccggcgccggag.......................      1
Gi2_10851779_x2.............................................ugguucucacccCacagaucc..............................................      1
Gi3_28026106_x2............................................................Cgauccggguucgauuccc.................................      1
Gi2_10967301_x2..............................................gguucucacccaacagauccC.............................................      1
Gi3_27995408_x2.................ucGggcguaguauaacggcuagaa.......................................................................      1
Gi2_10929383_x2.................uccggcguaguauUacggcuag.........................................................................      1
Gi2_10981691_x2.................uccggcguaguaGaacggcuaga........................................................................      1
Gi2_10912907_x2...................................................................gguNcgauucccggcgcc...........................      1
Gi1_4156505_x2.................uccggcguaguauaacggcuagaG.......................................................................      1
Gi3_28149006_x2..................................................Aucacccaacagauccgg............................................      1
Gi5_8741619_x2.................................................uGucacccaacagauccgg............................................      1
Gi5_8742463_x2..................ccggcguaguauaacggcCa..........................................................................      1
Gi4_564356_x2 ...............................................guucucacccaacagauccgg............................................      0
Gi4_559706_x2 .................uccggcguaguauaacggcCag.........................................................................      1
G1a_5935670_x2.............................................ugguucucacccaacagaCccg.............................................      1
Gi2_10947009_x2...............................................guucucaUccaacagauccg.............................................      1
Gi2_10948229_x2.........................................................................auucccggcgccggagcgA....................      1
Gi1_4145737_x2.................................................................cggguucgauucccggcgccgg.........................      0
Gi3_28006938_x2.................................................................cAgguucgauucccggcgccgga........................      1
Gi2_10831301_x2....................................................................guucgauucAcggcgccgga........................      1
Gi3_28135542_x2....................................................................guucgauucGcggcgccgg.........................      1
Gi3_28146416_x2...................................................................gguucgauucccggcgccggagcAu....................      1
Gi2_10985957_x2...............................................guucuAacccaacagauccg.............................................      1
Gi1_4112609_x2...................................................................gguucgauucccggcgUcggagc......................      1
Gi2_10830341_x2...................................................................ggGucgauucccggcgccgg.........................      1
Gi1_4100751_x2...................................................................gguucgauucccggcgccgAa........................      1
Gi1_4139401_x2...............aCuccggcguaguauaacggcuag.........................................................................      1
Gi5_8798343_x2...................................................................gguucgauuUccggcgccgga........................      1
Gi2_10922353_x2.......................................................................cgauucccggcgccggag.......................      0
Gi1_4105975_x2.................uccggcguaguaCaacggcua..........................................................................      1
Gi1_4156999_x2................................................uucucacccaacagUuccgg............................................      1
Gi3_28083252_x2....................................................................guucgauucAcggcgccggag.......................      1
Gi2_10922159_x2..............................................gguucucaUccaacagauccg.............................................      1
```

```
Gi3_28026004_x2...............................................guucucacccaacagauccU.............................................     1
Gi4_560328_x2 ..................................................cuUacccaacagauccggguucg.......................................     1
Gi2_10830033_x2.................uccggcguaguCuaacgg.............................................................................     1
Gi3_28023594_x2...................................................................Uacccaacagauccggguuc............................................     1
Gi5_8720847_x2..............................................ggGucucacccaacagauccgg............................................     1
Gi5_8799665_x2...............aguccggcguaguGuaacggcuagaaugg....................................................................     1
Gi1_4092259_x2.................uccAgcguaguauaacggcu...........................................................................     1
Gi5_8809121_x2.................uccggcgAaguauaacggcuaga........................................................................     1
Gi1_4138575_x2....................................................................guucgauucAcggcgccgga........................     1
Gi2_10965115_x2...............................................guucucacccaaUagauccg.............................................     1
Gi3_28149540_x2................................................uucucacccUacagauccggguuc........................................     1
Gi3_28210346_x2..................................................................gguucgauucccggGgccgg.........................     1
G1a_5943884_x2................................................uucucacAcaacagauccg.............................................     1
Gi3_28164796_x2..................................................................gguucgauuccGggcgcc...........................     1
Gi2_10830241_x2..................................................................gguucgauucccggcUccggag.......................     1
G1a_5966242_x2.....................................................................uucgaCucccggcgccggagc......................     1
Gi1_4123151_x2.................uccggcguaguUuaacggc............................................................................     1
Gi1_4107727_x2..............................................gguucucacccaacagauccC.............................................     1
Gi5_8803327_x2............................................................agaucUggguucgauuccc.................................     1
Gi2_10980179_x2..............................................gguucucacccaacCgauccg.............................................     1
Gi3_28018856_x2....................................................................guucgaAucccggcgccgg.........................     1
Gi1_4101767_x2.................uccggAguaguauaacggc............................................................................     1
Gi1_4152773_x2..............................................gguucucacccaacagaAccg.............................................     1
Gi2_10959617_x2........................................................................gauucccggcgccggUgc......................     1
Gi1_4154685_x2................................................uucucacccaaUagauccgg............................................     1
Gi3_28062282_x2.................................................ucucGcccaacagauccgg............................................     1
Gi5_8757139_x2.............................................ugguucuUacccaacagauccgg............................................     1
Gi4_558418_x2 ...................................................................gguucgauucccggAgccgg.........................     1
Gi5_8745417_x2...................................................................gguucgauucccgAcgccg..........................     1
Gi3_28130308_x2.......................................................................cCauucccggcgccggagc......................     1
Gi3_28073120_x2...............................................................uccggguucgauucccgAcgccgga........................     1
Gi5_8758435_x2................................................uucucaUccaacagauccgg............................................     1
Gi5_8808217_x2...................................................................gguucgauucccggcgAcggag.......................     1
Gi3_28127092_x2.................uccggcguaguauaacggcA...........................................................................     1
Gi3_28040690_x2................................................uucucacccaaUagauccg.............................................     1
Gi1_4151121_x2...................................................ucacccaaNagauccgggu..........................................     1
Gi3_28174226_x2........................................................................gauucccggcgccgAagc......................     1
Gi2_10927783_x2.............................................ugguucucacccaacagCuccg.............................................     1
Gi5_8740013_x2.................uAcggcguaguauaacggcuaga........................................................................     1
Gi3_28174380_x2......................................................................ucgauucccggcAccgga........................     1
G1a_5977502_x2.................ucUggcguaguauaacggcua..........................................................................     1
Gi1_4127477_x2.....................................................................uucgauucccAgcgccgg.........................     1
Gi3_28048824_x2............................................uugguucucacccaacCgaucc..............................................     1
Gi3_28150320_x2..................................................................ggguucgauucccggcgccggaA.......................     1
Gi3_28164826_x2....................................................................guucgauucGcggcgccgga........................     1
Gi1_4142987_x2.................................................ucucacccaCcagauccgg............................................     1
Gi3_28151816_x2...............................................guucucacccaacagauAcggg...........................................     1
```

Gi2_10973467_x2..caacagauccggguucgauuccc................................. 0
Gi1_4110413_x2.................uccggcguCguauaacggcuaga.. 1
Gi5_8726267_x2...................cggcguaguauaacggcuagC.. 1
Gi2_10982563_x2...ugguuUucacccaacagauccg... 1
Gi3_28038154_x2..caacagauccggUuucga...................................... 1
Gi1_4149379_x2...gguucgauucccggcAccg.......................... 1
Gi3_28021150_x2..gNucgauucccggcgccgga........................ 1
Gi1_4129407_x2...ugguucucacccaacagaucGgg.. 1
Gi1_4143625_x2...ugguucucacccaaAagauccgg.. 1
Gi3_28173668_x2...uucgauucccggcgUcgga........................ 1
Gi1_4106033_x2...gguucgauuccAggcgccgga........................ 1
Gi3_28067814_x2...gguucgauucccggcgGcggag....................... 1
G1a_5882038_x2..Auucgauucccggcgccggagc...................... 1
G1a_5900774_x2...ucGcacccaacagauccg... 1
Gi5_8807219_x2..uucucacGcaacagauccg... 1
Gi3_28118842_x2..gguucucacccaaAagauccg... 1
Gi1_4124701_x2...ucCcacccaacagauccgg.. 1
Gi1_4148397_x2..cucacccaacagCuccgg.. 1
Gi3_27989022_x2.................uccggcguaguauaacggcuagUau.. 1
Gi5_8804723_x2..uucucacccaGcagauccgg.. 1
Gi1_4156325_x2..................ccggcguaguauCacggcua.. 1
G1a_5889684_x2.................Accggcguaguauaacggcuaga.. 1
Gi3_28034640_x2...uggCucucacccaacagauc... 1
Gi1_4119473_x2..gguuUucacccaacagauccg... 1
Gi2_10868073_x2..guucgauuccAggcgccggag....................... 1
Gi5_8775383_x2..................ccggcguaguGuaacggcuag... 1
Gi5_8759301_x2..guucgauucccUgcgccg.......................... 1
Gi5_8746535_x2...guucucaUccaacagauccgg.. 1
Gi1_4129075_x2..gguucCcacccaacagauccg... 1
G1a_5919070_x2.................uccggcguaguGuaacggcuag... 1
Gi2_10872295_x2...gCuucgauucccggcgccgg......................... 1
Gi5_8799585_x2..uuUguucucacccaacagauccg... 1
Gi5_8818769_x2...ucucacccaacagaucUgg.. 1
Gi2_10886403_x2..guucgauAcccggcgccgga........................ 1
Gi3_28084158_x2..Cgguucgauucccggcgccggag....................... 1
Gi3_28233518_x2...ucucacccaacagCuccg... 1
G1a_5907016_x2.................ucUggcguaguauaacgg... 1
Gi2_10914825_x2..uUgauucccggcgccggag....................... 1
Gi3_28140736_x2..ucgaGucccggcgccggag....................... 1
Gi3_28108166_x2..ucgauucccggcgccggagcU..................... 1
Gi5_8738921_x2..guucgauucccggcgAcggagc...................... 1
G1a_5953910_x2.................Cccggcguaguauaacggcuag... 1
Gi2_10858231_x2...uccgAguucgauucccggcgccgga........................ 1
Gi1_4126973_x2..uucucacccaacagauGcgg.. 1
Gi2_10991491_x2...gguuAgauucccggcgccg.......................... 1
Gi2_10873443_x2...gguucgUuucccggcgccg.......................... 1
Gi5_8765215_x2..guuNgauucccggcgccg.......................... 1

```
Gi3_28187554_x2..................ccggcguaguauaaacAgc...........................................................................     1
Gi3_28145154_x2.................................................................cggguucgGuucccggcgccgga........................     1
Gi3_28000816_x2....................................................................guucgauucccggcgccgUa........................     1
Gi2_10950493_x2...................................................................gguucgauucccggGgccgg.........................     1
Gi2_10904223_x2.................uccggcgCaguauaaacggcuag........................................................................     1
Gi5_8769155_x2..............................................gguucucaNccaacagauc...............................................     1
Gi2_10856813_x2.................uccggcgGaguauaacgg.............................................................................     1
Gi1_4133331_x2.................uccggcguaguauaaacggcAaga.......................................................................     1
Gi3_28241752_x2...........................................................cagauccgAguucgauuccc.................................     1
Gi1_4089595_x2.................Accggcguaguauaaacggcuag........................................................................     1
Gi5_8730799_x2....................................................................guucgauucGcggcgccg..........................     1
Gi5_8826953_x2......................................................................uGgauucccggcgccggagc......................     1
Gi2_10940445_x2....................................................................guuNgauucccggcgccgg.........................     1
Gi5_8782019_x2...................................................................gguucAauucccggcgccggag.......................     1
Gi5_8811253_x2.......................................................................cgUuucccggcgccggagcguuuc.................     1
Gi4_566174_x2 .................uccggcguagCauaacggcuaga........................................................................     1
G1a_5955874_x2...................................................................gguucgauucccggcgccggCg.......................     1
G1a_5889316_x2....................................................................guucgaCucccggcgccgg.........................     1
Gi4_557714_x2 ....................................................................guucgauCcccggcgccgg.........................     1
Gi2_10858965_x2.................uccggcguaguauGacgg.............................................................................     1
Gi3_28113126_x2....................ggUguaguauaacggcuaga........................................................................     1
Gi1_4120567_x2....................................................................guucgauucccggcgcAgga........................     1
Gi5_8829199_x2......................................................................Ccgauucccggcgccggag.......................     1
Gi1_4151585_x2.................uccggcguagGauaacggc............................................................................     1
Gi5_8811325_x2....................................................................guucgauuccGggcgccgg.........................     1
G1a_5966164_x2.................uccggUguaguauaaacggcuag........................................................................     1
Gi2_10954433_x2.................uccggcguGguauaacggcu...........................................................................     1
Gi5_8795541_x2.................Nccggcguaguauaaacggcuag........................................................................     1
Gi1_4153047_x2....................................................................guAcgauucccggcgccggagc......................     1
G1a_5911486_x2...................................................................gguucgauucccggcgccggUgc......................     1
Gi5_8783157_x2.............................................ugguucCcacccaacagauccgg............................................     1
Gi3_28102078_x2.................uccggcguaguauaaacggcuCgaau.....................................................................     1
G1a_5896606_x2...............................................guucucacccaGcagauccg.............................................     1
Gi1_4106883_x2..................................................cucacccaCcagauccgg............................................     1
Gi5_8824933_x2...................................................................gguucgauCcccggcgccgg.........................     1
Gi1_4115003_x2.................Cccggcguaguauaaacggcu..........................................................................     1
Gi3_28001584_x2..................................................Gucacccaacagauccggguucgauu....................................     1
Gi3_28062436_x2.................uccggcguCguauaacggcu...........................................................................     1
Gi5_8761219_x2.............................................ugguucucacccaacGgauccgggu..........................................     1
Gi2_10838901_x2....................................................................guucgauucccgAcgccggag.......................     1
Gi3_28113092_x2.................uccggcguaguauaaacgAcu..........................................................................     1
Gi1_4139407_x2...................................................................gguucgauucccggcgUcgg.........................     1
Gi2_10925471_x2...................................................................gguucgauucccggcgcccCga.......................     1
Gi1_4149689_x2.................uccggcguUguauaacggcua..........................................................................     1
Gi3_28147456_x2.....................................................................uuUgauucccggcgccgg.........................     1
Gi2_10932327_x2...............................................guuUucacccaacagauccg.............................................     1
Gi3_28033412_x2.................uccggcguaguauaaacggcAagaau.....................................................................     1
```

```
Gi5_8808605_x2...................................................ucacAcaacagauccggguu.........................................     1
Gi3_28051960_x2...................................................................gguucgauucUcggcgcc...........................     1
Gi2_10943205_x2.................uccgAcguaguauaacggcuag.........................................................................     1
Gi3_28078084_x2...............................................guucucaccUaacagauccgg............................................     1
Gi2_10858249_x2.....................................................................uucgauucccggcgccggCg.......................     1
Gi2_10840641_x2.................uccggcgGaguauaacggcuagaa.......................................................................     1
Gi1_4102471_x2.............................................ugguucucacccGacagauccg.............................................     1
Gi3_28170350_x2.................uccggAguaguauaacggcuag.........................................................................     1
Gi3_28208094_x2................................................uucucacccaacagauccgggCuc........................................     1
Gi1_4151871_x2..................................................................ggguucgaNucccggcgccgga........................     1
Gi5_8726993_x2....................ggcguaguNuaacggcuaga........................................................................     1
Gi1_4149361_x2.................uccggcguagCauaacggcu...........................................................................     1
Gi1_4124833_x2.................................................ucucaAccaacagauccg.............................................     1
G1a_5885002_x2...................................................................ggAucgauucccggcgccggagc......................     1
Gi2_10897531_x2....................................................................guucgauAcccggcgccggag.......................     1
Gi3_28060866_x2.............................................ugguucucaccUaacagauccg.............................................     1
Gi5_8743865_x2........................................................................Aauucccggcgccggagc......................     1
Gi5_8774441_x2............................................uugguucucacccaCcagauccg.............................................     1
Gi3_28220846_x2................................................uucucacccaacagaucAggguuc........................................     1
Gi3_28255850_x2.................uccggcgGaguauaacggc............................................................................     1
Gi2_10928561_x2.................uccggcguaguauaacggAua..........................................................................     1
Gi1_4108265_x2...................................................................gCuucgauucccggcgccggagc......................     1
Gi1_4096007_x2...................................................Gcacccaacagauccggguucga......................................     1
Gi3_28169138_x2.....................................................................uAcgauucccggcgccggagc......................     1
Gi3_28057976_x2..............................................................auccUgguucgauucccggcgccgga........................     1
Gi3_28147408_x2...........................................................cagGuccggguucgauuccc.................................     1
Gi2_10981491_x2...................Gggcguaguauaacggcua..........................................................................     1
Gi5_8753995_x2....................................................................guucgauucccgCcgccgg.........................     1
Gi5_8801793_x2...................................................................ggCucgauucccggcgccggagcguu...................     1
Gi1_4155083_x2.................................................ucucacccaacCgauccg.............................................     1
Gi5_8769593_x2.................ucGggcguaguauaacggcuagaau......................................................................     1
G1a_5918104_x2.................uccggcguaAuauaacggcuaga........................................................................     1
Gi3_27993194_x2...................................................................gguucgaGucccggcgccggag.......................     1
Gi1_4125845_x2.................uccggcguaAuauaacggcua..........................................................................     1
Gi5_8780981_x2...................................................................gguucgauucccggcgGcgg.........................     1
Gi1_4152215_x2...............................................guucucacccaacagUuccg.............................................     1
Gi1_4117409_x2.............................................uUguucucacccaacagaucc..............................................     1
Gi5_8754887_x2.....................................................................uucgaCucccggcgccgg.........................     1
Gi3_28239600_x2................................................uucucacccaacagauccAg............................................     1
Gi3_28081378_x2.................................................ucucacccGacagauccg.............................................     1
Gi5_8745639_x2...................................................................gguucgauucccggcgGcggagc......................     1
Gi2_10962979_x2.............................................ugguucucacccaacagaucUg.............................................     1
Gi5_8747675_x2...................................................................gguucgauucAcggcgccgg.........................     1
Gi3_28106474_x2..............................................gguucuUacccaacagauccg.............................................     1
Gi3_28060622_x2..................Ucggcguaguauaacggc............................................................................     1
Gi3_28096670_x2..................................................cuUacccaacagauccggguucgauu....................................     1
Gi5_8732521_x2...............................................................uccggguucgGuucccggc..............................     1
```

```
Gi5_8719709_x2................................................uucAcacccaacagauccg.............................................   1
Gi2_10965185_x2......................................................................ucgGuucccggcgccggagc......................   1
Gi5_8729477_x2................................................uucucacccaacaAauccggguuc........................................   1
Gi1_4152497_x2..................................................cucaccUaacagauccgg............................................   1
Gi5_8748009_x2...................................................ucacccaacagGuccggguu.........................................   1
Gi2_10908611_x2................................................uucucacccaacagaucAg.............................................   1
Gi1_4137099_x2.................................................ucucGcccaacagauccgg............................................   1
Gi1_4101387_x2...................................................................gguucgauucccggcgccggaA.......................   1
Gi1_4145699_x2..................Gcggcguaguauaacggcua..........................................................................   1
Gi5_8744307_x2.................uccggcguaCuauaacggcua..........................................................................   1
Gi5_8781511_x2..............................................gguucucacccaacagaCc...............................................   1
Gi5_8814999_x2.................uAcggcguaguauaacggcuag.........................................................................   1
Gi3_28041284_x2.......................................................................cgauucccggcgccggaAc......................   1
Gi1_4094027_x2...................................................................gguucgauucccggcgccU..........................   1
Gi3_28051810_x2...................................................................gguucgauucccggcgccUga........................   1
Gi3_28177132_x2..................ccggcguaguauaacggcuGga........................................................................   1
Gi1_4153889_x2...............................................guucucacGcaacagauccg.............................................   1
Gi2_10844611_x2.................uccggcguaguauCacggcuag.........................................................................   1
Gi2_10905607_x2.............................................ugguGcucacccaacagauccg.............................................   1
Gi3_28201694_x2...............................................guucucacccaacagaucGg.............................................   1
Gi1_4145687_x2.................uccggcguagCauaacggc............................................................................   1
Gi3_28194368_x2...................................................................gguucgauucccggcgccggagcgC....................   1
Gi1_4112899_x2....................ggcguaguauaGcggcuag.........................................................................   1
Gi5_8754843_x2............................................................agUuccggguucgauuccc.................................   1
G1a_5936950_x2...................................................................Cguucgauucccggcgccgga........................   1
Gi1_4123847_x2........................................................Gaacagauccggguucgauucccggc..............................   1
Gi5_8823089_x2...............................................................uUcggguucgauucccggc..............................   1
Gi2_10973301_x2....................................................................guucgauucccggcgGcg..........................   1
Gi3_28077204_x2.................uccggcguaguaGaacggcuagaa.......................................................................   1
Gi3_28230940_x2......................................................................ucgauucccgCcgccggagc......................   1
Gi3_28012736_x2...................................................................ggNucgauucccggcgccg..........................   1
Gi3_28154320_x2........................................................caacagauccggguucgCuuccc.................................   1
Gi5_8746429_x2..................................................cucacccaacagauUcgg............................................   1
Gi3_28168954_x2............................................................agauccggguucgauCccc.................................   1
Gi5_8772669_x2.....................................................................uucgauucccggcgccggagcC.....................   1
Gi1_4107525_x2.....................................................................uCcgauucccggcgccgg.........................   1
Gi5_8750557_x2................................................uuUucacccaacagauccggguu.........................................   1
Gi3_28197924_x2.................................................ucucaccUaacagauccgg............................................   1
Gi3_28190458_x2....................................................................guucgauucccggcgcGgg.........................   1
Gi3_28177260_x2................................................uucuGacccaacagauccgg............................................   1
Gi2_10946935_x2.................uccggcgCaguauaacggcuagaau......................................................................   1
Gi5_8782065_x2.....................................................................uucgauucccggcgccggagcCu....................   1
Gi2_10904021_x2.................uccggcguaCuauaacggcuaga........................................................................   1
Gi3_28236300_x2..............................................................Guccggguucgauucccggcgccggagc......................   1
Gi5_8738783_x2.................uccggcguaguauaacggcuCgaau......................................................................   1
Gi3_28013354_x2.....................................................................uucgauucccAgcgccgga........................   1
Gi3_28112812_x2..................ccggUguaguauaacggcuag.........................................................................   1
```

```
Gi5_8785603_x2......................................................................ucgauucccgAcgccggag.......................      1
Gi3_28155424_x2.................uccggcguagGauaacggc............................................................................      1
Gi5_8721945_x2.................uccggcguaguauaacggcAagaa.......................................................................      1
G1a_5951118_x2...................................................................gguucgauucccggAgccggagc......................      1
Gi5_8728743_x2................................................uucucacccaacaAauccg.............................................      1
Gi5_8740795_x2...............................................guucucacccaaAagauccg.............................................      1
Gi2_10858659_x2...............................................guucucacccaacagauAcg.............................................      1
Gi3_27989860_x2......................................................cccaacagNuccggguucgauuccc.................................      1
Gi2_10859403_x2................................................uuUucacccaacagauccgg............................................      1
Gi3_28036522_x2..............................................ggGucucacccaacagaucc..............................................      1
Gi3_28016178_x2.......................................................ccaacagauccggguucgauucccggcgccgga........................      0
Gi1_4103711_x2.................uccggcguaguauaacCgcua..........................................................................      1
Gi5_8786747_x2................................................uucucacccaacagauccggAuu.........................................      1
Gi1_4136617_x2..................ccggcguaguauaacAgcuaga........................................................................      1
Gi2_10850057_x2.............................................ugguucucNcccaacagaucc..............................................      1
Gi1_4116277_x2.....................................................................uucgauucccggcgccggaU.......................      1
Gi2_10889625_x2.................uccggcguagCauaacggcu...........................................................................      1
Gi3_28167666_x2...........................................................cagauccggguucgauuccU.................................      1
Gi5_8746245_x2...............................................guucucaAccaacagauccg.............................................      1
Gi5_8740983_x2.................uccggAguaguauaacggc............................................................................      1
Gi1_4103413_x2..................ccggcguaNuauaacggcuaga........................................................................      1
Gi3_28104480_x2.....................................................................ucgauucccAgcgccggagc.......................      1
Gi3_28139806_x2.........................................................Gacagauccggguucgauucccggcgccgga........................      1
G1a_5905292_x2...................................................................gguucgauucccggcgccgC.........................      1
Gi1_4134587_x2....................................................................guucgauucccggcgccUga........................      1
G1a_5928082_x2.................uccAgcguaguauaacggcuaga........................................................................      1
Gi3_28194308_x2.................uccggAguaguauaacggcuagaau......................................................................      1
Gi5_8790799_x2....................................................................guucUauucccggcgccggag.......................      1
Gi5_8795831_x2..............................................gguucucacccaacGgauccgg............................................      1
G1a_5921074_x2...................................................................gguucgauucccggcgccggagA......................      1
Gi5_8820787_x2...................................................................gguucgauucccggcgUcggag.......................      1
Gi3_28175952_x2..................ccggcguaguaCaacggcuaga........................................................................      1
Gi1_4090145_x2.................uccgAcguaguauaacggcuaga........................................................................      1
Gi1_4118757_x2.....................................................................Cucgauucccggcgccggagc......................      1
Gi1_4100873_x2....................................................................guucgauucccggcAccggagc......................      1
Gi1_4129415_x2.............................................ugguucucacccaaUagauccgg............................................      1
Gi2_10964051_x2....................................................................guucgauucccggcgccggUg.......................      1
Gi5_8752755_x2..................Gcggcguaguauaacggcuag.........................................................................      1
G1a_5891236_x2.................uccggcguaguauaCcggcuag.........................................................................      1
Gi5_8772665_x2.............................................................agauccggguucgCuuccc................................      1
Gi5_8774521_x2..............................................gguucucaccGaacagauccg.............................................      1
Gi3_28148246_x2................................................uucucacccaUcagauccg.............................................      1
Gi2_10905079_x2................................................uucucacccaacagauGcg.............................................      1
Gi3_28023314_x2.................Nccggcguaguauaacggcuag.........................................................................      1
Gi3_28070692_x2...................cggcguaguauaacggcua..........................................................................      0
Gi3_28150738_x2....................................................................guuAgauucccggcgccggag.......................      1
Gi2_10895625_x2...............................................guucuUacccaacagauccg.............................................      1
```

G1a_5930474_x2...gguucgauucccggcgAc...........................   1
Gi1_4118461_x2...ggAucgauucccggcgccgga........................   1
Gi1_4114789_x2...gguuUgauucccggcgccggagc......................   1
Gi5_8774249_x2...ugguucucGcccaacagauccgggu..   1
Gi5_8738645_x2..uucucacccaacagGuccgg..   1
G1a_5894212_x2.................uccggcguagCauaacggcuag...   1
Gi5_8811637_x2.................uccggcguaguauaacggAuag...   1
Gi5_8723867_x2...gguucgauucccgCcgccggagc......................   1
Gi1_4153353_x2...ggGucgauucccggcgccgga........................   1
Gi1_4139763_x2.................uccggcguaguauaaAggcuag...   1
Gi2_10908019_x2...gguucgauuccAggcgccgg.........................   1
Gi2_10957595_x2.................uccggcAuaguauaacggcuagaau..   1
Gi5_8724119_x2...Nguucgauucccggcgccggagc......................   1
Gi2_10961799_x2.................uAcggcguaguauaacggc..   1
Gi3_28170812_x2..uucucacGcaacagauccgg..   1
Gi5_8784921_x2..guucgauucccggcgccggagcgC....................   1
Gi5_8826979_x2...................cggcguGguauaacggcuag...   1
Gi5_8762007_x2.................uccggcguaguaAaacggcuagaau..   1
Gi2_10834057_x2...ugguCcucacccaacagaucc..   1
Gi3_28239946_x2..ucgauucccggcgccggC........................   1
Gi3_28088832_x2.....................gcguaguauaacggcuGga..   1
Gi5_8775169_x2.................uccggcguaguauUacgg...   1
Gi5_8819389_x2..uugguucucacccaacagaucUg...   1
Gi5_8775529_x2..cucacccUacagauccgg..   1
Gi2_10845877_x2.................uccggcguaguauaaUggcuag...   1
Gi3_28112010_x2...ucCcacccaacagauccgg..   1
Gi1_4112635_x2..uucucacccaacagauccCg..   1
G1a_5929114_x2...gguucgauucccAgcgccggag.......................   1
Gi3_28067610_x2...................cggcguaguauaacggcuagaauggu...   0
Gi2_10901187_x2...guucucacccGacagauccg...   1
Gi3_28146482_x2..guucgauucccUgcgccg..........................   1
Gi3_28016184_x2...Nguucgauucccggcgccgga........................   1
Gi1_4132637_x2...guucucacccaaUagauccg...   1
Gi3_28128796_x2..cucacccaacagaCccgg..   1
Gi3_28078074_x2..guucgauuGccggcgccgg.........................   1
Gi3_28239860_x2...gauccggguucgaCuccc.................................   1
Gi1_4118543_x2..guucgauucccggcgccC..........................   1
Gi2_10831947_x2...Gucgauucccggcgccgga........................   1
Gi5_8787103_x2.................ucNggcguaguauaacgg...   1
Gi2_10847569_x2...guucucaccAaacagauccg...   1
Gi2_10850305_x2..guucgaGucccggcgccg..........................   1
Gi3_28096882_x2..guucgauucccggcgccUgag.......................   1
Gi3_28052776_x2..cucacccaaUagauccgg..   1
Gi1_4117525_x2...ucuUacccaacagauccgg..   1
G1a_5913426_x2...Gucgauucccggcgccggagc......................   1
Gi2_10949321_x2..uucucacccaacagauccAg..   1
Gi2_10947541_x2..Uccaacagauccggguuc..   1

```
Gi3_28241838_x2....................gUcguaguauaacggcuagaau......................................................................     1
Gi5_8812831_x2....................................................................guucgauucccgAcgccggagc......................     1
Gi3_28259230_x2...............................................guucucacUcaacagauccg.............................................     1
Gi5_8730133_x2.............................................uCguucucacccaacagauccgg............................................     1
Gi5_8819591_x2...................................................................gguucgauucccgAcgccggag.......................     1
G1a_5877838_x2................................................uCcucacccaacagauccg.............................................     1
Gi5_8741079_x2....................ggcguaguauaacggcuGga........................................................................     1
G1a_5955404_x2.....................................................................uucgGuucccggcgccggagc......................     1
Gi3_27985358_x2.................................................ucucacccNacagauccgg............................................     1
Gi5_8722591_x2......................................................................ucgauucccggcgccggCgc......................     1
Gi3_28075606_x2.............................................ugguucucaccAaacagauccg.............................................     1
Gi3_28169190_x2.....................................................................uuUgauucccggcgccgga........................     1
Gi5_8795627_x2........................................................caacagauccggguucgauucccggcgccggagc......................     0
Gi2_10980421_x2....................................................................guucgCuucccggcgccgga........................     1
Gi1_4144975_x2.................uccggcguaguauaacggcCagaa.......................................................................     1
Gi3_28150512_x2.................uccggcguaUuauaacggc............................................................................     1
Gi3_28160030_x2..........................................................Gcagauccggguucgauuccc.................................     1
Gi3_28117744_x2.............................................ugguucucaUccaacagauccg.............................................     1
Gi2_10918517_x2................................................uucucacccaGcagauccg.............................................     1
G1a_5938360_x2...................................................................gguNcgauucccggcgccg..........................     1
Gi5_8720941_x2.................uccggcguaguauaacggcuU..........................................................................     1
Gi3_28243794_x2.................uccggcguaguauaacCgcuag.........................................................................     1
Gi1_4091015_x2.................uccggcguaAuauaacgg.............................................................................     1
Gi5_8735137_x2....................................................................guucgauucccggcAccggagc......................     1
G1a_5964870_x2.................uccggcguaguauaacggcuagaaugguug.................................................................     0
Gi1_4151033_x2.................uccggcguaguauaacgUcuaga........................................................................     1
Gi3_28118820_x2.................uccggcguaguauUacggcuag.........................................................................     1
Gi2_10967907_x2.............................................ugguucuAacccaacagauccg.............................................     1
Gi2_10898493_x2...............................................guucucaccUaacagauccg.............................................     1
Gi3_27992666_x2....................................................................guucgauucccggcgccggagcAu....................     1
Gi5_8766853_x2...............................................guucucacccUacagauccgg............................................     1
Gi3_28002086_x2....................................................................guucgauucccggGgccggag.......................     1
Gi3_28062226_x2....................................................................guucgauucccggcgGcggag.......................     1
Gi3_28152912_x2...................................................................gguucgauucccCgcgccggag.......................     1
Gi1_4107505_x2...............................................guucAcacccaacagauccgg............................................     1
Gi3_27995030_x2........................................................................gauucccggcgccgUagc......................     1
Gi5_8718507_x2....................................................................guucgauucccggcgccggagA......................     1
Gi3_28200768_x2..............................................gguucucacccaacagGucc..............................................     1
Gi3_28201030_x2...................cggUguaguauaacggcua..........................................................................     1
Gi5_8807415_x2.............................................ugguucucaccAaacagauccg.............................................     1
Gi1_4115211_x2...................cUgcguaguauaacggcu...........................................................................     1
Gi5_8810763_x2...................................................................gguuGgauucccggcgccgg.........................     1
Gi2_10984877_x2......................................................cccaacGgauccggguuc........................................     1
Gi3_28123278_x2.................uccggcguaguauaacggcuagaGu......................................................................     1
G1a_5918610_x2.................uccgAcguaguauaacggcuag.........................................................................     1
Gi1_4118767_x2.................ucAggcguaguauaacggcuaga........................................................................     1
Gi5_8739661_x2.................uccggcguaguauaaGggcua..........................................................................     1
```

```
Gi3_28061330_x2..................................................ucucacccaacagauccC.............................................     1
Gi2_10924427_x2.....................................................................guucgCuucccggcgccg..........................     1
Gi1_4131255_x2......................................................................uucgauucccggcgccggCgc......................     1
Gi1_4143351_x2.................uccggcguaAuauaacggcuag..........................................................................     1
Gi3_28552813_x1.....................................................................guuNgauucccggcgccggag.......................     1
Gi3_28729090_x1.................................................uuGucacccaacagauccg.............................................     1
Gi5_9015306_x1.....................................................................guucgauGcccggcgccgg.........................     1
Gi5_8970024_x1...........................................................acagauccggguucgauucccggAgc............................     1
Gi3_28914915_x1..................................................................cggguucgauucccggcAccgga........................     1
Gi3_28965147_x1..............................................Agguucucacccaacagauc...............................................     1
Gi3_28883816_x1....................................................................gguucgauucccggNgccggagc......................     1
Gi3_28448279_x1.....................................................................guucCauucccggcgccggag.......................     1
G1a_6356277_x1.................uccggcguaguauaacggcuagaUugguugg.................................................................     1
Gi4_590903_x1 ....................................................................gguucgaCucccggcgccgg.........................     1
Gi3_28922194_x1................................................guucucUcccaacagauccg.............................................     1
Gi2_11176502_x1.................uccUgcguaguauaacggcuaga.........................................................................     1
Gi3_28607644_x1...................................................cucacccaacUgauccgg............................................     1
Gi3_28608323_x1..............................................................gGuccggguucgauucccggcgccgga........................     1
Gi3_28425771_x1.................uccggcguaguauaUcggcu............................................................................     1
Gi5_9071651_x1.................................................uucucaccAaacagauccggguuc........................................     1
Gi2_11042293_x1.................uccggcguaguauaaGggcua...........................................................................     1
Gi4_596980_x1 ...................cggcguaguauaacggcuaga.........................................................................     0
Gi5_9126598_x1.......................................................cccaGcagauccggguucgau.....................................     1
Gi5_9136247_x1.................uccggcguaguauaacAgcuagaaugguu...................................................................     1
Gi3_28676654_x1.......................................................cGcaacagauccggguucgauuccc.................................     1
Gi3_28370586_x1.................................................uucucacccaacagauAcggguuc........................................     1
Gi5_8997230_x1...............................................gguucuGacccaacagauccg.............................................     1
Gi5_8979070_x1.............................................uugguucucCcccaacagauccg.............................................     1
Gi5_9047390_x1.................uccggcguaguauaaUgg..............................................................................     1
Gi1_4274502_x1.........................................................caacUgauccggguucgauucccggc..............................     1
Gi1_4241720_x1....................................................................gguucgauucUcggcgccggagc......................     1
Gi5_8842286_x1....................................................................gguucgauuccAggcgccggagcguu...................     1
Gi2_11237360_x1....................................................................gguucgauuGccggcgccggag.......................     1
G1a_6126895_x1.....................................................................guucgauuNccggcgccgg.........................     1
Gi1_4387861_x1..............................................ugguucucacccaacaAauccgg............................................     1
Gi2_11390964_x1..........................................................aacagauccggguucgGuucccggcgccg..........................     1
Gi2_11289243_x1....................................................................gguucgauucccgCcgcc...........................     1
Gi2_11030898_x1..............................................ugguucucacccaacagauUcg.............................................     1
Gi3_28424382_x1.............................................uugguucucacccaacaga.................................................     0
Gi5_9082007_x1.................uccggcguagGauaacggcuagaau.......................................................................     1
Gi1_4193194_x1...............................................gguucucacccaacagaucc..............................................     0
Gi5_9111430_x1...................cggcguaguauCacggcuaga.........................................................................     1
Gi1_4290788_x1.................uccggcgGaguauaacggcuagaaugguugg.................................................................     1
Gi1_4331175_x1....................................................................gguucgauucccggcgccUga........................     1
Gi1_4323829_x1....................................................................gguucgauGcccggcgccgga........................     1
Gi1_4190688_x1...............................................gCuucucacccaacagauccgg............................................     1
Gi2_11116921_x1.................uccggcguaguauaacUgcuag..........................................................................     1
```

```
Gi5_8977628_x1.............................................ugguCcucacccaacagauccgg............................................      1
Gi5_9019155_x1.....................................................................uucgauucccggcgccgA.........................      1
Gi3_28288256_x1...............................................guucucacccGacagauccgggu..........................................      1
Gi1_4217601_x1..............................................gguucucacccaacCgauccgg............................................      1
Gi5_9120589_x1.................uccggcguaguauaaGggc............................................................................      1
Gi2_11004858_x1.................uccggcAuaguauaacggcuagaa.......................................................................      1
Gi5_9075210_x1...................................................ucacccaacagauccggguC.........................................      1
Gi2_11295088_x1...................................................................gguNcgauucccggcgccgga........................      1
Gi5_8865560_x1.................uccggcguaguauCacggcu...........................................................................      1
Gi1_4374162_x1.....................................................acccaacagauccggguucgauucUcg................................      1
Gi3_28266730_x1...............................................guucucacccaGcagauccgggu..........................................      1
Gi2_11393863_x1.................uccggAguaguauaacggcuag.........................................................................      1
Gi2_11331612_x1.....................................................................uucAauucccggcgccgg.........................      1
Gi3_28744785_x1.................................................ucucacccaacagCuccgg............................................      1
Gi3_28467124_x1.............................................ugguucucacccaacagauccAg............................................      1
Gi5_8946059_x1.................uccUgcguaguauaacggcuagaau......................................................................      1
Gi3_28492538_x1................................................uucucacccaacaCauccgg............................................      1
Gi2_11020624_x1....................................................................guucgauuccAggcgccgga........................      1
Gi3_28313780_x1......................................................cccaacagauccggguucgauucUc.................................      1
Gi2_11322039_x1...............................................guuAucacccaacagauccg.............................................      1
Gi5_8934544_x1.....................................................................guucgauCcccggcgccggag......................      1
Gi4_572129_x1 ...................................................................gguuAgauucccggcgccggagc......................      1
Gi1_4382342_x1............................................................agauccggguucgauCccc.................................      1
Gi5_9122569_x1..................ccggUguaguauaacggcuag.........................................................................      1
Gi4_604592_x1 ...................................................................gguucgauuUccggcgccgg.........................      1
Gi5_8856754_x1.................Nccggcguaguauaacggcua..........................................................................      1
Gi3_28908752_x1..................................................cucacccaCcagauccgg............................................      1
G1a_6166156_x1.................uccggcguaguaGaacggcuag.........................................................................      1
Gi1_4311669_x1...................................................................gguucgauuccAggcgccg..........................      1
Gi1_4327164_x1..............................................................auccggguucgauucccggcgccggU........................      1
Gi3_28694185_x1.................uccggcguaguauaNcggcuaga........................................................................      1
Gi1_4260712_x1.................................................ucucacccaacagGuccg.............................................      1
Gi5_9132180_x1.................uccggcguaguauaaUggcuagaa.......................................................................      1
Gi5_9095721_x1..............................................gguucucacccaacagGuc...............................................      1
Gi3_28422376_x1...............................................................uccggguucgauucccAgcgccgga........................      1
Gi5_8941812_x1...................................................................gguucgauucccggcgccggagcguuC..................      1
Gi3_28548837_x1.................................................................cgggCucgauucccggcgccgga........................      1
Gi2_11043616_x1....................................................................guucgUuucccggcgccgga........................      1
Gi1_4271575_x1...................................................................gAuucgauucccggcgccggag.......................      1
Gi4_576983_x1 ....................................................................guucAauucccggcgccgg.........................      1
Gi2_11189622_x1.....................................................................uucgauucccggcgUcgg.........................      1
Gi5_9016215_x1.................uccggcgGaguauaacggcuagaau......................................................................      1
Gi5_8872509_x1..................................................................ggguucgCuucccggcgccggagc......................      1
Gi4_600706_x1 .................uccggcguNguauaacggcuag.........................................................................      1
Gi5_9019263_x1...................................................................gguucgauucUcggcgccgga........................      1
Gi1_4354265_x1..........................................................................................guuucccgccuuccuucgucuu      0
Gi3_28658183_x1.................................................................cggguucgauucccggcgccggG........................      1
```

```
Gi4_575011_x1 .........................................................................auucccggcgccggagcC.....................      1
Gi1_4230138_x1.................uccggGguaguauaacgg.............................................................................      1
Gi3_28580965_x1............................................uuggguucucacccaUcagaucc.............................................      1
Gi3_28883800_x1................................................uucucacGcaacagauccggguuc........................................      1
Gi1_4315732_x1.................uUcggcguaguauaacggcu...........................................................................      1
Gi2_11036911_x1.................uccggcguaguauaaAggcua..........................................................................      1
Gi3_28409719_x1.................uccggcCuaguauaacggcuag.........................................................................      1
Gi1_4251497_x1...............................................guucucacccaacagaGccg.............................................      1
Gi1_4319907_x1....................................................................guuNgauucccggcgccgga........................      1
Gi5_9049413_x1.............................................ugguuAucacccaacagauccgggu..........................................      1
Gi1_4162336_x1...............................................................uccggguucgauucccggcgccgg.........................      0
Gi5_8993100_x1...............................................guucucacccaacagauGcg.............................................      1
Gi1_4227510_x1...............................................guucucacccaaGagauccgg............................................      1
Gi2_11263507_x1...................................................................gguucUauucccggcgccg..........................      1
Gi3_28426008_x1......................................................................ucgauuAccggcgccggagc......................      1
Gi4_573755_x1 .....................................................................uucgauucccggcgccgg.........................      0
Gi5_9104674_x1....................................................................guucgauucccgCcgccgga........................      1
Gi2_11231128_x1............................................................agauccggguucgauucccggcgccggag.......................      0
Gi2_11120609_x1......................................................................ucgauuccAggcgccggag.......................      1
G1a_6139298_x1................................................uucucaccAaacagauccg.............................................      1
Gi5_8867507_x1..........................................................acagaucGggguucgauucccggcgc............................      1
Gi1_4373113_x1......................................................cccaacUgauccggguuc........................................      1
Gi2_11000074_x1......................................................................ucgaGucccggcgccggag.......................      1
Gi5_8851114_x1.............................................ugguucucacccaacagauccggAu..........................................      1
Gi2_11088895_x1....................................................................Nuucgauucccggcgccggagc......................      1
Gi2_11292939_x1....................................................................guucgauuUccggcgccggag.......................      1
Gi2_11293023_x1................................................uucAcacccaacagauccggguucg.......................................      1
Gi2_11197426_x1.................uccggcguaguauaCcggcuag.........................................................................      1
Gi2_11314643_x1.....................................................................uucgauucccggcgccggUg.......................      1
Gi5_8876136_x1.................uccgUcguaguauaacggcu...........................................................................      1
Gi1_4323888_x1....................................................................gGucgauucccggcgccggag.......................      1
Gi3_28546806_x1.......................................................................cgauucccgAcgccggagc......................      1
Gi3_28846220_x1..................................................................ggguucgaNucccggcgccggagc......................      1
Gi5_9012621_x1................................................uucucacccaacagauccggguC.........................................      1
Gi1_4352878_x1..............................................gguucucacccaacagauccAg............................................      1
Gi1_4201504_x1..............................................ggNucucacccaacagauccg.............................................      1
Gi2_11031774_x1.......................................................................cAauucccggcgccggagcguuuc.................      1
Gi5_9084857_x1...............................................................uGcggguucgauucccggc..............................      1
Gi2_11222338_x1....................................................................guNcgauucccggcgccggagc......................      1
G1a_6161345_x1.................uccggcguaguauaacAgcu...........................................................................      1
Gi1_4199746_x1.................................................ucucacccaacagauUcg.............................................      1
Gi3_28429230_x1................................................uucucUcccaacagauccgg............................................      1
Gi3_28771419_x1....................ggcguaguauaacggcuagCau......................................................................      1
Gi4_569538_x1 ....................................................................guucUauucccggcgccgga........................      1
Gi1_4325991_x1.................uccggcguaguauaacggcuagaUu......................................................................      1
Gi3_28984872_x1.................................................ucucUcccaacagauccgg............................................      1
Gi3_28979015_x1.....................................................................Gucgauucccggcgccggag.......................      1
```

```
Gi2_11236544_x1.................uccggcguaguauaacgUcuagaau......................................................................     1
Gi1_4333676_x1.............................................ugguucucacccaacUgauccg.............................................     1
Gi2_11288815_x1...................................................................gguAcgauucccggcgcc...........................     1
Gi2_11116811_x1...................cUgcguaguauaacggcuaga........................................................................     1
Gi2_11018820_x1.....................................................................uAcgauucccggcgccggagc......................     1
Gi5_8928999_x1.............................................ugguucucacAcaacagauccgggu..........................................     1
Gi5_9071109_x1.................uccggcguaguauaacggcuagaauggGu..................................................................     1
Gi5_9119908_x1.....................................................................uucgauucccggcgcAgg.........................     1
Gi3_28330811_x1..............................................gguucucUcccaacagauccg.............................................     1
Gi3_28266424_x1............................................Gugguucucacccaacaga.................................................     1
Gi5_9125235_x1..........................................................................................guuucccgccCuccuucgucu.     1
Gi1_4354254_x1....................................................................guucgCuucccggcgccg..........................     1
Gi1_4312696_x1..........................................................................uucccggcgccggagcgA....................     1
Gi1_4362114_x1.................................................ucucacccUacagauccgg............................................     1
Gi1_4373711_x1.................uccggcguaCuauaacgg.............................................................................     1
Gi1_4242184_x1..................Acggcguaguauaacggcuag.........................................................................     1
Gi1_4322936_x1............................................uugguucucacccaacagauccg.............................................     0
Gi1_4285101_x1.................uccUgcguaguauaacggcuag.........................................................................     1
Gi2_11164530_x1....................................................................guucgauucccUgcgccgga........................     1
Gi5_8855431_x1.................uccggcguaUuauaacggc............................................................................     1
Gi5_8851787_x1.................uccggcguCguauaacggcu...........................................................................     1
Gi3_28672800_x1.................uccggcguaguaGaacggcuag.........................................................................     1
G1a_6197386_x1.................uccgUcguaguauaacggcuagaa.......................................................................     1
Gi5_8980189_x1.................uccggcguaguCuaacggcuagaa.......................................................................     1
Gi4_598994_x1 ....................................................................gguucgauucccggUgccgg........................     1
Gi3_28670617_x1......................................................cccaacagauccggguuc........................................     0
Gi2_11127044_x1......................................................................ucgauucccggcgccggagcA.....................     1
G1a_6310241_x1....................................................................Cuucgauucccggcgccg..........................     1
Gi3_28622818_x1...................cggcguaguauaacgAcuagaau......................................................................     1
Gi5_8947925_x1....................................................................guucgauucccggcgccgAag.......................     1
Gi3_28554081_x1..................................................................ggguucgGuucccggcgccg..........................     1
Gi3_28391790_x1...................................................................Uguucgauucccggcgccggagcguu...................     1
Gi2_11016792_x1...........................................................cagauccggguucgauuccc.................................     0
Gi5_9106455_x1..............................................................auccggguucgauucccggcgccggagc......................     0
Gi1_4343697_x1..................................................cucacccaacagauccggU...........................................     1
Gi5_9001673_x1..................ccggcguaNuauaacggcu...........................................................................     1
Gi5_8960439_x1...............Gguccggcguaguauaacggcuagaaugg....................................................................     1
Gi5_8943502_x1...............................................guucucacccaacaUauccg.............................................     1
Gi2_11373101_x1...............................................guucucaAccaacagauccg.............................................     1
Gi2_11111681_x1....................................................................guucgauuccGggcgccggagc......................     1
Gi2_11294997_x1..............................................gguucucacccaacagauccgggu..........................................     0
Gi5_9067346_x1....................................................................guucgauucccggcgGcgga........................     1
Gi5_8890299_x1.............................................ugguucucacccaacagaucAg.............................................     1
Gi3_28723782_x1.................................................................Aggguucgauucccggcgccgga........................     1
Gi3_28813720_x1.............................................uggAucucacccaacagauccg.............................................     1
Gi5_8907661_x1...................................................................Aguucgauucccggcgccggagcguu...................     1
Gi1_4218610_x1.....................................................................uucgauuccAggcgccgg.........................     1
```

```
Gi1_4205070_x1.................uccggcguaguauaGcggc............................................................................	1
Gi2_11134309_x1................................................................cgggGucgauucccggcgccgga.........................	1
Gi3_28617586_x1...............................................................uccggguucgauucccggcgccggC........................	1
Gi1_4366445_x1.................................................ucucacccaaUagauccgg............................................	1
Gi3_28420742_x1..................................................cucacccaacagauccgC............................................	1
Gi3_28746704_x1............................................................aCauccggguucgauucccggcgccgg.........................	1
Gi1_4306787_x1..................................................cucacccaGcagauccggguucga......................................	1
Gi4_595506_x1 ...................................................................gguuUgauucccggcgccgg.........................	1
Gi3_28728177_x1............................................................agauccgggCucgauucc..................................	1
Gi3_28339293_x1...................................................ucacccUacagauccggguuc........................................	1
Gi3_28766235_x1........................................................caacagauccggguucgC......................................	1
Gi3_28323073_x1......................................................cccaacagauccgggCucgauuccc.................................	1
Gi5_9138450_x1.................................................ucucacccaacagGuccgg............................................	1
Gi1_4343275_x1.................................................ucucacccaacaAauccgg............................................	1
Gi1_4326623_x1....................................................................guucgauAcccggcgccg..........................	1
Gi3_28677760_x1....................ggcguaguNuaacggcua..........................................................................	1
Gi2_11219074_x1......................................................cccaacagGuccggguuc........................................	1
Gi5_8877020_x1.....................................................................uucgauucccggcgccggagcgA....................	1
Gi3_28593556_x1..............................................................auccAgguucgauucccggcgccgga........................	1
Gi3_28759482_x1.................uccggcguaguauaacggUu...........................................................................	1
Gi1_4161951_x1.................uccggcguaguauaaGgg.............................................................................	1
G1a_5996456_x1..................................................................ggguucgauucccggcgccggagcC.....................	1
Gi3_28843634_x1...................cggcguaguauaacggcuaAa........................................................................	1
Gi2_11366978_x1......................................................................ucgauAcccggcgccggagc......................	1
Gi3_28578239_x1....................................................cacccaacagaucAggguuc........................................	1
Gi3_28912144_x1..............................................................auccggguuAgauucccggcgccgga........................	1
Gi5_9011318_x1...................................................................gguucgauucccggcgccgUagc......................	1
Gi2_10995462_x1.................uccggcgGaguauaacggcuag.........................................................................	1
Gi3_28706486_x1.................................................ucucacccaacagaucUgg............................................	1
Gi1_4215423_x1...................................................ucacccaacagauccgCgu..........................................	1
Gi5_9071310_x1.............................................uAguucucacccaacagauccgg............................................	1
Gi5_8907153_x1..............................................gguucucaGccaacagauccg.............................................	1
Gi5_9018245_x1...................................................................gguucgauucccggcgccAgagcguu...................	1
Gi2_11190614_x1................................................uucucacccaacaAauccg.............................................	1
Gi5_9063088_x1....................................................................guucgauucccggcCccgga........................	1
Gi5_8887622_x1.................uccggcguaguauaacgA.............................................................................	1
Gi5_8957435_x1....................................................................guucgauuccGggcgccg..........................	1
Gi3_28714050_x1...........................................................cagauccggguucgaCucccggcgccgg.........................	1
Gi2_11379186_x1.......................................................................cgauucccggcgccggagcguuuA.................	1
G1a_6208819_x1.................uccggcguCguauaacggcuag.........................................................................	1
Gi3_28909169_x1....................ggcguagCauaacggcuaga........................................................................	1
Gi2_11041640_x1.......................guaguGuaacggcuagaau......................................................................	1
Gi3_28309427_x1...............................................guuAucacccaacagauccgg............................................	1
Gi3_28589833_x1.............................................ugguucucacccaacagaucc..............................................	0
Gi3_28351186_x1...............................................guucucacccaacagaucc..............................................	0
Gi2_11278047_x1................................................................ccCgguucgauucccggcgccggagc......................	1
Gi4_593486_x1 ...................................................................Cguucgauucccggcgccgg.........................	1
```

```
Gi5_8857640_x1...................................................................gguucgUuucccggcgccggag.......................      1
Gi2_11321386_x1.................uccggcguaguaAaacggcua..........................................................................      1
Gi5_9011451_x1.................uccggcgAaguauaacggcuagaau......................................................................      1
Gi2_11005068_x1.............................................gguuAucacccaacagauccg..............................................      1
Gi5_9082787_x1...............................................uucucacccUacagauccggguu..........................................      1
Gi2_11373735_x1......................................................................Acgauucccggcgccggag.......................      1
Gi2_11285736_x1.......................................................................cgauucccggcgccggCgcguuu..................      1
Gi5_8975325_x1............................................uugguucucacUcaacagauccg.............................................      1
Gi1_4188205_x1..................ccggcguGguauaacggc............................................................................      1
Gi5_9061607_x1..............................................ggAucucacccaacagauccgg............................................      1
Gi1_4334308_x1..................ccggcguCguauaacggc............................................................................      1
Gi5_8857946_x1.............................................ugguucucaUccaacagauccgg............................................      1
Gi5_8982493_x1...................................................................gguucgauucccggcgGcg..........................      1
Gi3_28307645_x1....................ggcguaguauaacggcuaUa........................................................................      1
Gi2_11302589_x1.................................................................cggguucgauucAcggcgccgga........................      1
Gi3_28618792_x1...................................................................gguucgauucAcggcgcc...........................      1
G1a_6149094_x1.................uccgAcguaguauaacggc............................................................................      1
Gi1_4301285_x1...................................................................gguucgauuUccggcgccggagc......................      1
Gi1_4319024_x1..............................................gguucucacccaaAagauccg.............................................      1
Gi1_4283066_x1...................................................................gAuucgauucccggcgcc...........................      1
Gi2_11241331_x1.........................................................................auucccggcgccggagcC.....................      1
Gi1_4271800_x1..................................................cucacccaacUgauccgg............................................      1
Gi2_11053440_x1......................................................................ucgauucccggcgcUggagc......................      1
Gi3_28737614_x1......................................................................ucAauucccggcgccgga........................      1
Gi3_28395434_x1...................cggcgCaguauaacggcuagaau......................................................................      1
Gi2_11180345_x1..................................................................Ugguucgauucccggcgccggagc......................      1
Gi1_4367709_x1...........................................................cGgauccggguucgauucccggcgcc...........................      1
Gi5_9107136_x1...............................................................ucAggguucgauucccggcgccg..........................      1
Gi5_8942143_x1................................................uucAcacccaacagauccgg............................................      1
Gi5_8857528_x1...................................................................gguucgGuucccggcgccggagcguu...................      1
Gi5_9103469_x1............................................................aCauccggguucgauuccc.................................      1
Gi5_8895251_x1..............................................gguucucaccUaacagauccgg............................................      1
Gi5_9091215_x1.............................................uggAucucacccaacagauccg.............................................      1
Gi3_28633437_x1...................cggcguaguauaacggUuaga........................................................................      1
Gi3_28743439_x1..........................................................acagauccggguucgauucccggcgccgAa........................      1
Gi2_11346721_x1...............................................guucucacccaacagaucUgg............................................      1
G1a_6154963_x1.....................................................................uuUgauucccggcgccggagc......................      1
G1a_6021875_x1...............................................guucucacccaacagaucAg.............................................      1
Gi3_28985193_x1..................................................................ggguucgauucccggcgccggaCc......................      1
Gi4_604099_x1 .............................................ugguucucacccaacagauccgggu..........................................      0
Gi2_11051947_x1......................................................cccaacaUauccggguuc.......................................      1
G1a_6280602_x1...................................................................gguucgGuucccggcgccggag.......................      1
Gi5_9020509_x1..............................................gguucucacccaacagauccC.............................................      1
Gi1_4358284_x1...................................................................gguuAgauucccggcgccg..........................      1
G1a_6153473_x1...................................................................gguucgauucAcggcgccgga........................      1
Gi1_4196051_x1...............................................guucucacccaacagauccgC............................................      1
Gi1_4202460_x1...............................................................uccAgguucgauucccggcgccgga........................      1
```

```
Gi1_4175368_x1...................................................................gguucgauucccggcAccggag.......................	1
Gi3_28921023_x1.....................................................................uucAauucccggcgccgga........................	1
Gi1_4283334_x1.............................................Agguucucacccaacagauccg.............................................	1
Gi3_28501653_x1........................................................cUacagauccggguucga......................................	1
Gi2_11110564_x1.............................................ugguucucacccaacagaCcc..............................................	1
Gi5_8974533_x1......................................................cccaacagCuccggguuc........................................	1
Gi1_4352544_x1......................................................cccaacagauccggguuc........................................	0
Gi3_28283721_x1............................................uugguucucacccaCcagaucc..............................................	1
Gi5_8983010_x1...............aguccggcguaguauaacgAcuagaaugg....................................................................	1
Gi3_28689156_x1......................................................................ucgauucccgAcgccggag.......................	1
Gi5_9067315_x1......................................................cccaacagauccgAguuc........................................	1
Gi5_9114512_x1...................................................................gguucgauucccggcgccggagcgCuu..................	1
Gi2_11363201_x1...................cUgcguaguauaacggcuagaau......................................................................	1
Gi3_28335121_x1.................uccggcguagGauaacggcuagaa.......................................................................	1
Gi2_10997984_x1................................................uucucGcccaacagauccg.............................................	1
Gi3_28625718_x1..................................................................ggguucgaCucccggcgccggag.......................	1
Gi1_4360748_x1.................uccggcguaguaAaacgg.............................................................................	1
Gi1_4224993_x1..................................................cucacccaacagauccggguucg.......................................	0
Gi5_9054686_x1...............................................guucucacccaacagaucc..............................................	0
Gi3_28552848_x1....................................................................guucgauucccggcUccg..........................	1
Gi3_28501236_x1.................uNcggcguaguauaacggcuaga........................................................................	1
Gi2_11298164_x1......................................................................uGgauucccggcgccgga........................	1
Gi5_8903697_x1...................................................................gguucgauucccggcUccggagc......................	1
Gi2_11062455_x1....................................................................guucgauucccggcgccggagcg.....................	0
Gi1_4319217_x1...............aguccggcguaguauaacgg.............................................................................	0
Gi2_11300518_x1...........................................................Gagauccggguucgauucccggcgccggag.......................	1
Gi2_11397611_x1..................ccUgcguaguauaacggcua..........................................................................	1
Gi5_9051131_x1................................................uAcucacccaacagauccggguuc........................................	1
Gi3_28418808_x1....................................................................guucgauuccGggcgccg..........................	1
Gi3_28426142_x1...............................................................uccggguucgCuucccggcgccgga........................	1
Gi1_4246433_x1.................uccggcguaguauaCcggcu...........................................................................	1
Gi3_28337109_x1....................................................................guucgauGcccggcgccgg.........................	1
Gi3_28504071_x1...............................................guucucacccaacagUuccgg............................................	1
Gi2_11279186_x1...............................................guucucacccaaGagauccg.............................................	1
G1a_6327152_x1...................................................................gguucgauuccUggcgccgga........................	1
Gi5_9038982_x1.......................guaguauaacgCcuagaau......................................................................	1
Gi5_9107204_x1....................ggcguaguGuaacggcuag.........................................................................	1
Gi3_28962871_x1.................uccggcguaguauaacggG............................................................................	1
Gi2_11233810_x1.................uNcggcguaguauaacggc............................................................................	1
Gi2_11210225_x1......................................................................ucAauucccggcgccggagc......................	1
Gi5_8923647_x1.................uccggcguaguauaacggcGa..........................................................................	1
Gi2_11403150_x1.......................................................................cgauucccggcgccggagcguuC..................	1
Gi3_28685562_x1...............................................guucucacccaacagaucGgg............................................	1
Gi1_4365740_x1......................................................................ucgUuucccggcgccggagc......................	1
Gi3_28713029_x1.................uccggcguaguauaacggcuagaUu......................................................................	1
Gi1_4390025_x1.................................................ucucacccCacagauccgg............................................	1
G1a_6140685_x1.................uccggcguaguauaacggcuCga........................................................................	1
```

```
Gi3_28329919_x1.....................................................cUcccaacagauccggguuc........................................     1
Gi3_28898428_x1................................................guucucacccaacagaGccg.............................................     1
Gi5_8907632_x1....................................................ucacccaCcagauccggguu.........................................     1
Gi1_4249945_x1..................Ucggcguaguauaacggcu............................................................................     1
Gi1_4308228_x1...................................................................Agguucgauucccggcgccg..........................     1
Gi2_11237992_x1......................................................................uucgauuccUggcgccggagc......................     1
G1a_6083325_x1....................................................................gguucgauucccggAgccgg.........................     1
Gi5_9096651_x1......................................................acccaacagauccggguucgaCuccc.................................     1
Gi1_4237244_x1......................................................................uucgauucccggcgccAg.........................     1
Gi2_11005910_x1............................................................caUauccggguucgauucccggcgccgga........................     1
Gi5_9056287_x1.............................................Cugguucucacccaacagauccg.............................................     1
Gi3_28886212_x1.....................gcguaguauaacggcuagG..........................................................................     1
Gi5_8956571_x1...................................................................ggguucgauuccUggcgccggagc......................     1
Gi3_28493974_x1.....................................................cacccaacagGuccggguuc........................................     1
Gi3_28784225_x1......................................................................uucgUuucccggcgccgg.........................     1
Gi3_28943001_x1...............................................................auccggguucgauucccggcAccgga........................     1
Gi5_9086408_x1................................................guucucacccaacaCauccgg............................................     1
Gi2_11157678_x1....................................................................gguucgauucccgUcgccggag.......................     1
Gi3_28298784_x1.................................................uucucacccaacagauGcgg............................................     1
Gi2_11236569_x1..............................................ugguucucaAccaacagaucc..............................................     1
Gi5_9121068_x1..................................................ucucacccaacagauUcg.............................................     1
Gi5_9116513_x1..................................................ucucacccaacGgauccgggu..........................................     1
Gi3_28438278_x1.........................................................................gauucccggcgccggagcU.....................     1
Gi3_28343661_x1......................................................................uucgaCucccggcgccggag.......................     1
G1a_6152621_x1....................................................................Cguucgauucccggcgccgg.........................     1
Gi2_11111602_x1..................ccggcguaguauaacggcu............................................................................     0
Gi2_11323443_x1..................Ucggcguaguauaacggcuaga.........................................................................     1
Gi5_8964280_x1....................ggcguaguCuaacggcuag..........................................................................     1
Gi5_8884316_x1..................................................................cggguucgauucccggUgc............................     1
Gi1_4215364_x1..............................................ugguucucacccaacCgauccg.............................................     1
Gi2_11335105_x1.................uccgCcguaguauaacggcua...........................................................................     1
Gi2_11308415_x1.................uccggcguaCuauaacggcua...........................................................................     1
Gi2_11317602_x1................................................guucucacccaacagaucGg.............................................     1
Gi1_4300720_x1.................uAcggcguaguauaacggcuagaau.......................................................................     1
G1a_6043894_x1.................uUcggcguaguauaacggc.............................................................................     1
Gi3_28682260_x1....................................................................gguucgauucGcggcgccg..........................     1
Gi1_4296745_x1....................................................................gguucgauucccggcgccggaC.......................     1
Gi1_4199628_x1.....................................................................guucgauucccggcgccCgagc......................     1
G1a_6082016_x1.................uccggcguaguauaacggcCagaa........................................................................     1
Gi3_28418507_x1......................................................................uucgauuGccggcgccggagc......................     1
Gi1_4265161_x1..................................................ucucacAcaacagauccgg............................................     1
Gi4_595786_x1 .....................................................................guucgCuucccggcgccgg.........................     1
Gi3_28803447_x1...................cggcguGguauaacggcua...........................................................................     1
Gi3_28268109_x1.................uccggcguaguaAaacggcua...........................................................................     1
Gi4_591325_x1 .....................................................................guucgauucUcggcgccgg.........................     1
Gi2_11024176_x1...................................................................ggguucgauucccggUgcc...........................     1
Gi2_11311669_x1.........................................................................gauucccggcgcGggagc......................     1
```

```
Gi2_11121073_x1...................................................................gguucgauucccgAcgcc...........................     1
Gi3_28882545_x1....................ggcguaguauUacggcuag.........................................................................     1
Gi3_28773590_x1....................ggcguCguauaacggcuagaau......................................................................     1
Gi3_28545311_x1.........................................................aacagauccggguucgauucccggcgUcgga........................     1
Gi5_8946036_x1..................ccggcguaguauaaAggcua..........................................................................     1
Gi1_4312727_x1..............................................gguucucaccUaacagauccgg............................................     1
Gi5_8840045_x1......................................................................ucgauucccggcgccgAa........................     1
Gi2_11240885_x1..................cGggcguaguauaacggcuagaau......................................................................     1
Gi2_11352120_x1..................ccggcguaguauaGcggcua..........................................................................     1
Gi2_11275861_x1...............aguccggUguaguauaacgg.............................................................................     1
Gi4_584974_x1 ....................................................................Cuucgauucccggcgccgg.........................     1
Gi2_11093458_x1...............................................guucGcacccaacagauccgg............................................     1
Gi5_9000461_x1....................ggUguaguauaacggcuaga........................................................................     1
Gi1_4225088_x1...................................................................gguucgauucccggAgccg..........................     1
Gi3_28544544_x1.............................................ugguucucacccUacagauccg.............................................     1
Gi5_9062821_x1....................ggcguaguauaacggcCag.........................................................................     1
Gi2_11106519_x1...................................................ucacccaacagauccggguuc........................................     0
Gi5_8946116_x1...............................................uucucacccaacagaCccg..............................................     1
Gi4_606396_x1 ....................................................................guucgaGucccggcgccgg.........................     1
Gi3_28881524_x1..................Gcggcguaguauaacggcuag.........................................................................     1
Gi3_28418844_x1................................................uucucacccaacagauccgAguuc........................................     1
Gi5_9124826_x1..............................................gguucGcacccaacagauccg.............................................     1
Gi3_28446037_x1......................................................cccaacagGuccggguucgauuccc.................................     1
Gi4_597138_x1 ....................................................................guucgauucccggUgccgga........................     1
Gi3_28263261_x1.................uccggcguaguauaacgCcuagaa.......................................................................     1
Gi5_9088187_x1................................................................ccggguucgauucccggcgccggU........................     1
Gi2_11346832_x1............................................................agauccggguucgCuucc..................................     1
Gi2_11021814_x1.................uccggcguaguauaacggcuaCaa.......................................................................     1
Gi5_8960465_x1...............................................guucucacccaacagaucGg.............................................     1
Gi5_8906067_x1.............................................ugguucucacccaacagauUcg.............................................     1
Gi1_4307382_x1...............aguccggUguaguauaacggcua..........................................................................     1
Gi5_8908734_x1...............................................guuGucacccaacagauccgg............................................     1
Gi2_11261799_x1...................................................................gguucgauuAccggcgccggag.......................     1
G1a_6213932_x1.................uccggcguaguauaCcgg.............................................................................     1
Gi5_8988260_x1...................................................ucacccaaNagauccggguu.........................................     1
Gi3_28439388_x1...................................................................gNuucgauucccggcgccggagc......................     1
Gi3_28683519_x1.................................................ucucacccNacagauccg.............................................     1
Gi4_582552_x1 ...................................................................gguCcgauucccggcgccgg.........................     1
G1a_6080800_x1.................uccggcguaguGuaacggcua..........................................................................     1
G1a_6017759_x1.................Gccggcguaguauaacggcuagaau......................................................................     1
Gi5_8965890_x1.......................................................................Agauucccggcgccggagcguuuc.................     1
Gi3_28759523_x1..................................................................gAguucgauucccggcgccggagc......................     1
Gi3_28346019_x1..............................................gguucuUacccaacagauccgggu..........................................     1
Gi3_28787669_x1.................uccCgcguaguauaacggcua..........................................................................     1
Gi1_4180668_x1...............Uguccggcguaguauaacggcuag.........................................................................     1
Gi5_8925195_x1.................................................ucucacccaacagauccgC............................................     1
Gi2_11200439_x1...................Gggcguaguauaacggcuagaau......................................................................     1
```

```
Gi1_4374990_x1...............................................guucucacccaacagauccgggu..........................................     0
Gi3_28325103_x1.............................................ugguuUucacccaacagauccg.............................................     1
Gi1_4316704_x1...................................................................gguucgauucccggcgccgUag.......................     1
Gi5_8915832_x1......................................................cccaacagauccggguAc........................................     1
Gi1_4278973_x1.................uccggcguaguauaacgCcua..........................................................................     1
Gi3_28538421_x1..................ccggcguaguauaacggcuaA.........................................................................     1
Gi5_9064515_x1................................................Aucucacccaacagauccggguu.........................................     1
Gi5_8968586_x1...................................................................gguucgauGcccggcgccgg.........................     1
Gi3_28348367_x1...................................................................gguucgaAucccggcgcc...........................     1
Gi3_28603978_x1....................ggcguaguauaacAgcuaga........................................................................     1
Gi1_4374259_x1..................................................cucaccAaacagauccgg............................................     1
Gi3_28783069_x1......................................................................ucgauucccggcUccggagc......................     1
G1a_6095435_x1.................uGcggcguaguauaacggcu...........................................................................     1
Gi2_11307595_x1..............................................guucucacccaacagaucGgg.............................................     1
Gi3_28396191_x1.............................................ugguucucUcccaacagauc...............................................     1
Gi5_9087095_x1...................................................................gguucgCuucccggcgccggagcg.....................     1
Gi5_9112409_x1..............................................gguucucacccaacGgauccggguu.........................................     1
Gi5_9082933_x1..............................................gguucucacccaacagauccAg............................................     1
Gi3_28767525_x1......................................................................ucgCuucccggcgccggagc......................     1
Gi5_9097109_x1..........................................................aUagauccggguucgauucccggcgc............................     1
Gi3_28528581_x1.......................................................................cAauucccggcgccggagc......................     1
Gi5_8919154_x1..................cUggcguaguauaacggcuaga........................................................................     1
Gi1_4283760_x1.............................................ugguucuAacccaacagauccg.............................................     1
Gi3_28590145_x1....................................................................guucgauuGccggcgccggag.......................     1
Gi2_11100601_x1......................................................................ucgauucccggcUccggag.......................     1
Gi2_11337343_x1...................................................................gguucgauucGcggcgcc...........................     1
Gi3_28963182_x1.................uccggcguaguauaacggcGagaau......................................................................     1
Gi4_604450_x1 ..............................................gguucucacccaacGgauccg.............................................     1
Gi3_28488094_x1...........................................................Gagauccggguucgauucccggcgccgg.........................     1
Gi5_8895369_x1........................................................................gauucccggcgcAggagc......................     1
Gi1_4214280_x1...............................................guucucacccaacagauccgA............................................     1
Gi5_8866308_x1.................uccggcguaguauCacggcuagaauggu...................................................................     1
Gi2_11357110_x1.....................................................................uucgGuucccggcgccgg.........................     1
Gi2_11236176_x1.................uccggcAuaguauaacggcuag.........................................................................     1
Gi2_11407350_x1......................................................................ucgauucccCgcgccggagc......................     1
Gi3_28269762_x1.....................................................cacccaacagauccgggGuc.......................................     1
Gi3_28302604_x1...............................................gAucucacccaacagauccg.............................................     1
Gi2_11107148_x1.........................................................aacagauccggguuAgauucccggcgccg..........................     1
G1a_6049064_x1................................................uucucacccaacagaCccgg............................................     1
Gi4_604894_x1 .................uccAgcguaguauaacggcuaga........................................................................     1
Gi5_9099788_x1...............aguccggcguagGauaacggcu...........................................................................     1
Gi5_9050913_x1.................................................ucucacUcaacagauccg.............................................     1
Gi3_28686329_x1............................................................agCuccggguucgauucc..................................     1
Gi1_4224661_x1................................................uucucacccaacagaGccgg............................................     1
Gi5_8887075_x1...................................................................gguucgUuucccggcgccgg.........................     1
Gi2_11162931_x1...............................................Auucucacccaacagauccgggu..........................................     1
Gi3_28310326_x1.............................................................gauccggguCcgauccc..................................     1
```

```
Gi3_28616757_x1.................ucAggcguaguauaacggcua..........................................................................     1
Gi4_609748_x1 ...................................................................gguucgauucccggcgccggagcCu....................     1
Gi1_4386623_x1................................................uucucCcccaacagauccgg............................................     1
Gi5_9007222_x1.................uccggcguagAauaacggc............................................................................     1
Gi2_11070776_x1......................................................................ucgauucccggcgccUgag.......................     1
Gi1_4191986_x1..............................................................aCccggguucgauucccggcgccgg.........................     1
Gi5_9056183_x1.....................................................acccaacagauccggguucgauCcc..................................     1
Gi3_28976315_x1..............................................gguucucGcccaacagauccgg............................................     1
Gi3_28860126_x1......................................................cccaacagauccggguucgauuGcc.................................     1
Gi3_28987095_x1......................................................cccaacagauccggguucgaCuccc.................................     1
Gi1_4173855_x1..................ccgAcguaguauaacggcuag.........................................................................     1
G1a_6126156_x1.................uccggcguaguCuaacggcu...........................................................................     1
Gi1_4368147_x1.................................................uAucacccaacagauccg.............................................     1
Gi5_9129461_x1...................................................................gguucgauucccgCcgccgg.........................     1
G1a_6103745_x1...............................................guGcucacccaacagauccg.............................................     1
Gi3_28559884_x1......................................................ccUaacagauccggguucgauuccc.................................     1
Gi5_9062050_x1...................................................................gguucgauucUcggcgccggagcgu....................     1
Gi3_28874542_x1...................................................................gguucgauucAcggcgccgg.........................     1
Gi1_4325900_x1........................................................caacagGuccggguucgauucccggc..............................     1
Gi3_28923891_x1............................................................Ugauccggguucgauuccc.................................     1
Gi2_11356522_x1.....................gcguaguauaacggcuagaaA......................................................................     1
Gi2_11338905_x1...................................................................gguucgauucccggcgcGg..........................     1
Gi5_8935297_x1....................................................................guucgauucccCgcgccgga........................     1
Gi3_28653418_x1...............................................guNcucacccaacagauccg.............................................     1
Gi4_595029_x1 ....................................................................guucgauucccggcgAcgg.........................     1
Gi3_28873117_x1..................................................cucGcccaacagauccggguucgauu....................................     1
Gi1_4201497_x1.........................................................aacaUauccggguucgauucccggcgccgg.........................     1
Gi2_11107469_x1.....................................agaauAguugguucucacc........................................................     1
Gi4_592118_x1 ..............................................gguucucacccGacagauccg.............................................     1
Gi3_28554705_x1..............................................gguucucaNccaacagauccgg............................................     1
Gi5_9107189_x1..................................................cAcacccaacagauccgg............................................     1
Gi1_4259723_x1..............................................gguucucCcccaacagauccgg............................................     1
G1a_5995027_x1.................................................ucucGcccaacagauccg.............................................     1
Gi1_4292558_x1...............................................guucucacccaacUgauccgg............................................     1
G1a_6076007_x1.................uccggcguaguauaacAgcuagaau......................................................................     1
Gi3_28644085_x1................................................uucuUacccaacagauccggguu.........................................     1
Gi1_4353672_x1............................................................agauAcggguucgauuccc.................................     1
Gi3_28604818_x1......................................................................ucgauCcccggcgccggag.......................     1
Gi4_583360_x1 .................ucUggcguaguauaacggcuag.........................................................................     1
Gi1_4219145_x1....................ggcguaguauaacgAcuag.........................................................................     1
Gi3_28617574_x1..............................................gguuUucacccaacagaucc..............................................     1
Gi1_4267136_x1..............................................gguucucacccaacagauccA.............................................     1
Gi3_28690571_x1.................uccggcguaguauaacgCcuagaau......................................................................     1
Gi3_28688159_x1.......................................................................cAauucccggcgccggag.......................     1
Gi1_4248612_x1.................uccggcguaguGuaacggcuagaau......................................................................     1
Gi5_8877325_x1....................................................................guucgUuucccggcgccggagc......................     1
Gi3_28462976_x1...................................................................gguuNgauucccggcgccggagc......................     1
```

```
Gi2_11325663_x1...............................................................uGcggguucgauucccggcgccgga........................     1
Gi3_28557731_x1...........................................................cagauccggguucgaCuccc.................................     1
Gi5_8843088_x1..................................................................Agguucgauucccggcgccgg.........................     1
Gi5_8846253_x1..................ccggcguaguUuaacggcua..........................................................................     1
Gi5_9110145_x1...................................................................gguucgauuccAggcgccgga........................     1
Gi2_11304308_x1....................................................................guucgauuGccggcgccggag.......................     1
Gi1_4185539_x1........................................................................gauucccggcgccggagU......................     1
Gi3_28363045_x1..................ccggcguaguauaCcggcuaga........................................................................     1
Gi2_11134506_x1......................................................cccaacagauccgggGuc........................................     1
Gi1_4360244_x1.............................................ugguucucacGcaacagauccg.............................................     1
Gi2_11365551_x1........................................................................gauuccUggcgccggagc......................     1
Gi3_28734044_x1...............Cguccggcguaguauaac...............................................................................     1
G1a_6211574_x1.................uccggcguaguauGacggcuagaa.......................................................................     1
Gi3_28428452_x1......................................................................ucgaGucccggcgccggagc......................     1
Gi2_11231686_x1........................................................................gauucccggcgccggCgc......................     1
Gi2_11061990_x1......................................................................ucgauuccNggcgccgga........................     1
Gi4_604495_x1 ........................................................................gGuucccggcgccggagc......................     1
Gi3_28439206_x1...........................................................cagauccggguucgauuGcc.................................     1
Gi4_571954_x1 ....................................................................guAcgauucccggcgccgg.........................     1
Gi2_11306525_x1.................uccggcguagAauaacggc............................................................................     1
Gi5_8953895_x1................................................uucucUcccaacagauccg.............................................     1
Gi2_11345545_x1...................cAgcguaguauaacggcua..........................................................................     1
Gi5_8837034_x1...............................................guuGucacccaacagauccg.............................................     1
Gi3_28435245_x1..............................................gguCcucacccaacagauccgggu..........................................     1
Gi5_8891900_x1.................uccggcguaguauaacggGuagaau......................................................................     1
Gi3_28419469_x1...............................................................uccggguucgauucccggAgccgga........................     1
Gi3_28668767_x1.............................................ugguucucUcccaacagauccg.............................................     1
Gi3_28467341_x1.................................................ucucacUcaacagauccgg............................................     1
Gi5_8981725_x1.....................................................................Gucgauucccggcgccggagc......................     1
Gi1_4378876_x1.................uccggcguaguauaacggGua..........................................................................     1
Gi3_28399065_x1...............................................................uccggguuUgauucccggcgccgga........................     1
Gi1_4336294_x1...................................................................gguuAgauucccggcgccggag.......................     1
Gi1_4332412_x1.................uccggcguaguauaUcggcuaga........................................................................     1
Gi5_8915546_x1...................................................................gguucgUuucccggcgccggagc......................     1
Gi5_8869804_x1....................ggcguaguauaCcggcuag.........................................................................     1
Gi5_8839554_x1.................................................ucucacccUacagauccg.............................................     1
Gi3_28521586_x1................................................uucucGcccaacagauccgg............................................     1
Gi2_11058683_x1...........................................................cagauccggguucgauucccggcgccggagcC.....................     1
Gi5_8879346_x1...............................................guucucacccaacagaAccg.............................................     1
Gi1_4307754_x1.................uccggcguaguauaacggcuCgaa.......................................................................     1
Gi3_28937902_x1.............................................ugguCcucacccaacagauc...............................................     1
Gi2_11403305_x1.................uccggcguagAauaacggcuaga........................................................................     1
Gi5_8911749_x1..................ccggcguaguauaacggcuagaa.......................................................................     0
Gi4_572180_x1 ..................................................................gguucgauucccggcgGcggagc......................     1
Gi2_11112269_x1............................................................agaucGggguucgauucc..................................     1
Gi2_11009016_x1.....................................................................uGcgauucccggcgccggag.......................     1
Gi3_28974909_x1.....................................................................uucgauucccggAgccggagc......................     1
```

Gi3_28632715_x1..cucacccaacagauccggCu..	1
Gi5_9081632_x1....................ggcguaguauaacggcuagaGu..	1
Gi1_4388547_x1...Gacagauccggguucgauucccggcgccgg.........................	1
Gi2_11280951_x1...............aguccggcguaguauaGcgg...	1
G1a_6074540_x1...Cuucucacccaacagauccg...	1
Gi5_8997540_x1...ucUcccaacagauccggguu...	1
G1a_6325166_x1...ggAucgauucccggcgccggag.......................	1
Gi2_11317020_x1.................uccggcguaguauaacggcuagaaugguu..	0
Gi2_11395804_x1.................uccggcguaguauaaAggcuagaa...	1
Gi3_28399320_x1...guucucacccaacUgauccg...	1
Gi1_4310796_x1...................cggcguGguauaacggcu...	1
Gi3_28981538_x1...guGcucacccaacagauccgggu..	1
Gi1_4380110_x1..................ccggcguaNuauaacggcu...	1
Gi3_28944552_x1...Aucgauucccggcgccgga........................	1
G1a_6253918_x1...ucucacccaacCgauccg...	1
Gi3_28593613_x1..Ncgauucccggcgccggagc......................	1
Gi3_28629367_x1...ugguuAucacccaacagauccg...	1
Gi3_28381888_x1..ucgauucccUgcgccggagc......................	1
Gi1_4361419_x1..uucucacccaacagauccUg..	1
G1a_6100600_x1...gguucgauucccAgcgccgg.........................	1
Gi1_4283531_x1....................ggcguaguauCacggcuag...	1
G1a_6257853_x1...gguucgauCcccggcgccggag.......................	1
Gi5_8883673_x1..................ccggcguaguauaaUggcuag...	1
Gi2_11154551_x1...ugguuAucacccaacagaucc..	1
Gi5_8855244_x1.................uccggcguaguauaacggG..	1
G1a_6004225_x1...guucucacccaacagaCccg...	1
Gi1_4372687_x1.................uccggcgGaguauaacggcua..	1
Gi3_28972616_x1...Ngguucucacccaacagauccg...	1
Gi5_8931323_x1...............aguccggcguaguauaacggcuagaaugA..	1
Gi5_9050820_x1..uuNucacccaacagauccg...	1
Gi3_28599939_x1..................ccggcguaguCuaacggcuaga..	1
G1a_6024798_x1.................uccggcguaAuauaacggcuagaa...	1
Gi3_28940951_x1...uccggguucgauuccAggcgccgga........................	1
G1a_6183164_x1...Aguucgauucccggcgcc...........................	1
Gi3_28759870_x1..agauccggguucgauuccU.................................	1
Gi3_28613963_x1...guucucacGcaacagauccgg..	1
G1a_6263421_x1..uucucaUccaacagauccg...	1
Gi1_4217767_x1....................ggcguaguauaacggcuagU..	1
Gi5_8874790_x1.................uccggcguaguauaacggcuagaU...	1
Gi3_28716628_x1...uucgauucccggcgccUgagc......................	1
Gi2_11241927_x1.................uccggcguaguaAaacggc..	1
Gi1_4249870_x1...guugguucucacccaacagauccgggG..	1
Gi5_8962395_x1...ugCuucucacccaacagauccg...	1
Gi1_4242935_x1...............aguccggcguagCauaacgg...	1
Gi5_9092387_x1...gguucgauucccgCcgccg..........................	1
Gi3_28923004_x1.................uccggcguaCuauaacgg...	1
Gi5_8990492_x1...gguCcgauucccggcgccggagcguu...................	1

```
Gi3_28909365_x1.................uccggcguaguauaacggcuaU.........................................................................     1
Gi2_11267696_x1...................cggcguaguauCacggcua..........................................................................     1
Gi1_4261801_x1....................................................................guucgauucccggAgccggagc......................     1
Gi1_4213027_x1...................................................................gguucgauucccggcgccggaCc......................     1
Gi5_9127316_x1......................................................cccaacagauccggguucgau.....................................     0
Gi1_4170449_x1......................................................................ucgaCucccggcgccggagc......................     1
Gi3_28356424_x1...................cggcguaguauaaUggcuaga........................................................................     1
Gi5_8942041_x1..........................................................acagGuccggguucgauucccggcgc............................     1
Gi2_11031628_x1...............aguccggUguaguauaacggcuaga........................................................................     1
Gi3_28562024_x1.....................................................................uucgauuccAggcgccggagc......................     1
Gi2_11351729_x1...............................................................uccggguucgauucccggcgcUgga........................     1
Gi2_11057976_x1................................................uuNucacccaacagauccg.............................................     1
Gi3_28294130_x1....................................................................guucgauucccggcgccgCag.......................     1
G1a_6201526_x1...................................................................gAuucgauucccggcgccggag.......................     1
Gi3_28856543_x1......................cguaguauaacggcuGga........................................................................     1
Gi2_11136551_x1....................................................................guucgaAucccggcgccggag.......................     1
Gi3_28982503_x1...............................................guucCcacccaacagauccggg...........................................     1
Gi5_8971504_x1..................ccggcguagAauaacggcua..........................................................................     1
G1a_6087353_x1...................................................................gguucgauucccCgcgccgga........................     1
Gi3_28495574_x1............................................................agauGcggguucgauucc..................................     1
Gi3_28519129_x1.......................................................................cgauAcccggcgccggagc......................     1
Gi1_4371846_x1.................uccggcUuaguauaacggcua..........................................................................     1
Gi1_4257778_x1...................................................................gguucgauucccggcgcAg..........................     1
Gi3_28544578_x1.................uccggcguaguauaacggcuagaaG......................................................................     1
Gi5_9066794_x1.................uAcggcguaguauaacggc............................................................................     1
Gi1_4317254_x1...................................................................gguuAgauucccggcgccggagc......................     1
Gi2_11248282_x1.............................................ugUuucucacccaacagauccggguucga......................................     1
Gi2_11119338_x1...............................................guucucacccaacaCauccgg............................................     1
Gi3_28609946_x1.................uccggcguaguauaacggAu...........................................................................     1
Gi5_8848803_x1....................................................................guGcgauucccggcgccggag.......................     1
Gi3_28931829_x1........................................................caacagauccUgguucgauuccc.................................     1
Gi3_28851294_x1....................................................caccUaacagauccggguuc........................................     1
Gi1_4217338_x1.................................................ucucacUcaacagauccgg............................................     1
Gi2_11214536_x1.................ucGggcguaguauaacggc............................................................................     1
Gi5_8837121_x1..............................................gNuucucacccaacagauccg.............................................     1
Gi3_28872324_x1...............................................................ucUggguucgauucccggcgccgga........................     1
Gi1_4203859_x1................................................uuGucacccaacagauccgg............................................     1
Gi3_28529579_x1.....................................................................uucgauuAccggcgccggagc......................     1
Gi3_28732519_x1............................................uugguucucacccCacagaucc..............................................     1
Gi5_8884803_x1....................................................................guucgauucccggcgccggagcAuuucc................     1
Gi5_9023466_x1...................................................................gguucgauuccAggcgccgg.........................     1
Gi3_28686517_x1..................................................cucacccaacagauccUg............................................     1
Gi1_4211070_x1....................ggcguaguauaacggcCa..........................................................................     1
Gi1_4248954_x1...................................................ucacccUacagauccgggu..........................................     1
Gi2_11045741_x1....................................................................guucgauucccggcgccgAa........................     1
Gi1_4188947_x1.................uccggAguaguauaacggcuaga........................................................................     1
Gi3_28862388_x1.................uccggcguaguauaacggcuagaaugguuggu...............................................................     0
```

```
Gi5_8933169_x1....................................................cacccGacagauccggguucga......................................      1
Gi2_11171955_x1.................uccggcguaguauGacggc............................................................................      1
Gi1_4340501_x1...................................................................gguNcgauucccggcgccg..........................      1
Gi3_28534135_x1................................................uucucacccaacagauccU.............................................      1
Gi1_4274271_x1.....................................................................uucgauucccggcgccggagcgA....................      1
Gi3_28552888_x1..................................................cucacccaacagaucGgg............................................      1
Gi3_28816522_x1.....................................................................uAcgauucccggcgccgg.........................      1
G1a_6110384_x1.................uccggcguaguGuaacggcuagaau......................................................................      1
Gi2_11021809_x1.................uccggcguaguauaacggcuaUaa.......................................................................      1
Gi3_28391138_x1.................uccggcguaguaAaacggcuag.........................................................................      1
Gi5_9011630_x1................................................uucucCcccaacagauccgg............................................      1
G1a_6164324_x1.................uccggcguaguauaacggcC...........................................................................      1
Gi2_11271538_x1......................................................................ucgauucAcggcgccgga........................      1
Gi5_8880505_x1......................................................................ucgauucccggUgccgga........................      1
G1a_6235090_x1...................................................................gguucgauucccggcAccgg.........................      1
Gi5_9026887_x1.................................................ucucacccaacagauccU.............................................      1
Gi1_4202829_x1...................................................................gguuGgauucccggcgccggagc......................      1
Gi5_9086464_x1...................................................................Aguucgauucccggcgccggagcgu....................      1
Gi3_28374533_x1.................................................ucucacccaacaCauccg.............................................      1
Gi4_585429_x1 ...................................................................Aguucgauucccggcgccgg.........................      1
Gi3_28400264_x1...............................................guucucacccCacagauccggguucgau.....................................      1
Gi2_11396307_x1...............................................guucucacUcaacagauccgg............................................      1
Gi5_8870938_x1..............................................ggCucucacccaacagauc...............................................      1
Gi5_8945493_x1.......................Auaguauaacggcuagaau......................................................................      1
Gi2_11022110_x1.......................................................................cgauuccUggcgccggagcguuuc.................      1
Gi2_11116924_x1.................uccggcguaguauaacggGuaga........................................................................      1
Gi3_28772100_x1.............................................ugguucucacccaaUagauccg.............................................      1
Gi5_8887625_x1........................................................caacagauccgggCucgauucccggcgccg..........................      1
Gi5_8998405_x1................................................uucucacccaacagauccgggCu.........................................      1
Gi3_28383516_x1...................................................................gguucgauucccCgcgccgg.........................      1
Gi5_9013907_x1.............................................ugguucucacccaacagauccAg............................................      1
Gi1_4371977_x1.................uccggcguNguauaacggcuagaa.......................................................................      1
Gi5_9020413_x1.....................................................acccaacagauccggguucgauucUc.................................      1
Gi3_28945273_x1............................................uugguucucUcccaacagaucc..............................................      1
Gi5_9082802_x1.................................................ucucacccaacGgauccgg............................................      1
Gi1_4203577_x1.................................................ucucaccUaacagauccgg............................................      1
Gi2_11029933_x1.............................................ugguucucacccaacagauGcg.............................................      1
Gi2_11226528_x1............................................................agGuccggguucgauucc..................................      1
Gi3_28419933_x1............................................................agauccggNuucgauucc..................................      1
Gi2_11138380_x1......................................................................ucgauucccggcgcGggagc......................      1
Gi2_11308489_x1.......................................................................cgUuucccggcgccggagcguuuc.................      1
Gi2_11094102_x1................................................uucucacccaacagauccggguuc........................................      0
Gi1_4348404_x1...............................................guucucacccaaGagauccg.............................................      1
Gi5_9007676_x1.................uNcggcguaguauaacgg.............................................................................      1
Gi2_11129664_x1..................................................................ggguucgauucccggcgAcgga........................      1
Gi3_28311531_x1...........................................................Aagauccggguucgauuccc.................................      1
Gi3_28280924_x1....................................................................guucgauucccggcCccggagc......................      1
```

```
Gi5_8952778_x1...................................................................gguucgauucccggcgccUgag.......................      1
Gi2_11311014_x1.....................................................................uucgauucccggcgccUgag.......................      1
Gi3_28630680_x1....................................................................guucgauucccggcgccgUagc......................      1
Gi3_28378571_x1..................................................................ggguucgGuucccggcgccggagc......................      1
Gi5_8936068_x1...............................................uucucacccCacagauccggguu..........................................      1
Gi5_9137971_x1.................uccggcguaCuauaacggcuagaau......................................................................      1
Gi5_8961556_x1.................uccggcguaguauaacCgc............................................................................      1
Gi3_28987564_x1...................................................................gguucgauGcccggcgcc...........................      1
Gi3_28945706_x1...................cggcguaguauaacAgcuagaauggu...................................................................      1
Gi1_4173683_x1..................Ucggcguaguauaacggcuaga........................................................................      1
Gi3_28513443_x1.................uccggcAuaguauaacggcuagaa.......................................................................      1
Gi2_11312496_x1......................................................................ucgauAcccggcgccggag.......................      1
Gi1_4165399_x1....................................................................Nuucgauucccggcgccggagc......................      1
Gi4_594121_x1 ...................................................................gguucgaCucccggcgccggagc......................      1
Gi5_8990018_x1....................ggcguagCauaacggcuagaau......................................................................      1
Gi3_28407437_x1...............................................guucucacccaacagauccgggC..........................................      1
G1a_6096632_x1....................................................................Uuucgauucccggcgccggag.......................      1
Gi1_4280345_x1..................ccggcguaguCuaacggcua..........................................................................      1
Gi1_4380338_x1....................................................................guucgaGucccggcgccggagc......................      1
G1a_6195582_x1.................uccggcguaguauaacggcuaAaau......................................................................      1
Gi1_4371992_x1.................uccggcguaguauGacggcu...........................................................................      1
Gi3_28773250_x1.................uccggcguaguauUacggcuagaa.......................................................................      1
Gi5_9030840_x1........................................................................gauucccggcgccggagA......................      1
Gi1_4205593_x1...............................................guucucaAccaacagauccg.............................................      1
Gi5_8879089_x1..............................................gguucucacccaCcagauccgg............................................      1
Gi1_4278280_x1..................................................cucacccaacagauccggguucgauucA..................................      1
Gi1_4359562_x1........................................................caCcagauccggguucgauucccggc..............................      1
Gi1_4338588_x1...........................................................cagauccggguucgauuccc.................................      0
G1a_6130507_x1...................................................................gguucgauucccggcAccg..........................      1
Gi3_28284425_x1..........................................................acagauccggguucgaCucccggcgccgg.........................      1
Gi3_28403535_x1............................................................aUauccggguucgauucccggcgccgg.........................      1
Gi2_11021237_x1..................ccggcguGguauaacggcua..........................................................................      1
Gi2_11331587_x1....................................................................guNcgauucccggcgccgg.........................      1
Gi2_11328523_x1...........................................................cagauccgNguucgauuccc.................................      1
Gi3_28700299_x1.................uccggcguaguauaacgUcuag.........................................................................      1
Gi5_8878709_x1..................................................cucacccaacagaucccCg...........................................      1
Gi2_11046574_x1...................................................................gguucgauGcccggcgcc...........................      1
Gi2_11265027_x1.....................................agaaugguuAguucucacc........................................................      1
Gi5_9056703_x1............................................uuggGucucacccaacagauccg.............................................      1
Gi5_9045931_x1..............................................gguucucacccaacagCuccgg............................................      1
Gi1_4349308_x1...................cggcguaguauaacggcC...........................................................................      1
Gi5_8970648_x1.................Cccggcguaguauaacggcu...........................................................................      1
Gi2_11203524_x1.............................................uggCucucacccaacagaucc..............................................      1
Gi2_11177370_x1................uccNgcguaguauaacggc.............................................................................      1
Gi1_4240077_x1...........................................................cagauccgNguucgauucccggcgcc...........................      1
Gi4_587679_x1 ........................................................................gauucccggcgccggagcC.....................      1
Gi2_11393693_x1...............................................guucucacccaacaUauccg.............................................      1
```

```
Gi1_4243940_x1..................ccggcguaguauaacggUua..........................................................................     1
Gi3_28269161_x1.......................................................ccaacagauccggguucgCuuccc.................................     1
Gi1_4307504_x1.....................................................................Aucgauucccggcgccgg.........................     1
Gi5_9044436_x1.................................................ucucacccaacagauccggguu.........................................     0
Gi1_4367436_x1.................................................Acucacccaacagauccgg............................................     1
Gi3_28845316_x1.....................................................acccaGcagauccggguucgauuccc.................................     1
Gi5_8907434_x1.................................................ucucacccaCcagauccgg............................................     1
Gi1_4268271_x1..............................................gguucucacAcaacagauccgg............................................     1
Gi2_11193724_x1........................................................................gauuccAggcgccggagc......................     1
Gi1_4365183_x1..................................................cucacccaacagauccggguucgauuU...................................     1
Gi3_28433959_x1..................ccggcgCaguauaacggcuaga........................................................................     1
Gi3_28609483_x1......................................................................ucgauucccggcgccggUgc......................     1
Gi5_9102312_x1................................................Cucucacccaacagauccggguu.........................................     1
Gi3_28311232_x1..................ccUgcguaguauaacggc............................................................................     1
Gi2_11299578_x1...................cggcguagNauaacggcuagaau......................................................................     1
Gi3_28555814_x1.....................................................................uucgauucUcggcgccggagc......................     1
Gi5_8923123_x1..............................................gUuucucacccaacagauccggguu.........................................     1
Gi3_28764885_x1.............................................ugguucucacccaacagauA...............................................     1
Gi1_4361395_x1.....................................................................uucgauucccggcUccgg.........................     1
Gi3_28755984_x1..................................................cucacccaacagaucAgg............................................     1
Gi3_28440000_x1.............................................ugguucucacccaacagauUcg.............................................     1
G1a_6330986_x1....................................................................guucgauucccCgcgccg..........................     1
Gi5_9110155_x1...................................................................gguucgauucUcggcgccggag.......................     1
Gi2_11077411_x1..................................................cAcacccaacagauccgg............................................     1
Gi5_9017239_x1....................................................................guucUauucccggcgccggagcguuucc................     1
Gi3_28682632_x1..................................................cucacccaacagauccCg............................................     1
Gi1_4295070_x1........................................................caacagauccggguucgauuccUggc..............................     1
Gi1_4217533_x1...................................................................gguCcgauucccggcgcc...........................     1
Gi5_8848841_x1..............................................gguucucacccaacagaCccgg............................................     1
Gi5_8834483_x1...................................................................gguucgauucccggGgccggag.......................     1
Gi5_8944459_x1................................................uucucacccaacagauccgC............................................     1
Gi3_28973498_x1......................................................................ucgauuccGggcgccggagc......................     1
Gi1_4310529_x1.................uccggcgAaguauaacggcuagaa.......................................................................     1
Gi5_8873261_x1..................................................cucaUccaacagauccgg............................................     1
Gi5_8897594_x1................................................uucucacccaacagauccgAguu.........................................     1
G1a_6071562_x1...................................................................Aguucgauucccggcgccg..........................     1
G1a_6317276_x1...................................................................gguucgauucccggAgccg..........................     1
Gi5_8918949_x1......................................................................ucgGuucccggcgccggagc......................     1
Gi3_28435967_x1........................................................caacagauccgggGucga......................................     1
Gi5_9069029_x1..................ccggcguaguauCacggcua..........................................................................     1
Gi4_570145_x1 ....................................................................gGucgauucccggcgccgga........................     1
Gi2_11383158_x1....................................................................guucgauucccggcgccgUa........................     1
Gi1_4291873_x1.................uccggcguaguauaacggcuagaauA.....................................................................     1
Gi2_11391649_x1.............................................ugguucucacccaGcagaucc..............................................     1
Gi5_9087774_x1....................ggcguaguauaacggcuGg.........................................................................     1
G1a_6279999_x1....................................................................guucgauucccAgcgccggagc......................     1
Gi1_4164300_x1....................ggcguaguUuaacggcua..........................................................................     1
```

```
Gi1_4175107_x1.............................................ugNuucucacccaacagauccgg............................................      1
Gi3_28446139_x1.................................................................Uggguucgauucccggcgccggagc......................      1
Gi2_11355533_x1...............................................................uccggguucgauAcccggcgccgga........................      1
Gi4_581032_x1 ...................................................................gCuucgauucccggcgccggagc......................      1
Gi4_589372_x1 .................uccAgcguaguauaacggcuag.........................................................................      1
Gi3_28790025_x1.................................................ucucacUcaacagauccg.............................................      1
Gi3_28813576_x1.................ucNggcguaguauaacggcuag.........................................................................      1
Gi3_28351075_x1.............................................ugguAcucacccaacagauccg.............................................      1
Gi1_4328097_x1.................uccggcguaUuauaacggcua..........................................................................      1
Gi1_4295786_x1....................................................................guuAgauucccggcgccgga........................      1
Gi2_11161442_x1................................................uucucacccaacagauccgC............................................      1
Gi2_11074341_x1.............................................Agguucucacccaacagaucc..............................................      1
G1a_6167788_x1.................................................Ccucacccaacagauccg.............................................      1
Gi4_572457_x1 ...................................................................gguucgCuucccggcgccgg.........................      1
Gi3_28348399_x1.............................................ugguucucacccaacagUuccg.............................................      1
Gi2_11397560_x1....................................................................guucgauucccggcgGcgga........................      1
Gi5_8989993_x1.............................................Ggguucucacccaacagauccggguucga......................................      1
G1a_6296102_x1...................................................................gguAcgauucccggcgccggagc......................      1
Gi3_28870627_x1....................................................................guucCauucccggcgccggagc......................      1
Gi3_28505496_x1........................................................Uaacagauccggguucga......................................      1
Gi4_568121_x1 ............................................uugguucucaccUaacaga.................................................      1
G1a_6331626_x1.................uccggcguaguaCaacggcuag.........................................................................      1
Gi1_4376379_x1................................................uucucacccaacaCauccg.............................................      1
Gi5_9068058_x1.......................................................................cgauucccCgcgccggagcguuuc.................      1
Gi5_9122889_x1...................................................................gguGcgauucccggcgccggagc......................      1
Gi2_11340649_x1................................................uucucacccaacGgauccgg............................................      1
G1a_6195412_x1.................uccggcguaguauaacggcuagaGu......................................................................      1
Gi3_28727938_x1............................................uCgguucucacccaacagaucc..............................................      1
Gi3_28502031_x1......................................................................ucgauucccggcgccggGg.......................      1
Gi3_28441267_x1.....................................................................uucgauucccAgcgccgg.........................      1
Gi3_28538696_x1.............................................................gauccggguucgauAccc.................................      1
G1a_6104029_x1...................................................................gguucgauucccCgcgccggagc......................      1
Gi1_4174351_x1.................uccggcguaAuauaacggcuaga........................................................................      1
Gi2_11384375_x1.................uccggcguaguCuaacggcuagaau......................................................................      1
Gi2_11022829_x1......................................................cccaCcagauccggguuc........................................      1
Gi3_28546804_x1...............................................guucucaccUaacagauccggg...........................................      1
Gi2_11275583_x1...........................................................cagauccggguucgauucccggcgccggCg.......................      1
Gi3_28521686_x1.............................................uggNucucacccaacagauccg.............................................      1
Gi1_4214623_x1.......................................................................cgauucccggcgccggagcguuuc.................      0
Gi1_4193508_x1........................................................caacagauccggguucgauucUcggc..............................      1
Gi3_28809496_x1.......................................................................cgauucccAgcgccggagc......................      1
Gi3_28900399_x1..............................................................auccggguucgaGucccggcgccgga........................      1
Gi2_11372907_x1.............................................ugguucucCcccaacagauccg.............................................      1
Gi4_610398_x1 ..................................................cucacccaacagauccggguCcg.......................................      1
Gi3_28949480_x1.................uccggGguaguauaacggcua..........................................................................      1
Gi1_4199363_x1..............................................gguucucacccaacUgauccg.............................................      1
Gi1_4181418_x1.................uccggcguaguauaacggcCagaau......................................................................      1
```

```
Gi5_8955198_x1..............................................gguuAucacccaacagauccg.............................................     1
Gi3_28714124_x1....................................................cacccaacagauccgAguuc........................................     1
Gi4_591759_x1 ....................................................................guucgauucccggcgccgC.........................     1
Gi2_11009191_x1......................................................cccaacagNuccggguuc........................................     1
Gi1_4364503_x1.................uccNgcguaguauaacggcuag.........................................................................     1
Gi2_11218470_x1..................................................................ggguucgauucccggcgccggagcgA....................     1
Gi2_11318228_x1.................uccggcguaguauaaAgg.............................................................................     1
Gi3_28767597_x1........................................................................gauucccggcgUcggagc......................     1
Gi1_4225951_x1...................................................ucacAcaacagauccgggu..........................................     1
Gi5_9044106_x1....................Agcguaguauaacggcuagaa.......................................................................     1
Gi5_9072391_x1.............................................ugguucucacccaaUagauccg.............................................     1
Gi3_28682926_x1.................uccUgcguaguauaacggcuagaau......................................................................     1
Gi2_11138565_x1.................uccAgcguaguauaacggcuagaa.......................................................................     1
Gi3_28920230_x1..................................................cGcacccaacagauccggguucgauucc..................................     1
Gi5_9080958_x1...................................................................gCuucgauucccggcgccggagcgu....................     1
Gi2_11161412_x1..................................................cucacccaacagauccAg............................................     1
Gi5_9100593_x1................................................uucucacccaaUagauccg.............................................     1
Gi3_28503864_x1.............................................ugguucucacccaCcagauc...............................................     1
G1a_6108627_x1.............................................Agguucucacccaacagauccg.............................................     1
Gi5_9026152_x1...............................................guucucaGccaacagauccgg............................................     1
Gi1_4201748_x1.....................................................................uucgauucccggcgccggagcA.....................     1
Gi4_578696_x1 .................uccggcguaguGuaacggcua..........................................................................     1
Gi2_11314715_x1................................................uuNucacccaacagauccgg............................................     1
Gi3_28492309_x1.................uccggcCuaguauaacggcuagaau......................................................................     1
G1a_6020147_x1...................................................................gguucgauuccAggcgccggag.......................     1
Gi3_28530015_x1.......................................................ccaacagauccggguucgauucccggcgccggagc......................     0
Gi5_9083523_x1..............................................ggNucucacccaacagauccg.............................................     1
Gi3_28438328_x1......................................................................ucgauucAcggcgccggagc......................     1
G1a_6258115_x1....................................................................Auucgauucccggcgccgg.........................     1
Gi5_9020042_x1..................ccggcguGguauaacggcuagaau......................................................................     1
Gi3_28598908_x1...............................................guucucacccaCcagauccgggu..........................................     1
Gi5_9112221_x1...................................................................gguucgauucccggcgccggCgcguu...................     1
Gi2_11398309_x1..................................................cucacccaacagaCccgg............................................     1
Gi2_11006973_x1.............................................ugguAcucacccaacagauccg.............................................     1
Gi5_9095907_x1....................................................................guNcgauucccggcgccgg.........................     1
Gi3_28586589_x1...............................................................uccggguucgauucccggcgccggagc......................     0
Gi1_4358148_x1................Auccggcguaguauaacggc............................................................................     1
Gi3_28392848_x1...................................................................gguucgauucccggcgccggagcUu....................     1
Gi5_8932518_x1..................................................................ggguucgauuUccggcgccggagc......................     1
Gi5_9115682_x1..............................................gguucuGacccaacagauccgg............................................     1
Gi5_9103529_x1....................................................................guucgauucGcggcgccgg.........................     1
Gi3_28395763_x1........................................................caacagaCccggguucga......................................     1
Gi1_4280106_x1..................ccggcguaguauaaUggcuagaa.......................................................................     1
Gi3_28525201_x1............................................uugguucucacccaacaAaucc..............................................     1
Gi3_28471226_x1.................................................................cggAuucgauucccggcgccgga........................     1
Gi5_8901742_x1...................cggcguaguauaCcggcuaga........................................................................     1
Gi2_11178729_x1................................................uucucacccaacagauccggguuUg.......................................     1
```

```
Gi1_4166191_x1..................................................cucacccaacagauccggguucgG......................................      1
Gi5_8886831_x1...................................................................gguucgauuccUggcgccggagcguu...................      1
Gi3_28975968_x1..............................................................auccggguucAauucccggcgccgga........................      1
Gi3_28614583_x1.................uccggcguaguauaCcggcuagaa.......................................................................      1
Gi2_11273164_x1.....................................................................uucgauucccggcgccgC.........................      1
Gi2_11311702_x1....................................................................guucgauucccgUcgccgga........................      1
Gi1_4358312_x1...................................................ucacccCacagauccgggu..........................................      1
Gi2_11390656_x1.............................................ugguucCcacccaacagaucc..............................................      1
Gi2_11095496_x1.................uccUgcguaguauaacggcuagaa.......................................................................      1
Gi1_4323736_x1.......................................................................cgauucccggcgccggagcguuA..................      1
Gi3_28316339_x1...............................................guucuUacccaacagauccgg............................................      1
Gi5_8834769_x1.....................................................aGccaacagauccggguucgauuccc.................................      1
Gi5_8985418_x1.................uccggcguaguUuaacggcuagaau......................................................................      1
G1a_6162908_x1.................uccggcguaguauCacggcuagaau......................................................................      1
Gi1_4203173_x1..................................................Nucacccaacagauccgg............................................      1
Gi2_11234487_x1......................................................................ucgauucccggcgccgUagc......................      1
Gi5_8929645_x1...............................................guucucacUcaacagauccgg............................................      1
Gi3_28579118_x1......................................................................ucgauucccAgcgccggagc......................      1
Gi5_8902325_x1..................................................................Agguucgauucccggcgccggag.......................      1
Gi5_9079622_x1......................................................cccaacagauccgggCuc........................................      1
Gi3_28799960_x1............................................uugguucucacccaaUagaucc..............................................      1
Gi5_8892016_x1.............................................ugguucuGacccaacagauccg.............................................      1
Gi3_28549937_x1...................cggcguaguauaacggcuCga........................................................................      1
Gi5_8890773_x1...............................................guucucaAccaacagauccgg............................................      1
Gi2_11024313_x1...................................................................gguucgauucccggcgccggagcguuA..................      1
G1a_6044083_x1....................................................................guucAauucccggcgccggag.......................      1
Gi3_28893665_x1.................................................................cgguucgaCucccggcgccgga.........................      1
Gi5_8994404_x1....................................................................Cuucgauucccggcgccggag.......................      1
Gi5_8857226_x1....................................................................guucgauucccggcgccggagcgu....................      0
Gi1_4373866_x1....................ggcguaguGuaacggcua..........................................................................      1
Gi1_4324906_x1...........................................................cagauccggguucgCuucccggcgcc...........................      1
Gi1_4272720_x1...................................................................gCuucgauucccggcgccg..........................      1
Gi3_28405141_x1..............................................................auccggguCcgauucccggcgccggagc......................      1
Gi1_4270057_x1.................uccggcguagAauaacggcua..........................................................................      1
Gi2_11100270_x1...........................................................cagaGccggguucgauucccggcgccggag.......................      1
Gi1_4195659_x1..............................................................Uuccggguucgauucccggcgccgga........................      1
Gi2_11059842_x1...................................................................gguucgauucccggcgccgCa........................      1
Gi3_28627834_x1.....................................................................uNcgauucccggcgccggagc......................      1
Gi5_9119065_x1.....................................................acccaacagauccgggCucgauucc..................................      1
Gi2_11181027_x1.................uccggcguaguUuaacggcua..........................................................................      1
G1a_6220951_x1.....................................................................uAcgauucccggcgccggagc......................      1
Gi3_28335349_x1..........................................................acagauccNgguucgauucccggcgccgga........................      1
Gi2_11055804_x1.........................................................aacagauccggguucgauCcccggcgccg..........................      1
Gi5_8902008_x1...................................................ucacUcaacagauccggguu.........................................      1
Gi4_612650_x1 ....................................................................guucgauucccggcgUcgga........................      1
Gi2_11180185_x1......................................................Gccaacagauccggguuc........................................      1
Gi5_8979466_x1.......................guaguauaacgAcuagaau......................................................................      1
```

```
G1a_6322399_x1....................................................................gguucgGuucccggcgccg...........................	1
Gi5_9040313_x1..................................................ucucacccaCcagauccg..............................................	1
Gi2_11096192_x1..................uccggcguaguauaacggG.............................................................................	1
Gi1_4210347_x1..............................................uggNucucacccaacagauccgg.............................................	1
Gi2_11276217_x1..................uccggcguaguauaacggcuagaauggGu...................................................................	1
Gi3_28328912_x1...................ccggcguaguauaaUggcuag..........................................................................	1
G1a_6115971_x1.....................................................................Auucgauucccggcgccgga.........................	1
G1a_6239905_x1....................................................................gguucgauuccUggcgccggagc.......................	1
Gi2_11357651_x1.................................................uucucaccUaacagauccgg.............................................	1
G1a_6020043_x1.....................................................................guucgauCcccggcgccggagc.......................	1
Gi2_11294474_x1......................................................................uucgauucccggcAccgg..........................	1
Gi3_28960986_x1.......................................................................ucgauucAcggcgccggag........................	1
Gi1_4377282_x1....................................................ucacccaacagauccggAu...........................................	1
Gi2_11084435_x1..................ucAggcguaguauaacggcua...........................................................................	1
Gi5_8934047_x1.............................................uugguCcucacccaacagauccg..............................................	1
Gi5_9064252_x1..................uccggcguGguauaacggcuagaa........................................................................	1
Gi2_11095490_x1................................................guucAcacccaacagauccgg.............................................	1
Gi1_4324529_x1....................................................................gguucAauucccggcgccggagc.......................	1
Gi1_4335481_x1....................................................................gguNcgauucccggcgccgg..........................	1
Gi1_4178239_x1................................................guucucacccaacagauUcgg.............................................	1
Gi5_9075623_x1.....................................................................guucgaCucccggcgccggagcgu.....................	1
Gi2_11037821_x1......................................................................uucAauucccggcgccggag........................	1
Gi1_4237845_x1...................................................cucacccaacagauccAgguucgauucc...................................	1
Gi2_11037583_x1......................................Ggaaugguugguucucacc.........................................................	1
Gi2_11239010_x1.....................................................................guucgauucccCgcgccgga.........................	1
Gi2_11114223_x1.............................................................Ggauccggguucgauucc...................................	1
G1a_6272640_x1.....................................................................guCcgauucccggcgccggagc.......................	1
Gi5_9129030_x1..................uccggcguNguauaacggcuagaaugg.....................................................................	1
Gi1_4376683_x1....................................................................gguucgauuUccggcgccg...........................	1
Gi5_9038197_x1....................................................................gguucUauucccggcgcc............................	1
Gi1_4206593_x1................................................guucucacccaacagaCccgg.............................................	1
Gi2_11248524_x1...............................................gguucucacGcaacagauccgg.............................................	1
Gi1_4303009_x1................................................guucucacccaacagaAccgg.............................................	1
Gi1_4272790_x1..................uccggcguaguauaaAggcu............................................................................	1
Gi5_8947288_x1....................................................................gguucgaAucccggcgccggagc.......................	1
Gi2_11070767_x1...............................................gguucuAacccaacagauccg..............................................	1
Gi2_11066010_x1......................................................................uucgauucccggcgAcggagc.......................	1
Gi3_28284791_x1..............................................................gauccgAguucgauuccc..................................	1
Gi1_4185010_x1..................................................uUucacccaacagauccgg.............................................	1
Gi1_4202196_x1........................................................................cgauucccggcgccggag........................	0
Gi2_11218230_x1....................................................................gguucgauucccggAgccgg..........................	1
Gi5_9035518_x1...................................................................ggguucgauucccggcgcc............................	0
Gi1_4225206_x1................................................guuNucacccaacagauccg..............................................	1
Gi5_8993877_x1....................cggcguaguauaacggcAaga.........................................................................	1
Gi2_11168067_x1.....................................................................gNucgauucccggcgccggagc.......................	1
Gi5_9000256_x1........................................................................cgauucccggcgccggagcguu....................	0
Gi3_28692859_x1................................................guucucacccaacGgauccgggu...........................................	1
```

```
Gi3_28520573_x1................Cuccggcguaguauaacggcuaga........................................................................     1
Gi2_11008204_x1................................................Aucucacccaacagauccg.............................................     1
Gi3_28894219_x1.............................................ugguucucacccaUcagauccg.............................................     1
Gi2_11026771_x1.....................gcguaguauaacggcuaga........................................................................     0
Gi5_8910802_x1.................uAcggcguaguauaacgg.............................................................................     1
Gi3_28277343_x1................................................uucucUcccaacagauccggguu.........................................     1
Gi4_568345_x1 .................uccggcguaguGuaacggc............................................................................     1
Gi2_11135721_x1.....................................agaaugguugguucucaUc........................................................     1
Gi5_8972409_x1...................cggcguaguauaacAgcuagaau......................................................................     1
Gi5_9000909_x1....................................................................guucgauucccggcgccggaUc......................     1
Gi5_8835427_x1.............................................ugguuAucacccaacagauccg.............................................     1
Gi5_8892777_x1................................................ucucacccaacagaucAg..............................................     1
Gi5_9053379_x1...............................................guucucacccaacagaucNg.............................................     1
Gi2_11294708_x1..................................................cCcacccaacagauccgg............................................     1
Gi2_11372612_x1.............................................................gauccggguucgauuccc.................................     0
Gi3_28652680_x1.......................................................................cgauucccggcgccggCg.......................     1
Gi3_28326683_x1.................uccggcguaguauaaAggcuag.........................................................................     1
Gi2_11350563_x1.................ucGggcguaguauaacggcuagaau......................................................................     1
Gi5_9116846_x1..........................................................................................guuucccgccuuccuucgCcu.     1
Gi3_28430870_x1..................ccggcAuaguauaacggc............................................................................     1
Gi4_571028_x1 .................uccggcguaguauaacAgcuaga........................................................................     1
Gi5_9006296_x1.................uccggcguaguauaacggcuagaauggA...................................................................     1
Gi1_4287339_x1..................ccNgcguaguauaacggc............................................................................     1
Gi3_28419842_x1....................ggcguaguauaacggcuagaaC......................................................................     1
Gi3_28403797_x1............................................................agauccggguucgauuGc..................................     1
Gi3_28894200_x1..................................................cucacccaGcagauccgg............................................     1
Gi3_28951653_x1............................................................agauccAgguucgauuccc.................................     1
G1a_6031264_x1....................................................................guucgauucccggcgcAggagc......................     1
Gi2_11376939_x1...................................................................ggNucgauucccggcgccgga........................     1
Gi5_9014789_x1...............................................guucucacccaacagaucAgg............................................     1
Gi3_28283115_x1............................................................aAauccggguucgauucc..................................     1
G1a_6104292_x1...................................................................gguucgauuccUggcgcc...........................     1
Gi5_9042488_x1............................................uugguucCcacccaacagauccg.............................................     1
Gi5_8916156_x1....................................................................guucgauuNccggcgccggag.......................     1
Gi5_9045519_x1.........................................................aacagauccgggGucgauucccggcgccggagc......................     1
Gi1_4374766_x1.................uccUgcguaguauaacgg.............................................................................     1
Gi3_28628330_x1...............................................................uccggguucgauucccggcgccggG........................     1
G1a_6245004_x1.................uccggcguaguauaacggUuag.........................................................................     1
Gi3_28633401_x1..................................................................ggguucgauucccggGgccgga........................     1
G1a_6029100_x1...................................................................gguucgauucccAgcgccg..........................     1
Gi2_11295613_x1....................................................................guuGgauucccggcgccggagc......................     1
Gi1_4267799_x1..............................................gguucucacccaacagCuccgg............................................     1
Gi3_28827616_x1............................................................agauccggguAcgauuccc.................................     1
Gi1_4328858_x1................................................uGcucacccaacagauccggguucgau.....................................     1
Gi5_9118190_x1.............................................ugguucucNcccaacagauccgg............................................     1
Gi1_4262903_x1....................................................................guucgCuucccggcgccggagc......................     1
Gi5_8895229_x1.............................................ugguucucacccaacagauccA.............................................     1
```

```
Gi1_4187177_x1..................ccggcguaguauaacggcuGgaau......................................................................     1
Gi3_28971467_x1......................................................................ucgauucccggcgcccCga.......................     1
Gi4_600522_x1 ...............................................Cuucucacccaacagauccg.............................................     1
Gi5_9026656_x1.................uccggcguaguauaacgAcuagaa.......................................................................     1
G1a_6275200_x1...............................................guucucacccaCcagauccg.............................................     1
Gi4_584701_x1 ..................ccggcguaguauaacggcuag.........................................................................     0
Gi1_4323896_x1..............................................................auccggAuucgauucccggcgccgg.........................     1
G1a_6055536_x1...................................................................gguucgauucccgAcgcc...........................     1
Gi2_11086499_x1.................................................................cggguucgaGucccggcgccgga........................     1
Gi2_11327666_x1..............................................gguucucacccaacagaucc..............................................     0
Gi5_8975422_x1....................................................................guucgaCucccggcgccggagcguu...................     1
Gi5_8884338_x1...................cggcguagCauaacggcuag.........................................................................     1
Gi1_4329162_x1..............................................................Guccggguucgauucccggcgccgga........................     1
Gi1_4165615_x1.............................................ugguucucacccaacagauAcg.............................................     1
Gi3_28572412_x1....................................................................guucgauuGccggcgccgga........................     1
G1a_6183397_x1....................................................................guucgauucccggcgccggagcU.....................     1
Gi3_28675635_x1........................................................caacagauccAgguucga......................................     1
Gi5_8991978_x1..............................................gguucAcacccaacagauccgg............................................     1
Gi5_9095572_x1................................................uucucacccaacagaucAgg............................................     1
Gi1_4231488_x1..............................................gguucucacccaacagauUcg.............................................     1
Gi5_8924373_x1.................Gccggcguaguauaacggcuagaaug.....................................................................     1
Gi3_28868449_x1..............................................................auccgggCucgauucccggcgccgga........................     1
Gi5_9027007_x1.................uccggcguaguauaacCgcuaga........................................................................     1
Gi5_9061390_x1.................uAcggcguaguauaacggcuagaau......................................................................     1
Gi5_9041570_x1.................uccNgcguaguauaacggcuaga........................................................................     1
Gi2_11174349_x1..............................................Uguucucacccaacagauc...............................................     1
Gi1_4371490_x1..............................................gguucuUacccaacagauccgg............................................     1
Gi5_8905012_x1.................uccggcguaguauaacggcAaga........................................................................     1
Gi3_28935120_x1................guUcggcguaguauaacggcuaga........................................................................     1
G1a_6314845_x1...............................................guucucacccaacagGuccg.............................................     1
Gi2_11370999_x1................................................uucucCcccaacagauccgg............................................     1
Gi3_28350522_x1............................................................agauccggguucUauucccggcgccgg.........................     1
Gi4_582858_x1 ...................................................................ggAucgauucccggcgccgg.........................     1
Gi5_9086160_x1.....................................................................uucgauucccggcgccggagcAu....................     1
G1a_6189935_x1................................................uucucacccGacagauccg.............................................     1
Gi2_11271890_x1......................................................................ucgauucccggcgccgAa........................     1
Gi5_8950064_x1...................................................................gguuGgauucccggcgcc...........................     1
Gi3_28721884_x1....................................................cacccaacagauccAgguuc........................................     1
Gi3_28817395_x1..................ccggcguaguauCacggcuaga........................................................................     1
Gi3_28331776_x1......................................................................ucgauucccggcgGcggag.......................     1
Gi5_8917411_x1...............................................guucucacccaaGagauccgg............................................     1
Gi1_4202183_x1......................................................................ucgauucccAgcgccggagc......................     1
Gi5_8866757_x1.................................................ucucacccaacagauccggg...........................................     0
Gi1_4248204_x1.................uccggAguaguauaacggcuagaa.......................................................................     1
Gi3_28367049_x1........................................................caacagauccgAguucga......................................     1
Gi1_4203726_x1..................ccggcguaguauaacggcuagaU.......................................................................     1
Gi3_28646600_x1.....................................................................uucgauAcccggcgccgga........................     1
```

```
Gi5_8854179_x1.................uccggcguaCuauaacggcuag.........................................................................    1
Gi2_11227077_x1....................................................................guucgauucccggcgcGg..........................    1
Gi3_28371487_x1................................................uucucacccaacaUauccg.............................................    1
Gi5_9043316_x1..................ccggcguaguauaUcggcuag.........................................................................    1
Gi1_4290332_x1....................................................................guucgauucccggcgccggaC.......................    1
G1a_6336195_x1....................................................................gGucgauucccggcgccgga........................    1
Gi3_28320600_x1................................................uucucaUccaacagauccgg............................................    1
Gi2_11144303_x1....................................................................guucCauucccggcgccggag.......................    1
Gi4_608663_x1 ...................................................................gguucgauucccggcgccgA.........................    1
Gi2_11055791_x1...................................................................gUuucgauucccggcgccggagcguuu..................    1
Gi3_28605773_x1..................................................................gguGcgauucccggcgccggag........................    1
Gi3_28608683_x1..................................................................Cgguucgauucccggcgccg..........................    1
Gi5_8985589_x1...................cggcgCaguauaacggcuaga........................................................................    1
Gi1_4305945_x1.................uccggcguaguauGacggcuaga........................................................................    1
G1a_6026247_x1....................................................................guucUauucccggcgccgg.........................    1
Gi3_28963819_x1.....................gcguaguauaacAgcuagaa.......................................................................    1
Gi1_4364784_x1.................................................ucucacUcaacagauccg.............................................    1
Gi5_8846416_x1..................ccggcgCaguauaacggcuag.........................................................................    1
Gi2_11365591_x1......................................................................ucgauucccggcgccggGg.......................    1
Gi1_4191855_x1..................................................Gucacccaacagauccggguucgauucc..................................    1
Gi5_9102028_x1.............................................ugguucucNcccaacagauccgggu..........................................    1
Gi3_28379045_x1..................ccggcguaguauaacggcCagaau......................................................................    1
Gi5_9134259_x1...............................................guucucacccaacagaGccgg............................................    1
Gi2_11000590_x1........................................................................gauuAccggcgccggagc......................    1
Gi5_9097006_x1...................................................................gguucgUuucccggcgccgga........................    1
Gi2_11394100_x1...................cggcguaguauaacggcuaAaau......................................................................    1
G1a_6151198_x1...................................................................gguucgauuUccggcgccggagc......................    1
Gi2_11267230_x1...................cggcUuaguauaacggcuag.........................................................................    1
Gi3_28803494_x1.............................................................gauccggguucgauucAc.................................    1
Gi3_28482553_x1...................................................Ccacccaacagauccggguuc........................................    1
Gi3_28681081_x1........................................................caacagauccggguucgGuucccggcgccgg.........................    1
Gi4_583078_x1 .................uccggcguaguauaacggcuaAaa.......................................................................    1
Gi3_28983925_x1............................................................agauccggAuucgauucccggcgccggagc......................    1
Gi2_11082774_x1....................................................................guuGgauucccggcgccggag.......................    1
Gi5_8832448_x1...............................................guucucacccaacagauccAg............................................    1
Gi3_28554672_x1...............................................guucucacccaacagGuccgg............................................    1
Gi3_28519263_x1...........................................................cagauccggguucgauucccggcAccgg.........................    1
Gi1_4190703_x1............................................................agauccggguucgauuAcc.................................    1
Gi5_8855745_x1.............................................ugguucucacccaGcagauccgg............................................    1
Gi4_579019_x1 ....................................................................gGucgauucccggcgccggagc......................    1
Gi3_28782586_x1....................................................................guucgaAucccggcgccggag.......................    1
Gi2_11001856_x1...............................................guucGcacccaacagauccg.............................................    1
Gi3_28654841_x1...............................................guucucacccaCcagauccggg...........................................    1
Gi3_28890204_x1...............................................................ucAggguucgauucccggcgccgga........................    1
Gi2_11037431_x1............................................................agauccggguucgCuuccc.................................    1
Gi5_8913613_x1....................................................cacccaGcagauccggguucga......................................    1
Gi5_8859996_x1................................................uucucacccaacagauccgggAu.........................................    1
```

```
G1a_6058608_x1.................................................ucucacccaacagaucUg..............................................  1
Gi5_8923941_x1..................................................cucacAcaacagauccgg.............................................  1
Gi3_28647113_x1..................................................................ggguucgauucccggcgUcgga.........................  1
G1a_6297142_x1.................ucUggcguaguauaacggcuagaau.......................................................................  1
Gi2_11258334_x1......................................................cccaacagauGcggguuc.........................................  1
Gi5_8923861_x1...............................................guucAcacccaacagauccgg.............................................  1
Gi5_9051740_x1.....................................................................uucgauucccggUgccgg..........................  1
Gi3_28625165_x1..................................................cucacccaacagauUcgg.............................................  1
Gi2_11059354_x1...................................................................gguucgaAucccggcgcc............................  1
Gi1_4321648_x1....................................................cacccaacagauccggguucgCuuccc..................................  1
Gi2_11246227_x1...................................................................gguNcgauucccggcgccg...........................  1
Gi3_28725258_x1..................................................cucacccaNcagauccggguucgauucc...................................  1
Gi5_8878519_x1................................................uucAcacccaacagauccggguu..........................................  1
Gi3_28423118_x1...............................................guucucacGcaacagauccggg............................................  1
Gi5_8862420_x1.............................................uCguucucacccaacagauccgggu...........................................  1
Gi3_28459116_x1.....................................................................uucNauucccggcgccgga.........................  1
Gi3_28328436_x1....................................................cGcccaacagauccggguuc.........................................  1
Gi5_8906643_x1.............................................................gauccggguucgCuucccggcgccgga.........................  1
Gi5_9032219_x1.................uccggcguaguaGaacggc.............................................................................  1
Gi3_28400846_x1...............................................guucucacccGacagauccgg.............................................  1
Gi4_570399_x1 .................uccggcguaguauaGcggc.............................................................................  1
Gi2_11225737_x1.................uccUgcguaguauaacggcuagaau.......................................................................  1
Gi3_28659909_x1.....................................................................uucgauucNcggcgccgg..........................  1
Gi5_9098234_x1.............................................ugguucucacccaacGgauccgg.............................................  1
Gi1_4221503_x1............................................................agauccggguucgauucccggcgccggagc.......................  0
Gi5_8977196_x1..................ccggcguaguauaacUgcua...........................................................................  1
Gi2_11061751_x1..................cGggcguaguauaacggcua...........................................................................  1
Gi5_8958892_x1......................................................................ucgGuucccggcgccggag........................  1
Gi5_8965334_x1...................................................................gguucgauuUccggcgccggagcguu....................  1
Gi3_28606103_x1.......................................................Gcaacagauccggguucgauucccggcgccggagc.......................  1
Gi5_9126998_x1....................ggcguaguGuaacggcuaga.........................................................................  1
Gi3_28809061_x1..............................................Cguucucacccaacagauccgggu...........................................  1
Gi2_11025721_x1...............................................guucucacccaCcagauccgg.............................................  1
Gi4_570229_x1 .................uccgAcguaguauaacggcuaga.........................................................................  1
Gi2_11358108_x1............................................................agauccggguucgGuucc...................................  1
Gi2_11133795_x1........................................................................gauucAcggcgccggagc.......................  1
Gi2_11400291_x1...............................................................uccgggCucgauucccggcgccgga.........................  1
Gi3_28304608_x1.............................................Cgguucucacccaacagauccggguucga.......................................  1
Gi3_28686905_x1.....................................................................uucgauucccggcAccgg..........................  1
Gi2_11077775_x1................................................uucucacccaGcagauccgg.............................................  1
Gi5_9087436_x1.........................................................................auucccggcgccggagcgA.....................  1
Gi5_8938454_x1..............................................gguucucacccaacagCuccggguu..........................................  1
Gi1_4269386_x1.............................................ugguucucacccaacagauccCg.............................................  1
Gi2_11068295_x1...............................................guucucacccaacagauUcg..............................................  1
Gi1_4308627_x1....................................................................guucgauucccggGgccg...........................  1
Gi2_11235221_x1.................Gccggcguaguauaacggcuagaaugguugg.................................................................  1
Gi5_9022843_x1......................................................cccaacagauccAgguuc.........................................  1
```

```
Gi5_8877369_x1....................................................................guucgauucccggcgccgU.........................      1
Gi5_9045446_x1..............................................gguucucacccaacaUauccg.............................................      1
Gi3_28509361_x1....................................................................gCucgauucccggcgccggagcg.....................      1
Gi2_11343980_x1.................uccNgcguaguauaacggcua..........................................................................      1
Gi4_579821_x1 .................uccggcgCaguauaacggcuaga........................................................................      1
Gi5_9047439_x1..................ccggcguaguauaacggcuGgaau......................................................................      1
Gi5_9089016_x1...............................................guucucCcccaacagauccgg............................................      1
Gi5_8916660_x1................................................uucucacccCacagauccgg............................................      1
Gi3_28481643_x1...............................................................Cccggguucgauucccggcgccgga........................      1
Gi5_8940859_x1........................................................................gaGucccggcgccggagc......................      1
G1a_6084214_x1...................................................................gguucgauucccggcgUcggag.......................      1
Gi3_28793977_x1....................ggcguaguauaGcggcuaga........................................................................      1
Gi4_591882_x1 .................uccggUguaguauaacggcuagaau......................................................................      1
Gi2_11236505_x1.............................................ugguucucUcccaacagauccg.............................................      1
Gi3_28514188_x1.................................................ucGcacccaacagauccgg............................................      1
Gi1_4172506_x1.................uccggcAuaguauaacggcuagaa.......................................................................      1
Gi1_4193613_x1...................................................................gguucgauucccggcgccgAagc......................      1
Gi5_8981188_x1....................................................................guucgauuGccggcgccggagc......................      1
Gi5_9011185_x1......................................................................ucgauCcccggcgccggagc......................      1
Gi5_9100445_x1.................................................ucucacccCacagauccg.............................................      1
Gi5_8936367_x1...............................................guucucacGcaacagauccg.............................................      1
Gi5_9021916_x1...............................................guucucCcccaacagauccg.............................................      1
Gi1_4250698_x1...........................................................cagauccggguucgauucccggcgAc...........................      1
Gi3_28770490_x1....................................................................guucgauucccggcgcGggag.......................      1
Gi2_11154898_x1...................cggcguGguauaacggcuaga........................................................................      1
Gi5_8916664_x1....................................................cacccaacagauccAgguucga......................................      1
G1a_6053965_x1...................................................................gguucgGuucccggcgccgg.........................      1
Gi4_593467_x1 .................uccggcguaguauaacggcuagaG.......................................................................      1
Gi3_28525479_x1.........................................................aGcagauccggguucgauucc..................................      1
Gi3_28699645_x1....................Agcguaguauaacggcuaga........................................................................      1
Gi2_11054287_x1...................................................................gguucgauucccgUcgccg..........................      1
Gi2_11159583_x1.................uccggcguaguauaacgCc............................................................................      1
Gi1_4297345_x1.....................................................................uucgauucccggcgccggGgc......................      1
Gi4_597091_x1 ................................................uGcucacccaacagauccgg............................................      1
Gi5_8870495_x1...............................................guucucaccGaacagauccgg............................................      1
Gi3_28460388_x1.............................................ugguucucacccaacaAauccg.............................................      1
Gi2_11212311_x1...................................................................gguucgauuccGggcgccggag.......................      1
Gi3_28297674_x1....................................................................guNcgauucccggcgccgg.........................      1
Gi3_28845590_x1..................................................................ggguuUgauucccggcgccg..........................      1
Gi2_11125660_x1.............................................Ngguucucacccaacagaucc..............................................      1
Gi3_28369503_x1..............................................................auccggguucgauucccggcUccgga........................      1
Gi2_11291313_x1...................................................................gguucgauucccggcCccgga........................      1
G1a_6341186_x1.................uccggcguaguauaacggcCagaau......................................................................      1
Gi5_9135834_x1....................................................................guucgauuGccggcgccgga........................      1
Gi1_4205118_x1....................................................................guucgauucccgUcgccggagc......................      1
Gi1_4211252_x1..................................................................gUguucgauucccggcgccgga........................      1
Gi1_4339288_x1.................uccgAcguaguauaacggc............................................................................      1
```

Gi3_28577856_x1...ggNucucacccaacagauccg... 1
Gi3_28561027_x1....................cggcguagCauaacggcuaga.. 1
Gi3_28751554_x1...Acgauucccggcgccggagc...................... 1
Gi5_9129558_x1..gguucgaGucccggcgccgga........................ 1
Gi1_4217158_x1...cucacccaacagauccggguucAa...................................... 1
Gi3_28519252_x1...cucacccaNcagauccggguucgauu.................................... 1
Gi2_11348783_x1..guucucacccaacagCuccg... 1
Gi1_4354556_x1...gguucucaGccaacagauccgg.. 1
Gi5_8862355_x1..guucucacccaacaAauccgg.. 1
Gi3_28955418_x1...gauuAccggcgccggagc...................... 1
Gi2_11360205_x1..cgauucccggcgcUggagcguuu.................. 1
Gi1_4319846_x1..ucucacccaacagaCccg... 1
Gi3_28934251_x1..................uccggcguaguUuaacggcuagaau.. 1
Gi5_9044427_x1..ucucacGcaacagauccgggu.. 1
Gi5_8875925_x1..Cucgauucccggcgccggag....................... 1
Gi5_8911403_x1..................uccggcgGaguauaacggc.. 1
Gi5_8979444_x1..Cucgauucccggcgccgg......................... 1
Gi1_4369953_x1..gguucgauuccAggcgccggagc...................... 1
Gi5_8860021_x1.....................ggcguaguauaaUggcuag... 1
Gi5_8866573_x1..ugguucAcacccaacagauccg... 1
Gi3_28695765_x1..................uccggcguaguauaacgUcua.. 1
Gi1_4368785_x1...uucucacccaacagGuccggguucgau..................................... 1
Gi5_9127215_x1..gguucUauucccggcgccg.......................... 1
Gi2_11039943_x1...guucgauucccUgcgccg.......................... 1
Gi5_9017324_x1...cacccaaUagauccggguucga...................................... 1
Gi5_9068649_x1..gguucgauucGcggcgccggagc...................... 1
Gi5_8985932_x1..................uccggcgCaguauaacggcu... 1
Gi1_4275504_x1..gguucgauucccgAcgcc........................... 1
Gi2_11223709_x1...ucgauuUccggcgccggag....................... 1
Gi2_11311502_x1..uccggguucgCuucccggcgccgga........................ 1
Gi3_28381292_x1..gguucucacccGacagaucc... 1
Gi3_28435059_x1..ugguucucacccaGcagauc... 1
Gi2_11354062_x1..................uccggcguaguauaacgU... 1
Gi1_4174174_x1...caacagUuccggguucgauucccggc.............................. 1
Gi1_4204272_x1..ugguucucacUcaacagauccg... 1
Gi5_8837373_x1...Nuucgauucccggcgccg.......................... 1
Gi5_8846646_x1................aguccggcNuaguauaacggcuagaaugg.. 1
Gi1_4349061_x1...Gcagauccggguucgauuccc................................. 1
Gi3_28664228_x1.....................ggcguaguauaCcggcuaga.. 1
Gi3_28473247_x1..uUcggguucgauucccggcgccg.......................... 1
Gi3_28987072_x1..aaUagauccggguucgauucc.................................. 1
Gi3_28543581_x1...caacGgauccggguucga...................................... 1
Gi5_8850989_x1..gguucgauucccggcgcUggag....................... 1
G1a_6264563_x1..guucucacccaacUgauccg... 1
Gi3_28612011_x1..guucAcacccaacagauccgggu.. 1
Gi3_28325726_x1..................uccggcguaguCuaacggcuagaa... 1
Gi3_28520908_x1...................ccggcguaguauaaUggcuaga.. 1

```
Gi2_11191680_x1...................cggcguaguauaaUggcua...........................................................................    1
Gi3_28709885_x1....................................................................guucgauuGccggcgccggagc.......................    1
Gi3_28652401_x1......................................................................ucgauucccggcgccggagA.......................    1
Gi2_11168016_x1..................ccggcguGguauaacggc.............................................................................    1
Gi3_28557748_x1.................uccggcgGaguauaacggcuag..........................................................................    1
Gi2_11268373_x1......................................................................ucgauucccggcgccggaAc.......................    1
Gi5_8892886_x1.................uccggcguaguauaacggcuagaaAg......................................................................    1
Gi2_11031964_x1...................................................................gguucgauucccgCcgccgg..........................    1
Gi5_9116538_x1.................................................ucucacccaacagauAcg..............................................    1
Gi3_28621345_x1..............................................gguucucacccaacagaucUgggu...........................................    1
Gi1_4327807_x1...................................................ucaNccaacagauccgggu...........................................    1
Gi3_28296806_x1...............................................guAcucacccaacagauccggg............................................    1
Gi3_28963190_x1..................................................cucacccaacGgauccgg.............................................    1
Gi5_8977588_x1................................................uucucacccaacagauccC..............................................    1
Gi3_28632935_x1...................................................ucaccUaacagauccggguuc.........................................    1
Gi3_28970858_x1......................................................................ucgauucccggcgcUgga.........................    1
Gi3_28609217_x1................................................uucucacccaacagauccggguuA.........................................    1
Gi3_28434038_x1..................................................cucacccaacagauccAg.............................................    1
Gi5_8840844_x1.................uccggcguaguauUacggc.............................................................................    1
Gi5_9007798_x1.................................................ucGcacccaacagauccgg.............................................    1
Gi1_4371333_x1.....................................................................uucgauuccUggcgccggagc.......................    1
Gi5_8960424_x1...................................................................gguucgauucAcggcgcc............................    1
Gi5_8878632_x1..................................................cucacccaacaAauccgg.............................................    1
Gi1_4295209_x1...................................................................gguGcgauucccggcgccgg..........................    1
Gi3_28360332_x1.............................................ugguucucacccaacagaucGg..............................................    1
Gi3_28346336_x1...............................................guucucacccaUcagauccgg.............................................    1
Gi3_28763703_x1.................Nccggcguaguauaacggc.............................................................................    1
Gi3_28839168_x1...............................................guucucUcccaacagauccggg............................................    1
Gi3_28766511_x1..................ccggcguaguCuaacggc.............................................................................    1
Gi3_28828962_x1.................uccggUguaguauaacggcu............................................................................    1
Gi3_28398881_x1...................................................ucacccaacagauccggguCc.........................................    1
Gi5_8923742_x1...................cggcgCaguauaacggcuag..........................................................................    1
Gi1_4266472_x1......................................................................ucgaGucccggcgccggagc.......................    1
Gi2_11101619_x1..................ccggcguagCauaacggcua...........................................................................    1
Gi4_596185_x1 ......................................................................ucgauucccggcgccggagcC......................    1
Gi5_8887714_x1.................uccggcguaguauaacUg..............................................................................    1
Gi3_28765865_x1........................................................................gauucccgUcgccggagc.......................    1
Gi5_8983424_x1....................ggcguaguauaacggcGag..........................................................................    1
Gi2_11019638_x1.................uccCgcguaguauaacggcua...........................................................................    1
Gi2_11234424_x1....................ggcguaguauaacAgcuaga.........................................................................    1
Gi2_11085936_x1..................ccggGguaguauaacggcua...........................................................................    1
Gi5_9062397_x1......................................................................ucgauucccggUgccggag........................    1
Gi1_4278122_x1.................uccggcguaguauUacggc.............................................................................    1
Gi1_4218397_x1...............................................................uccggguucgauGcccggcgccgga.........................    1
Gi2_11000175_x1...................................................................gguucUauucccggcgccggag........................    1
Gi1_4390598_x1....................................................................guucgauGcccggcgccgg..........................    1
Gi3_28865077_x1............................................................agauccgggAucgauuccc..................................    1
```

```
Gi5_8851989_x1................................................uucucGcccaacagauccgg............................................ 1
Gi1_4320232_x1.................uccggcguaguauGacggc............................................................................ 1
Gi5_9077938_x1........................................................................gauucccggcgccggagcCu.................... 1
Gi1_4323646_x1....................................................................Nuucgauucccggcgccgga........................ 1
G1a_6037754_x1...................................................................gguucgauucccggcgccgAag....................... 1
Gi3_28362998_x1.................uccCgcguaguauaacggcuagaau...................................................................... 1
Gi5_9092859_x1.................uccggcguaguauaacgCcu........................................................................... 1
G1a_6246781_x1.................uUcggcguaguauaacggcua.......................................................................... 1
Gi2_11172346_x1.................uccggcguaguauaacggcuaUaau...................................................................... 1
Gi1_4324299_x1....................ggcguaguauaacggUua.......................................................................... 1
Gi3_28628149_x1...................................................................gUuucgauucccggcgccggagcg..................... 1
Gi3_28707866_x1..............................................gguucucacccaaUagauccgg............................................ 1
Gi5_8977507_x1..............................................gguucucacccGacagauccggguu......................................... 1
Gi5_8948110_x1....................Agcguaguauaacggcuagaau...................................................................... 1
Gi2_11114347_x1.......................................................................cCauucccggcgccggagcguuu.................. 1
Gi3_28828058_x1.................uccggcguaguauaacgUcuagaa....................................................................... 1
Gi2_11198248_x1................................................uucucacccaaAagauccg............................................. 1
Gi3_28399980_x1....................................................................guucgauucccCgcgccggag....................... 1
Gi5_8953659_x1....................................................................guucgauucccUgcgccgga........................ 1
Gi5_8990706_x1.................................................ucucacccaacUgauccg............................................. 1
Gi1_4179955_x1...................................................................gguucgauucccggcgcUg.......................... 1
Gi2_11197936_x1.............................................ugguucucacccaacagauUc.............................................. 1
Gi5_9124154_x1....................................................................guuAgauucccggcgccgga........................ 1
Gi4_574850_x1 ....................................................................guucgGuucccggcgccgga........................ 1
Gi5_8991770_x1...................cggcguaguauaCcggcuagaau...................................................................... 1
Gi5_8956080_x1...................................................................gguucgauucccggcCccgga........................ 1
Gi5_8857061_x1....................................................................guucgauucccggcgccggCg....................... 1
Gi2_11123658_x1.................uccggcguCguauaacgg............................................................................. 1
Gi3_28772532_x1.................uccggcguaguauaUcggcuagaau...................................................................... 1
G1a_6300659_x1.................uGcggcguaguauaacggc............................................................................ 1
G1a_6006925_x1.............................................ugguucucacccaacagauccA............................................. 1
Gi5_8904963_x1.......................................................................cgauucccggcgccggagcg..................... 0
Gi2_11144500_x1....................................................................guucgauucccggGgccggag....................... 1
Gi4_583265_x1 ....................................................................guucgauucUcggcgccgga........................ 1
Gi2_11199524_x1.................................................................Gggguucgauucccggcgccgga........................ 1
Gi5_8868571_x1....................................................................guuNgauucccggcgccgga........................ 1
Gi3_28872991_x1....................................................................guucgauucccggcUccgga........................ 1
G1a_6333977_x1................................................uCcucacccaacagauccgg............................................ 1
Gi3_28616852_x1...............................................guCcucacccaacagauccgggu.......................................... 1
Gi2_11226612_x1.................uccggcguaguauaacCgcuag......................................................................... 1
Gi5_9100425_x1...................cggcguaguauaacgAcuaga........................................................................ 1
Gi2_11386878_x1....................................................................guucgauucGcggcgccg.......................... 1
Gi5_8907107_x1...................................................................gguuAgauucccggcgccgg......................... 1
Gi5_9029503_x1.................ucGggcguaguauaacggcua.......................................................................... 1
G1a_6345276_x1.............................................ugguucucacccaacagGuccg............................................. 1
Gi3_28906139_x1.................................................ucucacccaacagGuccgg............................................ 1
Gi3_28462880_x1............................................uugguucucacccaacagCucc.............................................. 1
```

```
Gi2_11274935_x1.................uccggcgAaguauaacggcua..........................................................................     1
Gi1_4329538_x1....................................................................guucgauuccAggcgccg..........................     1
Gi2_11368469_x1..............................................gguucucaccAaacagauccg.............................................     1
Gi5_9108440_x1.................uccggcguNguauaacggcu...........................................................................     1
Gi5_9003046_x1....................................................................guucgauucccggcgccUga........................     1
Gi2_11088396_x1.................uccggcgCaguauaacggcuagaa.......................................................................     1
Gi3_28968747_x1.....................................................................uucgauucccggcAccgga........................     1
Gi5_8968009_x1................................................Nucucacccaacagauccgg............................................     1
Gi1_4197538_x1...................cggcguaguauaacggcuagaa.......................................................................     0
Gi3_28980890_x1............................................................agGuccggguucgauuccc.................................     1
Gi2_11195649_x1.................uccggcguaCuauaacgg.............................................................................     1
Gi3_28573501_x1...................................................ucacccaGcagauccggguuc........................................     1
Gi5_8988570_x1.................uccCgcguaguauaacggcuag.........................................................................     1
Gi2_11137772_x1.................uccgCcguaguauaacggcuag.........................................................................     1
Gi2_11349207_x1...................Uggcguaguauaacggcua..........................................................................     1
Gi3_28394896_x1.....................................................................uucgauucccUgcgccgga........................     1
Gi2_10994031_x1...................................................................gguucgauucccggcgcAgg.........................     1
Gi2_11064202_x1.................uccggcguaguauaacgCcuaga........................................................................     1
Gi4_609235_x1 ......................................................................Gcgauucccggcgccggagc......................     1
Gi3_28665053_x1............................................................agauccggguucgauuAcc.................................     1
Gi1_4223722_x1....................ggcAuaguauaacggcuag.........................................................................     1
G1a_6079268_x1...................................................................gguucgauuAccggcgccggagc......................     1
Gi3_28926284_x1.................uccggcguaUuauaacggcuagaau......................................................................     1
Gi1_4225630_x1.................uccggcguaguUuaacggcua..........................................................................     1
Gi3_28709223_x1......................................................................Acgauucccggcgccggag.......................     1
Gi4_569785_x1 ..................................................cCcacccaacagauccggguucg.......................................     1
Gi1_4172732_x1..........................................................Ucagauccggguucgauuccc.................................     1
G1a_6159216_x1...................................................................gguucgauucccAgcgccgga........................     1
Gi5_9012390_x1...................................................................gguucgauucccggcgcUggagcguu...................     1
Gi5_8978051_x1.............................................ugguuUucacccaacagauccgggu..........................................     1
Gi1_4390133_x1...............................................guucucUcccaacagauccg.............................................     1
Gi2_11098528_x1.................uccggcguaguauaacgUcuaga........................................................................     1
Gi1_4333951_x1....................................................................gAucgauucccggcgccg..........................     1
Gi3_28659346_x1...............................................guGcucacccaacagauccggg...........................................     1
Gi3_28334480_x1...............Uguccggcguaguauaac...............................................................................     1
G1a_6021407_x1...................................................................gguucgauucccggcgccggagU......................     1
Gi2_11162569_x1.................uccggcguaguauaaAggc............................................................................     1
G1a_6138379_x1.................uccggcguaguauGacggcuag.........................................................................     1
Gi4_598390_x1 ...................................................................gguucgauucccggGgccgg.........................     1
Gi2_11271529_x1...........................................................cagauccggguucgCuucccggcgccggag.......................     1
Gi1_4373086_x1...........................................guugguucucacccGacagauccgggu..........................................     1
Gi2_11362987_x1...................................................................gguucgauucccCgcgcc...........................     1
Gi3_28845642_x1............................................................agaucAggguucgauucc..................................     1
G1a_6343388_x1...................................................................gguucUauucccggcgccg..........................     1
Gi5_8841715_x1......................................................Accaacagauccggguucgauucc..................................     1
Gi1_4207462_x1.....................................................................uucgauucccggcgccggagcCu....................     1
Gi1_4278303_x1.............................................ugguucucacGcaacagauccgg............................................     1
```

```
Gi2_11036483_x1..............................................gguucucacccaacaCauccg.............................................     1
Gi2_11266359_x1.......................................................................cgauucccggcgccggagcCu....................     1
Gi2_11258655_x1.................uccgAcguaguauaacggcuagaau......................................................................     1
Gi2_11287437_x1.............................................................gauccggguucgauucccggcgccgga........................     0
Gi3_28323193_x1.............................................................Cauccggguucgauccc..................................     1
Gi3_28623379_x1...............................................gGucucacccaacagauccggguucga......................................     1
Gi3_28745510_x1..............................................gguucucacccaacagauccAg............................................     1
Gi5_9027678_x1.....................................................acccaacagauccggAuucgauucc..................................     1
Gi5_8985532_x1................................................uucucacccaacagauccU.............................................     1
Gi5_8957526_x1.........................................................aacagauccggAuucgauucccggcgccggagc......................     1
Gi5_8974330_x1...............................................guucucGcccaacagauccgg............................................     1
Gi3_28369938_x1.............................................................gaucUggguucgauccc..................................     1
Gi1_4380831_x1....................................................................guucCauucccggcgccgga........................     1
Gi3_28671000_x1...................................................................gguucgauucccggcgccggagcgG....................     1
Gi3_28500724_x1...............................................guucucacccaacagauccggA...........................................     1
Gi2_11099575_x1....................................................................guucgauuccAggcgccg..........................     1
Gi3_28647909_x1..............................................Cguucucacccaacagaucc..............................................     1
Gi3_28539468_x1...............................................guucucacGcaacagauccgggu..........................................     1
Gi1_4334700_x1................................................uucucUcccaacagauccggguucgau.....................................     1
Gi5_8851360_x1.............................................ugguucucacccaacagauAcgggu..........................................     1
Gi5_9013177_x1.................uccggcguaguauaacggcuagaaCgguu..................................................................     1
Gi4_575202_x1 .......................................................................cgauucccggcgccggagcC.....................     1
Gi5_9021819_x1.............................................ugguucucacccaacagaucGg.............................................     1
G1a_6355749_x1....................................................................guucgauAcccggcgccggagc......................     1
G1a_6236295_x1.................uccggcguaCuauaacggcuagaa.......................................................................     1
Gi5_9124832_x1...................................................................gguucgauucccggGgccggagc......................     1
Gi1_4262085_x1.................uccggcguaguauaacggcuaC.........................................................................     1
Gi5_8941978_x1...............aguccggcguaguauGacggcuagaaugg....................................................................     1
Gi1_4289002_x1..................................................cucacccaacagaucGgg............................................     1
Gi2_11012929_x1....................................................................guAcgauucccggcgccg..........................     1
Gi5_8871044_x1..............................................gguucucaUccaacagauccgg............................................     1
Gi1_4209480_x1.................uccggcgCaguauaacggcuagaau......................................................................     1
Gi5_8970092_x1.....................................................................uucgauucccggcgcUgg.........................     1
Gi3_28844265_x1..................ccUgcguaguauaacggcuaga........................................................................     1
Gi3_28580361_x1......................................................................ucgauucccggcgccggagcA.....................     1
Gi5_8941816_x1...................................................................gguucUauucccggcgccgg.........................     1
Gi1_4352042_x1.................uccggcguaguauaacggcuagUa.......................................................................     1
Gi2_11090457_x1................................................uucucacUcaacagauccggguucg.......................................     1
Gi3_28864523_x1......................................................................ucgauucccggAgccgga........................     1
Gi5_8867078_x1......................................................cccGacagauccggguuc........................................     1
Gi5_8985862_x1..................ccggcguaguauaacAgcua..........................................................................     1
Gi3_28309903_x1...............................................gAucucacccaacagauccgg............................................     1
Gi3_28339002_x1..................ccggUguaguauaacggcu...........................................................................     1
Gi2_11330095_x1...................................................................gguGcgauucccggcgccgga........................     1
G1a_6320710_x1....................................................................guAcgauucccggcgccgg.........................     1
Gi5_9023441_x1.................uccggcguaguauaacgUcuaga........................................................................     1
Gi1_4195597_x1......................cUuaguauaacggcuagaau......................................................................     1
```

```
Gi2_11281917_x1....................gUcguaguauaacggcuaga........................................................................ 1
Gi2_11002167_x1.............................................ugguucucacAcaacagauccg............................................. 1
Gi5_9098832_x1................................................uucucacccaaUagauccggguuc........................................ 1
Gi3_28621467_x1.................uccggcgAaguauaacggc............................................................................ 1
Gi1_4253110_x1....................................................................guucAauucccggcgccggagc...................... 1
Gi1_4179217_x1............................................................agauccggguCcgauuccc................................. 1
Gi5_8893376_x1...................................................................gguuGgauucccggcgccggag...................... 1
Gi5_9092731_x1..............................................gguucucaccAaacagauc............................................... 1
Gi2_11013224_x1.............................................uggGucucacccaacagauccg............................................. 1
Gi3_28908699_x1.................................................Ncucacccaacagauccgg.............................................. 1
Gi3_28365432_x1.............................................ugguucuAacccaacagauccg............................................. 1
Gi2_11157230_x1.............................................ugguucucacccaacaAauccg............................................. 1
Gi5_8876262_x1.............................................ugguucucacccUacagauccgggu......................................... 1
Gi4_585503_x1 ............................................uugguucucUcccaacaga................................................. 1
Gi3_28507220_x1.................uccggcguaguauaacggcuaUaaugguuggu.............................................................. 1
Gi3_28977093_x1..............................................gguucucacccaacagUuccg............................................. 1
Gi5_9048020_x1...................................................................gguucgauucccgUcgccggagc...................... 1
Gi3_28868060_x1...........................................................cagaGccggguucgauuccc................................. 1
Gi3_28962402_x1......................................................................ucgauucccggcgccggCg...................... 1
Gi1_4231263_x1................................................uuNucacccaacagauccg............................................. 1
Gi1_4244347_x1.......................................................................Uauucccggcgccggagc...................... 1
Gi5_9107706_x1....................................................................guucgauuccGggcgccgga........................ 1
Gi3_28296388_x1...............................................guucuAacccaacagauccg............................................. 1
Gi3_28630962_x1....................................................................Nuucgauucccggcgccgg......................... 1
Gi5_8985112_x1......................................................................ucgauucccggAgccggag....................... 1
G1a_6295680_x1.....................................................................uucgauucccggcgccggagcCu.................... 1
Gi2_11328054_x1.................uccNgcguaguauaacggcuag......................................................................... 1
Gi2_11395991_x1......................................................................ucgauucccggUgccggag...................... 1
Gi5_8968028_x1...................................................................gguucgauuccGggcgccggagc...................... 1
Gi5_9020591_x1..........................................................acagauccgAguucgauucccggcgc............................ 1
Gi5_9079014_x1..................ccggcguaguauaacggUuag......................................................................... 1
G1a_6065574_x1.................uccgUcguaguauaacgg............................................................................. 1
Gi1_4248327_x1.................uccggUguaguauaacggcu........................................................................... 1
Gi3_28577158_x1...............................................Cuucucacccaacagauccgggu.......................................... 1
Gi1_4197881_x1.................uccUgcguaguauaacggcua.......................................................................... 1
Gi5_9071985_x1.................................................ucucacccaacagauGcgggu......................................... 1
Gi1_4186427_x1....................................................................guucAauucccggcgccggag....................... 1
Gi3_28728018_x1..................................................................ggguucgauucAcggcgccggagc..................... 1
G1a_6024901_x1...................................................................gguucgauuAccggcgccggag....................... 1
Gi2_11360589_x1.............................................ugguucucacccaacagaAccg............................................. 1
Gi2_11361035_x1....................................................................guucgauuGccggcgccg.......................... 1
Gi1_4313092_x1...................................................................gguucgauucccAgcgccggag....................... 1
Gi5_8962046_x1.............................................Cgguucucacccaacagauccgggu......................................... 1
Gi5_8977529_x1...................................................................gguucgauuUccggcgcc........................... 1
Gi2_11299054_x1......................................................................ucgauuAccggcgccggagc..................... 1
Gi3_28595032_x1...................cggcguagNauaacggcuagaau...................................................................... 1
Gi3_28748580_x1.......................................................................cgauucccggcgccggagU..................... 1
```

```
Gi5_9011958_x1.........................................................aacagauccggguucgauucccggcgccgg.........................      0
Gi5_8978627_x1..............................................gguucucacccaacagauGcg.............................................      1
Gi5_8864055_x1...................cggcguaguauaacgAcuag.........................................................................      1
Gi5_9111668_x1............................................uugguucucacccaaUagauccg.............................................      1
Gi5_9021917_x1...............................................uucucacccGacagauccggguu..........................................      1
Gi1_4236220_x1.................uccggGguaguauaacggcuag.........................................................................      1
Gi2_11141503_x1................................................uucucacccaacagauUcgg............................................      1
Gi3_28458350_x1....................................................................guucgauucccggcUccggag.......................      1
Gi5_9078060_x1.................uccggcguaguauaacggUu...........................................................................      1
Gi3_28678017_x1..................................................................ggguucgauucccggcgccAgagc......................      1
Gi1_4216481_x1.................uNcggcguaguauaacgg.............................................................................      1
Gi3_28317217_x1..............................................gguucucacccaacagauccC.............................................      1
Gi3_28524515_x1...............................................................uccggguucUauucccggcgccgga........................      1
Gi5_8833405_x1.............................................ugguucucacccaacagaucUgggu..........................................      1
Gi3_28792058_x1....................................................................guucgaAucccggcgccg..........................      1
Gi1_4258694_x1.............................................ugguucucacccaacagUuccg.............................................      1
Gi5_9131644_x1...............aguccggcguaguauaCcggcu...........................................................................      1
Gi3_28366366_x1.................uccggcgGaguauaacggcuagaa.......................................................................      1
Gi2_11361367_x1.................uccUgcguaguauaacggc............................................................................      1
Gi1_4360605_x1.................uccggcguaAuauaacggcuagaau......................................................................      1
Gi5_8911690_x1........................................................caacagauccggguucgauucccggcgccgg.........................      0
G1a_6005304_x1.....................................................................uucgauuccUggcgccggagc......................      1
Gi4_592464_x1 ....................................................................guucgauucccggcgccgA.........................      1
Gi2_11095148_x1....................ggcguaguauaacggcuagaau......................................................................      0
Gi5_8920249_x1...................................................................gguucgauucccggcCccggagc......................      1
Gi1_4374646_x1.............................................ugguucucacccaacagauUcgg............................................      1
Gi5_8997089_x1................................................................ccggguucgGuucccggcgccgga........................      1
Gi5_9114652_x1.................uccggcguUguauaacgg.............................................................................      1
Gi5_8991135_x1..................ccggUguaguauaacggcuaga........................................................................      1
Gi1_4311204_x1..............................................gguAcucacccaacagauccg.............................................      1
Gi5_8908215_x1...................................................................gguucgUuucccggcgccg..........................      1
G1a_6174982_x1...................................................................gguucgauuccccCgcgccggag......................      1
Gi1_4206857_x1.....................................................................uucgauucccggGccgg..........................      1
G1a_6169655_x1.................................................ucucacccaacagauAcg.............................................      1
Gi1_4171949_x1.....................................................................uucgauucccgAcgccgg.........................      1
Gi5_8831624_x1..............................................gguucuAacccaacagauc...............................................      1
Gi1_4352148_x1...................................................................gguucgauCcccggcgccg..........................      1
Gi3_28281105_x1..................................................cucacccaacagauccAgguucgauu....................................      1
Gi5_8935009_x1................................................uucucacccaacagaCccggguuc........................................      1
Gi5_9070424_x1.................uccggcguaguauaacUgc............................................................................      1
Gi5_8865448_x1...............................................................uccggguucgauuAccggc..............................      1
Gi3_28494870_x1..................................................................ggguucgauucccggcgccggGgc......................      1
Gi2_11014586_x1.................uccggcguaAuauaacggcuag.........................................................................      1
Gi1_4337569_x1.................uccggcguagGauaacgg.............................................................................      1
Gi3_28725190_x1....................................................................guucgauuGccggcgccg..........................      1
Gi2_11252299_x1...............aguccggcguUguauaacgg.............................................................................      1
Gi2_11267262_x1.................Accggcguaguauaacgg.............................................................................      1
```

```
Gi2_11226846_x1.................ucNggcguaguauaacgg..............................................................................     1
Gi5_9118019_x1...............................................................uccggguucgauucccggA...............................     1
Gi1_4264100_x1.........................................................aacagauccggguucgauucccAgcgccgg..........................     1
Gi5_9076870_x1.....................................................................uucgauucccggcgccgC..........................     1
Gi3_28281794_x1..................................................................ggguucgauuccUggcgccgga.........................     1
Gi2_11057050_x1.....................gcguaguaNaacggcuagaa........................................................................     1
Gi3_28911893_x1...............................................guucucacUcaacagauccgggu...........................................     1
Gi3_28565650_x1........................................................caacagauccggguucgauCccc..................................     1
G1a_6303005_x1.................uccggcguaguauaacggcuUga.........................................................................     1
Gi5_8988332_x1....................ggcguaguNuaacggcua...........................................................................     1
Gi5_8909169_x1........................................................................gauucccggcgccgAagc.......................     1
Gi2_11279226_x1.......................................................................cgauucccAgcgccggagcguuu...................     1
Gi2_11228306_x1......................................................................ucAauucccggcgccggag........................     1
Gi1_4205937_x1..................ccggcguaguauaaAggcua...........................................................................     1
Gi3_28344790_x1.................uccggcguaguauaaGggcuagaa........................................................................     1
Gi1_4349058_x1....................................................................guucgGuucccggcgccggag........................     1
Gi1_4367466_x1.....................................................acccaacaAauccggguucgauuccc..................................     1
Gi2_11384950_x1............................................................agaCccggguucgauucc...................................     1
Gi5_9035748_x1...................................................................gNuucgauucccggcgccggagc.......................     1
Gi4_582105_x1 .................uccggcguaguauaacggcuCga.........................................................................     1
Gi2_11089352_x1........................................................................Cauucccggcgccggagc.......................     1
Gi2_11179071_x1.................uccggcguaguauaacggcuUgaa........................................................................     1
Gi1_4201107_x1....................................................................guucgauucUcggcgccggagc.......................     1
Gi4_606205_x1 ....................................................................guucgauucccggcgcAgg..........................     1
Gi2_11317674_x1...............................................guucucaccAaacagauccgggu...........................................     1
Gi1_4198238_x1................................................uucucacccaacagauccggU............................................     1
Gi3_28701100_x1.............................................ugguucucCcccaacagauc................................................     1
Gi3_28812310_x1..............................................gguucucacccaacagaucGg..............................................     1
Gi1_4360278_x1.................uccggcguUguauaacggc.............................................................................     1
Gi3_28789032_x1.................................................ucucacccaUcagauccgg.............................................     1
Gi5_8992771_x1.................uccggAguaguauaacggcuagaa........................................................................     1
Gi3_28333127_x1..........................................................Ucagauccggguucgauuccc..................................     1
Gi1_4314438_x1...............................................guucucacccaacaAauccgg.............................................     1
Gi3_28565667_x1.........................................................................auucccggcgccggagcCu.....................     1
Gi2_11374572_x1.................uccggcCuaguauaacggc.............................................................................     1
Gi1_4304370_x1.................uccggcguaguauaacgU..............................................................................     1
Gi3_28313959_x1..............................................gguCcucacccaacagauccgg.............................................     1
Gi3_28323628_x1.................uccggcguaguauaaGggcuag..........................................................................     1
Gi3_28466710_x1...................................................................Nguucgauucccggcgccg...........................     1
G1a_6056374_x1.................Cccggcguaguauaacggcuaga.........................................................................     1
Gi4_612542_x1 ........................................................................gauucccggcgccggagc.......................     0
Gi3_28449308_x1.....................................................................uucgaCucccggcgccgga.........................     1
Gi3_28984545_x1...................................................ucacccaacagauccgAguuc.........................................     1
Gi2_11218958_x1........................................................................gauucccggcAccggagc.......................     1
Gi5_8872085_x1...................................................................gguucgauucccgUcgccggag........................     1
G1a_5987938_x1.................uccggcguCguauaacggcuagaau.......................................................................     1
Gi5_9064949_x1.................uccUgcguaguauaacggc.............................................................................     1
```

```
Gi2_11309983_x1.......................................................................Ugauucccggcgccggagc......................      1
Gi3_28742472_x1........................................................................gauuccGggcgccggagc......................      1
G1a_6252954_x1................................................uucCcacccaacagauccg.............................................      1
Gi5_8849782_x1...................................................................gguucgauucccggcgccggGgcguuu..................      1
Gi5_9051916_x1....................................................................guNcgauucccggcgccggagc......................      1
Gi1_4234736_x1...................................................................gguucgauucccggcgccgCagc......................      1
Gi2_11387804_x1........................................................................gaCucccggcgccggagc......................      1
Gi3_28831761_x1.............................................................Aauccggguucgauucccggcgccgga........................      1
Gi2_11356626_x1...................cggUguaguauaacggcua..........................................................................      1
Gi5_9066233_x1.................uccggcguaguauaacggcuaUa........................................................................      1
Gi3_28534528_x1..........................................................aUagauccggguucgauu....................................      1
Gi3_28824909_x1.................uccggcguaguUuaacggcuag.........................................................................      1
Gi4_571453_x1 .................uccggcguaguauaacggAuagaau......................................................................      1
G1a_6057563_x1.................uccggcguaguauaaUggcuagaau......................................................................      1
Gi1_4280852_x1...............................................................uccggguucgaCucccggcgccgga........................      1
Gi3_28914095_x1...............................................guucucacccUacagauccgg............................................      1
G1a_6046161_x1.................uccggcguaguauCacggc............................................................................      1
Gi3_28457208_x1..............................................gguucucacccGacagauccgggu..........................................      1
Gi1_4301381_x1....................................................................guucgauucccggcgccggaCc......................      1
Gi3_28547559_x1..............................................gguucucacccaacaCaucc..............................................      1
Gi2_11061510_x1.................ucUggcguaguauaacggcu...........................................................................      1
G1a_6344008_x1...................................................................gguucgauucccggcgccA..........................      1
Gi2_11248818_x1.........................................................aacUgauccggguucgauucccggcgccg..........................      1
Gi3_28690276_x1.................uccNgcguaguauaacggcuagaau......................................................................      1
Gi5_9073696_x1.................uccgCcguaguauaacggcuag.........................................................................      1
Gi2_11075509_x1.....................................................................uucgaAucccggcgccggag.......................      1
G1a_6020122_x1.................uccggcguaguauaacgCcuag.........................................................................      1
Gi1_4167359_x1..................................................................gUguucgauucccggcgccgg.........................      1
Gi4_583134_x1 ....................................................................guucgauuccUggcgccgg.........................      1
Gi5_8939812_x1................................................uucucacccaacagaucGg.............................................      1
Gi1_4213486_x1.............................................ugguucucUcccaacagauccgg............................................      1
Gi5_9101242_x1...............................................................uccggguucgauuccUggc..............................      1
Gi5_8974423_x1....................................................cacccaacagauccgggAucga......................................      1
Gi5_9046109_x1...................cggcguUguauaacggcuagaau......................................................................      1
Gi5_9003694_x1................................................uucucacccGacagauccgg............................................      1
G1a_6201313_x1.....................................................................uucgauucccggcgccggCgc......................      1
Gi1_4325094_x1....................................................................guAcgauucccggcgccg..........................      1
Gi3_28370921_x1..................................................................ggguuAgauucccggcgccggagc......................      1
Gi3_28474226_x1.....................gcguGguauaacggcuagaa.......................................................................      1
Gi5_8839602_x1.................................................ucucacGcaacagauccg.............................................      1
Gi3_28940114_x1...................cggcguaguauaacggUuagaau......................................................................      1
Gi5_9082065_x1...................................................................gguucgauuGccggcgccgg.........................      1
G1a_6154052_x1.................uccgCcguaguauaacggcuag.........................................................................      1
Gi1_4197287_x1.........................................................................auucccggcgccggagcU.....................      1
G1a_6212261_x1....................................................................guucgauucccggcgAcg..........................      1
Gi5_8922531_x1...............................................guucucacccaacagaucccCg...........................................      1
Gi5_8970598_x1............................................uugguucGcacccaacagauccg.............................................      1
```

```
Gi5_8843127_x1..........................................................Ucagauccggguucgauucccggcgc............................	1
Gi2_11297972_x1...................................................................ggNucgauucccggcgccggag.......................	1
G1a_6321473_x1.....................................................................uucgauucccggAgccggagc......................	1
Gi1_4183056_x1...................cggcguagNauaacggcu...........................................................................	1
Gi3_28389825_x1...................Uggcguaguauaacggcuagaau......................................................................	1
Gi5_8890323_x1.................uccggAguaguauaacgg.............................................................................	1
Gi5_8961465_x1...................................................................gguuUgauucccggcgccggagcguuu..................	1
G1a_6265864_x1.................uAcggcguaguauaacggcuag.........................................................................	1
Gi2_11204160_x1...................................................................gguucgauucccCgcgccgg.........................	1
Gi5_9005679_x1...................................................................gguucgauucccggcgccggagcgAu...................	1
Gi2_11034520_x1................................................uucucacccaacaUauccg.............................................	1
G1a_5981303_x1.................Gccggcguaguauaacggcu...........................................................................	1
Gi2_11078882_x1.................uccggcguaguauaacgAcuagaa.......................................................................	1
Gi5_9120349_x1......................................................................ucgauuAccggcgccggagc......................	1
Gi3_28787178_x1......................................................................ucgauuAccggcgccggag.......................	1
Gi3_28559303_x1...............................................guAcucacccaacagauccgg............................................	1
Gi1_4320439_x1..............................................................auccAgguucgauucccggcgccgg.........................	1
Gi1_4382100_x1.................uccggcguCguauaacggcuagaau......................................................................	1
Gi5_9043186_x1.................uccggcguaguauaCcggcu...........................................................................	1
Gi5_8879847_x1..................ccggcguaguauaacggcuUga........................................................................	1
Gi3_28270759_x1.....................gcguaguauaacggUuaga........................................................................	1
Gi2_11394025_x1.....................................................................ucgauucccggcgccgA..........................	1
Gi3_28516924_x1.............................................................gauccggguucgauCccc.................................	1
Gi1_4230010_x1..................ccggcguaguUuaacggcuaga........................................................................	1
G1a_6287206_x1................................................uucucacccaacagaucUg.............................................	1
Gi2_11235190_x1....................................................................guucgauucccggcgccUgag.......................	1
Gi4_577047_x1 ...................................................................gguucgauNcccggcgcc...........................	1
Gi5_9104398_x1....................................................cUcccaacagauccggguucga......................................	1
Gi5_9096554_x1.................uccggcguaguauaacCgcuagaau......................................................................	1
Gi5_8865141_x1....................................................................guuNgauucccggcgccggagc......................	1
Gi1_4276289_x1.............................................ugguucucacccaacagCuccg.............................................	1
Gi1_4249968_x1..............................................gguucucacccaacagauccU.............................................	1
Gi4_574948_x1 ...................................................................gguucgauCcccggcgccggagc......................	1
Gi1_4226254_x1...............................................guucucaccGaacagauccg.............................................	1
Gi5_8927523_x1.............................................ugguucucacccaacagaCccgggu..........................................	1
Gi3_28603845_x1...............................................................uccggguucgauucccggc..............................	0
Gi3_28718778_x1...............................................guucucacccaUcagauccg.............................................	1
Gi5_9027416_x1................................................uucGcacccaacagauccg.............................................	1
G1a_5986143_x1...................................................................gCuucgauucccggcgcc...........................	1
Gi3_28607525_x1..................................................................ggguucgauucccggcgAcggagc......................	1
Gi5_8848946_x1......................................................................ucgauucUcggcgccggag.......................	1
Gi3_28596156_x1.......................................................................cgaNucccggcgccggagc......................	1
Gi3_28943427_x1........................................................caacagaucUggguucgauuccc.................................	1
Gi1_4247741_x1.................ucAggcguaguauaacggcu...........................................................................	1
Gi3_28350141_x1....................ggcguaguauaacggcuaAa........................................................................	1
Gi2_11100384_x1................................................uucucaGccaacagauccg.............................................	1
Gi2_11134632_x1.................uccggcguaguauaacggAuagaa.......................................................................	1
```

```
Gi5_8921644_x1....................................................................guucgauucccggcgcUggag.......................      1
Gi1_4218202_x1.................uccggcguaguauaacgAcu...........................................................................      1
Gi3_28480317_x1.....................................................................uucgauucccgAcgccgg.........................      1
Gi4_570039_x1 ....................................................................guucgauucccggcAccggag.......................      1
Gi3_28366159_x1.............................................................gauccggguucgauucccggcgccggagcC.....................      1
G1a_6302868_x1..............................................................auccggguucgaCucccggc..............................      1
Gi5_9048280_x1....................................................................guucgGuucccggcgccggag.......................      1
Gi5_8961680_x1..............................................gguucucacccCacagauc...............................................      1
Gi3_28692203_x1..............................................gguucuUacccaacagaucc..............................................      1
Gi5_9116548_x1.............................................ugguucuUacccaacagauccgggu..........................................      1
Gi3_28404516_x1.................uccggcguaguauaacggcuU..........................................................................      1
Gi3_28582896_x1..................Ucggcguaguauaacggcuagaa.......................................................................      1
Gi5_9067791_x1..............................................gguucuUacccaacagauc...............................................      1
G1a_6027245_x1...................................................................gguuAgauucccggcgccg..........................      1
G1a_6160626_x1...................................................................gAuucgauucccggcgccggagc......................      1
Gi2_11056791_x1............................................................agauccggAuucgauucc..................................      1
Gi3_28317100_x1..................ccggcguagGauaacggc............................................................................      1
Gi4_603168_x1 .................ucUggcguaguauaacgg.............................................................................      1
Gi2_11039119_x1..............................................................auccggguucgauucccggcgccggagc......................      0
Gi3_28967539_x1..............................................gguucucacccaacagauGcg.............................................      1
Gi2_11110040_x1....................................................................guucgauucccggGgccg..........................      1
Gi3_28518396_x1....................ggcguaguGuaacggcuaga........................................................................      1
Gi2_11060389_x1.....................................................................uucgauucccggcgGcggagc......................      1
Gi1_4328694_x1.......................................................................cgauucccggcgccggagc......................      0
Gi2_11043102_x1.................uccggcguaguauaacgUcuag.........................................................................      1
Gi1_4269937_x1....................ggcguaguNuaacggcua..........................................................................      1
Gi3_28855230_x1...............................................guucucacccaacUgauccgg............................................      1
Gi3_28811057_x1.................uccggcguaUuauaacggcuagaa.......................................................................      1
Gi1_4268067_x1..............................................gguucucacccaacaAauccgg............................................      1
Gi5_8993165_x1..................ccggUguaguauaacggcua..........................................................................      1
G1a_6322424_x1......................................................................ucgauucccggcgccggagcCu....................      1
Gi5_9053569_x1....................................................................guNcgauucccggcgccg..........................      1
Gi2_11346556_x1..................ccggcguaguauaCcggcua..........................................................................      1
Gi5_8862066_x1......................................................................uGgauucccggcgccgga........................      1
Gi1_4269826_x1....................................................cGcccaacagauccggguucgauuccc.................................      1
Gi1_4360840_x1.................uccggcguaguCuaacggcuaga........................................................................      1
Gi1_4264243_x1...............................................................uccggguucgauucccAgcgccgga........................      1
G1a_6088397_x1.................uccggcguaguaCaacggc............................................................................      1
Gi3_28522852_x1.................uAcggcguaguauaacggcua..........................................................................      1
Gi2_11056875_x1..............................................gguucucacccaacaAauccg.............................................      1
Gi5_8959505_x1....................................................................guucgauucccggcgcGgga........................      1
Gi5_8843860_x1..................ccggcguagCauaacggcuag.........................................................................      1
Gi5_8985201_x1.............................................................gGuccggguucgauucccggcgccgga........................      1
Gi1_4197516_x1.................uccggcguaguauaacCg.............................................................................      1
Gi2_11303602_x1...................................................................gguucgauucccggcgccggagcguuu..................      0
G1a_6130492_x1....................................................................guucUauucccggcgccggagc......................      1
Gi1_4348403_x1.................uccggcguaguauaacUgc............................................................................      1
```

```
Gi2_11133468_x1..............................................Cgguucucacccaacagauccg..............................................     1
Gi2_11299987_x1..................ccggcguaguauaacggc.............................................................................     0
Gi5_8994205_x1...................cggcguagNauaacggcuag..........................................................................     1
Gi5_9017302_x1.................................................................Ucggguucgauucccggcgccgga........................     1
Gi2_11007067_x1..............................................gguucucacccaacagauccgg.............................................     0
Gi2_11132369_x1.........................................................................gauucUcggcgccggagc......................     1
Gi5_9131642_x1....................................................................gguucgauucccggcgccggaA.......................     1
G1a_6330959_x1................................................guucucacccaacagaAccg..............................................     1
Gi2_11207120_x1................................................guucucCcccaacagauccg..............................................     1
Gi2_11050879_x1.................................................uucucacccaGcagauccggguucg.......................................     1
Gi5_9032630_x1...............................................gguucucacccaacagauccU..............................................     1
Gi5_8961489_x1.................................................uucucacccaacGgauccgg.............................................     1
Gi2_11209690_x1.....................................................................guucgauuccGggcgccgga........................     1
Gi5_8943072_x1.................................................uucucacccaacCgauccg..............................................     1
Gi3_28964329_x1...................................................................ggguGcgauucccggcgccg..........................     1
Gi3_28500151_x1...................................................................ggguucgauucccggcgA............................     1
G1a_6340938_x1....................................................................gguCcgauucccggcgccggagc......................     1
Gi3_28876647_x1.............................................................aAauccggguucgauucccggcgccggag.......................     1
Gi5_8934898_x1.................uccgCcguaguauaacgg..............................................................................     1
Gi2_11269785_x1.....................................................................guucgauucccggcgGcggag.......................     1
Gi2_11122381_x1......................................................................uucgauucccggUgccggag.......................     1
Gi1_4228821_x1...................................................cGcacccaacagauccggguucgauucc..................................     1
Gi2_11121269_x1.......................cguaguauaacggcuagaaugguugguuc..............................................................     0
Gi3_28584570_x1......................................................................Cucgauucccggcgccgg.........................     1
Gi1_4335218_x1................................................guucucacccUacagauccgg.............................................     1
Gi3_28678496_x1......................gcguaguauaaUggcuaga.......................................................................     1
G1a_6174368_x1.................uccggcguaguauaacggGuag..........................................................................     1
Gi3_28970556_x1.................uccNgcguaguauaacggcuagaa........................................................................     1
Gi2_11134767_x1...................................................cucacccaacagGuccgg.............................................     1
Gi3_28668874_x1.........................................................caacagauccggguucgauucccggcgccggagc......................     0
Gi1_4315660_x1..................................................................cUgguucgauucccggcgccgg.........................     1
Gi5_9138174_x1...................................................................ggguucgauucccggcgAcg..........................     1
Gi2_11228118_x1...............aguccggcguaguauaacggcua...........................................................................     0
Gi3_28819375_x1.............................................................agauccgggCucgauuccc.................................     1
Gi3_28319896_x1...................................................Gucacccaacagauccggguucgauuccc.................................     1
Gi3_28378752_x1..............................................................gauccggguucgauuccA.................................     1
Gi3_28756476_x1....................................................................gguucgauucccgCcgcc...........................     1
Gi3_28888554_x1..............................................ugguucucacAcaacagauccg..............................................     1
Gi5_8839447_x1.................uccggGguaguauaacggcua...........................................................................     1
Gi3_28656184_x1................................................guucucacccaaAagauccg..............................................     1
Gi2_11138958_x1...............aguccggcguaguauCacggcuaga.........................................................................     1
Gi5_9084668_x1.................uccggcguagGauaacgg..............................................................................     1
G1a_6308753_x1.....................................................................guucgauuNccggcgccggagc......................     1
Gi1_4266347_x1................................................guucucacccaacagaAccg..............................................     1
Gi2_11197417_x1.................uccggcguaguUuaacggcuaga.........................................................................     1
Gi1_4158953_x1....................................................................gguucgGuucccggcgccg...........................     1
Gi2_11312903_x1.................uccgAcguaguauaacggcu............................................................................     1
```

Gi5_9084990_x1...guucgauucccggcgccgCagc.......................	1
Gi1_4350415_x1..uucgauucccggcgccAgagc.......................	1
Gi5_8969622_x1...............aguccggcguaguauaGcggcu...	1
Gi2_11000963_x1...guucgauuGccggcgccggagc.......................	1
Gi4_600480_x1 ....guucgUuucccggcgccg...........................	1
Gi5_8949105_x1..gguucgauucccggcgccggCgcguuu...................	1
Gi1_4192941_x1...Aaacagauccggguucgauucccggc...............................	1
Gi1_4159395_x1...guuNgauucccggcgccggagc.......................	1
Gi2_11163367_x1..ugguucGcacccaacagauccg..	1
Gi1_4194882_x1..uGcggguucgauucccggcgccgga.........................	1
Gi5_8919948_x1...uugguucucacccaacaga..	0
Gi2_11291518_x1...cucacccaacagauccgC...	1
Gi4_588732_x1 ....Gccggcguaguauaacggcua..	1
Gi2_11394664_x1..gguucgauuccGggcgccg...........................	1
Gi3_28692740_x1...cucacccaacaAauccgg...	1
Gi5_8858148_x1..................ccggcguaguauaacggcuGga..	1
Gi5_9045459_x1...Agguucgauucccggcgccggagc.......................	1
Gi3_28705800_x1..gAuucucacccaacagauccgg..	1
Gi5_8928381_x1.................uccggcguagAauaacgg...	1
Gi1_4205978_x1..................cUggcguaguauaacggcua..	1
Gi3_28481122_x1.................uccggcguaguauaacgCcuag...	1
Gi2_11405600_x1..cggguucgauucccggcgccggU.........................	1
Gi2_11038569_x1..gguucgauucccggcgccgUa.........................	1
Gi2_11222250_x1.................uAcggcguaguauaacggcuagaau..	1
Gi3_28923883_x1...............Gguccggcguaguauaacggcuagaaugguu..	1
Gi3_28282996_x1..gguucgauucccggcUccgg..........................	1
Gi5_8834311_x1...guucgauucccggcgccggagU.......................	1
Gi2_11204646_x1..................ccggcguCguauaacggcua..	1
Gi1_4261491_x1..gguucgauuccUggcgccggag........................	1
Gi5_9020961_x1..guucucacccaacagauGcgg...	1
Gi4_598497_x1 ....gguucgauucccgAcgccgg..........................	1
Gi3_28263266_x1....................ggcguaguauaCcggcua..	1
Gi5_8866945_x1..ugguucucacccaacagaucUgg...	1
Gi3_28440888_x1..cagauccggguucgauucccggcgGcgg..........................	1
Gi2_11053014_x1..ugguucucacccaacagGucc...	1
Gi5_8893944_x1.................uccggGguaguauaacggc..	1
Gi3_28946249_x1..ugguucucacccCacagauc..	1
Gi2_11103065_x1...cccaacagauccggguGc...	1
Gi2_11237694_x1....................Ugcguaguauaacggcuagaau..	1
Gi1_4177121_x1.................uccggcAuaguauaacggcu...	1
G1a_6044462_x1...ucgauucccggcgccggagc.......................	0
Gi1_4222554_x1.................uccUgcguaguauaacggcuagaau..	1
Gi5_8969474_x1.................uccggcguaguGuaacggcuagaauggu...	1
Gi5_8850834_x1....................ggcguaguauaacggUuag...	1
Gi3_28636057_x1...gggCucgauucccggcgccg...........................	1
Gi3_28383338_x1..cgUguucgauucccggcgccgga.........................	1
Gi3_28815093_x1...ucgauGcccggcgccggag........................	1

```
Gi2_11282668_x1................................................................uccgggAucgauucccggcgccgga........................ 1
Gi2_11252634_x1....................................................................gguucgauucccggcgccU.......................... 1
Gi2_11118619_x1........................................................................cgUuucccggcgccggagcguuu.................. 1
Gi1_4207197_x1....................................................................gguucgauCcccggcgccggag....................... 1
Gi1_4207312_x1....................ggcguaguauaacAgcua........................................................................... 1
Gi5_8965383_x1....................................................................gguucgauucccUgcgcc........................... 1
Gi3_28402350_x1...............................................................auccggguNcgauucccggcgccggagc...................... 1
G1a_6047477_x1....................................................................gCuucgauucccggcgccggag....................... 1
Gi3_28822384_x1................................................guucucacccaaAagauccgg............................................ 1
Gi5_8935263_x1.......................................................cccaacagauUcggguuc........................................ 1
Gi5_9040047_x1................................................guAcucacccaacagauccgg............................................ 1
Gi2_11015154_x1......................................................acccaacagauccggguucgauucc.................................. 0
Gi1_4366673_x1.................uccggcguaguauaacgC.............................................................................. 1
Gi1_4388420_x1....................................................................gguucgaAucccggcgccgg......................... 1
G1a_6309399_x1.................uccggcguaguauaacggcuagaCu....................................................................... 1
G1a_6155221_x1................................................guucucacNcaacagauccg............................................. 1
Gi5_9036859_x1..................................................ucucacccNacagauccgggu.......................................... 1
Gi2_11296464_x1...............................................gguucucacccaacagaAccg............................................. 1
Gi2_11267232_x1..............................................ugguucucacUcaacagauccg............................................. 1
Gi2_11403411_x1...................................................cucacccaacUgauccgg............................................ 1
Gi3_28486264_x1...............................................gguucucacccaacGgauccgg............................................ 1
Gi3_28588303_x1.................................................Nucucacccaacagauccg............................................. 1
Gi5_8849804_x1..................................................ucAcacccaacagauccgg............................................ 1
Gi5_9088288_x1.................uccNgcguaguauaacggcuagaa........................................................................ 1
Gi1_4240262_x1......................................................................uucgauuccGggcgccgg......................... 1
Gi2_11000793_x1.................uccggGguaguauaacggc............................................................................. 1
G1a_6346630_x1.................uGcggcguaguauaacggcuagaau....................................................................... 1
Gi1_4217748_x1...................................................cucacccaaAagauccgg............................................ 1
Gi2_11312605_x1.................uccggcguagGauaacggcuaga......................................................................... 1
Gi1_4220943_x1..................ccggcguaguauaacCgcuaga......................................................................... 1
Gi3_28644478_x1................................................Uuucucacccaacagauccggguucgau..................................... 1
Gi3_28390158_x1......................cguaguauaacAgcuaga......................................................................... 1
Gi3_28353381_x1................................................................uccggguucgauucccggcUccgga........................ 1
Gi1_4388966_x1.......................................................................ucgauucccggcgUcggagc...................... 1
Gi2_11388762_x1...............................................gguucucacccaacGgauc............................................... 1
G1a_6004502_x1...................................................................ggguucgauucccggUgc............................ 1
Gi2_11119670_x1......................................................................uCcgauucccggcgccggagc...................... 1
Gi2_11063689_x1...............................................gguucucaccUaacagauccg............................................. 1
Gi5_8946318_x1.................Cccggcguaguauaacggcuagaaug...................................................................... 1
Gi3_28547069_x1.........................................................................gauucccUgcgccggagc...................... 1
Gi3_28281122_x1......................................................................uucgauucccggcgcUgg......................... 1
Gi2_11244944_x1.................uccggcguaguauaacUgcua........................................................................... 1
Gi5_8901041_x1....................................................................gguucgauucccggcgccgCag....................... 1
Gi3_28915508_x1.........................................................................gauucccggcgccAgagc...................... 1
Gi2_11121472_x1.................uccggcguagGauaacgg.............................................................................. 1
Gi3_28742968_x1.......................................................cccNacagauccggguucgauuccc................................. 1
Gi1_4347531_x1......................................................................uucgauucccggcgccggagcU..................... 1
```

Gi1_4372239_x1...guucucacccaacUgauccg...   1
Gi2_11033285_x1...ugguucucacccaaUagauccg...   1
Gi2_11201441_x1..guucgauucccggcgccgUag.......................   1
Gi2_11098079_x1...ugguucucacccaacagauccgggu..   0
Gi5_9045279_x1...gAuucgauucccggcgccggagcguuu..................   1
Gi1_4195531_x1.................uccggcguaguauaacggcuaUa..   1
Gi2_11271302_x1..Ugguucgauucccggcgcc...........................   1
Gi5_8873427_x1..cucacccaacagauccUg..   1
Gi5_8990686_x1..................ccggcguaguGuaacggcua..   1
Gi5_8952929_x1..gguucucacccaacagauA...   1
Gi2_11171329_x1.................uccggcguaguauaacggcuaC...   1
Gi5_8897780_x1...gguuAgauucccggcgcc...........................   1
Gi1_4260476_x1..................Gcggcguaguauaacggc..   1
Gi5_9058656_x1..uucucacccaacGgauccggguu...   1
Gi3_28529026_x1..guucgauGcccggcgccgga........................   1
Gi1_4284495_x1..ucgauucccggcgccggagU..................   1
Gi1_4256718_x1...guucucacccaacagUuccgg..   1
Gi5_9082997_x1..guucgauucccggcgccggagcg.....................   0
Gi5_8899476_x1...gguucgauucccggcgUcg..........................   1
Gi5_8843474_x1...Ccucacccaacagauccgg..   1
Gi3_28494622_x1...guucucaccAaacagauccgg..   1
Gi1_4229021_x1..ucgauucccggcgccggGgc......................   1
Gi3_28852957_x1..uucucacccaacagauccggguucga......................................   0
Gi3_28443901_x1.................uccggcguaguauaacggcuUg...   1
Gi2_11222279_x1..gauuUccggcgccggagc......................   1
Gi3_28332991_x1..aucUggguucgauucccggcgccggagc......................   1
Gi3_28897627_x1...cagaucUggguucgauuccc.................................   1
Gi5_8956102_x1...cgauucccggcgccggagcA.....................   1
Gi3_28272130_x1.....................gGguaguauaacggcuaga..   1
Gi2_11001708_x1...gAucucacccaacagauccg...   1
Gi2_11154360_x1...Ggauucccggcgccggagcguuuc.................   1
Gi5_8838283_x1...ugUuucucacccaacagauccgggu..   1
Gi3_28647294_x1...ugguGcucacccaacagauccg...   1
Gi5_9101593_x1...guucucacccaacagCuccgg..   1
G1a_6123275_x1.................uccggcguaguauaGcggc..   1
Gi5_8949533_x1...uggGucucacccaacagauccg...   1
G1a_6248764_x1..uuUucacccaacagauccgg..   1
Gi1_4364029_x1..cucacccaacagauccggguucgauuUc..................................   1
Gi4_589114_x1 ....gguucgauucccggcgccggaAc......................   1
Gi5_8858134_x1..gguAcucacccaacagauc...   1
Gi5_9022000_x1..................ccggcguaguauaGcggcuag...   1
Gi5_9063100_x1....................ggcguaAuauaacggcuagaau..   1
Gi1_4259666_x1...gguucgauucccggcUccgg.........................   1
Gi1_4336743_x1..uucuAacccaacagauccgg..   1
Gi5_8853453_x1..guucgauucccgUcgccggag.......................   1
Gi4_594417_x1 ....cucacccaacagauccggguuUg.......................................   1
Gi5_8932366_x1..................ccggcguGguauaacggcua..   1

```
Gi3_28310905_x1.......................................................................ucgauucUcggcgccgga........................     1
Gi2_11320069_x1.....................gcguaguauaacggcuGgaa........................................................................     1
Gi3_28702503_x1................................................guucucacccaacagauccgggA..........................................     1
Gi4_609932_x1 .................Cccggcguaguauaacggcuaga.........................................................................     1
Gi1_4362163_x1............................................guugguucucacccaacaAauccgggu..........................................     1
Gi5_9040621_x1....................ggcguaguauaacggcuagC.........................................................................     1
Gi2_11355574_x1...............aguccggcguaguauaaUggcuaga.........................................................................     1
Gi2_11130540_x1.......................................................................ucgauucccgAcgccgga........................     1
Gi5_9030671_x1....................................................................gguucgauucccggGgcc...........................     1
Gi1_4278256_x1.....................................................................guucgauuGccggcgccggagc......................     1
Gi5_9103980_x1.................uccggcguaguauGacggcu............................................................................     1
Gi1_4371947_x1.................uccggcguaguauaacgCc.............................................................................     1
Gi5_8862734_x1.....................................................................guucgauucccggcgccAgag.......................     1
Gi5_8938031_x1.................................................uucucGcccaacagauccggguu.........................................     1
Gi3_28365131_x1...................cggcguaguauGacggcuagaau.......................................................................     1
Gi3_28461352_x1....................ggcguaguauCacggcua...........................................................................     1
Gi5_8857495_x1..................................................ucuNacccaacagauccg.............................................     1
G1a_6024655_x1.....................................................................guucgauucccggcgccggC........................     1
Gi5_9000985_x1....................Cgcguaguauaacggcuaga.........................................................................     1
G1a_6015802_x1....................................................................gguucgauucccggcgUcg..........................     1
Gi5_9110281_x1.................................................uucNcacccaacagauccg.............................................     1
Gi5_8927650_x1...............................................gguucucacccaCcagauc...............................................     1
Gi3_28459091_x1......................................................................uucgGuucccggcgccgga........................     1
Gi1_4351216_x1....................................................................gguAcgauucccggcgccggagc......................     1
Gi3_28910896_x1....................ggcguaguauaacggcuagaaA.......................................................................     1
Gi5_9072967_x1...............................................gguucucGcccaacagauccgg............................................     1
Gi5_9041740_x1.............................................uugguucucacccaacagauccA.............................................     1
Gi1_4159385_x1...............................................................aGccggguucgauucccggcgccgga........................     1
Gi3_28752902_x1.............................................uugguucucacccGacagaucc..............................................     1
Gi2_11261593_x1............................................................cagauccggguucgauucccggcgccggagc......................     0
Gi3_28962457_x1.......................................................Gccaacagauccggguucgauuccc.................................     1
Gi3_28663955_x1.........................................................caUcagauccggguucgauucccggcgccgg.........................     1
Gi1_4172795_x1.....................................................................guucgUuucccggcgccg..........................     1
Gi3_28293012_x1.......................................................................ucgauuccAggcgccggag.......................     1
Gi4_608816_x1 .................uccggcguaguauaacggcuagU.........................................................................     1
Gi5_8871802_x1...................................................cucacccaacagaucAgg............................................     1
Gi5_9128802_x1....................................................................gguucgauucccgCcgccgga........................     1
Gi1_4284310_x1................................................uucucacccaacagaucGg..............................................     1
Gi3_28316960_x1....................ggcguaguauaUcggcuagaau.......................................................................     1
G1a_6330265_x1....................................................................gguucgauucccggcgcUgga........................     1
G1a_6119872_x1.................uccggcguaAuauaacggcuag..........................................................................     1
Gi2_11013948_x1.....................................................................guucgauuGccggcgccgga........................     1
Gi1_4229325_x1.................uccggcguaguauaaAggcuagaau.......................................................................     1
Gi3_28526989_x1..................cGggcguaguauaacggc.............................................................................     1
G1a_6328472_x1......................................................................uucgauucNcggcgccggagc......................     1
Gi2_11132632_x1.................uccggcguaguauaaAggcuag..........................................................................     1
Gi3_28582693_x1..................................................................Uggguucgauucccggcgccggag.......................     1
```

```
Gi3_28489770_x1......................................................................uucgauuccAggcgccgga........................     1
Gi3_28939063_x1................................................guucucacccaaUagauccg.............................................     1
Gi3_28957958_x1.................uccggcguagCauaacggcuagaa........................................................................     1
Gi3_28905466_x1...................................................cucUcccaacagauccgg............................................     1
Gi5_8993338_x1..............................................ugguucucGcccaacagauccgg............................................     1
Gi3_28672440_x1...............................................gguucucacccaacagauccggAu..........................................     1
Gi1_4211759_x1...............................................gguucucUcccaacagauccgg............................................     1
Gi3_28526016_x1........................................................................cgauucccggcgUcggag.......................     1
Gi3_28321592_x1................................................................Accggguucgauucccggcgccgga........................     1
G1a_6358700_x1.................................................uucucacccaacaCauccg.............................................     1
Gi1_4174400_x1................................................guucucacccaUcagauccg.............................................     1
Gi2_11104122_x1................................................gGucucacccaacagauccgggu..........................................     1
Gi4_586758_x1 ....................................................................gguucgauucUcggcgccgg.........................     1
Gi5_9072601_x1.................uccggcguaguauaacggcuagaauggCu...................................................................     1
Gi2_11072883_x1..................cUggcguaguauaacggcuagaau.......................................................................     1
Gi2_11182677_x1..................................................................Uggguucgauucccggcgccggagc......................     1
Gi1_4220382_x1.....................................................................guuAgauucccggcgccggagc......................     1
Gi1_4246193_x1....................Agcguaguauaacggcua...........................................................................     1
Gi2_11041101_x1....................................................................gguucgauucAcggcgccggga.......................     1
Gi3_28469741_x1....................................................................Nguucgauucccggcgcc...........................     1
Gi3_28467549_x1....................................................................gguucgUuucccggcgccg..........................     1
Gi5_9000220_x1.....................................................................guucgaCucccggcgccggagcguuuc.................     1
Gi2_10996523_x1....................................................................gguucgauucccCgcgccg..........................     1
Gi3_28834179_x1.............................................uugguucucacGcaacagaucc..............................................     1
Gi3_28587312_x1.........................................................................gauucccggcgccggUgc......................     1
G1a_6274430_x1....................................................................gguucgCuucccggcgccggagc......................     1
Gi2_11287223_x1.................uccgUcguaguauaacggcuagaau.......................................................................     1
Gi1_4220275_x1.....................................................................guuGgauucccggcgccggagc......................     1
Gi5_9124695_x1.................................................uucucaccUaacagauccgg............................................     1
Gi3_28584662_x1.........................................................................gauuccAggcgccggagc......................     1
Gi1_4175261_x1....................................................................gguucgauucccggcgccgAag.......................     1
Gi3_28771493_x1.........................................................caGcagauccggguucgauuccc.................................     1
Gi2_11014138_x1.....................................................................guucgauucccgUcgccggag.......................     1
Gi1_4344470_x1..............................................ugguucucacccaacagaAccgg............................................     1
Gi2_11362498_x1....................................................................gguucgauucccUgcgccgg.........................     1
Gi3_28290095_x1...............................................gguucucacccaacagauccgggA..........................................     1
Gi1_4226524_x1.................uccgCcguaguauaacggc.............................................................................     1
G1a_6114946_x1.....................................................................guucgauucccggcgccgA.........................     1
Gi5_8959181_x1...............................................gguGcucacccaacagauccg.............................................     1
Gi2_11328496_x1..............................................ugAuucucacccaacagaucc..............................................     1
Gi3_28740307_x1.........................................................................gauucccggcgccggagcgA....................     1
Gi3_28822165_x1.....................................................................guuGgauucccggcgccgg.........................     1
Gi1_4214366_x1....................................................................gguucgauuAccggcgccggag.......................     1
Gi3_28940332_x1.............................................................Ugauccggguucgauucc..................................     1
Gi5_9059061_x1.................................................uucucacccUacagauccg.............................................     1
Gi2_11184244_x1...................cggcguaguauaacggcuagG.........................................................................     1
```

```
G1a_6164369_x1.................uccggcguaguauaacAgcuagaa.......................................................................    1
Gi3_28577470_x1............................................................agauccggguuAgauuccc.................................    1
Gi3_28373286_x1..............................................................auccgggGucgauucccggcgccgga........................    1
Gi2_11168775_x1......................................................cccaacagauccgggCuc........................................    1
Gi3_28876879_x1...................................................................Aguucgauucccggcgccggagcg.....................    1
Gi3_28341037_x1....................................................................guucgauucccgCcgccggagc......................    1
Gi3_28688297_x1...................................................................gguAcgauucccggcgcc...........................    1
Gi5_9056090_x1.........................................................aacagauccgggCucgauucccggcgccggagc......................    1
G1a_6108774_x1...................................................................gguucgauGcccggcgccgga........................    1
Gi5_8918044_x1.................Gccggcguaguauaacggcuagaaugguu..................................................................    1
Gi1_4191041_x1..............................................gguucucacccaUcagauccg.............................................    1
Gi1_4321927_x1.................uccggcguaguaCaacggcuagaau......................................................................    1
Gi4_588625_x1 ................uccggUguaguauaacggcuag.........................................................................    1
Gi5_8931379_x1.................uccggcguaguauaUcgg.............................................................................    1
Gi2_11092858_x1....................................................................guucgauucccggcUccgga........................    1
Gi5_9065295_x1.................uccgCcguaguauaacggcuagaau......................................................................    1
Gi3_28492981_x1...............................................................uccggguucgGuucccggcgccg..........................    1
Gi2_11393516_x1...............................................guucucacccaacGgauccgg............................................    1
Gi3_28339698_x1...............................................guucucacccaacCgauccgggu..........................................    1
Gi3_28352247_x1...............................................guuAucacccaacagauccg.............................................    1
Gi5_9051218_x1...................................................ucacccaacaAauccggguu.........................................    1
Gi5_8898062_x1....................ggcguaguauaacggUuaga........................................................................    1
Gi5_8944332_x1.................Accggcguaguauaacggcuagaa.......................................................................    1
Gi2_11358202_x1.................uccggcguaguauaacCgc............................................................................    1
Gi5_9050165_x1....................................................................guuAgauucccggcgccg..........................    1
Gi4_604630_x1 ................uUcggcguaguauaacggcuag.........................................................................    1
Gi3_28336392_x1......................................................cccaacCgauccggguucgauuccc.................................    1
Gi1_4382064_x1.................uccggcguaguUuaacggcuaga........................................................................    1
Gi4_603804_x1 ..................................................cucacccaacagauccgg............................................    0
Gi5_8896035_x1................................................uucucacccaacagaucAggguu.........................................    1
Gi1_4169482_x1....................................................................guucgauucccUgcgccg..........................    1
Gi3_28311635_x1.............................................ugguucucacccaacagGuc...............................................    1
Gi5_9048238_x1...................................................................gguucgaAucccggcgccgga........................    1
Gi1_4260549_x1.................uccggcguaAuauaacggc............................................................................    1
Gi3_28624239_x1..................................................cCcacccaacagauccggguucgauucc..................................    1
Gi3_28572390_x1...................................................................gguucgauAcccggcgccgg.........................    1
Gi1_4171915_x1....................................................................guucgauucUcggcgccg..........................    1
Gi1_4215116_x1...................................................................gguucgauucccggGgccgga........................    1
Gi3_28935762_x1.................uccggcguaguauaacggcuagaaugguuggC...............................................................    1
Gi4_609562_x1 ................Gccggcguaguauaacggcuagaau......................................................................    1
Gi3_28412858_x1.......................................................................cgauucccggcgccggagcU.....................    1
Gi2_11113912_x1.................uccggcguaguauaacggcuaCa........................................................................    1
Gi3_28876102_x1...............................................................uccggguucgauucccggUgccgga........................    1
Gi1_4181266_x1.................................................ucucacccaacagaucAg.............................................    1
Gi2_11130143_x1...........................................................cagauccggguucgauucccggcgccAgag.......................    1
Gi4_596994_x1 ............................................uugguucucacAcaacaga.................................................    1
Gi5_8946289_x1...................................................uGacccaacagauccggguu.........................................    1
```

```
Gi3_28806175_x1...................................................................ggguucgGuucccggcgccgga........................     1
Gi4_586628_x1 ...................................................cucacccaacagGuccggguucg.......................................     1
Gi2_11131159_x1...............aguccUgcguaguauaacggcuaga.........................................................................     1
Gi3_28420422_x1.............................................................agauccggguucgauucccggcgccggaA.......................     1
Gi1_4368857_x1....................................................................ggNucgauucccggcgccgg.........................     1
Gi4_569927_x1 ....................................................................gguucgauucccggcgccggagcA.....................     1
Gi5_8837028_x1..................ccggcguaguauaacggUua...........................................................................     1
Gi1_4339970_x1.....................................................................guucgauucccggcgccAgagc......................     1
Gi5_9009059_x1..................ccggcguaguauaacggcuagC.........................................................................     1
Gi3_28909124_x1.................uccggcguaguGuaacggcu............................................................................     1
Gi3_28821354_x1..................................................................cggguucgauucccggcgccggagcA.....................     1
Gi5_9038163_x1....................................................................gguucCauucccggcgccggagc......................     1
Gi5_8906375_x1...................................................cucacccaacagauccgA............................................     1
Gi5_9103621_x1.................................................uucucacccaacagauccggguCc........................................     1
Gi3_28383019_x1...............................................ggCucucacccaacagaucc..............................................     1
G1a_6136401_x1.................uccggcCuaguauaacgg..............................................................................     1
Gi3_28881241_x1.........................................................................gauucccggcgccggagG......................     1
Gi2_11350215_x1....................................................................gguucgauucccggUgccggagcguu...................     1
Gi1_4339676_x1..............................................ugguuAucacccaacagauccg.............................................     1
Gi3_28596304_x1......................................................................uucgauucccggcgccggUgc......................     1
Gi5_9097868_x1.................uccggcguaguauaacgCcuaga.........................................................................     1
Gi5_9070968_x1.......................................................................ucgaCucccggcgccggagc......................     1
Gi1_4255742_x1....................ggcguaguauaacAgcuag...........................................................................     1
Gi2_11301148_x1......................................................................uucAauucccggcgccggagc......................     1
Gi3_28805885_x1....................ggAguaguauaacggcuagaau.......................................................................     1
Gi5_9017963_x1...................cggcguaguGuaacggcuagaau.......................................................................     1
Gi4_573574_x1 .................................................uucucacccaacagauccg.............................................     0
Gi1_4292922_x1.................................................uucucacccUacagauccg.............................................     1
Gi5_8946460_x1................................................guucucUcccaacagauccg.............................................     1
G1a_6098600_x1....................................................................gguucgauucccggcAcc...........................     1
Gi1_4218549_x1..............................................ugguucucacccaacagauccggguucgauuc...................................     0
Gi3_28594514_x1...................................................................ggguucgauucccggcgccggG........................     1
Gi5_9133169_x1..................ccggcguaguauaaUggcuaga.........................................................................     1
Gi5_8903574_x1....................Cgcguaguauaacggcuag...........................................................................     1
Gi5_9085182_x1...............................................gguucucaccAaacagauccgg............................................     1
Gi3_28607845_x1..................................................................cggNuucgauucccggcgccgga........................     1
Gi4_606285_x1 .................................................uucucacccaacagauccggguuc........................................     0
Gi2_11219967_x1.................ucAggcguaguauaacgg..............................................................................     1
G1a_6149789_x1................................................guucucaccUaacagauccg.............................................     1
Gi3_28544967_x1...............................................................Guccggguucgauucccggcgccgga........................     1
Gi2_11354537_x1.......................................................................ucgauuccAggcgccggagc......................     1
Gi5_8884315_x1...............................................Cguucucacccaacagauccggguu.........................................     1
G1a_6007522_x1.................uccggcguaguauaaUggcuaga.........................................................................     1
Gi5_8861140_x1................................................................uccggguucgauuccccCgc.............................     1
Gi5_9069420_x1....................................................ucacccUacagauccggguu.........................................     1
Gi3_28795804_x1...................................................cucGcccaacagauccgg............................................     1
Gi1_4295372_x1..............................................ugguucucUcccaacagaucc..............................................     1
```

```
Gi3_28467228_x1....................................................................gguucgauucccgCcgccggag.......................     1
Gi2_11071155_x1...................................................cucacccaacagauccCg............................................     1
Gi2_11377995_x1.....................................................................guucgauuccGggcgccg..........................     1
Gi1_4239349_x1.................uccggGguaguauaacggcua...........................................................................     1
Gi5_9077327_x1.......................gGaguauaacggcuagaau.......................................................................     1
Gi3_28912503_x1.........................................................................gauucccggcgccggaAc......................     1
G1a_6193663_x1.................Cccggcguaguauaacggcuagaa........................................................................     1
Gi2_11150518_x1....................................................................gguucgauucccggcgGcggag.......................     1
Gi1_4191676_x1.......................................................................ucgauucccggcgccgga........................     0
Gi5_8902456_x1.................................................uucucaUccaacagauccggguu.........................................     1
Gi2_11390696_x1.............................................................agauccggguucAauucc..................................     1
Gi2_11240438_x1.................uAcggcguaguauaacggcua...........................................................................     1
Gi3_28728864_x1...................................................................Agguucgauucccggcgccgga........................     1
Gi4_600813_x1 .................Cccggcguaguauaacggcua...........................................................................     1
Gi5_8836994_x1.................uccggcguaCuauaacggcuagaa........................................................................     1
Gi5_8884678_x1..................................................ucucCcccaacagauccgg............................................     1
Gi2_11404082_x1.................uccggcguaguUuaacggcuagaa........................................................................     1
Gi5_8971807_x1............................................................cagauccgNguucgauucccggcgccg..........................     1
Gi5_8911201_x1.................uccAgcguaguauaacggcu............................................................................     1
Gi4_581976_x1 .................uUcggcguaguauaacggcuaga.........................................................................     1
G1a_6154480_x1.................uccggcguNguauaacggcua...........................................................................     1
Gi5_8891167_x1...................................................cucacccaUcagauccgg............................................     1
Gi1_4222424_x1.....................................................................guucgauuccAggcgccggagc......................     1
Gi2_11033012_x1........................................................................cgaCucccggcgccggagcguuu..................     1
Gi2_11336996_x1................................................guAcucacccaacagauccg.............................................     1
Gi3_28815812_x1...............................................gguucucacccaacagaucAgg............................................     1
Gi5_8896571_x1..............................................uggCucucacccaacagauccgggu..........................................     1
Gi1_4360267_x1.................uccggcguaguauaacggcuaUaau.......................................................................     1
Gi5_8957344_x1.................................................uucucaAccaacagauccgg............................................     1
Gi3_28447617_x1......................................................................uucgauucccgAcgccgga........................     1
Gi1_4238223_x1..................................................ucucaAccaacagauccgg............................................     1
Gi1_4341708_x1....................................................................gguucgauucccggcgccggagcgG....................     1
G1a_6117902_x1.................uccggcguaguauCacggcuag..........................................................................     1
Gi3_28366738_x1.................uccggcguaguauaacgCcua...........................................................................     1
G1a_6262994_x1....................................................................gguAcgauucccggcgccggag.......................     1
Gi3_28467988_x1........................................................................cgauucccggcgccggagcguu...................     0
Gi5_9049110_x1....................................................ucacccaacagauccAgguu.........................................     1
Gi3_28955718_x1.....................................................caUccaacagauccggguuc........................................     1
Gi2_11274941_x1.....................................................................guucgauucccggcCccgga........................     1
Gi2_11266191_x1...................cUgcguaguauaacggcuag..........................................................................     1
Gi2_11319089_x1....................................................................gguucgauucccCgcgccggag.......................     1
Gi4_587775_x1 .................Cccggcguaguauaacggcuagaa........................................................................     1
Gi5_8833470_x1...........................................................acUgauccggguucgauucccggcgc............................     1
Gi2_11238491_x1.....................................................................guucgauucccggcgccggaC.......................     1
Gi3_28440405_x1........................................................................cgNuucccggcgccggagc......................     1
Gi1_4241493_x1...............................................................auccggguCcgauucccggcgccgg.........................     1
Gi5_9052472_x1..............................................ugguucucCcccaacagauccg.............................................     1
```

Gi1_4281978_x1..................Ucggcguaguauaacggc.. 1
Gi2_11358228_x1..ucgauuccGggcgccggagc...................... 1
Gi5_9013166_x1..Cgauccggguucgauuccc................................. 1
Gi4_610798_x1 ....gguucgauucccggcgccgC......................... 1
Gi3_28322090_x1..ggguucgauuccGggcgccgga........................ 1
Gi2_11256551_x1..uucucacccaacagaAccg... 1
Gi3_28795125_x1..................ccggcguaguauaaUggc.. 1
Gi1_4366842_x1..cGcacccaacagauccggguucga...................................... 1
Gi4_588102_x1 ....guucgauucccggcgccggag....................... 0
Gi1_4165188_x1..cucacccaacaAauccgg.. 1
Gi2_11146581_x1...Cgguucucacccaacagaucc.. 1
Gi5_8875077_x1..gguucucGcccaacagauc... 1
Gi3_28381752_x1..ucgauucccggAgccggag....................... 1
Gi5_8897113_x1..gguCcucacccaacagauccggguu... 1
Gi5_8838906_x1...................cggcguaguauaacggcuagG.. 1
G1a_6186067_x1...gguuAgauucccggcgccggagc...................... 1
Gi1_4167615_x1.....................................agaaugguugguucucacc.. 0
Gi3_28375014_x1..guucgauucccggcgccggagcg..................... 0
Gi3_28968230_x1...ucacccaacCgauccggguuc.. 1
Gi3_28941386_x1..cucacccaacagauccgggCucgauu.................................... 1
Gi3_28315274_x1...cagaGccggguucgauucccggcgccgg......................... 1
Gi5_8935087_x1...gguAcgauucccggcgccgg......................... 1
Gi5_9137140_x1.................uccCgcguaguauaacggcuaga.. 1
Gi5_8964200_x1...ggNucgauucccggcgccg.......................... 1
Gi3_28287707_x1..gguucucacAcaacagaucc.. 1
Gi3_28817192_x1...guAcucacccaacagauccg... 1
Gi2_11051528_x1...ugguCcucacccaacagauccg... 1
Gi5_8852998_x1...gguucAauucccggcgcc........................... 1
Gi3_28799809_x1.................uccggcAuaguauaacggcuagaau.. 1
G1a_6345035_x1...ugguucCcacccaacagauccg... 1
Gi3_28489861_x1..agauccggguuAgauucccggcgccggag....................... 1
Gi5_9089275_x1....................ggcguGguauaacggcuaga.. 1
Gi5_8848215_x1................aUccggcguaguauaacggcu... 1
Gi2_11265383_x1...ugguucucacccaacagauAcg... 1
Gi2_11264212_x1...gguucgauucccggcgccggUg....................... 1
Gi2_11323640_x1..gUguucgauucccggcgccggag....................... 1
Gi1_4356054_x1..cucacccaacagauccggguucgauuccU................................. 1
Gi3_28833541_x1...cgauGcccggcgccggagc...................... 1
Gi3_28662191_x1..acagauccggguucgauucccgUcgccgg......................... 1
Gi3_28985309_x1....................ggcguaguauaacggGuaga.. 1
Gi1_4316738_x1..uucccggcgccggagcCu.................... 1
Gi1_4266129_x1...gguucgauucccggcCccggagc...................... 1
Gi2_10996113_x1..gguucucacccaacagauUcg.. 1
Gi3_28352573_x1...Ggauucccggcgccggag....................... 1
Gi1_4361472_x1.................uccggcguGguauaacggcuagaa... 1
Gi1_4328204_x1...gguucgauucccCgcgcc........................... 1
Gi4_577890_x1 ....Gccggcguaguauaacggcuaga.. 1

Gi2_11295437_x1...aacagauccggguAcgauucccggcgccg.......................... 1
Gi3_28851934_x1...guucuUacccaacagauccg... 1
Gi2_11068692_x1.................uccggcguCguauaacggcuagaau.. 1
Gi3_28781900_x1..acagauccggguucgauucccggcgccggG........................ 1
G1a_6271337_x1...Gcccaacagauccggguucgauuccc................................. 1
G1a_5986255_x1.................uccggAguaguauaacggcuaga.. 1
Gi3_28635101_x1..uugguucucacccaacGgaucc.. 1
G1a_5985246_x1.................uccggcguaguGuaacggcuagaa... 1
Gi3_28391917_x1.................uccggcguaguauaaGggcua.. 1
Gi1_4364403_x1...ucUcccaacagauccgggu.. 1
Gi3_28563903_x1..ggguucgauucccggcgccggGg....................... 1
Gi5_8899033_x1...uccggguuNgauucccggc.............................. 1
Gi2_11144807_x1...ucucacccGacagauccgg.. 1
Gi1_4195490_x1..guucgauucccggcgccggaAc...................... 1
Gi3_28789606_x1...gguucgauucccggcUcc........................... 1
Gi3_28857693_x1...Agguucucacccaacagauccg... 1
Gi5_8992512_x1...gguucgauuccGggcgccgga........................ 1
Gi5_8876946_x1.................uccggcCuaguauaacggcuagaau.. 1
Gi5_8920815_x1.................Accggcguaguauaacggcuagaau.. 1
Gi3_28282524_x1...uUacccaacagauccggguuc.. 1
Gi3_28835913_x1...uccggguucgauucccggcgccAga........................ 1
Gi5_9053971_x1..acagauccggguucgauucGcggcgc............................ 1
Gi2_11007780_x1.................uccggcUuaguauaacggc.. 1
Gi5_9119606_x1...ucaccUaacagauccggguu... 1
Gi3_28642877_x1...guucCcacccaacagauccgg.. 1
Gi2_11045545_x1.....................Ucguaguauaacggcuaga.. 1
Gi5_8925876_x1...gguucgauucccggcgcGggagc...................... 1
Gi1_4355106_x1...gCuucgauucccggcgccggag....................... 1
Gi3_28339637_x1....................ggcguaguGuaacggcua.. 1
Gi5_9116559_x1..guucgauucccUgcgccggagc...................... 1
Gi1_4272815_x1..gAuucucacccaacagauccgg.. 1
Gi2_11368171_x1...gguucgauAcccggcgccggag....................... 1
Gi5_9069093_x1....................ggcguaguauaacggcuagaC... 1
Gi5_9124004_x1...............Gguccggcguaguauaacggcu... 1
Gi3_28939267_x1...ucucGcccaacagauccg... 1
Gi2_11292688_x1..uucucacccaacagauUcg... 1
Gi3_28335134_x1..caacagGuccggguucga...................................... 1
Gi3_28933221_x1..gguucucacccaacaUauccg... 1
Gi3_28508720_x1..gguuAucacccaacagauccg... 1
Gi5_9125988_x1..guCcgauucccggcgccggag....................... 1
Gi3_28478583_x1...ccGacagauccggguucgauucccggcgccgg......................... 1
Gi1_4218050_x1...gguucgauucccggcgccgCag....................... 1
Gi5_9128815_x1..guucgauucccggAgccggagc...................... 1
Gi1_4380293_x1..cucacccaacGgauccggguucgauucc.................................. 1
Gi3_28672092_x1..uucucacGcaacagauccg... 1
Gi1_4175616_x1...gguCcgauucccggcgccgga........................ 1
Gi5_8904536_x1..uugguucucacccaacagCuccg... 1

Gi5_9135537_x1..ucacccGacagauccggguu.. 1
Gi1_4387764_x1..ugguucucacccaacagaGccg.. 1
G1a_6216070_x1..Cguucgauucccggcgcc............................ 1
Gi3_28404475_x1..guucucacccaacagauUcgg... 1
Gi3_28756382_x1...uucucacccaacUgauccg.. 1
Gi3_28963298_x1..cgauucccggcgUcggagc....................... 1
Gi3_28878569_x1...gguucucacccaacagGuccgg... 1
Gi1_4347019_x1...guucgauuAccggcgccg........................... 1
Gi5_8904088_x1.................uccggcguagAauaacggcuagaa... 1
Gi3_28683435_x1...acagauccggguucgauCcccggcgccggagc....................... 1
Gi3_28837471_x1...gauCcccggcgccggagc....................... 1
Gi3_28396531_x1..ugguucucaUccaacagauc.. 1
Gi5_8853277_x1..................ccAgcguaguauaacggcua.. 1
Gi5_8974191_x1.................ucAggcguaguauaacggcuagaau.. 1
Gi5_8855737_x1...gguucucacccaacagUuccgg... 1
Gi3_28732959_x1.................uccggcUuaguauaacggcuagaa... 1
Gi4_575170_x1 ....ggCucgauucccggcgccggagc....................... 1
Gi2_11365196_x1...uucuGacccaacagauccg.. 1
Gi3_28428151_x1...guucgauucccgCcgccgga......................... 1
Gi5_9077616_x1..uucgauucccggcgccggagcguuucc................. 0
Gi4_587799_x1 ....uccggcguaguauaacAgcuagaau.. 1
Gi3_28699586_x1...gguucucacUcaacagauccgggu... 1
Gi3_28872144_x1..guucucacccaacaAauccg.. 1
Gi2_11053413_x1..ggCucgauucccggcgccgg.......................... 1
Gi5_8885001_x1...Uuucgauucccggcgccggagcguu.................... 1
Gi2_11372011_x1..cgauucccggUgccggagcguuu................... 1
Gi3_28817291_x1.................Nccggcguaguauaacggcuagaau.. 1
Gi1_4249212_x1..gguucgaCucccggcgccggag........................ 1
Gi5_9053210_x1...gguucCcacccaacagauccgg... 1
Gi2_11321425_x1...agauccggguucUauucc................................... 1
Gi3_28643184_x1..uucgauucccggcgcAggagc....................... 1
Gi5_9003495_x1..guucucacccaacaCauccg.. 1
Gi1_4234834_x1...auccggguucgauucccggcgcUgga......................... 1
Gi2_11261549_x1...uucucaUccaacagauccg.. 1
Gi2_11273501_x1..gguucgauucccgCcgccggag........................ 1
Gi3_28346108_x1..ucCcacccaacagauccg.. 1
Gi5_8876818_x1...cccaacagaucGggguuc... 1
Gi2_11164250_x1..gguucgauucccggcgccggaU........................ 1
Gi1_4247730_x1..uucgCuucccggcgccgg.......................... 1
Gi5_8965853_x1..ugguucucacccaacagCuccgggu... 1
Gi5_9088549_x1.................uccggcguaguauaUcggcu... 1
Gi2_11144917_x1..cgauucccggcgccggaAcguuu................... 1
Gi2_11284343_x1...ucgaAucccggcgccggagc....................... 1
G1a_6035669_x1..gguucgauucccUgcgccgga......................... 1
G1a_6202501_x1..gguucgauNcccggcgcc............................ 1
Gi3_28450955_x1....................ggcgGaguauaacggcua.. 1
Gi5_8858258_x1....................ggcgGaguauaacggcuag... 1

```
Gi3_28527212_x1...................................................................gguucCauucccggcgcc............................     1
Gi5_8921422_x1.....................................................acccaacagGuccggguucgauucc...................................     1
Gi5_8890709_x1.................uccggcguCguauaacggcuagaa........................................................................     1
Gi4_589084_x1 ...................................................................gguucgauucccggcAccgg..........................     1
G1a_6183620_x1.............................................ugguucucacccaacagCuccg..............................................     1
Gi5_8913058_x1......................................................................ucgauucccggcgccggGg........................     1
Gi5_8973379_x1......................................................................ucgauucccggcgAcggagc.......................     1
Gi3_28917137_x1......................................................................ucgauucGcggcgccggagc.......................     1
Gi5_8902831_x1...........................................................cagauccggguucgauCcccggcgccg...........................     1
Gi5_9112113_x1................................................................ccgggCucgauucccggcgccgga.........................     1
Gi3_28276348_x1......................................................................ucgaCucccggcgccggag........................     1
Gi3_28972842_x1........................................................................gauucccCgcgccggagc.......................     1
Gi1_4246456_x1.................................................ucucacccaacaUauccg..............................................     1
Gi3_28344517_x1....................................................cacccaacUgauccggguuc.........................................     1
Gi3_28708828_x1.................................................ucucacccaacagauccgC.............................................     1
Gi1_4375213_x1....................................................................guucgauucccggcgccggagcg......................     0
Gi2_11299356_x1............................................................agauccgAguucgauucc...................................     1
Gi2_11160678_x1...............................................................uccggguucgauucccggcgccggagc.......................     0
Gi2_11267438_x1.................ucNggcguaguauaacggcuagaau.......................................................................     1
Gi5_8964739_x1..................ccggcgCaguauaacggcua...........................................................................     1
Gi2_11326604_x1...................................................................Nguucgauucccggcgccg...........................     1
Gi5_8835091_x1.................uccggAguaguauaacggcu............................................................................     1
Gi5_9021911_x1................................................uuUucacccaacagauccggguuc.........................................     1
Gi2_11388017_x1....................................................................gNucgauucccggcgccgg..........................     1
Gi3_28358554_x1.................uccggcguaCuauaacggcuagaau.......................................................................     1
Gi1_4376981_x1.....................................................................uucgGuucccggcgccggagc.......................     1
Gi5_9001238_x1...................................................................gguNcgauucccggcgccg...........................     1
Gi2_11289923_x1......................................................................ucgNuucccggcgccggagc.......................     1
Gi3_28411947_x1.................................................................cggguucgauucccggcgccgCa.........................     1
Gi5_9010274_x1.................uccggcguaguauaacggcuaAaa........................................................................     1
Gi5_8896414_x1.................................................ucucacccaacagauccAggu...........................................     1
Gi5_9084290_x1................................................uucNcacccaacagauccgg.............................................     1
Gi5_9122369_x1...................................................ucacccaacagauccgCguu..........................................     1
Gi2_11279772_x1.............................................ugguucucacccaacaUauccg..............................................     1
Gi1_4326763_x1.................uccNgcguaguauaacgg..............................................................................     1
Gi2_11085676_x1................................................uucucacccaacagauccU..............................................     1
Gi2_11154727_x1.................uccggcguaguauaCcggcuagaau.......................................................................     1
Gi1_4242195_x1..................................................................ggguucgauucccggcgccgU..........................     1
Gi3_28619795_x1.................................................................cggguucgauuGccggcgccgga.........................     1
G1a_6280899_x1...................................................................gguucgauucccggcgccggagcgA.....................     1
Gi3_28569503_x1.................uccUgcguaguauaacggcuagaa........................................................................     1
Gi5_9042368_x1.................uccggcguaguauaacgCcuag..........................................................................     1
Gi1_4356775_x1....................................................................guuAgauucccggcgccg...........................     1
G1a_6016120_x1.....................................................................uucgauucccggcgccggGgc.......................     1
Gi1_4172546_x1.................uccAgcguaguauaacggc.............................................................................     1
Gi1_4280231_x1....................................................................guucgauGcccggcgccg...........................     1
Gi2_11034387_x1...............................................................uccggguucgaCucccggcgccgga.........................     1
```

Gi4_594361_x1 ....Aucacccaacagauccggguucg.......................................   1
Gi2_11225171_x1..ugguucucacGcaacagauccg...   1
Gi1_4257203_x1...Ugguucgauucccggcgccgga........................   1
Gi5_8948057_x1...Cgguucgauucccggcgccggagc......................   1
G1a_5999126_x1.................uccggcguaguauaacggcCa...   1
Gi3_28533199_x1.................uccggcguaguauaacgUc...   1
Gi5_9111585_x1.................uccggGguaguauaacgg..   1
Gi5_8944125_x1...ggguucgauucccggcgA............................   1
Gi5_9099443_x1...acagauccggguucAauucccggcgc............................   1
Gi1_4308261_x1..gguucgauucccAgcgccg..........................   1
Gi3_28901503_x1...caacagauccggguCcga......................................   1
Gi2_11108052_x1...uucucacccaacagauccgggCucg.......................................   1
G1a_6346247_x1..gguucgauucccgAcgccgg.........................   1
Gi3_28795788_x1...gaAucccggcgccggagc......................   1
Gi5_8895238_x1..gguucgauCcccggcgccggagcguuu..................   1
Gi3_28954306_x1...ucgauucUcggcgccggag.......................   1
G1a_6071110_x1.................uGcggcguaguauaacggcuagaa..   1
Gi2_11349991_x1......................................agGaugguugguucucacc..   1
Gi1_4171366_x1..gguucgauucccggcgcAgga........................   1
Gi2_11144940_x1.................uccggGguaguauaacggcuagaau...   1
Gi2_11109699_x1..guucAcacccaacagauccg...   1
Gi2_11097809_x1..uucgauucAcggcgccgg.........................   1
Gi5_8889832_x1...guucgauucccCgcgccggagc......................   1
Gi1_4389669_x1..gguucUauucccggcgccgg.........................   1
Gi5_8994262_x1...ggguucgauucccggcgccggagcC.....................   1
Gi2_11031798_x1..gguucgauucccggcgcG...........................   1
Gi3_28902693_x1.................uccggcguaguauaaGggcu..   1
Gi5_8942160_x1.................uccggcguaguauaUcggcuagaau...   1
G1a_6109479_x1...uucucaccUaacagauccg...   1
Gi1_4170363_x1.................uccggcguGguauaacggcuagaau...   1
Gi2_11115133_x1..gguuAgauucccggcgcc...........................   1
Gi5_9053092_x1..ugguucucacccaacaUauccgggu..   1
Gi3_28439530_x1..uccggguucgauCcccggcgccgga........................   1
Gi3_28599906_x1...auccggguNcgauucccggcg.............................   1
Gi3_28578201_x1..guucucacccaacagauGcgg..   1
Gi3_28610467_x1..Uuucucacccaacagauccggguucga......................................   1
Gi2_11016489_x1..ugguucucaccAaacagauccg...   1
Gi1_4257465_x1..gguucgauAcccggcgccggag.......................   1
Gi1_4160066_x1..gguucgUuucccggcgccggag.......................   1
Gi2_11135237_x1...guucgauuccUggcgccg..........................   1
Gi3_28838892_x1...auccgAguucgauucccggcgccgga........................   1
G1a_6194525_x1.................Cccggcguaguauaacggcua...   1
Gi5_9050629_x1..acccCacagauccggguucgauuccc.................................   1
Gi5_9066493_x1...guucgauucccggcgccgUa........................   1
Gi3_28361222_x1...Ugauccggguucgauucccggcgccggag.......................   1
Gi3_28336947_x1...guucgauucccggcgccgAag.......................   1
Gi5_9007667_x1...................cggcguaguauaacggcuagaaug..   0

```
Gi2_11387347_x1.................uccggcguaguauaacgCcua..........................................................................    1
Gi2_11158769_x1.....................................................................uucgauucccggcgccAga........................    1
Gi3_28751347_x1..................ccggcguaguauaacggcuGgaau......................................................................    1
Gi3_28573061_x1...............................................................uccggguucgauucccggcAccgga........................    1
Gi4_568672_x1 ...................................................................gUuucgauucccggcgccgga........................    1
Gi5_9052619_x1................................................uucucaccAaacagauccgg............................................    1
Gi1_4372665_x1....................ggcguaguauaacggcCag.........................................................................    1
Gi4_606072_x1 ............................................uuggCucucacccaacaga.................................................    1
Gi5_8892905_x1............................................................agauccggguucgaCuccc.................................    1
Gi2_11282194_x1.....................................................................uucgauucNcggcgccggag.......................    1
Gi3_28724358_x1....................................................cacccGacagauccggguuc........................................    1
Gi5_8863732_x1...................................................................gguucgauucccggcgccggagcguC...................    1
Gi3_28633513_x1.................uccggcguaCuauaacggcuag.........................................................................    1
Gi3_28310110_x1..................ccgNcguaguauaacggc............................................................................    1
Gi2_11059441_x1...................................................................gguucgauucAcggcgccgg.........................    1
Gi4_585899_x1 ...................................................................Aguucgauucccggcgccggagc......................    1
Gi5_9005272_x1.................uccggcguaguauaacggcuUgaau......................................................................    1
Gi3_28427369_x1...................................................ucacccaacagGuccggguuc.........................................    1
Gi3_28571596_x1.................uUcggcguaguauaacggcu...........................................................................    1
Gi3_28675988_x1....................ggcguaguauCacggcuagaau......................................................................    1
Gi2_11301120_x1.......................................................................cgauucccNgcgccggagcguuuc.................    1
Gi1_4332510_x1...................................................................gguucgaGucccggcgccgga........................    1
Gi1_4212311_x1.................................................ucucacccaacagauccAg............................................    1
Gi1_4380634_x1................................................uucNcacccaacagauccgg............................................    1
Gi2_11319370_x1......................................................................ucgGuucccggcgccggag.......................    1
Gi3_28970333_x1.............................................ugguucucacccaacagauAcgg............................................    1
Gi2_11063992_x1..................ccggcguaguauaUcggcua..........................................................................    1
Gi5_8883005_x1..............................................gguucucacccaacagauccgggGu.........................................    1
Gi1_4316550_x1...................................................................gguucgauAcccggcgccggagc......................    1
Gi3_28941279_x1...................................................................gguucgauucccggcgccgUag.......................    1
Gi1_4240444_x1...................................................................gguucgauucccggcgAcggag.......................    1
Gi5_8873819_x1............................................uugguucucacccaacagGuccg.............................................    1
Gi1_4212124_x1.........................................................aacagauccggguucgCuucccggcgccgg.........................    1
Gi3_28311070_x1......................................................................ucgauucccggcgcUggag.......................    1
Gi2_11340065_x1.................uccggcguaUuauaacggcua..........................................................................    1
Gi1_4336238_x1............................................uugguucucacccaacagaucc..............................................    0
Gi1_4205325_x1..................ccgAcguaguauaacggcuaga........................................................................    1
Gi3_28362273_x1..................................................cucaGccaacagauccgg............................................    1
G1a_6163184_x1.................uccggcguaguauaacggcuaAa........................................................................    1
Gi1_4176742_x1.........................................................aacagauccggguucgauucccggUgccgg.........................    1
Gi2_11026130_x1.................uccggGguaguauaacggcuag.........................................................................    1
Gi2_11215724_x1....................................................................guuNgauucccggcgccggag.......................    1
Gi5_9033874_x1.............................................ugguucucacccaacagauAcgg............................................    1
G1a_6158177_x1...................................................................gguucgauucccggcgcAgga........................    1
Gi5_8870721_x1.............................................ugguucucaccUaacagauccgg............................................    1
Gi2_11185963_x1...................................................................gguucgauucccggcgccggagcAuuuc.................    1
Gi2_11016230_x1.....................................................................uucgauucccggcgccAg.........................    1
```

```
G1a_6038980_x1...................................................................ggucgauucccggUgccggagc......................      1
Gi2_11294964_x1.....................gcAuaguauaacggcuagaau.....................................................................      1
Gi3_28400890_x1.................uccggcguaguauaacUgcuag........................................................................      1
Gi2_11358279_x1....................................................................guucgauucccggcgccggagcgC...................      1
Gi3_28271680_x1...................................................................gguucgauucccggGgcc..........................      1
G1a_6137072_x1.................uUcggcguaguauaacggcu..........................................................................      1
Gi3_28813952_x1......................................................................ucgGuucccggcgccggag......................      1
Gi3_28580755_x1.................................................ucucacccaacagauccA............................................      1
Gi5_9097929_x1..............................................gguucucacccaacUgauccgg...........................................      1
Gi3_28811273_x1...................................................................gguucCauucccggcgccggag......................      1
Gi3_28764665_x1..............................................gguucucGcccaacagauccgggu.........................................      1
Gi3_28715958_x1..................................................................ggguCcgauucccggcgccggag......................      1
Gi5_9003785_x1................................................uucucacccaacagauccAg...........................................      1
Gi5_8840570_x1....................................................................guucgauucccggcgGcggag......................      1
Gi5_9052659_x1..............................................gguucucaNccaacagauccgg...........................................      1
Gi5_9083362_x1................................................uucucacccaacagauUcgg...........................................      1
Gi5_9088725_x1.................uccggcguaguauaacAgcu..........................................................................      1
Gi5_9056797_x1....................ggUguaguauaacggcuagaau.....................................................................      1
Gi5_9120741_x1...................................................................gguNcgauucccggcgcc..........................      1
Gi2_11276863_x1..................................................cucacccaaUagauccgg...........................................      1
Gi5_9106722_x1.......................................................................cgauucccggcgcUggagcguuuc................      1
Gi1_4346213_x1....................................................................guucgaAucccggcgccg.........................      1
Gi3_28973006_x1...........................................................cagauccgggGucgauucccggcgccgg........................      1
Gi5_9070166_x1.................................................ucuUacccaacagauccgg...........................................      1
Gi5_8959419_x1..................ccggcguaNuauaacggcuagaau.....................................................................      1
Gi4_592725_x1 ....................................................................guucgauucGcggcgccggagc....................      1
Gi5_9082398_x1.....................................................................uucgGuucccggcgccgg........................      1
Gi1_4264312_x1.................................................ucucaUccaacagauccg............................................      1
Gi3_28509370_x1.............................................ugguucucacccUacagauc..............................................      1
Gi3_28599412_x1..............................................gguucucacAcaacagauccgg...........................................      1
Gi2_11366968_x1.................uccggcguUguauaacggcuag........................................................................      1
Gi3_28481247_x1................................................uucucacccaacagCuccgg...........................................      1
G1a_6242963_x1.................................................ucAcacccaacagauccg............................................      1
Gi2_11172189_x1...............aguccAgcguaguauaacggcuaga.......................................................................      1
Gi1_4368827_x1...................................................................gguucgauucccggcgccCgagc.....................      1
Gi1_4289921_x1.................uccggcguaguUuaacggcuagaau.....................................................................      1
Gi1_4213676_x1.................uccggcguaguauaacCgcuagaau.....................................................................      1
Gi1_4224178_x1...................................................................gguucgauucccCgcgccgg........................      1
Gi3_28585848_x1..............................................................Uuccggguucgauucccggcgccgga.......................      1
Gi2_11352222_x1.................ucNggcguaguauaacggcuaga.......................................................................      1
Gi2_11107507_x1.................uNcggcguaguauaacggcuaga.......................................................................      1
Gi4_571218_x1 ...................................................................gguucgauucccggcgcA.........................      1
Gi3_28615613_x1............................................................Ugauccggguucgauucccggcgccgg........................      1
Gi5_9007389_x1....................................................................guucgauucccggcgccgUagc.....................      1
Gi2_11396827_x1....................................................................guucgauucccCgcgccggag......................      1
G1a_6002738_x1...................................................................gCuucgauucccggcgccgg........................      1
Gi3_28516877_x1............................................................agauccggguCcgauucccggcgccggag......................      1
```

```
G1a_6353152_x1...................................................................gguucAauucccggcgccggagc......................      1
G1a_6289377_x1...................................................................gguucgauucccggcgccggGg.......................      1
G1a_6256295_x1....................................................................guucgauucUcggcgccgga........................      1
Gi1_4256719_x1..............................................gguucucacccaGcagauc...............................................      1
Gi5_8883160_x1...............aguccggcguGguauaacgg.............................................................................      1
Gi1_4372405_x1.................uccggcguaguauGacggcua..........................................................................      1
Gi3_28778243_x1......................................................cccaacagaucUggguucgauuccc.................................      1
Gi2_11010459_x1......................................................................ucgaCucccggcgccgga........................      1
Gi3_28305474_x1....................ggcguaguauaaUggcuaga........................................................................      1
G1a_6305575_x1....................................................................guucgauucccgCcgccgg.........................      1
Gi5_8838262_x1...................................................................gguucgauAcccggcgccgga........................      1
Gi2_11264536_x1........................................................................gauucccggcgccgAagc......................      1
Gi2_11272262_x1.................uccggcUuaguauaacggcua..........................................................................      1
Gi5_8979582_x1.................uccggcguaguaGaacggcuag.........................................................................      1
Gi5_8866384_x1......................................................................ucgauucccggcgccggC........................      1
Gi5_8893444_x1......................................................................ucgauucccggcgccgCagc......................      1
Gi5_8855692_x1.................................................ucucacccaacagaucGg.............................................      1
G1a_6330116_x1....................................................................guucgauucccggcgGcggagc......................      1
Gi5_9132765_x1.................................................ucucacGcaacagauccgg............................................      1
Gi2_11128906_x1...............................................guucucacccaacagauccgA............................................      1
Gi3_28297477_x1..............................................gguucucaAccaacagauccg.............................................      1
G1a_6215091_x1.................uccggcguaguGuaacggc............................................................................      1
Gi4_604906_x1 ....................................................................guucgauucccAgcgccgg.........................      1
Gi2_11243308_x1.................uccggcCuaguauaacggcuag.........................................................................      1
Gi3_28318453_x1...................................................................gguNcgauucccggcgccg..........................      1
Gi2_11151932_x1...............................................................uccggguucgauuccUggcgccgga........................      1
Gi2_11001660_x1.............................................ugguucucacccUacagauccg.............................................      1
Gi5_8954286_x1........................................................................gauucccggcgccggaUc......................      1
Gi5_8915072_x1................................................uucucacccaacagauUcg.............................................      1
G1a_6035510_x1....................................................................guucgaCucccggcgccggag.......................      1
Gi3_28897993_x1.............................................................gaGccggguucgauuccc.................................      1
Gi3_28584321_x1............................................................agauccggguucgauucccggcgccggGg.......................      1
Gi5_9037534_x1................................................uucucacccaacagaGccg.............................................      1
Gi2_11077824_x1.........................................................aUcagauccggguucgauucccggcgccg..........................      1
Gi1_4247162_x1...................................................................gguucgauucccggAgccggag.......................      1
Gi3_28346100_x1..............................................................auccggguucgauucccggcC.............................      1
Gi5_9092870_x1.................uccggcguaguaGaacggcuagaa.......................................................................      1
Gi5_8940077_x1.....................................................................uucgauucccggcgccgga........................      0
Gi2_11257600_x1................................................uucucacccaacagGuccgg............................................      1
Gi5_9036811_x1.................uccggcguaguauUacggcuagaau......................................................................      1
G1a_6278848_x1...................................................................gguucgauucccggcCccggagc......................      1
G1a_6150146_x1................................................uucucacccaacGgauccg.............................................      1
Gi1_4223970_x1....................................................................guucgauucccggcgcGgga........................      1
Gi4_595660_x1 ..................................................cucacccaacagauccggguucA.......................................      1
Gi4_567782_x1 ..................................................Gucacccaacagauccggguucg.......................................      1
Gi2_11248111_x1..............................................gguuGucacccaacagauccg.............................................      1
Gi2_11333413_x1....................................................................guucgaAucccggcgccgga........................      1
```

```
Gi5_9130071_x1............................................................agauccggguCcgauuccc................................   1
Gi1_4364259_x1...................................................................gguucgauucAcggcgccggag......................   1
Gi2_11294532_x1.....................................................................uucgauucccgAcgccgga.......................   1
Gi5_9119994_x1..................cUggcguaguauaacggcua.........................................................................   1
G1a_6021359_x1.................uccggcguaguaAaacggcuaga.......................................................................   1
Gi3_28424338_x1.............................................................gauccggguucgauucccggU.............................   1
Gi4_596825_x1 .................uccggcguaguauaacggUuag........................................................................   1
Gi3_28538480_x1...................................................................gguucgauucccggcgGc..........................   1
Gi3_28568661_x1..............................................gguucucacccaacagaCcc.............................................   1
Gi1_4378852_x1............................................................agauccggguucgauucUc................................   1
Gi5_9050029_x1.............................................................gauccggguucgauucccggUgccgga.......................   1
Gi5_9053796_x1.................uccggUguaguauaacggcu..........................................................................   1
Gi1_4221446_x1..............................................gguucucacccaacaCauccg............................................   1
Gi5_8876399_x1.................uccggcguaguCuaacggcu..........................................................................   1
Gi2_11155780_x1...............................................guucucacccaacagCuccgg...........................................   1
Gi1_4255937_x1..................ccggUguaguauaacggcuag........................................................................   1
Gi5_9078320_x1................................................uucucacccaacUgauccgg...........................................   1
Gi3_28662358_x1.................uccggcguagAauaacggcu..........................................................................   1
Gi2_11002141_x1.....................gGguaguauaacggcuagaa......................................................................   1
Gi1_4274575_x1..................ccggcguagAauaacggcua.........................................................................   1
G1a_6100783_x1...................................................................gguucgauucccgAcgccg.........................   1
Gi3_28555333_x1......................................................................ucgauucccggcgccggaC......................   1
Gi2_11146961_x1...................................................................gguucgauucccggcgccggagcgC...................   1
Gi2_11386236_x1............................................................agaNccggguucgauucc.................................   1
Gi2_11386665_x1......................................................................ucgauucccggGgccgga.......................   1
G1a_6047473_x1....................................................................guucgauucccggcgccgCagc.....................   1
Gi5_9116262_x1.................................................uUucacccaacagauccgg...........................................   1
Gi4_605653_x1 .................uccggcguaguauaGcggcuagaa......................................................................   1
Gi5_9001791_x1.......................................................................cUauucccggcgccggagcguuucc...............   1
Gi3_28684600_x1.....................................................................uucgauuUccggcgccgg........................   1
Gi2_11023000_x1.................uccggcguaguUuaacgg............................................................................   1
Gi1_4342867_x1.......................................................................cgauucccggcgccggagcU....................   1
Gi3_28465680_x1..................ccggcguGguauaacggcuaga.......................................................................   1
Gi1_4214955_x1..................ccggcguaguauaCcggcuaga.......................................................................   1
Gi5_9082625_x1.....................................................................uucgCuucccggcgccgg........................   1
Gi3_28320605_x1..............................................gguucucacccaacUgaucc.............................................   1
Gi5_9047258_x1...................................................................gguucgauucccgAcgccggagcgu...................   1
Gi5_8884712_x1.....................................................................uucgauuAccggcgccgg........................   1
Gi1_4250048_x1..................ccggcguagGauaacggc...........................................................................   1
Gi2_11375064_x1.......................................................ccaacagaNccggguucgauucccggcgccggagc.....................   1
Gi5_8847668_x1.............................................ugguucucacccaacagauUcgggu.........................................   1
G1a_6004882_x1...............................................guucucacccaacCgauccg............................................   1
Gi3_28550482_x1..................................................cucaUccaacagauccgg...........................................   1
Gi5_8911041_x1......................cguaguauaacAgcuagaaugguugguuc............................................................   1
Gi2_11395681_x1.................uccggcguaUuauaacggc...........................................................................   1
Gi1_4277007_x1....................................................................guucgauucccggcUccgg........................   1
Gi3_28783571_x1..................ccggcguGguauaacggcuagaau.....................................................................   1
```

```
Gi1_4348091_x1...................................................................gguucgauucccggcgcccCga.......................      1
Gi1_4320148_x1....................Cgcguaguauaacggcua..........................................................................      1
Gi3_28984927_x1............................................uugguucucacccaacagGucc..............................................      1
Gi2_11088551_x1.......................................................................cgauucccggcgccggagcU.....................      1
Gi1_4186097_x1............................................................agauccggguucgaCuccc.................................      1
Gi5_8883410_x1................................................uucucacccaacagCuccgg............................................      1
Gi2_11193403_x1......................................................cccaacagCuccggguuc........................................      1
Gi1_4302283_x1...............................................guucucacccaacagaucAgg............................................      1
Gi3_28584641_x1..................................................................ggguucgauucccggcgcc...........................      0
Gi5_8937867_x1....................................................................guucCauucccggcgccggag.......................      1
Gi3_28594797_x1....................ggcAuaguauaacggcuag.........................................................................      1
Gi2_11090701_x1................................................uucucacccaacagaGccggguucg.......................................      1
Gi3_28327543_x1...............................................guucucacccaacagaucUgg............................................      1
Gi3_28405878_x1...........................................................cagauccggguucgauucccggcgccg..........................      0
Gi1_4228899_x1...................................................................gguucgauuccGggcgccggag.......................      1
Gi4_579742_x1 ....................................................................guCcgauucccggcgccgg.........................      1
Gi2_11021314_x1......................................................................ucgauucccAgcgccggagc......................      1
Gi3_28702826_x1.......................................................................cgGuucccggcgccggag.......................      1
Gi3_28748721_x1.............................................ugguucucacccCacagauccg.............................................      1
Gi3_28910686_x1.............................................................Aauccggguucgauucccggcgccggag.......................      1
Gi3_28644290_x1..................................................................gggGucgauucccggcgccgga........................      1
Gi1_4278672_x1........................................................caGcagauccggguucgauucccggc..............................      1
Gi3_28680340_x1....................................................................Nuucgauucccggcgccg..........................      1
G1a_6250668_x1....................................................................guucgauucccggUgccgg.........................      1
Gi1_4339347_x1.................uccggcguaguauaacggcAag.........................................................................      1
Gi3_28923520_x1.......................................................................cCauucccggcgccggag.......................      1
Gi1_4236171_x1............................................................aAauccggguucgauuccc.................................      1
Gi3_28444929_x1...............................................................uGcggguucgauucccggcgccg..........................      1
Gi1_4186633_x1...................................................uGacccaacagauccgggu..........................................      1
Gi5_8916686_x1.....................................................................uucgauucccggcgccAg.........................      1
Gi3_28332252_x1............................................................aCauccggguucgauucccggcgccggag.......................      1
Gi1_4332899_x1.............................................ugguucucacccaUcagauccg.............................................      1
Gi5_8956689_x1.................................................ucucGcccaacagauccgg............................................      1
Gi5_8890193_x1....................................................................guucgauucccggcgccggGg.......................      1
Gi5_9016590_x1...............................................guuNucacccaacagauccgg............................................      1
Gi2_11222589_x1......................................................................ucgauucccAgcgccgga........................      1
Gi3_28582943_x1..................................................................ggguucgauucccggAgccgga........................      1
Gi2_11303749_x1....................................................................guucgaAucccggcgccgg.........................      1
Gi4_578888_x1 .................uccggcguNguauaacggcua..........................................................................      1
Gi5_9134220_x1.................uccggcguaguauaaAggcuagaa.......................................................................      1
G1a_6171124_x1.................uccggcguaguauaacgAc............................................................................      1
Gi2_11304603_x1.................uccggcguaguGuaacggcuagaa.......................................................................      1
Gi5_9073159_x1...............................................guucuUacccaacagauccgg............................................      1
Gi5_8950806_x1....................................................................guucgauuUccggcgccggagc......................      1
Gi1_4165515_x1...........................................guugguucCcacccaacagauccgggu..........................................      1
Gi2_11352187_x1.................uccggcguaguauaacggcAaga........................................................................      1
Gi2_11320157_x1.................uccggcguaguauaaGggcuagaa.......................................................................      1
```

Gi5_8872269_x1..guucgauucccggcgUcggag....................... 1
Gi1_4206479_x1..................ccggcguUguauaacggcua.. 1
Gi5_9045801_x1..................ccggcguaguauCacggcuag... 1
Gi1_4240235_x1...Ngguucucacccaacagauccgg.. 1
Gi1_4230805_x1..................ccggcguaguCuaacggc.. 1
Gi3_28881903_x1...Cccggguucgauucccggcgccg.......................... 1
Gi3_28419247_x1...ucacccaacagauccggguuU.. 1
Gi2_11074895_x1...............agGccggcguaguauaacgg... 1
Gi2_11251255_x1.................uccggcguaguauaacCgcuagaa... 1
Gi1_4313075_x1..guucgauucccggcAccg.......................... 1
Gi4_592962_x1 ....gAuucgauucccggcgccggagc...................... 1
G1a_6175626_x1.................uccggcguaguauaacggcuagCa... 1
Gi5_9101401_x1...ucGcacccaacagauccgggu.. 1
Gi2_11252747_x1.................uccggcguaguauaacggGuagaau.. 1
Gi3_28855045_x1.................uccggcguaguauaacUgc.. 1
Gi1_4338121_x1..auccggguucgGuucccggcgccgg......................... 1
Gi2_11278841_x1...gguGcgauucccggcgccggag....................... 1
Gi5_9038510_x1...gguucgaAucccggcgccgg......................... 1
Gi5_9026824_x1..agauAcggguucgauuccc................................. 1
Gi3_28737152_x1..uucucacccaacagaucGgg.. 1
G1a_6239732_x1...gguucgauucccggcgccUgagc...................... 1
Gi5_8901004_x1...................cggcguGguauaacggcuagaau.. 1
G1a_6170784_x1..guucgauucccggcgccAg......................... 1
Gi1_4288143_x1..................Gcggcguaguauaacggcuag... 1
Gi2_11272735_x1...cggguucgauCccggcgccgga......................... 1
Gi3_28641596_x1..gguucucacccaGcagaucc.. 1
Gi5_9073493_x1...cagauccggguucgauucccggAgccg.......................... 1
Gi3_28642363_x1...guGcucacccaacagauccgg.. 1
Gi5_8832460_x1..gguCcucacccaacagauc... 1
Gi5_9050163_x1..uucuAacccaacagauccg... 1
Gi2_11271428_x1...gguucgaGucccggcgccg.......................... 1
Gi5_8888853_x1..guucgauucccggcgGcggagc...................... 1
Gi1_4264589_x1..ucgauucccggUgccggagc...................... 1
Gi3_28890051_x1..guucgauucccUgcgccggag....................... 1
Gi1_4170752_x1...ugguucucacccaacagauccC... 1
Gi5_8833944_x1..uugguucuNacccaacagauccg... 1
Gi1_4266422_x1..uucucacccaacagauccU... 1
Gi3_28922062_x1...Auucucacccaacagauccggg... 1
Gi2_10999928_x1...gguucgauuccGggcgccgga........................ 1
Gi5_9001980_x1..guucgauGcccggcgccg.......................... 1
Gi5_8902752_x1.................uccggcguaguauaaUggcuagaaugguu.. 1
Gi5_9025860_x1...ugguucAcacccaacagauccgggu.. 1
Gi5_9031108_x1..................Gcggcguaguauaacggcuaga.. 1
Gi5_8992574_x1.................uccgUcguaguauaacggcuagaaug... 1
Gi3_28829414_x1..cucacccaacagauccgA.. 1
Gi5_9109561_x1.................uccggcCuaguauaacggcua.. 1
Gi1_4260944_x1..gguucucacccaacGgauccgg.. 1

```
Gi2_11072067_x1..................................................................ggguucgauucccggcgccg..........................    0
Gi1_4175714_x1...................................................................gguucgauucccggcgGcggagc......................    1
Gi1a_5999775_x1................................................Cucucacccaacagauccg.............................................    1
Gi3_28351550_x1.................uccggcguaguaAaacggc............................................................................    1
Gi2_11381308_x1....................................................................guucgauucccggcgccU..........................    1
Gi2_11133986_x1.................................................................cggguucgauucccAgcgccgga........................    1
Gi5_9120986_x1.............................................ugguucucaccAaacagauccgg............................................    1
Gi2_11040866_x1.................uccggAguaguauaacggcuagaau......................................................................    1
Gi5_9025560_x1...................................................................gguucgauucccggcgccggaC.......................    1
Gi2_11243515_x1.......................guaguauaacggcuagaau......................................................................    0
Gi5_8840835_x1...............................................................uccggguucgauucUcggc..............................    1
Gi1_4159212_x1............................................................Ggauccggguucgauuccc.................................    1
Gi1_4221935_x1....................ggcguaguauCacggcua..........................................................................    1
Gi3_28377151_x1.............................................ugguucucaccAaacagauccgg............................................    1
Gi2_11366958_x1.................uccggcguaUuauaacgg.............................................................................    1
Gi1_4275615_x1.................uccggcguUguauaacggcu...........................................................................    1
Gi2_11139607_x1...................cggcCuaguauaacggcuaga........................................................................    1
Gi1_4246585_x1.................uccggcguagAauaacggcuag.........................................................................    1
Gi3_28888618_x1......................................................................ucgauucccggcAccggagc......................    1
Gi3_28425353_x1........................................................caacagauccgggCucgauuccc.................................    1
Gi2_11142208_x1...................cggcguagCauaacggcua..........................................................................    1
Gi3_28475828_x1...........................................................cagauccggguucgCuuccc.................................    1
Gi3_28344005_x1......................................................cccaacagauccUgguucgauuccc.................................    1
Gi5_8849942_x1...............aguccggcguaguauaacggcuagaaAgg....................................................................    1
Gi3_28361274_x1............................................................agauccggguucgauucUc.................................    1
Gi3_28558286_x1....................................................................guucgauucccgAcgccg..........................    1
Gi2_11007588_x1....................................................................guucgaGucccggcgccggag.......................    1
Gi3_28303949_x1...............................................guucucaccAaacagauccg.............................................    1
Gi5_9026658_x1..........................................................aGagauccggguucgauucccggcgc............................    1
Gi1_4267734_x1..............................................................auccgAguucgauucccggcgccgg.........................    1
Gi1_4248739_x1..................ccggcguaguauaGcggcuaga........................................................................    1
Gi2_11324069_x1.................................................................cAgguucgauucccggcgccgga........................    1
Gi5_9077290_x1.....................................................................uucgaCucccggcgccggagc......................    1
Gi2_11148909_x1.................uccggcguaguauaCcggc............................................................................    1
Gi5_8886401_x1...................cggcguaguauaacggcuagCau......................................................................    1
Gi3_28920241_x1........................................................caacagauccggguucgGuuccc.................................    1
Gi5_8969081_x1..............................................gguucucacccaacagauccAgguu.........................................    1
Gi2_11344505_x1.................uccggcguaguauaacggcuagaG.......................................................................    1
Gi1a_6340775_x1.................uccggcguaguauaacggcuagaG.......................................................................    1
Gi1a_6301306_x1...................................................................gguucgauCcccggcgccggagc......................    1
Gi3_28570107_x1..................ccggcguaguauaaAggcuaga........................................................................    1
Gi3_28576477_x1......................................................................ucAauucccggcgccggag.......................    1
Gi1a_6219977_x1.................ucAggcguaguauaacggcuaga........................................................................    1
Gi2_11047646_x1....................Agcguaguauaacggcuaga........................................................................    1
Gi4_570127_x1 ....................................................................guCcgauucccggcgccg..........................    1
Gi5_9045643_x1............................................................agauccggguucgUuuccc.................................    1
Gi5_9129758_x1...................................................................gguucgauucccggcgcGg..........................    1
```

```
Gi5_8910947_x1.................uccggcgGaguauaacggcua..........................................................................      1
Gi5_8876071_x1....................................................................guucgaGucccggcgccgga........................      1
Gi5_8846494_x1.................uccggcguaAuauaacggcuagaa.......................................................................      1
Gi3_28878353_x1.................uccNgcguaguauaacgg.............................................................................      1
Gi1_4274906_x1................................................uucucacccCacagauccg.............................................      1
Gi5_9018585_x1...................................................................gguucgaAucccggcgccggag.......................      1
Gi3_28869815_x1................................................uucuUacccaacagauccgg............................................      1
Gi2_11157373_x1............................................................agauccgggCucgauucc..................................      1
Gi1_4213675_x1.................uccggcgAaguauaacgg.............................................................................      1
Gi3_28825253_x1.................uccggGguaguauaacggc............................................................................      1
Gi2_11310277_x1.................uccggcguaguauaacgUcuagaa.......................................................................      1
Gi2_11061560_x1...................................................................gguucgauucccggcgccggagcAu....................      1
Gi1_4296833_x1..................ccggcAuaguauaacggc............................................................................      1
Gi1_4248429_x1....................................................................guucgauuccGggcgccg..........................      1
Gi2_11071232_x1.................uccggcguaguauaacCg.............................................................................      1
Gi5_9070817_x1....................ggcguaAuauaacggcuaga........................................................................      1
Gi3_28903629_x1...............................................................uccggguucgaCucccggcgccgga........................      1
Gi3_28851276_x1........................................................caacagauccggguucAa......................................      1
Gi5_8952807_x1.....................................................................uucgauCcccggcgccgg.........................      1
Gi3_28507336_x1......................................................................ucgauucccggcgAcgga........................      1
Gi2_11140477_x1................................................uucucacccaacagauccgA............................................      1
Gi3_28323490_x1.............................................ugguucucacUcaacagauccggguucga......................................      1
Gi1_4176203_x1.................uccggcguaguaCaacggcuagaa.......................................................................      1
Gi1_4330992_x1....................................................................guucgaGucccggcgccg..........................      1
Gi5_8886018_x1..................Ucggcguaguauaacggcu...........................................................................      1
Gi2_11060369_x1.................uccggcguaguauaacggcuagaauggu...................................................................      0
Gi2_11318275_x1................................................uGcucacccaacagauccggguucg.......................................      1
Gi3_28263398_x1......................cguaguauaacggcuagC........................................................................      1
Gi5_8892467_x1......................................................cUcaacagauccggguuc........................................      1
Gi1_4259039_x1...............................................guucuAacccaacagauccg.............................................      1
Gi3_28376579_x1....................................................................guucgauuUccggcgccg..........................      1
Gi3_28684001_x1.................uccggcguaCuauaacggcuagaa.......................................................................      1
G1a_6177820_x1.....................................................................uucgauucccggcgccgAagc......................      1
Gi4_612632_x1 ...................................................................gGucgauucccggcgccg..........................      1
Gi1_4185751_x1..............................................ggCucucacccaacagauccgg............................................      1
Gi3_28796834_x1.................uccggcguaguauaaGggcuagaau......................................................................      1
Gi3_28846649_x1.......................................................................cgauucccggcgccggagcgC....................      1
Gi2_11168535_x1.....................................................................uucgauucccggcgccUga........................      1
Gi1_4274785_x1........................................................caacagauccgAguucgauucccggc..............................      1
Gi3_28513235_x1.................uNcggcguaguauaacgg.............................................................................      1
Gi5_8925769_x1....................................................................guucgaAucccggcgccggagcguuu..................      1
Gi5_9102986_x1..................ccggcguagCauaacggcuaga........................................................................      1
Gi3_28552136_x1......................................................................ucgauucccggcgccggU........................      1
Gi4_578344_x1 ...........................................uugguucucacccGacaga.................................................      1
Gi3_28782911_x1..................................................................ggguucgGuucccggcgccggag.......................      1
Gi3_28368155_x1...................................................................ggNucgauucccggcgccgga........................      1
Gi5_8984487_x1....................................................Uacccaacagauccggguucga......................................      1
```

```
Gi1_4194051_x1....................................................................guGcgauucccggcgccg...........................   1
Gi3_28983412_x1.................................................ucucacccaacagauUcgg.............................................   1
Gi2_11173314_x1...................................................................gNuucgauucccggcgccggagc.......................   1
Gi1_4250444_x1..................ccggcguaguaCaacggcua...........................................................................   1
Gi5_8938866_x1.................uccggcguaguauaacggcuaU..........................................................................   1
Gi1_4180272_x1.................................................ucucCcccaacagauccg..............................................   1
G1a_6138565_x1...................................................................gguucgauucccggcgccggagcU......................   1
Gi1_4374816_x1......................................................................Ccgauucccggcgccggagc.......................   1
Gi3_28712356_x1....................................................................guucgauuccGggcgccggag........................   1
Gi5_9047984_x1.....................gcguaguauaGcggcuaga.........................................................................   1
G1a_6299274_x1...................................................................gguucgauucccUgcgccggag........................   1
Gi2_11054248_x1..................ccggcguaNuauaacggcua...........................................................................   1
Gi2_11154278_x1...................................................................gguucgauuAccggcgccg...........................   1
Gi3_28317899_x1...............................................gCucucacccaacagauccgggu...........................................   1
Gi5_8970129_x1............................................uugguucucacAcaacagauccg..............................................   1
Gi1_4241313_x1...........................................................cCgauccggguucgauucccggcgcc............................   1
Gi2_11361890_x1................................................................ccggguucgauucccggcgUcgga.........................   1
Gi3_28712821_x1....................................................................guucgauucccCgcgccg...........................   1
Gi2_10997619_x1......................................................................ucgauucccggcgccggagcU......................   1
Gi1_4286025_x1..............................................gguucucUcccaacagauccg..............................................   1
Gi5_9133689_x1.................uccggcguaguauaacggcuaUaau.......................................................................   1
Gi1_4354408_x1.................................................ucucacccaacagauccgA.............................................   1
Gi5_9063159_x1..............................................gguucucacccaacagGuccggguu..........................................   1
Gi1_4275075_x1...........................................................cagauccgggCucgauucccggcgcc............................   1
Gi2_11059949_x1.................uccggcguaguauaacAgcuagaa........................................................................   1
Gi3_28953674_x1..................ccggcguaguauaaAggc.............................................................................   1
Gi1_4280151_x1....................................................................guucgUuucccggcgccgga.........................   1
Gi5_9067202_x1.................uccggcguagCauaacggcu............................................................................   1
Gi3_28349661_x1...................................................ucacccaacagauccggAuuc.........................................   1
Gi3_28827725_x1...............................................................uccggguucgauAcccggcgccgga.........................   1
Gi2_11330651_x1...............................................................uccggguucgauucccggcgUcgga.........................   1
Gi3_28759380_x1.............................................Ggguucucacccaacagauccgg.............................................   1
Gi3_28471742_x1.................................................ucucacAcaacagauccgg.............................................   1
Gi3_28902627_x1..................ccggcguaguauaacgAc.............................................................................   1
Gi2_11244713_x1......................................................................ucgauucccggcgccAga.........................   1
Gi5_8905453_x1................................................uucucacccaacCgauccggguu..........................................   1
Gi3_28376183_x1...................................................................gguucgUuucccggcgccgg..........................   1
Gi3_28748657_x1..................ccAgcguaguauaacggcuag..........................................................................   1
Gi5_8891075_x1..................................................cucacccaaAagauccgg.............................................   1
Gi5_8924460_x1.............................................ugguucucaGccaacagauccg..............................................   1
Gi2_11124518_x1...................................................................gguucgauAcccggcgccgga.........................   1
Gi2_11209821_x1.................uccggcguaUuauaacggcuagaa........................................................................   1
Gi2_11275391_x1......................................................................ucgauucccggcgccggagA.......................   1
Gi2_11284605_x1...........................................................cagauccggCuucgauucccggcgccggag........................   1
Gi3_28423031_x1...........................................................cagauccggguucgauucccggcgccgC..........................   1
Gi3_28718085_x1.............................................ugguucucacccaacagauU................................................   1
Gi5_9125734_x1................................................uucucacccaacagGuccggguuc.........................................   1
```

```
Gi4_575714_x1 ...............................................ugguucucacccaacGga............................................     1
Gi5_9133714_x1........................................................................ucgauucccggUgccggagc.................     1
Gi4_577240_x1 .................................................Uuucucacccaacagauccgg.......................................     0
Gi1_4328051_x1.................aguccggcguaguauaacggcuaga...................................................................     0
Gi5_9037758_x1...................................................ucNcacccaacagauccg........................................     1
Gi3_28465157_x1...............................................ugguucucacccaacagauccU........................................     1
Gi3_28885944_x1...................uccggcguaUuauaacggcua.....................................................................     1
Gi3_28324364_x1.....................................................................gguucgauucccggcgcGg.....................     1
Gi1_4234343_x1.....................................................................gguucUauucccggcgccg.....................     1
G1a_6169776_x1.....................................................................gguucgaCucccggcgccgga...................     1
Gi3_28774530_x1.................................................................uccgAguucgauucccggcgccgga...................     1
G1a_6286866_x1.....................................................................Cguucgauucccggcgccg.....................     1
Gi1_4282852_x1.....................................................................ggAucgauucccggcgccggag..................     1
G1a_6176978_x1.....................................................................gguucgauCccggcgccgga...................     1
Gi2_11340941_x1.......................................................................uucgCuucccggcgccgg....................     1
Gi2_11070334_x1...................uccggcguaUuauaacggcuaga...................................................................     1
>GLCHR05_5213
The candidate has been discarded because the structure is inconsistent with Dicer processing:
the candidate mature sequence does not form part of a likely miRNA duplex
exp           nnnnnnnnnnnnnnnnnnnnnnnnnnnnnnnnnnnnnnnnnnnnnnnnnnnnnnnnnnnnnnnnnnnnnnnnnnnnnnnnnnnnnnnnnnnnnnnnnnnnnnnnnnn
pri_seq       auaucuggcgcgcccccaguccggcguaguauaacggcuagaaugguugguucucacccaacagauccggguucgauucccggcgccggagcguuucccgccuuccuuc
pri_struct    ......((((((......((((((((((......)))).....(((((......)))))....(((((......))))))))))))))))).....)))).......  #MM


Gi3_8900428_x63185.....................................................................gguucgauucccggcgccggagc.................     0
Gi3_9682832_x43166...................uccggcguaguauaacggcuaga...................................................................     0
Gi3_9927636_x38745.....................................................................gguucgauucccggcgccggagcC................     1
Gi2_2216035_x30808.....................................................................gguucgauucccggcgccggagc.................     0
Gi1_1331494_x26933......................................................................guucgauucccggcgccgg....................     0
Gi3_12301936_x14323.....................................................................gguucgauucccggcgccgga...................     0
Gi3_12467744_x13384......................................................................guucgauucccggcgccgg....................     0
Gi3_12507553_x13080......................................................................guucgauucccggcgccggagcC................     1
Gi2_2855789_x12876.....................................................................gguucgauucccggcgccggagcC................     1
Gi3_12546417_x12822.....................................................................gguucgauucccggcgccggag..................     0
Gi3_12730796_x11530......................................................................guucgauucccggcgccgga...................     0
Gi3_12799683_x11385...................uccggcguaguauaacggcua.....................................................................     0
Gi5_3589931_x10963......................................................................guucgauucccggcgccgg....................     0
Gi2_3032616_x10345......................................................................guucgauucccggcgccggagc.................     0
Gi5_3654433_x9879......................................................................guucgauucccggcgccg.....................     0
Gi3_13160908_x9669......................................................................guucgauucccggcgccggagc.................     0
Gi2_3177371_x8982......................................................................guucgauucccggcgccgg....................     0
Gi3_13264247_x8954...................uccggcguaguauaacgg........................................................................     0
Gi2_3195303_x8882...................uccggcguaguauaacggcuaga...................................................................     0
Gi5_3728878_x8684.....................................................................gguucgauucccggcgccggagc.................     0
Gi3_13351844_x8572...................uccggcguaguauaacggc.......................................................................     0
Gi5_3795072_x7593...................uccggcguaguauaacggcuaga...................................................................     0
```

```
Gi5_3810050_x7380...................uccggcguaguauaacggcuag....................................................................      0
Gi3_13864308_x6910.....................................................................gguucgauucccggcgccg.....................      0
Gi5_3872205_x6628................................................gguucucacccaacagauccg........................................      0
Gi2_3537957_x6599......................................................................guucgauucccggcgccggagcC................      1
Gi3_14018747_x6489.....................................................................Uguucgauucccggcgccggagc.................      1
Gi3_14430267_x5377......................................................................guucgauucccggcgccggag..................      0
Gi5_3990121_x5185.....................................................................gguucgauucccggcgccggagcC................      1
Gi3_14604826_x4914.....................................................................gguucgauucccggcgccgg....................      0
Gi1_1946380_x4673.....................................................................gguucgauucccggcgccgg....................      0
Gi3_14790128_x4604.....................................................................gUuucgauucccggcgccggagc.................      1
Gi1_1960321_x4547...................uccggcguaguauaacgg........................................................................      0
Gi2_3825436_x4527.....................................................................gguucgauucccggcgccggag..................      0
Gi5_4103580_x4349.................................................guucucacccaacagauccg........................................      0
Gi2_3883487_x4338.....................................................................gguucgauucccggcgcc......................      0
Gi5_4133188_x4096.....................................................................gguucgauucccggcgccg.....................      0
Gi3_15101518_x4074...................uccggcguaguauaacggcuag....................................................................      0
Gi1_1995350_x4001...................uccggcguaguauaacggcuag....................................................................      0
Gi3_15217882_x3936................................................gguucucacccaacagauccg........................................      0
Gi5_4205184_x3893...................uccggcguaguauaacggcua.....................................................................      0
Gi5_4228222_x3725...................uccggcguaguauaacgg........................................................................      0
Gi3_15427175_x3678...................uccggcguaguauaacggcuagaau.................................................................      0
Gi5_4301044_x3513...................uccggcguaguauaacggcuagaau.................................................................      0
Gi3_15580261_x3458.....................................................................gguucgauucccggcgcc......................      0
Gi2_4129219_x3450.....................................................................gguucgauucccggcgccg.....................      0
Gi5_4325292_x3416...................uccggcguaguauaacggc.......................................................................      0
Gi5_4342263_x3360.....................................................................gguucgauucccggcgccgga...................      0
Gi1_2046517_x3321...............................................ugguucucacccaacagauccgg.......................................      0
Gi1_2049838_x3306.................................................guucucacccaacagauccg........................................      0
Gi1_2059731_x3261..................................................uucucacccaacagauccg........................................      0
Gi2_4175715_x3240.....................................................................Uguucgauucccggcgccggagc.................      1
Gi3_15854949_x3080..................................................uucucacccaacagauccg........................................      0
Gi2_4263674_x3014...................uccggcguaguauaacggc.......................................................................      0
Gi2_4266688_x3008......................................................................guucgauucccggcgccg.....................      0
Gi1_2084611_x2882..................................................uucucacccaacagauccgg.......................................      0
Gi3_16166426_x2766...................uccggcguaguauaacgA........................................................................      1
Gi2_4338576_x2763.....................................................................gguucgauucccggcgccgga...................      0
Gi3_16196749_x2745......................................................................guucgauucccggcgccg.....................      0
Gi5_4464014_x2733..................................................uucucacccaacagauccg........................................      0
Gi5_4466747_x2732......................................................................guucgauucccggcgccgga...................      0
Gi1_2098525_x2672......................................................................Uuucgauucccggcgccgg....................      1
Gi1_2106457_x2553......................................................................guucgauucccggcgccggagcC................      1
Gi3_16419246_x2520...................uccggcguaguauaacggcuagaa..................................................................      0
Gi1_2118934_x2435...................uccggcguaguauaacggcua.....................................................................      0
Gi5_4584884_x2359.....................................................................gguucgauucccggcgccgg....................      0
Gi2_4550526_x2301.....................................................................gUuucgauucccggcgccggagc.................      1
Gi3_16719768_x2271........................................................................ucgauucccggcgccggagcC................      1
Gi1_2160670_x2236......................................................................guucgauucccggcgccgga...................      0
```

```
Gi1_2162906_x2212................................................gguucucacccaacagauccg........................................     0
Gi2_4595781_x2187...................uccggcguaguauaacggcua.....................................................................     0
Gi2_4659874_x2088......................................................................guucgauucccggcgccgga...................     0
Gi5_4689295_x2063......................................................................guucgauucccggcgccggagcC................     1
Gi2_4678540_x2059......................................................................guucgauucccggcgccggag..................     0
Gi1_2196056_x1931...................uccggcguaguauaacggcuaga...................................................................     0
Gi5_4746918_x1924.....................................................................gguucgauucccggcgcc......................     0
Gi3_17173976_x1910.................................................guucucacccaacagauccg........................................     0
Gi3_17258833_x1863..................................................uucucacccaacagauccgg.......................................     0
Gi5_4797320_x1809......................................................................guucgauucccggcgccggagc.................     0
Gi5_4829364_x1752.....................................................................gguucgauucccggcgccggag..................     0
Gi1_2219646_x1714.................................................guucucacccaacagauccgg.......................................     0
Gi2_4889877_x1702...................uccggcguaguauaacgg........................................................................     0
Gi5_4856838_x1692....................................................cucacccaacagauccgg.......................................     0
Gi3_17723670_x1584.....................................................................Uguucgauucccggcgccgga...................     1
Gi2_5042785_x1544................................................gguucucacccaacagauccg........................................     0
Gi1_2263260_x1508......................................................................guucgauucccggcgccg.....................     0
Gi2_5126744_x1455...................uccggcguaguauaacggcuag....................................................................     0
Gi5_4974773_x1454...............................................ugguucucacccaacagauccg........................................     0
Gi1_2279516_x1437.....................................................................gGucgauucccggcgccgg.....................     1
Gi3_18002792_x1423........................................................................ucgauucccggcgccggagc.................     0
Gi2_5159750_x1419...................uccggcguaguauaacggcuagaau.................................................................     0
Gi2_5190590_x1386..................................................uucucacccaacagauccg........................................     0
Gi3_18114895_x1376.....................................................................Uguucgauucccggcgccggag..................     1
Gi1_2308841_x1354...............................................ugguucucacccaacagauccg........................................     0
Gi3_18209951_x1340...................Gccggcguaguauaacggcuaga...................................................................     1
Gi2_5265076_x1323........................................................................ucgauucccggcgccggagc.................     0
Gi3_18253831_x1317......................................................................Uuucgauucccggcgccgg....................     1
Gi2_5286105_x1300.................................................guucucacccaacagauccg........................................     0
Gi1_2332554_x1264.....................................................................gguucgauucccggcgccggagc.................     0
Gi5_5082952_x1247.................................................guucucacccaacagauccgg.......................................     0
Gi1_2340050_x1226......................................................................guucgauucccggcgccggagc.................     0
Gi3_18631876_x1148......................................................................Uuucgauucccggcgccgga...................     1
Gi5_5178308_x1083......................................................................Uuucgauucccggcgccgg....................     1
Gi5_5191186_x1059......................................................................Uuucgauucccggcgccg.....................     1
Gi3_18881639_x1044.....................................................................gUuucgauucccggcgccgga...................     1
Gi2_5612893_x1032......................................................................Uuucgauucccggcgccggagc.................     1
Gi3_18985137_x1003......................................................................Uuucgauucccggcgccggagc.................     1
Gi5_5228053_x994...................uccggcguaguauaacggcuagaa..................................................................     0
Gi2_5713753_x968...................uccggcguaguauaacggcuagaa..................................................................     0
Gi3_19108956_x964.....................................................................gUuucgauucccggcgccggag..................     1
Gi3_19131046_x956...............................................ugguucucacccaacagauccg........................................     0
Gi1_2424457_x950...................uccggcguaguauaacggc.......................................................................     0
Gi5_5277356_x931.....................................................................Uguucgauucccggcgccggagc.................     1
Gi3_19223083_x923.......................................................................uucgauucccggcgccggagc.................     0
Gi3_19277081_x906..........................................................................gauucccggcgccggagc.................     0
Gi2_5857907_x868......................................................................Uuucgauucccggcgccgg....................     1
```

```
Gi5_5341340_x840...................................................ucucacccaacagauccg........................................ 0
G1a_3705913_x820...................uccggcguaguauaacggcuaga................................................................... 0
Gi2_5947887_x817...............................................ugguucucacccaacagauccg........................................ 0
Gi1_2480982_x805.....................................................................gguucgauucccggcgccg..................... 0
Gi3_19615623_x803.....................................................................Uguucgauucccggcgccg..................... 1
G1a_3739453_x774.....................................................................gguucgauucccggcgccggagc................. 0
Gi3_19734571_x772.................................................guucucacccaacagauccgg....................................... 0
Gi2_6110175_x725.....................................................................gguucgauucccggcgccgg.................... 0
Gi5_5431764_x722................................................Uguucucacccaacagauccg........................................ 1
Gi5_5453834_x702..................................................uucucacccaacagauccgg....................................... 0
Gi3_20044512_x691......................................................................gGucgauucccggcgccgg.................... 1
Gi3_20062420_x686......................................................................guucgauucccggcgccgC.................... 1
Gi2_6221840_x679...................uccggcguaguauaacgA........................................................................ 1
Gi5_5504875_x652.....................................................................gUuucgauucccggcgccggagc................. 1
Gi1_2562309_x634.....................................................................gguucgauucccggcgccgga................... 0
Gi3_20331714_x633.....................................................................Aguucgauucccggcgccggagc................. 1
Gi3_20356944_x628.........................................................................cgauucccggcgccggagc................. 0
G1a_3873470_x626...................uccggcguaguauaacggcuag.................................................................... 0
Gi3_20450237_x607.......................................................................uucgauucccggcgccgga................... 0
Gi3_20477458_x603.......................................................................uucgauucccggcgccggagcC................ 1
Gi3_20490687_x600......................................................................gGucgauucccggcgccgga................... 1
Gi3_20592804_x574..........................................................................gauucccggcgccggagcC................ 1
Gi3_20641249_x565.....................................................................gguucgauucccggcgccgC.................... 1
Gi2_6524433_x562......................................................................gGucgauucccggcgccggagc................. 1
Gi3_20654213_x562......................................................................guucgaCucccggcgccggagc................. 1
Gi3_20695535_x555...................uGcggcguaguauaacggcuaga................................................................... 1
Gi3_20711587_x552.....................................................................gguucgauNcccggcgccggagc................. 1
Gi3_20742876_x547.....................................................................gUuucgauucccggcgccg..................... 1
Gi5_5635437_x543......................................................................gGucgauucccggcgccgg.................... 1
Gi3_20766281_x542........................................................................ucgauucccggcgccggag.................. 0
Gi1_2621476_x538...................uccggcguaguauaacggcuagaau................................................................. 0
Gi3_20813076_x534......................................................................Uucgauucccggcgccggag................... 1
Gi1_2629414_x520.....................................................................Uguucgauucccggcgccgg.................... 1
Gi3_20890919_x518........................................................................ucgauucccggcgccgga................... 0
Gi3_20912056_x514.....................................................................Uguucgauucccggcgccgg.................... 1
Gi5_5680282_x510................................................gUuucucacccaacagauccg........................................ 1
Gi3_20940158_x508....................................................cucacccaacagauccgg....................................... 0
Gi1_2635580_x506...................uccggcguaguauaacggcu...................................................................... 0
Gi2_6718036_x497..........................................................................gauucccggcgccggagc................. 0
Gi2_6721016_x496......................................................................gGucgauucccggcgccgg.................... 1
Gi3_21017851_x495...........................................................................auucccggcgccggagcC................ 1
Gi2_6751449_x486.....................................................................Uguucgauucccggcgcc...................... 1
Gi3_21069318_x486.....................................................................gguucgauucccggcgccggCgc................. 1
Gi2_6769824_x481.....................................................................Uguucgauucccggcgccggag.................. 1
Gi2_6779412_x478........................................................................ucgauucccggcgccggagcC................ 1
Gi1_2664335_x471....................................................cucacccaacagauccgg....................................... 0
Gi3_21175959_x470...................................................ucucacccaacagauccg........................................ 0
```

```
Gi5_5742002_x468......................................................................gGucgauucccggcgccg.....................      1
Gi5_5754997_x462.................................................Uuucucacccaacagauccg........................................      1
Gi3_21241144_x461......................................................................gGucgauucccggcgccggagc.................      1
Gi3_21272709_x455................................................Uguucucacccaacagauccg........................................      1
Gi1_2680859_x446...................................................ucucacccaacagauccg........................................      0
Gi1_2686601_x437.......................................................................uucgauucccggcgccgg....................      0
Gi5_5802448_x432.....................................................................Uguucgauucccggcgccg.....................      1
Gi3_21433943_x429.....................................................................ggCucgauucccggcgccggagc.................      1
Gi5_5815709_x425...................uccggcguaguauaacggcu......................................................................      0
Gi3_21502240_x419...................uccggcguaguauaacAgcuaga...................................................................      1
Gi2_7002965_x415.....................................................................Uguucgauucccggcgccg.....................      1
Gi3_21540181_x414...................uccggcguaguauaacggcu......................................................................      0
G1a_4132002_x412.....................................................................gguucgauucccggcgccggag..................      0
Gi3_21582156_x408...................ucUggcguaguauaacggcuaga...................................................................      1
Gi3_21603665_x404...................uccggcguaguauaacggcuGga...................................................................      1
Gi1_2728696_x387...................................................ucucacccaacagauccgg.......................................      0
Gi3_21757565_x383...................uccggcguaguauaacggcuagG...................................................................      1
Gi3_21767117_x381.....................................................................Uguucgauucccggcgcc......................      1
Gi5_5902217_x373.....................................................................Uguucgauucccggcgccgga...................      1
Gi3_21824415_x373.....................................................................gUuucgauucccggcgccgg....................      1
Gi3_21849616_x369.........................................................................cgauucccggcgccggagcC................      1
Gi3_21850354_x369.....................................................................gguucgauucccggcgccggagcCu...............      1
Gi1_2749901_x367.....................................................................gguucgauucccggcgccggag..................      0
Gi5_5915080_x362..........................................................................gauucccggcgccggagc.................      0
Gi3_21945585_x355...............................................ugguucucacccaacagauc..........................................      0
Gi3_21982977_x351...................Gccggcguaguauaacggcua.....................................................................      1
Gi1_2772066_x347.................................................Uuucucacccaacagauccg........................................      1
Gi3_22011911_x347....................ccggcguaguauaacggc.......................................................................      0
Gi2_7279530_x347..........................................................................gauucccggcgccggagcC................      1
Gi3_22045248_x341.............................................................cagauccggguucgauuccc............................      0
Gi1_2780262_x336.....................................................................gUuucgauucccggcgccgg....................      1
Gi5_5989302_x333................................................gguucucacccaacagauccgg.......................................      0
Gi2_7348465_x332.....................................................................Aguucgauucccggcgccggagc.................      1
Gi2_7360380_x330.....................................................................gUuucgauucccggcgccggag..................      1
Gi5_6005812_x327.....................................................................gUuucgauucccggcgccg.....................      1
Gi3_22176246_x324...................uccggcguaguauaacggcCaga...................................................................      1
Gi3_22209728_x320...................uccggcguaguauaacggcuaA....................................................................      1
Gi2_7428877_x314......................................................................Uuucgauucccggcgccg.....................      1
Gi2_7451906_x308......................................................................guucgaCucccggcgccggagc.................      1
Gi3_22310260_x308.....................................................................gguucgauucccggcgccggGgc.................      1
Gi3_22313956_x308.....................................................................gUuucgauucccggcgcc......................      1
Gi1_2813435_x304...................uccggcguaguauaacgA........................................................................      1
Gi3_22350015_x303.................................................................uccggguucgauucccggcgccgga...................      0
Gi3_22355469_x303................................................gUuucucacccaacagauccg........................................      1
Gi3_22369960_x301......................................................................gGucgauucccggcgccggag..................      1
G1a_4343709_x299.....................................................................gguucgauucccggcgccggagcC................      1
Gi3_22406772_x297.....................................................................gguucgauuccUggcgccggagc.................      1
```

Gi3_22426894_x295..Uuucgauucccggcgccg..................... 1
Gi3_22440421_x293...Cguucgauucccggcgccggagc................. 1
Gi3_22470199_x290...uucgauucccggcgccgg.................... 0
Gi1_2833307_x289..Uucacccaacagauccgg....................................... 1
Gi1_2837629_x286...gguucgauucccggcgccggagcC................ 1
Gi2_7584647_x283...gUuucgauucccggcgcc...................... 1
Gi3_22550896_x282...................Gccggcguaguauaacgg.. 1
Gi2_7604625_x280...uucgauucccggcgccgg.................... 0
Gi3_22591439_x277...................Gccggcguaguauaacggc... 1
Gi2_7623265_x277...Uguucgauucccggcgccgga................... 1
Gi2_7633467_x275...gguucgauNcccggcgccggagc................. 1
Gi3_22625362_x274...................uccggcguNguauaacggcuaga... 1
Gi3_22667789_x270..guucgauucccggcgccggagcCu............... 1
Gi1_2865516_x267..guucgauucccggcgccggagcCu............... 1
Gi5_6179994_x266..Uuucgauucccggcgccgga................... 1
Gi3_22715945_x265..agauccggguucgauuccc............................ 0
Gi1_2868710_x265..ucgauucccggcgccggagc................. 0
Gi2_7692801_x265...gUuucgauucccggcgccg..................... 1
Gi3_22720450_x265...gguucgaCucccggcgccggagc................. 1
G1a_4422156_x265...gguucgauucccggcgccgga................... 0
Gi2_7696235_x264...................Gccggcguaguauaacggcuaga... 1
Gi1_2876324_x260..guucgaCucccggcgccgg.................... 1
Gi5_6222628_x254..agauccggguucgauuccc............................ 0
Gi1_2891471_x253..Uguucucacccaacagauccg.. 1
Gi5_6228460_x253..cccaacagauccggguuc................................... 0
Gi5_6235510_x251...ucucacccaacagauccgg....................................... 0
Gi5_6245734_x248...Uguucgauucccggcgcc...................... 1
Gi3_22899164_x248..guucgaCucccggcgccgga................... 1
Gi1_2903961_x247...uUguucucacccaacagauccgg....................................... 1
Gi5_6257064_x245....................ccggcguaguauaacggcuag.. 0
Gi4_339684_x242...gguucgauucccggcgccgg.................... 0
Gi3_22994850_x240...gguucgGuucccggcgccggagc................. 1
Gi1_2928825_x236...uucgauucccggcgccggagcC................ 1
Gi3_23052063_x235..cacccaacagauccggguuc................................... 0
Gi3_23081319_x233...................uccggcguaguauaacgAcuaga... 1
Gi5_6308565_x232...Uguucgauucccggcgccgg.................... 1
Gi1_2938596_x230...Cguucgauucccggcgccgg.................... 1
Gi5_6317809_x230..ucgauucccggcgccggag.................. 0
Gi2_7899826_x230...uucgauucccggcgccggagC................. 1
Gi5_6326282_x228..ucgauucccggcgccggagc................. 0
Gi5_6331966_x226..guucgauucccggcgccggag.................. 0
Gi2_7938475_x225...gguucgauucccggcgccggCgc................. 1
Gi3_23176138_x225...................uUcggcguaguauaacggcuaga... 1
Gi3_23193875_x224...gguucgauuccUggcgccggag.................. 1
Gi3_23200799_x223..guucgaCucccggcgccgg.................... 1
Gi5_6359700_x221....................ccggcguaguauaacggcua... 0
G1a_4538766_x220..uucucacccaacagauccg.. 0

```
Gi1_2963766_x219......................................................................gCucgauucccggcgccgg....................       1
G1a_4546649_x218.....................................................................gguucgauucccggcgccgg....................       0
Gi2_7994399_x217......................................................................Uuucgauucccggcgccggag..................       1
G1a_4549477_x217.................................................guucucacccaacagauccg........................................       0
Gi1_2970519_x216...................uccggcguaguauaacggcuagaa..................................................................       0
Gi3_23304530_x215...................................................ucucacccaacagauccgg.......................................       0
Gi3_23309690_x215...................uccggcguaguauaGcggcuaga...................................................................       1
G1a_4557874_x214.....................................................................gguucgauucccggcgccg.....................       0
Gi5_6398702_x214.....................................................................gUuucgauucccggcgccgga...................       1
Gi2_8017908_x214.......................................................................uucgauucccggcgccggagc.................       0
Gi5_6408310_x213...................Gccggcguaguauaacggcuag....................................................................       1
Gi5_6417005_x211........................................................................ucgauucccggcgccggagcC................       1
Gi3_23359579_x211....................ccggcguaguauaacggcuaga...................................................................       0
Gi3_23382327_x210......................................................................guucgauucccggcgccgA....................       1
Gi1_2985636_x210......................................................................Uuucgauucccggcgccgga...................       1
Gi3_23458130_x204...................uccggcguGguauaacggcuaga...................................................................       1
Gi5_6461919_x203.....................................................................Uguucgauucccggcgccggag..................       1
Gi5_6465567_x202...............................................ugguucucacccaacagauccgggu.....................................       0
Gi1_3010075_x201.......................................................................uucgauucccggcgccggagc.................       0
Gi3_23506713_x201..........................................................caacagauccggguucgauuccc............................       0
Gi3_23516931_x200.....................................................................gguCcgauucccggcgccggagc.................       1
Gi2_8127732_x200.....................................................................ggCucgauucccggcgccggagc.................       1
Gi2_8144076_x198......................................................................Uuucgauucccggcgccgga...................       1
Gi3_23560200_x197...............................................................gauccggguucgauuccc............................       0
Gi3_23572013_x196...................uccggcguaguGuaacggcuaga...................................................................       1
Gi3_23573973_x196.....................................................................gCuucgauucccggcgccggagc.................       1
Gi5_6501422_x196................................................gguucucacccaacagauc..........................................       0
Gi3_23604809_x194..................uGcggcguaguauaacggcua......................................................................       1
Gi5_6522813_x193...................Gccggcguaguauaacggcuaga...................................................................       1
Gi3_23634928_x192................................................gguucucacccaacagaucc.........................................       0
G1a_4632800_x191......................................................................guucgauucccggcgccggagcC................       1
Gi5_6534139_x191.................................................gGucucacccaacagauccg........................................       1
Gi3_23641643_x191......................................................................guucgauucccggUgccggag..................       1
Gi3_23661655_x190.................................................Uuucucacccaacagauccg........................................       1
Gi2_8225747_x189................................................Uguucucacccaacagauccg........................................       1
Gi3_23694905_x188....................................................................Agguucgauucccggcgccggagc.................       1
Gi3_23697725_x188.....................................................................ggGucgauucccggcgccggagc.................       1
Gi3_23707837_x187...................Cccggcguaguauaacggcuaga...................................................................       1
Gi5_6557180_x187.....................................................ucacccaacagauccggguu....................................       0
G1a_4644493_x187......................................................................guucgauucccggcgccggagc.................       0
Gi3_23739640_x185...................uccggcguaguauaacggA.......................................................................       1
Gi2_8259226_x185........................................................cccaacagauccggguuc...................................       0
Gi3_23749033_x184.....................................................................gAuucgauucccggcgccggagc.................       1
Gi1_3057106_x183................................................gguucucacccaacagauccgg.......................................       0
G1a_4658536_x183.....................................................................gguucgauucccggcgcc......................       0
Gi5_6615364_x179......................................................................Uuucgauucccggcgccggagc.................       1
Gi4_367620_x179......................................................................guucgauucccggcgccgg.................... 0
```

```
Gi5_6612679_x179......................ggcguaguauaacggcuag....................................................................      0
Gi1_3071396_x179................................................gUuucucacccaacagauccg........................................      1
Gi1_3070143_x179.............................................................agauccggguucgauuccc.............................      0
Gi3_23840974_x178..............................................uugguucucacccaacagaucc.........................................      0
Gi5_6632435_x177.....................................................................gguucgauucccggcgccggagcCu...............      1
Gi3_23865777_x176.........................................................................cgauucccggcgccggag..................      0
Gi1_3086057_x175......................................................................Uuucgauucccggcgccg.....................      1
Gi1_3085182_x175.................................................Uuucucacccaacagauccgg.......................................      1
Gi5_6646480_x175...............................................ugguucucacccaacagauccgg.......................................      0
Gi3_23894898_x174................................................gguucucacccaacagauccgggu.....................................      0
Gi1_3095764_x172...............................................Ggguucucacccaacagauccgg.......................................      1
Gi3_23948305_x171......................................................................guucgaCucccggcgccggag..................      1
Gi2_8390578_x170.....................................................................gUuucgauucccggcgccgga...................      1
Gi3_23962653_x170...................ucUggcguaguauaacggcua.....................................................................      1
Gi1_3103256_x169.....................................................ucacccaacagauccgggu.....................................      0
Gi5_6685354_x169.....................................................................gUuucgauucccggcgccgg....................      1
Gi3_24013811_x167...................uccgUcguaguauaacggcuaga...................................................................      1
Gi2_8419886_x166......................................................................gGucgauucccggcgccg.....................      1
Gi2_8436782_x165.....................................................................gguucgauucccggcgccggGgc.................      1
Gi3_24044896_x165...................uccggcguaguauaacggcuaAa...................................................................      1
Gi5_6719756_x164..........................................................................gauucccggcgccggagcC................      1
Gi3_24072831_x163.....................................................................gguucgauucccggUgccggagc.................      1
G1a_4721617_x163......................................................................guucgauucccggcgccgg....................      0
Gi3_24090728_x162...................................................................cggguucgauucccggcgccgga...................      0
Gi5_6738710_x162......................................................................guucgaCucccggcgccgg....................      1
Gi2_8472510_x161........................................................................ucgauucccggcgccggag..................      0
Gi5_6747445_x161..................................................uucucacccaacagauccggguu....................................      0
Gi3_24113960_x161.....................................................................gguucgauCcccggcgccggagc.................      1
Gi3_24111706_x161......................................................................gGucgauucccggcgccg.....................      1
Gi5_6756601_x160.......................................................................uucgauucccggcgccgg....................      0
Gi5_6755161_x160.................................................Uuucucacccaacagauccgg.......................................      1
Gi3_24132052_x160................................................gguucucacccaacagauccgg.......................................      0
Gi1_3143858_x159.................................................gGucucacccaacagauccg........................................      1
Gi3_24143030_x159...................uccggcguaguauaaUggcuaga...................................................................      1
Gi3_24150185_x159.....................................................ucacccaacagauccggguuc...................................      0
Gi3_24142553_x159.....................................................................gguucgauucccAgcgccggagc.................      1
Gi5_6778507_x158.................................................................uccggguucgauucccggc.........................      0
Gi2_8512592_x157.....................................................................gguucgaCucccggcgccggagc.................      1
G1a_4740628_x157...................................................ucucacccaacagauccg........................................      0
Gi1_3155014_x156.................................................guucucacccaacaCauccgg.......................................      1
Gi2_8518708_x156.........................................................................cgauucccggcgccggagcC................      1
Gi1_3162430_x154.....................................................................gguucgauucccggcgccggagcCu...............      1
Gi3_24232226_x153...................uccggcguagCauaacggcuaga...................................................................      1
Gi5_6811730_x153......................................................................guucgauucccggcgccggaCc.................      1
Gi1_3167020_x152......................................................................Auucgauucccggcgccgg....................      1
Gi3_24263996_x151.....................................................................gguucgauucccggcgccggagcA................      1
Gi3_24257654_x151.....................................................................Aguucgauucccggcgccggag..................      1
```

Gi3_24258258_x151...................uccggUguaguauaacggc... 1
Gi2_8579332_x150..agauccggguucgauucc............................. 0
Gi2_8603634_x148.......................................agaaugguugguucucacc... 0
Gi3_24312302_x148...gguucgauucccggcgAcggagc................. 1
Gi3_24331334_x147..auccggguucgauucccggcgccgga................... 0
Gi1_3193841_x145..guucgauuNccggcgccgg.................... 1
Gi2_8651444_x144...Uuucucacccaacagauccg.. 1
Gi1_3205093_x143....................ccggcguaguauaacggcua... 0
Gi3_24415889_x142...gguucAauucccggcgccggagc................. 1
Gi2_8670143_x142...Cguucgauucccggcgccggagc................. 1
Gi3_24417877_x142..guucgauucccggcgccggaC.................. 1
G1a_4799734_x141...................uccggcguaguauaacgg.. 0
Gi1_3216716_x141...................Gccggcguaguauaacgg.. 1
Gi2_8681736_x141...ugguucucacccaacagaucc... 0
Gi3_24449273_x140...guucucacccaacagauccggg...................................... 0
Gi5_6938237_x139..guucgauucccggcgccggagcCu............... 1
Gi5_6930592_x139...auucccggcgccggagcC................ 1
Gi2_8698787_x139...................uccggcguaguauaacggcuaAaa.. 1
Gi3_24512108_x136...ggAucgauucccggcgccggagc................. 1
Gi2_8736673_x136..gGucgauucccggcgccgga................... 1
Gi2_8744776_x135..guuUgauucccggcgccggag.................. 1
Gi1_3244829_x134..Uuucgauucccggcgccggagc................. 1
Gi3_24559584_x134..uucucacccaacagauccggguuc................................... 0
Gi5_6979068_x134...gguucgauuUccggcgccgg.................... 1
Gi3_24551410_x134..ggguucgauucccggcgccggagc................. 0
Gi5_6975584_x134...gUuucgauucccggcgccggag.................. 1
Gi1_3250029_x133....................ccggcguaguauaacggcuaga... 0
Gi5_7003636_x131..gGucgauucccggcgccgga................... 1
Gi1_3258716_x131..cucacccaacagauccCg....................................... 1
Gi3_24613453_x131...gguuUgauucccggcgccggagc................. 1
Gi5_7008202_x130...gUuucgauucccggcgcc...................... 1
G1a_4841853_x129...................uccggcguaguauaacggc... 0
Gi3_24642742_x129...................uccggcguaguauCacggcuaga... 1
Gi1_3271991_x127..guCcgauucccggcgccgg.................... 1
Gi5_7036657_x127..guucgaCucccggcgccg..................... 1
Gi3_24687352_x126...Aguucgauucccggcgccgga................... 1
Gi3_24694660_x126...gguucgauucccggcgccggagA................. 1
Gi5_7068052_x124....................ccggcguaguauaacggcuaga... 0
Gi3_24739411_x123...gguucgauNcccggcgccgga................... 1
Gi3_24759981_x122...................uGcggcguaguauaacggc... 1
Gi3_24749611_x122...gguucgauucccggcgcUggagc................. 1
Gi1_3298259_x121....................ccggcguaguauaacggc... 0
Gi5_7102542_x121..uugguucucacccaacagauccg.. 0
Gi2_8905500_x121...gguCcgauucccggcgccggagc................. 1
Gi5_7117155_x119...................Gccggcguaguauaacgg.. 1
Gi3_24812582_x119..caacagauccggguucga................................. 0
Gi3_24828166_x118...guucucacccaacagauccgggu..................................... 0

Gi1_3311683_x118...Uguucgauucccggcgccggagc.................   1
Gi4_406299_x117...................uccggcguaguauaacggcuaga...0
Gi3_24835557_x117...gguucgauNcccggcgccggag..................   1
Gi5_7131131_x117...Cguucgauucccggcgcc......................   1
Gi2_8951030_x117...Uguucgauucccggcgccgg....................   1
Gi3_24857697_x116...................uccggUguaguauaacggcuaga...   1
Gi1_3326847_x115..Gucucacccaacagauccg..   1
Gi3_24876850_x115...................uccggcguGguauaacggcua...   1
Gi5_7166130_x114...................Gccggcguaguauaacggc...   1
Gi1_3331080_x114...................Gccggcguaguauaacggcuag..   1
Gi3_24942169_x111...gguucgauucccggcgccggaCc.................   1
Gi2_9037156_x110..ucgauucccggcgccgga...................   0
Gi5_7202873_x110...uUguucucacccaacagauccg..   1
Gi3_24965152_x110...................uGcggcguaguauaacgg..   1
Gi5_7222001_x108...................Gccggcguaguauaacggcuagaau...   1
Gi3_24998583_x108...................uccggcguaguauaacggUuaga...   1
Gi5_7223837_x108..Gucucacccaacagauccg..   1
Gi3_24991239_x108...................Gccggcguaguauaacggcuagaau...   1
Gi3_25019293_x107..guucgaCucccggcgccg.....................   1
Gi3_25009663_x107..Uauucccggcgccggagc.................   1
Gi3_25015334_x107..ggguucgauucccggcgccggag..................   0
Gi1_3358450_x107..Gucucacccaacagauccgg.......................................   1
Gi5_7233480_x107...................uGcggcguaguauaacggcuaga...   1
Gi2_9076486_x106.................aguccggcguaguauaacgg..   0
Gi4_415438_x105...gguucgauucccggcgccggagc.................0
Gi5_7247474_x105...ucucacccaacagauccgggu.....................................   0
Gi1_3372775_x105..gGucgauucccggcgccgga...................   1
G1a_4940392_x105...uucgauucccggcgccggagc.................   0
Gi2_9109341_x104..gGucgauucccggcgccggag..................   1
Gi5_7261905_x104...................Gccggcguaguauaacggcua...   1
Gi3_25095319_x103....................ccggcguaguauaacggcuag..   0
Gi3_25091096_x103...................Gccggcguaguauaacggcuag..   1
Gi2_9115834_x103...gguucgGuucccggcgccggagc.................   1
Gi3_25104011_x102...gguAcgauucccggcgccggagc.................   1
Gi3_25119990_x101...gguucgCuucccggcgccggagc.................   1
Gi5_7296674_x101...gguucgauucccggcgccggagcgu...............   0
Gi2_9150774_x100...................uGcggcguaguauaacggcuaga...   1
Gi2_9144974_x100...cgauucccggcgccggagcguuA.............   1
Gi3_25149226_x100...................uccggcguaguauaacggcuagC..   1
Gi3_25155666_x99..Gucucacccaacagauccg..   1
Gi1_3403571_x98...gUuucgauucccggcgccggagc.................1
Gi3_25180281_x98......................ggcguaguauaacggcuaga...   0
Gi1_3407858_x97...uUguucucacccaacagauccg..1
Gi1_3410380_x97...gguucgauucccggcgccggagcU................1
Gi3_25193808_x97..uGcucacccaacagauccg..   1
Gi2_9186957_x97..guucgaCucccggcgccgg....................1
Gi1_3412885_x96...gguucgauucccggcgccAg....................1

```
Gi1_3412405_x96.....................................................................gguucgauucccggcgcc......................0
Gi3_25219584_x95...................uccggcguaguaCaacggcuaga..................................................................       1
Gi5_7369994_x94......................................................................gCucgauucccggcgccg.....................1
Gi3_25254537_x94.....................................................................gguucgauucUcggcgccggagc................       1
Gi3_25246829_x94..............................................................agauccggguucgauucc............................       0
Gi1_3431858_x93......................................................................Cuucgauucccggcgccgg....................1
Gi3_25280139_x92.................................................gGucucacccaacagauccg.......................................       1
Gi3_25293472_x91......................................................................Auucgauucccggcgccgga..................       1
Gi3_25304938_x91.....................................................................gguucgauucccggcgUcggagc................       1
Gi1_3443389_x90....................................................................Agguucgauucccggcgccgg....................1
Gi2_9277568_x89.....................................................................gAuucgauucccggcgccggagc.................1
Gi3_25328468_x89.................................................Uuucucacccaacagauccgg......................................       1
Gi5_7424212_x89............................................................acagauccggguucgauucccggcgc.......................0
Gi5_7436018_x88......................................................................gGucgauucccggcgccggagc.................1
Gi3_25349424_x88...................uccggcguaguauaacggcuagaaA................................................................       1
Gi1_3455056_x88......................................................................guucgGuucccggcgccgg....................1
Gi1_3460306_x87......................ggcguaguauaacggcuag....................................................................0
Gi1_3459958_x87.................................................gGucucacccaacagauccgg.......................................1
Gi2_9296624_x87.....................................................................ggGucgauucccggcgccggagc.................1
Gi5_7437946_x87...................uGcggcguaguauaacggcuag....................................................................1
Gi3_25380784_x86...................uccggcgCaguauaacggcuaga..................................................................       1
Gi2_9316258_x86.....................cggcguaguauaacggcua.....................................................................0
Gi1_3464465_x86.....................................................................Uguucgauucccggcgccg.....................1
Gi3_25408497_x85...................uccggcguaguauaacggcuagaauA...............................................................       1
G1a_5035827_x85...................uccggcguaguauaacggcua.....................................................................0
Gi3_25413852_x85........................................................cccaacagauccggguucgauuccc...........................       0
Gi2_9323159_x85...................Gccggcguaguauaacggc.......................................................................1
Gi3_25423855_x84.....................................................................gguucgauucccggcAccggagc................       1
Gi3_25425115_x84.............................................................cagauccggguucgauucccggcgccgg...................       0
Gi3_25418899_x84......................................................................Auucgauucccggcgccggagc................       1
Gi3_25424863_x84.....................................................................gguucgauucccggcgcAggagc................       1
G1a_5050925_x83.....................................................................Uguucgauucccggcgccggagc.................1
Gi4_435328_x83..............................................uugguucucacccaacaga............................................ 0
Gi3_25442693_x83...................Gccggcguaguauaacggcuagaa.................................................................       1
Gi5_7492827_x83.....................................................................Aguucgauucccggcgccggagc.................1
Gi2_9348290_x83....................................................cucacccaacagauccgg.......................................0
Gi5_7506722_x82.....................................................................gguucgauucccggcgccggagcguu..............0
Gi1_3484149_x82......................................................................guucAauucccggcgccgg....................1
Gi1_3491081_x81......................................................................guucgauucccggcgccAg....................1
Gi3_25483376_x81.....................................................................gguucgauucccggcgccAgagc................       1
Gi3_25469201_x81.....................................................................gguucgauucccUgcgccggagc................       1
Gi3_25483214_x81.....................................................................gguucgauucccggcgccggG..................       1
Gi3_25500696_x80...................ucUggcguaguauaacggc......................................................................       1
Gi3_25486936_x80.....................................................................ggCucgauucccggcgccgga..................       1
Gi2_9378699_x80.......................................................................uucgauucccggcgccggag..................0
Gi2_9385499_x80................................................gUuucucacccaacagauccg........................................1
Gi3_25504765_x79......................................................................guucgauucccggcgccggGg.................       1
```

```
Gi3_25536986_x78...............................................uUguucucacccaacagauccg.......................................       1
Gi1_3501301_x78...................uccggcguaguauaacggcuagaauU................................................................1
Gi1_3501457_x78......................................................................guuUgauucccggcgccgg....................1
Gi1_3506059_x78..................................................uGcucacccaacagauccg........................................1
Gi2_9414179_x77......................................................................Auucgauucccggcgccgg....................1
Gi1_3506604_x77......................................................................guucUauucccggcgccgg....................1
Gi1_3510223_x77......................................................................gGucgauucccggcgccg.....................1
Gi5_7559961_x77.........................................................................cgauucccggcgccggagcguuuc............0
Gi2_9417028_x77.....................................................................ggAucgauucccggcgccggagc.................1
Gi4_442433_x77......................................................................guucgauucccggcgccgga................... 0
Gi3_25568178_x76.....................................................................gguucgauucccggcgccggagU................       1
Gi1_3511686_x76..................................................uGcucacccaacagauccgg.......................................1
Gi3_25563010_x76................................................Cguucucacccaacagauccg.......................................       1
Gi2_9423957_x76...................uccggcguaguauaacggcu......................................................................0
Gi3_25594935_x75......................................................................guucgauuNccggcgccgga..................       1
Gi2_9445432_x75.....................cggcguaguauaacggcuaga...................................................................0
Gi2_9443182_x75.....................................................................gguuUgauucccggcgccggagc.................1
Gi5_7588434_x74.....................cggcguaguauaacggcuaga...................................................................0
Gi5_7589840_x74......................................................................guucgaCucccggcgccggagc.................1
Gi3_25620397_x73......................................................................guucgauuNccggcgccgg...................       1
Gi3_25627916_x73...................uccggcguUguauaacggcuaga..................................................................       1
Gi5_7599143_x73...................uccggcguaguauaacggcuagG...................................................................1
Gi3_25621492_x73......................................................................Auucgauucccggcgccgg...................       1
Gi2_9465654_x73...................uccggcguaguauaacggcuGga...................................................................1
Gi3_25641906_x72...................ucUggcguaguauaacgg.......................................................................       1
Gi3_25635066_x72...............................................ugguucucacccaacagauccgg......................................       0
Gi3_25652994_x72...................uccggcguNguauaacggcua....................................................................       1
G1a_5103639_x72...................uccggcguaguauaacggcuagaa..................................................................0
Gi2_9477623_x72.........................................................................cgauucccggcgccggagcguuuc............0
Gi5_7616829_x72..................................................uucucacccaacagauccggguuc...................................0
Gi5_7618053_x72......................ggcguaguauaacggcuaga...................................................................0
Gi3_25636938_x72......................ggcguaguauaacggcuag...................................................................       0
Gi3_25652418_x72.....................................................................gguucgauuUccggcgccggagc................       1
Gi1_3538184_x71......................................................................gGucgauucccggcgccggagc.................1
Gi3_25659541_x71....................................................................ggguucgauucccggcgccgga..................       0
Gi3_25661387_x71.....................................................................Cguucgauucccggcgccgga..................       1
Gi1_3539746_x71......................................................................guucgauucccggUgccgg....................1
G1a_5110245_x71.........................................................................cgauucccggcgccggagcC................1
Gi2_9490621_x71.....................................................................gCuucgauucccggcgccggagc.................1
Gi1_3536125_x71.....................................................................Uguucgauucccggcgccgga...................1
Gi1_3536906_x71...................uccggcguaguauaacggcuagaU..................................................................1
Gi2_9500831_x70.................................................guucucacccaacagauccgg.......................................0
Gi2_9499641_x70..................................................uucucacccaacagauccgg.......................................0
Gi3_25682750_x70...................uccggcguCguauaacggcuaga..................................................................       1
G1a_5117872_x70......................................................................guucgauucccggcgccg.....................0
G1a_5115352_x70...................uccggcguaguauaacggcuagaau.................................................................0
Gi3_25690730_x70......................................................................guucgGuucccggcgccgga..................       1
```

```
Gi1_3541786_x70...................Gccggcguaguauaacggcua......................................................................1
Gi4_448843_x70....................................................cucacccaacagauccggguucg................................... 0
Gi5_7641508_x70................................................gguucucacccaacagauccggguu.....................................0
Gi2_9498941_x70.....................................................................gguucgauucccggcgAcggagc..................1
Gi1_3542696_x70......................ggcguaguauaacggcua......................................................................0
Gi5_7642558_x70...................uccggcguaguauaacggcuGga....................................................................1
Gi1_3549294_x69...................Gccggcguaguauaacggcuaga....................................................................1
Gi3_25693495_x69......................................................................gCucgauucccggcgccgg....................       1
Gi2_9515471_x69...................ucUggcguaguauaacggcuaga....................................................................1
Gi3_25706398_x69......................................................................guucgauucccggcgccggG...................       1
Gi5_7645819_x69...................uGcggcguaguauaacggcuagaau..................................................................1
Gi2_9511814_x69.....................................................................gUuucgauucccggcgccgg.....................1
Gi3_25697014_x69.....................................................................ggCucgauucccggcgccggag..................       1
Gi5_7651960_x69................................................Aguucucacccaacagauccg.........................................1
Gi2_9507881_x69.....................................................................gguucgauucccggcgccggagA..................1
Gi5_7667663_x68......................................................................Auucgauucccggcgccg......................1
Gi5_7659095_x68.....................................................................gguucgauNcccggcgccggagc..................1
Gi2_9519724_x68.....................................................................gguucgauucccAgcgccggagc..................1
Gi3_25725127_x68.....................................................................Aguucgauucccggcgccg....................       1
Gi3_25713635_x68...................uccggcguaguauaacggcuUga...................................................................       1
Gi5_7664807_x68...............................................Ggguucucacccaacagauccg.........................................1
Gi5_7668269_x67.....................................................................gguucgauucccggcgccAg.....................1
Gi2_9542198_x67....................ccggcguaguauaacggcua......................................................................0
Gi3_25740233_x67...................Accggcguaguauaacggcuaga...................................................................       1
Gi2_9536503_x67.....................................................................gguucgauCcccggcgccggagc..................1
Gi5_7687168_x66........................................................................ucgauucccggcgccgga....................0
Gi2_9554639_x66...................Gccggcguaguauaacggcua......................................................................1
Gi5_7680238_x66....................................................................ggguucgauucccggcgccggagc..................0
Gi5_7691722_x66.............................................................cagauccggguucgauucccggcgccg......................0
Gi1_3561746_x66......................................................................guucgauCcccggcgccgg.....................1
Gi1_3561548_x66...............................................Ggguucucacccaacagauccg.........................................1
Gi1_3559898_x66......................................................................guucgauucccAgcgccgg.....................1
Gi2_9546257_x66.....................................................................gguucAauucccggcgccggagc..................1
Gi2_9562796_x65.................................................gGucucacccaacagauccg.........................................1
Gi5_7698366_x65..................................................................ccggguucgauucccggcgccgga....................0
Gi3_25769037_x65......................................................................gCucgauucccggcgccggagc.................       1
Gi3_25773912_x65...................uccAgcguaguauaacggcuaga...................................................................       1
Gi1_3573800_x64......................................................................guucgauucccggcgcUgg.....................1
Gi3_25791259_x64.....................................................................Cguucgauucccggcgccggag..................       1
Gi5_7709861_x64.....................cggcguaguauaacggcuag.....................................................................0
Gi2_9578297_x64.........................................................................cgauucccggcgccggagcguuu..............0
Gi1_3572008_x64..........................................................caacagauccggguucgauucccggc..........................0
Gi2_9577273_x64......................................................................Auucgauucccggcgccggagc..................1
Gi2_9571513_x64..................................................uucucacccaacagauccggguucg...................................0
Gi2_9571449_x64.................aguccggcguaguauaacggcuaga....................................................................0
Gi1_3575068_x63.....................................................................gUuucgauucccggcgccg......................1
Gi5_7726656_x63......................................................................Auucgauucccggcgccgg.....................1
```

```
Gi5_7721679_x63.....................................................................gguucgauucccggcgccggagcguuu............0
Gi3_25815684_x63......................ggcguaguauaacggcua...................................................................       0
Gi3_25805856_x63.....................................................................gguucgauucccggcgccggagcgA.............       1
Gi3_25813101_x63...................uccggcguaguauaCcggcuaga.................................................................       1
Gi3_25813920_x63......................................................................guucgauucccggcgccggCgc...............       1
Gi2_9582921_x63......................................................................guucgaCucccggcgccg....................1
Gi3_25833619_x62.....................................................................gguucgauucccggcgccgAagc...............       1
Gi3_25826303_x62......................................................................guucgGuucccggcgccggagc...............       1
Gi2_9593361_x62.....................................................................gguucgauucccggcgccggGg.................1
Gi3_25830829_x62...................uccggcguaguauaacggcuCga.................................................................       1
Gi3_25836223_x62.....................................................................gguucgauucccggcgAc....................       1
G1a_5165112_x62..................................................uucucacccaacagauccgg......................................0
Gi3_25822955_x62...................uccggcguGguauaacgg......................................................................       1
Gi3_25833123_x62.....................................................................gguucgauucccggUgcc....................       1
Gi5_7741133_x61.....................cggcguaguauaacggcuagaau................................................................0
Gi3_25845764_x61...................uccggcguaguauaacggcCa...................................................................       1
Gi1_3588041_x61......................................................................guucgauuccUggcgccgg...................1
Gi3_25842104_x61...................uccggcguaguauaGcggcua...................................................................       1
Gi1_3585662_x61......................................................cacccaacagauccgguucgauuccc............................0
Gi2_9613011_x61...................uccggcguaguauaacggcuagG..................................................................1
Gi1_3588651_x61.............................................................cagauccgguucgauucccggcgcc......................0
Gi5_7745403_x61......................ggcguaguauaacggcuagaau................................................................0
Gi2_9614597_x61.....................................................................gguucgauuccUggcgccggagc................1
Gi5_7750771_x61.................................................gGucucacccaacagauccgg......................................1
Gi5_7750649_x61................................................ggCucucacccaacagauccg.......................................1
Gi3_25847533_x61.....................................................................gguucgauucccggcgccggGg................       1
Gi3_25867609_x60.....................................................................gguucgauNcccggcgccg...................       1
Gi3_25870549_x60.....................................................................gguucgauuccAggcgccggagc...............       1
Gi5_7760416_x60...................ucUggcguaguauaacggcuaga..................................................................1
Gi5_7764076_x60..................................................uGcucacccaacagauccg.......................................1
Gi5_7768413_x59.........................................................................cgauucccggcgccggagcguuuA...........1
Gi3_25881254_x59...................uccggcguGguauaacggc.....................................................................       1
Gi2_9636740_x59......................................................................guucgauucccggcgccggagcCu..............1
Gi3_25895650_x59.....................cggcguaguauaacggcuaga.................................................................       0
Gi2_9637625_x59...................Gccggcguaguauaacgg.......................................................................1
Gi2_9631725_x59...................uccggcguNguauaacggcuaga..................................................................1
Gi3_25900812_x58..................................................Gucucacccaacagauccgg.....................................       1
Gi2_9646919_x58.....................................................................Aguucgauucccggcgccggag.................1
Gi5_7786211_x58...................uccggcguNguauaacggcuag...................................................................1
Gi5_7790155_x58...................ucUggcguaguauaacggcuag...................................................................1
Gi5_7785515_x58.....................................................................gguucgauucccggcgccggCgc................1
Gi3_25908468_x58...................uccgAcguaguauaacggcuaga.................................................................       1
Gi3_25913050_x58......................................................................gCucgauucccggcgccgga.................       1
Gi1_3611657_x57...................uGcggcguaguauaacgg.......................................................................1
Gi2_9663188_x57......................................................................guucgauuNccggcgccggagc................1
Gi2_9655550_x57...............................................uUguucucacccaacagauccg.......................................1
Gi3_25956726_x56.....................................................................Aguucgauucccggcgccgg..................       1
```

```
Gi2_9671918_x56..........................................................................Uauucccggcgccggagc..................1
Gi3_25940150_x56......................................................................guucgauucccggcgccggagcA................       1
Gi2_9670070_x56.......................................................................uucgauucccggcgccgga....................0
Gi3_25954822_x56.....................................................................gguucgaCucccggcgccgga...................       1
Gi3_25948550_x56......................................................................guCcgauucccggcgccgg....................       1
Gi2_9670686_x56......................................................................gCucgauucccggcgccggagc..................1
Gi3_25942054_x56.....................................................................gguucgauucccggcgccggaAc.................       1
Gi3_25976215_x55...................uccggcguNguauaacgg........................................................................       1
Gi5_7824359_x55......................................................................gCucgauucccggcgccgg.....................1
Gi5_7823094_x55...................uGcggcguaguauaacgg.........................................................................1
Gi3_25969945_x55.....................................................................gguucgauucccggcgcUggag..................       1
Gi1_3622888_x55...................uGcggcguaguauaacggcuag.....................................................................1
Gi2_9702216_x54.....................................................................gguucgauucccggcAccggagc..................1
Gi3_25983306_x54.........................................................................cUauucccggcgccggagc.................       1
Gi2_9697464_x54..................................................Gucucacccaacagauccg.........................................1
Gi1_3627210_x54...................uccggcguGguauaacggcuag.....................................................................1
Gi3_25997127_x53...................uccggcguaguGuaacggcua.....................................................................       1
Gi3_26015306_x53..................Cuccggcguaguauaacggcua.....................................................................       1
Gi3_26016048_x53.....................................................................gguucgauucccggAgccggagc.................       1
Gi3_26000095_x53.....................cggcguaguauaacggcuagaau.................................................................       0
Gi3_26033432_x52......................................................................Cuucgauucccggcgccgga...................       1
Gi1_3633854_x52......................................................................guucgauucccggcAccgg.....................1
Gi3_26030988_x52...............................................Ggguucucacccaacagauccg........................................       1
Gi3_26031092_x52........................................................................Gcgauucccggcgccggagc.................       1
Gi3_26032808_x52.........................................................................cgauucccggcgccggagcguuucA...........       1
Gi5_7874053_x51.....................................................................gguucgauucccggcgccggagcA.................1
Gi5_7864261_x51.....................................................................ggCucgauucccggcgccggagc..................1
Gi3_26052100_x51.....................................................................gguucgauucccgAcgccggagc.................       1
Gi1_3640695_x51......................................................................guucgauucccggcgcAgg.....................1
Gi3_26049448_x51......................................................................Cuucgauucccggcgccgg....................       1
Gi5_7872115_x51.................................................guCcucacccaacagauccg.........................................1
Gi3_26076736_x50...................uccggcguNguauaacggc.......................................................................       1
Gi3_26077436_x50.....................................................................gguucgauucccggcgccggagcU................       1
Gi3_26067836_x50...............................................ugguucucacccaUcagauc..........................................       1
Gi3_26077036_x50......................................................................guucgauuNccggcgccggagc.................       1
Gi3_26058286_x50.....................................................................gCuucgauucccggcgccggag..................       1
Gi3_26057686_x50.....................................................................gguuGgauucccggcgccggagc.................       1
Gi3_26069236_x50.....................................................................Cguucgauucccggcgccg.....................       1
Gi2_9747145_x50.....................................................................gguucgauucccggcgcAggagc..................1
Gi1_3642664_x50......................................................................guucgauucUcggcgccgg.....................1
Gi3_26064286_x50.....................................................................gguucgauNcccggcgccgg....................       1
Gi3_26057186_x50................................................Aguucucacccaacagauccg........................................       1
Gi3_26079272_x49......................................................................guucgGuucccggcgccgg....................       1
Gi5_7897160_x49..........................................................................Uauucccggcgccggagc..................1
Gi3_26081820_x49.....................................................................gAuucgauucccggcgccggag..................       1
Gi1_3647644_x49................................................................auccggguucgauucccggcgccgga....................0
Gi5_7894955_x49.........................guaguauaacggcuagaau..................................................................0
```

```
Gi1_3652936_x49.....................................................................gguucgauCcccggcgccgg.....................1
Gi5_7894759_x49...................uccggcguaguauaacAgcuaga....................................................................1
Gi2_9757944_x49......................................................................guucgauucccggcgccAga....................1
Gi2_9755445_x49.....................................................................gguucgauucccggcgccggagcCu................1
Gi1_3647301_x49.....................................................................gguucgauNcccggcgccgg.....................1
Gi3_26081134_x49...................uccggcguaguauGacggcuaga...................................................................        1
Gi2_9772973_x48.....................................................................gguucgCuucccggcgccggagc..................1
Gi3_26116112_x48...................uccggcguaguauaacAgc.......................................................................        1
Gi1_3657785_x48.....................................................................Aguucgauucccggcgccgg.....................1
Gi3_26103488_x48...................uccggcguaguauCacggcua.....................................................................        1
Gi2_9773741_x48.....................................................................Aguucgauucccggcgcc.......................1
Gi3_26097632_x48...................uccggcguaguauaacAgcua.....................................................................        1
Gi2_9777389_x48......................................................................guCcgauucccggcgccgg.....................1
Gi2_9775181_x48...................uccggcguaguauaacAgcuaga....................................................................1
Gi1_3653081_x48......................................................................guucgauucccggcgccggagcA.................1
Gi3_26101520_x48...................uccggcguaguauaUcggcuaga...................................................................        1
Gi3_26110784_x48...................uccggcguaguCuaacggcuaga...................................................................        1
Gi3_26113280_x48.....................................................................gguucgauCcccggcgccgga...................        1
Gi2_9784714_x47.....................................................................gguucgauucccggcgccggagU..................1
Gi2_9784902_x47......................................................................guucgauucccggcgccggCgc..................1
Gi2_9786124_x47...................Gccggcguaguauaacggcuag.....................................................................1
Gi3_26130456_x47.....................................................................gguucgGuucccggcgccggag..................        1
Gi3_26125662_x47.................................................gGucucacccaacagauccgg.......................................        1
Gi5_7923906_x47.....................................................................gguucgauucccCgcgccggag...................1
Gi1_3662555_x47......................................................................guucgauucccggcgUcgg.....................1
Gi3_26153973_x46....................................................................ggguucgauucccggcgccg.....................        0
Gi3_26142795_x46...................uGcggcguaguauaacggcuag....................................................................        1
Gi5_7931489_x46...................uGcggcguaguauaacggc........................................................................1
Gi2_9796478_x46......................................................................guucgauucccggcgccggGgc..................1
Gi2_9805034_x46.....................................................................gguucgauucccggUgccggagc..................1
Gi3_26153651_x46.....................................................................gguucgauuUccggcgccggag..................        1
Gi5_7940367_x46......................................................................guucgGuucccggcgccg......................1
Gi5_7938021_x46......................................................................guucgauuNccggcgccg......................1
G1a_5280140_x46.....................................................................gUuucgauucccggcgccggagc..................1
Gi3_26152501_x46.....................................................................gguucgauCcccggcgccggag..................        1
Gi5_7937975_x46......................................................................Cuucgauucccggcgccgg.....................1
Gi3_26145785_x46...................uccggcguaguauaGcggc.......................................................................        1
Gi3_26161378_x45...................uccggcguaguauCacgg........................................................................        1
Gi2_9812634_x45...................uccggcguaguauaacggcuagC....................................................................1
Gi2_9809439_x45........................................................................Gcgauucccggcgccggagc..................1
Gi5_7946502_x45......................................................................guCcgauucccggcgccgg.....................1
Gi2_9814704_x45.....................................................................gguucgauucccggcgcUggagc..................1
Gi2_9809574_x45.....................................................................gguucgauucccgAcgccggagc..................1
Gi3_26169523_x45...................uccggcguaguauaacggcuG.....................................................................        1
Gi2_9825942_x44.....................................................................gguucgauucccggcgccggagcA.................1
Gi1_3679485_x44......................................................................guGcgauucccggcgccgg.....................1
Gi2_9830694_x44...................uccggcguaguauaGcggcuaga....................................................................1
```

```
Gi3_26181005_x44...................uccggcguaguauaacggcuagU...................................................................        1
Gi3_26186901_x44.....................................................................gguucgauucccAgcgccgga...................        1
Gi5_7956443_x44..................................................Cucucacccaacagauccg........................................1
G1a_5301606_x43.....................................................................Uguucgauucccggcgccggag..................1
Gi5_7971012_x43...................uccggcguNguauaacggcuaga...................................................................1
Gi5_7971399_x43...................uccggcguaguauaacggcCaga...................................................................1
Gi2_9841797_x43......................................................................guCcgauucccggcgccggagc.................1
Gi2_9846570_x43...................uccggcguaguauaacggcCaga...................................................................1
Gi5_7973936_x43......................................................................guucgauuNccggcgccgg....................1
Gi2_9855499_x42...................Gccggcguaguauaacggcuagaau.................................................................1
Gi3_26228227_x42...................uccgUcguaguauaacggcua.....................................................................        1
Gi2_9855625_x42...................uccggUguaguauaacggcuagaa..................................................................1
Gi5_7984822_x42......................................................cacccaacagauccggguucga.................................0
Gi2_9851257_x42...................................................................cggguucgauucccggcgccgga...................0
Gi5_7989610_x42.....................................................................gguucgauucccggcgccggagcgA...............1
Gi1_3688259_x42...............................................ugguucucNcccaacagauccgg.......................................1
Gi2_9858523_x42.....................................................................gguucgauucccggcgccAgagc.................1
Gi5_7990408_x42...................uccggcguaguauaacgAcua.....................................................................1
Gi2_9849745_x42......................ggcguaguauaacggcuaga...................................................................0
Gi2_9852685_x42.....................................................................gguucgauucUcggcgccggagc.................1
Gi2_9856885_x42...................uccggcguGguauaacggc.......................................................................1
Gi5_7979992_x42...................uGcggcguaguauaacggcua.....................................................................1
Gi3_26226589_x42...................uGcggcguaguauaacggcuagaau.................................................................        1
Gi3_26228521_x42......................................................................guCcgauucccggcgccggagc.................        1
Gi3_26229739_x42...................uccggcguaguauaGcgg........................................................................        1
Gi5_7996774_x41.................aguccggcguaguauaacggcuagaaugg...............................................................0
Gi3_26252492_x41.....................................................................gguucgauucccggcgccggUgc.................        1
Gi5_7996569_x41.....................................................................gguucgauNcccggcgccg.....................1
Gi1_3695766_x41......................................................................guAcgauucccggcgccgg....................1
Gi3_26241340_x41...................Cccggcguaguauaacggcua.....................................................................        1
Gi3_26256018_x41...................uccggcguagCauaacggcua.....................................................................        1
Gi5_7993658_x41.................................................guucucacccCacagauccg........................................1
Gi5_7993863_x41.....................................................................Aguucgauucccggcgccg.....................1
Gi2_9863452_x41......................................................................guucgaCucccggcgccggag..................1
Gi5_7998250_x41................................................gguucucacccaacGgauccg........................................1
Gi5_8004564_x41................................................gguucucacccCacagauccg........................................1
Gi5_8000628_x41...................Cccggcguaguauaacggcuag....................................................................1
Gi3_26260364_x41................................................................auccggguucgauucccggcgccggagc.................        0
Gi1_3695561_x41....................ccggcguaguauaacggcu......................................................................0
Gi3_26266650_x40...................uUcggcguaguauaacggc.......................................................................        1
Gi3_26275650_x40...................uUcggcguaguauaacgg........................................................................        1
Gi3_26276090_x40......................................................................guucgauucccggcgccAga...................        1
Gi3_26282010_x40...................uccggAguaguauaacggcuaga...................................................................        1
Gi3_26263370_x40...............................................uUguucucacccaacagauc..........................................        1
Gi1_3698477_x40......................................................................guucgauucccgAcgccgg....................1
Gi3_26263930_x40.....................................................................gCuucgauucccggcgccgga...................        1
Gi3_26276010_x40.....................................................................gguucgauAcccggcgccggagc.................        1
```

```
Gi2_9877330_x40...............................................Ggguucucacccaacagauccg........................................1
Gi1_3697317_x40.....................................................................gUuucgauucccggcgccgga...................1
Gi5_8014854_x40...................uccggcguaguauaacggcCag....................................................................1
Gi1_3704321_x39.............................................guugguucucacccaacagauccgggu.....................................0
Gi3_26299059_x39......................................................................Cuucgauucccggcgccggagc................       1
Gi3_26305026_x39..............................................................agauccggguucgauucccggcgccggagcC...............       1
Gi2_9893236_x39......................................................................Cuucgauucccggcgccgg....................1
Gi3_26305572_x39.....................................................................gguucgauucAcggcgccggagc................       1
Gi5_8026280_x39......................................................................guucgauucccggcAccgg....................1
Gi3_26283693_x39.....................................................................gguucgaCucccggcgccggag.................       1
Gi3_26327082_x38...................uUcggcguaguauaacggcua....................................................................       1
Gi2_9909383_x38......................................................................guucgGuucccggcgccggagc.................1
Gi1_3714126_x38...................uccggcguNguauaacggcuag....................................................................1
Gi2_9905051_x38.................................................................uccggguucgauucccggcgccgga...................0
Gi2_9911093_x38......................................................................guucgauuNccggcgccgg....................1
Gi3_26311426_x38.....................................................................gguucgauucccggcgccggCg.................       1
Gi1_3709984_x38.....................................................................gUuucgauucccggcgccggag..................1
Gi1_3713404_x38...................uGcggcguaguauaacggcua.....................................................................1
Gi2_9913069_x38.....................................................................gguucgauNcccggcgccggag..................1
Gi3_26326702_x38......................................................................guucgauucccggUgccgg...................       1
Gi5_8031125_x38......................................................................guucgauucccggcgccA.....................1
Gi5_8033481_x38.......................................................acccaacagauccggguucgauucc.............................0
Gi3_26320318_x38.....................................................................gguucgauucccggcAccgg...................       1
Gi3_26318608_x38.....................................................................gguucgGuucccggcgccgga..................       1
Gi2_9912081_x38......................................................................guucgaCucccggcgccgga...................1
Gi3_26320546_x38...................uccggUguaguauaacggcua....................................................................       1
Gi3_26349402_x37.....................................................................gguucgauucccggcgccgAag.................       1
Gi1_3717732_x37......................................................................guucgauucccggcgAcgg....................1
Gi3_26332530_x37...................uccggcAuaguauaacggcuaga..................................................................       1
Gi2_9921627_x37...................uUcggcguaguauaacggcuaga...................................................................1
Gi5_8046744_x37.....................................................................gguucgauNcccggcgccgga...................1
Gi3_26345147_x37.....................................................................ggAucgauucccggcgccgga..................       1
Gi5_8052664_x37.....................................................................Cguucgauucccggcgccggagc.................1
Gi3_26331161_x37......................................................................Auucgauucccggcgccggag.................       1
Gi1_3718472_x37...................uccggcguGguauaacggcua.....................................................................1
Gi5_8043118_x37................................................gguucCcacccaacagauccg........................................1
Gi1_3715031_x37......................................................................guucgauucccggcgccggag..................0
G1a_5352502_x37...................Gccggcguaguauaacggcuaga...................................................................1
Gi2_9916965_x37.....................................................................Aguucgauucccggcgccg.....................1
Gi2_9924698_x37.....................................................................gguucgauucccggcgAc......................1
Gi2_9923107_x37.....................................................................gguucgauNcccggcgcc......................1
Gi3_26335638_x37.....................................................................gguucgauucccggcgccA....................       1
Gi5_8063536_x36...................uccggcguaguauaGcggcuag....................................................................1
Gi3_26361443_x36.....................................................................gguCcgauucccggcgccggag.................       1
Gi3_26352767_x36.....................................................................gguucgauucccggUgccgga..................       1
Gi3_26372855_x36.....................................................................gguucgauucccggcgccggC..................       1
Gi3_26353271_x36..................................................uGcucacccaacagauccgg......................................       1
```

```
Gi3_26359031_x36.....................................................................gguucgauucccggcAccgga...................       1
Gi3_26352083_x36......................................................................guucgauucccggcgccggGgc.................       1
Gi5_8065660_x36................................................Uguucucacccaacagauccgg........................................1
Gi2_9929187_x36.....................................................................gguucgauucccggAgccggagc..................1
Gi2_9939159_x36.....................................................................gguucgauucccggcgccgAagc..................1
Gi3_26358887_x36...................uccgUcguaguauaacgg........................................................................       1
Gi1_3721587_x36....................................................cucacccaacagauccggguucgauucc..............................0
Gi5_8061016_x36................................................gguucucaNccaacagauccg.........................................1
Gi5_8058424_x36....................................................cucacccCacagauccgg........................................1
Gi2_9939771_x36.....................................................................gguucgauucccggcgccggaAc..................1
Gi3_26378230_x35...................uccggcguaguauaacggcuagaaC.................................................................       1
Gi5_8069594_x35...................uccggcguaguauaacAgcuag.....................................................................1
Gi5_8068229_x35......................................................................guucgaCucccggcgccgga....................1
Gi1_3728352_x35...................uccggcguaguauaGcgg.........................................................................1
Gi2_9948054_x35...................uGcggcguaguauaacggcua......................................................................1
Gi5_8076769_x35...................uccggcguaguGuaacggcuaga....................................................................1
Gi3_26377950_x35......................................................................guCcgauucccggcgccgga...................       1
Gi3_26393525_x35......................................................................guucgauucccAgcgccgg....................       1
Gi3_26381415_x35..............................................................agauccggguucgauucccggcgccggagc.................       0
G1a_5365600_x35......................................................................guucgauucccggcgccgga....................0
Gi3_26394540_x35......................................................................guucAauucccggcgccgg....................       1
Gi5_8072429_x35......................................................................Cuucgauucccggcgccg......................1
Gi3_26394120_x35.................................................................uccggguucgauucccggcgccg.....................       0
G1a_5364060_x35...............................................ugguucucacccaacagauccg.........................................0
Gi5_8077714_x35......................................................................guucUauucccggcgccgg.....................1
Gi3_26382465_x35...................uccggcguaAuauaacggcuaga...................................................................       1
Gi1_3728247_x35......................................................................guucgauucccggAgccgg.....................1
Gi2_9949979_x35.....................................................................gguuGgauucccggcgccggagc..................1
Gi3_26384355_x35.....................................................................ggCucgauucccggcgccg.....................       1
Gi1_3735733_x34...................ucUggcguaguauaacgg.........................................................................1
Gi1_3735189_x34....................ccggcguaguauaacggcuag.....................................................................0
Gi5_8085535_x34......................................................................guCcgauucccggcgccg......................1
Gi5_8085025_x34....................................................cGcacccaacagauccgg........................................1
Gi5_8088731_x34................................................gguucucacccGacagauccg.........................................1
Gi3_26414972_x34......................................................................guucgauucccggUgccgga...................       1
Gi3_26398108_x34..................................................uCcucacccaacagauccg........................................       1
Gi5_8090499_x34...................uccggcguGguauaacggcuag.....................................................................1
Gi2_9967459_x34.....................................................................gguucgauuUccggcgccggagc..................1
Gi5_8088935_x34.................................................guucucacNcaacagauccg.........................................1
Gi1_3735325_x34.................................................guucucacccCacagauccg.........................................1
Gi2_9958755_x34.....................................................................Aguucgauucccggcgccgga....................1
Gi5_8084005_x34...................uccggcguaguauaacgAcuaga....................................................................1
Gi2_9956477_x34.....................................................................gguucgauucccUgcgccggagc..................1
Gi1_3737467_x34...................uccggcguNguauaacgg.........................................................................1
Gi2_9965147_x34.....................................................................gguucgauNcccggcgccg......................1
Gi5_8085263_x34...................ucUggcguaguauaacggcua......................................................................1
Gi3_26413068_x34.....................................................................gguucgauucccAgcgccggag..................       1
```

```
Gi1_3743568_x33..................................................uucucaccNaacagauccg........................................1
Gi5_8097372_x33.....................................................................gguucgauucccggcgccggGgc.................1
Gi1_3742776_x33................................................Aguucucacccaacagauccg........................................1
Gi2_9978951_x33.....................................................................gguucgauucccggcgccggCg..................1
Gi3_26426809_x33.....................................................................Aguucgauucccggcgcc......................       1
Gi5_8099451_x33......................................................................guucgauucccggcgcUg.....................1
Gi5_8105061_x33.....................................................................gguucgGuucccggcgccggagc.................1
Gi3_26424631_x33...................uccgUcguaguauaacggc.......................................................................       1
Gi3_26436082_x33...................uccggcguaguauUacggcuaga...................................................................       1
Gi5_8099352_x33...................uUcggcguaguauaacggcuag....................................................................1
Gi3_26426050_x33......................................................................guucUauucccggcgccgg....................       1
Gi3_26434465_x33......................................................................guucgauucccggcgccA.....................       1
Gi3_26424136_x33.....................................................................gguCcgauucccggcgccgga...................       1
Gi3_26428129_x33.....................................................................gguucgauuccUggcgccgga...................       1
Gi3_26424796_x33...................uccggcguaguauaUcggcua.....................................................................       1
Gi5_8099088_x33...................uUcggcguaguauaacggcuaga...................................................................1
Gi3_26436412_x33...................ucUggcguaguauaacggcuag....................................................................       1
Gi2_9982581_x33.....................................................................gguucgauAcccggcgccggagc.................1
Gi3_26442022_x33......................................................................guucgauucccggcgccAg....................       1
Gi3_26430076_x33........................cguaguauaacggcuaga...................................................................       0
Gi2_9980106_x33.....................................................................gguucgauucccggcgUcggagc.................1
Gi1_3738717_x33.................................................................uccggguucgauucccggcgccgga...................0
Gi3_26460286_x32.....................................................................gAuucgauucccggcgccgga...................       1
Gi3_26446270_x32............................................................acagauccggguucgauucccggcgccgg....................       0
Gi3_26466494_x32.....................................................................ggGucgauucccggcgccggag..................       1
Gi1_3745344_x32......................................................................guucgauucccgUcgccgg....................1
Gi3_26468638_x32..................................................uucucacccaCcagauccg........................................       1
Gi3_26455070_x32...................Cccggcguaguauaacggc.......................................................................       1
Gi1_3744160_x32...................uccggcguaguauaacAgcuag....................................................................1
Gi5_8116710_x32...................uccggcguaguGuaacggcuag....................................................................1
Gi3_26457918_x32.....................................................................ggGucgauucccggcgccgga...................       1
Gi3_26452766_x32...................ucAggcguaguauaacggcuaga...................................................................       1
Gi3_26457758_x32.....................................................................gguucgauuAccggcgccggagc.................       1
Gi5_8114374_x32...................uccggcguaguauaGcgg........................................................................1
Gi2_9993405_x32...................uGcggcguaguauaacggc.......................................................................1
Gi5_8111366_x32....................................................cucacccaNcagauccgg.......................................1
Gi2_9997213_x32...................uccggcguGguauaacggcuaga...................................................................1
Gi2_9996477_x32...................uccggcguaguGuaacggcuaga...................................................................1
Gi3_26469278_x32.....................................................................gguucgauucccggcgcUgga...................       1
Gi3_26450590_x32......................................................................guuUgauucccggcgccgga...................       1
Gi1_3747552_x32...............................................ugguucucacccaacagaucc.........................................0
Gi5_8108550_x32.....................................................................gAuucgauucccggcgccggagc.................1
Gi3_26477902_x31..............................................................agauccggguucgauucccggcgccggag..................       0
Gi4_490729_x31...................uccggcguaguauaacggcuag.................................................................... 0
Gi3_26478336_x31......................................................................guucUauucccggcgccgga...................       1
Gi1_3754020_x31..................................................uuUucacccaacagauccg........................................1
Gi1_3754578_x31....................................................................ggguucgauucccggcgccgga...................0
```

```
Gi5_8127401_x31...................uUcggcguaguauaacggcuagaau.................................................................1
Gi3_26471795_x31...................uccggcguaguGuaacgg........................................................................       1
Gi2_9999863_x31......................................................................Cuucgauucccggcgccggagc.................1
Gi5_8130315_x31......................................................................guucgauucccggcAccg.....................1
Gi5_8120085_x31...................uccggcguaguauaGcggcuaga...................................................................1
Gi3_26487574_x31.....................................................................gguuAgauucccggcgccggagc.................       1
Gi3_26480072_x31.....................................................................gguucgaCucccggcgccgg....................       1
Gi3_26475019_x31......................................................................guucgauCcccggcgccgga...................       1
Gi2_10002994_x31...................uccggcguaguauCacggcuaga...................................................................       1
Gi5_8118814_x31...................uccggcguGguauaacggcua.....................................................................1
Gi5_8126192_x31.....................................................................gguucgaCucccggcgccggagc.................1
Gi3_26472911_x31......................................................................guuUgauucccggcgccgg....................       1
Gi2_10012480_x31...................Cccggcguaguauaacggcuaga...................................................................       1
Gi2_10011860_x31......................................................................guucgauucccggcgccgC....................       1
Gi3_26491728_x31......................................................................guucgauuccUggcgccgg....................       1
Gi3_26473655_x31......................................................................guucgauCcccggcgccgg....................       1
Gi2_10008326_x31.................................................guucucacccaacagauccgggu.....................................       0
Gi5_8137855_x30......................................................................guucgauuccUggcgccg.....................1
Gi1_3758915_x30................................................................auccggguucgauucccggcgccgg....................0
Gi1_3758705_x30....................................................cucacccaacagauccggguucga.................................0
Gi3_26512770_x30......................................................................guucgauucccAgcgccgga...................       1
Gi5_8140135_x30................................................gguucucGcccaacagauccg........................................1
Gi2_10028005_x30.....................................................................Cguucgauucccggcgcc......................       1
Gi1_3756845_x30...........................................................aacagauccggguucgauucccggcgccgg....................0
Gi3_26513970_x30...................uccggcguaguauUacgg........................................................................       1
Gi5_8136295_x30...................ucUggcguaguauaacggc.......................................................................1
Gi5_8141005_x30...................uccggcguaguauaacggcuagaaug................................................................0
Gi2_10027315_x30...................Gccggcguaguauaacggcuagaa..................................................................       1
Gi1_3756935_x30...................uccggcguaguauaGcggcuag....................................................................1
Gi2_10024765_x30..................................................uucucacccaacGgauccg........................................       1
Gi5_8133235_x30...................uccggcguaguauaacggcuagaCu.................................................................1
Gi1_3761195_x30...................Gccggcguaguauaacggc.......................................................................1
Gi5_8140915_x30...................uccggcguaguauaacggcuagC...................................................................1
Gi5_8141425_x30.....................................................................gguucgauucccggcgccggC...................1
G1a_5399946_x30.....................................................................Uguucgauucccggcgccg.....................1
Gi3_26516520_x30......................................................................guucgauucccggcgUcgga...................       1
Gi2_10018675_x30......................................................................guucgauucccAgcgccggagc.................       1
Gi3_26506200_x30.....................................................................gguucAauucccggcgccgga...................       1
Gi5_8153061_x29...................uccggcguaguauaacggUuag....................................................................1
Gi5_8154772_x29................................................gguCcucacccaacagauccg........................................1
Gi5_8143752_x29...................ucUggcguaguauaacggcuagaau.................................................................1
Gi1_3767858_x29......................................................................guucgauucccggcgcccCg...................1
Gi5_8151785_x29...................uccggcguGguauaacggcuaga...................................................................1
Gi1_3764610_x29......................................................................guucgauucccggcgccggagcgA...............1
Gi5_8148972_x29.................................................gCucucacccaacagauccg........................................1
Gi5_8152887_x29......................................................................guucgauuccUggcgccgg....................1
Gi2_10032948_x29.............................................................cagauccggguucgauucccggcgccggag..................       0
```

```
Gi1_3764407_x29.......................................................acccaacagauccggguucgauucccg...........................0
Gi5_8144825_x29......................................................................guucgGuucccggcgccgg....................1
Gi3_26527030_x29......................................................................guucgauucccggcgcUgga..................       1
Gi3_26537209_x29......................................................................guucgauuNccggcgccggag.................       1
Gi3_26523260_x29...................uccggcgCaguauaacgg.......................................................................       1
Gi2_10029874_x29.....................................................................gguCcgauucccggcgcc.....................       1
Gi5_8145608_x29...................ucUggcguaguauaacgg........................................................................1
Gi5_8150915_x29...................................................Gcucacccaacagauccg........................................1
Gi3_26539645_x29.......................................................................Gucgauucccggcgccggagc................       1
Gi3_26538456_x29...................uccggcguaguauaacgAcua....................................................................       1
Gi3_26538688_x29.....................................................................gguucgauucccggcgGcggagc................       1
Gi3_26539384_x29...................uccggcguagCauaacggc......................................................................       1
Gi2_10036109_x29...................uccggcguGguauaacggcua....................................................................       1
Gi1_3767597_x29......................................................................guucgauuUccggcgccgg....................1
Gi5_8148740_x29.......................................................acccaacagauccggguucgauuccc............................0
Gi5_8143462_x29...................uccgUcguaguauaacggcuaga...................................................................1
Gi3_26528828_x29......................................................................guucAauucccggcgccgga..................       1
Gi3_26542980_x29.....................................................................gguucgauucccggcgAcggag.................       1
Gi3_26554693_x28.....................................................................gguucgauucccggcgAcgga..................       1
Gi5_8160949_x28.....................................................................gguucgauNcccggcgccggag..................1
Gi5_8158877_x28..................................................uucucaccNaacagauccg........................................1
Gi2_10045729_x28......................................................................guucgauuccUggcgccggagc................       1
Gi2_10046429_x28.....................................................................gguucgauucAcggcgccggagc................       1
Gi3_26545733_x28...................Cccggcguaguauaacgg.......................................................................       1
Gi3_26556541_x28......................................................................guucgauucccggcAccgga..................       1
Gi3_26559173_x28...................uccggcguaguauCacggc......................................................................       1
Gi1_3770940_x28.....................................................................Uguucgauucccggcgccggag..................1
Gi2_10055053_x28.....................................................................gguucgauucccggcgccggG..................       1
Gi5_8166913_x28.....................................................................gguucgauucccggcgAc......................1
Gi5_8162657_x28.....................................................................gCuucgauucccggcgccggagc.................1
Gi5_8156945_x28......................................................................guucgauucccggcgUcgg....................1
Gi2_10057461_x28...................uccggcguaguauaacggcuaAa..................................................................       1
Gi3_26554441_x28....................................................cucacccaacagauccggguucgauucc............................       0
Gi3_26547861_x28......................................................................guucgGuucccggcgccggag.................       1
Gi2_10045757_x28.....................................................................gguuAgauucccggcgccggagc................       1
Gi3_26545705_x28...................uccggcguaguauaacggcuGg...................................................................       1
Gi3_26555953_x28......................................................................guucgauuccUggcgccggagc................       1
Gi5_8163133_x28................................................gguucucacccaCcagauccg........................................1
Gi3_26587578_x27.....................................................................gguucgauucccggcgccAga..................       1
Gi3_26588604_x27.....................................................................gguucgauucccggcAccggag.................       1
Gi3_26578533_x27....................................................cucacccaacagauccggguucgauu..............................       0
Gi3_26593599_x27...................ucUggcguaguauaacggcuagaau................................................................       1
Gi5_8180396_x27...................uccggcguaguauaacAgcua.....................................................................1
Gi1_3779598_x27...................uccggcguaguauaacggcCag....................................................................1
Gi2_10059158_x27......................................................................guucUauucccggcgccgg...................       1
Gi2_10066853_x27......................Cgcguaguauaacggcuaga..................................................................       1
Gi3_26577156_x27......................................................................guucgaGucccggcgccggagc................       1
```

```
Gi3_26590710_x27...........................................................aacagauccggguucgauucccggcgccgga....................	0
Gi5_8171027_x27...................uccggcguaguauaaUggcuaga....................................................................1
Gi3_26585094_x27.....................................................................ggAucgauucccggcgccggag...................	1
Gi1_3776601_x27......................................................................guucgaCucccggcgccgga....................1
Gi3_26592168_x27......................................................................guucgauucccAgcgccggagc..................	1
Gi3_26582178_x27...................ucUggcguaguauaacggcuagaa...................................................................	1
Gi2_10059887_x27...................ucUggcguaguauaacggc........................................................................	1
Gi3_26586768_x27......................Ugcguaguauaacggcuaga....................................................................	1
Gi2_10067744_x27......................................................................guucgauucccggcAccgg.....................	1
Gi1_3780354_x27.....................................................................gguucgauucccggcgccgU.....................1
Gi3_26582799_x27...................................................Gcucacccaacagauccg.........................................	1
Gi3_26588037_x27...................uccggcguaguauaacggcuGgaa...................................................................	1
Gi5_8170649_x27......................................................................guucgauCcccggcgccg......................1
Gi5_8178101_x27................................................gguucucacccaGcagauccg.........................................1
G1a_5426177_x27.....................................................................Uguucgauucccggcgccgg.....................1
Gi5_8169947_x27...............................................................gauccggguucgauucccggcgccgga....................0
Gi5_8186200_x26......................................................................guucgauucccggUgccg......................1
Gi3_26620993_x26...................uccggcguaguaCaacgg.........................................................................	1
Gi3_26600531_x26............................................................acagauccggguucgauucccggcgccggagc..................	0
Gi3_26619537_x26...................uccggcguaguauaGcggcuag.....................................................................	1
Gi1_3786287_x26...................uccggcguaguGuaacggcuaga....................................................................1
Gi3_26621123_x26.......................................................................Gucgauucccggcgccgga....................	1
Gi1_3785741_x26..................................................uucucacccaCcagauccg.........................................1
Gi2_10076679_x26......................................................................guucgGuucccggcgccgg.....................	1
Gi1_3781945_x26......................................................................guucgauucccggcgccgA.....................1
Gi1_3784623_x26...............................................ugguucucGcccaacagauccgg........................................1
Gi1_3784987_x26...................uGcggcguaguauaacggcuaga....................................................................1
Gi5_8194208_x26.....................................................................gguucgauucccggUgcc.......................1
Gi5_8189398_x26...................uccggcguaguauCacggcuaga....................................................................1
Gi5_8183444_x26......................................................................guGcgauucccggcgccg......................1
Gi5_8186590_x26......................................................................guucgauucccAgcgccg......................1
Gi5_8187812_x26...................uccgUcguaguauaacggcuag.....................................................................1
Gi3_26601935_x26.....................................................................gguucgauucUcggcgccggag...................	1
Gi1_3786209_x26...................uccggUguaguauaacggcuag.....................................................................1
Gi3_26596267_x26......................................................................guucgauucccggcgAcgg.....................	1
Gi3_26619511_x26...................uccggcguaguauaaUggcua......................................................................	1
Gi5_8191868_x26....................................................Gucacccaacagauccgg........................................1
Gi2_10082815_x26..................................................uGcucacccaacagauccg.........................................	1
Gi5_8196534_x25...................uccggcguaguauaacgAcuag.....................................................................1
Gi1_3790881_x25......................................................................gAucgauucccggcgccgg.....................1
Gi3_26624893_x25......................................................................guucgauucUcggcgccg......................	1
Gi3_26621768_x25.....................................................................gguucgCuucccggcgccgga....................	1
Gi3_26646318_x25...................uccggcguaguauaacAgcuagaau..................................................................	1
Gi1_3790031_x25.................................................guucucacNcaacagauccg.........................................1
Gi2_10104647_x25......................................................................guucgauucccggcgccAg.....................	1
Gi2_10097697_x25...................uccggcguaguauaacgAcuaga....................................................................	1
Gi5_8197809_x25.....................................................................Aguucgauucccggcgccgga....................1
```

```
Gi2_10100072_x25.....................................................................gguucgauNcccggcgccgga...................       1
Gi5_8203184_x25.....................................................................gguuUgauucccggcgccggagc..................1
Gi3_26629968_x25.................................................Uuucucacccaacagauccggg......................................       1
Gi3_26633193_x25.....................................................................gguucgauNcccggcgcc......................       1
Gi5_8199184_x25...................uccggcguaguauaacggcuagaaA..................................................................1
Gi5_8198434_x25.................................................guucucacccaacagGuccg.........................................1
Gi3_26645368_x25......................................................................guucgauucccggcAccgg....................       1
Gi1_3792481_x25..................................................uuUucacccaacagauccgg........................................1
G1a_5444687_x25.....................................................................gUuucgauucccggcgccggag...................1
Gi5_8200709_x25..................................................uuUucacccaacagauccg.........................................1
Gi3_26640343_x25......................................................................guucgauucGcggcgccggagc.................       1
Gi5_8196184_x25...................uccggcguNguauaacggcuagaau..................................................................1
Gi5_8201009_x25.................................................guucucacccaCcagauccg.........................................1
Gi3_26629143_x25...................uccggAguaguauaacggcua.....................................................................       1
Gi3_26647618_x25.....................................................................gguuUgauucccggcgccggag..................       1
Gi2_10090447_x25......................................................................guucgauuccUggcgccgg....................       1
Gi2_10095222_x25......................................................................guuUgauucccggcgccggagc.................       1
Gi5_8201084_x25................................................Cguucucacccaacagauccg.........................................1
G1a_5450987_x25.....................................................................Uguucgauucccggcgccgga....................1
Gi3_26640368_x25...................uccggcguaguGuaacggc.......................................................................       1
Gi5_8207059_x25......................................................................guucgauucccggcgccAg.....................1
Gi2_10100297_x25...........................................................aacagauccggguucgauucccggcgccg.....................       0
Gi2_10097647_x25.....................................................................gguucgauucccggUgcc......................       1
Gi3_26638018_x25.....................................................................gguucgauuccAggcgccgg....................       1
Gi3_26649193_x25.....................................................................gguucgauucccggUgccggag..................       1
Gi5_8196384_x25................................................gguucucacUcaacagauccg.........................................1
Gi1_3790131_x25......................................................................guucgaCucccggcgccg......................1
Gi2_10120123_x24...................uccggcguaguauaacggUuaga...................................................................       1
Gi3_26663600_x24...................uccggcguGguauaacggcuag....................................................................       1
Gi1_3794750_x24...................ucUggcguaguauaacggcuag.....................................................................1
Gi5_8218152_x24...................uccggcguGguauaacgg.........................................................................1
Gi3_26666672_x24..............................................................aUauccggguucgauuccc............................       1
Gi3_26671712_x24.....................................................................gguuUgauucccggcgccgga...................       1
Gi5_8219064_x24...............................................uUguucucacccaacagauccgggu......................................1
Gi3_26674952_x24.....................................................................gguucgauucccggcgUcgga...................       1
Gi3_26658896_x24......................................................................guucgauucccggcgUcgg....................       1
Gi3_26675624_x24......................................................................guucgauCcccggcgccggagc.................       1
Gi3_26660288_x24................................................gguucucaNccaacagauccg........................................       1
Gi1_3795806_x24...................uccggcguaguauaacggcuGga....................................................................1
Gi1_3795614_x24...............................................ugguucucNcccaacagauccg.........................................1
Gi3_26663672_x24.....................................................................gguucAauucccggcgccggag..................       1
Gi5_8209344_x24...................uccggcguaguaCaacggcuaga....................................................................1
Gi3_26675528_x24.....................................................................gguucgauucccggcgccggUg..................       1
Gi5_8207304_x24...................uccggcguaguauaacggcuGgaau..................................................................1
Gi3_26675888_x24...................uccgCcguaguauaacggcuaga...................................................................       1
Gi1_3795686_x24...................uccggcguaguauaacggcuagG....................................................................1
Gi3_26661824_x24................................................gguCcucacccaacagauccg........................................       1
```

```
Gi3_26664320_x24...................uUcggcguaguauaacggcuag...................................................................        1
Gi5_8217048_x24......................Ugcguaguauaacggcuag....................................................................1
Gi5_8207976_x24...................Gccggcguaguauaacggcuagaa..................................................................1
Gi1_3798686_x24.....................................................................gguucgaCucccggcgccgg....................1
Gi3_26668208_x24...................uccggcguaguauGacgg.......................................................................        1
Gi2_10111627_x24...................uccggcguNguauaacggc......................................................................        1
Gi5_8218584_x24......................................................................guucgauucccggcgccggagcguuucc...........0
Gi5_8208528_x24................................................gguucucacccaacagGuccg........................................1
Gi5_8207880_x24...................uccggcguagCauaacggcuaga...................................................................1
Gi3_26665520_x24.....................................................................gguucgaCucccggcgccg....................        1
Gi2_10115803_x24......................................................................guucgauucccggUgccggagc................        1
Gi3_26679536_x24...................uccggcguagCauaacgg.......................................................................        1
Gi3_26650424_x24...................ucGggcguaguauaacggcuaga..................................................................        1
Gi3_26682400_x23...................uccggcguNguauaacggcuagaa.................................................................        1
Gi1_3800485_x23......................................................................guucgauucccggcgccggaU..................1
Gi5_8225756_x23.................................................Auucucacccaacagauccg........................................1
Gi5_8223433_x23..................................................Gucucacccaacagauccgg.......................................1
Gi5_8221616_x23.................................................guucucacccaGcagauccg........................................1
Gi5_8230356_x23...................uccggcguaguauaacggcuGg....................................................................1
Gi4_499827_x23.....................................................................Uguucgauucccggcgccgg.................... 1
Gi5_8229942_x23.....................................................................Cguucgauucccggcgccg.....................1
Gi5_8223571_x23......................................................................guucgauucccggcgccggagcgA...............1
Gi5_8232656_x23...................uccggcguaguauaacggcuagaaugg...............................................................0
Gi3_26697948_x23.....................................................................gguucgauucUcggcgccgga..................        1
Gi3_26708827_x23..................................................uuUucacccaacagauccg.......................................        1
Gi3_26689323_x23...................uccggcguaguauaacggUua....................................................................        1
Gi3_26687230_x23.....................................................................ggGucgauucccggcgccg....................        1
Gi5_8227343_x23.....................................................................gguucgauucccAgcgccggagc.................1
Gi5_8225825_x23...................uccggUguaguauaacggcuag....................................................................1
Gi3_26688150_x23...................uccggcguaguauaacAg.......................................................................        1
Gi3_26701122_x23......................................................................guucgauucccgAcgccggagc................        1
Gi1_3804694_x23.................................................guucucacccCacagauccgg.......................................1
Gi3_26697833_x23.....................................................................gguucgauCcccggcgccg....................        1
Gi2_10128003_x23......................................................................guucUauucccggcgccggagc................        1
Gi2_10126186_x23...................uccggcguNguauaacggcua....................................................................        1
Gi5_8226906_x23................................................Uguucucacccaacagauc..........................................1
Gi3_26708482_x23................................................gguucucacccaGcagauccg.......................................        1
Gi3_26687529_x23.....................................................................gguAcgauucccggcgccggag.................        1
Gi5_8220144_x23......................................................................guuUgauucccggcgccgg....................1
Gi3_26686057_x23...................uccggcguNguauaacggcuag...................................................................        1
Gi2_10122253_x23...................uccggcguaguauaacggcuGgaau................................................................        1
Gi4_499091_x23.....................................................................gUuucgauucccggcgccgg.................... 1
Gi1_3805131_x23.................................................guCcucacccaacagauccg........................................1
Gi5_8231069_x23......................................................................guucUauucccggcgccg.....................1
Gi5_8228263_x23.....................................................................gguucgauccUggcgccggagc..................1
Gi2_10130648_x23.................................................guucucacccCacagauccg.......................................        1
Gi2_10137065_x23......................................................................gCucgauucccggcgccgg...................        1
```

```
Gi3_26684470_x23...................uccggcguaguauaacggcuagaCu.................................................................       1
Gi1_3799979_x23......................................................................guucgCuucccggcgccgg.....................1
Gi5_8222398_x23......................................................................guucgauucccggcgccggagcA.................1
Gi5_8227987_x23...................uccggcguNguauaacggcua......................................................................1
Gi5_8228700_x23......................................................................guucAauucccggcgccg......................1
Gi1_3812227_x22......................................................................guucgauucccggcgccgC.....................1
Gi3_26718166_x22......................................................................guAcgauucccggcgccgga...................       1
Gi5_8248083_x22................................................gguucucacccaacagCuccg.........................................1
Gi3_26718562_x22.................................................guucucacccCacagauccg........................................       1
Gi3_26709784_x22......................................................................guAcgauucccggcgccgg....................       1
Gi3_26713986_x22..................................................uuUucacccaacagauccgg.......................................       1
Gi5_8238293_x22...................uccggcguaguauCacggcuag.....................................................................1
Gi5_8234575_x22.....................................................................Aguucgauucccggcgcc.......................1
Gi5_8242539_x22......................ggcguaguauaacggcuagaa...................................................................0
Gi5_8240427_x22.....................................................................gguucgauNcccggcgccgg.....................1
Gi3_26719574_x22................................................ggCucucacccaacagauccg........................................       1
Gi2_10138452_x22......................................................................guuUgauucccggcgccgg....................       1
Gi3_26727098_x22......................................................................guucAauucccggcgccggagc.................       1
Gi1_3808399_x22..................................................uucucaccUaacagauccgg........................................1
Gi3_26737284_x22...................uccggcguaguauaacUgcuaga...................................................................       1
Gi3_26729452_x22...................uccggcguaguauaacggcuC.....................................................................       1
Gi5_8243045_x22....................ccggcguaguauaacggcuagaau..................................................................0
Gi3_26716120_x22......................................................................gCucgauucccggcgccggag..................       1
Gi3_26730772_x22.....................................................................gguucgauucccUgcgccgga...................       1
Gi1_3807937_x22................................................Uguucucacccaacagauccgg........................................1
Gi2_10140696_x22......................................................................guGcgauucccggcgccgg....................       1
Gi3_26740848_x22.....................................................................gguCcgauucccggcgccg.....................       1
Gi3_26725954_x22.....................................................................gguucgauucccggcgcAgga...................       1
Gi3_26717066_x22.....................................................................gguucgGuucccggcgccgg....................       1
Gi3_26728792_x22.....................................................................gguNcgauucccggcgccggagc.................       1
Gi1_3813437_x22.....................................................................gAuucgauucccggcgccgg.....................1
Gi3_26727450_x22....................................................cGcacccaacagauccgg.......................................       1
Gi3_26722500_x22...................uccggcguaguUuaacggcuaga...................................................................       1
Gi3_26728088_x22......................................................................guucgauucccggcgcAgga...................       1
Gi2_10139948_x22......................................................................guucgauucccggcgccggG...................       1
Gi2_10151036_x22........................................................................uGgauucccggcgccggagc.................       1
Gi1_3808971_x22...................uUcggcguaguauaacggcuag.....................................................................1
Gi1_3811083_x22..................................................uucucacccaCcagauccgg........................................1
Gi3_26711566_x22......................................................................guucgauucccAgcgccggag..................       1
Gi3_26721092_x22................................................Uguucucacccaacagauccgg.......................................       1
Gi3_26726042_x22...................uccggUguaguauaacgg........................................................................       1
Gi3_26729320_x22...................uccggcguaguauaacggAuaga...................................................................       1
Gi3_26726196_x22.....................................................................Cguucgauucccggcgccgg....................       1
Gi3_26725778_x22...................uccggcguaguauaacggGuaga...................................................................       1
Gi3_26725008_x22...................uccggcguaguaCaacggcua.....................................................................       1
Gi2_10142500_x22.....................cggcguaguauaacggcuagaau.................................................................       0
Gi2_10147758_x22.....................................................................Cguucgauucccggcgccggag..................       1
```

```
Gi3_26731828_x22.....................................................................gguCcgauucccggcgccgg....................       1
Gi1_3810995_x22...................uccggUguaguauaacgg........................................................................1
Gi3_26717924_x22.......................................................................uGcgauucccggcgccgga...................       1
Gi5_8253804_x21................................................gguucucacccaacagaCccg........................................1
Gi2_10171897_x21......................................................................guucgauucccggcgUcggagc.................       1
Gi5_8253741_x21......................................................................guucgauucccggcgccgC....................1
Gi5_8257437_x21.....................................................................gguucgauCcccggcgccggagc.................1
Gi1_3820035_x21..................................................uucucacccaacagauccggguucgau................................0
Gi3_26769219_x21...................uccggcguGguauaacggcuagaau.................................................................       1
Gi2_10157743_x21.....................................................................gguucgauuccAggcgccggagc.................       1
Gi2_10155790_x21...................uccggcguGguauaacgg........................................................................       1
Gi2_10163098_x21.....................................................................gguucgauucccggcgcUg.....................       1
Gi5_8250171_x21.................................................guucucacccGacagauccg........................................1
Gi1_3818985_x21....................................................Gucacccaacagauccgg.......................................1
G1a_5481220_x21.................................................Uuucucacccaacagauccg........................................1
Gi5_8252838_x21.....................................................................gguCcgauucccggcgccggagc.................1
Gi3_26764158_x21...................uccggcgCaguauaacggcua.....................................................................       1
Gi3_26755716_x21...............................................................Uauccggguucgauuccc............................       1
Gi3_26754267_x21......................................................................guGcgauucccggcgccgga...................       1
Gi5_8258970_x21.....................................................................ggGucgauucccggcgccggagc.................1
Gi3_26768988_x21...................uccggcguaguGuaacggcuag....................................................................       1
Gi5_8262477_x21...................uccggcguaguauaacggcCagaau.................................................................1
Gi5_8255211_x21.....................................................................gguucAauucccggcgccggagc.................1
Gi2_10172401_x21...................uGcggcguaguauaacgg........................................................................       1
Gi1_3820434_x21...................uUcggcguaguauaacgg........................................................................1
Gi5_8255085_x21......................................................................guucgaCucccggcgccggag..................1
Gi5_8251956_x21.....................................................................ggAucgauucccggcgccggagc.................1
Gi1_3818649_x21..................................................uucucacccaacGgauccgg.......................................1
Gi2_10171771_x21.....................................................................gguucgauucccAgcgccggag..................       1
Gi1_3813861_x21..................................................uucucacccGacagauccgg.......................................1
Gi3_26758068_x21...................uccggcguaguauaacAgcuag....................................................................       1
Gi5_8254014_x21.....................................................................gguucgauucccggcgccggagA.................1
Gi3_26764221_x21...................uccggcguaguauaaUgg........................................................................       1
Gi3_26755359_x21...................uccggcguaguauaaAggcuaga...................................................................       1
Gi2_10157638_x21...................uccggcguagCauaacggcuaga...................................................................       1
Gi2_10171645_x21...................uccggcguaguauaacggcA......................................................................       1
Gi3_26765145_x21.............................................................cagauccggguucgauucccggcgccggagcC................       1
Gi2_10166227_x21...................uccgUcguaguauaacggcuaga...................................................................       1
Gi1_3816633_x21...................uUcggcguaguauaacggcua.....................................................................1
Gi3_26762877_x21...................uccggcguaguaCaacggc.......................................................................       1
Gi1_3814533_x21.....................................................................gguucgauucccggcgccggaU..................1
Gi3_26742633_x21...................uccggcguNguauaacggcuagaau.................................................................       1
Gi3_26768379_x21...................uccggcguaguauaaGggcuaga...................................................................       1
Gi5_8251242_x21...................uccggcgCaguauaacggcuaga...................................................................1
Gi5_8254392_x21...............................................ugguucucacccaacagauccggguucga.................................0
G1a_5485777_x21.....................................................................gUuucgauucccggcgccg.....................1
Gi3_26767035_x21...................uccggcguaguauaacCgcuaga...................................................................       1
```

Gi5_8254728_x21..gAuucucacccaacagauccg..1
Gi1_3820980_x21...................Gccggcguaguauaacggcuagaau...1
Gi5_8270349_x20...................Cccggcguaguauaacggc...1
Gi1_3821550_x20..guucgauAcccggcgccgg....................1
Gi2_10175769_x20...Aguucgauucccggcgccgg....................   1
Gi2_10173549_x20..guucgauucccggcgccggagcA................   1
Gi3_26786165_x20...gauccggguucgauucccggcgccgga...................   0
Gi3_26780245_x20...ugguucucaccUaacagauc..   1
Gi2_10187089_x20...ggGucgauucccggcgccggag..................   1
Gi5_8276429_x20..guucAauucccggcgccgg....................1
Gi5_8271389_x20..guuUgauucccggcgccg.....................1
Gi5_8274529_x20..gCuucucacccaacagauccg..1
Gi3_26802185_x20...gguucgCuucccggcgccggag..................   1
Gi2_10183169_x20..guucgauCcccggcgccgg....................   1
Gi5_8265889_x20.................aguccggcguaguauaacggcu..0
Gi3_26801825_x20..gAucgauucccggcgccgg....................   1
Gi3_26793365_x20...................uccAgcguaguauaacggcua...   1
Gi2_10185989_x20...gguucgauucccggcgccggagcU................   1
Gi2_10189489_x20...................uccggcguaguauaacgAcua...   1
Gi1_3821370_x20...................uccggcguaguaCaacgg..1
Gi5_8271009_x20...................Cccggcguaguauaacggcuaga...1
Gi1_3827030_x20..uucucaccNaacagauccgg.......................................1
Gi1_3825530_x20...ggCucgauucccggcgccgg....................1
Gi3_26804425_x20..Ugguucgauucccggcgccggagc.................   1
Gi1_3825930_x20...................Cccggcguaguauaacgg..1
Gi3_26772925_x20......................ggcguaguauaacggcuagaau...   0
Gi1_3823770_x20...gguucgauucccggcgAcgg....................1
Gi3_26799545_x20...gguucgauucccgAcgccgga...................   1
Gi5_8272489_x20...................uccggcguaguauaacggUuaga...1
Gi2_10183269_x20...................Cccggcguaguauaacggc...   1
Gi5_8265869_x20...................uccggcCuaguauaacggc...1
Gi5_8268609_x20..guucgauucccggcgAcgg....................1
Gi2_10186169_x20...gguucgauucccggcgccggaCc.................   1
Gi1_3824130_x20...ugguucucacccaacGgauccgg.......................................1
Gi2_10181629_x20..guucgauCcccggcgccggagc.................   1
Gi3_26795625_x20...................uAcggcguaguauaacggcuaga...   1
Gi1_3825750_x20.....................cggcguaguauaacggcu..0
Gi5_8276009_x20...................Cccggcguaguauaacggcua...1
Gi3_26803925_x20...gguucgauucccggcgccAgag..................   1
Gi1_3827510_x20...Gcucacccaacagauccg..1
Gi3_26835193_x19...................uccggcguaguaGaacggcuaga...   1
Gi3_26808840_x19...gguucgauucccggcgccAg....................   1
Gi1_3830617_x19...................uccgUcguaguauaacgg..1
Gi5_8283296_x19....................ccggcguaguauaacggcu..0
Gi3_26830880_x19..Cuucgauucccggcgccggag..................   1
Gi5_8291903_x19...gguucgauucccggcAccggagc.................1
Gi5_8280085_x19...................uccggcguagCauaacgg..1

```
Gi1_3830427_x19.....................................................ucacccaacagauccggguucga..................................0
Gi2_10199340_x19......................................................................guucgauucccggcgAcgg.....................       1
Gi3_26820943_x19...................uccggUguaguauaacggcuag.....................................................................       1
Gi2_10208726_x19...................uccggcguaguauaaUggcuaga....................................................................       1
Gi5_8287020_x19......................................................................guucgauCcccggcgccgg.....................1
Gi3_26836029_x19...................uccggcguaguauaacggcuagGau..................................................................       1
Gi3_26832894_x19......................................................................guucgauucUcggcgccgg.....................       1
Gi3_26808612_x19.................Gguccggcguaguauaac...........................................................................       1
Gi3_26816193_x19...................uccggcguaguauaaUggc........................................................................       1
Gi3_26820183_x19......................................................................guucgauucccgAcgccgg.....................       1
Gi3_26821722_x19......................................................................guucgauuccUggcgccgga....................       1
Gi2_10196756_x19......................................................................guucgauucccggcgccgCagc..................       1
Gi1_3834930_x19......................................................................Auucgauucccggcgccggagc..................1
Gi2_10206731_x19.....................................................................gguucgauuAccggcgccggagc..................       1
Gi3_26806161_x19...................uccggcguaguauaacgAc........................................................................       1
Gi5_8285652_x19...................uccggcguaguauaacggcuagaGu..................................................................1
Gi2_10199530_x19......................................................................guAcgauucccggcgccggagc..................       1
Gi1_3830807_x19.................................................Auucucacccaacagauccg.........................................1
Gi2_10192918_x19...................uUcggcguaguauaacggc........................................................................       1
Gi1_3831985_x19.....................................................................gguucgauucccggcgcUgg.....................1
Gi3_26805230_x19......................................................................guCcgauucccggcgccggag...................       1
Gi1_3833676_x19...................ucUggcguaguauaacggcuaga....................................................................1
Gi3_26828410_x19.....................................................................gguucgauucccggcgAcg......................       1
Gi3_26822102_x19......................................................................guucgauucccggcgAcgga....................       1
Gi3_26819936_x19.........................................................................cUauucccggcgccggag...................       1
Gi3_26815452_x19.....................................................................gAuucgauucccggcgccg......................       1
Gi3_26810854_x19..................................................Cucucacccaacagauccg.........................................       1
Gi2_10206845_x19.....................................................................gguucgGuucccggcgcc.......................       1
Gi5_8291884_x19...................uccggcguGguauaacggc........................................................................1
Gi5_8303733_x18...................uccggcguaguaCaacggc........................................................................1
Gi1_3838809_x18................................................gUuucucacccaacagauccgg........................................1
Gi1_3841635_x18...................uccggcguaguauaacggcuGg.....................................................................1
Gi2_10224454_x18.....................................................................gguuUgauucccggcgccgga....................       1
Gi3_26859778_x18......................................................................guucgauucccggcgcUgg.....................       1
Gi3_26850778_x18.....................................................................gguucgauucccggcgccgCagc..................       1
Gi3_26843128_x18......................................................................guGcgauucccggcgccgg.....................       1
G1a_5511635_x18.....................................................................Uguucgauucccggcgcc.......................1
Gi5_8299737_x18...................uccggUguaguauaacggcua......................................................................1
Gi1_3842319_x18...................uccggcguNguauaacggcua......................................................................1
Gi2_10218010_x18.....................................................................gguucgauucccggcgcUgga....................       1
Gi1_3838593_x18..................................................uucucacccaacGgauccg.........................................1
Gi5_8297325_x18..................................................uCcucacccaacagauccg.........................................1
Gi5_8300925_x18...................uUcggcguaguauaacgg.........................................................................1
Gi1_3840465_x18......................................................................guucgauucccggcgccggG....................1
Gi3_26846368_x18.....................................................................gguuUgauucccggcgccg......................       1
Gi5_8294553_x18...................uccAgcguaguauaacggcuag.....................................................................1
Gi2_10223176_x18......................................................................guucAauucccggcgccggagc..................       1
```

```
Gi1_3843435_x18...................uccgUcguaguauaacggcuag.....................................................................1
Gi3_26859400_x18.....................................................................gguucgauucccggcgccUgagc.................        1
Gi2_10224562_x18......................................................................guucgauucccggcgcUgg....................        1
Gi3_26844622_x18.......................................................................uGcgauucccggcgccggagc.................        1
Gi5_8296209_x18................................................ggAucucacccaacagauccg.........................................1
Gi2_10215526_x18......................................................................guucgauucccggcgUcgg....................        1
Gi1_3837495_x18..................................................Cucucacccaacagauccg.........................................1
Gi5_8302311_x18......................................................................guucgauucccggcgUcg......................1
Gi2_10218298_x18..............................................................agauccggguucgauucccggcgccggagcC................        1
Gi3_26865556_x18...................uccggcguaguCuaacggcua.....................................................................        1
Gi3_26842750_x18......................................................................guucgauucccggUgccggagc.................        1
Gi4_506352_x18.....................................................................gguucgauucccggcgccggagcC................ 1
Gi2_10226272_x18...................uccggcgCaguauaacggcuaga...................................................................        1
Gi5_8297415_x18...................uccggcguaguauaacggcuCga....................................................................1
Gi3_26839870_x18................................................Uguucucacccaacagauccgggu.....................................        1
Gi1_3842463_x18.......................................................................Gucgauucccggcgccgg.....................1
Gi3_26866942_x18......................................................................guucgauucccggAgccgg....................        1
Gi5_8296497_x18..................................................uucucacccaCcagauccg.........................................1
Gi3_26870074_x18......................................................................guucgauuccUggcgccggag..................        1
Gi3_26854252_x18.......................gcguaguauaacggcuaga...................................................................        0
Gi2_10223590_x18...................uccggcguaguauaacggcuagaaugguugg...........................................................        0
Gi1_3843075_x18...............................................ugguCcucacccaacagauccgg........................................1
Gi3_26873890_x18........................................................................uGgauucccggcgccggagc.................        1
Gi1_3843633_x18..................................................uucCcacccaacagauccg.........................................1
Gi5_8315104_x17...................uccggcguaguauaacggcuagaaugguu..............................................................0
Gi3_26882054_x17.....................................................................gguucgauucccggGgccggagc.................        1
Gi3_26889806_x17..................................................uucucacccaacagaCccg........................................        1
Gi1_3850973_x17..............................................................aUauccggguucgauuccc.............................1
Gi5_8315223_x17......................................................................gAucgauucccggcgccgg.....................1
Gi4_506824_x17...................uccggcguaguauaacggc....................................................................... 0
Gi5_8311296_x17...................uccggcguaguauaGcggc........................................................................1
Gi2_10238093_x17...................uGcggcguaguauaacggcuag....................................................................        1
Gi2_10246083_x17......................................................................guucgauucccggcgccAgagc.................        1
Gi1_3850905_x17......................................................................Auucgauucccggcgccgga....................1
Gi3_26876937_x17...................uccggcguaguauaacggcCagaau.................................................................        1
Gi1_3853115_x17...................ucUggcguaguauaacggcua......................................................................1
Gi4_508099_x17......................................................................Uuucgauucccggcgccgg.................... 1
Gi2_10232534_x17.....................................................................gguucgauucccggcAcc......................        1
Gi1_3847658_x17...................uccggcguaguauaacggcuaU.....................................................................1
Gi1_3848746_x17......................................................................guucgaCucccggcgccggagc..................1
Gi5_8313914_x17...................Gccggcguaguauaacggcu.......................................................................1
Gi3_26882904_x17......................................................................guucgauucccggcgccUga...................        1
Gi2_10232636_x17...................uccggUguaguauaacggcuaga...................................................................        1
Gi5_8308066_x17...................uccggcguaguauaaUggcuag.....................................................................1
Gi5_8310769_x17..........................................................caacagauccggguucgauucccggcgccg......................0
Gi5_8319592_x17...................uccgUcguaguauaacgg.........................................................................1
Gi1_3850395_x17.................................................guucucacccaacagGuccg.........................................1
```

```
Gi5_8316906_x17...................uccggcguaguauaacggcuCg....................................................................1
Gi2_10238671_x17......................................................................guucgauucUcggcgccggagc................       1
Gi5_8310820_x17........................................................................guucgauuUccggcgccgg..................1
Gi3_26896844_x17......................................................................guucgauuUccggcgccgg...................       1
Gi3_26877923_x17......................................................................guucgauAcccggcgccgg...................       1
Gi2_10229746_x17.....................................................................gguucgauucccggcgAcg....................       1
Gi3_26912263_x17....................................................................Ugguucgauucccggcgccggag.................       1
Gi3_26898238_x17...................uccggcguagAauaacggcuaga..................................................................       1
Gi1_3847335_x17...............................................uAguucucacccaacagauccgg.......................................1
Gi5_8308746_x17.................................................guucucGcccaacagauccg........................................1
Gi5_8319660_x17................................................gUuucucacccaacagauccgg.......................................1
Gi5_8311126_x17...................uccggcguaguauaGcggcuagaau.................................................................1
Gi1_3848661_x17................................................ggCucucacccaacagauccg........................................1
Gi3_26909917_x17......................................................................guucgauucccggcgcAgg...................       1
Gi3_26882003_x17.....................................................................gguucgGuucccggcgccg....................       1
Gi3_26878246_x17.....................................................................gguucgUuucccggcgccggagc................       1
Gi3_26893376_x17......................................................................guucgauucccggcgccgAa..................       1
Gi2_10230664_x17.....................................................................gguucgauucccggcgccggUgc................       1
Gi3_26878059_x17..........................................................................gGuucccggcgccggagc................       1
Gi1_3851194_x17..................................................uCcucacccaacagauccg........................................1
Gi2_10247120_x17...................uccggcguUguauaacggcuaga..................................................................       1
Gi2_10239453_x17......................................................................Auucgauucccggcgccgga..................       1
Gi3_26903763_x17......................................................................guucgauucccggcgccggagcgA..............       1
Gi2_10240048_x17...................uccggcguaguauaacAgc......................................................................       1
Gi2_10243414_x17......................................................................guucgauucUcggcgccgg...................       1
Gi1_3846808_x17......................................................................guucgauuAccggcgccgg....................1
Gi1_3854747_x17.....................................................................gguucgauucccggUgccgg....................1
Gi5_8318266_x17..............................................................aUauccggguucgauuccc............................1
Gi1_3848525_x17......................................................................guucgauucccggcgcGgg....................1
Gi3_26892560_x17................................................gguucucacccaacagCuccg.......................................       1
Gi3_26907112_x17.......................gcguaguauaacggcuagaa.................................................................       0
Gi5_8321479_x17...................uccggcguNguauaacggc.......................................................................1
Gi2_10238654_x17.....................................................................ggCucgauucccggcgcc.....................       1
Gi3_26896538_x17...................uccggcgCaguauaacggc......................................................................       1
Gi5_8319694_x17......................................................................guucgauucccggcgcUgg....................1
Gi3_26901825_x17.....................................................................gguucgauucccggAgccgga..................       1
Gi3_26931325_x16...................uccggcguaguauaacgCcuaga..................................................................       1
Gi5_8337504_x16.....................................................................Aguucgauucccggcgccggag..................1
Gi3_26921565_x16.....................................................................ggAucgauucccggcgccg....................       1
Gi3_26926957_x16..................................................uucucaccNaacagauccg.......................................       1
Gi5_8325104_x16...................uccggcguagCauaacggcuag....................................................................1
Gi3_26919517_x16...................................................................cUgguucgauucccggcgccgga..................       1
Gi1_3862977_x16......................................................................guucgauuccAggcgccgg....................1
Gi1_3861793_x16.....................................................................Uguucgauucccggcgcc......................1
Gi1_3856657_x16......................................................................gCucgauucccggcgccgga...................1
Gi5_8327520_x16......................................................................guAcgauucccggcgccg.....................1
Gi5_8324224_x16...................uccggcguaguauaacAgc.......................................................................1
```

```
Gi2_10258249_x16.....................................................................gguucgaCucccggcgccg.....................       1
Gi2_10256553_x16...................uccggcCuaguauaacggcuaga...................................................................       1
Gi3_26943453_x16.....................................................................gguucgauCcccggcgccgg....................       1
Gi3_26919165_x16.....................................................................ggCucgauucccggcgccgg....................       1
Gi3_26930301_x16..................................................uucucacccaacagGuccg........................................       1
Gi3_26921389_x16...................uGcggcguaguauaacggcuagaa..................................................................       1
Gi3_26935261_x16.....................................................................gguucgauucccgAcgccggag..................       1
Gi3_26945757_x16...................uccggcguaguauaGcggcuagaau.................................................................       1
Gi1_3857777_x16.....................................................................gguucgCuucccggcgccgg....................1
Gi2_10269849_x16......................................................................guucgauucccggcgGcggagc.................       1
Gi1_3858497_x16................................................gguucucacccaCcagauccg........................................1
Gi3_26942061_x16................................................gUuucucacccaacagaucc.........................................       1
Gi1_3858289_x16.....................................................................gguucgGuucccggcgccgg....................1
Gi1_3861857_x16.................................................gCucucacccaacagauccg........................................1
Gi5_8333488_x16...................uccggcguaguauaacggcuaAa...................................................................1
Gi1_3857521_x16...................uccggcguGguauaacgg........................................................................1
Gi2_10263561_x16.....................................................................gguucgauuccGggcgccggagc.................       1
Gi3_26942173_x16...................uccAgcguaguauaacgg........................................................................       1
G1a_5537341_x16.....................................................................gUuucgauucccggcgccgga...................1
Gi3_26950013_x16................................................gguucucacccaacGgauccg........................................       1
Gi2_10268377_x16......................................................................guucgauucccggcgAcggagc.................       1
Gi2_10267337_x16.......................................aUaaugguugguucucacc...................................................       1
Gi3_26922189_x16................................................Uguucucacccaacagaucc.........................................       1
Gi3_26946093_x16......................................................................guucgauucccggcgccggC...................       1
Gi3_26932605_x16.....................................................................gguucgauucccggcgccggaA..................       1
Gi3_26942973_x16................................................gguucucacccCacagauccg........................................       1
Gi3_26941533_x16......................................................................guucgauucUcggcgccgga...................       1
Gi1_3861985_x16......................................................................guucgauucAcggcgccgg....................1
Gi3_26920877_x16......................................................................guucgauAcccggcgccgga...................       1
Gi1_3858945_x16.....................................................................gguucgauucccgAcgccg.....................1
Gi5_8335344_x16...................uccAgcguaguauaacgg........................................................................1
Gi5_8335584_x16......................................................................guucgauucccggUgccgg....................1
Gi2_10259961_x16...................uccgUcguaguauaacggc.......................................................................       1
Gi1_3861281_x16..................................................uucucGcccaacagauccg........................................1
Gi5_8333760_x16.....................................................................ggCucgauucccggcgccg.....................1
Gi2_10252361_x16.....................................................................gguucgUuucccggcgccggagc.................       1
Gi3_26916285_x16.....................................................................gguucgauuccGggcgccggagc.................       1
Gi1_3855969_x16..................................................uucucacccaacagGuccgg.......................................1
Gi2_10266009_x16...................uccggcguaguauaacggcuCga...................................................................       1
Gi3_26925405_x16.....................................................................gguucgauucccggAgccg.....................       1
Gi5_8331232_x16......................................................................Auucgauucccggcgccgga...................1
Gi2_10260585_x16................................................gguucucaNccaacagauccg........................................       1
Gi3_26936845_x16................................................gguucCcacccaacagauccg........................................       1
Gi3_26948301_x16.....................................................................gAuucgauucccggcgccgg....................       1
Gi3_26935277_x16......................................................................gAucgauucccggcgccgga...................       1
Gi2_10256521_x16.....................................................................gguucgauucccggcgccAga...................       1
Gi1_3860049_x16..................................................uCcucacccaacagauccgg.......................................1
```

```
Gi2_10263641_x16......................................................................guAcgauucccggcgccgg...................        1
Gi2_10267577_x16.....................................................................Cguucgauucccggcgccg....................        1
Gi3_26944653_x16...................uccggcguaguauaacggcuGgaau................................................................        1
Gi1_3862593_x16...............................................ugguucucacccaCcagauccgg.......................................1
Gi2_10257705_x16......................................................................guucgauucccggcgcUggagc................        1
Gi5_8323808_x16......................................................................guucgauucUcggcgccg.....................1
Gi5_8323536_x16................................................gguucucacccaacagaucUg........................................1
Gi2_10261465_x16.....................................................................gguucgauucccAgcgcc.....................        1
Gi5_8335312_x16...................uccggcguNguauaacgg........................................................................1
Gi5_8336528_x16.....................................................................Aguucgauucccggcgccgg....................1
Gi2_10261641_x16...................uGcggcguaguauaacggcuagaau................................................................        1
Gi3_26947453_x16...................uccggcguCguauaacgg.......................................................................        1
Gi1_3860929_x16.................................................guucucacccaacGgauccg........................................1
Gi3_26942765_x16......................................................................gAucgauucccggcgccggagc................        1
Gi3_26985805_x15......................................................................guucgauuUccggcgccggag.................        1
Gi5_8352632_x15...................uccggcguaguauCacgg........................................................................1
Gi5_8338577_x15......................................................................guucgauucccAgcgccgg....................1
Gi3_26955550_x15...................Gccggcguaguauaacggcu.....................................................................        1
Gi3_26972620_x15.....................................................................gguucgauuccAggcgccgga..................        1
Gi1_3869582_x15...................uccggcguaguauaacggcuG.....................................................................1
Gi2_10281595_x15...................uccAgcguaguauaacggcuaga..................................................................        1
Gi1_3872252_x15................................................gguucucacccGacagauccg........................................1
Gi2_10287895_x15......................................................................guucAauucccggcgccgg...................        1
Gi5_8345297_x15................................................ggGucucacccaacagauccg........................................1
Gi2_10279780_x15...................ucUggcguaguauaacggcua....................................................................        1
Gi2_10287565_x15......................................................................guGcgauucccggcgccggagc................        1
Gi5_8340467_x15......................................................................guucgauucccggcgcccCg...................1
Gi3_26966170_x15...................uccggcguaguGuaacggcuagaau................................................................        1
Gi2_10292530_x15......................................................................guucgauucccggcAccggagc................        1
Gi2_10277185_x15...................uccggcguaguaCaacggcuaga..................................................................        1
Gi3_26957575_x15......................................................................Auucgauucccggcgccg....................        1
Gi5_8351162_x15....................................................cCcacccaacagauccgg.......................................1
Gi5_8351762_x15................................................gUuucucacccaacagauc..........................................1
Gi3_26977105_x15.............................................................cagauccggguucgauucccggcgccggagc................        0
Gi2_10287145_x15......................................................................guucgauucccggcgccgA...................        1
Gi3_26986555_x15...............................................Ggguucucacccaacagauc.........................................        1
Gi1_3867872_x15.................................................guucucacNcaacagauccgg.......................................1
Gi3_26961715_x15...................uccAgcguaguauaacggc......................................................................        1
Gi2_10275640_x15......................................................................gCucgauucccggcgccg....................        1
Gi1_3870467_x15...................uccggcguaguauaacAg........................................................................1
Gi3_26954470_x15.....................................................................gguucgauuGccggcgccggagc................        1
Gi3_26952685_x15.....................................................................gguucgauuUccggcgccgga..................        1
Gi3_26992975_x15...................uUcggcguaguauaacggcuagaau................................................................        1
Gi3_26978710_x15............................................................acagauccggguucgauucccggcgccgga..................        0
Gi5_8344892_x15...................................................ucucacccGacagauccgg.......................................1
Gi2_10274605_x15......................................................................guCcgauucccggcgccg....................        1
Gi3_26983810_x15.....................................................................gguucgauucccUgcgccggag.................        1
```

```
Gi2_10294195_x15..................................................uucucacccaCcagauccg........................................       1
Gi4_510572_x15...................uccggcguaguauaacggcuagaau................................................................. 0
Gi1_3870362_x15.....................................................................ggGucgauucccggcgccgg....................1
Gi1_3868877_x15...................uccggcguaguauaacgAcuag....................................................................1
Gi3_26977270_x15................................................gguucucacUcaacagauccg........................................       1
Gi5_8354912_x15.....................................................................gguucgauucccggcgcUggagc.................1
Gi5_8341367_x15...................uccggcguagCauaacggc.......................................................................1
G1a_5544824_x15......................................................................Uuucgauucccggcgccggagc.................1
Gi2_10273030_x15......................................................................guucgGuucccggcgccgga...................       1
Gi3_26968030_x15..................................................uucucacccaacGgauccg........................................       1
Gi3_26953060_x15......................................................................guucUauucccggcgccggagc.................       1
Gi2_10291435_x15...............................................ugguucucNcccaacagauccg........................................       1
Gi3_26988265_x15................................................gguucucacccGacagauccg........................................       1
Gi5_8349707_x15......................................................................guucgauucccggAgccgg....................1
Gi3_26981320_x15.....................................................................ggCucgauucccggcgcc......................       1
G1a_5552324_x15.....................................................................gUuucgauucccggcgcc......................1
Gi3_26960050_x15......................................................................guucgauuAccggcgccggagc.................       1
Gi5_8350472_x15...................uUcggcguaguauaacggcua.....................................................................1
Gi3_26987620_x15...................uccggcguaguauaacgAcuagaau.................................................................       1
Gi1_3870317_x15.....................................................................gCuucgauucccggcgccgg....................1
Gi3_26953450_x15......................................................................guucgauucccggcgcccCg....................       1
Gi2_10291720_x15......................................................................guucgauAcccggcgccggagc.................       1
Gi5_8344007_x15.....................................................................gguucgauucccggUgccggagc.................1
Gi2_10284595_x15......................................................................guucgauucccggUgccgg....................       1
Gi1_3866552_x15...............................................uggCucucacccaacagauccgg.......................................1
Gi5_8339057_x15.....................................................................gguucgauucccAgcgccgga...................1
Gi2_10288225_x15...............................................uUguucucacccaacagaucc.........................................       1
Gi2_10293895_x15.....................................................................gguucgGuucccggcgccggag..................       1
Gi3_26988595_x15.................................................guucucacNcaacagauccg........................................       1
Gi1_3867737_x15................................................Cguucucacccaacagauccg........................................1
Gi3_26957860_x15..................................................uucCcacccaacagauccg........................................       1
Gi3_26975290_x15......................................................................guucgauucUcggcgccggagc.................       1
Gi3_26986465_x15......................................................................guucgauucccggcgcAggagc.................       1
Gi1_3872102_x15...................uccggcguaguGuaacggcuag....................................................................1
Gi2_10291690_x15.....................................................................ggCucgauucccggcgccggag..................       1
Gi2_10281325_x15.....................................................................gAuucgauucccggcgccggag..................       1
Gi3_26966755_x15.....................................................................gCuucgauucccggcgccg.....................       1
Gi5_8346857_x15.....................................................................gguucgauNcccggcgcc......................1
Gi3_26969215_x15......................................................................guAcgauucccggcgccggagc.................       1
Gi5_8338427_x15..................................................uucucacccaacGgauccg........................................1
Gi1_3865502_x15...................uccggcgCaguauaacgg........................................................................1
Gi1_3867422_x15...................uccggcguaguauaacggcC......................................................................1
Gi3_26998135_x14..................................................uucucaccNaacagauccgg.......................................       1
Gi3_27000277_x14.......................................................................uucgauucccggcgccggCgc.................       1
Gi3_27036425_x14...............................................ugguucucacccaacagauccggguucga.................................       0
Gi2_10305815_x14.......................gcguaguauaacggcuagaa..................................................................       0
Gi2_10296631_x14......................................................................guucgauuNccggcgccg.....................       1
```

Gi3_27007347_x14...................Cccggcguaguauaacggcuagaau...   1
Gi5_8368683_x14...................uccgUcguaguauaacggcua...1
Gi2_10313039_x14..guucgauucccggcgcUg.....................   1
Gi3_27018169_x14..guuUgauucccggcgccggagc.................   1
Gi1_3882092_x14...guucucacccaacagGuccgg.......................................1
Gi5_8371357_x14...................uccggcguaguauaGcggcua...1
Gi2_10316595_x14...gAuucgauucccggcgcc......................   1
Gi3_27015145_x14...gguucgauAcccggcgccggag..................   1
Gi2_10308545_x14...................uccggUguaguauaacggcua...   1
Gi3_27005527_x14...................uccUgcguaguauaacggcuaga...   1
Gi5_8363685_x14...gguucgauucccggcgccggagU.................1
Gi5_8366779_x14...................uccggcguaguaCaacggcuag..1
Gi2_10313193_x14..guucgauucccggcgccA.....................   1
Gi3_27023615_x14..uucucGcccaacagauccg..   1
Gi1_3880440_x14...ugguucucacccaacagaCccg..1
Gi3_26999759_x14...gguucgauucccggUgccg.....................   1
Gi3_27021445_x14...................uccggcguaguauaGcggcuagaa..   1
Gi3_27005163_x14..guucgauucccggcgccgAagc.................   1
Gi3_27001719_x14...gAuucgauucccggcgcc......................   1
Gi3_27008271_x14...guucucacccaacagauccggguucgau................................   0
Gi5_8363433_x14..Cuucgauucccggcgccgga...................1
Gi2_10304737_x14...................uccggcguaguauaacggcuagGau...   1
Gi3_27010399_x14...ggNucgauucccggcgccggagc.................   1
Gi3_27016139_x14...gguuGgauucccggcgccgga...................   1
Gi1_3875372_x14...gguucgauucccggcAccgg....................1
Gi5_8359135_x14...ggCucgauucccggcgccgga...................1
Gi5_8362635_x14...gguucgauucccggcgccggGg..................1
Gi2_10316175_x14..guucgauucccggcgccgAagc.................   1
Gi3_27012821_x14..gCucgauucccggcgccg.....................   1
Gi5_8368865_x14...ggCucgauucccggcgcc......................1
Gi3_27000333_x14...gguucgauucccggcgcccCgagc................   1
Gi1_3874140_x14..Cucucacccaacagauccgg.......................................1
Gi3_27037237_x14..gguucucacccaCcagauccg..   1
Gi5_8363377_x14...gguucgCuucccggcgccg.....................1
Gi3_27029341_x14...uucgauucccggcgccgC....................   1
Gi3_27002881_x14...................uccNgcguaguauaacggcuaga...   1
Gi5_8356405_x14...gguucgauucccAgcgccg.....................1
Gi5_8369299_x14...................Cccggcguaguauaacgg..1
Gi3_27036271_x14...................uccggcguaguauaacggcuaUa...   1
Gi3_27015621_x14..guGcgauucccggcgccggagc.................   1
Gi3_27019345_x14..guucgauuccAggcgccgg....................   1
Gi2_10301433_x14...................uccggcguaguGuaacggc...   1
Gi5_8365687_x14...................uGcggcguaguauaacggcuagaa..1
Gi1_3878970_x14...ugguucucacccCacagauccgg.......................................1
Gi2_10308713_x14...................ucUggcguaguauaacggcuagaau...   1
Gi5_8358995_x14..gCucgauucccggcgccgga...................1
Gi1_3877304_x14..uucucacccaacagGuccg..1

G1a_5559747_x14...................uccggcguaguauaacgAcuagaau..1
Gi3_27011939_x14..uucucacccaGcagauccg...   1
Gi5_8359107_x14...................uccggcgCaguauaacggcuagaau..1
Gi3_27009573_x14..guucgauucccggcgAcggagc..................   1
Gi2_10303701_x14...................uccggcguGguauaacggcuag...   1
Gi1_3873146_x14..uucucGcccaacagauccgg..1
Gi5_8365253_x14...Cguucgauucccggcgccgga....................1
Gi5_8360045_x14...gAuucgauucccggcgccg......................1
Gi1_3873118_x14..gguucucacccaacagaCccg...1
G1a_5558613_x14..Gucucacccaacagauccg...1
Gi3_26999605_x14...gguucgauuccUggcgccgg.....................   1
Gi3_27005429_x14...gCuucgauucccggcgccgg.....................   1
Gi3_27024385_x14...................uccggcguaguauaCcgg...   1
Gi5_8365309_x14........................cguaguauaacggcuagaaugguugguuc...0
Gi3_27012527_x14...................uccggcguaguauaCcggcua..   1
Gi2_10297023_x14...gguucgauCccggcgccgga.....................   1
Gi5_8362173_x14...................uccggcguaguauaacggcCa..1
Gi1_3877780_x14...ggCucgauucccggcgccggagc..................1
Gi3_27029817_x14...................uccggcgGaguauaacggcuaga..   1
Gi5_8367703_x14...guucucacccaacCgauccg...1
Gi3_27002125_x14...gguucgauucccggcgAcgg.....................   1
Gi2_10298059_x14..guucgauuUccggcgccgg.....................   1
Gi3_27021501_x14..guGcgauucccggcgccggag...................   1
Gi3_27004211_x14...gguucgauucccggcgUcggag...................   1
Gi3_27022649_x14..guucgauucccggcgGcggagc..................   1
Gi5_8363755_x14...gguucAauucccggcgccg......................1
Gi2_10306039_x14..guucgauucccggAgccggagc..................   1
Gi5_8372561_x14..guGcgauucccggcgccgg.....................1
Gi3_27006661_x14...guCcucacccaacagauccg...   1
Gi5_8360829_x14...gguucgauucccUgcgccggagc..................1
Gi3_27006577_x14..uCcucacccaacagauccgg..   1
Gi3_27014207_x14...................uccggcguUguauaacggc..   1
Gi1_3876772_x14..gguucucacccaacagCuccg...1
Gi3_27000123_x14...gguucgauucccggcgccgAa....................   1
Gi5_8359835_x14...................uccggcguaguauaacAgcuagaau..1
Gi5_8373351_x13...gguucgauucccggcgccggG....................1
Gi3_27060023_x13...................uccggcguaguauaacggcuagaC...   1
Gi3_27077807_x13...Auucucacccaacagauccg...   1
Gi3_27075623_x13.................Gguccggcguaguauaacggcuagaau..   1
Gi3_27038209_x13...................uccggcguaguauaacggcuaAaau..   1
Gi3_27067238_x13...................uccggcguaguauaacgUcuaga..   1
Gi1_3885592_x13..guucgauucccggcgGcgg.....................1
Gi3_27056617_x13...................uccggcguaguauaacggAua..   1
Gi3_27067030_x13...gguuAgauucccggcgccgga....................   1
Gi3_27075558_x13...................uccggcguCguauaacggc..   1
Gi1_3882914_x13...guucucGcccaacagauccg...1
Gi3_27048310_x13...gguucgauuccUggcgccg......................   1

```
Gi3_27063572_x13................................................gguucucGcccaacagauccg........................................       1
Gi3_27050338_x13......................................................................guucgauucccggcgccggagA.................       1
Gi3_27045437_x13......................................................................guucgauucccggcgccAgagc.................       1
Gi1_3888413_x13...............................................ugUuucucacccaacagauccgg.......................................1
Gi5_8373884_x13.....................................................................gguucgauucccggcgccAgagc.................1
G1a_5572419_x13.....................................................................gUuucgauucccggcgccgg....................1
Gi5_8380748_x13..................................................uucucacccaacagGuccg........................................1
Gi2_10338106_x13.....................................................................gCuucgauucccggcgccggag..................       1
Gi3_27046542_x13.....................................................................gguuGgauucccggcgccggag..................       1
Gi1_3889024_x13...............................................ugguucucacccGacagauccgg.......................................1
Gi2_10338912_x13......................................................................guucgaGucccggcgccggagc.................       1
Gi2_10324599_x13...................uccggcguagCauaacggcua.....................................................................       1
Gi2_10330592_x13......................................................................guucgauucccggcgccggaAc.................       1
Gi5_8389601_x13.....................................................................gguucgaCucccggcgccg.....................1
Gi5_8385987_x13...................uccggcguaguauaacggcuagaaC.................................................................1
Gi3_27057319_x13......................................................................guucgCuucccggcgccggagc.................       1
Gi5_8373858_x13.....................................................................Cguucgauucccggcgccgg....................1
Gi2_10322298_x13.....................................................................gguucgauucccggcAccgga...................       1
Gi5_8380410_x13......................................................................guucgauucccggcgGcgg....................1
Gi1_3891559_x13......................Ugcguaguauaacggcuag....................................................................1
Gi3_27064742_x13....................................................Gucacccaacagauccgg.......................................       1
Gi3_27059763_x13...................uccggcguaguauaacggU.......................................................................       1
Gi2_10322402_x13......................................................................guucgauucccggcgccggagcU................       1
Gi1_3885540_x13......................................................................guuNgauucccggcgccgg....................1
Gi3_27063494_x13........................................................................uGgauucccggcgccggag..................       1
Gi2_10324222_x13.....................................................................gguucgauucccggcgccAgag..................       1
Gi3_27062961_x13......................................................................guucgauCcccggcgccggag..................       1
Gi3_27065496_x13................................................gUuucucacccaacagauccgg.......................................       1
Gi5_8384674_x13.....................................................................gguucgauucccggcgAcggagc.................1
Gi3_27044670_x13......................................................................guucgauuUccggcgccggagc.................       1
Gi5_8381086_x13.................................................guucCcacccaacagauccg........................................1
Gi3_27069760_x13...................uccggcguaguauaCcggc.......................................................................       1
Gi3_27043162_x13.....................................................................gguucgauucccggcgcGggagc.................       1
Gi3_27062818_x13.....................................................................gguucgauuccUggcgcc......................       1
Gi1_3892781_x13................................................gguucucaNccaacagauccg........................................1
Gi1_3892261_x13......................................................................guucgauucccggcgccggU...................1
Gi5_8382412_x13................................................gguucucaccUaacagauccg........................................1
Gi1_3891182_x13.................................................guucucacccGacagauccgg.......................................1
Gi4_514174_x13.....................................................................Uguucgauucccggcgccggagc................. 1
Gi3_27059126_x13................................................gCuucucacccaacagauccg........................................       1
Gi3_27077092_x13.....................................................................gguucgauucccggcgccggaUc.................       1
Gi3_27042083_x13.....................................................................Cguucgauucccggcgcc......................       1
Gi3_27059906_x13.....................................................................gguucUauucccggcgccggagc.................       1
Gi3_27065327_x13......................................................................guucgGuucccggcgccg.....................       1
Gi2_10328044_x13...................uccggcguaguauaacAgcuagaau.................................................................       1
Gi5_8373910_x13.....................................................................ggGucgauucccggcgccg.....................1
Gi2_10322532_x13.....................................................................gguucgauucccggcgccggagcgA...............       1
```

```
Gi5_8390368_x13.....................................................................guucgauucccgUcgccgg.....................1
Gi2_10336663_x13.................................................Cucucacccaacagauccg........................................       1
Gi2_10342500_x13...................uccggcguaguGuaacggcuagaau................................................................       1
Gi3_27084086_x13...............................................gguucucacccaacagaucUg........................................       1
Gi1_3885072_x13...................uccggcguaguauaacggcuagU...................................................................1
Gi3_27063065_x13....................................................................gguucgauucccggcgccgA....................       1
G1a_5576280_x13...................uccggcguaguauaacggcu......................................................................0
Gi5_8383855_x13.....................................................................guucgauucUcggcgccgg.....................1
Gi5_8376679_x13.....................................................................guucgauuNccggcgccgga....................1
Gi1_3886073_x13...................Gccggcguaguauaacggcu......................................................................1
Gi3_27079484_x13...............................................ggGucucacccaacagauccg........................................       1
Gi5_8373143_x13...................uccggcguaguauaacggcuagGau.................................................................1
Gi5_8382022_x13.....................................................................guucgauucccggcgccggCgc..................1
Gi1_3891845_x13....................................................................gguucgauNcccggcgccggagc..................1
Gi2_10321648_x13.....................................................................guucgauucccggcgccggagU.................       1
Gi3_27043669_x13.....................................................................guucgauuccAggcgccgga...................       1
Gi5_8385636_x13.....................................................................guAcgauucccggcgccgg.....................1
Gi2_10333738_x13...................uccggcguaguGuaacggcua....................................................................       1
Gi2_10332256_x13....................................................................gguAcgauucccggcgccggagc.................       1
Gi1_3887464_x13...............................................ugguucCcacccaacagauccg........................................1
Gi2_10328811_x13....................................................................ggCucgauucccggcgccg.....................       1
Gi3_27072802_x13....................................................................gguucgauucccCgcgccggagc.................       1
Gi3_27052301_x13....................................................................gguucgauucccggcgcAggag..................       1
Gi5_8376926_x13....................................................................gguucgauuUccggcgccg......................1
Gi2_10332919_x13.............................................................aUauccggguucgauucc.............................       1
Gi1_3884643_x13.....................................................................guucgauucccAgcgccgga....................1
Gi3_27052756_x13....................................................................gguucgauucccggcgUcg.....................       1
Gi3_27051573_x13...................uccgUcguaguauaacggcuag...................................................................       1
Gi1_3885293_x13................................................guucucacccaCcagauccg.........................................1
Gi2_10336390_x13....................................................................gguucgauucccggcgccgCagc.................       1
Gi3_27039015_x13....................................................................gguucgauuUccggcgccgg....................       1
Gi3_27058840_x13...................uccggcguaguauaacggcCag...................................................................       1
Gi4_513719_x13.....................................................................guucgauucccggcgccg...................... 0
Gi3_27047153_x13...................uccggcAuaguauaacggcua....................................................................       1
Gi2_10345018_x12.....................................................................guucgauuNccggcgccggag..................       1
Gi1_3901406_x12...................uccggcguaguauaacggU.......................................................................1
Gi5_8400258_x12...................uccggcguaguauaacgAc.......................................................................1
Gi3_27117369_x12....................................................................gguucgauucccggcgcAg.....................       1
Gi1_3895478_x12...................uccggcguaguauaacggcuagaaA.................................................................1
Gi5_8401206_x12...................uccggcguaguaCaacggcua.....................................................................1
Gi1_3902114_x12...................uccggcguaguauaGcggcua.....................................................................1
Gi5_8392722_x12................................................guucucacccaacagaCccg.........................................1
Gi5_8396058_x12......................ggcguaguauaacggcua.....................................................................0
Gi5_8393478_x12....................................................................gguucgCuucccggcgccggagc..................1
Gi5_8392002_x12...................uccggUguaguauaacgg........................................................................1
Gi3_27110877_x12.....................................................................guucgauucccggcgccAgag..................       1
Gi3_27126693_x12.....................................................................guucgauucccCgcgccggagc.................       1
```

```
Gi5_8402034_x12......................................................................guucgauucccggcgcAgg.....................1
Gi5_8406570_x12...................uccggcguaguGuaacggcuagaau..................................................................1
Gi1_3897482_x12......................................................................guucgaGucccggcgccgg.....................1
Gi5_8400198_x12...................uccggcgCaguauaacggcuag.....................................................................1
Gi3_27097293_x12................................................gguucucacccaacagaCccg.........................................      1
Gi3_27124785_x12.....................................................................gguucgauucccggUgccgg.....................      1
Gi3_27094065_x12...................uccggcguaguCuaacggc........................................................................      1
Gi5_8400042_x12.....................................................................gAuucgauucccggcgccgga....................1
Gi1_3896822_x12...................uccggcguaguGuaacgg.........................................................................1
Gi1_3893738_x12.................................................guucucacccaacagauccAg........................................1
Gi2_10364326_x12.....................................................................gguucgauucccggGgccggagc..................      1
Gi3_27097029_x12.....................................................................gguucgauucccggcAcc.......................      1
Gi5_8404566_x12.....................................................................gguucgaCucccggcgccgg.....................1
Gi1_3898814_x12...................uccggcguaguauaaUggcuaga....................................................................1
Gi2_10354426_x12.....................................................................gguucgGuucccggcgccgga....................      1
Gi2_10359730_x12..................................................uucucacccaacagGuccg.........................................      1
Gi4_515028_x12...................uccggcguaguauaacggcuagaa................................................................... 0
Gi5_8401710_x12...................................................uGucacccaacagauccg.........................................1
Gi5_8394810_x12......................................................................guucgauucccgAcgccgg.....................1
Gi3_27103185_x12...................uccggcguaguauaacAgcuagaa...................................................................      1
Gi1_3900650_x12......................................................................guucgauucccCgcgccgg.....................1
Gi5_8407494_x12......................................................................guucgauucccggcgUcggagc..................1
Gi5_8404746_x12...................uccggUguaguauaacggcuaga....................................................................1
Gi1_3895370_x12.....................................................................gguucgauucccAgcgccgg.....................1
Gi5_8393466_x12................................................gguucucacccaacagaucAg.........................................1
Gi2_10356154_x12.....................................................................ggGucgauucccggcgccgga....................      1
Gi5_8406114_x12..............................................Gugguucucacccaacagauccg.........................................1
Gi2_10367230_x12......................................................................guucgauucccAgcgccgg.....................      1
Gi3_27131349_x12......................................................................guucgauuUccggcgccgga....................      1
Gi1_3898454_x12.................................................guucucacccaacGgauccgg........................................1
Gi5_8394774_x12......................................................................guucgauucccggcgcAg......................1
Gi3_27113469_x12........................................................................uUgauucccggcgccggagc..................      1
Gi2_10359070_x12......................................................................Cuucgauucccggcgccggag...................      1
Gi3_27101049_x12...............................................ugguucucNcccaacagauccg.........................................      1
Gi3_27127785_x12......................................................................guucgauucccggcgccggagcU.................      1
Gi2_10353190_x12.....................................................................gguucgauuccUggcgcc.......................      1
Gi3_27102801_x12...................uccggcguUguauaacggcua......................................................................      1
Gi1_3899774_x12.......................................................................uGcgauucccggcgccgg.....................1
Gi3_27108045_x12..................................................uucucacccaGcagauccgg........................................      1
Gi1_3897170_x12..................................................uucCcacccaacagauccgg........................................1
Gi3_27109329_x12...................uccggcguaguauaaAggcua......................................................................      1
Gi3_27088305_x12.....................................................................gguucgauucccggcgcUgg.....................      1
Gi1_3902258_x12................................................gguucucGcccaacagauccg.........................................1
Gi3_27103977_x12..............................................Gugguucucacccaacagaucc..........................................      1
Gi2_10354906_x12......................................................................guucgauucccAgcgccg......................      1
Gi3_27112941_x12.....................................................................gguucgaAucccggcgccggagc..................      1
Gi2_10350382_x12......................................................................Auucgauucccggcgccg......................      1
```

```
Gi3_27126453_x12................................................gUuucucacccaacagauccgggu.....................................       1
Gi5_8404698_x12..................................................uucCcacccaacagauccg........................................1
Gi1_3897854_x12......................................................................guucgauucccUgcgccgg....................1
Gi1_3895874_x12..................................................uucucacccaacaAauccgg.......................................1
Gi5_8407074_x12.....................................................................gguucgauucccggcgcAggagc.................1
Gi5_8401998_x12.................................................guucucaccUaacagauccg........................................1
Gi2_10361794_x12................................................Aguucucacccaacagauccg........................................       1
Gi2_10347886_x12...................uGcggcguaguauaacggcuagaa..................................................................       1
Gi3_27122361_x12.....................................................................gguucgauuUccggcgccg.....................       1
Gi1_3899882_x12....................ccggcguGguauaacggcuag....................................................................1
Gi3_27092649_x12...................uccggcAuaguauaacgg........................................................................       1
Gi2_10349386_x12......................................................................gAucgauucccggcgccggagc.................       1
Gi3_27117285_x12.....................................................................gguucgauucAcggcgccggag..................       1
Gi5_8406918_x12...................uccggcguaguauaacggcuG.....................................................................1
Gi5_8408574_x12...................uUcggcguaguauaacggc.......................................................................1
Gi1_3894194_x12...............................................uCguucucacccaacagauccgg.......................................1
Gi2_10365874_x12.....................................................................gguNcgauucccggcgccggagc.................       1
Gi3_27104649_x12..................................................uucCcacccaacagauccgg.......................................       1
Gi2_10344010_x12......................................................................guuUgauucccggcgccg.....................       1
Gi3_27105573_x12...................uccggcguaguauaacggcuagCa..................................................................       1
Gi3_27117957_x12.................................................guucucacccaacCgauccg........................................       1
Gi5_8395722_x12.....................................................................gguucgauucccggcgcUgga...................1
Gi2_10348270_x12..................................................uCcucacccaacagauccg........................................       1
Gi3_27106473_x12...................Accggcguaguauaacggc.......................................................................       1
Gi1_3900710_x12...................uccggcguaguauaacAgcuaga...................................................................1
Gi2_10355794_x12...................uccggcguaguauaacggcuagGa..................................................................       1
Gi3_27096909_x12......................................................................guucgauucccggcgGcgg....................       1
Gi3_27093009_x12.....................................................................gguucgauucccggcgccggU...................       1
Gi5_8403234_x12.....................................................................gguucgauucccggcgUcggagc.................1
Gi5_8392854_x12...................uccggcguaguauCacggcua.....................................................................1
Gi5_8400306_x12......................................................................guucgauucccggcgAcg.....................1
Gi2_10363918_x12.....................................................................gguucgCuucccggcgccggag..................       1
Gi5_8399538_x12...................Accggcguaguauaacggcuag....................................................................1
Gi3_27101517_x12................................................gguucucacccaacagGuccg........................................       1
Gi5_8401914_x12......................................................................Uuucgauucccggcgccggag..................1
Gi5_8404434_x12......................................................................gCucgauucccggcgccggagc.................1
Gi1_3896510_x12...................uccggUguaguauaacggcua.....................................................................1
Gi2_10344394_x12......................................................................guucgauucccggcAccgga...................       1
Gi5_8401218_x12.....................................................................gUuucgauucccggcgccggagcgu...............1
Gi3_27087333_x12..........................................................caacagauccggguucgauucccggcgccgg....................       0
Gi3_27113829_x12...................Accggcguaguauaacggcua.....................................................................       1
Gi5_8404902_x12.....................................................................gguucgauucccggcgccggCg..................1
Gi2_10356202_x12..........................................................................gGuucccggcgccggagc.................       1
Gi3_27123429_x12......................................................................guucgauucccggcgccggagU.................       1
Gi3_27118293_x12......................................................................guucUauucccggcgccggag..................       1
Gi2_10377172_x11...................uccggcguaguauCacggc.......................................................................       1
Gi3_27144360_x11..............................................................aUauccggguucgauucc.............................       1
```

```
Gi2_10391230_x11.....................cUgcguaguauaacggcua......................................................................       1
Gi3_27177382_x11.......................................................................guucgauuNccggcgccg.....................       1
Gi3_27173730_x11.......................................................................guucgauucccggcgccgU....................       1
Gi1_3909963_x11.......................................................................guucgauucccggcgccAga...................1
Gi5_8428219_x11..................................................guucucacUcaacagauccg........................................1
Gi2_10376281_x11.......................................................................guucgauucccggcgccggCg..................       1
Gi5_8419232_x11.....................................................cucacccaacagGuccgg.......................................1
Gi2_10384421_x11...................uUcggcguaguauaacgg.........................................................................       1
Gi3_27166690_x11.........................................................................Gcgauucccggcgccgga...................       1
Gi5_8413666_x11...................uccgAcguaguauaacgg.........................................................................1
Gi2_10376468_x11.......................................................................guucgauuccUggcgccggag..................       1
Gi3_27183982_x11.......................................................................guucgauucccggAgccggagc.................       1
Gi3_27149948_x11......................gUcguaguauaacggcua......................................................................       1
Gi3_27183509_x11...................uccggcguaguauaacgAcuag.....................................................................       1
Gi2_10384201_x11.......................................................................guucgauucccggcgcAgg....................       1
Gi3_27183586_x11.......................................................................guucgauucccggcAccggag..................       1
Gi5_8410355_x11.................................................Uguucucacccaacagauccggguu....................................1
Gi5_8418066_x11......................................................................gCuucgauucccggcgccg.....................1
Gi5_8418451_x11...................uccggUguaguauaacggc........................................................................1
G1a_5609469_x11...................Gccggcguaguauaacggcuag.....................................................................1
Gi3_27173664_x11...................Accggcguaguauaacgg.........................................................................       1
Gi3_27179087_x11...................uccggcguaguauCacggcuag.....................................................................       1
Gi5_8412951_x11..................................................guucucacccaacagauccA........................................1
Gi2_10373432_x11......................................................................gguucgauuUccggcgcc......................       1
Gi3_27169869_x11..................................................guucucacccaacagGuccg........................................       1
Gi3_27137221_x11..................................................Uuucucacccaacagauccgggu.....................................       1
Gi3_27169121_x11......................................................................gguucgauucccAgcgccgg....................       1
Gi5_8423874_x11.......................................................................guucgauucccgAcgccgga...................1
Gi3_27180308_x11.......................................................................guuAgauucccggcgccgg....................       1
Gi5_8422455_x11.......................................................................gAucgauucccggcgccg.....................1
Gi5_8414458_x11......................................................................gguucgauuUccggcgccggagc.................1
Gi3_27167427_x11.......................................................................guucgauucccgUcgccgga...................       1
Gi1_3909710_x11......................................................................Aguucgauucccggcgccggagc.................1
Gi5_8429264_x11.......................................................................guucgauuUccggcgccg.....................1
Gi5_8410553_x11...................Accggcguaguauaacggcuaga....................................................................1
Gi5_8413479_x11......................gUcguaguauaacggcuag.....................................................................1
Gi5_8418671_x11...................uccggcguaguauaacggcuagaauggu...............................................................0
Gi1_3905453_x11......................................................................Aguucgauucccggcgccgga...................1
Gi3_27145086_x11...................uccggcguaguauaacggcuagCau..................................................................       1
Gi1_3909952_x11......................................................................gguucgauuccUggcgccgg....................1
Gi5_8431057_x11.......................................................................guucgauucAcggcgccg.....................1
Gi2_10371342_x11.......................................................................guucgauucccggUgccgga...................       1
Gi3_27157802_x11................................................ugguucucacccaacGgauccg........................................       1
Gi3_27186776_x11........................................................................uuUgauucccggcgccggagc.................       1
Gi3_27145449_x11.................................................gguucucacccaacagauccA........................................       1
Gi3_27178009_x11.....................................................................Ugguucgauucccggcgccgga...................       1
Gi2_10392935_x11......................................................................gguucgaAucccggcgccggagc.................       1
```

```
Gi2_10393540_x11.....................................................................gguucgauucccggcgUc......................        1
Gi1_3907873_x11......................................................................guucgauucccggcgcUgga....................1
Gi3_27144250_x11...................uccggcguaguauCacggcuagaau.................................................................        1
Gi5_8415030_x11.................................................guucucacccaacGgauccg.........................................1
Gi3_27164699_x11.....................................................................gguucgauAcccggcgccgga...................        1
Gi3_27151818_x11...................uccggcguaguauaacggcuagGa..................................................................        1
Gi3_27147528_x11...................uccggGguaguauaacggcuaga...................................................................        1
Gi2_10391307_x11......................................................................Cuucgauucccggcgccg.....................        1
Gi5_8428120_x11.....................................................Gcacccaacagauccggguu.....................................1
Gi3_27168615_x11........................................................................ucgauuccNggcgccggagc.................        1
G1a_5613319_x11......................................................................Uuucgauucccggcgccgg.....................1
Gi5_8420310_x11......................................................................guucgauucccggcgccgA.....................1
Gi5_8418330_x11.................................................guGcucacccaacagauccg.........................................1
Gi2_10373322_x11......................................................................guGcgauucccggcgccg.....................        1
Gi5_8418286_x11.....................................................................gguucgauuccUggcgccg......................1
Gi1_3905156_x11.....................................................................gguucgauucccggcgcAgg.....................1
Gi3_27167878_x11......................................................................guucgCuucccggcgccgg....................        1
Gi2_10397588_x11.....................................................................gguucgauCcccggcgcc......................        1
Gi3_27143898_x11...................uccggcguaguaAaacggcuaga...................................................................        1
Gi2_10385290_x11........................................................................gCucgauucccggcgccggag................        1
Gi2_10384608_x11...................uccgUcguaguauaacggcua.....................................................................        1
Gi2_10388832_x11.....................................................................ggAucgauucccggcgcc......................        1
Gi3_27142325_x11.........................................................ccaacagauccggguucgauucccggcgccgg....................        0
Gi5_8424138_x11...................................................Gcucacccaacagauccgg........................................1
Gi5_8420024_x11.....................................................................gguucgauucccggcgccA......................1
Gi2_10381913_x11......................................................................Auucgauucccggcgccggag..................        1
Gi3_27151873_x11......................................................................guucgauucccggcgUcggagc.................        1
Gi1_3914561_x11...................................................Gcucacccaacagauccgg........................................1
Gi3_27165073_x11...................uccggcguaguauaacggUuag....................................................................        1
Gi2_10396785_x11...................uccggcguaguauaacggcuGg....................................................................        1
Gi2_10392671_x11................................................ggCucucacccaacagauccg........................................        1
Gi3_27156955_x11....................................................cucacccaacagauccggguucgauuccc............................        0
Gi3_27184686_x11...................uccggAguaguauaacgg........................................................................        1
Gi3_27178471_x11................................................gguucucacccaacagaucAg........................................        1
Gi1_3910568_x11...............................................ugguucucacccaacagGuccgg........................................1
Gi3_27162972_x11.................................................................Gccggguucgauucccggcgccgga...................        1
Gi1_3908324_x11......................................................................guucgauucccggcgccggagcU.................1
Gi3_27156548_x11...................uccggcguaguauaaUggcuag....................................................................        1
Gi3_27150520_x11...................uccggcguaguauGacggc.......................................................................        1
Gi2_10387710_x11...................uccggcguaguauaacAgcuag....................................................................        1
Gi2_10379944_x11.....................................................................ggGucgauucccggcgcc......................        1
Gi5_8426822_x11.....................................................................gguucgauucccggcgcUg......................1
Gi1_3915683_x11........................cguaguauaacggcuagaau..................................................................0
Gi3_27138805_x11.....................................................................ggGucgauucccggcgcc......................        1
Gi5_8427636_x11...................uccggcguaguGuaacggc........................................................................1
Gi5_8419903_x11......................................................................guuUgauucccggcgccgga....................1
Gi2_10378382_x11...................uccgAcguaguauaacggcuaga...................................................................        1
```

Gi5_8420563_x11...................uccggcguaguaCaacgg..1
Gi2_10369868_x11..ucgauuccNggcgccggagc................   1
Gi3_27133789_x11...................uccggcguaguaGaacggcua..   1
Gi3_27157648_x11...uGcgauucccggcgccgg...................   1
Gi2_10395916_x11..guucgauucccggcgccggagcgA..............   1
Gi3_27179593_x11..guucgauuAccggcgccgga..................   1
Gi2_10377293_x11...................uccggcguaguauaGcggc..   1
Gi5_8429451_x11...................uUcggcguaguauaacggcuagaa..1
Gi5_8426338_x11...................uccggcguaguauaacAg..1
Gi2_10397500_x11...................uccggcguCguauaacggcuaga..   1
Gi3_27178779_x11...gguucgauuAccggcgccggag.................   1
Gi5_8425271_x11..gguucucaUccaacagauccg..1
Gi2_10394222_x11...gguucgaCucccggcgccgga..................   1
Gi1_3910612_x11...................Cccggcguaguauaacggcua...1
Gi3_27148716_x11...................uccggUguaguauaacggcuagaa...   1
Gi1_3908104_x11..uucucacccaGcagauccg..1
Gi1_3910887_x11..uAcucacccaacagauccg..1
Gi1_3915661_x11...................uccggcguagCauaacgg..1
Gi2_10389547_x11....................ccggcguaguauaacggcuagaau..   0
Gi2_10383827_x11...................uccggcguaguauaGcgg...   1
Gi2_10395388_x11..uuUucacccaacagauccg.......................................   1
Gi3_27172443_x11..guucgauucccggcgccgCagc................   1
Gi5_8410993_x11...................uccggcguaguGuaacggcua...1
Gi3_27167482_x11...gguucgauucccAgcgccg....................   1
Gi3_27180517_x11...gCucucacccaacagauccg.......................................   1
Gi1_3907752_x11..guucAauucccggcgccgga...................1
Gi3_27233622_x10..gCguucgauucccggcgccggagc................   1
Gi5_8442986_x10...................uccgAcguaguauaacggcuagaau...1
Gi5_8434526_x10..uGcucacccaacagauccgg.......................................1
Gi5_8449856_x10...gguucgGuucccggcgccg.....................1
Gi3_27190342_x10...................uccggcguCguauaacggcua..   1
Gi3_27225642_x10...................uccAgcguaguauaacggcuag...   1
Gi3_27204172_x10...gguucAauucccggcgccg....................   1
Gi1_3925234_x10...gCucucacccaacagauccgg.......................................1
Gi3_27209772_x10...gguucgauucccggcAccg....................   1
Gi3_27189252_x10...................uccggcguaguGuaacggcuagaa...   1
Gi3_27205472_x10..guuUgauucccggcgccggag.................   1
Gi5_8447786_x10...gguucgauucccgAcgccggagc.................1
Gi2_10412292_x10...gguucgUuucccggcgccggag.................   1
Gi2_10401452_x10..guucgauucccggcAccg....................   1
Gi1_3924494_x10...ugguCcucacccaacagauccg..1
Gi3_27220922_x10...gguucgauucccUgcgccg....................   1
Gi5_8447776_x10...................ucUggcguaguauaacggcuagaa..1
Gi2_10415192_x10...guucucacNcaacagauccg.......................................   1
Gi2_10404252_x10...gguucgauuUccggcgccgga..................   1
Gi2_10412482_x10...................uccggcguaguCuaacggcuaga..   1
Gi2_10407172_x10..guucgauucccgAcgccggagc................   1

Gi1_3923774_x10...gguucgauuUccggcgccgg....................1
Gi1_3920124_x10...ugguucucacUcaacagauccgg.......................................1
Gi1_3921274_x10..guCcgauucccggcgccg.....................1
Gi1_3920724_x10...aacagauccggguucgauucccggcgccggagc................. 0
Gi2_10405312_x10..guucgauucccggcgcAggagc.................   1
Gi2_10415872_x10...................uUcggcguaguauaacggcua...   1
Gi1_3926524_x10...guucucacccaCcagauccgg.......................................1
Gi2_10413142_x10..guucgauucccggcgccggGg..................   1
Gi3_27231662_x10..gguucAcacccaacagauccg..   1
Gi3_27222132_x10...................uccggcguaguauaaUggcuagaa..   1
Gi2_10411452_x10...................uccggcguaguauaacggcCa...   1
Gi2_10427882_x10...gguucgauucccggcgccCgagc.................   1
Gi2_10409192_x10...gguuUgauucccggcgcc......................   1
Gi2_10397762_x10..guucgauuUccggcgccggagc.................   1
Gi3_27188212_x10..guucgCuucccggcgccgga...................   1
Gi5_8439926_x10...Cgguucucacccaacagauccg..1
Gi3_27239032_x10...................uccggcgCaguauaacggcuagaau...   1
Gi2_10417612_x10...................uccggcguaguauaGcggcua...   1
Gi3_27217412_x10...gguucgauuccAggcgccggag..................   1
Gi2_10409622_x10...gguucgauucccggcgccggC...................   1
Gi3_27192892_x10..guucgauucccgUcgccgg....................   1
Gi2_10410772_x10...gguucgauucccggcgUcgga...................   1
Gi2_10414892_x10..ggGucucacccaacagauccg..   1
Gi2_10399502_x10...guucucacccaacagGuccg..   1
Gi3_27205052_x10...................uUcggcguaguauaacggcuagaa..   1
Gi2_10412052_x10...................uccggcguaguauaacggcuagaaC...   1
Gi3_27209272_x10..guucgauucccggcgccggUgc.................   1
Gi5_8439896_x10...................uccggcAuaguauaacggcuagaau...1
Gi2_10419612_x10...cUauucccggcgccggagcguuu.............   1
Gi5_8448436_x10..guucgauucccggcgccggGgc.................1
Gi1_3927234_x10...................uccggcguGguauaacggcuaga...1
Gi5_8447646_x10..guucgauucAcggcgccgg....................1
Gi3_27204082_x10..guucgauucccggcgcUggagc.................   1
Gi5_8449706_x10...ucucacccNacagauccg..1
Gi3_27210902_x10..guucgauAcccggcgccggagc.................   1
Gi2_10407042_x10...gguucgauuccUggcgccggag..................   1
Gi1_3927194_x10..gguCcucacccaacagauccg..1
Gi3_27221662_x10...gGucucacccaacagauccgggu.....................................   1
Gi2_10403602_x10..gguucCcacccaacagauccg..   1
Gi5_8448506_x10...................uccggcguaguauGacggcuagaau...1
Gi4_520909_x10..gGucgauucccggcgccgg.................... 1
Gi5_8452316_x10..guucgauuNccggcgccggagc.................1
Gi2_10403512_x10...gguucgauucccggcgccgUagc.................   1
Gi3_27205302_x10...................uccggcguaguauaacggcuaCa...   1
Gi3_27226722_x10..guucgauucccggcAccggagc.................   1
Gi2_10409482_x10...................uccggcguaguauaacgAc...   1
Gi1_3923174_x10..guucgaAucccggcgccgg....................1

```
Gi1_3926114_x10...................Cccggcguaguauaacggcuag....................................................................1
Gi2_10427752_x10...................uccggAguaguauaacggcuaga...................................................................       1
Gi3_27217682_x10...............................................uggCucucacccaacagauccg........................................       1
Gi3_27206282_x10.....................................................................gguucgauucccgAcgccg.....................       1
Gi1_3924034_x10......................Ugcguaguauaacggcua.....................................................................1
Gi1_3917614_x10...................uccggcguaguauaaUggcua.....................................................................1
Gi3_27191172_x10.....................................................................gguucgCuucccggcgccgg....................       1
Gi1_3926564_x10.................................................guucucacccaGcagauccg........................................1
Gi3_27243812_x10...................uccgUcguaguauaacggcuagaa..................................................................       1
Gi3_27239182_x10........................................................................Gcgauucccggcgccggag..................       1
Gi1_3922454_x10...................uccgUcguaguauaacggcua.....................................................................1
Gi3_27235662_x10...................uccggcCuaguauaacggcuaga...................................................................       1
Gi2_10403522_x10.....................................................................gguucgCuucccggcgcc......................       1
Gi3_27197082_x10......................................................................guAcgauucccggcgccggag..................       1
Gi5_8448566_x10...................uccggcguaguauaCcggcuaga...................................................................1
Gi2_10412472_x10.....................................................................gguucAauucccggcgcc......................       1
Gi1_3926694_x10.................................................guucCcacccaacagauccg........................................1
Gi5_8452586_x10...............................................uggCucucacccaacagauccg........................................1
Gi5_8431736_x10...................uccggcguaguauaacggcuagCau.................................................................1
Gi3_27240502_x10......................................................................Cuucgauucccggcgccg.....................       1
Gi5_8444156_x10.................................................Cuucucacccaacagauccg........................................1
Gi2_10415822_x10.................................................Auucucacccaacagauccg........................................       1
Gi1_3928064_x10...................uccggcguaguauaacgAcuaga...................................................................1
Gi1_3920084_x10...............................................Cgguucucacccaacagauccgg.......................................1
Gi3_27198502_x10....................ccggcguaguauaacggcuagaau.................................................................       0
Gi3_27240962_x10................................................gguucucaccUaacagauccg........................................       1
Gi2_10412842_x10................................................gguuUucacccaacagauccg........................................       1
Gi3_27217962_x10...................uccggAguaguauaacggc.......................................................................       1
Gi3_27209962_x10...................uccggcguagGauaacggcuaga...................................................................       1
Gi3_27220262_x10......................................................................guucgauucccggcgccC.....................       1
Gi2_10403692_x10......................................................................guucgauucccgAcgccgg....................       1
Gi5_8433596_x10..................................................uucucacccaGcagauccg........................................1
Gi5_8434636_x10.....................................................................ggAucgauucccggcgccg.....................1
Gi3_27204492_x10......................................................................guucgauucccgAcgccgga...................       1
Gi1_3916754_x10.....................................................................gguucAauucccggcgccgg....................1
Gi1_3927214_x10...................uccggcguaguauCacggcuaga...................................................................1
Gi5_8451806_x10...................uccggcguaAuauaacggcuaga...................................................................1
Gi5_8436516_x10................................................gguucucacAcaacagauccggguu....................................1
Gi5_8447406_x10......................................................................guucgCuucccggcgccgg....................1
Gi1_3917474_x10...................uccggcguaguauCacggcuag....................................................................1
Gi2_10417302_x10.....................................................................gguucgauucccggcgcGggagc.................       1
Gi2_10419012_x10.....................................................................gCuucgauucccggcgcc......................       1
Gi4_520139_x10................................................gguucucacccaacagauccg........................................ 0
Gi3_27190712_x10.....................................................................gguucgauucAcggcgccgga...................       1
Gi1_3917414_x10....................ccggcguaguauaacggcuagaa..................................................................0
Gi3_27222292_x10...................uccggcguaguauaaUggcuagaau.................................................................       1
Gi2_10423752_x10...................uccggcguaguGuaacgg........................................................................       1
```

Gi1_3917484_x10..uucucacccaacagauAcgg..1
Gi2_10411892_x10..guucgGuucccggcgccg.....................   1
Gi5_8435706_x10...................uccggcguaguCuaacggcuag...1
Gi5_8452576_x10...gguucgauucccggAgccggagc..................1
Gi1_3928104_x10..cGcacccaacagauccgg..1
Gi5_8435356_x10...gguCcgauucccggcgccg......................1
Gi5_8473674_x9...................uccgAcguaguauaacggcuaga.. 1
Gi5_8454351_x9...ggGucgauucccggcgccgg..................... 1
Gi2_10454124_x9...................uccggcguaguauaacggcuagaCu..1
Gi2_10447860_x9..guucgauucAcggcgccgg.....................1
Gi3_27258040_x9...................Cccggcguaguauaacggcuag...1
Gi2_10443045_x9..guucgauucccggcgccggagA..................1
Gi3_27264709_x9...................uccggcgAaguauaacggcuaga..1
Gi3_27302113_x9...gguuAgauucccggcgccggag...................1
Gi5_8467374_x9..guucgauucccCgcgccgg..................... 1
Gi2_10437555_x9..guucgauucccggcgccCg.....................1
Gi3_27281638_x9...Ggauucccggcgccggagc..................1
Gi3_27283429_x9..guucgauucccggAgccgga....................1
Gi5_8462433_x9...................uccggcguaguauaacggcuC.. 1
Gi2_10434306_x9...gguucgauucccggcgccggaUc..................1
Gi5_8461515_x9..guucUauucccggcgccgga.................... 1
Gi5_8465457_x9...................uccggUguaguauaacggcuagaau.. 1
Gi2_10446852_x9...gguucgauucccggUgccg......................1
Gi3_27249112_x9..guGcgauucccggcgccg......................1
Gi5_8466249_x9...................uccggcgCaguauaacggc.. 1
Gi3_27297991_x9...ggAucgauucccggcgccgg.....................1
Gi5_8463774_x9..gGuucccggcgccggagc.................. 1
Gi1_3940336_x9..uucucaccUaacagauccg... 1
Gi1_3935098_x9...................uccggUguaguauaacggc.. 1
Gi5_8472180_x9...gguucgauuccUggcgccgg..................... 1
G1a_5648809_x9...acccaacagauccggguucgauuccc............................. 0
Gi2_10455438_x9...................uccggcgCaguauaacggcua..1
Gi1_3929473_x9...................uccggcguaguauCacggcua.. 1
Gi5_8454072_x9..Ggauccggguucgauuccc............................. 1
Gi1_3936016_x9..guuUgauucccggcgccgga.................... 1
Gi2_10446348_x9...................uccggcguaAuauaacggcuaga..1
Gi5_8455422_x9...Ggguucucacccaacagauccgg.. 1
Gi1_3940768_x9...................uccAgcguaguauaacgg... 1
Gi5_8460930_x9..guucgauucccggcgcUgga.................... 1
Gi5_8458752_x9...gguucgauucccggcgccAga.................... 1
Gi5_8466123_x9..guucgauucccggAgccg...................... 1
Gi2_10440687_x9.....................cggcguaguauaacggcuagaaC..1
Gi2_10447644_x9...gguucgCuucccggcgccg......................1
Gi5_8470929_x9...................Accggcguaguauaacgg... 1
Gi2_10461621_x9...................uccggcguagCauaacggc..1
Gi3_27270208_x9...................uccgAcguaguauaacgg...1
Gi3_27245107_x9...ggGucgauucccggcgccgg.....................1

```
Gi1_3934828_x9.................................................guucucaUccaacagauccg........................................ 1
Gi5_8463549_x9...................uccggcguaguauaaUggc....................................................................... 1
Gi5_8458428_x9.....................................................................gguucgauucccggcgccgC.................... 1
Gi5_8455944_x9...................uccggcguaguauaacggcuagU................................................................... 1
Gi5_8469930_x9.....................................................................gguucgauucccggcAccgga................... 1
Gi1_3932884_x9......................................................................gCucgauucccggcgccggagc................. 1
Gi3_27263017_x9......................................................................guucgauucccCgcgccgga...................1
Gi2_10432020_x9......................................................................guucAauucccggcgccgga...................1
Gi3_27296749_x9....................................................................ggguucgauucccggcgccggagcC................1
Gi2_10457940_x9...................uccggcguaguauaUcggcuaga...................................................................1
Gi2_10457346_x9.....................................................................gguucgauCcccggcgccg.....................1
Gi2_10438842_x9......................................................................guucgauucccggcgUcgga...................1
Gi5_8470668_x9.....................................................................gguucgauucccggcgccggaCc................. 1
Gi5_8455935_x9...................uccggcguaguauGacgg........................................................................ 1
Gi3_27260965_x9......................................................................guucgauucccAgcgccg.....................1
Gi2_10458642_x9.....................................................................gguucgauucccggAgccg.....................1
Gi1_3929077_x9................................................gguucucacccaacGgauccg........................................ 1
G1a_5644426_x9......................................................................guucgauucccggcgccggag.................. 0
Gi5_8467230_x9.....................................................................gguucgCuucccggcgccggag.................. 1
Gi3_27285409_x9.................................................guucucacccaCcagauccg........................................1
Gi5_8453289_x9................................................gguucucCcccaacagauccg........................................ 1
Gi2_10448607_x9......................................................................guucgauucccggcgAcg.....................1
Gi4_521253_x9 ......................................................................guucgauucccggcgccggagc................. 0
Gi3_27288757_x9......................................................................guucAauucccggcgccggag..................1
Gi2_10447014_x9.................................................gCucucacccaacagauccg........................................1
Gi5_8458914_x9................................................gguucucacccaacUgauccg........................................ 1
Gi1_3931039_x9...............................................ugguucucGcccaacagauccg........................................ 1
Gi1_3937897_x9...................uccggcguaguauGacgg........................................................................ 1
Gi1_3934774_x9...................uccggcguaguauCacgg........................................................................ 1
Gi5_8453658_x9...................uccggcguaguauaacggcuaA.................................................................... 1
Gi1_3933055_x9...................uccggcgCaguauaacggcuag.................................................................... 1
Gi5_8473503_x9...................uccggcguaguCuaacggcua..................................................................... 1
Gi2_10451217_x9...................uccggcguaguauaacAg........................................................................1
Gi3_27257527_x9...................uccggcguaguauaacgC........................................................................1
Gi3_27248194_x9......................Ugcguaguauaacggcuag....................................................................1
Gi5_8476581_x9......................................................................guucgauucccAgcgccgga................... 1
Gi3_27247375_x9.....................................................................gguucgauucccggcCccggagc.................1
Gi5_8459643_x9.....................................................................gguucgGuucccggcgccgg.................... 1
Gi1_3936475_x9.....................................................................gguucgauNcccggcgccg..................... 1
Gi1_3932569_x9...................uccggcguagCauaacggcuag.................................................................... 1
Gi5_8471865_x9...................uccggcguagCauaacggcuagaau................................................................. 1
Gi5_8465142_x9.................................................guucucacccaacagaucUg........................................ 1
Gi2_10434270_x9.....................................................................gguucgauucccCgcgccggagc.................1
Gi3_27245278_x9..................................................uucucUcccaacagauccg........................................1
Gi5_8459157_x9................................................gguucAcacccaacagauccg........................................ 1
G1a_5644372_x9...................uGcggcguaguauaacggcuaga................................................................... 1
Gi1_3939337_x9...................uccggcguGguauaacggc....................................................................... 1
```

```
Gi5_8464548_x9.................................................guucucacccCacagauccgg....................................... 1
Gi1_3931120_x9........................................................................ucgauucccggcgccggagcC................ 1
Gi3_27271405_x9......................................................................guucgauucccCgcgccgg....................1
Gi1_3932146_x9.....................................................................gguucgauucccggcgccggagcgA............... 1
Gi1_3931111_x9..................................................uucucacccaacagaucUg........................................ 1
G1a_5650780_x9...................uGcggcguaguauaacggcuag.................................................................... 1
Gi2_10439625_x9...................ucUggcguaguauaacgg........................................................................1
Gi5_8468166_x9.................................................guucucacccaacagaucAg........................................ 1
Gi3_27290737_x9.....................................................................gguucgauuccGggcgccgga...................1
Gi1_3933244_x9..................................................uucucacccaacagaucUgg....................................... 1
Gi5_8464125_x9.......................................................................Gucgauucccggcgccgg.................... 1
Gi5_8473449_x9......................Ugcguaguauaacggcuaga................................................................... 1
Gi3_27268903_x9.....................................................................gguGcgauucccggcgccggagc.................1
Gi2_10450506_x9.....................................................................Cguucgauucccggcgccgga...................1
Gi1_3933739_x9.................................................guucucacccaacCgauccgg....................................... 1
Gi5_8453325_x9....................................................cucaccUaacagauccgg....................................... 1
Gi2_10443882_x9.....................................................................gguucAauucccggcgccggag..................1
Gi5_8461047_x9.....................................................................Uguucgauucccggcgccggagcgu............... 1
Gi5_8466564_x9...................uccggcguaguGuaacggcuagaa.................................................................. 1
Gi1_3941641_x9..................................................uucucacUcaacagauccg........................................ 1
Gi3_27303679_x9..................................................uucucacccaCcagauccgg.......................................1
Gi3_27256195_x9.....................................................................gguucgauucccggcgccgUagc.................1
Gi5_8453280_x9...............................................ugguucucNcccaacagauccg........................................ 1
Gi3_27257707_x9................................................gguucucacccaaUagauccg........................................1
Gi5_8469489_x9......................................................................guucgauAcccggcgccg..................... 1
Gi3_27306514_x9.................................................guucucacccaGcagauccg........................................1
Gi5_8461218_x9.....................................................................ggGucgauucccggcgcc...................... 1
Gi3_27273718_x9..................................................uucucacccGacagauccg........................................1
Gi5_8463207_x9...............................................Ggguucucacccaacagauccgggu..................................... 1
Gi5_8465079_x9...................uccggcguaguauaCcggc....................................................................... 1
Gi5_8498585_x8................................................gguucucacAcaacagauccg........................................ 1
Gi5_8492497_x8...................................................ucCcacccaacagauccg........................................ 1
Gi5_8501385_x8.....................................................................gguucgauuccAggcgccggagc................. 1
Gi1_3942421_x8.....................................................................gguucgauucUcggcgccgg.................... 1
Gi5_8479753_x8.................................................guucucacccaacUgauccg........................................ 1
Gi2_10474298_x8.....................................................................gguucgauucccggcgccggagG.................1
Gi5_8488553_x8...................uccggcguaguauGacggcuaga................................................................... 1
Gi2_10492594_x8......................................................................guucgauCcccggcgccgga...................1
Gi5_8488249_x8...................uccggcguagCauaacggcua..................................................................... 1
Gi2_10489418_x8...................uccggcguaguauaGcggcuagaau.................................................................1
Gi1_3943805_x8......................................................................guucgUuucccggcgccgg.................... 1
Gi1_3947309_x8......................................................................guucgauucccggcgccggGgc................. 1
Gi3_27324385_x8.......................................................................Gucgauucccggcgccgg....................1
Gi3_27317361_x8...................uccggcguaguaCaacggcuagaa..................................................................1
Gi2_10466818_x8......................................................................Cuucgauucccggcgccgga...................1
Gi3_27317417_x8...................uccggcguaguCuaacggcuag....................................................................1
G1a_5671057_x8.....................................................................Aguucgauucccggcgccggagc................. 1
```

```
Gi2_10483818_x8...................uccggcguGguauaacggcuagaau..................................................................1
Gi2_10487410_x8......................................................................gguucgauucUcggcgcc......................1
Gi2_10493754_x8.......................................................................guucgauuAccggcgccggagc.................1
Gi5_8479161_x8.....................................................cucGcccaacagauccgg....................................... 1
Gi5_8488329_x8...................uccggcguaguauaaUggcua...................................................................... 1
Gi5_8476985_x8.................................................gguucucacccaacagauAcg........................................ 1
Gi2_10480930_x8......................................................................gguucgCuucccggcgccgga...................1
Gi5_8490161_x8......................................................................ggGucgauucccggcgccggag.................. 1
Gi1_3952669_x8...................uccggcguNguauaacggcuaga.................................................................... 1
Gi2_10466082_x8......................................................................gguucgauucccggcgUcg.....................1
Gi2_10482514_x8...................uccggcguaguauaacggcuC.......................................................................1
Gi2_10467834_x8.......................................................................guucgauucccggcCccggagc.................1
Gi5_8500185_x8...................Cccggcguaguauaacggcuagaa................................................................... 1
Gi5_8486489_x8.......................................................................gGucgauucccggcgccggag.................. 1
Gi3_27348209_x8......................................................................gguuUgauucccggcgcc......................1
Gi5_8494609_x8......................................................................gguucgauucccggcgUc...................... 1
Gi5_8487249_x8.......................................................................guucgauucccggcgccggG................... 1
G1a_5654737_x8..................................................gGucucacccaacagauccg........................................ 1
Gi1_3947997_x8...................uccggcguaguGuaacggcua...................................................................... 1
Gi1_3954701_x8...................uccggcguaguCuaacgg......................................................................... 1
Gi3_27353129_x8...................uccggcguaguauaacggcCagaa...................................................................1
Gi3_27354289_x8..................................................guucucacccaacGgauccg........................................1
Gi5_8479537_x8......................................................................gguucgauucccggAgccgga................... 1
Gi1_3942557_x8...................uccggcguaguauaacggcCa...................................................................... 1
Gi2_10479218_x8......................................................................gguucgauucccggcgAcgga...................1
Gi2_10497954_x8...................uccggcguaguauGacggcuaga....................................................................1
Gi1_3943245_x8...................ucUggcguaguauaacggc........................................................................ 1
Gi3_27310729_x8......................................................................gguucgauucccggcgccgCag..................1
Gi2_10480546_x8......................................................................gguucgauucccggUgccgga...................1
Gi2_10474722_x8...................uccggcguUguauaacggc........................................................................1
Gi1_3952253_x8...................Cccggcguaguauaacggcuaga.................................................................... 1
Gi3_27361201_x8...................................................uucucacccaacagauAcg........................................1
Gi5_8477785_x8.......................................................................guCcgauucccggcgccgga................... 1
Gi1_3952509_x8...................uccggcguaguauaacggUuaga.................................................................... 1
Gi3_27358193_x8...................uccggcgCaguauaacggcuagaa...................................................................1
Gi2_10482586_x8.......................................................................guucgauucccggcgccUgagc.................1
Gi3_27331577_x8......................................................................gguucgCuucccggcgcc......................1
Gi1_3957061_x8................................................ugguucucacccaacagauAcgg....................................... 1
Gi1_3943317_x8...................uccggcguaguauaacggcuaA..................................................................... 1
Gi2_10498522_x8...................uccggcguNguauaacggcuagaa...................................................................1
Gi3_27350665_x8...................uccggcguUguauaacggcuagaau..................................................................1
Gi2_10494290_x8...................uccggcguaguauaacAgcua......................................................................1
Gi3_27359665_x8......................................................................gguucgauuccGggcgccg.....................1
Gi5_8488537_x8...................................................uucucaccAaacagauccg........................................ 1
Gi2_10495498_x8...................uccggUguaguauaacggc........................................................................1
Gi2_10477890_x8......................................................................gguuUgauucccggcgccg.....................1
Gi1_3948949_x8...................uccggcguaguauaacAgcua...................................................................... 1
```

```
Gi2_10486098_x8.....................................................................gguucgaCucccggcgccgg....................1
Gi3_27362081_x8......................................................................guucgauucccggcgcGgga...................1
Gi2_10472362_x8.....................................................................gguucgauucccAgcgccgga...................1
Gi2_10486738_x8.....................................................................gguucgauucccggcgGcggagc.................1
Gi3_27359369_x8......................................................................guucgauucccggcgccggaAc.................1
Gi3_27318697_x8................................................gguucucacccaacagauAcg........................................1
Gi1_3952357_x8................................................gguucucacccaacagauAcg........................................ 1
Gi3_27363793_x8......................................................................guucgauucccggcgGcgga...................1
Gi2_10492602_x8...................................................ucucacccaacagauccg........................................0
Gi1_3943421_x8.....................................................................gguucgauucccggcgccggU................... 1
Gi5_8493593_x8.....................................................................gguucgauucccggcgUcgga................... 1
Gi5_8485265_x8...................uccggcguaguauGacggcuag.................................................................... 1
Gi2_10479466_x8......................................................................guucgauucccCgcgccggagc.................1
Gi2_10483890_x8.....................................................................ggCucgauucccggcgccgga...................1
Gi1_3944557_x8................................................gguucucacccaacagGuccg........................................ 1
Gi2_10469098_x8.....................................................................gguucgauucccggcgccA.....................1
Gi2_10494178_x8.....................................................................gguucgauucccggcAccggag..................1
Gi5_8483649_x8.......................gcguaguauaacggcuaga................................................................... 0
Gi3_27320833_x8.........................................................................cgauucccggcgccggCgc.................1
Gi3_27324993_x8................................................ggAucucacccaacagauccg........................................1
Gi5_8493065_x8.....................................................................gguucgauucUcggcgccggagc................. 1
Gi2_10472842_x8......................................................................guucgauuNccggcgccgga...................1
Gi1_3951533_x8...................uGcggcguaguauaacggc....................................................................... 1
Gi3_27346385_x8......................................................................guucgauucccggcgccCgagc.................1
Gi1_3945037_x8.....................................................................gguucgauucccggcgccggG................... 1
Gi3_27361169_x8...................uccggcUuaguauaacggcuaga...................................................................1
Gi2_10494842_x8........................................................................ucgauucccggcgccggGgc.................1
Gi2_10475530_x8...................uccggcguaguauaacggcuaAaau.................................................................1
Gi5_8477473_x8...................uccAgcguaguauaacggc....................................................................... 1
Gi2_10487194_x8.......................................................................Gucgauucccggcgccgg....................1
Gi5_8486761_x8...................uccggcguaguCuaacggcuaga................................................................... 1
Gi2_10487810_x8......................................................................guCcgauucccggcgccggag..................1
Gi3_27341601_x8...................uccggcguaguauGacggcuagaau.................................................................1
Gi3_27328017_x8......................................................................guucgaGucccggcgccgga...................1
Gi5_8487297_x8......................................................................guucgauuccAggcgccg..................... 1
Gi4_525296_x8 .....................................................................gUuucgauucccggcgccggagc................. 1
Gi2_10481602_x8...................uccggcguaguauaacggcuagU...................................................................1
Gi5_8494881_x8...................uccggcguaguauaaAggcuag.................................................................... 1
Gi3_27332217_x8..................................................uucucacccaacagGuccgg.......................................1
Gi5_8488777_x8...................uccggcguCguauaacgg........................................................................ 1
Gi3_27366841_x8...................uccgUcguaguauaacggcuagaau.................................................................1
Gi1_3943365_x8.....................................................................gguucgauucccggcgccggagU................. 1
Gi2_10495458_x8...................uccggUguaguauaacgg........................................................................1
Gi2_10471362_x8...........................................................aacagauccggguucgauucccggcgccgg....................0
Gi3_27342481_x8.....................................................................gguucgauuccAggcgccg.....................1
Gi3_27335497_x8.........................................................................cgUuucccggcgccggagc.................1
Gi2_10467634_x8......................................................................guucgauucccgUcgccggagc.................1
```

```
Gi5_8486097_x8.....................................................................guuUgauucccggcgccggagc................. 1
Gi1_3950797_x8...............................................gAuucucacccaacagauccg........................................ 1
Gi3_27317057_x8................................................gGucucacccaacagauccggg......................................1
Gi2_10464722_x8....................................................................gguucgGuucccggcgccg.....................1
Gi1_3954469_x8................................................guucucaccUaacagauccg........................................ 1
Gi1_3943637_x8...................uccggcguaguauaacggUuag................................................................... 1
Gi5_8499257_x8...............................................gguucuUacccaacagauccg........................................ 1
Gi2_10469946_x8.....................................................................guucgUuucccggcgccgg....................1
Gi3_27369817_x8...................ucGggcguaguauaacggcua....................................................................1
Gi2_10488730_x8....................................................................gguucgauucccggcAccgg....................1
Gi5_8496129_x8....................................................................ggCucgauucccggcgccgg.................... 1
Gi3_27366081_x8...................uccgAcguaguauaacggcua....................................................................1
Gi2_10481186_x8....................................................................gguucgauucUcggcgccggag..................1
Gi2_10486802_x8.......................................................................ucgauucUcggcgccggagc.................1
Gi1_3950469_x8...................uccggcguaguauaacggcuaAaau................................................................ 1
Gi2_10472418_x8....................................................................gguCcgauucccggcgccggag..................1
Gi1_3946485_x8...............................................gguucucacccCacagauccg........................................ 1
Gi5_8497897_x8.....................................................................guucgGuucccggcgccggagc................. 1
Gi5_8481825_x8....................................................................ggCucgauucccggcgccggag.................. 1
Gi1_3949389_x8...............................................gguucucacccaGcagauccg........................................ 1
Gi1_3954109_x8......................gUcguaguauaacggcuag................................................................... 1
Gi5_8498865_x8.....................................................................guucgUuucccggcgccg..................... 1
Gi3_27347513_x8...................uccggcguaguauaaAgg.......................................................................1
Gi1_3946141_x8....................................................................gguCcgauucccggcgccgg.................... 1
Gi5_8496041_x8....................................................................gguucgauucccggcgAcgga................... 1
Gi5_8482833_x8...................uccggcguaguCuaacgg....................................................................... 1
Gi5_8479385_x8...................uccggcguaguauCacggcuagaau................................................................ 1
Gi4_526408_x8 ....................................................................gguucgauucccggcgccgga................... 0
Gi2_10478338_x8....................................................................gguucgauucccggAgcc......................1
Gi1_3955573_x8...................uccggcguaguauaacggcuaAa.................................................................. 1
Gi3_27328089_x8.....................................................................guucgauucAcggcgccgg....................1
Gi3_27360993_x8.....................................................................guucgauucccggcgccggUg..................1
Gi5_8493825_x8....................................................................ggGucgauucccggcgccgga................... 1
Gi2_10487602_x8.....................................................................guucgauuccAggcgccgg....................1
Gi1_3943093_x8..................................................ucucGcccaacagauccg........................................ 1
Gi3_27337049_x8................................................guucucaccUaacagauccg........................................1
Gi2_10495786_x8.....................................................................guucgauucAcggcgccggagc.................1
Gi1_3947829_x8....................................................................gguuUgauucccggcgccgg.................... 1
Gi1_3955053_x8....................................................................gguucgauucccggcgccggagcA................ 1
Gi3_27320697_x8.....................................................................guucgauucccggGgccgg....................1
Gi3_27323897_x8..............................................ugguucucacccaacagGuccg........................................1
Gi2_10475850_x8....................................................................gguucAauucccggcgccgga...................1
Gi5_8476993_x8.............................................................agauccggNuucgauuccc............................ 1
Gi4_524184_x8 ................................................guucucacccaacagauccg........................................ 0
Gi2_10497218_x8.....................................................................guucgauucccAgcgccgga...................1
Gi2_10462786_x8...................ucUggcguaguauaacggcuag...................................................................1
Gi3_27356329_x8.........................................................................gauucccAgcgccggagc.................1
```

```
Gi5_8482345_x8......................................................................gAuucgauucccggcgcc....................... 1
Gi5_8483369_x8...................uccggcguaguauaaUggcuagaau................................................................... 1
Gi2_10488106_x8.................................................gguucucacccGacagauccg.........................................1
Gi5_8493233_x8......................................................................gCuucgauucccggcgccgg..................... 1
Gi3_27341417_x8...................uGcggcguaguauaacggcu........................................................................1
Gi3_27364521_x8......................................................................gguucgauucccggAgccggag...................1
Gi3_27330617_x8................................................ugguucucGcccaacagauccg.........................................1
Gi5_8488025_x8......................................................................ggAucgauucccggcgccgga.................... 1
Gi3_27374497_x8.......................................................................gAucgauucccggcgccggag...................1
Gi2_10476746_x8......................................................................gguucgauNcccggcgccgg.....................1
Gi5_8491289_x8...................uccggcguaguauCacggc......................................................................... 1
Gi1_3949949_x8...................uccgAcguaguauaacgg.......................................................................... 1
Gi3_27409758_x7.........................................................................ucgGuucccggcgccggagc..................1
Gi5_8511635_x7..................................................guucucacNcaacagauccg......................................... 1
Gi2_10522161_x7......................................................................gguCcgauucccggcgccg......................1
Gi5_8529464_x7......................................................................Uguucgauucccggcgccggagcguuu.............. 1
Gi5_8527224_x7...................uccAgcguaguauaacggcuaga..................................................................... 1
Gi2_10516162_x7......................................................................Cguucgauucccggcgccgg.....................1
Gi2_10537610_x7...................uUcggcguaguauaacggcuag......................................................................1
Gi5_8506252_x7......................................................................gguucgauucccggcgccgAagc.................. 1
Gi2_10527012_x7...................uccggcguaguauaaUggc.........................................................................1
Gi1_3961238_x7..................................................guCcucacccaacagauccg......................................... 1
Gi5_8530668_x7....................................................Ccucacccaacagauccg......................................... 1
Gi3_27445290_x7.......................................................................guucgauucccggcgccCga....................1
Gi1_3967139_x7...................................................uucucacccaacagaAccg......................................... 1
Gi2_10525598_x7......................................................................gguucgauucccggcgccC......................1
Gi1_3967965_x7.......................................................................guucgauucccggcgccUg..................... 1
G1a_5688204_x7.......................................................................gGucgauucccggcgccggagc.................. 1
Gi1_3973075_x7....................ccggcguaguauaacggcuagaau................................................................... 0
Gi5_8504649_x7................................................ugguucucGcccaacagauccg......................................... 1
Gi1_3962540_x7......................................................................gguAcgauucccggcgccgg..................... 1
Gi3_27426285_x7.......................................................................guucgauuccUggcgccg......................1
Gi2_10499586_x7......................................................................gguucgauucccggcgUcggag...................1
Gi2_10534873_x7.......................................................................guucCauucccggcgccg......................1
Gi2_10501994_x7.......................................................................guucgCuucccggcgccgg.....................1
Gi3_27437044_x7...................uccggcguaguUuaacgg..........................................................................1
Gi3_27379581_x7......................................................................gguucgauucccggcgUc.......................1
Gi1_3963688_x7.......................................................................guuAgauucccggcgccgg..................... 1
Gi5_8528162_x7...................uccgUcguaguauaacggcuagaau................................................................... 1
Gi3_27425942_x7.......................................................................guucgauucccggcgccggagcgC................1
Gi5_8530850_x7................................................ugguucucaccUaacagauccg......................................... 1
Gi1_3959887_x7...................uccggcguaguauaacggcuagC..................................................................... 1
Gi5_8515569_x7.......................................................................guucgauucccggUgccgga.................... 1
Gi1_3968819_x7..................................................guGcucacccaacagauccg......................................... 1
Gi3_27427447_x7................................................ugguucucacAcaacagauc...........................................1
Gi2_10529371_x7......................................................................gguucgaCucccggcgccggag...................1
Gi3_27423527_x7..........................................................................cgauucccNgcgccggagc..................1
```

```
Gi3_27431668_x7..................................................uucucaccAaacagauccg........................................1
Gi3_27382640_x7......................................................................guucgauucccggcgcUg.....................1
Gi2_10535055_x7........................................................................ucgauuUccggcgccggagc.................1
Gi1_3971206_x7..................................................uAcucacccaacagauccgg....................................... 1
Gi3_27416478_x7...................uccggcguaguauaacgAcuagaa..................................................................1
Gi1_3973922_x7.......................................................................uuUgauucccggcgccgg.................... 1
Gi5_8529632_x7.....................................................................ggAucgauucccggcgccgg.................... 1
Gi2_10538863_x7...................uccggcguaguauaacggcuGgaa..................................................................1
Gi5_8521813_x7.....................................................................gguucgauCcccggcgccg..................... 1
Gi2_10525038_x7......................................................................guucUauucccggcgccg.....................1
Gi2_10525304_x7.....................................................................gguucgauucccggcgGcg.....................1
Gi2_10503198_x7................................................gguucucacccaacagaucUg........................................1
Gi5_8503200_x7......................................................................guucgCuucccggcgccg..................... 1
Gi1_3961980_x7.....................................................................gguuGgauucccggcgccgg.................... 1
Gi3_27446438_x7......................................................................guucgauuAccggcgccgg....................1
Gi1_3967720_x7...................uccggcguaguauaacggUua..................................................................... 1
Gi5_8511859_x7.....................................................................gguucgauucccggcgAcg..................... 1
Gi3_27411417_x7......................................................................guucgauucAcggcgccggagc.................1
Gi2_10520656_x7..................................................uucucacccaacCgauccg........................................1
Gi3_27381044_x7......................................................................guucgauucccggcgcAggag..................1
Gi3_27447943_x7.......................................................................uucgauucNcggcgccggagc.................1
Gi1_3960720_x7...................uccggcguagCauaacggcua..................................................................... 1
Gi3_27447873_x7.....................................................................gguucgauucccggcgccgCa...................1
Gi5_8506868_x7.....................................................................gguucgCuucccggcgccgga................... 1
Gi3_27411053_x7...................uccggcguaAuauaacggc.......................................................................1
Gi5_8521939_x7......................................................................guucgauucccggcgGcg..................... 1
Gi2_10514335_x7...................uccggcguNguauaacgg........................................................................1
Gi3_27424451_x7....................................................cucacccaNcagauccgg.......................................1
Gi3_27407525_x7......................................................................guucgauucccggcgccggCg..................1
Gi5_8508282_x7.....................................................................gguucgauucccgAcgccgga................... 1
Gi3_27407336_x7...................uccggcguaUuauaacggcuaga...................................................................1
Gi1_3967559_x7...................Accggcguaguauaacgg........................................................................ 1
Gi3_27432732_x7...................uccggcguGguauaacggcuagaa..................................................................1
Gi5_8514484_x7.....................................................................gguucgauucccggcAccg..................... 1
Gi4_526928_x7 ...................uccggcguaguauaacgg........................................................................ 0
Gi1_3972557_x7...................uccggcguaguauaacAgc....................................................................... 1
Gi5_8530052_x7...................uccggcguaguauaacggGuag.................................................................... 1
Gi3_27393896_x7...............................................ugguCcucacccaacagauccg........................................1
Gi5_8503165_x7......................................................................guucgauuAccggcgccg..................... 1
Gi2_10507881_x7......................................................................guCcgauucccggcgccgga...................1
Gi5_8510725_x7...............................................ugguucucacccaacGgauccg........................................ 1
Gi1_3968056_x7..................................................uucucacccaacCgauccgg....................................... 1
Gi3_27436547_x7...................uccggcguaAuauaacggcua.....................................................................1
Gi2_10502547_x7...................uccggcguaguauaacggcuagCau.................................................................1
Gi2_10508679_x7...................uccggcguNguauaacggcuag....................................................................1
Gi1_3965249_x7..................................................uucucacccUacagauccgg....................................... 1
Gi3_27393791_x7...................uccgCcguaguauaacggc.......................................................................1
```

Gi2_10514692_x7..guuAgauucccggcgccgg.....................1
Gi2_10515833_x7...gguucgauuccUggcgccg......................1
Gi1_3957976_x7..Cuucgauucccggcgccgga.................... 1
Gi4_528594_x7 ....uccggcguaguauaacggcuGga... 1
Gi3_27449252_x7..guucgauucccggcgccggaCc..................1
Gi2_10509813_x7..guuUgauucccggcgccgga....................1
Gi1_3959257_x7...uCguucucacccaacagauccg... 1
Gi5_8523892_x7..guucgauucccggcgccAgagc.................. 1
Gi5_8505391_x7.....................cUgcguaguauaacggcuag... 1
Gi5_8515625_x7..gguucucacccaacCgauccg... 1
Gi2_10537638_x7...................uccgAcguaguauaacggc..1
Gi5_8524718_x7...................Cccggcguaguauaacggcuagaau.. 1
Gi3_27420545_x7...gguucgauGcccggcgccggagc..................1
Gi2_10501630_x7..guucgauucccggUgccggag...................1
Gi5_8505811_x7..guucgauucccgAcgccg...................... 1
Gi3_27451051_x7...gguCcgauucccggcgcc.......................1
Gi5_8525586_x7...guucAcacccaacagauccg... 1
Gi1_3970065_x7...................Gccggcguaguauaacggcuagaa... 1
Gi2_10536210_x7...gguucgauucccggUgccgg.....................1
Gi5_8507155_x7..Gcgauucccggcgccggagc.................. 1
Gi1_3972865_x7...ugguucucacccaCcagauccg... 1
Gi1_3968938_x7...................uccggcguaguauaacggcCaga.. 1
Gi5_8526216_x7..cucacUcaacagauccgg.. 1
Gi5_8503221_x7...................uccggcguCguauaacggcuaga.. 1
Gi1_3973369_x7...gguucgauucccggcgccggCgc.................. 1
Gi1_3958641_x7...gguucgauucccggcAccggagc.................. 1
Gi5_8512076_x7..gguuGucacccaacagauccg... 1
Gi2_10512956_x7...................uccggcguaguauaacggcuaA...1
Gi3_27402597_x7..uucucacccaacagauccgC..1
Gi5_8516486_x7..guucgauucccggcgccggC.................... 1
Gi5_8507519_x7...................uccggcguaguauaGcggcuagaa... 1
Gi5_8516255_x7...................uccggcguaguauaCcggcua.. 1
Gi3_27445787_x7...gguucgauucccggcgcUg......................1
Gi5_8519342_x7...ugguucucacccaCcagauccg... 1
Gi5_8521162_x7...gguucgauucccggcgccggUgc.................. 1
Gi3_27405733_x7..guucgaGucccggcgccgg.....................1
Gi5_8519272_x7...gguCcgauucccggcgccgg..................... 1
Gi3_27394498_x7...gguucgauucccggAgccgg.....................1
Gi2_10530001_x7...................uccggcguaguauaCcggcuaga..1
Gi3_27386826_x7...guucucacccaacagaucUg...1
Gi1_3958452_x7..uucucacccaacagaucAgg.. 1
Gi3_27385167_x7...guucucacccaacagaCccg...1
Gi1_3960223_x7...ugguucucacccaacGgauccg... 1
Gi3_27398712_x7..uucucacccaacGgauccgg..1
Gi3_27411676_x7...................uccggcguaguauGacggcua..1
Gi3_27381107_x7...gguucgauucccggcgUcgg.....................1
Gi5_8528449_x7...gguucgauucccAgcgcc....................... 1

Gi3_27414959_x7...gguucgCuucccggcgccg.....................1
Gi2_10513705_x7...................Accggcguaguauaacggcuaga...1
Gi3_27418193_x7...Nguucgauucccggcgccggagc.................1
Gi1_3963856_x7...ugguucucacAcaacagauccgg....................................... 1
Gi1_3960671_x7..guucCauucccggcgccgg.................... 1
Gi3_27416835_x7...................uccggAguaguauaacggcuagaa..1
Gi5_8516479_x7...guucucacccaacagGuccgg....................................... 1
Gi2_10525864_x7...................uccggcgCaguauaacggc...1
Gi3_27382605_x7...uucgauucccggcgccAgagc.................1
Gi2_10504444_x7...................uccgUcguaguauaacgg..1
Gi2_10517387_x7...ggGucgauucccggcgccg.....................1
Gi1_3973243_x7..uucucacccaacagauccAg....................................... 1
Gi2_10508567_x7......................Ugcguaguauaacggcuaga...1
Gi3_27390683_x7...Cuucucacccaacagauccg..1
Gi3_27446081_x7...gguucgauuccAggcgcc......................1
Gi2_10499642_x7...Gucgauucccggcgccggagc.................1
Gi5_8505384_x7......................gUcguaguauaacggcuaga... 1
Gi2_10503373_x7..ucgaCucccggcgccggagc.................1
Gi3_27453277_x7...gguucgauucUcggcgccgg....................1
Gi2_10537645_x7..uucucaccNaacagauccg..1
G1a_5682051_x7...................uccggcguGguauaacggcua... 1
Gi2_10520985_x7..gCuucucacccaacagauccg..1
Gi3_27407007_x7...................ucUggcguaguauaacggcu..1
Gi1_3969239_x7..gguucucacccaacagaucAg.. 1
Gi1_3968728_x7...................uccggcgCaguauaacggcua... 1
Gi3_27427111_x7..guucgauucccggcgcGggagc.................1
Gi3_27448104_x7..ucgaCucccggcgccggagc.................1
Gi5_8512531_x7..uGcucacccaacagauccggguuc................................... 1
Gi2_10517688_x7...gguucgauCcccggcgccggag..................1
Gi2_10505571_x7...................uccggcguNguauaacggcuagaau...1
Gi5_8511789_x7...guuUucacccaacagauccg.. 1
Gi1_3958823_x7..uucucacccaacagaCccg.. 1
Gi3_27386308_x7...gguucgauucccggcUccggagc.................1
Gi3_27451499_x7..guucgauucccggUgccg.....................1
Gi5_8511397_x7..gguuUucacccaacagauccg.. 1
Gi5_8512132_x7..guuAgauucccggcgccgg.................... 1
Gi3_27423296_x7..guuUgauucccggcgccg.....................1
Gi3_27431339_x7..uGgguucucacccaacagaucc...1
Gi3_27447810_x7...gguucgauucUcggcgccg.....................1
Gi2_10529735_x7..guucUauucccggcgccggag..................1
Gi1_3972046_x7..uucucacccaGcagauccgg....................................... 1
Gi3_27448888_x7...gguucgauucccggcgccggagG.................1
Gi3_27432172_x7..uucucacccaacagaucUg..1
Gi5_8516521_x7...................uccggcguaguCuaacggc... 1
Gi2_10509617_x7..guucgauuccUggcgccgga...................1
G1a_5701007_x7...gguucgauucccggUgcc...................... 1
Gi3_27422820_x7...................uccggcguaguauUacggcua...1

Gi3_27442532_x7...gguucgauucAcggcgccg......................1

Gi5_8507673_x7..guucAauucccggcgccggagc.................. 1

Gi2_10524779_x7...gguucgauucccggUgccggag...................1

Gi5_8514904_x7..Auucgauucccggcgccggagc.................. 1

Gi3_27436477_x7...................uccggcguaguaCaacggcuagaau..1

Gi3_27434055_x7...gguucgauucccggcgccggagcg.................0

Gi3_27423996_x7...guucucacccCacagauccgg..1

Gi5_8512580_x7...gguucgGuucccggcgccggag................... 1

Gi5_8530185_x7...Uguucgauucccggcgccggagcguu............... 1

Gi5_8518824_x7...................uccggcguaguCuaacggcuagaau.. 1

Gi3_27435749_x7..uucuUacccaacagauccg...1

Gi2_10522343_x7...uGcgauucccggcgccggagc..................1

Gi3_27408729_x7...ugguucCcacccaacagauccg...1

Gi3_27386875_x7..guucgauucccggcgccggaA...................1

Gi3_27407679_x7...gguucgauucccggcgcGggag...................1

Gi3_27395842_x7...................ucNggcguaguauaacggcuaga..1

Gi3_27444058_x7..gguAcucacccaacagauccg...1

Gi2_10512207_x7..guucgauucccggcgccCgagc..................1

Gi3_27456651_x7...................uccAgcguaguauaacggcuagaau..1

Gi5_8527161_x7..Ugguucgauucccggcgccggagc.................. 1

Gi5_8507288_x7...guucucacccaacagauAcg... 1

Gi1_3967475_x7...ggCucgauucccggcgccgga.................... 1

Gi2_10534418_x7...................uccggcguaguaCaacggcua..1

Gi2_10523505_x7...uGcgauucccggcgccgg.....................1

Gi3_27441874_x7..guucAauucccggcgccg......................1

Gi3_27396451_x7..guucgCuucccggcgccg......................1

Gi2_10508350_x7...gguucgauucccUgcgccggag...................1

Gi3_27425508_x7..guCcgauucccggcgccg......................1

Gi5_8523577_x7..guucgauucccggcgccUg..................... 1

Gi1_3967076_x7...gguucgauucccggcgccggGgc.................. 1

Gi2_10515805_x7...................uccggcguaguauaacggcuaUa..1

Gi3_27444233_x7..guucgauucAcggcgccgga....................1

Gi5_8527399_x7...................uccggcguaguauaacggcCagaa... 1

G1a_5682142_x7...gguucgauNcccggcgccggagc.................. 1

Gi1_3962561_x7...ggAucgauucccggcgccgg..................... 1

Gi5_8518145_x7...................uccggcAuaguauaacggcuaga.. 1

Gi2_10535097_x7...................uccggcguaguauaacggcCagaau..1

Gi5_8504460_x7...gguuUgauucccggcgccg...................... 1

Gi3_27416485_x7...gguucgauucccggAgcc.......................1

Gi1_3970982_x7...Auucucacccaacagauccgg.. 1

G1a_5687742_x7...Gcucacccaacagauccg... 1

Gi3_27419789_x7...................uccCgcguaguauaacggcuaga..1

Gi5_8521729_x7..gCuucgauucccggcgccggag.................... 1

Gi3_27405824_x7...gguucgauucccggcgGcgga....................1

Gi3_27422295_x7...................uccggcguaAuauaacgg...1
[truncated: 585,292 more chars]
